# Supplementary material for: Ketones from aldehydes via alkyl C(sp3)−H functionalization under photoredox cooperative NHC/palladium catalysis
Source: Nat Commun. 2023 Jul 8;14:4044. doi: 10.1038/s41467-023-39707-8 (PMC10329650; doi:10.1038/s41467-023-39707-8)
Supplement: Supplementary file 1 — Supplementary Information [file 41467_2023_39707_MOESM1_ESM.pdf]

Supplementary Information

Ketones from Aldehydes via Alkyl C(sp^3)−H Functionalization under Photoredox Cooperative NHC/Palladium Catalysis

Hai-Ying Wang [†,‡], Xin-Han Wang [†,‡], Bang-An Zhou [†,‡], Chun-Lin Zhang*[,†] and Song Ye*[,†,‡]

[†] Beijing National Laboratory for Molecular Sciences, CAS Key Laboratory of Molecular Recognition and Function, CAS Research/Education Center for Excellence in Molecular Sciences, Institute of Chemistry, Chinese Academy of Sciences, Beijing 100190, China;

[‡] University of Chinese Academy of Sciences, Beijing 100049, China;

*Corresponding author. Email: songye@iccas.ac.cn. zhangchunlin@iccas.ac.cn

Table of Contents

1. Supplementary Notes S3

2. Supplementary Methods S4

    2.1 Preparation of starting materials S4

    2.2 Optimization of reaction conditions S5

    2.3 General procedure for substrate scope S9

    2.4 Gram-scale synthesis and chemical transformation of products S62

    2.5 Mechanistic studies S68

3. Supplementary Figures S74

4. Supplementary Reference S263

1. Supplementary Notes

Unless otherwise noted, all starting materials were obtained from commercial supplies and directly used without further purification unless otherwise stated. Unless otherwise indicated, all reactions were carried out under N_2 atmosphere with magnetic stirring. Anhydrous THF and diethyl ether were distilled from sodium and benzophenone. Anhydrous CH_2Cl_2 was distilled from CaH_2. Dichloroethane, trifluorotoluene and ethyl acetate were purchased as dry solvents from J&K commercial supplier and stored over molecular sieves (4 Å). PreNHCs was synthesized according to literature.[1]

Analytical thin layer chromatography was carried out with silica gel pre-coated glass plates (TLC-Silica gel GF254, coating thickness: 0.25 mm) purchased from Xinnuo Chemical (Yantai, China). Visualization was accomplished with short wave UV light (254 nm, 365 nm) and/or $KMnO_4$ staining solutions followed by heating. Column chromatograph was performed on silica gel 200~300 mesh. All ^1H, ^{13}C and 2D spectra were recorded on a Bruker AV 300, 400 and 500 spectrometers. Chemical shifts were reported in parts per million (ppm, δ), and the residual solvent peak was used as internal reference. ^1H and ^{13}C NMR Spectroscopy splitting patterns were designated as singlet (s), doublet (d), triplet (t), quartet (q). Splitting patterns that could not be interpreted or easily visualized were designated as multiplet (m) or broad (br). High-resolution mass spectra (HRMS) were obtained with the mass analyzer of an orbitrap. The calculated values are based on the most abundant isotope. Infrared spectra were recorded on a JASCO FT/IR-480 spectrophotometer and reported as wave number (cm^{-1}). UV/vis absorption spectra were recorded on a Jasco V-650 spectrophotometer, equipped with a temperature control unit at 25 °C, and the samples were measured in Hellma fluorescence QS quartz cuvettes (chamber volume = 3.0 mL) fitted with a PTFE stopper.

2. Supplementary Methods

2.1 Preparation of starting materials

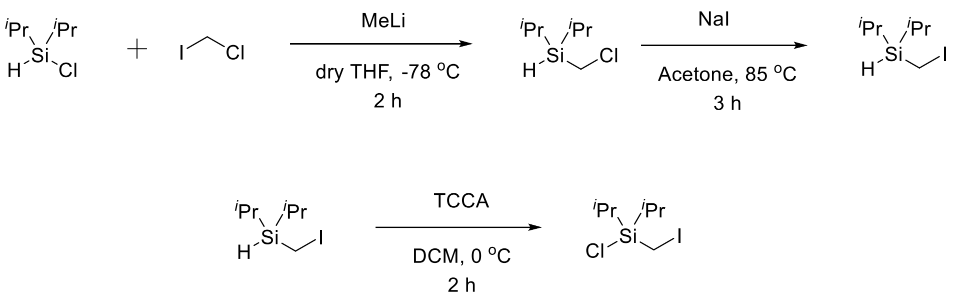

To a solution of chlorodiisopropylsilane (50 mmol) and chloroiodomethane (1.5 equiv) in THF (100 mL) was added a solution of MeLi (1.5 equiv) dropwise at -78 °C under N_2. The reaction mixture was stirred at -78 °C for 2 h and then allowed to warm to room temperature before quenching with saturated NH_4Cl solution. The aqueous layer was extracted with petroleum ether. The combined organic layer was dried over $MgSO_4$ and concentrated in vacuo. The crude product, (chloromethyl)diisopropylsilane, was used without further purification.

To a solution of NaI (5 equiv) in acetone (100 mL) was added crude (chloromethyl)diisopropylsilane. The reaction mixture was refluxed at 85 °C for 3 h. The reaction allowed to cool to room temperature before quenching with saturated solution of $Na_2S_2O_3$.The aqueous layer was extracted with petroleum ether. The combined organic layer was dried over $MgSO_4$ and concentrated in vacuo. The crude product was used for the next step without further purification.

To a solution of TCCA (0.36 equiv) in dry DCM (50 mL) under N_2 was added crude (iodomethyl)diisopropylsilane (1 equiv) in DCM (20 mL) dropwise at 0 °C for 2 h. The mixture was allowed to warm to room temperature and then filtered through celite and concentrated. The residue was then dissolved in petroleum ether and re-filtered through celite and then concentrated to yield chloro(iodomethyl)diisopropylsilane as a pink/purple oil. Yield = 65% over three steps.[2]

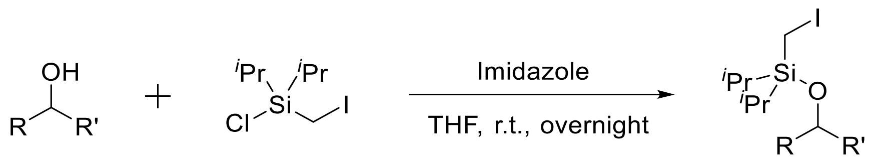

To a stirred solution of alcohol (1 equiv.) and imidazole (2 equiv.) in THF, chloro(iodomethyl)diisopropylsilane (1.2 equiv.) was added at room temperature under N_2 atmosphere. After being stirred until completion of the reaction as judged by TLC analysis. The filtrate was then concentrated under reduced pressure. The residue was purified by column chromatography in petroleum ether to afford the desired product alkyl silyl ether.[2]

2.2 Optimization of reaction conditions

2.2.1 Optimization of reaction conditions for heteroaromatic aldehydes

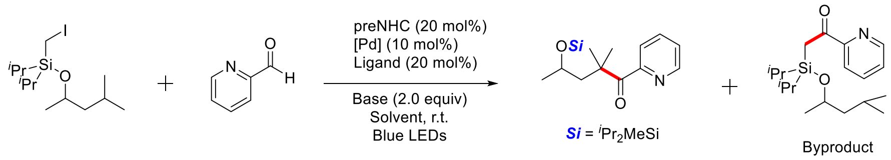

1a 2a 3a 4a

Typical procedure: A 4 mL vial equipped with a stir bar was charged with preNHC (0.02 mmol), [Pd] (0.01 mmol), Ligand (0.02 mmol) and 0.5 mL of solvent. After stirring for 30 min in glove box, to the solution was added the base (0.2 mmol), picolinaldehyde **2a** (0.15 mmol), alkyl silyl ether **1a** (0.1 mmol), and 0.5 mL of solvent. The reaction mixture was removed from the glove box and stirred under 36W Blue LEDs at room temperature until the complete consumption of **1a** (generally 16 hours) monitored by TLC analysis. The reaction mixture was filtered through a small pad of silica and eluted with ethyl acetate. Then, the residue was analyzed by ^{1}H NMR using CH_2Br_2 as standard.

Supplementary Table 1. Optimization studies of preNHC[a]

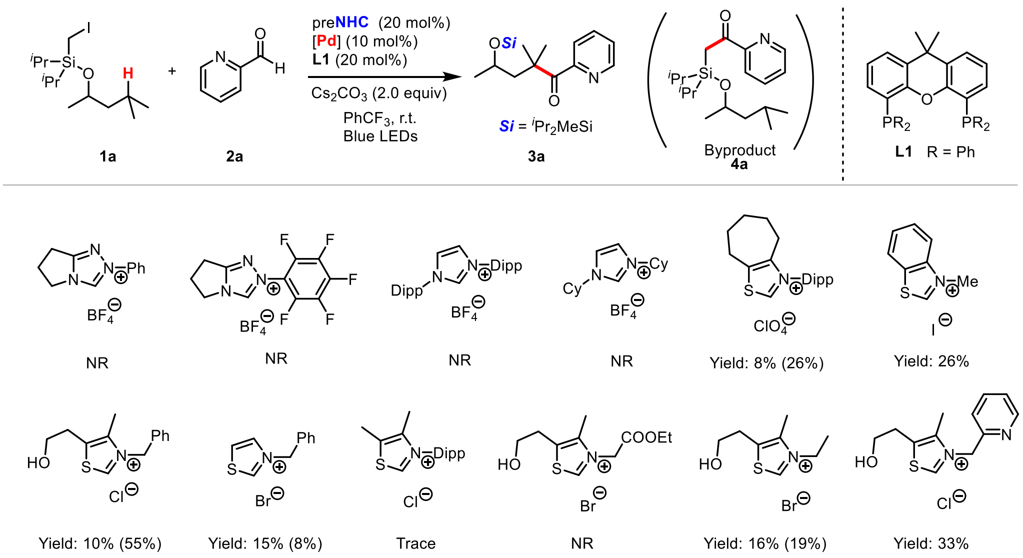

[a]Reaction conditions: **1a** (0.1 mmol), **2a** (1.5 equiv), $Pd(OAc)_2$ (10 mol%), **L1** (20 mol%), preNHC (20 mol%), Cs_2CO_3 (2.0 equiv), 1.0 mL $PhCF_3$, 36 W Blue LEDs, r.t., under N_2; the yield was determined by ^1H NMR using CH_2Br_2 as standard; the yield of **4a** was given in parentheses.

Supplementary Table 2. Optimization studies of other conditions[a]

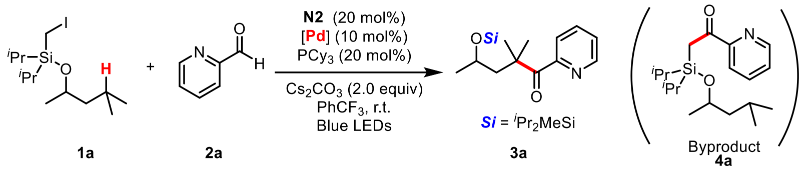

entry	Pd	base	solvent	**3a**[b] (%)	**4a**[c] (%)
1	$Pd(OAc)_2$	Cs_2CO_3	$PhCF_3$	67(69 [c])	0
2	$Pd(PPh_3)_4$	Cs_2CO_3	$PhCF_3$	39	0
3	$Pd(TFA)_2$	Cs_2CO_3	$PhCF_3$	19	14
4	$Pd_2(dba)_3$	Cs_2CO_3	$PhCF_3$	24	26
5	$PdCl_2$	Cs_2CO_3	$PhCF_3$	17	19
6	$PdCl_2(MeCN)_2$	Cs_2CO_3	$PhCF_3$	11	16
7	$Pd(C_5H_7O_2)_2$	Cs_2CO_3	$PhCF_3$	23	14
8	$Pd(OAc)_2$	$KHCO_3$	$PhCF_3$	0	0
9	$Pd(OAc)_2$	KO^tBu	$PhCF_3$	0	0
10	$Pd(OAc)_2$	K_3PO_4	$PhCF_3$	32	22
11	$Pd(OAc)_2$	KOAc	$PhCF_3$	0	0
12	$Pd(OAc)_2$	NaOH	$PhCF_3$	0	0
13	$Pd(OAc)_2$	K_2HPO_4	$PhCF_3$	0	0

14	$Pd(OAc)_2$	NEt_3	$PhCF_3$	0	0
15	$Pd(OAc)_2$	DBU	$PhCF_3$	29	14
16	$Pd(OAc)_2$	DBN	$PhCF_3$	22	0
17	$Pd(OAc)_2$	Li_2CO_3	$PhCF_3$	0	0
18	$Pd(OAc)_2$	Na_2CO_3	$PhCF_3$	0	0
19	$Pd(OAc)_2$	K_2CO_3	$PhCF_3$	32	10
20	$Pd(OAc)_2$	Rb_2CO_3	$PhCF_3$	0	0
21	$Pd(OAc)_2$	Cs_2CO_3	1,4-Dioxane	17	0
22	$Pd(OAc)_2$	Cs_2CO_3	Et_2O	9	0
23	$Pd(OAc)_2$	Cs_2CO_3	THF	12	23
24	$Pd(OAc)_2$	Cs_2CO_3	MTBE	35	0
25	$Pd(OAc)_2$	Cs_2CO_3	Ph_2O	0	0
26	$Pd(OAc)_2$	Cs_2CO_3	DCM	17	17
27	$Pd(OAc)_2$	Cs_2CO_3	DCE	19	0
28	$Pd(OAc)_2$	Cs_2CO_3	EA	20	0
29	$Pd(OAc)_2$	Cs_2CO_3	Acetone	0	0
30	$Pd(OAc)_2$	Cs_2CO_3	MeCN	7	12
31	$Pd(OAc)_2$	Cs_2CO_3	DMF	0	0
32	$Pd(OAc)_2$	Cs_2CO_3	DMSO	0	0
33	$Pd(OAc)_2$	Cs_2CO_3	PhCl	26	0
34	$Pd(OAc)_2$	Cs_2CO_3	PhOMe	0	0
35	/	Cs_2CO_3	$PhCF_3$	0	0
36[d]	$Pd(OAc)_2$	Cs_2CO_3	$PhCF_3$	20	11
37[e]	$Pd(OAc)_2$	Cs_2CO_3	$PhCF_3$	24	31
38[f]	$Pd(OAc)_2$	Cs_2CO_3	$PhCF_3$	29	19
39[g]	$Pd(OAc)_2$	Cs_2CO_3	$PhCF_3$	0	0
40[h]	$Pd(OAc)_2$	Cs_2CO_3	$PhCF_3$	13	0
41[i]	$Pd(OAc)_2$	Cs_2CO_3	$PhCF_3$	0	0
42[j]	$Pd(OAc)_2$	Cs_2CO_3	$PhCF_3$	29	0
43[k]	$Pd(OAc)_2$	Cs_2CO_3	$PhCF_3$	0	0
44[l]	$Pd(OAc)_2$	Cs_2CO_3	$PhCF_3$	25	0
45[m]	$Pd(OAc)_2$	Cs_2CO_3	$PhCF_3$	0	0
46[n]	$Pd(OAc)_2$	Cs_2CO_3	$PhCF_3$	13	0

a: reaction conditions: **1a** 0.1 mmol, **2a** 1.5 equiv, [Pd] 10 mol%, PCy_3 20 mol%, **N2** 20 mol%, base 2.0 equiv, 1.0 mL solvent, 36 W Blue LEDs, r.t., under N_2; b: the yield of **3a** determined by NMR using

CH_2Br_2 as standard; c: the yield of **4a** determined by NMR using CH_2Br_2 as standard; d: PCy_3 10 mol%; e: PCy_3 40 mol%; f: $Pd(OAc)_2$ 5 mol%; g: $Pd(OAc)_2$ 20 mol%; h: **N2** 10 mol%; i: **N2** 30 mol%; j: White LED; k: $AgBF_4$ 1.0 equiv; l: under air; m: w/o **N2**; n: w/o PCy_3; o: isolated yields.

2.2.2 Optimization of reaction conditions for common aromatic aldehydes

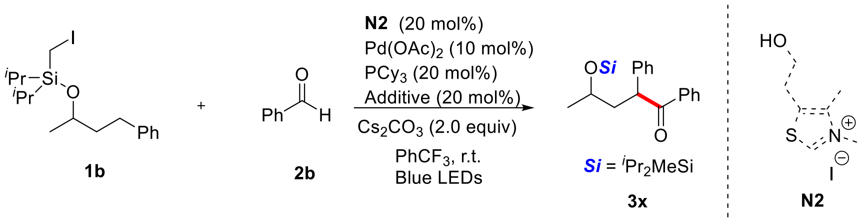

Typical procedure: A 4 mL vial equipped with a stir bar was charged with **N2** (0.02 mmol), $Pd(OAc)_2$ (0.01 mmol), PCy_3 (0.02 mmol), additive (0.02 mmol) and 0.5 mL of $PhCF_3$. After stirring for 30 min in glove box, to the solution was added the Cs_2CO_3 (0.2 mmol), benzaldehyde **2b** (0.15 mmol), alkyl silyl ether **1b** (0.1 mmol), and 0.5 mL of $PhCF_3$. The reaction mixture was removed from the glove box and stirred under 36W Blue LEDs at room temperature until the complete consumption of **1b** (generally 16 hours) monitored by TLC analysis. The reaction mixture was filtered through a small pad of silica and eluted with ethyl acetate. Then, the residue was analyzed by ^1H NMR using CH_2Br_2 as standard.

Supplementary Table 3. Optimization studies of the additive[a]

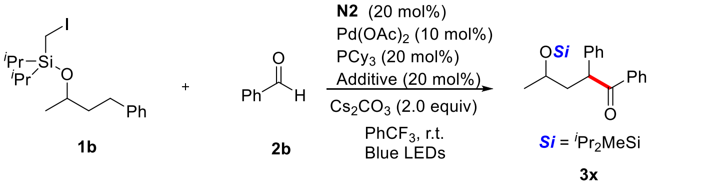

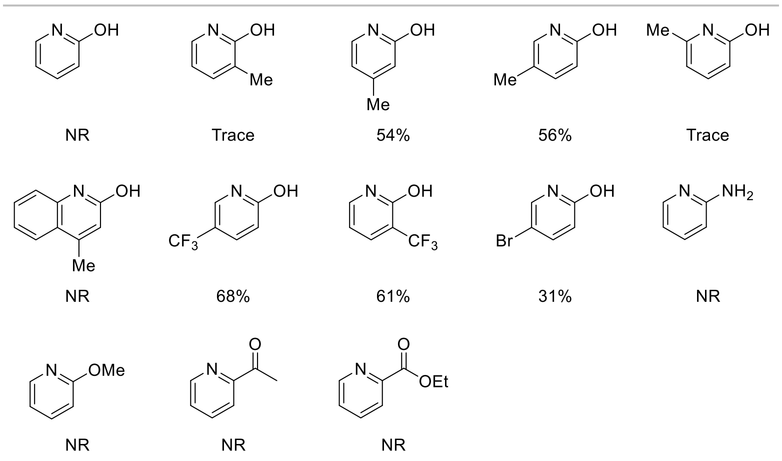

[a]The yield was determined by ^1H NMR using CH_2Br_2 as standard.

Supplementary Table 4. Attempts for the asymmetric variant of this reaction

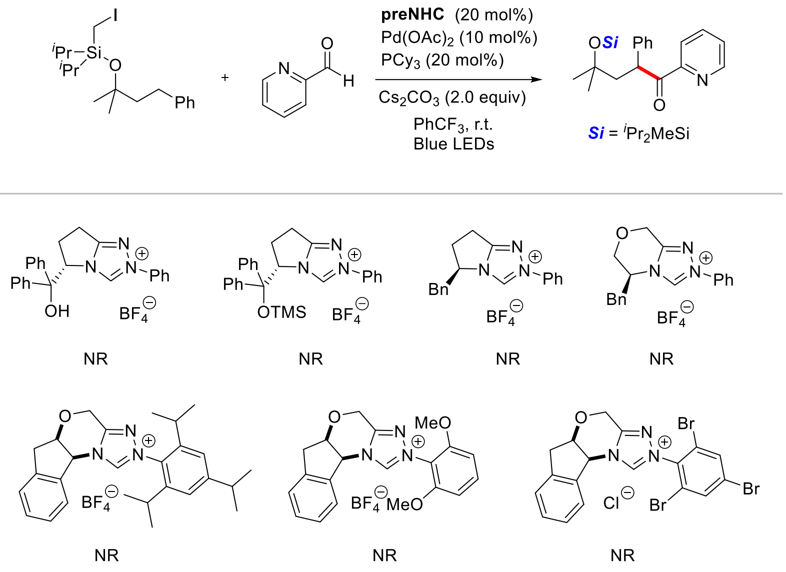

2.3 General procedure for substrate scope

2.3.1. Two-component reaction

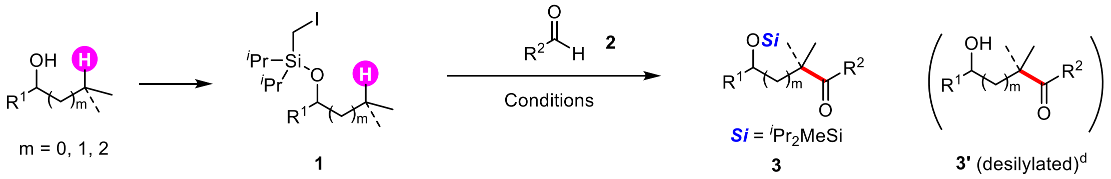

Typical procedure A: A 5 mL vial equipped with a stir bar was charged with **N2** (0.06 mmol), $Pd(OAc)_2$ (0.03 mmol), PCy_3 (0.06 mmol) and 2.0 mL of $PhCF_3$. After stirring for 30 min in glove box, to the solution was added Cs_2CO_3 (0.6 mmol), aldehydes **2** (0.45 mmol), silyl ether derived from varied alcohols **1** (0.3 mmol), and 1.0 mL of $PhCF_3$. The reaction mixture was removed from the glove box and stirred under 36W Blue LEDs at room temperature for 16 h. The solution was concentrated under reduced pressure, and purified by column chromatography on silica gel to afford the desired ketones **3**. Note: The reactions with benzaldehydes and enals use 5-trifluomethylpyridinone or 4- methylpyridinone (0.06 mmol) as additive.

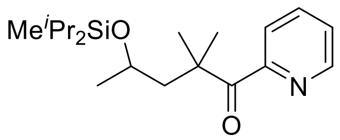

(why-08-167-1)

4-((diisopropyl(methyl)silyl)oxy)-2,2-dimethyl-1-(pyridin-2-yl)pentan-1-one (3a)

69.3 mg, 69% yield, Colorless oil, R_f = 0.7 (petroleum ether/ethyl acetate = 10:1).

^{1}H NMR (500 MHz, $CDCl_3$) δ 8.62 – 8.57 (m, 1H), 7.94 (d, *J* = 7.9 Hz, 1H), 7.75 (td, *J* = 7.8, 1.9 Hz, 1H), 7.35 – 7.30 (m, 1H), 3.98 – 3.92 (m, 1H), 2.76 (dd, *J* = 14.1, 7.7 Hz, 1H), 2.09 (dd, *J* = 14.1, 5.3 Hz, 1H), 1.42 (s, 3H), 1.39 (s, 3H), 1.02 (d, *J* = 6.1 Hz, 3H), 0.90 – 0.75 (m, 14H), -0.12 (s, 3H).

^{13}C NMR (126 MHz, $CDCl_3$) δ 205.4, 154.9, 147.5, 136.4, 125.5, 123.7, 66.6, 50.3, 46.5, 26.9 26.3, 24.8, 17.51, 17.46, 17.44, 17.41, 13.4, 13.3, -7.8.

IR (KBr) ν 2941, 2865, 1686, 1464, 1252, 1136, 1062, 998, 783, 743.

HRMS (ESI) m/z: Calc. For $C_{19}H_{34}NO_2Si$ ($[M+H]^+$) 336.2353, Found 336.2351.

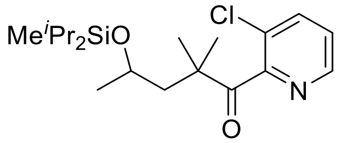

(why-08-173-6)

1-(3-chloropyridin-2-yl)-4-((diisopropyl(methyl)silyl)oxy)-2,2-dimethylpentan-1-one (3b)

32.1 mg, 29% yield, Colorless oil, R_f = 0.5 (petroleum ether/ethyl acetate = 10:1).

^{1}H NMR (500 MHz, $CDCl_3$) δ 8.31 (dd, *J* = 4.7, 1.4 Hz, 1H), 7.59 (dd, *J* = 8.2, 1.4 Hz, 1H), 7.13 (dd, *J* = 8.4, 4.6 Hz, 1H), 3.99 – 3.91 (m, 1H), 1.91 (dd, *J* = 14.1, 5.4 Hz, 1H), 1.82 (dd, *J* = 14.2, 6.0 Hz, 1H), 1.18 (s, 3H), 1.17 (s, 3H), 1.06 (d, *J* = 6.1 Hz, 3H), 0.89 – 0.70 (m, 14H), -0.12 (s, 3H).

^{13}C NMR (126 MHz, $CDCl_3$) δ 207.7, 155.8, 146.1, 137.7, 128.3, 124.6, 66.7, 49.1, 47.3, 26.2, 25.5, 24.5, 17.61, 17.57, 17.5, 13.6, 13.5, -7.3.

IR (KBr) ν 2941, 2865, 1700, 1463,1418, 1252, 1134, 1074, 996, 784, 735.

HRMS (ESI) m/z: Calc. For $C_{19}H_{33}NO_2ClSi$ ($[M+H]^+$) 370.1964 Found 370.1962.

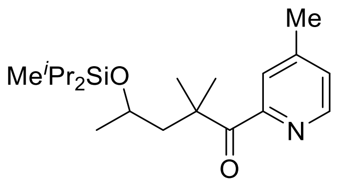

(why-08-175-1)

4-((diisopropyl(methyl)silyl)oxy)-2,2-dimethyl-1-(4-methylpyridin-2-yl)pentan-1-one (3c)

74.3 mg, 71% yield, Red oil, R_f = 0.8 (petroleum ether/ethyl acetate = 10:1).

^{1}H NMR (500 MHz, $CDCl_3$) δ 8.44 (d, *J* = 5.0 Hz, 1H), 7.75 (s, 1H), 7.15 (d, *J* = 5.3 Hz, 1H), 3.98 – 3.90 (m, 1H), 2.69 (dd, *J* = 14.2, 7.5 Hz, 1H), 2.37 (s, 3H), 2.12 (dd, *J* = 14.1, 5.4 Hz, 1H), 1.42 (s, 3H), 1.38 (s, 3H), 1.01 (d, *J* = 6.1 Hz, 3H), 0.89 – 0.82 (m, 14H), -0.10 (s, 3H).

^{13}C NMR (126 MHz, $CDCl_3$) δ 205.9, 154.8, 147.5, 147.4, 126.4, 124.5, 66.6, 50.2, 46.6, 26.9, 26.4, 24.8, 21.1, 17.48, 17.46, 17.45, 17.42, 13.42, 13.37, -7.7.

IR (KBr) ν 2940, 2865, 1683, 1597, 1461, 1252, 1062, 996, 883, 784.

HRMS (ESI) m/z: Calc. For $C_{20}H_{36}NO_2Si$ ($[M+H]^+$) 350.2510, Found 350.2506.

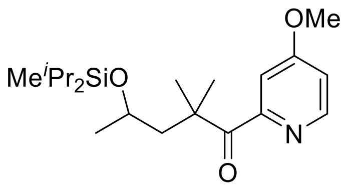

(why-08-163-2)

4-((diisopropyl(methyl)silyl)oxy)-1-(4-methoxypyridin-2-yl)-2,2-dimethylpentan-1-one (3d)

47.1 mg, 43% yield, Red oil, R_f = 0.5 (petroleum ether/ethyl acetate = 10:1).

^{1}H NMR (500 MHz, $CDCl_3$) δ 8.40 (d, *J* = 5.6 Hz, 1H), 7.49 (d, *J* = 2.6 Hz, 1H), 6.85 (dd, *J* = 5.6, 2.6 Hz, 1H), 3.98 – 3.90 (m, 1H), 3.86 (s, 3H), 2.73 (dd, *J* = 14.2, 7.6 Hz,

1H), 2.10 (dd, J = 14.0, 5.3 Hz, 1H), 1.42 (s, 3H), 1.38 (s, 3H), 1.02 (d, J = 6.1 Hz, 3H), 0.91 – 0.75 (m, 14H), -0.10 (s, 3H).

^{13}C NMR (126 MHz, $CDCl_3$) δ 205.3, 166.1, 156.7, 148.9, 112.4, 108.9, 66.6, 55.2, 50.3, 46.6, 26.9, 26.3, 24.8, 17.49, 17.47, 17.46, 17.4, 13.5, 13.4, -7.7.

IR (KBr) ν 2941, 2865, 1684, 1590, 1473, 1277, 1252, 999, 780.

HRMS (ESI) m/z: Calc. For $C_{20}H_{36}NO_3Si$ ($[M+H]^+$) 366.2459, Found 366.2457.

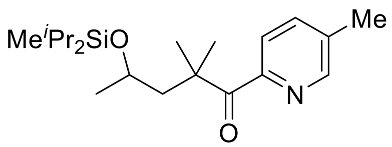

(why-08-175-5)

4-((diisopropyl(methyl)silyl)oxy)-2,2-dimethyl-1-(5-methylpyridin-2-yl)pentan-1-one (3e)

70.1 mg, 67% yield, Light yellow oil, R_f = 0.8 (petroleum ether/ethyl acetate = 10:1).

^{1}H NMR (500 MHz, $CDCl_3$) δ 8.41 (d, J = 2.4 Hz, 1H), 7.86 (d, J = 8.1 Hz, 1H), 7.54 (dd, J = 8.1, 2.4 Hz, 1H), 3.96 – 3.90 (m, 1H), 2.67 (dd, J = 14.0, 7.3 Hz, 1H), 2.36 (s, 3H), 2.16 (dd, J = 14.0, 5.5 Hz, 1H), 1.41 (s, 3H), 1.38 (s, 3H), 1.00 (d, J = 6.1 Hz, 3H), 0.90 – 0.77 (m, 14H), -0.11 (s, 3H).

^{13}C NMR (126 MHz, $CDCl_3$) δ 205.2, 152.4, 147.9, 136.7, 135.7, 123.5, 66.6, 50.3, 46.5, 26.8, 26.5, 24.8, 18.6, 17.50, 17.47, 17.45, 17.4, 13.39, 13.35, -7.7.

IR (KBr) ν 2942, 2866, 1682, 1463, 1251, 1132, 1061, 995, 780, 735.

HRMS (ESI) m/z: Calc. For $C_{20}H_{36}NO_2Si$ ($[M+H]^{+)}$ 350.2510, Found 350.2506.

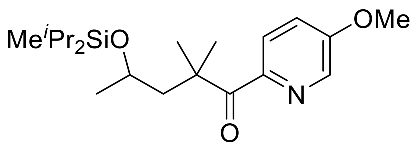

(why-08-181-2)

4-((diisopropyl(methyl)silyl)oxy)-1-(5-methoxypyridin-2-yl)-2,2-dimethylpentan-1-one (3f)

53.7 mg, 49% yield, Colorless oil, R_f = 0.6 (petroleum ether/ethyl acetate = 10:1).

^1H NMR (500 MHz, $CDCl_3$) δ 8.26 (d, *J* = 2.9 Hz, 1H), 7.99 (d, *J* = 8.9 Hz, 1H), 7.20 (dd, *J* = 8.7, 3.1 Hz, 1H), 3.97 – 3.91 (m, 1H), 3.90 (s, 3H), 2.67 (dd, *J* = 14.1, 7.2 Hz, 1H), 2.18 (dd, *J* = 14.1, 5.7 Hz, 1H), 1.40 (s, 3H), 1.38 (s, 3H), 0.99 (d, *J* = 6.1 Hz, 3H), 0.91 – 0.75 (m, 14H), -0.11 (s, 3H).

^{13}C NMR (126 MHz, $CDCl_3$) δ 204.2, 157.0, 147.6, 135.1, 125.4, 119.7, 66.6, 55.6, 50.3, 46.4, 26.9, 26.6, 24.7, 17.51, 17.47, 17.46, 17.4, 13.33, 13.29, -7.8.

IR (KBr) ν 2925, 2865, 1677, 1581, 1472, 1254, 994, 784.

HRMS (ESI) m/z: Calc. For $C_{20}H_{36}NO_3Si$ ($[M+H]^{+}$) 366.2459, Found 366.2457.

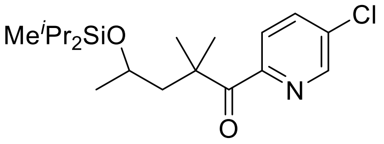

(why-08-173-8)

1-(5-chloropyridin-2-yl)-4-((diisopropyl(methyl)silyl)oxy)-2,2-dimethylpentan-1-one (3g)

54.2 mg, 49% yield, Light yellow oil, R_f = 0.5 (petroleum ether/ethyl acetate = 10:1).

^1H NMR (500 MHz, $CDCl_3$) δ 8.54 (d, *J* = 2.6 Hz, 1H), 7.93 (d, *J* = 8.4 Hz, 1H), 7.72 (dd, *J* = 8.5, 2.5 Hz, 1H), 3.99 – 3.93 (m, 1H), 2.81 (dd, *J* = 14.2, 8.2 Hz, 1H), 1.98 (dd, *J* = 14.2, 4.9 Hz, 1H), 1.40 (s, 3H), 1.35 (s, 3H), 1.02 (d, *J* = 6.1 Hz, 3H), 0.88 – 0.69 (m, 14H), -0.13 (s, 3H).

^{13}C NMR (126 MHz, $CDCl_3$) δ 203.9, 152.6, 146.3, 136.1, 134.3, 124.9, 66.5, 50.4, 46.5, 27.0, 25.9, 24.8, 17.5, 17.44, 17.41, 17.39, 13.4, 13.3, -7.7.

IR (KBr) ν 2958, 2866, 1685, 1461,1418, 1252, 1108, 1062, 994, 884, 780, 735.

HRMS (ESI) m/z: Calc. For $C_{19}H_{33}NO_2ClSi$ ($[M+H]^{+}$) 370.1964, Found 370.1960.

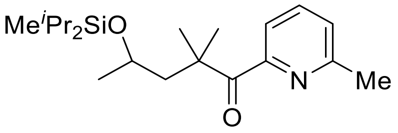

(why-08-176-2)

4-((diisopropyl(methyl)silyl)oxy)-2,2-dimethyl-1-(6-methylpyridin-2-yl)pentan-1-

one (3h)

27.2 mg, 26% yield, Red oil, R_f = 0.8 (petroleum ether/ethyl acetate = 10:1).

^{1}H NMR (500 MHz, $CDCl_3$) δ 7.72 (d, *J* = 7.8 Hz, 1H), 7.63 (t, *J* = 7.7 Hz, 1H), 7.18 (d, *J* = 7.8 Hz, 1H), 3.96 – 3.89 (m, 1H), 2.67 (dd, *J* = 14.1, 7.1 Hz, 1H), 2.56 (s, 3H), 2.17 (dd, *J* = 14.0, 5.5 Hz, 1H), 1.42 (s, 3H), 1.39 (s, 3H), 1.02 (d, *J* = 6.0 Hz, 3H), 0.91 – 0.79 (m, 14H), -0.11 (s, 3H).

^{13}C NMR (126 MHz, $CDCl_3$) δ 205.7, 156.3, 154.3, 136.5, 125.0, 120.7, 66.7, 50.5, 46.7, 26.9, 26.6, 24.9, 24.3, 17.51, 17.47, 17.45, 17.4, 13.40, 13.37, -7.8.

IR (KBr) ν 2941, 2866, 1684, 1587,1454, 1252, 1137, 1064, 996, 783.

HRMS (ESI) m/z: Calc. For $C_{20}H_{36}NO_2Si$ ($[M+H]^+$) 350.2510, Found 350.2506.

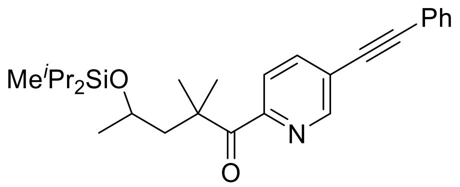

(why-09-34-6)

4-((diisopropyl(methyl)silyl)oxy)-2,2-dimethyl-1-(5-(phenylethynyl)pyridin-2-yl)pentan-1-one (3i)

117.5 mg, 90% yield, Light yellow oil, R_f = 0.8 (petroleum ether/ethyl acetate = 10:1).

^{1}H NMR (500 MHz, $CDCl_3$) δ 8.73 (d, *J* = 2.4 Hz, 1H), 7.96 (d, *J* = 8.2 Hz, 1H), 7.86 (dd, *J* = 8.2, 2.2 Hz, 1H), 7.57 (dd, *J* = 6.7, 3.1 Hz, 2H), 7.38 (dd, *J* = 4.4, 2.1 Hz, 3H), 4.01 – 3.94 (m, 1H), 2.79 (dd, *J* = 14.1, 7.9 Hz, 1H), 2.07 (dd, *J* = 14.1, 5.0 Hz, 1H), 1.43 (s, 3H), 1.39 (s, 3H), 1.04 (d, *J* = 6.1 Hz, 3H), 0.91 – 0.75 (m, 14H), -0.10 (s, 3H).

^{13}C NMR (126 MHz, $CDCl_3$) δ 204.5, 153.1, 149.9, 138.7, 131.8, 129.0, 128.5, 123.1, 122.40, 122.36, 94.5, 86.1, 66.6, 50.4, 46.6, 26.9, 26.2, 24.9, 17.6, 17.51, 17.49, 17.45, 13.5, 13.4, -7.6.

IR (KBr) ν 2941, 2865, 1682, 1462, 1252, 1138, 1060, 992, 957, 782, 755, 689.

HRMS (ESI) m/z: Calc. For $C_{27}H_{38}NO_2Si$ ($[M+H]^+$) 436.2666, Found 436.2661.

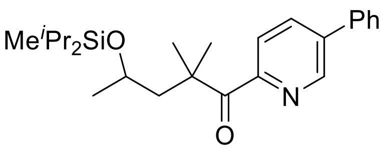

(why-09-34-5)

4-((diisopropyl(methyl)silyl)oxy)-2,2-dimethyl-1-(5-phenylpyridin-2-yl)pentan-1-one (3j)

108.5 mg, 88% yield, Light yellow oil, R_f = 0.8 (petroleum ether/ethyl acetate = 10:1).

^{1}H NMR (500 MHz, $CDCl_3$) δ 8.84 (d, *J* = 2.4 Hz, 1H), 8.05 (d, *J* = 8.2 Hz, 1H), 7.95 (dd, *J* = 8.2, 2.4 Hz, 1H), 7.62 (dd, *J* = 8.3, 1.3 Hz, 2H), 7.50 (t, *J* = 7.6 Hz, 2H), 7.47 – 7.42 (m, 1H), 4.02 – 3.96 (m, 1H), 2.80 (dd, *J* = 14.2, 7.6 Hz, 1H), 2.15 (dd, *J* = 14.0, 5.2 Hz, 1H), 1.47 (s, 3H), 1.43 (s, 3H), 1.06 (d, *J* = 6.1 Hz, 3H), 0.92 – 0.72 (m, 14H), -0.09 (s, 3H).

^{13}C NMR (126 MHz, $CDCl_3$) δ 205.0, 153.6, 146.0, 138.2, 137.4, 134.5, 129.2, 128.5, 127.3, 123.9, 66.7, 50.4, 46.6, 26.9, 26.4, 24.9, 17.52, 17.50, 17.48, 17.4, 13.5, 13.4, -7.6.

IR (KBr) ν 2941, 2864, 1681, 1468, 1061, 995, 782, 754, 695.

HRMS (ESI) m/z: Calc. For $C_{25}H_{38}NO_2Si$ ([M+H]$^+$) 412.2666, Found 412.2661.

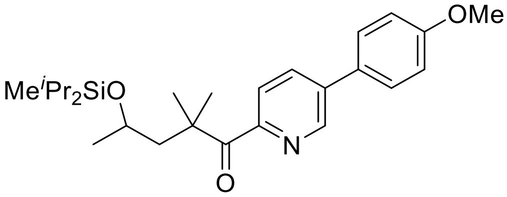

(why-09-33-6)

4-((diisopropyl(methyl)silyl)oxy)-1-(5-(4-methoxyphenyl)pyridin-2-yl)-2,2-dimethylpentan-1-one (3k)

116.4 mg, 88% yield, Light yellow oil, R_f = 0.6 (petroleum ether/ethyl acetate = 10:1).

^{1}H NMR (500 MHz, $CDCl_3$) δ 8.81 (d, *J* = 2.4 Hz, 1H), 8.01 (d, *J* = 8.2 Hz, 1H), 7.90 (dd, *J* = 8.2, 2.4 Hz, 1H), 7.59 – 7.54 (m, 2H), 7.05 – 7.00 (m, 2H), 4.00 – 3.95 (m, 1H), 3.87 (s, 3H), 2.76 (dd, *J* = 14.1, 7.4 Hz, 1H), 2.17 (dd, *J* = 14.1, 5.4 Hz, 1H), 1.46 (s, 3H), 1.42 (s, 3H), 1.05 (d, *J* = 6.1 Hz, 3H), 0.92 – 0.73 (m, 14H), -0.09 (s, 3H).

^{13}C NMR (126 MHz, $CDCl_3$) δ 205.0, 160.2, 153.0, 145.5, 137.9, 133.9, 129.7, 128.4, 123. 9, 114.7, 66.7, 55.4, 50.4, 46.6, 26.9, 26.4, 24.9, 17.52, 17.50, 17.48, 17.4, 13.5, 13.4, -7.6.

IR (KBr) ν 2940, 2865, 1680, 1519, 1468, 1252, 1178, 998, 830, 784.

HRMS (ESI) m/z: Calc. For $C_{26}H_{40}NO_3Si$ ($[M+H]^+$) 442.2772, Found 442.2768.

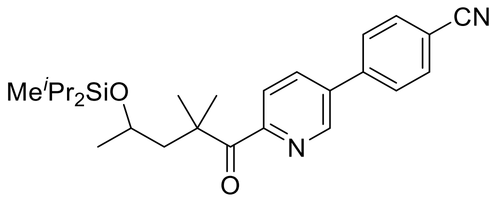

(why-09-33-7)

4-(6-(4-((diisopropyl(methyl)silyl)oxy)-2,2-dimethylpentanoyl)pyridin-3-yl)benzonitrile (3l)

43.2 mg, 33% yield, Colorless oil, R_f = 0.4 (petroleum ether/ethyl acetate = 10:1).

^1H NMR (500 MHz, $CDCl_3$) δ 8.84 (d, J = 2.6 Hz, 1H), 8.09 (d, J = 8.2 Hz, 1H), 7.96 (dd, J = 8.2, 2.4 Hz, 1H), 7.80 (d, J = 8.4 Hz, 2H), 7.72 (d, J = 8.2 Hz, 2H), 4.02 – 3.97 (m, 1H), 2.86 (dd, J = 14.2, 7.9 Hz, 1H), 2.07 (dd, J = 14.2, 4.9 Hz, 1H), 1.46 (s, 3H), 1.41 (s, 3H), 1.05 (d, J = 6.1 Hz, 3H), 0.89 – 0.75 (m, 14H), -0.10 (s, 3H).

^{13}C NMR (126 MHz, $CDCl_3$) δ 204.5, 154.6, 146.0, 141.9, 136.2, 134.8, 132.9, 127.9, 124.0, 118.4, 112.4, 66.6, 50.4, 46.6, 27.0, 26.1, 24.9, 17.52, 17.49, 17.46, 17.4, 13.5, 13.4, -7.7.

IR (KBr) ν 2923, 2863, 2229, 1680, 1470, 1369, 1251, 1161, 1060, 992, 837, 782.

HRMS (ESI) m/z: Calc. For $C_{26}H_{37}N_2O_2Si$ ($[M+H]^+$) 437.2619, Found 437.2618.

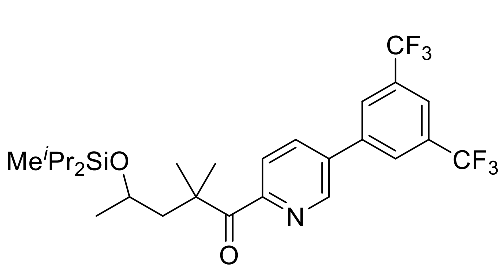

(why-09-34-3)

1-(5-(3,5-bis(trifluoromethyl)phenyl)pyridin-2-yl)-4-

((diisopropyl(methyl)silyl)oxy)-2,2-dimethylpentan-1-one (3m)

133.2 mg, 81% yield, Light yellow oil, R_f = 0.9 (petroleum ether/ethyl acetate = 10:1).

^{1}H NMR (500 MHz, $CDCl_3$) δ 8.89 (d, *J* = 2.4 Hz, 1H), 8.15 – 8.09 (m, 1H), 8.05 (s, 2H), 8.03 – 7.98 (m, 1H), 7.95 (s, 1H), 4.05 – 3.98 (m, 1H), 2.88 (dd, *J* = 14.2, 8.1, 1H), 2.09 – 2.02 (m, 1H), 1.47 (s, 3H), 1.41 (s, 3H), 1.06 (d, *J* = 6.0 Hz, 3H), 0.90 – 0.66 (m, 14H), -0.10 (s, 3H).

^{13}C NMR (126 MHz, $CDCl_3$) δ 204.4, 154.9, 146.0, 139.7, 135.3, 134.9, 132.8 (q, J_{C-F} = 34 Hz), 127.4, 124.1, 123.1 (q, J_{C-F} = 273 Hz), 122.2, 66.6, 50.4, 46.6, 27.0, 26.0, 24.9, 17.52, 17.48, 17.45, 17.4, 13.5, 13.4, -7.7.

IR (KBr) ν 2942, 2866, 1684, 1384, 1280, 1183, 1139.

HRMS (ESI) m/z: Calc. For $C_{27}H_{36}NO_2F_6Si$ ($[M+H]^+$) 548.2414, Found 548.2408.

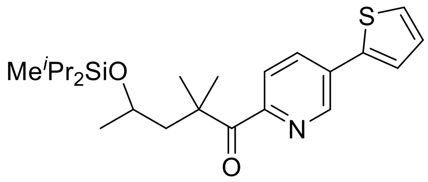

(why-09-34-4)

4-((diisopropyl(methyl)silyl)oxy)-2,2-dimethyl-1-(5-(thiophen-2-yl)pyridin-2-yl)pentan-1-one (3n)

83.8 mg, 67% yield, Light yellow oil, R_f = 0.8 (petroleum ether/ethyl acetate = 10:1).

^{1}H NMR (500 MHz, $CDCl_3$) δ 8.87 (d, *J* = 2.4 Hz, 1H), 8.01 (d, *J* = 8.2 Hz, 1H), 7.93 (dd, *J* = 8.2, 2.4 Hz, 1H), 7.61 (d, *J* = 3.2, 1H), 7.49 – 7.41 (m, 2H), 4.00 – 3.95 (m, 1H), 2.75 (dd, *J* = 14.0, 7.5 Hz, 1H), 2.17 (d, *J* = 5.3 Hz, 1H), 1.45 (s, 3H), 1.42 (s, 3H), 1.04 (d, *J* = 6 Hz, 3H) 0.91 – 0.77 (m, 14H), -0.09 (s, 3H).

^{13}C NMR (126 MHz, $CDCl_3$) δ 204.8, 153.2, 145.3, 138.4, 133.6, 133.1, 127.2, 125.9, 124.0, 122.4, 66.7, 50.4, 46.6, 26.9, 26.4, 24.9, 17.53, 17.51, 17.48, 17.4, 13.5, 13.4, -7.6.

IR (KBr) ν 2924, 2863, 1678, 1458, 1060, 992, 773.

HRMS (ESI) m/z: Calc. For $C_{23}H_{36}NO_2SSi$ ($[M+H]^+$) 418.2231, Found 418.2226.

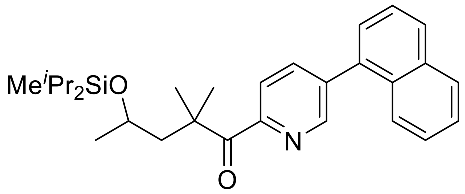

(why-09-33-5)

4-((diisopropyl(methyl)silyl)oxy)-2,2-dimethyl-1-(5-(naphthalen-1-yl)pyridin-2-yl)pentan-1-one (3o)

71.1 mg, 55% yield, Light yellow oil, R_f = 0.8 (petroleum ether/ethyl acetate = 10:1).

^{1}H NMR (500 MHz, $CDCl_3$) δ 8.75 (d, *J* = 2.4 Hz, 1H), 8.13 (d, *J* = 8.1 Hz, 1H), 7.98 – 7.87 (m, 3H), 7.81 (d, *J* = 8.4 Hz, 1H), 7.60 – 7.51 (m, 2H), 7.51 – 7.46 (m, 1H), 7.43 (d, *J* = 7.2 Hz, 1H), 4.08 – 4.01 (m, 1H), 2.94 (dd, *J* = 14.2, 7.8 Hz, 1H), 2.14 (d, *J* = 5.0 Hz, 1H), 1.53 (s, 3H), 1.46 (s, 3H), 1.11 (d, *J* = 6.1 Hz, 3H), 0.96 – 0.87 (m, 14H), -0.05 (s, 3H).

^{13}C NMR (126 MHz, $CDCl_3$) δ204.9, 153.7, 148.3, 138.3, 137.7, 136.0, 133.9, 131.4, 128.8, 128.5, 127.4, 126.7, 126.2, 125.4, 125.2, 123.4, 66.7, 50.5, 46.6, 27.2, 26.2, 24.9, 17.6, 17.53, 17.52, 17.47, 13.54, 13.47, -7.7.

IR (KBr) ν 2957, 2865, 1682, 1463, 1252, 1135, 1062, 993, 801, 777.

HRMS (ESI) m/z: Calc. For $C_{29}H_{40}NO_2Si$ ($[M+H]^+$) 462.2823, Found 462.2818.

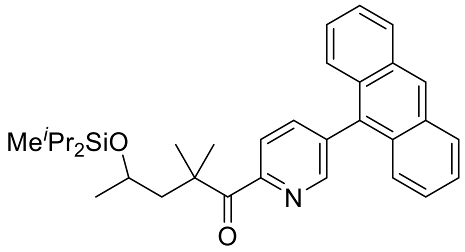

(why-09-33-8)

1-(5-(anthracen-9-yl)pyridin-2-yl)-4-((diisopropyl(methyl)silyl)oxy)-2,2-dimethylpentan-1-one (3p)

67.5 mg, 44% yield, Light yellow oil, R_f = 0.8 (petroleum ether/ethyl acetate = 10:1).

^{1}H NMR (500 MHz, $CDCl_3$) δ 8.68 (d, *J* = 2.4 Hz, 1H), 8.56 (s, 1H), 8.23 (d, *J* = 8.1 Hz, 1H), 8.08 (d, *J* = 8.4 Hz, 2H), 7.89 – 7.84 (m, 1H), 7.57 (dd, *J* = 8.9, 2.9 Hz, 2H), 7.50 (t, *J* = 7.5 Hz, 2H), 7.44 – 7.37 (m, 2H), 4.13 – 4.07 (m, 1H), 3.04 (dd, *J* = 14.2,

8.1 Hz, 1H), 2.10 (dd, J = 14.2, 4.9 Hz, 1H), 1.58 (s, 3H), 1.50 (s, 3H), 1.15 (d, J = 6.1, 3H), 0.98 – 0.85 (m, 14H), -0.02 (s, 3H).

^{13}C NMR (126 MHz, $CDCl_3$) δ 205.0, 154.0, 149.5, 139.3, 136.8, 132.3, 131.33, 131.30, 130.4, 128.6, 127.8, 126.1, 126.0, 125.92, 125.89, 125.34, 125.31, 123.5, 66.7, 50.5, 46.7, 27.4, 26.1, 25.0, 17.7, 17.61, 17.59, 17.55, 13.6, 13.5, -7.6.

IR (KBr) ν 2940, 2864, 1682, 1461, 1252, 1060, 992, 884, 783, 735.

HRMS (ESI) m/z: Calc. For $C_{33}H_{42}NO_2Si$ ([M+H]$^+$) 512.2979, Found 512.2977.

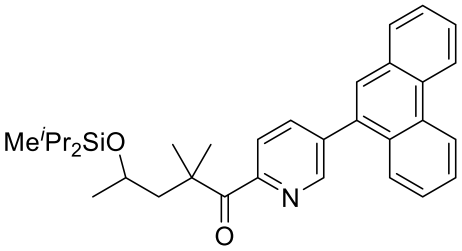

(why-09-34-2)

4-((diisopropyl(methyl)silyl)oxy)-2,2-dimethyl-1-(5-(phenanthren-9-yl)pyridin-2-yl)pentan-1-one (3q)

99.6 mg, 65% yield, Light yellow oil, R_f = 0.8 (petroleum ether/ethyl acetate = 10:1).

^{1}H NMR (500 MHz, $CDCl_3$) δ 8.83 – 8.78 (m, 2H), 8.74 (d, J = 8.4 Hz, 1H), 8.15 (d, J = 8.1 Hz, 1H), 7.98 – 7.90 (m, 2H), 7.82 (d, J = 8.2 Hz, 1H), 7.75 – 7.69 (m, 3H), 7.65 (t, J = 7.3 Hz, 1H), 7.58 (t, J = 7.6 Hz, 1H), 4.09 – 4.04 (m, 1H), 2.96 (dd, J = 14.2, 7.8 Hz, 1H), 2.14 (dd, J = 14.2, 5.0 Hz, 1H), 1.55 (s, 3H), 148 (s, 3H), 1.13 (d, J = 6.1 Hz, 3H), 0.97 – 0.81 (m, 14H), -0.04 (s, 3H).

^{13}C NMR (126 MHz, $CDCl_3$) δ 205.0, 153.8, 148.3, 138.4, 137.8, 134.7, 131.3, 130.8, 130.5, 130.4, 128.9, 128.4, 127.2, 127.1, 126.9, 126.2, 123.4, 123.2, 122.6, 66.7, 50.5, 46.7, 27.2, 26.2, 25.0, 17.63, 17.56, 17.54, 17.49, 13.6, 13.5, -7.6.

IR (KBr) ν 2941, 2864, 1681, 1452, 1251, 1137, 1061, 993, 782, 747, 726.

HRMS (ESI) m/z: Calc. For $C_{33}H_{42}NO_2Si$ ([M+H]$^+$) 512.2979, Found 512.2976.

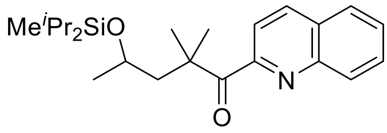

(why-08-175-3)

4-((diisopropyl(methyl)silyl)oxy)-2,2-dimethyl-1-(quinolin-2-yl)pentan-1-one (3r)

33.5 mg, 29% yield, Red oil, R_f = 0.8 (petroleum ether/ethyl acetate = 10:1).

^{1}H NMR (500 MHz, $CDCl_3$) δ 8.20 (d, J = 8.5 Hz, 1H), 8.15 (d, J = 8.5 Hz, 1H), 8.04 (d, J = 8.5 Hz, 1H), 7.83 (dd, J = 8.2, 1.4 Hz, 1H), 7.78 – 7.71 (m, 1H), 7.64 – 7.57 (m, 1H), 4.02 – 3.95 (m, 1H), 2.86 (dd, J = 14.1, 7.4 Hz, 1H), 2.28 (dd, J = 14.2, 5.5 Hz, 1H), 1.54 (s, 3H), 1.49 (s, 3H), 1.02 (d, J = 6.0 Hz, 3H), 0.87 – 0.74 (m, 14H), -0.12 (s, 3H).

^{13}C NMR (126 MHz, $CDCl_3$) δ 205.9, 154.1, 146.3, 136.2, 130.5, 129.5, 128.6, 128.0, 127.5, 120.3, 66.7, 50.7, 46.8, 27.2, 26.6, 24.9, 17.49, 17.46, 17.43, 17.40, 13.4, 13.3, -7.7.

IR (KBr) ν 2941, 2865, 1681, 1462, 1418, 1061, 993, 784.

HRMS (ESI) m/z: Calc. For $C_{23}H_{36}NO_2Si$ ($[M+H]^+$) 386.2510, Found 386.2506.

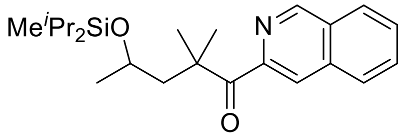

(why-08-181-4)

4-((diisopropyl(methyl)silyl)oxy)-1-(isoquinolin-3-yl)-2,2-dimethylpentan-1-one (3s)

80.9 mg, 70% yield, Red oil, R_f = 0.7 (petroleum ether/ethyl acetate = 10:1).

^{1}H NMR (500 MHz, $CDCl_3$) δ 9.21 (s, 1H), 8.40 (s, 1H), 8.00 (d, J = 7.9 Hz, 1H), 7.94 (d, J = 7.9 Hz, 1H), 7.75 – 7.65 (m, 2H), 4.02 – 3.94 (m, 1H), 2.78 (dd, J = 14.0, 7.3 Hz, 1H), 2.26 (dd, J = 14.1, 5.6 Hz, 1H), 1.47 (s, 3H), 1.45 (s, 3H), 1.02 (d, J = 6.1 Hz, 3H), 0.86 – 0.71 (m, 14H), -0.12 (s, 3H).

^{13}C NMR (126 MHz, $CDCl_3$) δ 205.5, 150.2, 149.1, 135.6, 130.4, 129.3, 128.8, 128.3, 127.2, 122.0, 66.6, 50.2, 46.7, 26.8, 26.5, 24.7, 17.41, 17.36, 13.32, 13.25, -7.8.

IR (KBr) ν 2941, 2865, 1680, 1461,1385, 1251, 1157, 1127, 1080, 998, 883, 784, 741.

HRMS (ESI) m/z: Calc. For $C_{23}H_{36}NO_2Si$ ($[M+H]^+$) 386.2510, Found 386.2506.

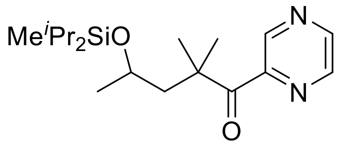

(why-08-173-2)

4-((diisopropyl(methyl)silyl)oxy)-2,2-dimethyl-1-(pyrazin-2-yl)pentan-1-one (3t)

47.4 mg, 47% yield, Red oil, R_f = 0.3 (petroleum ether/ethyl acetate = 10:1).

^{1}H NMR (500 MHz, $CDCl_3$) δ 9.16 (d, *J* = 1.5 Hz, 1H), 8.61 (d, *J* = 2.4 Hz, 1H), 8.57 – 8.55 (m, 1H), 4.05 – 3.96 (m, 1H), 2.98 – 2.89 (m, 1H), 1.84 (dd, *J* = 14.3, 4.1 Hz, 1H), 1.42 (s, 3H), 1.35 (s, 3H), 1.05 (d, *J* = 6.1 Hz, 3H), 0.87 – 0.72 (m, 14H), -0.14 (s, 3H).

^{13}C NMR (126 MHz, $CDCl_3$) δ 204.1, 149.2, 145.9, 145.5, 141.9, 66.4, 50.2, 46.6, 27.1, 25.2, 24.8, 17.5, 17.40, 17.37, 13.5, 13.3, -7.8.

IR (KBr) ν 2934, 2866, 1686, 1464, 1250, 1136, 1062, 992, 783, 736.

HRMS (ESI) m/z: Calc. For $C_{18}H_{33}N_2O_2Si$ ($[M+H]^+$) 337.2306, Found 337.2302.

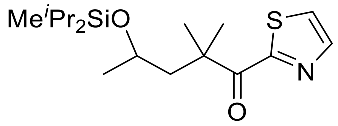

(why-08-175-6)

4-((diisopropyl(methyl)silyl)oxy)-2,2-dimethyl-1-(thiazol-2-yl)pentan-1-one (3u)

36.6 mg, 26% yield, Light yellow oil, R_f = 0.8 (petroleum ether/ethyl acetate = 10:1).

^{1}H NMR (500 MHz, $CDCl_3$) δ 7.94 (d, *J* = 3.1 Hz, 1H), 7.51 (d, *J* = 3.2 Hz, 1H), 4.03 – 3.95 (m, 1H), 2.91 (dd, *J* = 14.3, 8.6 Hz, 1H), 1.93 (dd, *J* = 14.2, 4.4 Hz, 1H), 1.43 (s, 3H), 1.37 (s, 3H), 1.06 (d, *J* = 6.0 Hz, 3H), 0.89 – 0.75 (m, 14H), -0.10 (s, 3H).

^{13}C NMR (126 MHz, $CDCl_3$) δ 197.5, 168.0, 143.9, 124.5, 66.3, 50.0, 46.1, 27.0, 24.9, 24.7, 17.5, 17.44, 17.41, 17.39, 13.5, 13.4, -7.8.

IR (KBr) ν 2942, 2866, 1673, 1463,1390, 1252, 1138, 1061, 987, 875, 784, 735.

HRMS (ESI) m/z: Calc. For $C_{17}H_{32}NO_2SSi$ ($[M+H]^+$) 342.1918, Found 342.1915.

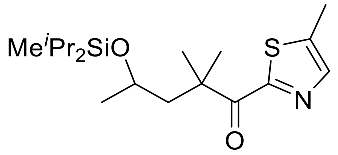

(why-08-173-3)

4-((diisopropyl(methyl)silyl)oxy)-2,2-dimethyl-1-(5-methylthiazol-2-yl)pentan-1-one (3v)

29.8 mg, 28% yield, Light yellow oil, R_f = 0.7 (petroleum ether/ethyl acetate = 10:1).

^{1}H NMR (500 MHz, $CDCl_3$) δ 7.60 – 7.56 (m, 1H), 4.00 – 3.93 (m, 1H), 2.76 (dd, J = 14.2, 8.2 Hz, 1H), 2.52 – 2.49 (m, 3H), 1.98 (dd, J = 14.2, 4.7 Hz, 1H), 1.40 (s, 3H), 1.36 (s, 3H), 1.05 (d, J = 6.1 Hz, 3H), 0.91 – 0.78 (m, 14H), -0.09 (s, 3H).

^{13}C NMR (126 MHz, $CDCl_3$) δ 197.6, 166.1, 142.2, 140.4, 66.4, 50.0, 46.0, 26.9, 25.3, 24.7, 17.47, 17.45, 17.43, 17.40, 13.5, 13.4, 12.2, -7.7.

IR (KBr) ν 2941, 2866, 1670, 1462,1418, 1252, 1138, 1080, 994, 883, 784, 733.

HRMS (ESI) m/z: Calc. For $C_{18}H_{33}N_2NaSSi$ ($[M+Na]^+$) 378.1894, Found 378.1890.

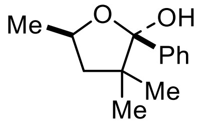

(why-09-154-2)

3,3,5-trimethyl-2-phenyltetrahydrofuran-2-ol (3w')

37.7 mg, 61% yield (according to typical procedure A with 5-trifluomethylpyridinone as the additive), Colorless oil, d.r. > 20:1, R_f = 0.2 (petroleum ether/ethyl acetate = 10:1).

^{1}H NMR (500 MHz, $CDCl_3$) δ 7.57 – 7.52 (m, 2H), 7.27 (t, J = 7.4 Hz, 2H), 7.24 – 7.20 (m, 1H), 5.12 (d, J = 2.0 Hz, 1H), 4.12 – 4.04 (m, 1H), 1.64 – 1.55 (m, 1H), 1.49 – 1.43 (m, 1H), 1.37 (d, J = 6.3 Hz, 3H), 1.09 (s, 3H), 1.08 (s, 3H).

^{13}C NMR (126 MHz, $CDCl_3$) δ 149.7, 136.2, 128.0, 127.7, 124.6, 108.1, 69.6, 44.8, 30.92, 30.87, 30.1, 21.4.

IR (KBr) ν 2957, 2931, 2866, 1648, 1493, 1448, 1360, 1317, 1281, 1091, 1053, 760, 692.

HRMS (ESI) m/z: Calc. For $C_{13}H_{17}O_2$ ($[M-H]^{-}$) 205.1234, Found 205.1220.

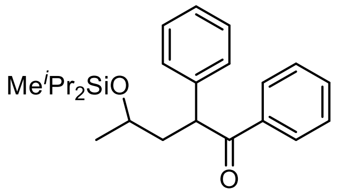

(why-09-142-1)

4-((diisopropyl(methyl)silyl)oxy)-1,2-diphenylpentan-1-one (3x)

83.4 mg, 72% yield (according to typical procedure A with 5-trifluomethylpyridinone as the additive), d.r. = 1:1, Colorless oil, R_f= 0.7 (petroleum ether/ethyl acetate = 10:1).

^{1}H NMR (500 MHz, $CDCl_3$) δ 8.00 – 7.92 (m, 2H), 7.49 – 7.42 (m, 1H), 7.40 – 7.33 (m, 2H), 7.28 – 7.15 (m, 5H), 4.89 – 4.81 (m, 1H), 3.85 – 3.75 (m, 1H), 2.48 – 2.33 (m, 1H), 1.97 – 1.87 (m, 1H), 1.19 – 1.09 (m, 3H), 0.94 – 0.74 (m, 14H), -0.15 – -0.18 (m, 3H).

^{13}C NMR (126 MHz, $CDCl_3$) δ 200.0, 199.6, 140.4, 139.6, 137.0, 136.9, 132.73, 132.71, 129.0, 128.9, 128.8, 128.7, 128.6, 128.5, 128.41, 128.38, 128.1, 127.6, 126.93, 126.86, 125.9, 66.9, 66.5, 49.9, 49.5, 44.4, 43.7, 24.3, 24.2, 17.7, 17.61, 17.55, 17.52, 17.49, 17.46, 17.41, 17.38, 13.7, 13.62, 13.55, 13.4, 13.3, -7.2, -7.6.

IR (KBr) ν 2942, 2894, 2865, 1684, 1452, 1252, 1136, 1095, 997, 883, 782, 699.

HRMS (ESI) m/z: Calc. For $C_{24}H_{33}O_2Si$ ($[M-H]^{-}$) 381.2255, Found 381.2240.

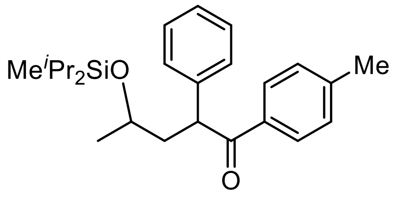

(why-09-153-4)

4-((diisopropyl(methyl)silyl)oxy)-2-phenyl-1-(p-tolyl)pentan-1-one (3y)

57.3 mg, 48% yield (according to typical procedure A with 4-methylpyridinone as the additive), d.r. = 1:1, Light yellow oil, R_f = 0.6 (petroleum ether/ethyl acetate = 10:1).

^{1}H NMR (500 MHz, CDCl$_3$) δ 7.90 – 7.82 (m, 2H), 7.33 – 7.20 (m, 3H), 7.17 – 7.12 (m, 2H), 7.10 – 7.02 (m, 2H), 4.89 – 4.73 (m, 1H), 3.87 – 3.71 (m, 1H), 2.48 – 2.35 (m, 1H), 2.33 (s, 3H), 1.77 (d, J = 1.1 Hz, 1H), 1.14 (m, 4H), 1.03 – 0.80 (m, 14H), -0.15 – -0.18 (m, 3H).

^{13}C NMR (126 MHz, CDCl$_3$) δ 199.6, 199.1, 143.52, 143.46, 140.7, 139.8, 134.4, 134.3, 129.4, 129.2, 129.1, 129.0, 128.91, 128.87, 128.8, 128.7, 128.6, 128.3, 128.1, 127.54, 127.50, 127.4, 126.84, 126.76, 125.9, 66.9, 66.5, 49.8, 49.3, 44.4, 43.6, 30.9, 24.3, 24.2, 22.8, 21.5, 17.7, 17.61, 17.57, 17.54, 17.49, 17.45, 17.41, 17.36, 13.7, 13.6, 13.5, 13.4, 13.2, -7.2, -7.6.

IR (KBr) ν 2941, 2893, 2865, 1681, 1458, 1252, 1135, 1094, 1065, 997, 882, 781, 700.

HRMS (ESI) m/z: Calc. For $C_{25}H_{35}O_2Si$ ([M-H]$^-$) 395.2412, Found 395.2398.

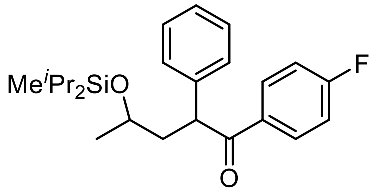

(why-09-144-2)

4-((diisopropyl(methyl)silyl)oxy)-1-(4-fluorophenyl)-2-phenylpentan-1-one (3z)

50.2 mg, 42% yield (according to typical procedure A with 5-trifluomethylpyridinone as the additive), d.r. = 1:1, Colorless oil, R_f = 0.8 (petroleum ether/ethyl acetate = 10:1).

^{1}H NMR (500 MHz, CDCl$_3$) δ 8.03 – 7.93 (m, 2H), 7.30 – 7.20 (m, 5H), 7.06 – 6.99 (m, 2H), 4.89 – 4.69 (m, 1H), 3.87 – 3.75 (m, 1H), 2.47 – 2.31 (m, 1H), 1.96 – 1.72 (m, 1H), 1.19 – 1.09 (m, 3H), 0.93 – 0.73 (m, 14H), -0.15 – -0.18 (m, 3H).

^{13}C NMR (126 MHz, CDCl$_3$) δ 198.4, 198.0, 165.6 (d, J_{CF} = 256 Hz), 165.5 (d, J_{CF} = 255 Hz), 140.3, 139.4, 131.4 (d, J_{CF} = 9 Hz), 131.3 (d, J_{CF} = 9 Hz), 129.1, 128.9, 128.5, 128.0, 127.0, 115.6 (d, J_{CF} = 22 Hz), 115.5 (d, J_{CF} = 22 Hz), 66.8, 66.5, 49.9, 49.6, 44.4, 43.6, 24.3, 24.2, 17.7, 17.60, 17.55, 17.53, 17.47, 17.45, 17.4, 13.7, 13.6, 13.5, 13.4, -7.2, -7.6.

IR (KBr) ν 2942, 2865, 1684, 1598, 1459, 1239, 1155, 1136, 1096, 1062, 996, 882, 781, 700.

HRMS (ESI) m/z: Calc. For $C_{24}H_{33}O_2FSi$ ([M-H]$^{-}$) 399.2161, Found 399.2144.

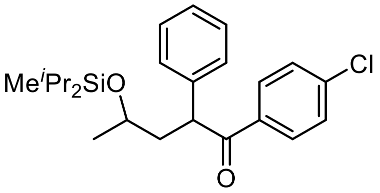

(why-09-142-3)

4-((diisopropyl(methyl)silyl)oxy)-1-(4-chlorophenyl)-2-phenylpentan-1-one (3aa)

92.1 mg, 74% yield (according to typical procedure A with 5-trifluomethylpyridinone as the additive), d.r. = 1:1, Colorless oil, R_f = 0.6 (petroleum ether/ethyl acetate = 10:1).

^{1}H NMR (500 MHz, $CDCl_3$) δ 7.98 – 7.84 (m, 2H), 7.38 – 7.28 (m, 2H), 7.28 – 7.12 (m, 5H), 4.84 – 4.70 (m, 1H), 3.89 – 3.72 (m, 1H), 2.46 – 2.27 (m, 1H), 1.99 – 1.84 (m, 1H), 1.14 (m, 3H), 0.94 – 0.85 (m, 14H), -0.15 – -0.18 (m, 3H).

^{13}C NMR (126 MHz, $CDCl_3$) δ 198.8, 198.3, 140.2, 139.3, 139.23, 139.17, 135.3, 135.1, 130.2, 130.1, 129.1, 128.9, 128.8, 128.7, 128.5, 128.0, 127.1, 127.0, 66.8, 66.5, 50.0, 49.6, 44.3, 43.6, 24.3, 24.2, 17.7, 17.61, 17.56, 17.54, 17.48, 17.46, 17.4, 13.7, 13.6, 13.5, 13.4, -7.2, -7.5.

IR (KBr) ν 2942, 2893, 2865, 1685, 1589, 1461, 1377, 1251, 1137, 1094, 1065, 997, 883, 781, 755, 734, 701.

HRMS (ESI) m/z: Calc. For $C_{24}H_{32}O_2ClSi$ ([M-H]$^{-}$) 415.1866, Found 415.1849.

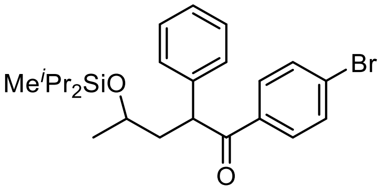

(why-09-142-4)

4-((diisopropyl(methyl)silyl)oxy)-1-(4-bromophenyl)-2-phenylpentan-1-one (3ab)

97.0 mg, 70% yield (according to typical procedure A with 5-trifluomethylpyridinone as the additive), d.r. = 1:1, Colorless oil, R_f = 0.6 (petroleum ether/ethyl acetate = 10:1).

^{1}H NMR (500 MHz, $CDCl_3$) δ 7.86 – 7.77 (m, 2H), 7.56 – 7.44 (m, 2H), 7.29 – 7.19 (m, 5H), 4.89 – 4.66 (m, 1H), 3.91 – 3.73 (m, 1H), 2.47 – 2.30 (m, 1H), 1.94 – 1.71 (m,

1H), 1.19 – 1.08 (m, 3H), 0.95 – 0.75 (m, 14H), -0.15 – -0.18 (m, 3H).

^{13}C NMR (126 MHz, $CDCl_3$) δ 199.0, 198.5, 140.1, 139.2, 135.7, 135.5, 131.8, 131.7, 130.29, 130.26, 129.1, 128.9, 128.5, 128.0, 127.0, 66.8, 66.4, 50.0, 49.6, 44.3, 43.5, 24.3, 17.7, 17.61, 17.56, 17.54, 17.49, 17.4, 13.7, 13.6, 13.5, 13.4, -7.2, -7.5.

HRMS (ESI) m/z: Calc. For $C_{24}H_{32}O_2BrSi$ ($[M-H]^-$) 459.1360, Found 459.1344.

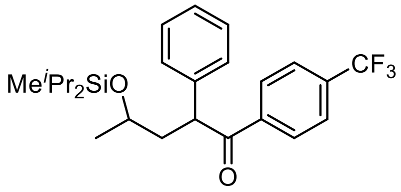

(why-09-144-5)

4-((diisopropyl(methyl)silyl)oxy)-2-phenyl-1-(4-(trifluoromethyl)phenyl)pentan-1-one (3ac)

59.2 mg, 44% yield (according to typical procedure A with 5-trifluomethylpyridinone as the additive), d.r. = 1:1, Colorless oil, R_f = 0.8 (petroleum ether/ethyl acetate = 10:1).

^{1}H NMR (500 MHz, $CDCl_3$) δ 8.08 – 8.00 (m, 2H), 7.66 – 7.58 (m, 2H), 7.30 – 7.18 (m, 5H), 4.92 – 4.74 (m, 1H), 3.88 – 3.76 (s, 1H), 2.50 – 2.32 (m, 1H), 1.98 – 1.72 (m, 1H), 1.20 – 1.08 (m, 3H), 1.02 – 0.84 (m, 14H), -0.15 – -0.18 (m, 3H).

^{13}C NMR (126 MHz, $CDCl_3$) δ 199.0, 198.6, 139.8, 138.8, 134.0 (d, J_{CF} = 32 Hz), 129.2, 129.1, 129.01, 128.99, 128.5, 128.1, 127.3, 127.2, 125.5 (d, J_{CF} = 4 Hz), 123.6 (q, J_{CF} = 273 Hz), 66.8, 66.4, 50.3, 50.0, 44.2, 43.4, 24.27, 24.26, 17.7, 17.6, 17.52, 17.51, 17.45, 17.4, 13.7, 13.6, 13.5, 13.4, -7.2, -7.6.

IR (KBr) ν 2944, 2894, 2866, 1690, 1461, 1409, 1324, 1252, 1171, 1135, 1068, 997, 883, 782, 700.

HRMS (ESI) m/z: Calc. For $C_{25}H_{32}O_2F_3Si$ ($[M-H]^-$) 449.2129, Found 449.2112.

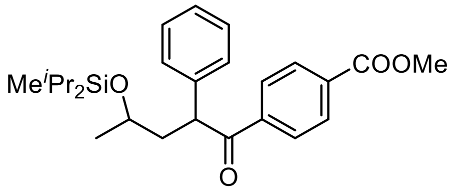

(why-09-144-4)

methyl 4-(4-((diisopropyl(methyl)silyl)oxy)-2-phenylpentanoyl)benzoate (3ad)

67.4 mg, 51% yield (according to typical procedure A with 5-trifluomethylpyridinone as the additive), d.r. = 1:1, Light yellow oil, R_f = 0.5 (petroleum ether/ethyl acetate = 10:1).

^{1}H NMR (500 MHz, $CDCl_3$) δ 8.08 – 7.91 (m, 4H), 7.31 – 7.15 (m, 5H), 4.88 – 4.78 (m, 1H), 3.89 (s, 3H), 3.83 – 3.75 (m, 1H), 2.54 – 2.30 (m, 1H), 1.97 – 1.73 (m, 1H), 1.20 – 1.07 (m, 3H), 1.05 – 0.78 (m, 14H), -0.15 – -0.18 (m, 3H).

^{13}C NMR (126 MHz, $CDCl_3$) δ 199.6, 199.1, 166.2, 140.2, 140.1, 139.9, 139.0, 133.6, 133.5, 129.7, 129.6, 129.1, 128.9, 128.63, 128.58, 128.1, 127.14, 127.07, 66.8, 66.4, 52.3, 50.3, 50.0, 44.2, 43.5, 24.29, 24.26, 17.7, 17.6, 17.54, 17.53, 17.47, 17.4, 13.7, 13.6, 13.5, 13.4, -7.2, -7.6.

IR (KBr) ν 2942, 2893, 2865, 1715, 1685, 1585, 1458, 1363, 1252, 1222, 1137, 1094, 1070, 996, 883, 780, 735, 701.

HRMS (ESI) m/z: Calc. For $C_{26}H_{35}O_4Si$ ($[M-H]^-$) 439.2310, Found 439.2293.

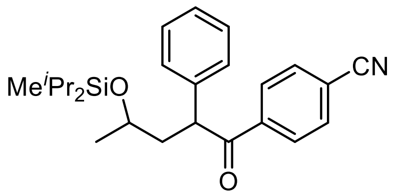

(why-09-144-6)

4-(4-((diisopropyl(methyl)silyl)oxy)-2-phenylpentanoyl)benzonitrile (3ae)

73.7mg, 60% yield (according to typical procedure A with 5-trifluomethylpyridinone as the additive), d.r. = 1:1, Light yellow oil, R_f = 0.4 (petroleum ether/ethyl acetate = 10:1).

^{1}H NMR (500 MHz, $CDCl_3$) δ 8.08 – 8.00 (m, 2H), 7.71 – 7.63 (m, 2H), 7.32 – 7.16 (m, 5H), 4.90 – 4.62 (m, 1H), 3.90 – 3.77 (m, 1H), 2.57 – 2.33 (m, 1H), 1.98 – 1.73 (m, 1H), 1.21 – 1.10 (m, 3H), 1.05 – 0.79 (m, 14H), -0.15 – -0.18 (m, 3H).

^{13}C NMR (126 MHz, $CDCl_3$) δ 198.6, 198.1, 139.9, 139.8, 139.4, 138.5, 132.33, 132.25, 129.2, 129.1, 129.04, 129.03, 128.5, 128.0, 127.34, 127.27, 117.9, 116.02, 115.98, 66.7, 66.3, 50.2, 50.1, 44.1, 43.4, 24.3, 24.2, 17.7, 17.6, 17.50, 17.49, 17.4, 17.3, 13.7, 13.6,

13.44, 13.35, -7.2, -7.6.

IR (KBr) ν2943, 2893, 2865, 2231, 1690, 1458, 1365, 1251, 1138, 1095, 1067, 997, 883, 782, 739, 701.

HRMS (ESI) m/z: Calc. For $C_{25}H_{32}O_2NSi$ ($[M-H]^{-}$) 406.2208, Found 406.2192.

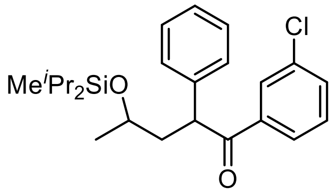

(why-09-151-1)

1-(3-chlorophenyl)-4-((diisopropyl(methyl)silyl)oxy)-2-phenylpentan-1-one (3af)

60.4 mg, 48% yield (according to typical procedure A with 5-trifluomethylpyridinone as the additive), d.r. = 1:1, Light yellow oil, R_f = 0.7 (petroleum ether/ethyl acetate = 10:1).

^{1}H NMR (500 MHz, $CDCl_3$) δ 7.96 – 7.92 (m, 1H), 7.86 – 7.82 (m, 1H), 7.45 – 7.38 (m, 1H), 7.35 – 7.17 (m, 6H), 4.83 – 4.73 (m, 1H), 3.85 – 3.73 (m, 1H), 2.52 – 2.28 (m, 1H), 2.00 – 1.75 (m, 1H), 1.20 – 1.09 (m, 3H), 1.02 – 0.84 (m, 14H), -0.17 (s, 3H).

^{13}C NMR (126 MHz, $CDCl_3$) δ 198.7, 198.3, 139.8, 139.0, 138.5, 138.4, 134.9, 134.8, 132.7, 132.6, 129.8, 129.7, 129.1, 128.9, 128.8, 128.5, 128.1, 127.14, 127.07, 126.8, 126.7, 66.8, 66.4, 50.0, 49.7, 44.3, 43.5, 26.9, 24.26, 24.24, 17.7, 17.6, 17.52, 17.51, 17.46, 17.4, 13.7, 13.6, 13.5, 13.4, -7.2, -7.6.

IR (KBr) ν 2943, 2893, 2865, 1687, 1572, 1462, 1421, 1377, 1251, 1203, 1138, 1095, 1070, 998, 883, 780, 727, 701.

HRMS (ESI) m/z: Calc. For $C_{24}H_{32}O_2ClSi$ ($[M-H]^{-}$) 415.1866, Found 415.1849.

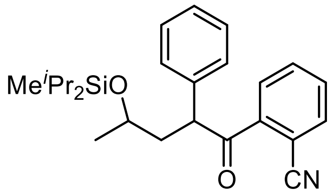

(why-09-151-4)

2-(4-((diisopropyl(methyl)silyl)oxy)-2-phenylpentanoyl)benzonitrile (3ag)

106.9 mg, 88% yield (according to typical procedure A with 5-trifluomethylpyridinone as the additive), d.r. = 1:1, Light yellow oil, R_f= 0.2 (petroleum ether/ethyl acetate = 10:1).

^{1}H NMR (500 MHz, $CDCl_3$) δ 7.90 – 7.85 (m, 1H), 7.74 – 7.70 (m, 1H), 7.60 – 7.48 (m, 2H), 7.29 – 7.18 (m, 5H), 4.80 – 4.73 (m, 1H), 3.92 – 3.74 (m, 1H), 2.55 – 2.32 (m, 1H), 1.99 – 1.74 (m, 1H), 1.20 – 1.10 (m, 3H), 1.03 – 0.80 (m, 14H), -0.14 – -0.18 (m, 3H).

^{13}C NMR (126 MHz, $CDCl_3$) δ 198.6, 197.8, 140.0, 139.2, 138.2, 135.3, 135.2, 132.14, 132.07, 132.0, 131.8, 129.5, 129.2, 129.06, 129.05, 128.6, 128.1, 127.4, 127.3, 118.0, 117.8, 111.9, 111.8, 66.8, 66.2, 51.1, 51.0, 44.0, 43.1, 24.2, 17.7, 17.61, 17.55, 17.53, 17.47, 17.4, 13.7, 13.6, 13.5, 13.4, -7.2, -7.5.

IR (KBr) ν 2942, 2865, 1693, 1457, 1251, 1137, 1095, 1069, 996, 961, 883, 765, 701.

HRMS (ESI) m/z: Calc. For $C_{25}H_{32}O_2NSi$ ($[M-H]^-$) 406.2208, Found 406.2192.

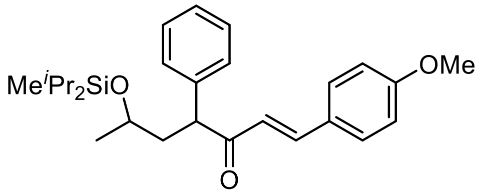

(why-09-153-1)

(E)-6-((diisopropyl(methyl)silyl)oxy)-1-(4-methoxyphenyl)-4-phenylhept-1-en-3-one (3ah)

50.1mg, 38% yield (according to typical procedure A with 4-methylpyridinone as the additive), d.r. = 1:1, Light yellow oil, R_f = 0.3 (petroleum ether/ethyl acetate = 10:1).

^{1}H NMR (500 MHz, $CDCl_3$) δ 7.46 – 7.41 (m, 1H), 7.30 (dd, J = 8.8, 6.0 Hz, 2H), 7.20 – 7.05 (m, 6H), 6.73 (dd, J = 8.8, 2.1 Hz, 2H), 6.51 – 6.44 (m, 1H), 4.08 – 4.02 (m, 1H), 3.74 – 3.62 (m, 4H), 2.29 – 2.20 (m, 1H), 1.67 – 1.67 (m, 1H), 1.08 – 1.00 (m, 3H), 0.91 – 0.80 (m, 14H), -0.15 – -0.18 (m, 3H).

^{13}C NMR (126 MHz, $CDCl_3$) δ 199.0, 198.7, 161.5, 142.43, 142.36, 139.9, 139.4, 130.01, 129.97, 128.9, 128.8, 128.6, 128.4, 128.3, 127.3, 127.01, 126.97, 123.5, 123.1, 114.3, 66.9, 66.5, 55.3, 53.7, 53.6, 43.0, 42.3, 24.2, 24.1, 17.7, 17.61, 17.59, 17.5, 17.4,

13.6, 13.5, 13.4, -7.3, -7.6.

IR (KBr) ν 2941, 2865, 1685, 1599, 1573, 1512, 1460, 1254, 1172, 1064, 1034, 995, 883, 782, 702.

HRMS (ESI) m/z: Calc. For $C_{27}H_{37}O_3Si$ ($[M-H]^{-}$) 437.2517, Found 437.2502.

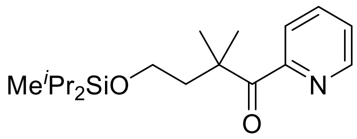

(why-09-63-2)

4-((diisopropyl(methyl)silyl)oxy)-2,2-dimethyl-1-(pyridin-2-yl)butan-1-one (3ai)

70.3 mg, 73% yield, Light yellow oil, R_f = 0.7 (petroleum ether/ethyl acetate = 10:1).

^{1}H NMR (500 MHz, $CDCl_3$) δ 8.61 – 8.57 (m, 1H), 7.91 (dd, J = 8.0, 3.4 Hz, 1H), 7.79 – 7.73 (m, 1H), 7.34 (dt, J = 8.2, 4.1 Hz, 1H), 3.64 – 3.56 (m, 2H), 2.44 – 2.37 (m, 2H), 1.42 (s, 6H), 0.89 – 0.71 (m, 14H), -0.15 (s, 3H).

^{13}C NMR (126 MHz, $CDCl_3$) δ 205.5, 155.0, 147.6, 136.5, 125.6, 123.6, 60.2, 46.2, 43.0, 26.0, 17.30, 17.25, 12.8, -8.9.

IR (KBr) ν 2924, 2865, 1685, 1463 1251, 1098, 996, 975, 882, 809, 780, 743.

HRMS (ESI) m/z: Calc. For $C_{18}H_{32}NO_2Si$ ($[M+H]^{+}$) 322.2197, Found 322.2194.

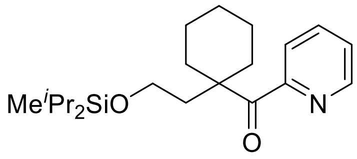

(why-09-63-4)

(1-(2-((diisopropyl(methyl)silyl)oxy)ethyl)cyclohexyl)(pyridin-2-yl)methanone (3aj)

55.9 mg, 58% yield, Light yellow oil, R_f = 0.7 (petroleum ether/ethyl acetate = 10:1).

^{1}H NMR (500 MHz, $CDCl_3$) δ 8.60 – 8.55 (m, 1H), 7.85 (d, J = 7.8 Hz, 1H), 7.77 (dd, J = 7.7, 1.9 Hz, 1H), 7.38 – 7.32 (m, 1H), 3.56 (t, J = 7.4 Hz, 2H), 2.46 (t, J = 7.5 Hz, 2H), 2.42 – 2.36 (m, 2H), 1.63 – 1.54 (m, 4H), 1.39 – 1.27 (m, 4H), 0.89 – 0.73 (m, 14H), -0.15 (s, 3H).

^{13}C NMR (126 MHz, $CDCl_3$) δ 205.9, 155.7, 147.5, 136.5, 125.6, 123.4, 59.7, 50.5, 41.0, 34.3, 26.3, 22.9, 17.32, 17.26, 12.8, -8.9.

IR (KBr) ν 2934, 2864, 1682, 1458, 1251, 1098, 996, 780, 743.

HRMS (ESI) m/z: Calc. For $C_{21}H_{36}NO_2Si$ ($[M+H]^+$) 362.2510, Found 362.2505.

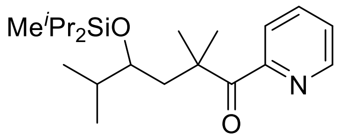

(why-09-63-3)

4-((diisopropyl(methyl)silyl)oxy)-2,2,4-trimethyl-1-(pyridin-2-yl)pentan-1-one (3ak)

53.4 mg, 49% yield, Light yellow oil, R_f = 0.7 (petroleum ether/ethyl acetate = 10:1).

^1H NMR (500 MHz, $CDCl_3$) δ 8.59 (dd, J = 4.7, 1.7 Hz, 1H), 7.97 – 7.91 (m, 1H), 7.75 (td, J = 7.8, 1.8 Hz, 1H), 7.34 – 7.30 (m, 1H), 3.75 – 3.69 (m, 1H), 2.52 (dd, J = 14.3, 8.2 Hz, 1H), 2.09 (dd, J = 14.3, 4.1 Hz, 1H), 1.43 (s, 3H), 1.40 (s, 3H), 1.01 – 0.86 (m, 10H), 0.94 – 0.86 (m, 8H), 0.66 (d, J = 7.0 Hz, 3H), -0.08 (s, 3H).

^{13}C NMR (126 MHz, $CDCl_3$) δ 205.5, 155.0, 147.5, 136.4, 125.5, 123.7, 74.5, 46.4, 41.9, 33.6, 27.0, 26.4, 17.74, 17.72, 17.68, 17.6, 16.3, 13.8, 13.7, -6.9.

IR (KBr) ν 2958, 2866, 1685, 1464, 1252, 1057, 996, 782, 743.

HRMS (ESI) m/z: Calc. For $C_{21}H_{38}NO_2Si$ ($[M+H]^+$) 364.2666, Found 364.2661.

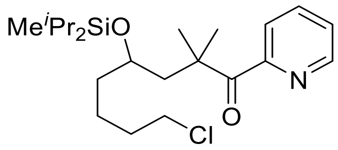

(why-09-67-8)

8-chloro-4-((diisopropyl(methyl)silyl)oxy)-2,2-dimethyl-1-(pyridin-2-yl)octan-1-one (3al)

82.6 mg, 67% yield, Light yellow oil, R_f = 0.7 (petroleum ether/ethyl acetate = 10:1).

^1H NMR (500 MHz, $CDCl_3$) δ 8.60 (d, J = 4.7Hz, 1H), 7.94 (d, J = 7.9, 1H), 7.76 (td, J = 7.8, 1.8 Hz, 1H), 7.36 – 7.32 (m, 1H), 3.86 – 3.81 (m, 1H), 3.43 (t, J = 6.8 Hz, 2H),

2.57 (dd, J = 14.3, 7.4 Hz, 1H), 2.28 (dd, J = 14.2, 5.3 Hz, 1H), 1.70 – 1.59 (m, 2H), 1.42 (s, 3H), 1.40 (s, 3H), 1.38 – 1.28 (m, 4H), 0.92 – 0.74 (m, 14H), -0.08 (s, 3H).

^{13}C NMR (126 MHz, $CDCl_3$) δ 205.3, 154.9, 147.6, 136.5, 125.6, 123.7, 70.1, 47.1, 46.6, 44.9, 37.3, 32.8, 26.8, 26.6, 22.1, 17.64, 17.60, 17.57, 17.56, 13.7, 13.6, -7.3.

IR (KBr) ν 2956, 2865, 1685, 1459, 1258, 1086, 1028, 998, 789, 743.

HRMS (ESI) m/z: Calc. For $C_{22}H_{39}ClNO_2Si$ ([M+H]$^+$) 412.2433, Found 412.2425.

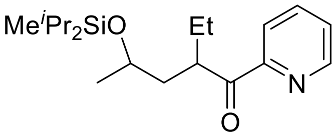

(why-09-73-1)

4-((diisopropyl(methyl)silyl)oxy)-2-ethyl-1-(pyridin-2-yl)pentan-1-one (3am)

28.1 mg, 28% yield, d.r. = 1:1, Colorless oil, R_f = 0.6 (petroleum ether/ethyl acetate = 10:1).

^{1}H NMR (500 MHz, $CDCl_3$) δ 8.67 (d, J = 3.7 Hz, 1H), 8.06 – 8.00 (m, 1H), 7.82 (td, J = 7.6, 1.8 Hz, 1H), 7.47 – 7.41 (m, 1H), 4.09 – 4.03 (m, 1H), 3.83 – 3.76 (m, 1H), 1.21 – 1.16 (m, 3H), 1.13 – 1.09 (m, 3H), 1.06 – 0.99 (m, 4H), 0.98 – 0.82 (m, 14H), -0.03 (s, 3H).

^{13}C NMR (126 MHz, $CDCl_3$) δ 205.9, 205.6, 153.3, 148.9, 136.8, 126.7, 122.3, 68.7, 68.5, 47.1, 39.1, 37.3, 37.2, 29.3, 29.0, 23.6, 23.4, 17.48, 17.46, 17.4, 17.2, 16.8, 16.5, 13.4, 13.3, -7.9.

IR (KBr) ν 2925, 2862, 1698, 1458, 1093, 1026, 996, 796.

HRMS (ESI) m/z: Calc. For $C_{19}H_{34}NO_2Si$ ([M+H]$^+$) 336.2353, Found 336.2352.

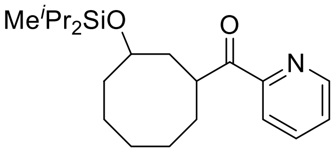

(why-09-73-2)

(3-((diisopropyl(methyl)silyl)oxy)cyclooctyl)(pyridin-2-yl)methanone (3an)

22.7 mg, 21% yield, d.r. = 1:1, Colorless oil, R_f = 0.5 (petroleum ether/ethyl acetate = 10:1).

^{1}H NMR (500 MHz, $CDCl_3$) δ 8.67 (dt, J = 4.7, 2.3 Hz, 1H), 8.03 (dd, J = 7.8, 3.4 Hz, 1H), 7.85 – 7.78 (m, 1H), 7.43 (dd, J = 7.9, 4.5 Hz, 1H), 4.15 – 4.02 (m, 1H), 3.97 – 3.90 (m, 1H), 1.95 – 1.60 (m, 10H), 1.03 – 0.84 (m, 16H), 0.04 – 0.01 (m, 3H).

^{13}C NMR (126 MHz, $CDCl_3$) δ 205.1, 205.0, 153.2, 148.9, 148.8, 136.84, 136.80, 126.7, 122.6, 122.5, 72.3, 71.9, 43.7, 43.4, 35.4, 35.2, 33.61, 33.55, 27.3, 27.1, 26.3, 26.1, 24.3, 23.9, 22.6, 22.5, 17.50, 17.46, 13.39, 13.36, 13.3, -8.1.

IR (KBr) ν 2935, 2863, 1697, 1460, 1253, 1059, 995, 783.

HRMS (ESI) m/z: Calc. For $C_{21}H_{36}NO_2Si$ ($[M+H]^+$) 362.2510, Found 362.2506.

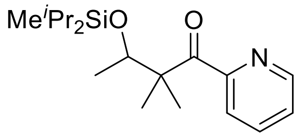

(why-09-67-1)

3-((diisopropyl(methyl)silyl)oxy)-2,2-dimethyl-1-(pyridin-2-yl)butan-1-one (3ao)

36.6 mg, 38% yield, Light yellow oil, R_f = 0.7 (petroleum ether/ethyl acetate = 10:1).

^{1}H NMR (500 MHz, $CDCl_3$) δ 8.61 – 8.56 (m, 1H), 7.91 (dd, J = 7.9, 1.2 Hz, 1H), 7.77 (td, J = 7.7, 1.8 Hz, 1H), 7.38 – 7.34 (m, 1H), 5.27 (q, J = 6.3 Hz, 1H), 1.35 (s, 3H), 1.34 (s, 3H), 1.08 (d, J = 6.3 Hz, 3H), 0.96 – 0.76 (m, 14H), -0.13 (s, 3H).

^{13}C NMR (126 MHz, $CDCl^3$) δ 205.1, 155.1, 147.5, 136.6, 125.8, 123.8, 71.9, 54.0, 20.6, 20.4, 18.9, 17.54, 17.52, 17.50, 13.9, 13.5, -7.1.

IR (KBr) ν 2938, 2865, 1688, 1464, 1112, 1091, 960, 785, 745.

HRMS (ESI) m/z: Calc. For $C_{18}H_{32}NO_2Si$ ($[M+H]^+$) 322.2197, Found 322.2192.

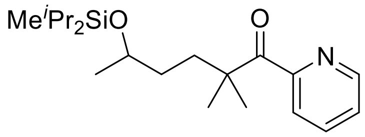

(why-09-63-5)

5-((diisopropyl(methyl)silyl)oxy)-2,2-dimethyl-1-(pyridin-2-yl)hexan-1-one (3ap)

37.7 mg, 36% yield, Light yellow oil, R_f = 0.7 (petroleum ether/ethyl acetate = 10:1).

^{1}H NMR (500 MHz, $CDCl_3$) δ 8.59 (dd, *J* = 5.2, 1.8 Hz, 1H), 7.86 (dd, *J* = 7.8, 1.1 Hz, 1H), 7.80 – 7.73 (m, 1H), 7.38 – 7.33 (m, 1H), 3.72 – 3.65 (m, 1H), 2.15 – 2.07 (m, 1H), 2.01 – 1.93 (m, 1H), 1.40 (s, 3H), 1.39 (s, 3H), 1.31 – 1.24 (m, 2H), 1.04 (d, *J* = 6.1 Hz, 3H), 0.97 – 0.79 (m, 14H), -0.07 (s, 3H).

^{13}C NMR (126 MHz, $CDCl_3$) δ 206.7, 155.2, 147.7, 136.6, 125.7, 123.4, 69.0, 47.5, 36.2, 35.0, 25.63, 25.59, 23.4, 17.47, 17.45, 17.4, 13.4, 13.3, -8.0.

IR (KBr) ν 2929, 2865, 1685, 1460, 1091, 997, 882, 781, 744.

HRMS (ESI) m/z: Calc. For $C_{20}H_{36}NO_2Si$ ($[M+H]^+$) 350.2510, Found 350.2506.

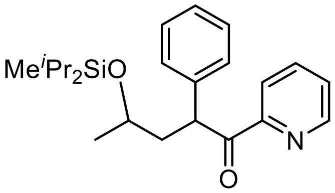

(wxh-1-100a)

4-((diisopropyl(methyl)silyl)oxy)-2-phenyl-1-(pyridin-2-yl)pentan-1-one (3aq)

96.5 mg, 84% yield (according to typical procedure A with 5-trifluomethylpyridinone as the additive), d.r. = 1:1, Light yellow oil, R_f = 0.6 (petroleum ether/ethyl acetate = 10:1).

^{1}H NMR (500 MHz, $CDCl_3$) δ 8.67 – 8.61 (m, 1H), 8.01 – 7.95 (m, 1H), 7.76 – 7.67 (m, 1H), 7.43 – 7.33 (m, 2H), 7.26 – 7.21 (m, 2H), 7.17 – 7.11 (m, 2H), 5.68 – 5.29 (m, 1H), 3.92 – 3.74 (m, 1H), 2.51 – 2.41 (m, 1H), 2.02 – 1.88 (m, 1H), 1.24 – 1.13 (m, 3H), 1.03 – 0.85 (m, 14H), -0.15 – -0.18 (m, 3H).

^{13}C NMR (126 MHz, $CDCl_3$) δ 201.2, 200.9, 153.0, 148.80, 148.75, 139.8, 139.5, 136.6, 136.5, 129.1, 128.9, 128.43, 128.40, 126.73, 126.70, 126.61, 126.59, 122.7, 122.6, 66.98, 66.95, 47.1, 47.0, 42.92, 42.90, 24.1, 23.9, 17.6, 17.51, 17.48, 17.46, 17.39, 17.36, 13.5, 13.44, 13.39, 13.3, -7.6, -8.0.

IR (KBr) ν 2941, 2865, 1697, 1458, 1251, 1136, 1095, 1062, 995, 965, 882, 782, 740, 701.

HRMS (ESI) m/z: Calc. For $C_{23}H_{34}O_2NSi$ ($[M+H]^{+)}$ 384.2353, Found 384.2350.

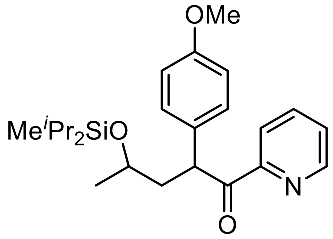

(why-09-149-1)

4-((diisopropyl(methyl)silyl)oxy)-2-(4-methoxyphenyl)-1-(pyridin-2-yl)pentan-1-one (3ar)

74.1 mg, 60% yield (according to typical procedure A with 5-trifluomethylpyridinone as the additive), d.r. = 1:1, Colorless oil, R_f = 0.3 (petroleum ether/ethyl acetate = 10:1).

^{1}H NMR (500 MHz, $CDCl_3$) δ 8.64 (d, J = 5.3 Hz, 1H), 7.98 (d, J = 7.8 Hz, 1H), 7.72 (t, J = 7.7 Hz, 1H), 7.37 – 7.27 (m, 3H), 6.81 – 6.75 (m, 2H), 5.60 – 5.48 (m, 1H), 3.72 (s, 3H), 3.64 – 3.60 (m, 1H), 2.43 (m, 1H), 2.08 – 1.87 (m, 1H), 1.23 – 1.10 (m, 3H), 0.92 (m, 14H), -0.08 – -0.15 (m, 3H).

^{13}C NMR (126 MHz, $CDCl_3$) δ 201.1, 201.0, 158.5, 158.4, 153.23, 153.15, 148.78, 148.75, 148.7, 136.6, 130.1, 130.0, 129.9, 126.6, 122.6, 113.94, 113.92, 67.04, 66.96, 61.3, 55.1, 46.3, 46.1, 36.0, 24.2, 23.8, 17.6, 17.5, 17.4, 17.3, 13.4, 13.3, 13.0, -7.5, -7.9.

IR (KBr) ν 2942, 2865, 1696, 1510, 1463, 1251, 1101, 995, 882, 807, 783, 754.

HRMS (ESI) m/z: Calc. For $C_{24}H_{36}O_3NSi$ ($[M+H]^{+}$) 414.2459, Found 414.2456.

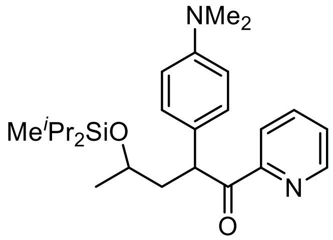

(wxh-01-100d)

4-((diisopropyl(methyl)silyl)oxy)-2-(4-(dimethylamino)phenyl)-1-(pyridin-2-yl)pentan-1-one (3as)

79.7 mg, 62% yield (according to typical procedure A with 5-trifluomethylpyridinone as the additive), d.r. = 1:1, Red oil, R_f = 0.1 (petroleum ether/ethyl acetate = 10:1).

^{1}H NMR (500 MHz, $CDCl_3$) δ 8.64 (d, J = 5.0 Hz, 1H), 7.97 (d, J = 7.8 Hz, 1H), 7.71 (d, J = 7.8 Hz, 1H), 7.36 – 7.30 (m, 1H), 7.25 (d, J = 8.5 Hz, 2H), 6.62 (d, J = 8.5 Hz, 2H), 5.50 – 5.43 (m, 1H), 3.63 (t, J = 6.5 Hz, 1H), 2.86 (s, 6H), 2.49 – 2.39 (m, 1H), 2.08 – 1.93 (m, 1H), 1.21 – 0.99 (m, 3H), 0.98 – 0.86 (m, 14H), -0.15 – -0.18 (m, 3H).

^{13}C NMR (126 MHz, $CDCl_3$) δ 201.1, 201.0, 153.5, 149.4, 148.7, 136.5, 136.4, 129.73, 129.66, 129.6, 126.48, 126.46, 122.7, 122.6, 112.74, 112.69, 112.67, 67.1, 67.0, 61.5, 46.1, 40.54, 40.51, 36.0, 24.1, 23.9, 17.5, 17.39, 17.37, 17.3, 13.5, 13.4, 13.3, 13.0, -7.6, -7.9.

IR (KBr) ν 2941, 2892, 2864, 1693, 1612, 1520, 1462, 1349, 1250, 1103, 1062, 995, 882, 807, 746.

HRMS (ESI) m/z: Calc. For $C_{25}H_{39}O_2N_2Si$ ($[M+H]^{+}$) 427.2775, Found 427.2771.

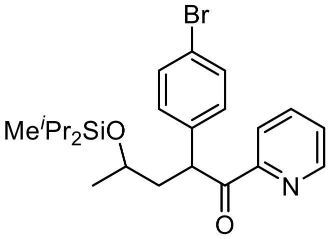

(why-09-143-2)

2-(4-bromophenyl)-4-((diisopropyl(methyl)silyl)oxy)-1-(pyridin-2-yl)pentan-1-one (3at)

81.1 mg, 63% yield (according to typical procedure A with 5-trifluomethylpyridinone as the additive), d.r. = 1:1, Colorless oil, R_f = 0.6 (petroleum ether/ethyl acetate = 10:1).

^{1}H NMR (500 MHz, $CDCl_3$) δ 8.69 – 8.56 (m, 1H), 8.05 – 7.91 (m, 1H), 7.79 – 7.70 (m, 1H), 7.41 – 7.33 (m, 3H), 7.31 – 7.23 (m, 2H), 5.66 – 5.53 (m, 1H), 3.84 – 3.74 (m, 1H), 2.47 – 2.38 (m, 1H), 1.95 – 1.85 (m, 1H), 1.22 – 1.11 (m, 3H), 1.01 – 0.79 (m, 14H), -0.15 – -0.18 (m, 3H).

^{13}C NMR (126 MHz, $CDCl_3$) δ 200.7, 200.4, 152.7, 148.83, 148.77, 138.9, 138.7, 136.70, 136.66, 131.6, 131.5, 130.8, 130.7, 126.94, 126.90, 122.73, 122.67, 120.7, 120.6, 66.9, 46.4, 42.73, 42.71, 24.1, 23.9, 17.6, 17.50, 17.46, 17.44, 17.38, 17.35, 13.54, 13.46, 13.4, 13.3, -7.5, -7.9.

IR (KBr) ν 2942, 2892, 2865, 1697, 1486, 1462, 1252, 1137, 1093, 1072, 1010, 996, 883, 784, 741.

HRMS (ESI) m/z: Calc. For $C_{23}H_{33}O_2NBrSi$ ($[M+H]^{+}$) 462.1458, Found 462.1452.

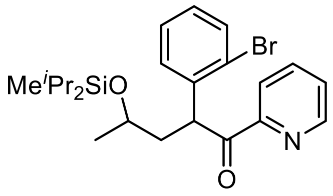

(why-09-143-3)

2-(2-bromophenyl)-4-((diisopropyl(methyl)silyl)oxy)-1-(pyridin-2-yl)pentan-1-one (3au)

60.1 mg, 43% yield (according to typical procedure A with 5-trifluomethylpyridinone as the additive), d.r. = 1:1, Colorless oil, R_f = 0.6 (petroleum ether/ethyl acetate = 10:1).

^{1}H NMR (500 MHz, $CDCl_3$) δ 8.63 (d, J = 4.9 Hz, 1H), 8.00 (d, J = 7.8 Hz, 1H), 7.84 – 7.71 (m, 1H), 7.55 (d, J = 8.1 Hz, 1H), 7.36 (d, J = 4.7 Hz, 1H), 7.26 – 7.14 (m, 2H), 7.02 (d, J = 7.6 Hz, 1H), 5.95 – 5.84 (m, 1H), 3.99 – 3.84 (m, 1H), 2.51 – 2.77 (m, 1H), 1.95 – 1.79 (m, 1H), 1.31 – 1.18 (m, 3H), 1.04 – 0.79 (m, 14H), -0.15 – -0.18 (m, 3H).

^{13}C NMR (126 MHz, $CDCl_3$) δ 200.86, 200.85, 152.9, 149.1, 149.0, 139.5, 136.6, 136.5, 133.30, 133.25, 129.3, 129.1, 128.1, 128.0, 127.4, 127.3, 126.82, 126.76, 125.2, 122.5, 122.4, 67.3, 66.9, 47.6, 47.1, 42.8, 42.5, 24.1, 23.7, 17.49, 17.46, 17.45, 17.42, 17.39, 13.4, 13.3, -7.8, -7.9.

IR (KBr) ν 2941, 2893, 2865, 1698, 1468, 1435, 1251, 1137, 1096, 1062, 1024, 996, 883, 783, 749.

HRMS (ESI) m/z: Calc. For $C_{23}H_{33}O_2NBrSi$ ($[M+H]^{+}$) 462.1458, Found 462.1453.

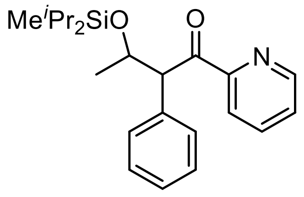

(why-09-149-4)

3-((diisopropyl(methyl)silyl)oxy)-2-phenyl-1-(pyridin-2-yl)butan-1-one (3av)

103.9 mg, 94% yield (according to typical procedure A with 5-trifluomethylpyridinone as the additive), d.r. > 20:1, Light yellow oil, R_f = 0.6 (petroleum ether/ethyl acetate = 10:1).

^{1}H NMR (500 MHz, $CDCl_3$) δ 8.81 (d, J = 5.0 Hz, 1H), 8.15 (d, J = 7.8 Hz, 1H), 7.91 – 7.86 (m, 1H), 7.62 (d, J = 7.6 Hz, 2H), 7.53 (dd, J = 7.6, 4.6 Hz, 1H), 7.38 (t, J = 7.6 Hz, 2H), 7.32 (t, J = 7.5 Hz, 1H), 5.61 (d, J = 8.7 Hz, 1H), 4.79 – 4.74 (m, 1H), 1.41 (d, J = 6.0 Hz, 3H), 1.07 – 0.81 (m, 14H), -0.12 (s, 3H).

^{13}C NMR (126 MHz, $CDCl_3$) δ 200.9, 153.4, 148.9, 137.2, 136.7, 130.2, 128.0, 126.9, 126.8, 122.5, 71.2, 59.3, 23.2, 17.47, 17.45, 17.3, 13.6, 13.2, -7.8.

IR (KBr) ν 2942, 2893, 2865, 1692, 1460, 1307, 1251, 1132, 994, 883, 782, 743, 702.

HRMS (ESI) m/z: Calc. For $C_{22}H_{32}O_2NSi$ ($[M+H]^+$) 370.2197, Found 370.2195.

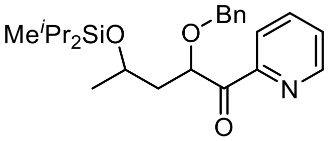

(why-09-143-4)

2-(benzyloxy)-4-((diisopropyl(methyl)silyl)oxy)-1-(pyridin-2-yl)pentan-1-one (3aw)

93.8 mg, 76% yield (according to typical procedure A with 5-trifluomethylpyridinone as the additive), d.r. = 1:1, Colorless oil, R_f = 0.4 (petroleum ether/ethyl acetate = 10:1).

^{1}H NMR (500 MHz, $CDCl_3$) δ 8.69 – 8.62 (m, 1H), 8.03 – 7.94 (m, 1H), 7.76 (td, J = 7.8, 1.9 Hz, 1H), 7.58 – 7.50 (m, 2H), 7.43 – 7.38 (m, 1H), 7.29 – 7.19 (m, 2H), 6.50 (d, J = 8.9 Hz, 1H), 4.05 – 3.96 (m, 1H), 3.67 – 3.54 (m, 2H), 1.87 – 1.74 (m, 2H), 1.17 – 1.10 (m, 3H), 0.97 – 0.84 (m, 14H), -0.02 – -0.05 (m, 3H).

^{13}C NMR (126 MHz, $CDCl_3$) δ 197.6, 197.1, 148.69, 148.66, 136.8, 136.6, 128.6, 128.5, 128.4, 128.3, 128.10, 128.06, 127.1, 122.94, 122.92, 81.7, 81.5, 66.7, 66.4, 66.01, 65.97, 39.73, 39.70, 24.0, 23.9, 17.49, 17.46, 17.44, 17.43, 13.4, 13.3, -7.86, -7.88.

IR (KBr) ν 2942, 2893, 2865, 1710, 1459, 1252, 1109, 1028, 995, 882, 782, 741, 701.

HRMS (ESI) m/z: Calc. For $C_{24}H_{36}O_3NSi$ ($[M+H]^+$) 414.2459, Found 414.2458.

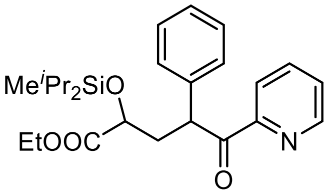

(why-09-158-7)

ethyl 2-((diisopropyl(methyl)silyl)oxy)-5-oxo-4-phenyl-5-(pyridin-2-yl)pentanoate (3ax)

83.7 mg, 63% yield (according to typical procedure A with 5-trifluomethylpyridinone as the additive), d.r. = 1:1, Colorless oil, R_f = 0.2 (petroleum ether/ethyl acetate = 10:1).

^{1}H NMR (500 MHz, $CDCl_3$) δ 8.53 – 8.48 (m, 1H), 7.90 – 7.83 (m, 1H), 7.60 (td, *J* = 7.7, 1.8 Hz, 1H), 7.32 – 7.20 (m, 3H), 7.15 – 7.08 (m, 2H), 7.03 (d, *J* = 8.0, 1H), 5.59 – 5.52 (m, 1H), 4.14 – 4.07 (m, 1H), 4.02 – 3.94 (m, 2H), 2.77 – 2.60 (m, 1H), 2.24 – 2.04 (m, 1H), 1.15 – 1.06 (m, 3H), 0.90 – 0.74 (m, 14H), -0.14 – -0.30 (m, 3H).

^{13}C NMR (126 MHz, $CDCl_3$) δ 200.2, 200.1, 173.5, 173.3, 152.8, 148.8, 139.1, 138.6, 136.53, 136.48, 129.1, 129.0, 128.49, 128.45, 128.42, 128.40, 128.38, 128.37, 128.34, 128.29, 126.9, 126.8, 126.7, 122.62, 122.59, 70.8, 60.6, 46.4, 46.3, 38.4, 38.2, 17.4, 17.3, 17.2, 17.1, 14.1, 13.2, 13.14, 13.12, -8.2, -8.5.

IR (KBr) ν 2942, 2866, 1751, 1698, 1583, 1493, 1463, 1365, 1133, 996, 883, 781, 701.

HRMS (ESI) m/z: Calc. For $C_{25}H_{36}O_4NSi$ ([M+H]$^{+}$) 442.2408, Found 442.2404.

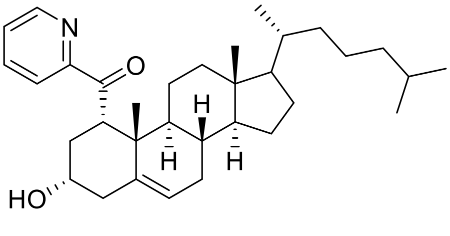

(why-09-67-6)

((1S,3S,8S,9S,10S,13R,14S)-3-hydroxy-10,13-dimethyl-17-((R)-5-methylhexan-2-yl)hexadecahydro-1H-cyclopenta[a]phenanthren-1-yl)(pyridin-2-yl)methanone (3ay')

81.0 mg, 55% yield, d.r. > 20:1, Colorless oil, R_f = 0.4 (petroleum ether/ethyl acetate = 10:1).

^{1}H NMR (500 MHz, $CDCl_3$) δ 8.64 (d, J = 4.9 Hz, 1H), 8.05 (d, J = 7.8 Hz, 1H), 7.82 – 7.80 (m, 1H), 7.43 (dd, J = 7.8, 4.6 Hz, 1H), 5.03 – 4.96 (m, 1H), 4.89 – 4.85 (m, 1H), 4.07 (s, 1H), 2.06 – 1.98 (m, 1H), 1.91 – 1.71 (m, 5H), 1.67 – 1.47 (m, 6H), 1.45 – 1.22 (m, 14H), 0.94 – 0.84 (m, 12H), 0.71 (s, 3H).

^{13}C NMR (126 MHz, $CDCl_3$) δ 204.3, 153.8, 149.0, 147.0, 136.9, 127.0, 124.1, 122.0, 67.7, 56.2, 55.9, 54.5, 44.6, 42.7, 39.9, 39.5, 38.2, 36.2, 35.8, 35.2, 34.7, 28.8, 28.2, 28.0, 24.2, 23.9, 22.8, 22.5, 21.2, 19.7, 18.7, 12.0.

IR (KBr) ν 3366, 2932, 2868, 1696, 1464, 1376.

HRMS (ESI) m/z: Calc. For $C_{33}H_{50}NO_2$ ($[M+H]^+$) 492.3836, Found 492.3829.

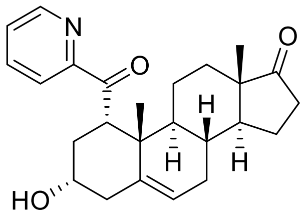

(why-09-55-8)

(1S,3R,8R,9S,10S,13S,14S)-3-hydroxy-10,13-dimethyl-1-picolinoyl-1,2,3,4,7,8,9,10,11,12,13,14,15,16-tetradecahydro-17H-cyclopenta[a]phenanthren-17-one (3az’)

81.4 mg, 69 % yield, d.r. > 20:1, Colorless oil, R_f = 0.2 (petroleum ether/ethyl acetate = 4:1).

^{1}H NMR (500 MHz, $CDCl_3$) δ 8.65 (d, J = 4.9 Hz, 1H), 8.06 (d, J = 7.8 Hz, 1H), 7.87 – 7.80 (m, 1H), 7.45 (dd, J = 7.6, 4.7 Hz, 1H), 5.08 – 5.01 (m, 1H), 4.89 (d, J = 1.7 Hz, 1H), 4.09 – 4.05 (m, 1H), 2.48 – 2.40 (m, 1H), 2.13 – 2.05 (m, 1H), 1.94 – 1.82 (m, 4H), 1.80 – 1.74 (m, 1H), 1.71 – 1.64 (m, 1H), 1.56 – 1.48 (m, 2H), 1.45 – 1.32 (m, 4H), 1.32 – 1.23 (m, 5H), 1.03 – 0.94 (m, 1H), 0.92 (s, 3H).

^{13}C NMR (126 MHz, $CDCl_3$) δ 220.5, 204.0, 153.6, 149.1, 146.2, 137.0, 127.1, 124.8, 122.1, 67.5, 54.6, 51.0, 47.7, 44.3, 38.3, 35.8, 35.2, 34.8, 33.5, 31.5, 28.7, 21.8, 20.5, 19.7, 13.8.

IR (KBr) ν 3445, 2932, 2855, 1735, 1696, 1456, 1374, 730.

HRMS (ESI) m/z: Calc. For $C_{25}H_{32}NO_3$ ($[M+H]^+$) 394.2377, Found 394.2375.

2.3.2 Three-component reaction

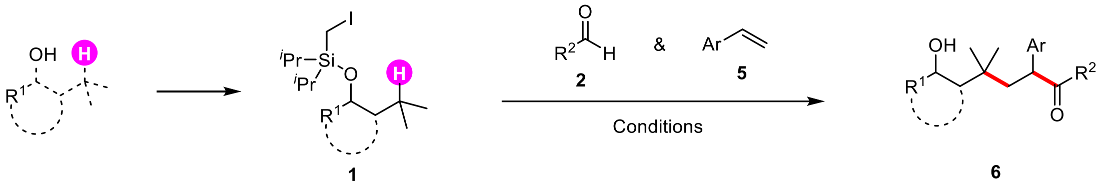

Typical procedure B: A 5 mL vial equipped with a stir bar was charged with **N2** (0.06 mmol), $Pd(OAc)_2$ (0.03 mmol), PCy_3 (0.06 mmol) and 2.0 mL of $PhCF_3$. After stirring for 30 min in glove box, to the solution was added Cs_2CO_3 (97.8 mg, 0.6 mmol), aldehydes **2** (0.45 mmol), styrenes **5** (0.6 mmol), silyl ether derived from varied alcohols **1** (0.3 mmol), and 1.0 mL of $PhCF_3$. The reaction mixture was removed from the glove box and stirred under 36W Blue LEDs at room temperature for 16 h. Then, TBAF (2.0 equiv, 1.0 M in THF) was added. After being stirred for 2 h, The solution was concentrated under reduced pressure, and purified by column chromatography on silica gel to afford the desired ketones **6**.

Unsuccessful alkenes:

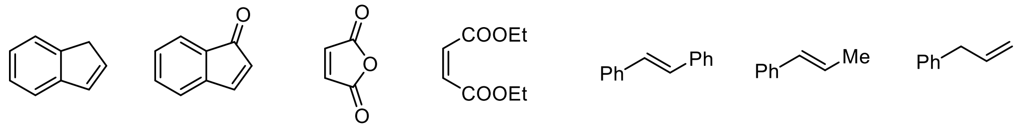

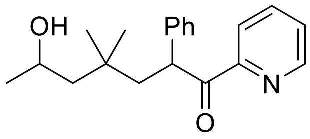

(why-09-35-1)

6-hydroxy-4,4-dimethyl-2-phenyl-1-(pyridin-2-yl)heptan-1-one (6a)

86.8 mg, 93% yield, d.r. = 1:1, Light yellow oil, R_f = 0.2 (petroleum ether/ethyl acetate = 4:1).

^{1}H NMR (500 MHz, $CDCl_3$) δ 8.69 (d, *J* = 4.9 Hz, 1H), 7.98 (d, *J* = 7.9 Hz, 1H), 7.77 – 7.72 (m, 1H), 7.43 – 7.37 (m, 3H), 7.27 – 7.18 (m, 2H), 7.13 (dd, *J* = 7.5, 3.8 Hz, 1H), 5.71 – 5.63 (m, 1H), 3.99 – 3.94 (m, 1H), 2.69 – 2.60 (m, 1H), 1.80 – 1.70 (m, 2H), 1.53 – 1.43 (m, 1H), 1.40 – 1.34 (m, 1H) 1.15 (d, *J* = 6.1 Hz, 3H), 0.97 – 0.86 (m, 6H).

^{13}C NMR (126 MHz, $CDCl_3$) δ 201.7, 201.5, 153.0, 148.9, 148.8, 141.0, 140.8, 136.83, 136.75, 129.0, 128.9, 128.5, 126.9, 126.8, 126.6, 126.5, 123.0, 122.9, 65.4, 51.4, 51.0, 46.3, 46.1, 45.5, 45.3, 33.8, 33.7, 28.4, 28.22, 28.16, 28.1, 26.00, 25.97.

IR (KBr) ν 3421, 2959, 2926, 1696, 1456, 742, 702.

HRMS (ESI) m/z: Calc. For $C_{20}H_{25}NONa$ ($[M+Na]^+$) 334.1778, Found 334.1775.

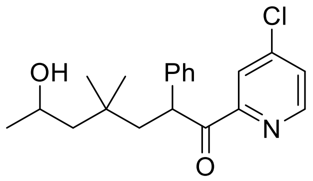

(why-09-45-1)

1-(4-chloropyridin-2-yl)-6-hydroxy-4,4-dimethyl-2-phenylheptan-1-one (6b)

67.3 mg, 65% yield, d.r. = 1:1, Colorless oil, R_f = 0.4 (petroleum ether/ethyl acetate = 4:1).

^{1}H NMR (500 MHz, $CDCl_3$) δ 8.59 (dd, *J* = 5.3, 1.9 Hz, 1H), 7.97 (d, *J* = 2.1 Hz, 1H), 7.40 (dt, *J* = 5.3, 2.0 Hz, 1H), 7.36 (dt, *J* = 8.1, 1.7 Hz, 2H), 7.22 (td, *J* = 7.6, 2.0 Hz, 2H), 7.18 – 7.10 (m, 1H), 5.62 – 5.55 (m, 1H), 3.99 – 3.93 (m, 1H), 2.69 – 2.59 (m, 1H), 1.76 – 1.70 (m, 1H), 1.50 – 1.44 (m, 1H), 1.39 – 1.34 (m, 1H), 1.17 – 1.13 (m, 3H), 0.96 – 0.86 (m, 6H).

^{13}C NMR (126 MHz, $CDCl_3$) δ 200.4, 200.2, 154.2, 149.78, 149.76, 145.5, 145.4, 140.5, 140.3, 129.0, 128.9, 128.6, 126.99, 126.95, 126.8, 126.7, 123.43, 123.36, 65.42, 65.38, 51.4, 51.1, 46.6, 46.5, 45.4, 45.2, 33.8, 33.7, 28.3, 28.14, 28.08, 26.1.

IR (KBr) ν 3377, 2958, 2926, 1697, 1568, 1454, 1391, 1366, 1287, 1213, 713.

HRMS (ESI) m/z: Calc. For $C_{20}H_{24}ClNO_2Na$ ($[M+ Na]^+$) 368.1388, Found 368.1386.

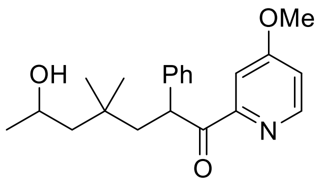

(why-09-43-1)

6-hydroxy-1-(4-methoxypyridin-2-yl)-4,4-dimethyl-2-phenylheptan-1-one (6c)

90.0 mg, 88% yield, d.r. = 1:1, Colorless oil, R_f = 0.1 (petroleum ether/ethyl acetate = 4:1).

^{1}H NMR (500 MHz, $CDCl_3$) δ 8.51 – 8.47 (m, 1H), 7.51 (d, J = 2.6 Hz, 1H), 7.39 (dd, J = 7.9, 2.4 Hz, 2H), 7.24 – 7.19 (m, 2H), 7.17 – 7.10 (m, 1H), 6.91 (dt, J = 6.0, 2.8 Hz, 1H), 5.71 – 5.62 (m, 1H), 3.99 – 3.93 (m, 1H), 3.83 (s, 3H), 2.69 – 2.58 (m, 1H), 1.79 – 1.69 (m, 1H), 1.54 – 1.43 (m, 1H), 1.40 – 1.33 (m, 1H), 1.17 – 1.12 (m, 3H), 0.97 – 0.84 (m, 6H).

^{13}C NMR (126 MHz, $CDCl_3$) δ 201.7, 201.5, 166.6, 166.5, 154.9, 149.99, 149.97, 141.0, 140.7, 129.0, 128.9, 128.47, 128.45, 126.6, 126.5, 113.81, 113.75, 108.0, 107.9, 65.4, 65.3, 55.32, 55.30, 51.4, 51.0, 46.5, 46.2, 45.5, 45.2, 33.8, 33.7, 28.5, 28.3, 28.2, 28.1, 26.02, 25.97.

IR (KBr) ν 3421, 2960, 2925, 1696, 1592, 1475, 1306, 1036, 995, 838, 737, 699.

HRMS (ESI) m/z: Calc. For $C_{21}H_{28}NO_3$ ($[M+H]^+$) 342.2064, Found 342.2065.

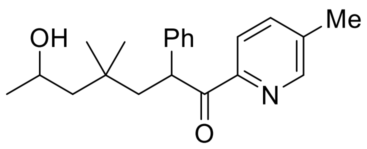

(why-09-43-5)

6-hydroxy-4,4-dimethyl-1-(5-methylpyridin-2-yl)-2-phenylheptan-1-one (6d)

56.6 mg, 58% yield, d.r. = 1:1, Light yellow oil, R_f = 0.2 (petroleum ether/ethyl acetate = 4:1).

^{1}H NMR (500 MHz, $CDCl_3$) δ 8.53 – 8.49 (m, 1H), 7.90 (dd, J = 8.0, 2.5 Hz, 1H), 7.55 – 7.52 (m, 1H), 7.40 (dt, J = 8.1, 1.6 Hz, 2H), 7.27 – 7.21 (m, 1H), 7.21 – 7.18 (m, 1H), 7.14 – 7.11 (m, 1H), 5.69 – 5.62 (m, 1H), 4.00 – 3.92 (m, 1H), 2.69 – 2.60 (m, 1H),

2.37 (s, 3H), 1.78 – 1.68 (m, 2H), 1.50 – 1.40 (m, 1H), 1.39 – 1.33 (m, 1H), 1.16 – 1.13 (m, 3H), 0.96 – 0.85 (m, 6H).

^{13}C NMR (126 MHz, $CDCl_3$) δ 201.6, 201.3, 150.69, 150.66, 149.38, 149.35, 141.2, 141.0, 137.4, 137.3, 137.2, 137.1, 129.0, 128.9, 128.44, 128.42, 126.5, 126.4, 122.7, 122.6, 65.38, 65.36, 51.4, 51.0, 46.3, 46.0, 45.5, 45.2, 33.8, 33.7, 28.5, 28.3, 28.2, 28.1, 25.99, 25.95, 18.6.

IR (KBr) ν 3430, 2960, 2927, 2870, 1692, 1453, 1367, 1208, 1125, 728, 699.

HRMS (ESI) m/z: Calc. For $C_{21}H_{27}NO_2Na$ ($[M+ Na]^+$) 348.1934, Found 348.1933.

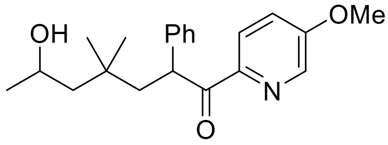

(why-09-49-3)

6-hydroxy-1-(5-methoxypyridin-2-yl)-4,4-dimethyl-2-phenylheptan-1-one (6e)

96.2 mg, 94% yield, d.r. = 1:1, Colorless oil, R_f = 0.1 (petroleum ether/ethyl acetate = 4:1).

^{1}H NMR (500 MHz, $CDCl_3$) δ 8.35 (t, *J* = 2.4 Hz, 1H), 8.00 (dd, *J* = 8.7, 3.4 Hz, 1H), 7.40 (dt, *J* = 8.1, 2.0 Hz, 2H), 7.27 – 7.10 (m, 5H), 5.66 – 5.60 (m, 1H), 3.98 – 3.94 (m, 1H), 3.89 (s, 3H), 2.70 – 2.58 (m, 1H), 1.77 – 1.66 (m, 1H), 1.50 – 1.41 (m, 1H), 1.39 – 1.33 (m, 1H), 1.14 (d, *J* = 6.1 Hz, 3H), 0.96 – 0.87 (m, 6H).

^{13}C NMR (126 MHz, $CDCl_3$) δ 200.6, 200.3, 158.4, 158.3, 146.2, 141.5, 141.3, 136.8, 128.91, 128.86, 128.4, 126.5, 126.4, 124.6, 124.5, 120.2, 120.1, 65.4, 55.74, 55.73, 51.4, 51.0, 46.2, 46.0, 45.6, 45.3, 33.8, 33.7, 28.4, 28.3, 28.2, 28.1, 26.00, 25.96.

IR (KBr) ν 3361, 2959, 2923, 1685, 1578, 1311, 1270, 1211, 1126, 700.

HRMS (ESI) m/z: Calc. For $C_{21}H_{27}NO_3Na$ ($[M+ Na]^+$) 364.1883, Found 364.1882.

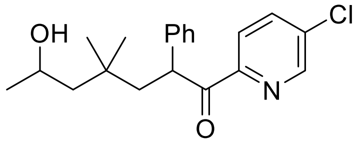

(why-09-45-2)

1-(5-chloropyridin-2-yl)-6-hydroxy-4,4-dimethyl-2-phenylheptan-1-one (6f)

72.5 mg, 70% yield, d.r. = 1:1, Colorless oil, R_f = 0.4 (petroleum ether/ethyl acetate = 4:1).

^{1}H NMR (500 MHz, $CDCl_3$) δ 8.64 (t, *J* = 2.1 Hz, 1H), 7.95 (d, *J* = 8.4 Hz, 1H), 7.72 (m, 1H), 7.37 (dt, *J* = 8.1, 2.1 Hz, 2H), 7.22 (td, *J* = 7.7, 1.8 Hz, 2H), 7.17 – 7.10 (m, 1H), 5.60 – 5.53 (m, 1H), 3.99 – 3.92 (m, 1H), 2.69 – 2.59 (m, 1H), 1.78 – 1.70 (m, 1H), 1.49 – 1.42 (m, 1H), 1.39 – 1.32 (m, 1H), 1.17 – 1.13 (m, 3H), 0.95 – 0.87 (m, 6H).

^{13}C NMR (126 MHz, $CDCl_3$) δ 200.5, 200.3, 150.9, 147.9, 140.7, 140.5, 136.7, 136.6, 135.94, 135.90, 128.92, 128.89, 128.6, 126.70, 126.67, 123.9, 123.8, 65.43, 65.37, 51.3, 51.1, 46.4, 46.3, 45.4, 45.2, 33.8, 33.7, 28.3, 28.14, 28.11, 28.07, 26.1, 26.0.

IR (KBr) ν 3429, 2959, 2927, 1696, 1456, 1369, 1108, 1012, 712, 699.

HRMS (ESI) m/z: Calc. For $C_{20}H_{24}ClNO_2Na$ ([M+ Na]$^+$) 368.1388, Found 368.1387.

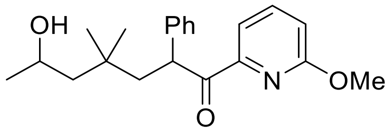

(why-09-43-3)

6-hydroxy-1-(6-methoxypyridin-2-yl)-4,4-dimethyl-2-phenylheptan-1-one (6g)

66.5 mg, 65% yield, d.r. = 1:1, Light yellow oil, R_f = 0.3 (petroleum ether/ethyl acetate = 4:1).

^{1}H NMR (500 MHz, $CDCl_3$) δ 7.62 (dd, *J* = 5.3, 2.7 Hz, 2H), 7.37 (dd, *J* = 7.3, 5.2 Hz, 2H), 7.23 (t, *J* = 7.6 Hz, 2H), 7.14 (d, *J* = 1.8 Hz, 1H), 6.89 (dd, *J* = 6.5, 2.7 Hz, 1H), 5.62 – 5.54 (m, 1H), 4.08 (s, 3H), 4.01 – 3.93 (m, 1H), 2.71 – 2.60 (m, 1H), 1.78 – 1.70 (m, 1H), 1.49 – 1.43 (m, 1H), 1.39 – 1.33 (m, 1H), 1.15 – 1.11 (m, 3H), 0.98 – 0.90 (m, 6H).

^{13}C NMR (126 MHz, $CDCl_3$) δ 201.4, 201.0, 163.2, 150.4, 141.1, 141.0, 139.19, 139.17, 128.7, 128.6, 128.5, 126.59, 126.56, 116.4, 116.3, 115.4, 115.3, 65.4, 65.3, 53.59, 53.57, 51.3, 51.1, 46.5, 46.4, 45.7, 45.6, 33.7, 28.2, 28.1, 28.0, 26.0.

IR (KBr) ν 3433, 2958, 2925, 1694, 1590, 1467, 1325, 1273, 1033, 810, 698.

HRMS (ESI) m/z: Calc. For $C_{21}H_{27}NO_3Na$ ([M+ Na]$^+$) 364.1883, Found 364.1880.

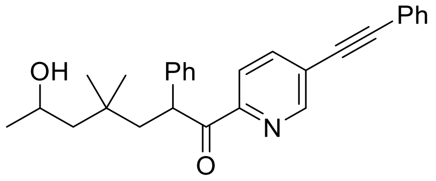

(why-09-41-1)

6-hydroxy-4,4-dimethyl-2-phenyl-1-(5-(phenylethynyl)pyridin-2-yl)heptan-1-one (6h)

97.4 mg, 79% yield, d.r. = 1:1, Colorless oil, R_f = 0.5 (petroleum ether/ethyl acetate = 3:1).

^{1}H NMR (500 MHz, $CDCl_3$) δ 8.81 (d, *J* = 1.5 Hz, 1H), 7.97 (d, *J* = 8.1 Hz, 1H), 7.84 (dd, *J* = 8.2, 1.9 Hz, 1H), 7.54 (dd, *J* = 6.4, 3.4 Hz, 2H), 7.43 – 7.35 (m, 5H), 7.26 – 7.20 (m, 2H), 7.17 – 7.11 (m, 1H), 5.68 – 5.60 (m, 1H), 4.01 – 3.93 (m, 1H), 2.70 – 2.60 (m, 1H), 1.80 – 1.71 (m, 1H), 1.52 – 1.44 (m, 1H), 1.40 – 1.34 (m, 1H), 1.17 – 1.13 (m, 3H), 0.97 – 0.87 (m, 6H).

^{13}C NMR (126 MHz, $CDCl_3$) δ 201.0, 200.7, 151.24, 151.22, 151.08, 151.07, 140.8, 140.7, 139.12, 139.07, 131.8, 129.2, 129.0, 128.9, 128.53, 128.50, 126.62, 126.59, 123.92, 123.86, 122.3, 122.21, 122.18, 95.52, 95.46, 85.80, 85.77, 65.41, 65.38, 51.4, 51.1, 46.4, 46.3, 45.4, 45.2, 33.8, 33.7, 28.4, 28.20, 28.15, 28.1, 26.02, 26.00.

HRMS (ESI) m/z: Calc. For $C_{28}H_{29}NO_2Na$ ([M+ Na]$^+$) 434.2091, Found 434.2089.

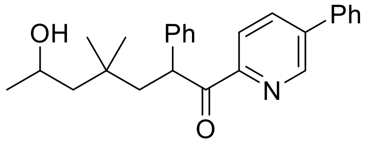

(why-09-41-2)

6-hydroxy-4,4-dimethyl-2-phenyl-1-(5-phenylpyridin-2-yl)heptan-1-one (6i)

97.5 mg, 84% yield, d.r. = 1:1, Colorless oil, R_f = 0.5 (petroleum ether/ethyl acetate = 3:1).

^{1}H NMR (500 MHz, $CDCl_3$) δ 8.92 (t, *J* = 2.1 Hz, 1H), 8.06 (dd, *J* = 8.1, 2.0 Hz, 1H), 7.92 (dt, *J* = 8.1, 2.6 Hz, 1H), 7.62 – 7.56 (m, 2H), 7.50 – 7.41 (m, 5H), 7.24 (td, *J* =

7.6, 3.2 Hz, 2H), 7.14 (dd, J = 7.5, 4.0 Hz, 1H), 5.75 – 5.67 (m, 1H), 4.00 – 3.95 (m, 1H), 2.73 – 2.63 (m, 1H), 1.80 – 1.72 (m, 1H), 1.54 – 1.46 (m, 1H), 1.44 – 1.35 (m, 1H), 1.18 – 1.14 (m, 3H), 0.98 – 0.89 (m, 6H).

^{13}C NMR (126 MHz, $CDCl_3$) δ 201.4, 201.1, 151.60, 151.57, 147.4, 147.3, 141.1, 140.9, 139.7, 139.6, 137.0, 136.9, 135.0, 134.9, 129.2, 129.0, 128.9, 128.8, 128.7, 128.5, 127.3, 126.6, 126.5, 123.1, 123.0, 65.4, 51.4, 51.0, 46.4, 46.3, 45.5, 45.3, 33.8, 33.7, 28.5, 28.3, 28.2, 28.1, 26.02, 25.99.

IR (KBr) ν 3423, 2959, 2927, 1691, 1453, 1370, 766, 724, 697.

HRMS (ESI) m/z: Calc. For $C_{26}H_{29}NO_2Na$ ($[M+ Na]^+$) 410.2091, Found 410.2088.

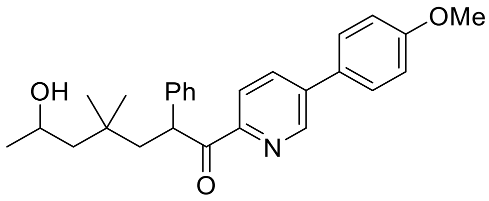

(why-09-41-4)

6-hydroxy-1-(5-(4-methoxyphenyl)pyridin-2-yl)-4,4-dimethyl-2-phenylheptan-1-one (6j)

88.8 mg, 71% yield, d.r. = 1:1, Colorless oil, R_f = 0.4 (petroleum ether/ethyl acetate = 3:1).

^{1}H NMR (500 MHz, $CDCl_3$) δ 8.89 (t, J = 2.1 Hz, 1H), 8.03 (dd, J = 8.2, 2.1 Hz, 1H), 7.90 – 7.84 (m, 1H), 7.54 (dd, J = 9.3, 2.6 Hz, 2H), 7.46 – 7.41 (m, 2H), 7.23 (td, J = 7.8, 3.1 Hz, 2H), 7.17 – 7.11 (m, 1H), 7.03 – 6.98 (m, 2H), 5.75 – 5.66 (m, 1H), 4.01 – 3.95 (m, 1H), 3.85 (s, 3H), 2.73 – 2.61 (m, 1H), 1.80 – 1.71 (m, 1H), 1.53 – 1.45 (m, 1H), 1.41 – 1.35 (m, 1H), 1.16 (d, J = 6.3 Hz, 3H), 0.99 – 0.88 (m, 6H).

^{13}C NMR (126 MHz, $CDCl_3$) δ 201.4, 201.1, 160.39, 160.36, 151.01, 150.98, 146.87, 146.85, 141.2, 141.0, 139.3, 139.2, 134.3, 134.2, 129.3, 129.2, 129.0, 128.9, 128.5, 128.4, 126.51, 126.47, 123.1, 123.0, 114.7, 65.4, 55.4, 51.4, 51.0, 46.4, 46.2, 45.5, 45.3, 33.8, 33.7, 28.5, 28.3, 28.2, 28.1, 26.00, 25.97.

IR (KBr) ν 3444, 2959, 2926, 1690, 1608, 1518, 1468, 1289, 1252, 1180, 828, 714.

HRMS (ESI) m/z: Calc. For $C_{27}H_{31}NO_3Na$ ($[M+ Na]^+$) 440.2196, Found 440.2194.

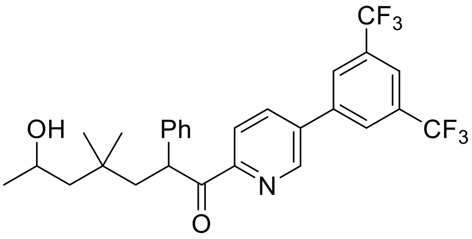

(why-09-41-7)

1-(5-(3,5-bis(trifluoromethyl)phenyl)pyridin-2-yl)-6-hydroxy-4,4-dimethyl-2-phenylheptan-1-one (6k)

112.9 mg, 72% yield, d.r. = 1:1, Light yellow oil, R_f = 0.7 (petroleum ether/ethyl acetate = 3:1).

^{1}H NMR (500 MHz, $CDCl_3$) δ 8.96 (t, *J* = 2.0 Hz, 1H), 8.13 (d, *J* = 8.1 Hz, 1H), 8.05 – 7.94 (m, 4H), 7.41 (d, *J* = 8.1 Hz, 2H), 7.24 (dd, *J* = 8.6, 6.2 Hz, 2H), 7.15 (dd, *J* = 7.9, 2.8 Hz, 1H), 5.71 – 5.64 (m, 1H), 4.02 – 3.97 (m, 1H), 2.73 – 2.64 (m, 1H), 1.82 – 1.73 (m, 1H), 1.52 – 1.45 (m, 1H), 1.42 – 1.35 (m, 1H), 1.16 (d, *J* = 6.3 Hz, 3H), 0.99 – 0.89 (m, 6H).

^{13}C NMR (126 MHz, $CDCl_3$) δ 200.9, 200.7, 152.9, 147.4, 147.3, 140.7, 140.6, 139.3, 136.7, 135.5, 135.4, 132.8 (q, $J_{C\text{-}F}$ = 34 Hz), 129.0, 128.9, 128.6, 127.4, 126.72, 126.69, 124.2, 123.3 (q, $J_{C\text{-}F}$ = 273 Hz), 123.2, 122.4, 122.0, 65.44, 65.40, 51.4, 51.1, 46.6, 46.5, 45.4, 45.2, 33.8, 33.7, 28.4, 28.2, 28.14, 28.12, 26.1, 26.0.

IR (KBr) ν 3443, 2961, 2925, 1696, 1384, 1280, 1183, 1138, 900, 702.

HRMS (ESI) m/z: Calc. For $C_{28}H_{27}F_6NO_2Na$ ($[M+ Na]^+$) 546.1838, Found 546.1834.

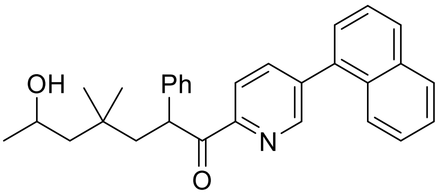

(why-09-41-3)

6-hydroxy-4,4-dimethyl-1-(5-(naphthalen-1-yl)pyridin-2-yl)-2-phenylheptan-1-one (6l)

127.2 mg, 97% yield, d.r. = 1:1, Light yellow oil, R_f = 0.5 (petroleum ether/ethyl acetate = 3:1).

^{1}H NMR (500 MHz, $CDCl_3$) δ 8.83 (d, *J* = 2.4 Hz, 1H), 8.13 (dd, *J* = 8.0, 1.0 Hz, 1H), 7.94 – 7.86 (m, 3H), 7.76 (d, *J* = 8.5 Hz, 1H), 7.57 – 7.43 (m, 5H), 7.40 (d, *J* = 7.0 Hz, 1H), 7.27 (dt, *J* = 7.9, 3.9 Hz, 2H), 7.19 – 7.14 (m, 1H), 5.78 – 5.71 (m, 1H), 4.04 – 3.95 (m, 1H), 2.77 – 2.66 (m, 1H), 1.84 – 1.75 (m, 1H), 1.56 – 1.46 (m, 1H), 1.45 – 1.38 (m, 1H), 1.19 – 1.15 (m, 3H), 1.01 – 0.93 (m, 6H).

^{13}C NMR (126 MHz, $CDCl_3$) δ 201.5, 201.2, 151.8, 151.7, 149.67, 149.65, 141.1, 140.9, 139.84, 139.76, 138.2, 138.1, 135.62, 135.57, 133.8, 131.2, 129.1, 129.02, 129.00, 128.6, 127.5, 126.77, 126.76, 126.60, 126.56, 126.2, 125.3, 125.0, 122.6, 122.5, 65.43, 65.41, 51.4, 51.1, 46.5, 46.4, 45.7, 45.4, 33.84, 33.77, 28.5, 28.3, 28.23, 28.17, 26.04, 26.01.

IR (KBr) ν 3447, 2959, 2927, 2869, 1693, 1454, 1368, 1215, 802, 778, 711, 698.

HRMS (ESI) m/z: Calc. For $C_{30}H_{31}NO_2Na$ ($[M+ Na]^+$) 460.2247, Found 460.2245.

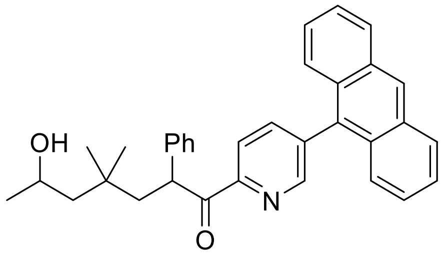

(why-09-41-5)

1-(5-(anthracen-9-yl)pyridin-2-yl)-6-hydroxy-4,4-dimethyl-2-phenylheptan-1-one (6m)

111.0 mg, 76% yield, d.r. = 1:1, Light yellow oil, R_f = 0.5 (petroleum ether/ethyl acetate = 3:1).

^{1}H NMR (500 MHz, $CDCl_3$) δ 8.76 (s, 1H), 8.55 (s, 1H), 8.23 (d, *J* = 7.9 Hz, 1H), 8.06 (t, *J* = 8.2 Hz, 2H), 7.86 (dt, *J* = 7.9, 2.7 Hz, 1H), 7.57 – 7.38 (m, 7H), 7.37 – 7.23 (m, 3H), 7.21 (dd, *J* = 7.4, 2.8 Hz, 1H), 5.82 – 5.75 (m, 1H), 4.04 – 4.00 (m, 1H), 2.80 – 2.68 (m, 1H), 1.89 – 1.79 (m, 1H), 1.57 – 1.52 (m, 1H), 1.47 – 1.41 (m, 1H), 1.21 – 1.16 (m, 3H), 1.04 – 0.95 (m, 6H).

^{13}C NMR (126 MHz, $CDCl_3$) δ 201.6, 201.4, 152.2, 150.94, 150.92, 141.1, 140.9, 139.84, 139.77, 138.4, 138.3, 131.8, 131.3, 130.3, 129.2, 129.1, 128.7, 128.6, 128.00, 127.98, 126.7, 126.6, 126.3, 126.24, 126.21, 125.78, 125.76, 125.74, 125.4, 122.7, 122.6, 65.5, 51.5, 51.1, 46.7, 46.5, 45.8, 45.6, 33.9, 33.8, 28.5, 28.4, 28.3, 28.2, 26.9, 26.06, 26.04.

IR (KBr) ν 3424, 2959, 2926, 1694, 1455, 1366, 1219, 976, 758, 738, 708.

HRMS (ESI) m/z: Calc. For $C_{34}H_{33}NO_2Na$ ($[M+ Na]^+$) 510.2404, Found 510.2399.

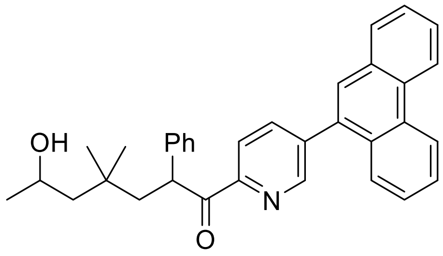

(why-09-41-6)

6-hydroxy-4,4-dimethyl-1-(5-(phenanthren-9-yl)pyridin-2-yl)-2-phenylheptan-1-one (6n)

124.2 mg, 85% yield, d.r. = 1:1, Colorless oil, R_f = 0.5 (petroleum ether/ethyl acetate = 3:1).

^1H NMR (500 MHz, $CDCl_3$) δ 8.90 – 8.86 (m, 1H), 8.78 (d, J = 8.4 Hz, 1H), 8.72 (d, J = 8.2 Hz, 1H), 8.15 (d, J = 7.9 Hz, 1H), 7.93 (dt, J = 7.9, 2.4 Hz, 1H), 7.89 (dd, J = 7.9, 1.5 Hz, 1H), 7.76 (d, J = 8.2 Hz, 1H), 7.73 – 7.66 (m, 3H), 7.66 – 7.60 (m, 1H), 7.55 (t, J = 7.6 Hz, 1H), 7.52 – 7.46 (m, 2H), 7.31 – 7.23 (m, 2H), 7.21 – 7.14 (m, 1H), 5.80 – 5.73 (m, 1H), 4.06 – 3.96 (m, 1H), 2.78 – 2.67 (m, 1H), 1.85 – 1.76 (m, 1H), 1.57 – 1.49 (m, 1H), 1.46 – 1.38 (m, 1H), 1.20 – 1.16 (m, 3H), 1.03 – 0.93 (m, 6H).

^{13}C NMR (126 MHz, $CDCl_3$) δ 201.5, 201.2, 151.9, 149.69, 149.67, 141.1, 140.9, 139.9, 138.3, 138.2, 134.3, 134.2, 131.2, 130.8, 130.4, 130.3, 129.1, 129.0, 128.9, 128.6, 128.5, 127.4, 127.2, 127.0, 126.62, 126.59, 126.1, 123.2, 122.62, 122.55, 122.5, 65.5, 65.4, 51.5, 51.1, 46.5, 46.4, 45.7, 45.4, 33.9, 33.8, 28.5, 28.30, 28.25, 28.2, 26.9, 26.1, 26.0.

IR (KBr) ν 3447, 2959, 2926, 1693, 1470, 1364, 1217, 749, 727, 705.6

HRMS (ESI) m/z: Calc. For $C_{34}H_{33}NO_2Na$ ($[M+ Na]^+$) 510.2404, Found 510.2399.

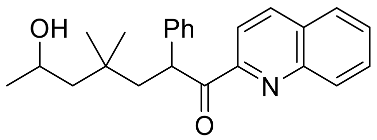

(why-09-49-5)

6-hydroxy-4,4-dimethyl-2-phenyl-1-(quinolin-2-yl)heptan-1-one (6o)

75.8 mg, 70% yield, d.r. = 1:1, Light yellow oil, R_f = 0.3 (petroleum ether/ethyl acetate = 4:1).

^{1}H NMR (500 MHz, $CDCl_3$) δ 8.28 (d, *J* = 8.4 Hz, 1H), 8.21 – 8.16 (m, 1H), 8.10 – 8.04 (m, 1H), 7.84 – 7.75 (m, 2H), 7.65 – 7.58 (m, 1H), 7.48 (m, 2H), 7.27 – 7.18 (m, 2H), 7.12 (dt, *J* = 7.3, 2.0 Hz, 1H), 5.99 – 5.91 (m, 1H), 4.03 – 3.96 (m, 1H), 2.75 – 2.65 (m, 1H), 1.87 – 1.79 (m, 1H), 1.55 – 1.45 (m, 1H), 1.45 – 1.38 (m, 1H), 1.18 – 1.14 (m, 3H), 1.01 – 0.91 (m, 6H).

^{13}C NMR (126 MHz, $CDCl_3$) δ 201.9, 201.6, 152.50, 152.47, 147.2, 147.1, 141.2, 141.0, 136.9, 136.8, 130.9, 130.8, 129.9, 129.8, 129.60, 129.57, 129.1, 129.0, 128.54, 128.52, 128.49, 128.45, 127.6, 127.5, 126.54, 126.51, 119.12, 119.08, 65.5, 65.4, 51.5, 51.2, 46.2, 46.1, 45.5, 45.3, 33.9, 33.8, 28.4, 28.29, 28.26, 28.1, 26.1, 26.0.

IR (KBr) ν 3442, 2959, 2928, 2867, 1692, 1455, 1339, 1114, 830, 756, 729, 698.

HRMS (ESI) m/z: Calc. For $C_{24}H_{27}NO_2Na$ ($[M+ Na]^+$) 384.1934, Found 384.1931.

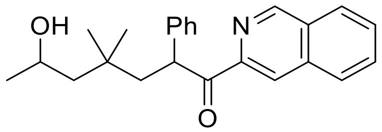

(why-09-49-6)

6-hydroxy-1-(isoquinolin-3-yl)-4,4-dimethyl-2-phenylheptan-1-one (6p)

56.3 mg, 52% yield, d.r. = 1:1, Light yellow oil, R_f = 0.2 (petroleum ether/ethyl acetate = 4:1).

^{1}H NMR (500 MHz, $CDCl_3$) δ 9.31 – 9.27 (m, 1H), 8.46 – 8.41 (m, 1H), 8.02 – 7.96 (m, 1H), 7.91 (dt, *J* = 7.9, 1.4 Hz, 1H), 7.69 (m, 2H), 7.49 – 7.43 (m, 2H), 7.24 – 7.19 (m, 2H), 7.15 – 7.09 (m, 1H), 5.84 – 5.77 (m, 1H), 4.04 – 3.95 (m, 1H), 2.79 – 2.68 (m,

1H), 1.86 – 1.66 (m, 1H), 1.57 – 1.46 (m, 1H), 1.43 – 1.37 (m, 1H), 1.18 – 1.14 (m, 3H), 1.00 – 0.89 (m, 6H).

^{13}C NMR (126 MHz, $CDCl_3$) δ 202.0, 201.7, 151.72, 151.70, 147.20, 147.16, 141.3, 141.1, 135.7, 130.8, 130.7, 130.1, 129.33, 129.26, 129.04, 128.98, 128.52, 128.50, 128.47, 128.46, 127.5, 127.4, 126.50, 126.47, 121.6, 121.5, 65.43, 65.42, 51.5, 51.1, 46.9, 46.7, 45.6, 45.3, 33.84, 33.76, 28.6, 28.33, 28.25, 28.2, 26.02, 25.98.

IR (KBr) ν 3425, 2959, 2927, 1689, 1493, 1451, 1387, 1367, 1162, 1125, 754, 732, 698.

HRMS (ESI) m/z: Calc. For $C_{24}H_{27}NO_2Na$ ([M+ Na]$^+$) 384.1934, Found 384.1931.

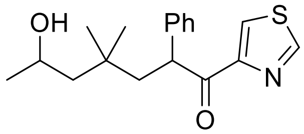

(why-09-49-7)

6-hydroxy-4,4-dimethyl-2-phenyl-1-(thiazol-4-yl)heptan-1-one (6q)

46.6 mg, 49% yield, d.r. = 1:1, Colorless oil, R_f = 0.1 (petroleum ether/ethyl acetate = 4:1).

^{1}H NMR (500 MHz, $CDCl_3$) δ 8.80 (s, 1H), 8.18 (s, 1H), 7.43 – 7.37 (m, 2H), 7.25 (m, 2H), 7.19 – 7.13 (m, 1H), 5.28 – 5.22 (m, 1H), 3.99 – 3.94 (m, 1H), 2.73 – 2.61 (m, 1H), 1.77 – 1.68 (m, 1H), 1.67 – 1.56 (m, 1H), 1.51 – 1.42 (m, 1H), 1.18 – 1.12 (m, 3H), 0.97 – 0.88 (m, 6H).

^{13}C NMR (126 MHz, $CDCl_3$) δ 195.3, 195.0, 155.63, 155.59, 152.5, 152.4, 140.7, 140.6, 128.84, 128.79, 128.6, 126.8, 126.7, 126.1, 125.9, 65.40, 65.36, 51.4, 51.1, 50.2, 50.1, 45.4, 45.2, 33.70, 33.65, 28.4, 28.2, 28.14, 28.11, 26.0.

IR (KBr) ν 3426, 2959, 2927, 1684, 1474, 1149, 878, 731, 699.

HRMS (ESI) m/z: Calc. For $C_{18}H_{23}NO_2SNa$ ([M+ Na]$^+$) 340.1342, Found 340.1340.

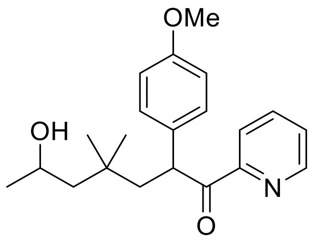

(why-09-37-2)

6-hydroxy-2-(4-methoxyphenyl)-4,4-dimethyl-1-(pyridin-2-yl)heptan-1-one (6r)

79.8 mg, 78% yield, d.r. = 1:1, Colorless oil, R_f = 0.1 (petroleum ether/ethyl acetate = 4:1).

^{1}H NMR (500 MHz, $CDCl_3$) δ 8.69 (d, J = 4.7 Hz, 1H), 7.98 (d, J = 7.8 Hz, 1H), 7.78 – 7.72 (m, 1H), 7.42 – 7.37 (m, 1H), 7.31 (dd, J = 8.8, 2.1 Hz, 2H), 6.77 (dd, J = 8.7, 3.4 Hz, 2H), 5.64 – 5.57 (m, 1H), 4.01 – 3.92 (m, 1H), 3.74 – 3.70 (m, 3H), 2.67 – 2.57 (m, 1H), 1.76 – 1.67 (m, 1H), 1.52 – 1.43 (m, 1H), 1.39 – 1.34 (m, 1H), 1.17 – 1.13 (m, 3H), 0.96 – 0.84 (m, 6H).

^{13}C NMR (126 MHz, CDCl3) δ 201.8, 201.5, 158.38, 158.35, 153.1, 148.9, 148.8, 136.84, 136.75, 132.9, 132.7, 130.0, 129.9, 126.9, 126.8, 123.0, 122.9, 114.0, 65.4, 55.2, 51.4, 51.1, 45.41, 45.39, 45.18, 45.16, 33.74, 33.66, 28.5, 28.3, 28.19, 28.15, 26.02, 25.97.

IR (KBr) ν 3422, 2960, 2925, 1695, 1510, 1367, 995, 755.

HRMS (ESI) m/z: Calc. For $C_{21}H_{27}NO_3Na$ ($[M+ Na]^+$) 364.1883, Found 364.1880.

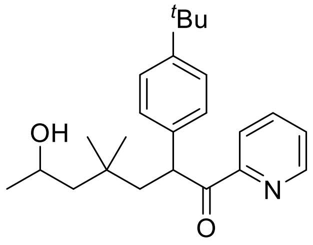

(why-09-37-1)

2-(4-(tert-butyl)phenyl)-6-hydroxy-4,4-dimethyl-1-(pyridin-2-yl)heptan-1-one (6s)

76.1 mg, 65% yield, d.r. = 1:1, Colorless oil, R_f = 0.5 (DCM/MeOH, 50:1).

^{1}H NMR (500 MHz, $CDCl_3$) δ 8.71 (dd, *J* = 4.8, 2.4 Hz, 1H), 8.02 – 7.97 (m, 1H), 7.78 – 7.72 (m, 1H), 7.43 – 7.38 (m, 1H), 7.32 (dd, *J* = 8.4, 3.1 Hz, 2H), 7.28 – 7.21 (m, 2H), 5.71 – 5.62 (m, 1H), 3.99 – 3.93 (m, 1H), 2.68 – 2.59 (m, 1H), 1.78 – 1.69 (m, 1H), 1.49 – 1.41 (m, 1H), 1.39 – 1.34 (m, 1H), 1.24 (s, 9H), 1.15 – 1.11 (m, 3H), 0.97 – 0.86 (m, 6H).

^{13}C NMR (126 MHz, $CDCl_3$) δ 202.0, 201.7, 153.1, 149.4, 149.3, 148.88, 148.86, 137.8, 137.6, 136.8, 136.7, 128.49, 128.45, 126.9, 126.8, 125.42, 125.41, 123.0, 122.9, 65.42,

65.41, 51.4, 51.0, 45.64, 45.58, 45.4, 34.3, 33.8, 33.7, 31.3, 28.5, 28.3, 28.2, 28.1, 26.0, 25.9.

IR (KBr) ν 3421, 2961, 2871, 1696, 1508, 1459, 1365, 572.

HRMS (ESI) m/z: Calc. For $C_{24}H_{33}NO_2Na$ ($[M+ Na]^+$) 390.2404, Found 390.2403.

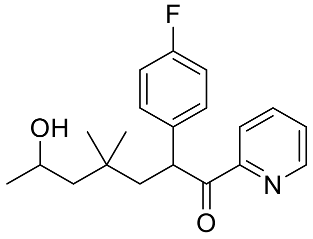

(why-09-37-6)

2-(4-fluorophenyl)-6-hydroxy-4,4-dimethyl-1-(pyridin-2-yl)heptan-1-one (6t)

81.9 mg, 83% yield, d.r. = 1:1, Colorless oil, R_f = 0.2 (petroleum ether/ethyl acetate = 4:1).

^{1}H NMR (500 MHz, $CDCl_3$) δ 8.72 – 8.67 (m, 1H), 8.01 – 7.96 (m, 1H), 7.79 – 7.73 (m, 1H), 7.41 (dd, J = 7.6, 3.7 Hz, 1H), 7.37 (dd, J = 8.8, 5.4 Hz, 2H), 6.91 (td, J = 8.7, 5.6 Hz, 2H), 5.70 – 5.62 (m, 1H), 4.01 – 3.92 (m, 1H), 2.65 – 2.57 (m, 1H), 1.76 – 1.69 (m, 1H), 1.50 – 1.41 (m, 1H), 1.39 – 1.32 (m, 1H), 1.18 – 1.13 (m, 3H), 0.96 – 0.84 (m, 6H).

^{13}C NMR (126 MHz, $CDCl_3$) δ 201.6, 201.4, 161.7 (d, J_{CF} = 241 Hz), 152.7, 148.88, 148.85, 136.9, 136.8, 130.4 (d, J_{CF} = 9 Hz), 130.3 (d, J_{CF} = 9 Hz), 127.03, 126.96, 123.0, 122.9, 121.7, 115.3 (d, J_{CF} = 20 Hz), 65.4, 51.3, 51.0, 45.49, 45.45, 45.3, 45.2, 33.8, 33.7, 28.5, 28.2, 28.14, 28.10, 26.0.

IR (KBr) ν 3424, 2960, 2927, 1696, 1507, 1223, 1158, 757.

HRMS (ESI) m/z: Calc. For $C_{20}H_{24}FNO_2Na$ ($[M+ Na]^+$) 352.1683, Found 352.1681.

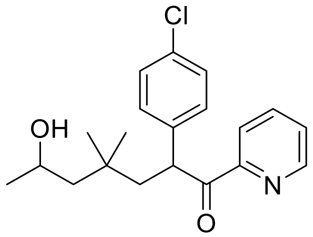

(why-09-47-2)

2-(4-chlorophenyl)-6-hydroxy-4,4-dimethyl-1-(pyridin-2-yl)heptan-1-one (6u)

75.6 mg, 73% yield, d.r. = 1:1, Colorless oil, R_f = 0.2 (petroleum ether/ethyl acetate = 4:1).

^{1}H NMR (500 MHz, $CDCl_3$) δ 8.71 – 8.67 (m, 1H), 8.01 – 7.95 (m, 1H), 7.79 – 7.74 (m, 1H), 7.44 – 7.39 (m, 1H), 7.37 – 7.31 (m, 2H), 7.22 – 7.16 (m, 2H), 5.68 – 5.61 (m, 1H), 4.01 – 3.91 (m, 1H), 2.67 – 2.57 (m, 1H), 1.75 – 1.67 (m, 1H), 1.52 – 1.43 (m, 1H), 1.38 – 1.32 (m, 1H), 1.17 – 1.14 (m, 3H), 0.95 – 0.85 (m, 6H).

^{13}C NMR (126 MHz, $CDCl_3$) δ 201.3, 201.1, 152.73, 152.69, 148.91, 148.89, 139.6, 139.4, 136.9, 136.8, 132.5, 132.4, 130.4, 130.30, 130.28, 128.7, 128.6, 127.1, 127.0, 123.0, 122.9, 65.4, 51.3, 51.1, 45.7, 45.5, 45.4, 45.2, 33.8, 33.7, 28.4, 28.19, 28.16, 28.1, 26.1.

IR (KBr) ν 3381, 2959, 2925, 1695, 1488, 1091, 995, 744.

HRMS (ESI) m/z: Calc. For $C_{20}H_{24}ClNO_2Na$ ([M+ Na]$^+$) 368.1388, Found 368.1387.

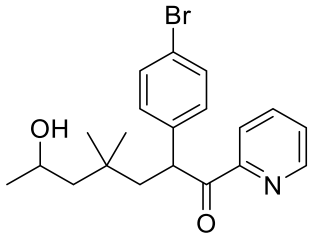

(why-09-37-8)

2-(4-bromophenyl)-6-hydroxy-4,4-dimethyl-1-(pyridin-2-yl)heptan-1-one (6v)

66.5 mg, 57% yield, d.r. = 1:1, Colorless oil, R_f = 0.2 (petroleum ether/ethyl acetate = 4:1).

^{1}H NMR (500 MHz, $CDCl_3$) δ 8.71 – 8.67 (m, 1H), 7.98 (d, *J* = 7.8 Hz, 1H), 7.80 – 7.74 (m, 1H), 7.45 – 7.40 (m, 1H), 7.35 (dd, *J* = 8.5, 5.5 Hz, 2H), 7.31 – 7.24 (m, 2H), 5.68 – 5.59 (m, 1H), 4.01 – 3.92 (m, 1H), 2.66 – 2.57 (m, 1H), 1.75 – 1.67 (m, 1H), 1.52 – 1.43 (m, 1H), 1.38 – 1.32 (m, 1H), 1.17 – 1.14 (m, 3H), 0.95 – 0.84 (m, 6H).

^{13}C NMR (126 MHz, $CDCl_3$) δ 201.2, 201.1, 152.7, 148.91, 148.89, 140.1, 139.9, 137.0, 136.9, 131.6, 130.74, 130.67, 127.11, 127.05, 123.0, 122.9, 120.61, 120.55, 65.4, 51.3, 51.0, 45.8, 45.6, 45.3, 45.1, 33.8, 33.7, 28.5, 28.19, 28.17, 28.1, 26.08, 26.07.

IR (KBr) ν 3382, 2959, 2926, 1695, 1485, 1010, 995, 743.

HRMS (ESI) m/z: Calc. For $C_{20}H_{24}BrNO_2Na$ ([M+ Na]$^+$) 412.0883, Found 412.0880.

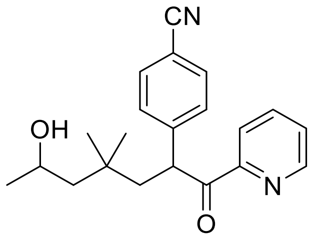

(why-09-33-1)

4-(6-hydroxy-4,4-dimethyl-1-oxo-1-(pyridin-2-yl)heptan-2-yl)benzonitrile (6w)

71.6 mg, 71% yield, d.r. = 1:1, Light yellow oil, R_f = 0.2 (petroleum ether/ethyl acetate = 2:1).

^{1}H NMR (500 MHz, $CDCl_3$) δ 8.72 – 8.68 (m, 1H), 8.00 (d, *J* = 7.9 Hz, 1H), 7.83 – 7.76 (m, 1H), 7.57 – 7.49 (m, 4H), 7.49 – 7.42 (m, 1H), 5.79 – 5.70 (m, 1H), 4.01 – 3.91 (m, 1H), 2.68 – 2.60 (m, 1H), 1.77 – 1.69 (m, 1H), 1.53 – 1.44 (m, 1H), 1.37 – 1.30 (m, 1H), 1.18 – 1.14 (m, 3H), 0.95 – 0.83 (m, 6H).

^{13}C NMR (126 MHz, $CDCl_3$) δ 200.6, 200.5, 152.4, 152.3, 149.00, 148.97, 146.7, 146.4, 137.1, 137.0, 132.24, 132.22, 129.8, 129.7, 127.4, 127.3, 123.1, 123.0, 118.8, 110.5, 110.4, 65.3, 51.1, 51.0, 46.6, 46.3, 45.3, 45.2, 33.9, 33.8, 28.4, 28.2, 28.1, 26.2, 26.1.

IR (KBr) ν 3427, 2960, 2926, 2228, 1697, 1308, 1285, 995, 751, 564.

HRMS (ESI) m/z: Calc. For $C_{21}H_{25}N_2O_2$ ([M+ H]$^+$) 337.1911, Found 337.1911.

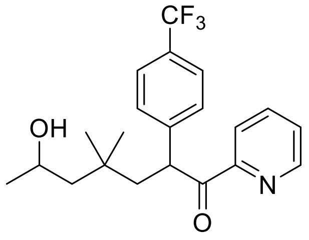

(why-09-38-2)

6-hydroxy-4,4-dimethyl-1-(pyridin-2-yl)-2-(4-(trifluoromethyl)phenyl)heptan-1-one (6x)

97.8 mg, 86% yield, d.r. = 1:1, Colorless oil, R_f = 0.5 (petroleum ether/ethyl acetate = 3:1).

^1H NMR (500 MHz, $CDCl_3$) δ 8.67 (d, *J* = 4.7, 1H), 7.96 (d, *J* = 7.8 Hz, 1H), 7.76 – 7.70 (m, 1H), 7.53 – 7.42 (m, 4H), 7.41 – 7.36 (m, 1H), 5.77 – 5.68 (m, 1H), 3.98 – 3.88 (m, 1H), 2.67 – 2.59 (m, 1H), 1.74 – 1.69 (m, 1H), 1.50 – 1.41 (m, 1H), 1.35 – 1.28 (m, 1H), 1.14 – 1.10 (m, 3H), 0.93 – 0.81 (m, 6H).

^{13}C NMR (126 MHz, $CDCl_3$) δ 201.0, 200.9, 152.6, 152.5, 148.93, 148.90, 145.2, 145.0, 137.0, 136.9, 129.3, 129.2, 126.5 (q, J_{CF} = 232 Hz), 125.4, 125.3, 123.0, 122.9, 65.3, 51.2, 51.0, 46.2, 46.0, 45.4, 45.3, 33.9, 33.8, 28.4, 28.14, 28.09, 28.05, 26.1, 26.0, 17.28, 17.25, 17.1.

IR (KBr) ν 3423, 2962, 2929, 1667, 1325, 1165, 1125, 1068.

HRMS (ESI) m/z: Calc. For $C_{21}H_{25}F_3NO_2$ ($[M+H]^+$) 380.1832, Found 380.1828.

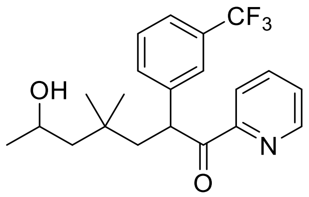

(why-09-38-3)

6-hydroxy-4,4-dimethyl-1-(pyridin-2-yl)-2-(3-(trifluoromethyl)phenyl)heptan-1-one (6y)

103.5 mg, 91% yield, d.r. = 1:1, Colorless oil, R_f = 0.5 (petroleum ether/ethyl acetate = 3:1).

^1H NMR (500 MHz, $CDCl_3$) δ 8.73 – 8.69 (m, 1H), 8.03 – 7.98 (m, 1H), 7.80 – 7.75 (m, 1H), 7.69 (s, 1H), 7.64 – 7.59 (m, 1H), 7.46 – 7.32 (m, 3H), 5.80 – 5.72 (m, 1H), 4.00 – 3.94 (m, 1H), 2.70 – 2.62 (m, 1H), 1.78 – 1.71 (m, 1H), 1.52 – 1.44 (m, 1H), 1.39 – 1.32 (m, 1H), 1.17 – 1.14 (m, 3H), 0.96 – 0.85 (m, 6H).

^{13}C NMR (126 MHz, $CDCl_3$) δ 201.1, 200.9, 152.51, 152.45, 149.0, 148.9, 142.1, 141.9, 137.0, 136.9, 132.4, 132.3, 130.8 (q, J_{CF} = 33 Hz), 128.84, 128.82, 127.22, 127.15, 124.5 (q, J_{CF} = 285 Hz), 123.0, 122.9, 65.3, 51.2, 51.0, 46.1, 45.9, 45.5, 45.3, 33.9, 33.8, 28.4, 28.2, 28.1, 26.10, 26.05.

IR (KBr) ν 3420, 2962, 2929, 1697, 1328, 1165, 1126, 1075.

HRMS (ESI) m/z: Calc. For $C_{21}H_{25}F_3NO_2$ ($[M+H]^+$) 380.1832, Found 380.1827.

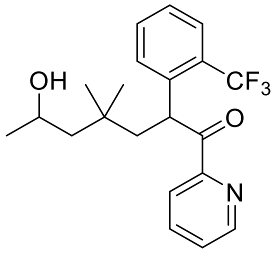

(why-09-38-4)

6-hydroxy-4,4-dimethyl-1-(pyridin-2-yl)-2-(2-(trifluoromethyl)phenyl)heptan-1-one (6z)

90.9 mg, 80% yield, d.r. = 1:1, Colorless oil, R_f = 0.5 (petroleum ether/ethyl acetate = 3:1).

^{1}H NMR (500 MHz, $CDCl_3$) δ 8.69 (d, *J* = 4.7 Hz, 1H), 7.95 (d, *J* = 7.9 Hz, 1H), 7.80 – 7.72 (m, 2H), 7.60 (d, *J* = 8.1 Hz, 1H), 7.53 – 7.40 (m, 2H), 7.28 (d, *J* = 7.8 Hz, 1H), 6.12 – 6.03 (m, 1H), 4.04 – 3.92 (m, 1H), 2.55 – 2.42 (m, 1H), 1.88 – 1.74 (m, 1H), 1.65 – 1.58 (m, 1H), 1.45 – 1.31 (m, 1H), 1.19 – 1.13 (m, 3H), 0.98 – 0.89 (m, 6H).

^{13}C NMR (126 MHz, $CDCl_3$) δ 201.5, 201.4, 152.8, 152.7, 148.9, 148.8, 138.9, 138.6, 136.9, 136.7, 131.6, 131.5, 130.8, 130.6, 129.8, 128.4 (q, J_{CF} = 32 Hz), 127.1, 127.0, 126.6, 126.5, 124.4 (q, J_{CF} = 275 Hz), 122.9, 122.7, 65.4, 65.3, 51.8, 50.6, 46.4, 45.2, 42.2, 42.0, 34.43, 34.37, 28.9, 28.2, 28.0, 26.0, 25.9.

IR (KBr) ν 3384, 2963, 2929, 1697, 1312, 1153, 1124, 1037, 768.

HRMS (ESI) m/z: Calc. For $C_{21}H_{25}F_3NO_2$ ($[M+H]^+$) 380.1832, Found 380.1827.

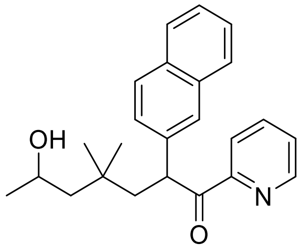

(why-09-38-7)

6-hydroxy-4,4-dimethyl-2-(naphthalen-2-yl)-1-(pyridin-2-yl)heptan-1-one (6aa)

74.7 mg, 69% yield, d.r. = 1:1, Colorless oil, R_f = 0.5 (petroleum ether/ethyl acetate = 3:1).

^{1}H NMR (500 MHz, $CDCl_3$) δ 8.70 (d, J = 4.7 Hz, 1H), 7.98 (d, J = 7.9 Hz, 1H), 7.81 (s, 1H), 7.78 – 7.68 (m, 4H), 7.57 (dd, J = 8.5, 2.0 Hz, 1H), 7.38 (m, 3H), 5.87 – 5.79 (m, 1H), 4.03 – 3.94 (m, 1H), 2.80 – 2.70 (m, 1H), 1.87 – 1.79 (m, 1H), 1.57 – 1.48 (m, 1H), 1.43 – 1.37 (m, 1H), 1.17 – 1.13 (m, 3H), 1.01 – 0.89 (m, 6H).

^{13}C NMR (126 MHz, $CDCl_3$) δ 201.6, 201.3, 153.0, 152.9, 148.87, 148.85, 148.7, 138.4, 138.3, 136.84, 136.75, 133.5, 132.30, 132.27, 128.2, 127.71, 127.66, 127.6, 127.5, 127.19, 127.16, 126.92, 126.86, 125.86, 125.85, 125.6, 125.5, 123.0, 122.9, 65.4, 51.4, 51.1, 46.5, 46.3, 45.5, 45.2, 33.9, 33.8, 28.5, 28.3, 28.2, 26.0.

IR (KBr) ν 3424, 2959, 2926, 1694, 1367, 995, 816, 745, 684, 477.

HRMS (ESI) m/z: Calc. For $C_{24}H_{27}NO_2Na$ ($[M+ Na]^+$) 384.1934, Found 384.1928.

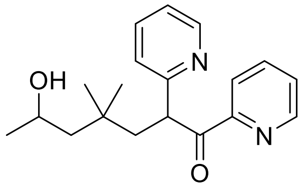

(why-09-38-6)

6-hydroxy-4,4-dimethyl-1,2-di(pyridin-2-yl)heptan-1-one (6ab)

63.6 mg, 68% yield, d.r. = 1:1, Colorless oil, R_f = 0.2 (petroleum ether/ethyl acetate = 3:1).

^{1}H NMR (500 MHz, $CDCl_3$) δ 8.72 – 8.65 (m, 1H), 8.53 – 8.47 (m, 1H), 8.09 – 8.02 (m, 1H), 7.82 – 7.75 (m, 1H), 7.59 – 7.50 (m, 1H), 7.47 – 7.38 (m, 2H), 7.09 – 7.03 (m, 1H), 5.89 – 5.80 (m, 1H), 4.03 – 3.96 (m, 1H), 2.63 – 2.48 (m, 1H), 2.09 – 1.91 (m, 1H), 1.68 – 1.52 (m, 1H), 1.42 – 1.33 (m, 1H), 1.20 – 1.14 (m, 3H), 0.96 – 0.83 (m, 6H).

^{13}C NMR (126 MHz, $CDCl_3$) δ 200.9, 200.7, 160.5, 160.4, 153.0, 149.4, 149.3, 149.0, 148.9, 136.9, 136.8, 136.6, 136.3, 126.9, 123.8, 123.0, 122.9, 122.8, 121.4, 65.3, 64.9, 50.9, 50.7, 49.6, 49.1, 44.0, 43.5, 33.8, 33.7, 28.6, 28.5, 28.3, 28.1, 25.99, 25.98.

IR (KBr) ν 3380, 2959, 2927, 1699, 1586, 1470, 1434, 1308, 995, 748.

HRMS (ESI) m/z: Calc. For $C_{19}H_{24}N_2O_2Na$ ([M+ Na]$^+$) 335.1730, Found 335.1724.

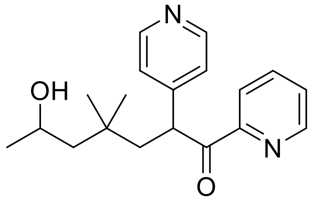

(why-09-47-4)

6-hydroxy-4,4-dimethyl-1-(pyridin-2-yl)-2-(pyridin-4-yl)heptan-1-one (6ac)

51.5 mg, 55% yield, d.r. = 1:1, Colorless oil, R_f = 0.4 (petroleum ether/ethyl acetate = 4:1).

^{1}H NMR (500 MHz, $CDCl_3$) δ 8.73 – 8.69 (m, 1H), 8.47 – 8.42 (m, 2H), 8.03 – 7.98 (m, 1H), 7.83 – 7.76 (m, 1H), 7.49 – 7.42 (m, 1H), 7.36 – 7.32 (m, 2H), 5.72 – 5.64 (m, 1H), 3.99 – 3.94 (m, 1H), 2.68 – 2.59 (m, 1H), 1.78 – 1.67 (m, 1H), 1.53 – 1.45 (m, 1H), 1.32 – 1.37 (m, 1H), 1.18 – 1.15 (m, 3H), 0.97 – 0.84 (m, 6H).

^{13}C NMR (126 MHz, $CDCl_3$) δ 200.5, 200.4, 152.4, 152.3, 150.1, 149.9, 149.83, 149.80, 148.99, 148.96, 137.1, 137.0, 127.4, 127.3, 124.2, 124.1, 123.0, 122.9, 65.29, 65.27, 51.1, 51.0, 46.0, 45.8, 45.1, 44.9, 33.9, 33.8, 28.4, 28.2, 28.1, 26.2, 26.1.

IR (KBr) ν 3365, 2959, 2926, 1698, 1596, 1417, 995, 794.

HRMS (ESI) m/z: Calc. For $C_{19}H_{25}N_2O_2$ ([M+ H]$^+$) 313.1911, Found 313.1910.

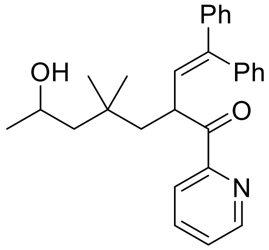

(why-09-37-7)

2-(2,2-diphenylvinyl)-6-hydroxy-4,4-dimethyl-1-(pyridin-2-yl)heptan-1-one (6ad)

121.4 mg, 98% yield, d.r. = 1:1, Colorless oil, R_f = 0.2 (petroleum ether/ethyl acetate = 4:1).

^{1}H NMR (500 MHz, $CDCl_3$) δ 8.54 – 8.48 (m, 1H), 8.02 – 7.94 (m, 1H), 7.83 – 7.77 (m, 1H), 7.45 – 7.37 (m, 1H), 7.34 – 7.26 (m, 3H), 7.24 – 7.15 (m, 5H), 7.13 – 7.07 (m,

1H), 7.07 – 7.02 (m, 1H), 6.20 – 6.09 (m, 1H), 5.13 – 5.00 (m, 1H), 3.90 – 3.77 (m, 1H), 2.28 – 2.16 (m, 1H), 1.66 – 1.60 (m, 1H), 1.22 – 1.16 (m, 2H), 1.07 – 1.02 (m, 3H), 0.89 – 0.77 (m, 6H).

^{13}C NMR (126 MHz, $CDCl_3$) δ 203.09, 203.07, 153.0, 152.8, 148.73, 148.69, 142.8, 142.7, 142.1, 142.0, 139.7, 137.0, 136.7, 130.0, 129.8, 129.6, 129.4, 128.1, 127.23, 127.21, 126.9, 126.8, 122.8, 122.7, 65.4, 65.2, 51.3, 50.5, 45.6, 45.0, 42.94, 42.85, 33.9, 33.8, 29.1, 28.5, 28.4, 28.2, 25.9, 25.8.

IR (KBr) ν 3427, 2959, 2925, 1692, 1444, 1367, 765, 701.

HRMS (ESI) m/z: Calc. For $C_{28}H_{31}NO_2Na$ ([M+ Na]$^+$) 436.2247, Found 436.2244.

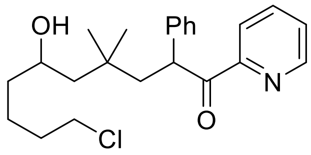

(why-09-57-5)

10-chloro-6-hydroxy-4,4-dimethyl-2-phenyl-1-(pyridin-2-yl)decan-1-one (6ae)

67.3 mg, 58% yield, d.r. = 1:1, Colorless oil, R_f = 0.2 (petroleum ether/ethyl acetate = 4:1).

^{1}H NMR (500 MHz, $CDCl_3$) δ 8.71 – 8.68 (m, 1H), 8.01 – 7.95 (m, 1H), 7.78 – 7.72 (m, 1H), 7.42 – 7.38 (m, 3H), 7.25 – 7.19 (m, 2H), 7.17 – 7.10 (m, 1H), 5.71 – 5.63 (m, 1H), 3.79 – 3.72 (m, 1H), 3.54 – 3.49 (m, 2H), 2.71 – 2.61 (m, 1H), 1.92 – 1.82 (m, 1H), 1.79 – 1.73 (m, 2H), 1.55 – 1.34 (m, 6H), 0.98 – 0.87 (m, 6H).

^{13}C NMR (126 MHz, $CDCl_3$) δ 201.8, 201.5, 152.9, 148.9, 148.8, 147.8, 140.9, 140.7, 136.9, 136.8, 129.0, 128.9, 128.5, 127.0, 126.9, 126.6, 126.5, 123.0, 122.9, 121.7, 68.9, 68.8, 49.7, 46.4, 46.1, 45.5, 44.9, 38.8, 38.7, 33.8, 33.7, 32.61, 32.57, 28.5, 28.4, 28.22, 28.17, 23.0, 22.9.

IR (KBr) ν 3371, 2953, 2865, 1696, 1463, 994, 885, 745, 702.

HRMS (ESI) m/z: Calc. For $C_{23}H_{31}ClNO_2$ ([M+ H]$^+$) 388.2043, Found 388.2047.

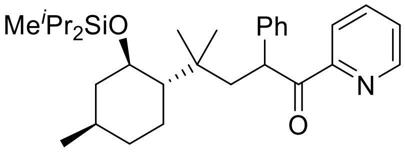

(why-09-57-8)

4-((1S,2S,5S)-2-((diisopropyl(methyl)silyl)oxy)-5-methylcyclohexyl)-4-methyl-2-phenyl-1-(pyridin-2-yl)pentan-1-one (6af')

56.2 mg, 38% yield, d.r. = 5:1, Colorless oil, R_f = 0.5 (petroleum ether/ethyl acetate = 10:1).

^{1}H NMR (500 MHz, $CDCl_3$) δ 8.70 (d, *J* = 5.0 Hz, 1H), 7.99 (d, *J* = 7.8 Hz, 1H), 7.73 (td, *J* = 7.6, 1.9 Hz, 1H), 7.44 – 7.35 (m, 3H), 7.22 (td, *J* = 7.6, 2.3 Hz, 2H), 7.13 (td, *J* = 7.3, 1.8 Hz, 1H), 5.84 – 8.74 (m, 1H), 3.56 – 3.38 (m, 1H), 2.85 – 2.76 (m, 1H), 2.38 – 2.22 (m, 1H), 1.93 – 1.77 (m, 2H), 1.65 – 1.53 (m, 2H), 1.38 – 1.30 (m, 1H), 1.09 – 1.03 (m, 6H), 1.02 – 0.96 (m, 4H), 0.92 – 0.80 (m, 12H), 0.73 (dd, *J* = 7.0, 4.3 Hz, 1H), 0.67 (t, *J* = 6.6 Hz, 2H), 0.11 – 0.09 (m, 3H).

^{13}C NMR (126 MHz, $CDCl_3$) δ 201.6, 201.5, 153.1, 148.82, 148.80, 141.6, 136.6, 128.94, 128.93, 128.38, 128.35, 126.6, 126.24, 126.21, 122.9, 73.00, 72.98, 72.78, 72.77, 50.7, 50.64, 50.61, 45.8, 45.70, 45.66, 45.0, 41.6, 41.5, 41.4, 41.3, 34.60, 34.59, 31.72, 31.70, 31.69, 24.9, 24.8, 23.9, 23.8, 23.79, 23.77, 23.7, 23.3, 23.2, 22.73, 22.69, 22.38, 22.36, 21.5, 21.41, 21.39, 19.0, 18.9, 18.83, 18.79, 15.9, 15.8, 15.7, 14.38, 14.36, 14.34, 14.32, -6.5, -7.1.

IR (KBr) ν 2953, 2927, 2866, 1696, 1457, 1252, 1107, 1083, 1067, 996,779, 755, 702.

HRMS (ESI) m/z: Calc. For $C_{31}H_{47}NO_2SiNa$ ($[M+ Na]^+$) 516.3268, Found 516.3268.

2.4 Gram-scale synthesis and chemical transformation of products

2.4.1 Gram-scale reaction

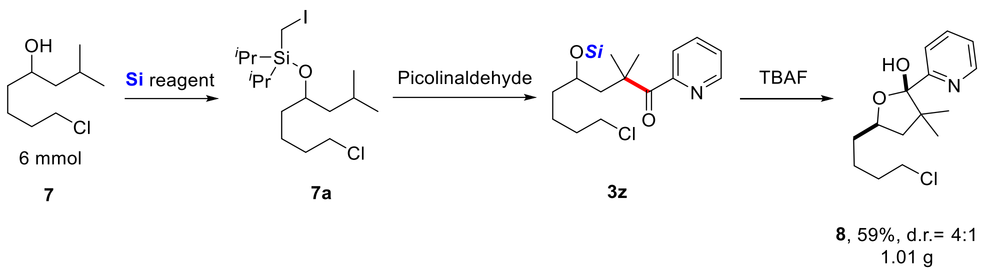

To a stirred solution of **7** (6 mmol) and imidazole (12 mmol) in THF, chloro(iodomethyl)diisopropylsilane (9 mmol) was added at room temperature under N_2 atmosphere. After being stirred for 12 h, the resulting mixture was purified by column chromatography in petroleum ether to afford corresponding alkyl silyl ether **7a**. A 100 mL vial equipped with a stir bar was charged with **N2** (1.2 mmol), $Pd(OAc)_2$ (0.6 mmol), PCy_3 (1.2 mmol) and 40 mL of $PhCF_3$. After stirring for 30 min in glove box, to the solution was added Cs_2CO_3 (12 mmol), picolinaldehyde **2a** (9 mmol), alkyl silyl ether **7a** (6 mmol), and 20 mL of $PhCF_3$. The reaction mixture was removed from the glove box and stirred under 36W Blue LEDs at room temperature until the complete consumption of **7a** by TLC analysis. Then, TBAF (12 mmol, 1.0 M THF) was added. After being stirred for 2 h, The solution was concentrated under reduced pressure, and purified by column chromatography on silica gel with petroleum ether/ethyl acetate = 6 : 1 to afford the desired hemiketal **8** in 1.01 g, 59% yield total yield.

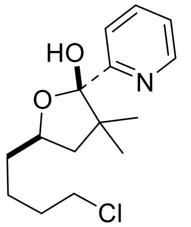

(why-09-81-2)

cis-5-(4-chlorobutyl)-3,3-dimethyl-2-(pyridin-2-yl)tetrahydrofuran-2-ol (8)

1.01 g, 59% yield, d.r. = 4:1, Light yellow oil, R_f = 0.2 (petroleum ether/ethyl acetate = 4:1).

^{1}H NMR (500 MHz, $CDCl_3$) δ 8.56 – 8.50 (m, 1H), 7.68 (td, J = 7.6, 1.8 Hz, 1H), 7.62 (dt, J = 7.9, 1.2 Hz, 1H), 7.29 – 7.20 (m, 1H), 4.98 – 4.90 (m, 1H), 4.43 – 4.29 (m, 1H),

3.60 – 3.51 (m, 2H), 2.05 – 1.95 (m, 1H), 1.95 – 1.79 (m, 3H), 1.69 – 1.60 (m, 2H), 1.45 – 1.37 (m, 1H),1.19 – 1.10 (m, 3H), 1.07 – 0.99 (m, 1H), 0.73 (m, 3H).

^{13}C NMR (126 MHz, $CDCl_3$) δ 159.0, 147.8, 136.0, 123.0, 122.9, 121.7, 106.4, 78.3, 47.4, 45.2, 45.0, 37.1, 32.7, 24.9, 23.6, 22.2.

IR (KBr) ν 3426, 2937, 2867, 1592, 1468, 1435, 1365, 1047, 996, 785, 750.

HRMS (ESI) m/z: Calc. For $C_{15}H_{22}ClNO_2Na$ ($[M+ Na]^+$) 306.1231, Found 306.1230.

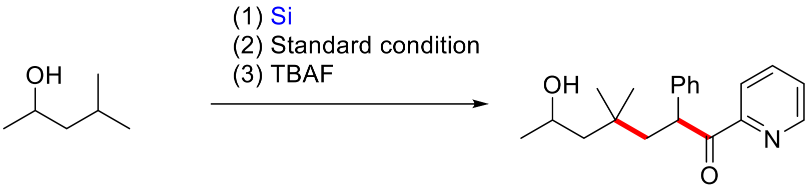

6 mmol

6a, 60 %
1.12g

To a stirred solution of 4-methylpentan-2-ol (6 mmol) and imidazole (12 mmol) in THF, chloro(iodomethyl)diisopropylsilane (9 mmol) was added at room temperature under N_2 atmosphere. After being stirred for 12 h, the resulting mixture was purified by column chromatography in petroleum ether to afford corresponding alkyl silyl ether **1a**. A 100 mL vial equipped with a stir bar was charged with **N2** (1.2 mmol), $Pd(OAc)_2$ (0.6 mmol), PCy_3 (1.2 mmol) and 40 mL of $PhCF_3$. After stirring for 30 min in glove box, to the solution was added Cs_2CO_3 (12 mmol), picolinaldehyde (9 mmol), styrene (12 mmol), corresponding alkyl silyl ether **1a**, and 20 mL of $PhCF_3$. The reaction mixture was removed from the glove box and stirred under 36W Blue LEDs at room temperature until the complete consumption of corresponding alkyl silyl ether **1a** by TLC analysis. Then, TBAF (12 mmol, 1.0 M THF) was added. After being stirred for 2 h, the solution was concentrated under reduced pressure, and purified by column chromatography on silica gel with petroleum ether/ethyl acetate = 6 : 1 to afford the desired ketone **6a** in 1.12 g, 60 % total yield.

2.4.2 Chemical transformations

Reduction

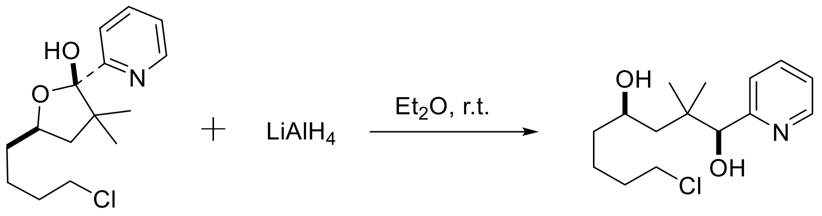

8

9, 56%, dr = 4:1

To a stirred solution of $LiAlH_4$ (1.0 mmol, 2.0 equiv) in THF (3.0 mL) at 0 °C, hemiketal **8** (0.5 mmol, 1.0 equiv) in THF (2.0 mL) was added under N_2 atmosphere. After being stirred for 6 h, the reaction mixture was quenched with saturated Na_2SO_4. The aqueous layer was extracted with two portions of ethyl acetate. The combined extract was dried over $MgSO_4$ and concentrated under reduced pressure. The residue was purified by column chromatography on silica gel with ethyl acetate to give reduction compound **9** in 56% yield.[3]

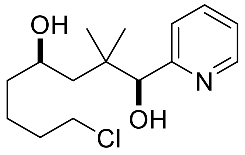

(why-09-87-2)

cis-8-chloro-2,2-dimethyl-1-(pyridin-2-yl)octane-1,4-diol (9)

79.8 mg, 56% yield, d.r. = 4:1, Colorless oil, R_f = 0.5 (ethyl acetate).

^{1}H NMR (500 MHz, $CDCl_3$) δ 8.56 – 8.51 (m, 1H), 8.56 – 8.47 (m, 1H), 7.71 – 7.63 (m, 2H), 7.25 – 7.17 (m, 2H), 5.84 – 5.74 (m, 1H), 4.85 – 4.28 (m, 1H), 3.89 – 3.83 (m, 1H), 3.60 – 3.52 (m, 1H), 1.86 – 1.78 (m, 1H), 1.69 – 1.60 (m, 1H), 1.56 – 1.48 (m, 2H), 1.45 – 1.40 (m, 1H), 1.38 – 1.32 (m, 1H), 1.27 – 1.18 (m, 4H), 1.02 – 0.99 (m, 1H), 0.83 – 0.74 (m, 3H).

^{13}C NMR (126 MHz, $CDCl_3$) δ 161.0, 160.8, 147.9, 147.2, 136.5, 136.4, 136.2, 124.2, 124.1, 122.9, 122.5, 122.4, 81.0, 68.1, 67.7, 45.9, 45.0, 39.4, 38.9, 38.3, 32.7, 27.9, 25.0, 24.9, 23.1, 22.8, 22.2, 14.1.

IR (KBr) ν 3318, 2957, 2930, 2870, 1595, 1471, 1436, 1056, 754.

HRMS (ESI) m/z: Calc. For $C_{15}H_{24}ClNO_2Na$ ($[M+Na]^+$) 308.1388, Found 308.1387.

Substitution

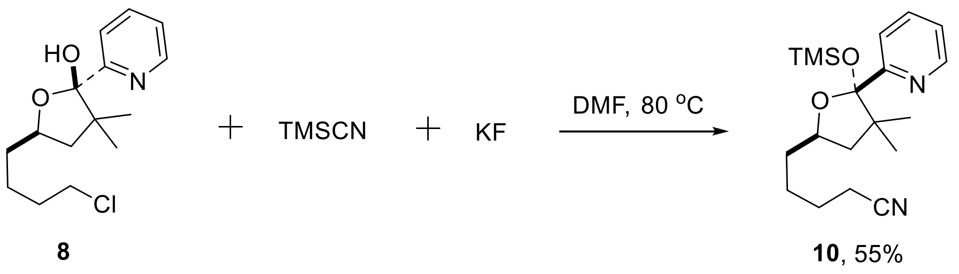

To a stirred solution of hemiketal **8** (0.2 mmol, 1.0 equiv) and KF (0.6 mmol, 3.0 equiv) in DMF (2.0 mL), TMSCN (0.6 mmol, 3.0 equiv) was added at 80°C under N_2 atmosphere. After being stirred for 14 h, the reaction mixture was poured into aq. $NaHCO_3$ (sat) with ethyl acetate. The aqueous layer was extracted with two portions of EA. The combined extract was washed with brine, dried over $MgSO_4$ and concentrated under reduced pressure. The residue was purified by column chromatography on silica gel with petroleum ether/ethyl acetate = 10 : 1 to give **10** in 55% yield.[4]

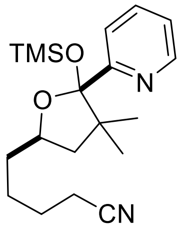

(why-09-90)

5-(trans-4,4-dimethyl-5-(pyridin-2-yl)-5-((trimethylsilyl)oxy)tetrahydrofuran-2-yl)pentanenitrile (10)

38.1 mg, 55% yield, d.r. > 20:1, Colorless oil, R_f = 0.7 (petroleum ether/ethyl acetate = 4:1).

^{1}H NMR (500 MHz, $CDCl_3$) δ 8.59 (dd, *J* = 4.9, 1.9, 1H), 7.65 – 7.58 (m, 1H), 7.56 (d, *J* = 7.9, 1H), 7.17 – 7.13 (m, 1H), 4.26 – 4.19 (m, 1H), 2.37 (t, *J* = 7.1 Hz, 2H), 1.94 – 1.88 (m, 1H), 1.87 – 1.77 (m, 2H), 1.77 – 1.70 (m, 2H), 1.67 – 1.52 (m, 3H), 1.27 (s, 3H), 0.54 (s, 3H), -0.13 (s, 9H).

^{13}C NMR (126 MHz, $CDCl_3$) δ 161.0, 148.5, 135.5, 122.3, 121.6, 119.6, 108.3, 77.7, 47.9, 46.1, 36.8, 25.8, 25.5, 24.2, 22.7, 17.2, 1.6.

IR (KBr) ν 2956, 2870, 1588, 1467, 1433, 1248, 1102, 1043, 995, 912, 842, 752.

HRMS (ESI) m/z: Calc. For $C_{19}H_{30}N_2O_2SiNa$ ([M+ Na]$^+$) 369.1969, Found 369.1967.

Oxidation

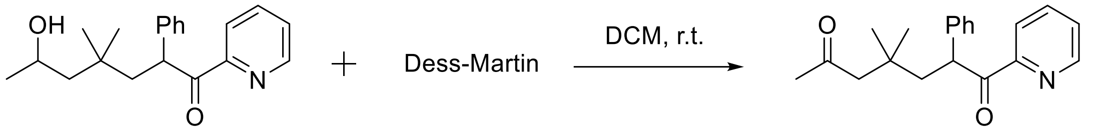

6a **11**, 79%

To a stirred solution of ketones **6a** (2.0 mmol, 1.0 equiv) in CH_2Cl_2 (10 mL), Dess-Martin periodinane (4.0 mmol, 2.0 equiv) was added at rt under N_2 atmosphere. After being stirred for 24 h, the reaction mixture was poured into aq. $NaHCO_3$ (sat) with EA. The aqueous layer was extracted with two portions of EA. The combined extract was washed with brine, dried over $MgSO_4$, and concentrated under reduced pressure. The residue was purified by column chromatography on silica gel with petroleum ether/ethyl acetate = 10 : 1 to give **11** in 79% yield.[5]

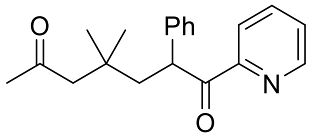

(why-09-105)

4,4-dimethyl-2-phenyl-1-(pyridin-2-yl)heptane-1,6-dione (11)

488.2 mg, 79% yield, Light yellow oil, R_f= 0.5 (petroleum ether/ethyl acetate = 4:1).

^{1}H NMR (400 MHz, $CDCl_3$) δ 8.69 (d, *J* = 5.6 Hz, 1H), 7.98 (d, *J* = 7.9 Hz, 1H), 7.75 (td, *J* = 7.7, 1.8 Hz, 1H), 7.44 – 7.35 (m, 3H), 7.25 – 7.18 (m, 2H), 7.17 – 7.10 (m, 1H), 5.66 (dd, *J* = 8.7, 4.0 Hz, 1H), 2.59 (dd, *J* = 14.1, 8.6 Hz, 1H), 2.35 (s, 2H), 2.04 (s, 3H), 1.89 (dd, *J* = 14.1, 4.0 Hz, 1H), 1.01 (s, 3H), 0.99 (s, 3H).

^{13}C NMR (101 MHz, $CDCl_3$) δ 208.6, 201.2, 152.7, 148.8, 140.4, 136.8, 129.0, 128.9, 128.5, 128.4, 127.0, 126.6, 122.9, 54.0, 46.0, 45.0, 34.2, 32.4, 27.6, 27.4.

IR (KBr) ν 2954, 2867, 1696, 1581, 1454, 1435, 1361, 1215, 994, 742, 701.

HRMS (ESI) m/z: Calc. For $C_{20}H_{23}NO_2Na$ ([M+ Na]$^+$) 332.1621, Found 332.1620.

2.5 Mechanistic studies

2.5.1 Radical trapping experiment:

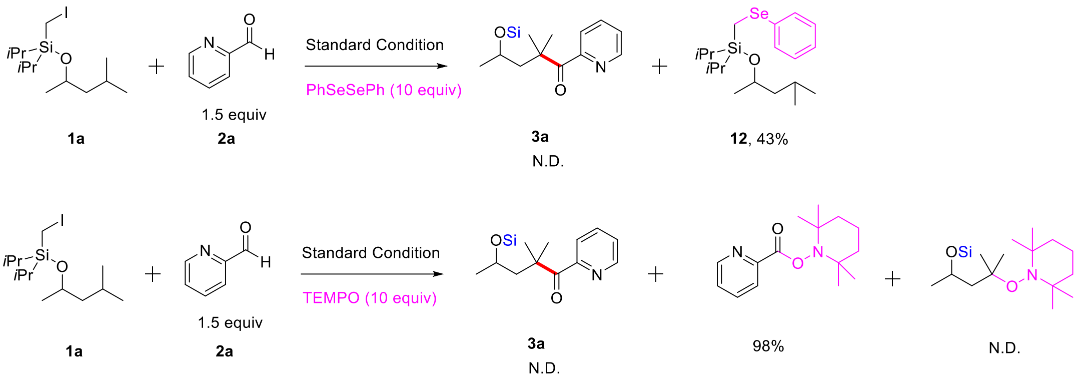

A 5 mL vial equipped with a stir bar was charged with **N2** (0.06 mmol), $Pd(OAc)_2$ (0.03 mmol), PCy_3 (0.06 mmol) and 2 mL of $PhCF_3$. After stirring for 30 min in glove box, to the solution was added Cs_2CO_3 (0.6 mmol), picolinaldehyde **2a** (0.45 mmol), styrene **5a** (0.6 mmol), alkyl silyl ether **1a** (0.3 mmol), PhSeSePh (or TEMPO) (3 mmol) and 1.0 mL of $PhCF_3$. The reaction mixtrue was removed from the glove box and stirred under 36W Blue LEDs lights at room temperature for 16 h. The solution was concentrated under reduced pressure, and purified by column chromatography on silica gel to afford the desired ketones.

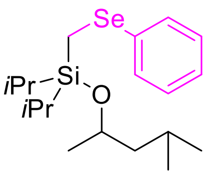

(why-09-94-2)

diisopropyl((4-methylpentan-2-yl)oxy)((phenylselanyl)methyl)silane (12)

49.8 mg, 43% yield, Colorless oil, R_f = 0.8 (petroleum ether/ethyl acetate = 25:1).

^{1}H NMR (500 MHz, $CDCl_3$) δ 7.48 – 7.43 (m, 2H), 7.28 – 7.21 (m, 2H), 7.21 – 7.16 (m, 1H), 4.09 – 4.01 (m, 1H), 2.26 (s, 2H), 1.73 – 1.65 (m, 1H), 1.49 – 1.41 (m, 1H), 1.29 – 1.22 (m, 1H), 1.19 (d, J = 6.1 Hz, 3H), 1.09 (s, 14H), 0.90 (d, 6.6 Hz, 3H), 0.88 (d, 6.6 Hz, 3H).

^{13}C NMR (126 MHz, $CDCl_3$) δ 133.9, 130.1, 128.9, 125.9, 67.8, 49.3, 24.8, 24.1, 23.1, 22.8, 17.72, 17.68, 17.66, 13.41, 13.35, 6.9.

IR (KBr) ν 2955, 2866, 1578, 1463, 1373, 1125, 1070, 1039, 994, 882, 729, 688.

HRMS (ESI) m/z: Calc. For $C_{19}H_{35}OSe$ ($[M+H]^+$) 387.1622, Found 387.1624.

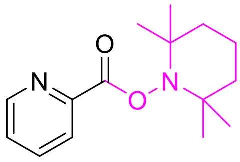

(why-09-91-1)

2,2,6,6-tetramethylpiperidin-1-yl picolinate (15)

77.0 mg, 98% yield, White solid, mp 94-97 °C, R_f = 0.1 (petroleum ether/ethyl acetate = 10:1).

^1H NMR (500 MHz, $CDCl_3$) δ 8.82 – 8.76 (m, 1H), 8.10 – 8.03 (m, 1H), 7.85 (t, J = 7.7 Hz, 1H), 7.50 – 7.44 (m, 1H), 1.85 – 1.73 (m, 2H), 1.73 – 1.67 (m, 1H), 1.64 – 1.55 (m, 2H), 1.50 – 1.44 (m, 1H), 1.30 (s, 6H), 1.16 (s, 6H).

^{13}C NMR (126 MHz, $CDCl_3$) δ 164.9, 150.1, 148.1, 136.7, 126.5, 124.6, 60.4, 39.0, 20.7, 16.9.

IR (KBr) ν 2975, 2939, 1762, 1733, 1300, 1282, 1224, 1109, 1071, 1045, 755, 703.

HRMS (ESI) m/z: Calc. For $C_{15}H_{22}N_2O_2Na$ ($[M+Na]^+$) 285.1574, Found 285.1573.

2.5.2 Crossover experiment:

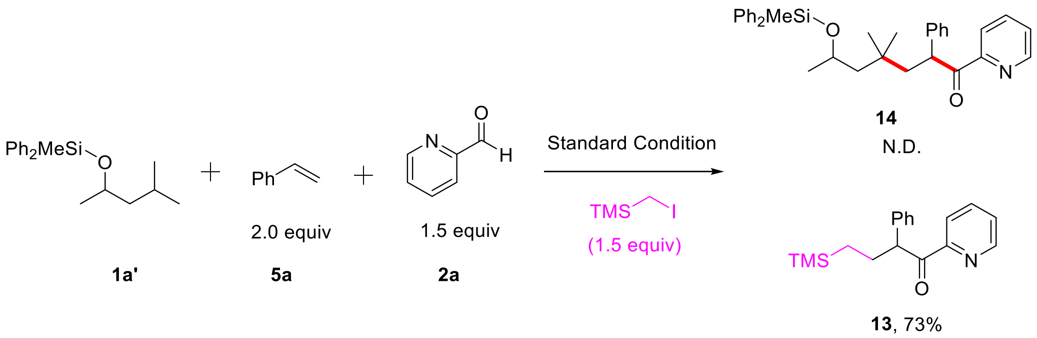

A 5 mL vial equipped with a stir bar was charged with **N2** (0.06 mmol), $Pd(OAc)_2$ (0.03 mmol), PCy_3 (0.06 mmol) and 2 mL of $PhCF_3$. After stirring for 30 min in glove box, to the solution was added Cs_2CO_3 (97.8 mg, 0.6 mmol), picolinaldehyde **2a** (0.45

mmol), styrene **5a** (0.6 mmol), alkyl silyl ether **1a'** (0.3 mmol), trimethyl(iodomethyl)silane (0.45 mmol) and 1.0 mL of $PhCF_3$. The reaction mixtrue was removed from the glove box and stirred under 36W Blue LEDs lights at room temperature for 16 h. The solution was concentrated under reduced pressure, and purified by column chromatography on silica gel with petroleum ether/ethyl acetate = 10 : 1 to afford the desired ketones **12** in 73% yield .

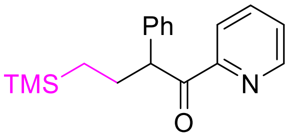

(why-09-97-2)

2-phenyl-1-(pyridin-2-yl)-4-(trimethylsilyl)butan-1-one (13)[6]

86.9 mg, 73% yield, Light yellow oil, R_f = 0.5 (petroleum ether/ethyl acetate = 10:1).

^{1}H NMR (500 MHz, $CDCl_3$) δ 8.69 – 8.64 (m, 1H), 8.01 (dt, *J* = 7.9, 1.1 Hz, 1H), 7.72 (td, *J* = 7.6, 1.8 Hz, 1H), 7.43 (dd, *J* = 8.1, 1.4 Hz, 2H), 7.38 – 7.33 (m, 1H), 7.27 (t, *J* = 7.7 Hz, 2H), 7.18 (t, *J* = 7.3, 1H), 5.39 (t, *J* = 7.5 Hz, 1H), 2.25 – 2.18 (m, 1H), 1.95 – 1.86 (m, 1H), 0.58 – 0.44 (m, 2H), 0.00 (s, 9H).

^{13}C NMR (126 MHz, $CDCl_3$) δ 201.7, 153.3, 148.7, 139.4, 136.6, 128.9, 128.4, 126.7, 126.6, 122.5, 53.8, 27.6, 14.8, -1.9.

IR (KBr) ν 3058, 2952, 1695, 1582, 1342, 1247, 1218, 860, 836, 757, 744, 701.

HRMS (ESI) m/z: Calc. For $C_{18}H_{24}NO_2Si$ ($[M+H]^+$) 298.1622, Found 298.1620.

2.5.3 Cation and anion trapping experiment

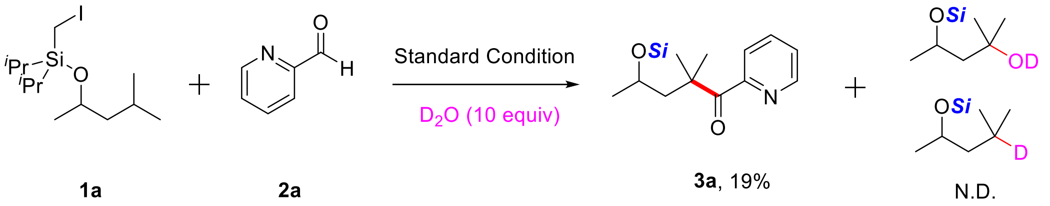

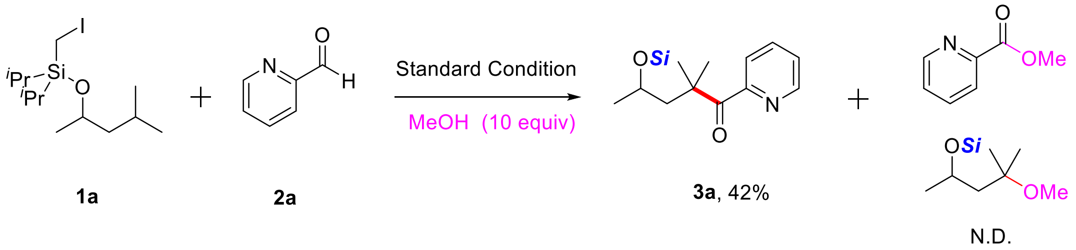

A 5 mL vial equipped with a stir bar was charged with **N4** (0.06 mmol), $Pd(OAc)_2$ (0.03 mmol), PCy_3 (0.06 mmol) and 2 mL of $PhCF_3$. After stirring for 30 min in glove box, to the solution was added Cs_2CO_3 (0.6 mmol), picolinaldehyde **2a** (0.45 mmol), alkyl silyl ether **1a** (0.3 mmol), D_2O (or MeOH) (10 equiv) and 1.0 mL of $PhCF_3$. The reaction mixtrue was removed from the glove box and stirred under 36W Blue LEDs at room temperature for 16 h. The solution was concentrated under reduced pressure, and purified by column chromatography on silica gel to afford the desired products.

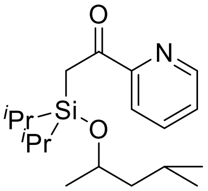

(why-09-91-4)

2-(diisopropyl((4-methylpentan-2-yl)oxy)silyl)-1-(pyridin-2-yl)ethan-1-one (4a)

12.1 mg, 12% yield, Colorless oil, R_f = 0.5 (petroleum ether/ethyl acetate = 25:1).

^{1}H NMR (500 MHz, $CDCl_3$) δ 8.65 (dd, *J* = 4.8, 1.4 Hz, 1H), 8.02 (d, *J* = 7.8 Hz, 1H), 7.80 (td, *J* = 7.6, 1.8 Hz, 1H), 7.42 (dd, *J* = 7.6, 4.6 Hz, 1H), 4.02 – 3.99 (m, 1H), 3.19 (s, 2H), 1.60 – 1.54 (m, 1H), 1.34 – 1.26 (m, 1H), 1.19 – 1.11 (m, 1H), 1.06 (d, *J* = 6.1 Hz, 3H), 1.05 – 0.99 (m, 14H), 0.87 – 0.80 (m, 6H).

^{13}C NMR (126 MHz, $CDCl_3$) δ 201.2, 154.6, 148.5, 136.7, 126.5, 121.6, 67.7, 49.0, 27.0, 24.7, 23.6, 23.1, 22.7, 17.35, 17.32, 13.88, 13.87.

IR (KBr) ν 2940, 2865, 1685, 1462, 1251, 1134, 1079, 1060, 996, 957, 882, 780, 739, 705.

HRMS (ESI) m/z: Calc. For $C_{19}H_{34}NO_2Si$ ([M+ H]$^+$) 336.2353, Found 336.2352.

2.5.4 Light on/off experiments

A 5 mL vial equipped with a stir bar was charged with **N2** (0.06 mmol), $Pd(OAc)_2$ (0.03 mmol), PCy_3 (0.06 mmol) and 2 mL of $PhCF_3$. After stirring for 30 min in glove box, to the solution was added Cs_2CO_3 (97.8 mg, 0.6 mmol), picolinaldehyde **2a** (0.45 mmol), alkyl silyl ether **1a** (0.3 mmol), and 1.0 mL of $PhCF_3$. The reaction mixtrue was removed from the glove box and stirred under 36W Blue LEDs at room temperature for

2 h, followed by 2 h in the dark, which was continued for a total reaction time of 12 h. After each sequence. The aliquot was taken and the reaction was analyzed by ^{1}H NMR using CH_2Br_2 as standard.

Time / h	Yield / %
0 (on)	0
2 (off)	16
4 (on)	16
6 (off)	31
8 (on)	31
10(off)	45
12(off)	45

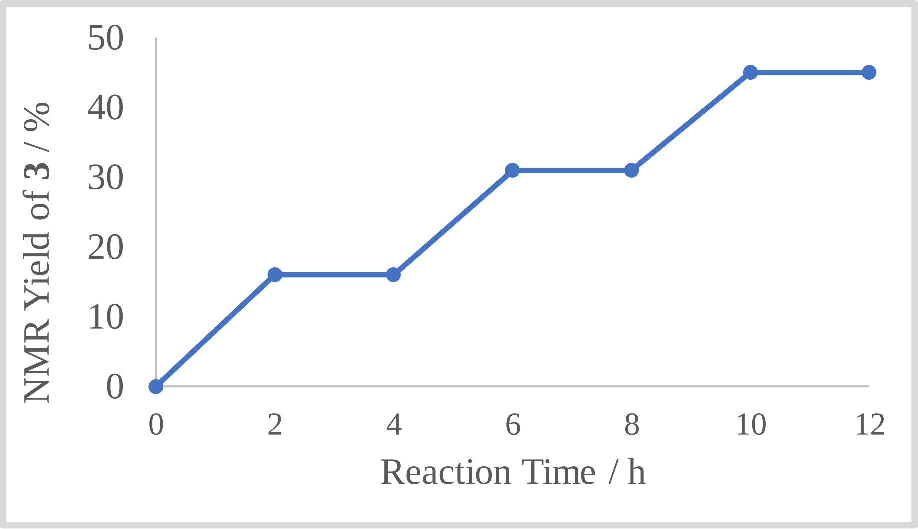

Supplementary Figure 1 Light on/off experiments

2.5.5 UV-Visible absorption analysis

UV/Vis absorption spectra were recorded on a Jasco V-650 spectrophotometer, equipped with a temperature control unit at 25 °C. The samples were measured in Hellma fluorescence QS quartz cuvettes (chamber volume = 3.0 mL) fitted with a PTFE stopper. The UV-visible absorption of the substrates $Pd(OAc)_2$ (1.0 x 10^{-3} M), PCy_3 (2.0 x 10^{-3} M), , **N2** (2.0 x 10^{-3} M), picolinaldehyde **2a** (1.5 x 10^{-2} M) and alkyl silyl ether **1a** (1.0 x 10^{-3} M) were determined in $PhCF_3$.[7]

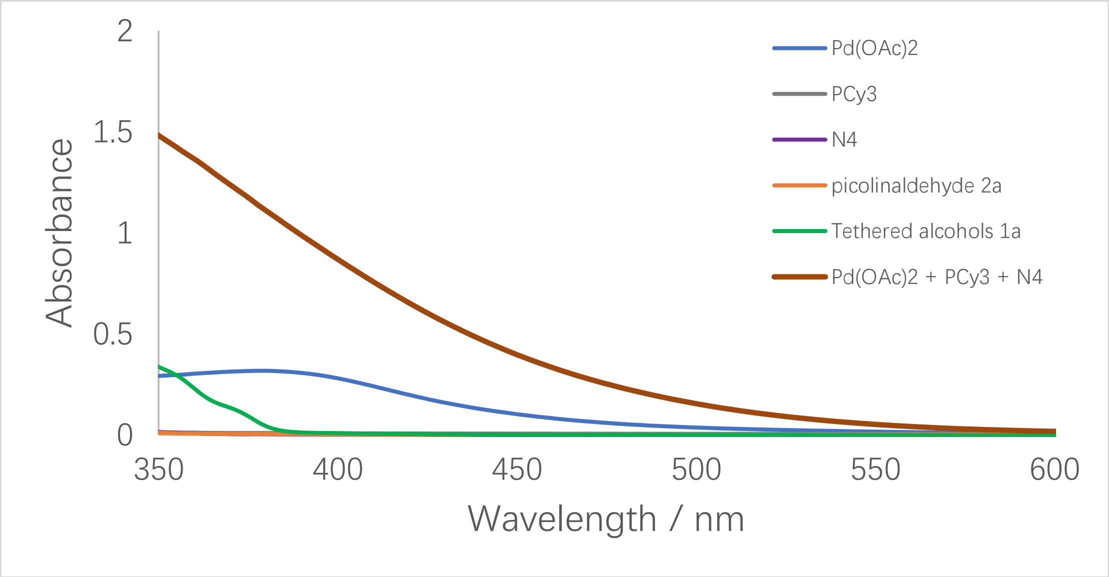

Supplementary Figure 2 UV-Visible absorption spectra

3. Supplementary Figures

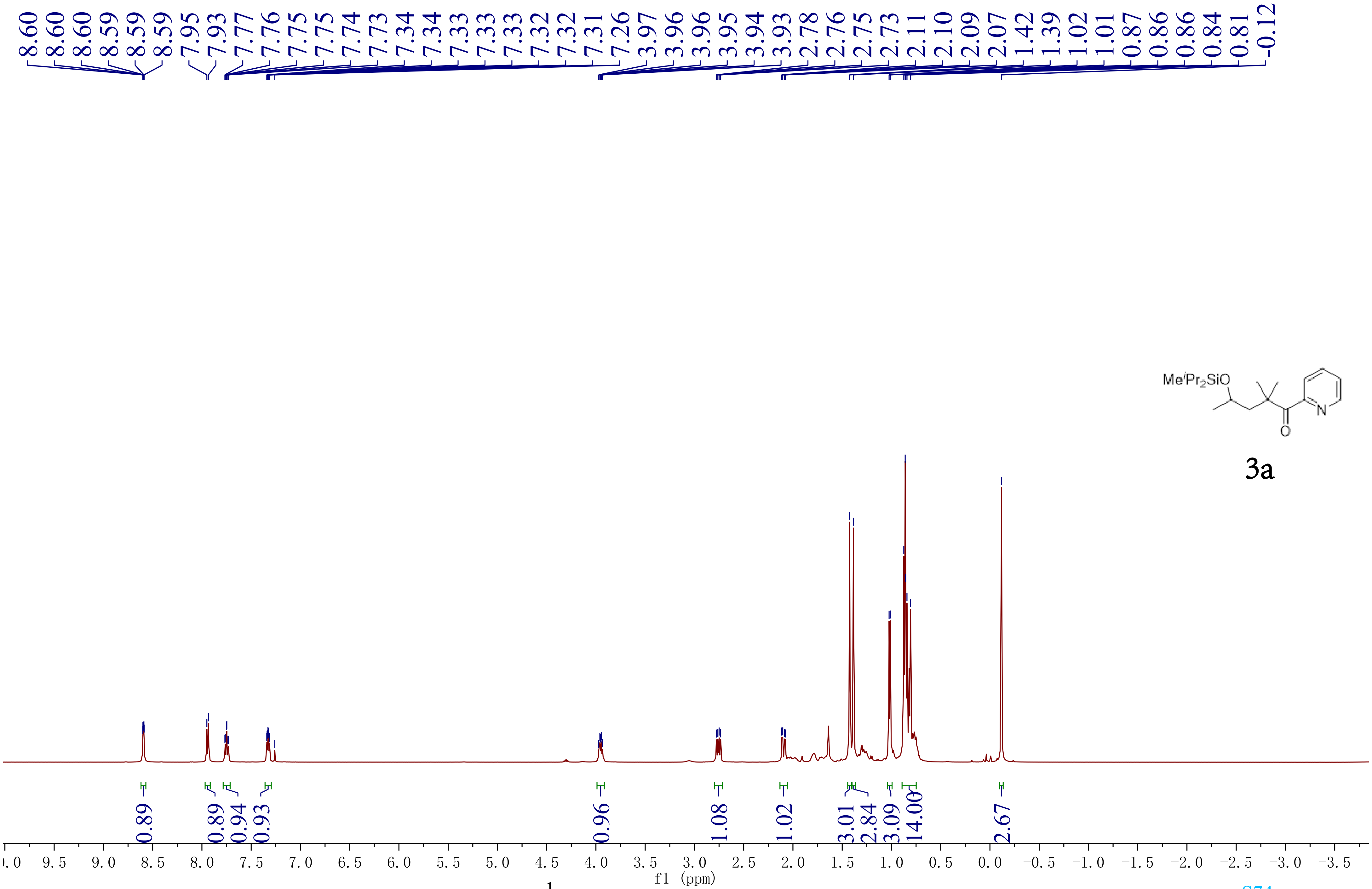

3a

Supplementary Figure 3. ^{1}H NMR spectrum of **3a**, recorded at 500 MHz and 25 °C in $CDCl_3$

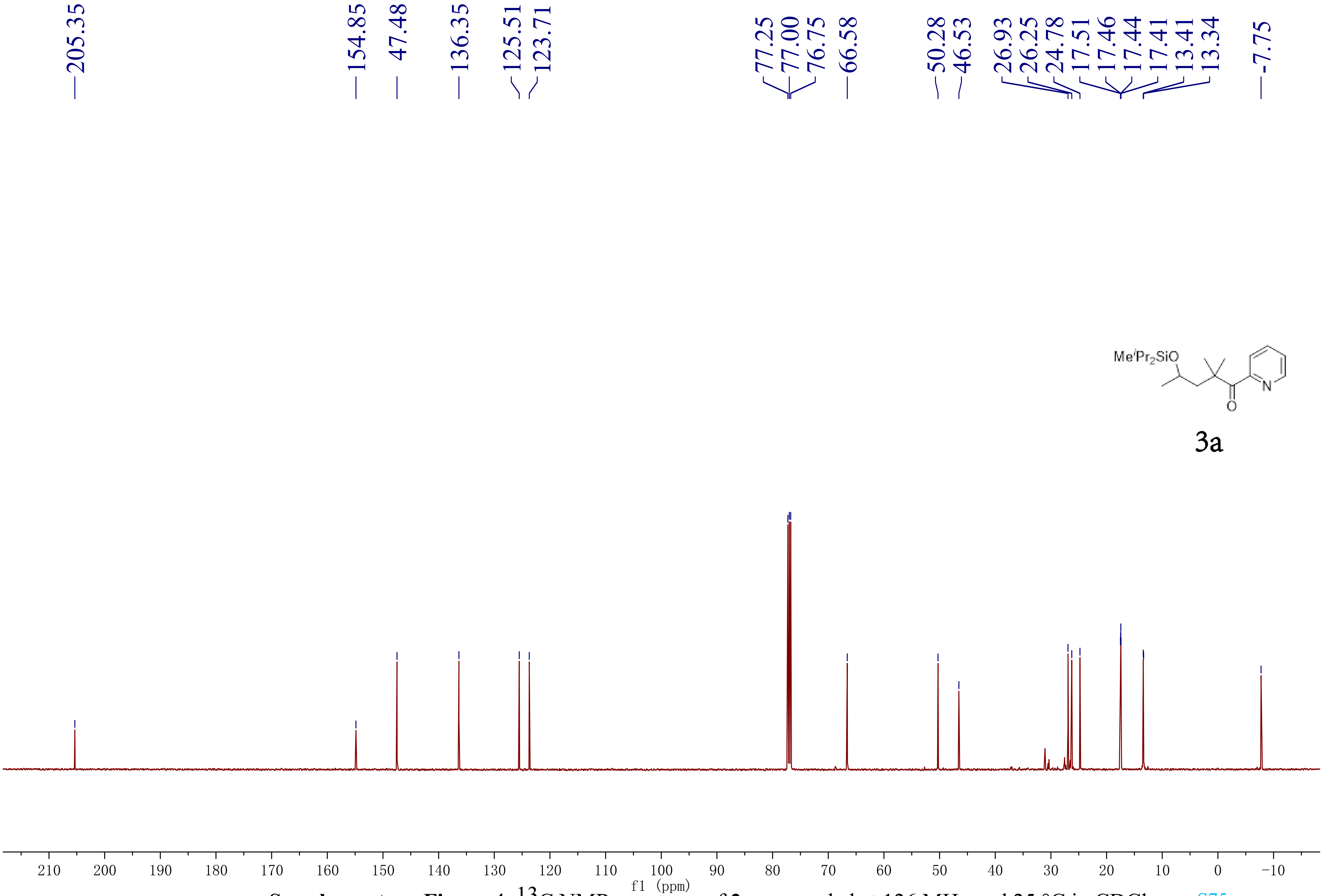

Supplementary Figure 4. ^{13}C NMR spectrum of **3a**, recorded at 126 MHz and 25 °C in $CDCl_3$

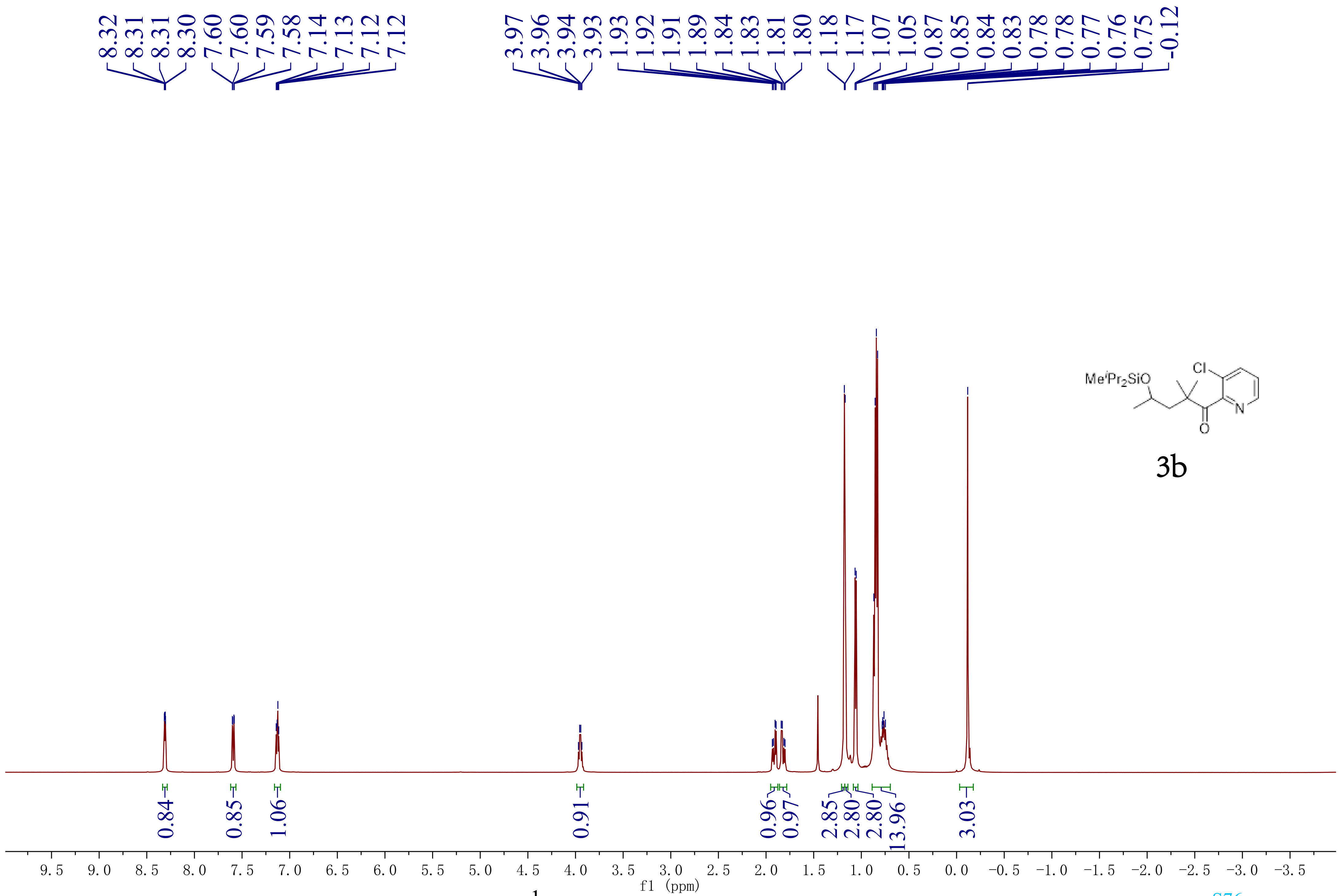

Supplementary Figure 5. ^{1}H NMR spectrum of **3b**, recorded at 500 MHz and 25 °C in $CDCl_3$

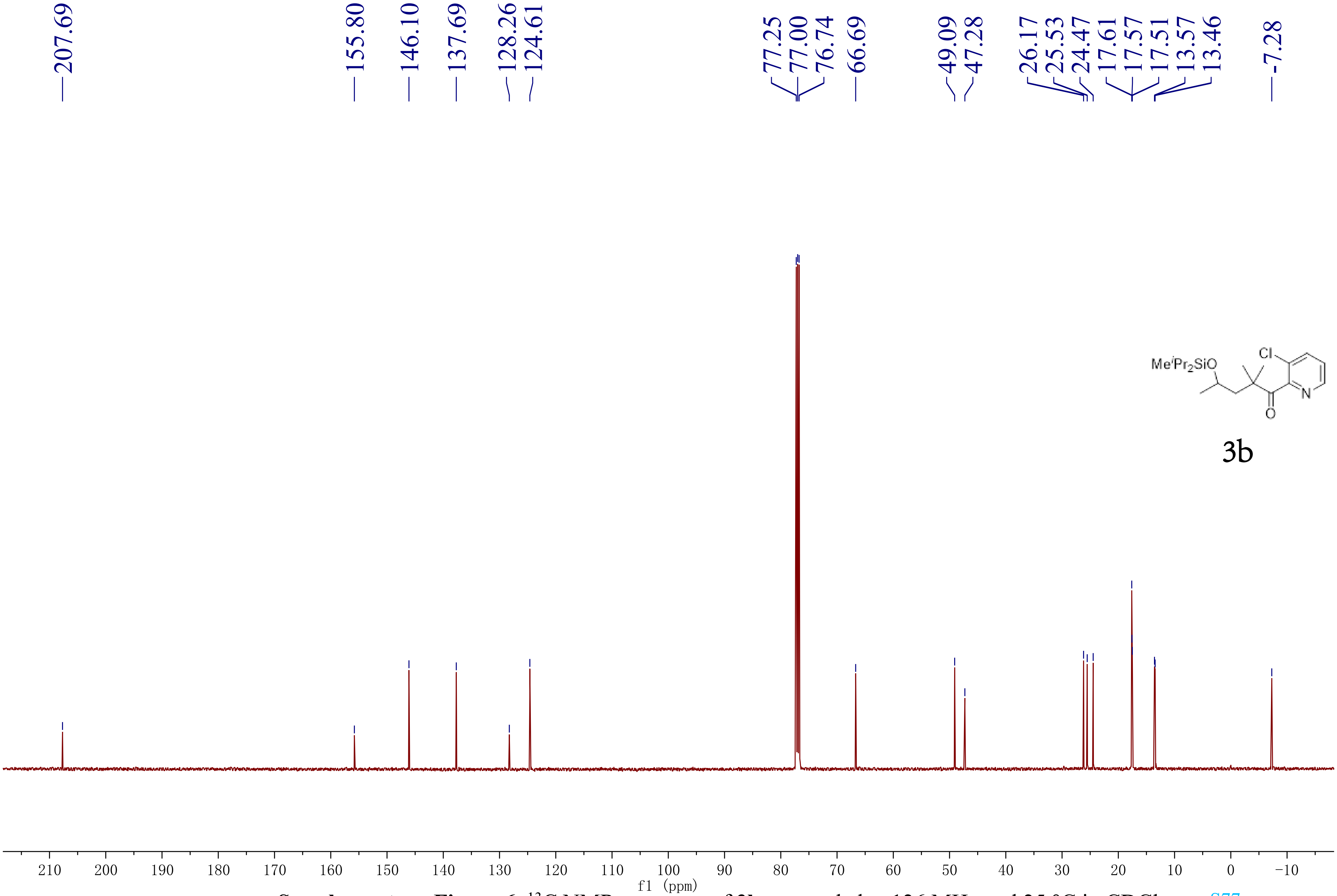

3b

Supplementary Figure 6. ^{13}C NMR spectrum of **3b**, recorded at 126 MHz and 25 °C in $CDCl_3$

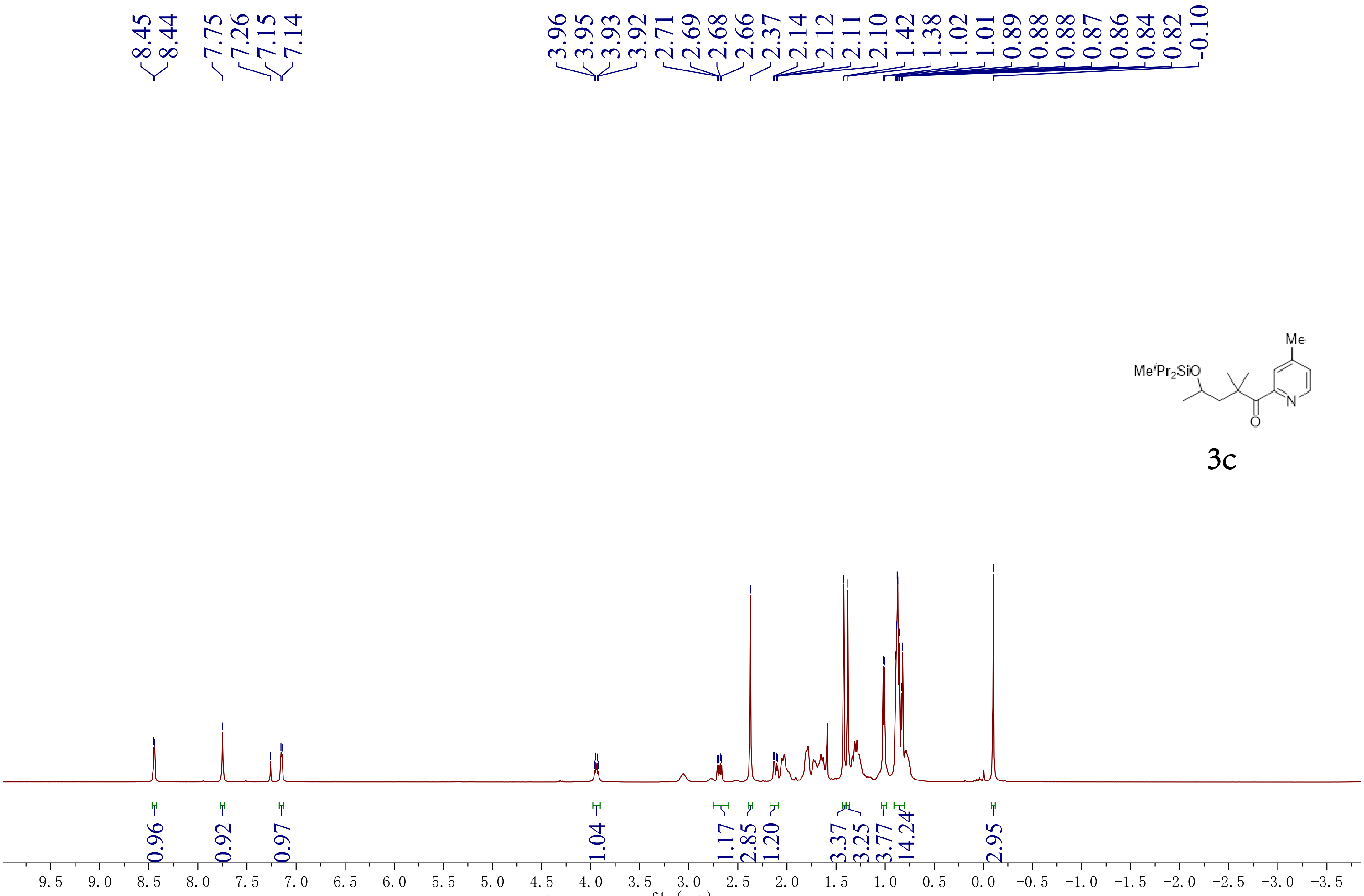

Supplementary Figure 7. ^{1}H NMR spectrum of **3c,** recorded at 500 MHz and 25 °C in $CDCl_3$

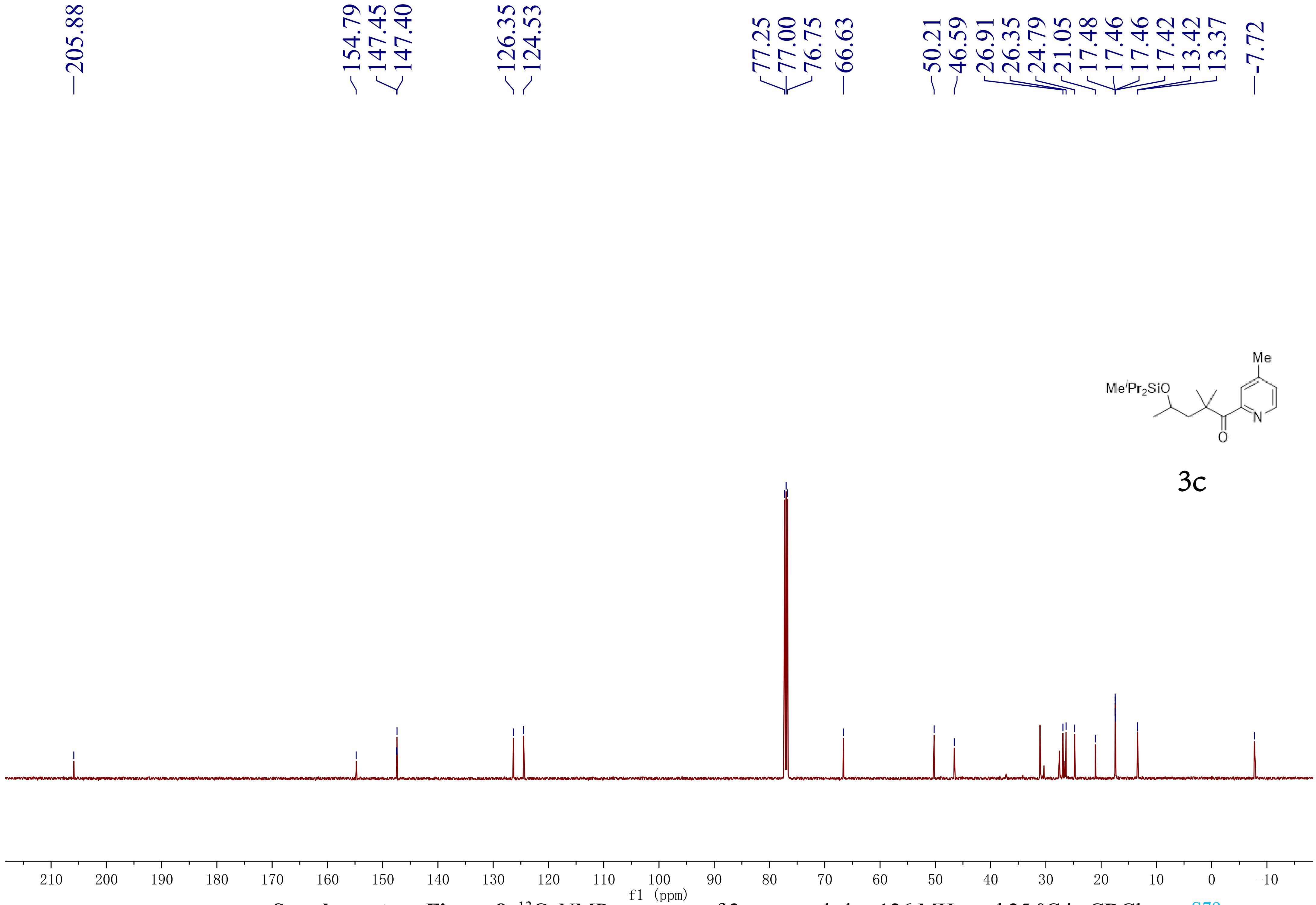

3c

Supplementary Figure 8. ^{13}C NMR spectrum of **3c**, recorded at 126 MHz and 25 °C in $CDCl_3$

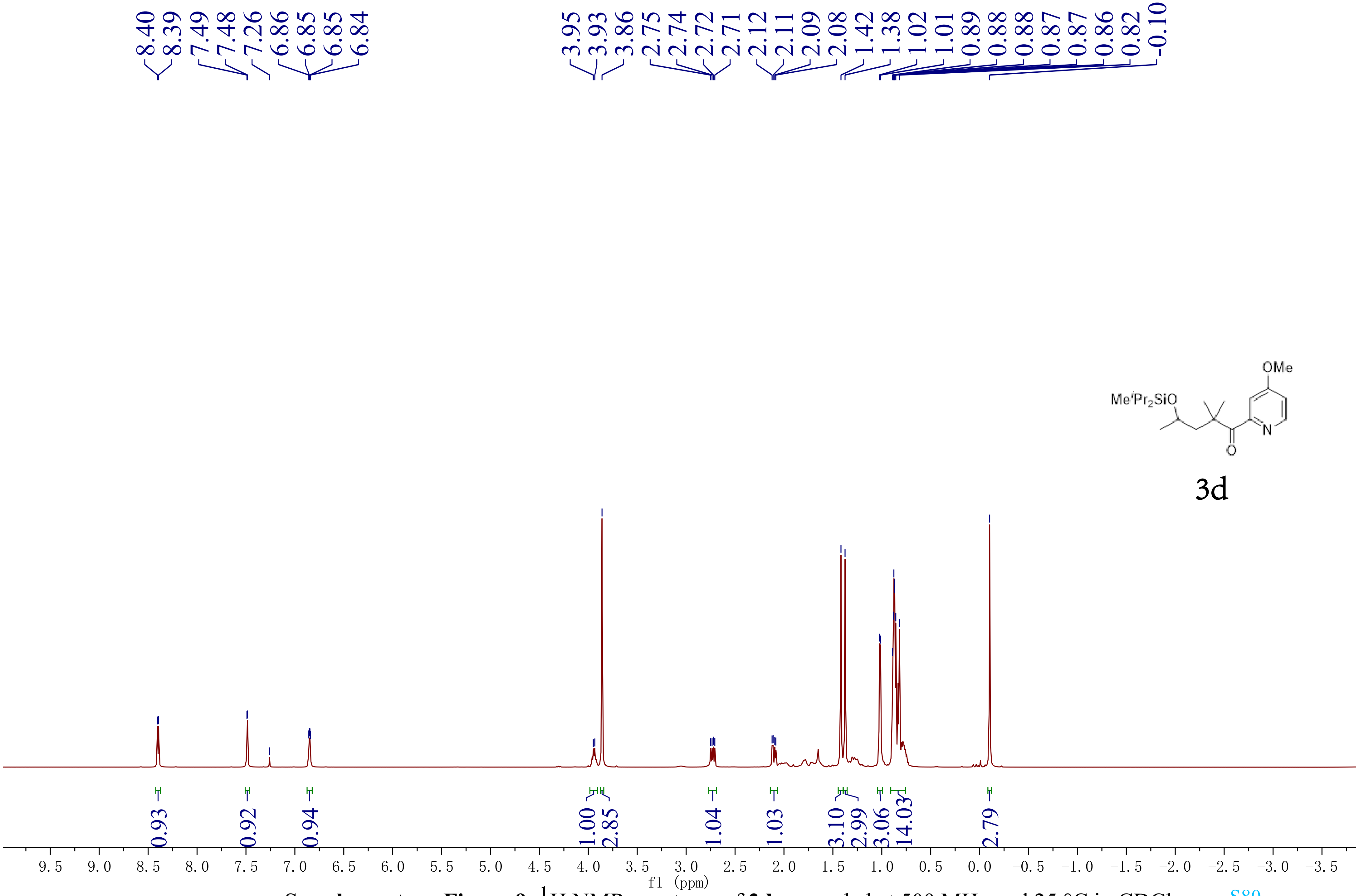

Supplementary Figure 9. ^{1}H NMR spectrum of **3d**, recorded at 500 MHz and 25 °C in $CDCl_3$

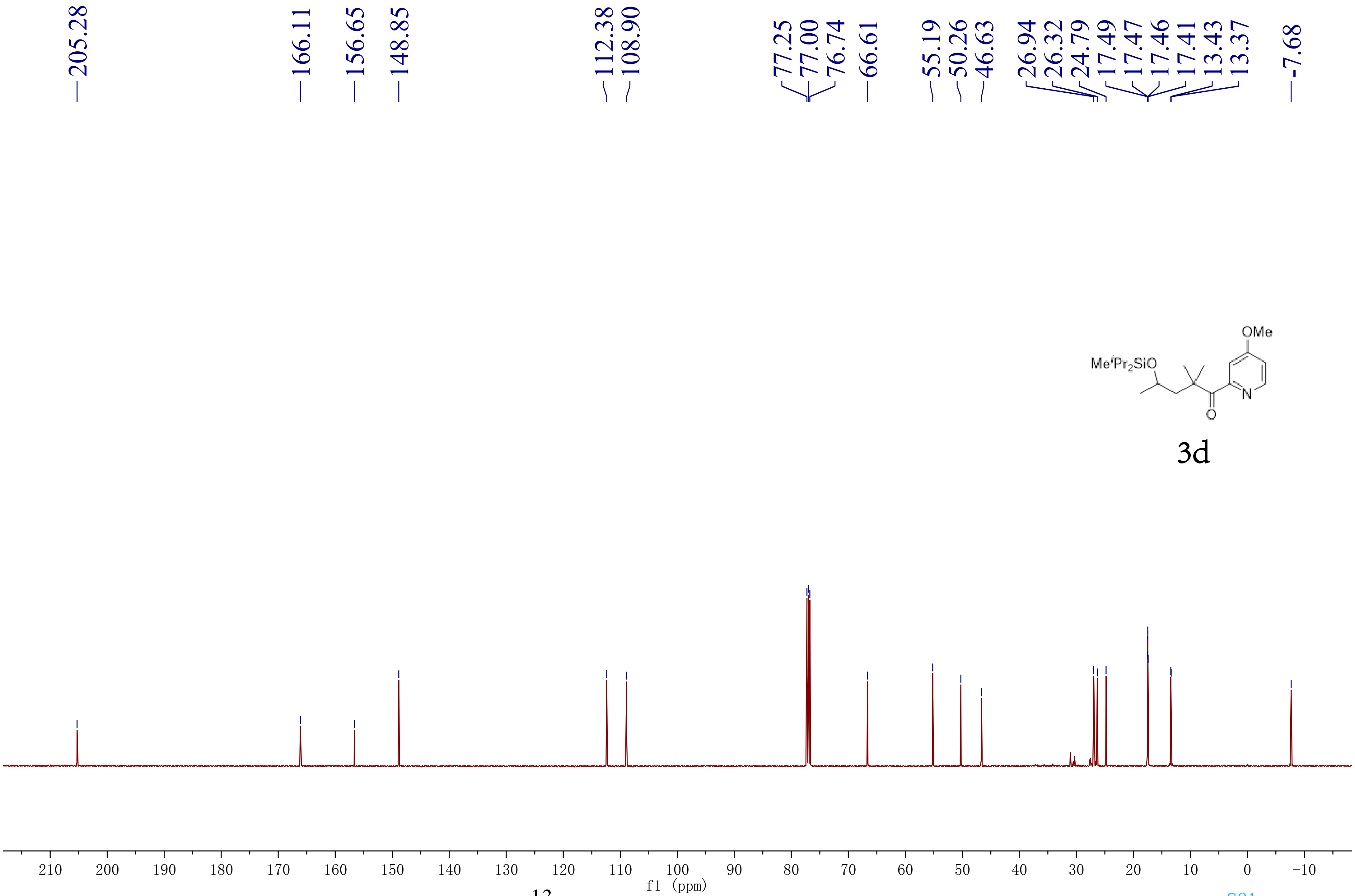

Supplementary Figure 10. ^{13}C NMR spectrum of **3d**, recorded at 126 MHz and 25 °C in $CDCl_3$

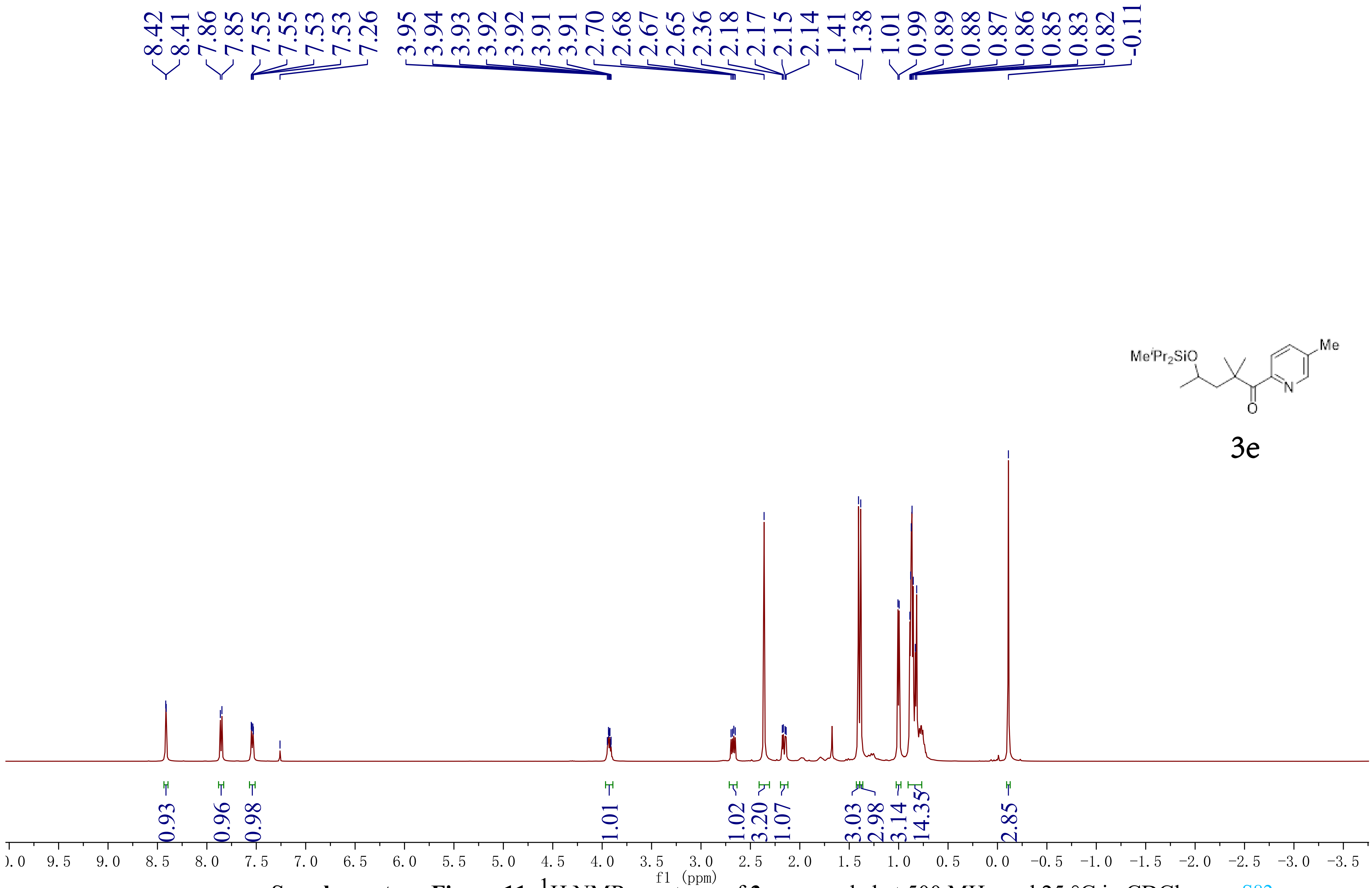

Supplementary Figure 11. ^{1}H NMR spectrum of **3e**, recorded at 500 MHz and 25 °C in $CDCl_3$

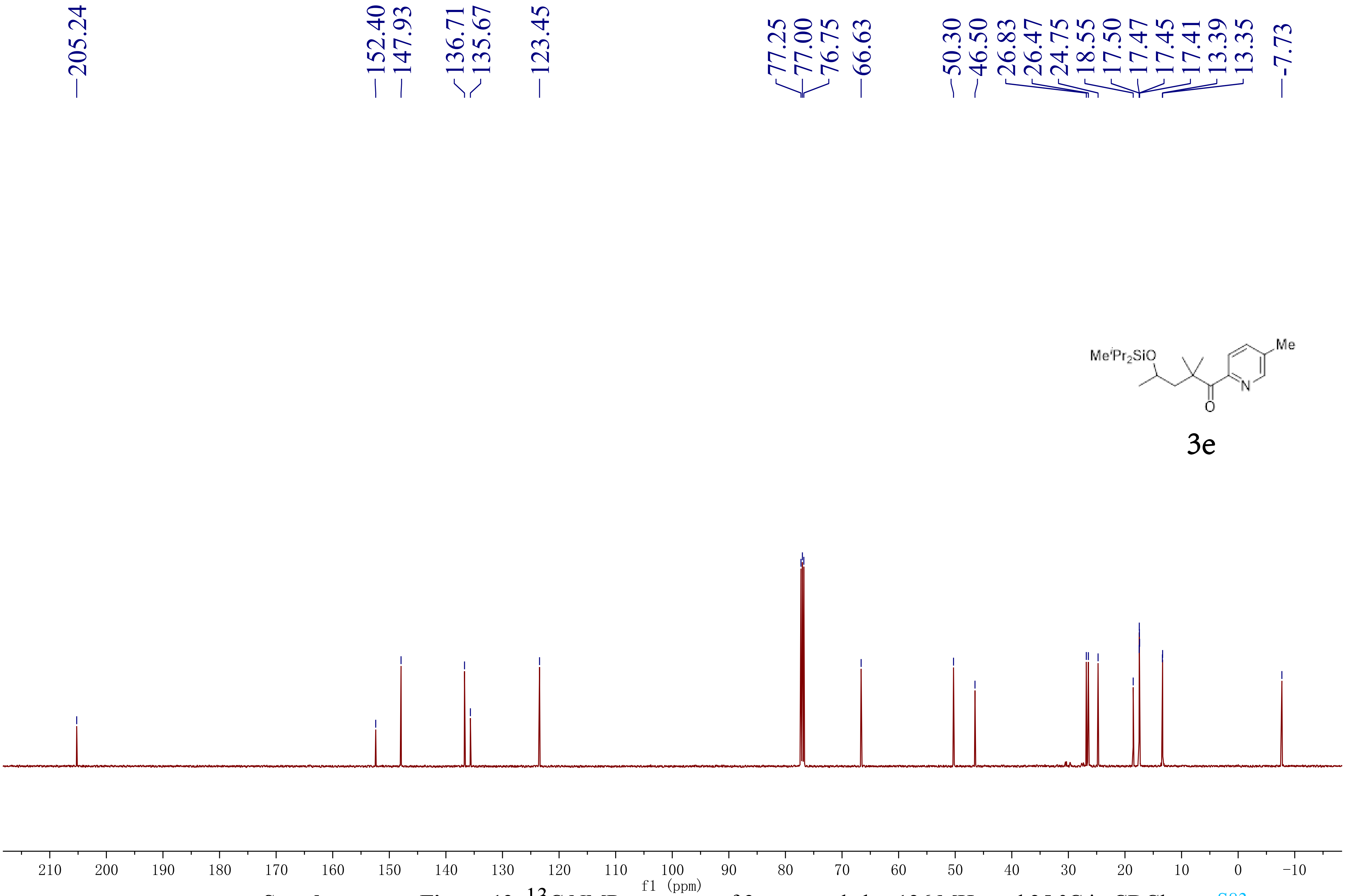

3e

Supplementary Figure 12 . ^{13}C NMR spectrum of **3e**， recorded at 126 MHz and 25 °C in $CDCl_3$

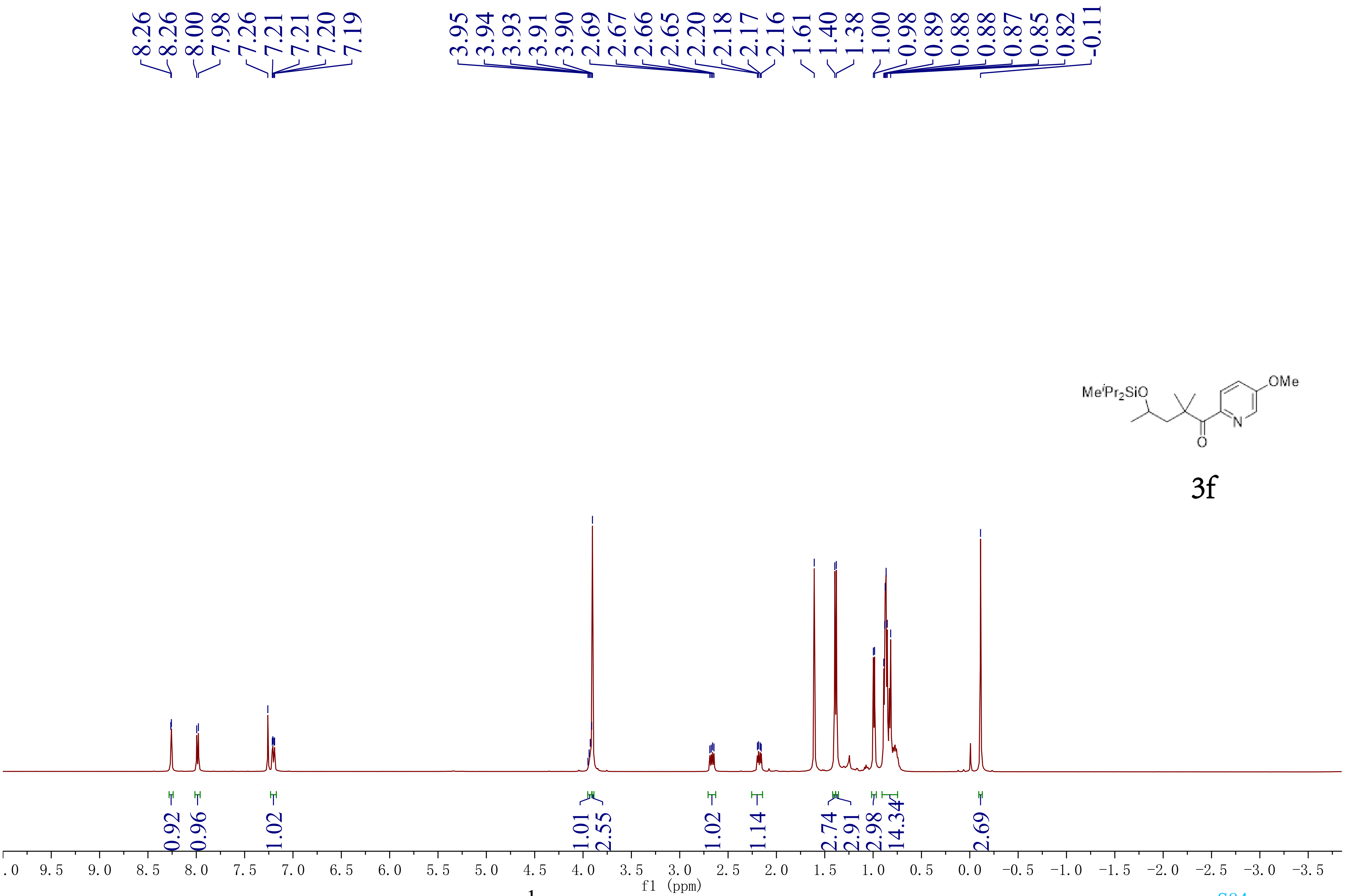

Supplementary Figure 13. ^{1}H NMR spectrum of **3f**, recorded at 500 MHz and 25 °C in $CDCl_3$

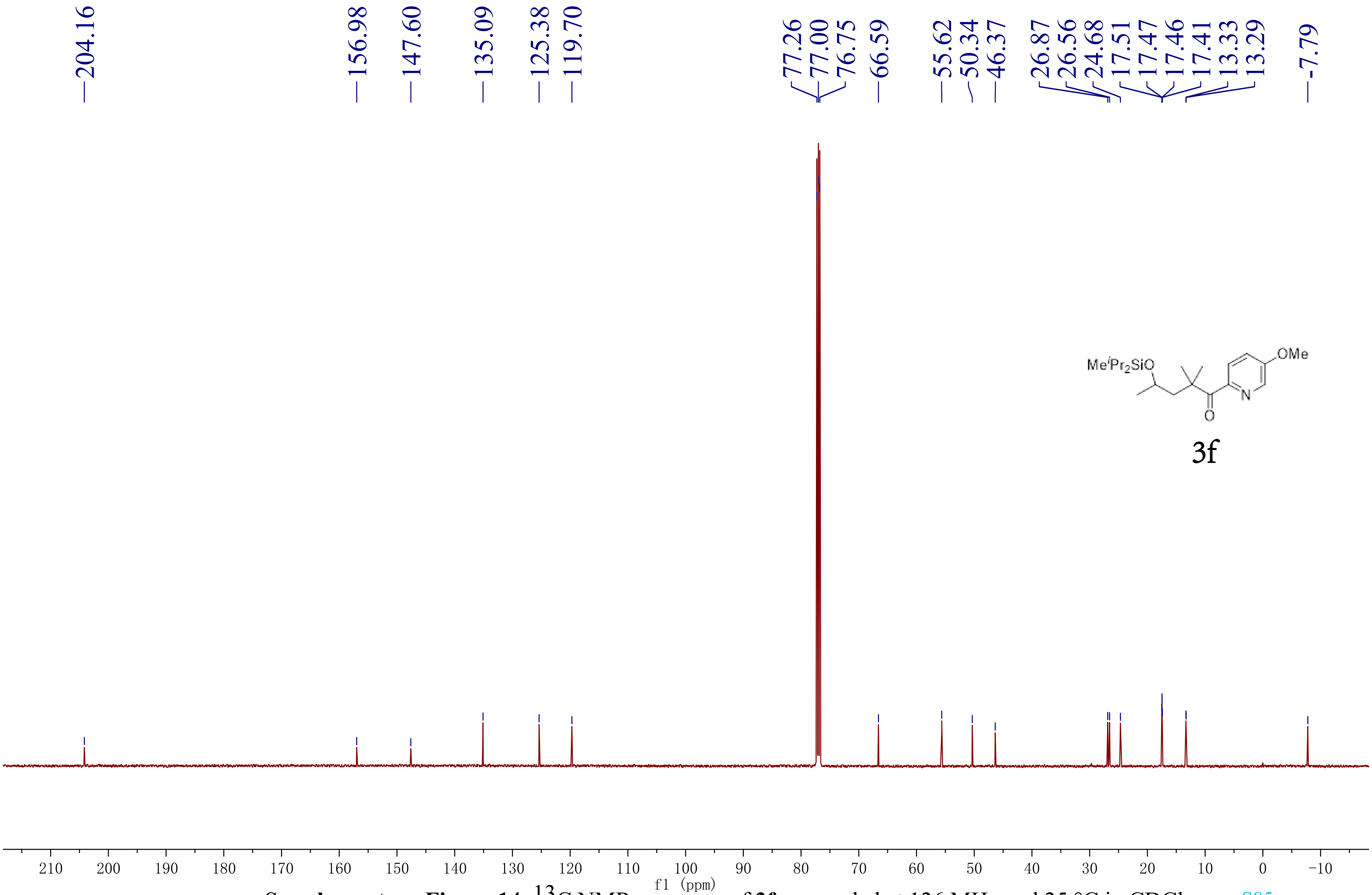

Supplementary Figure 14. ^{13}C NMR spectrum of **3f**, recorded at 126 MHz and 25 °C in $CDCl_3$

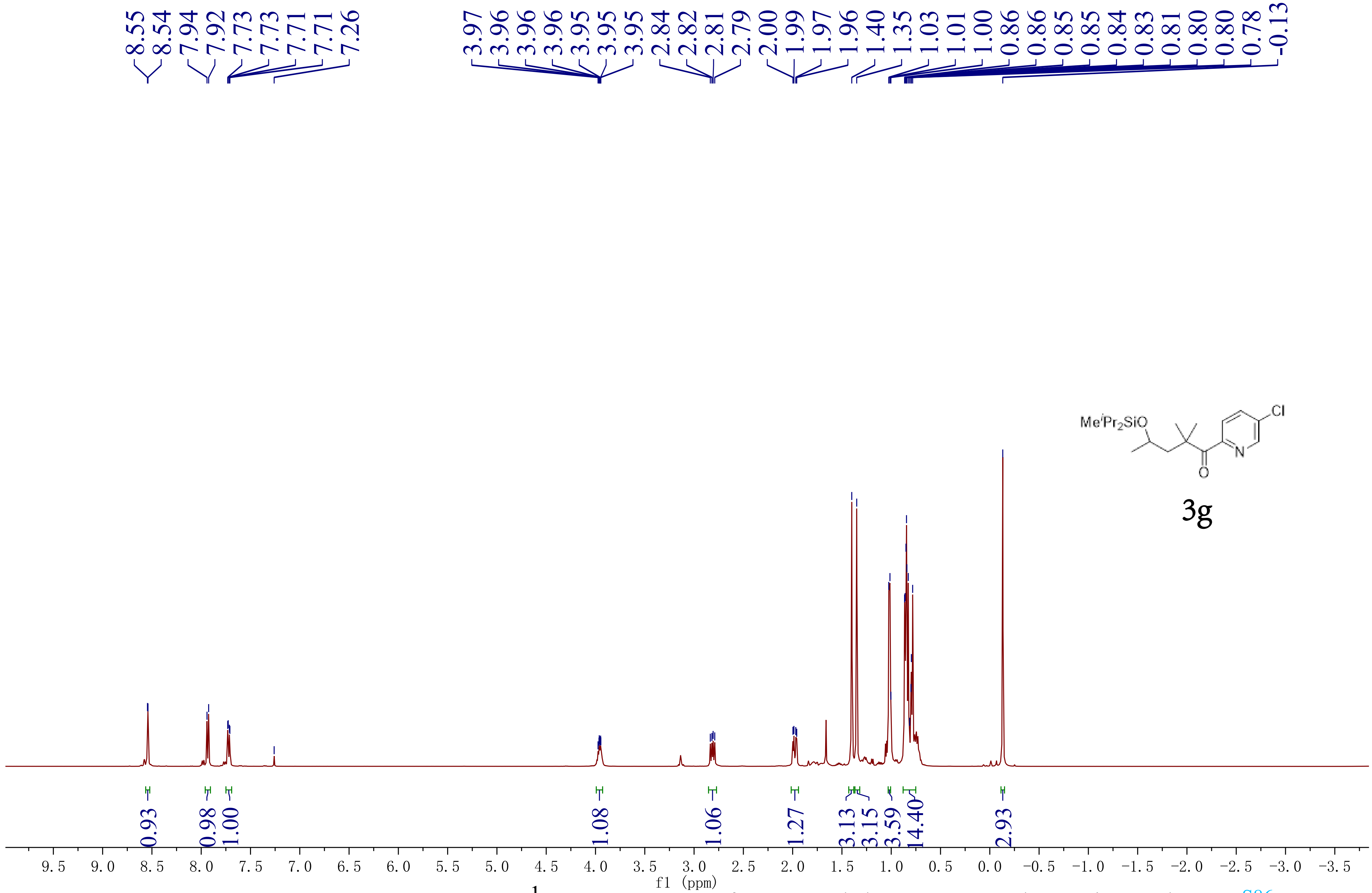

Supplementary Figure 15. ^{1}H NMR spectrum of **3g**, recorded at 500 MHz and 25 °C in $CDCl_3$

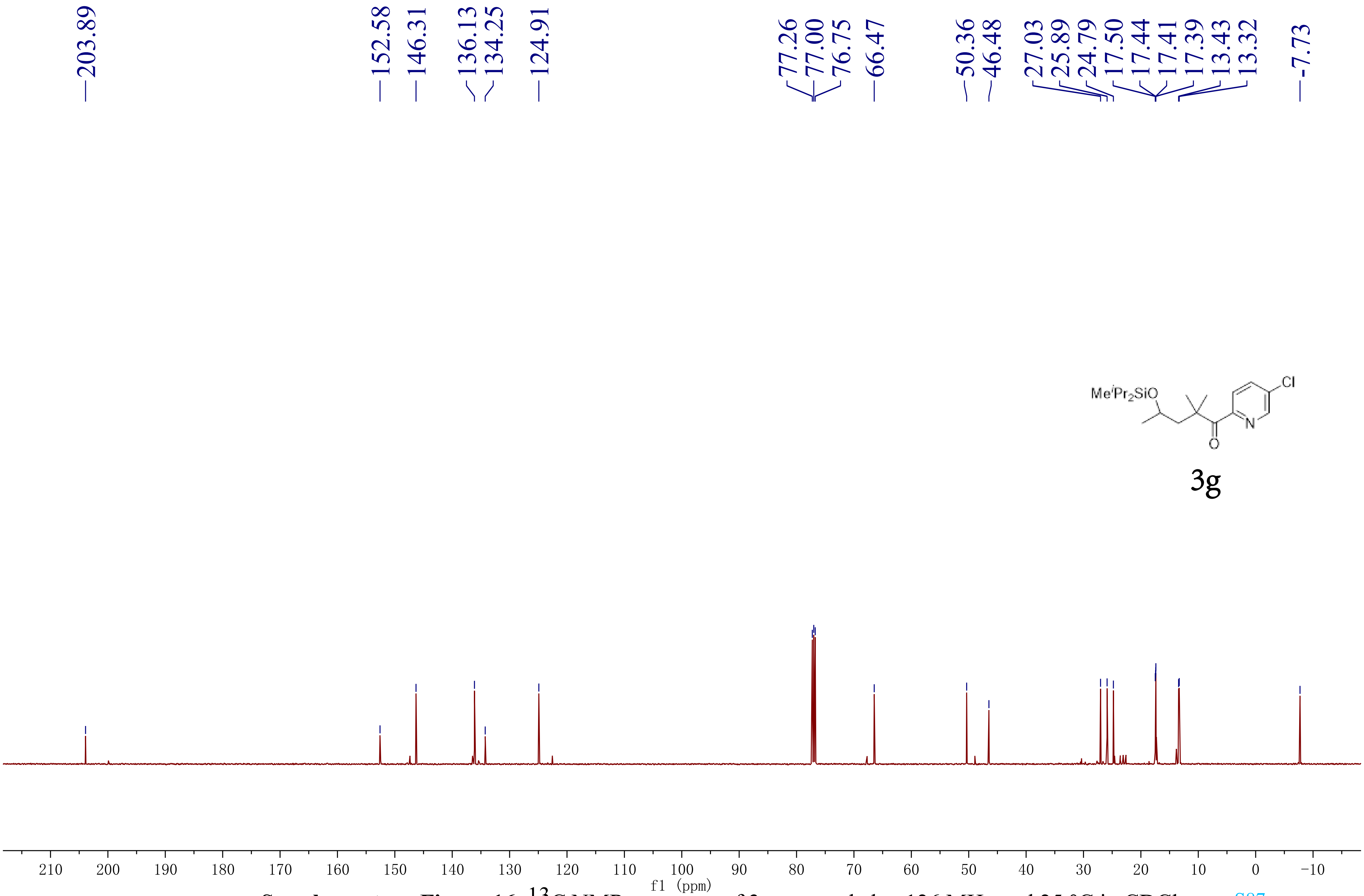

3g

Supplementary Figure 16. ^{13}C NMR spectrum of **3g**, recorded at 126 MHz and 25 °C in $CDCl_3$

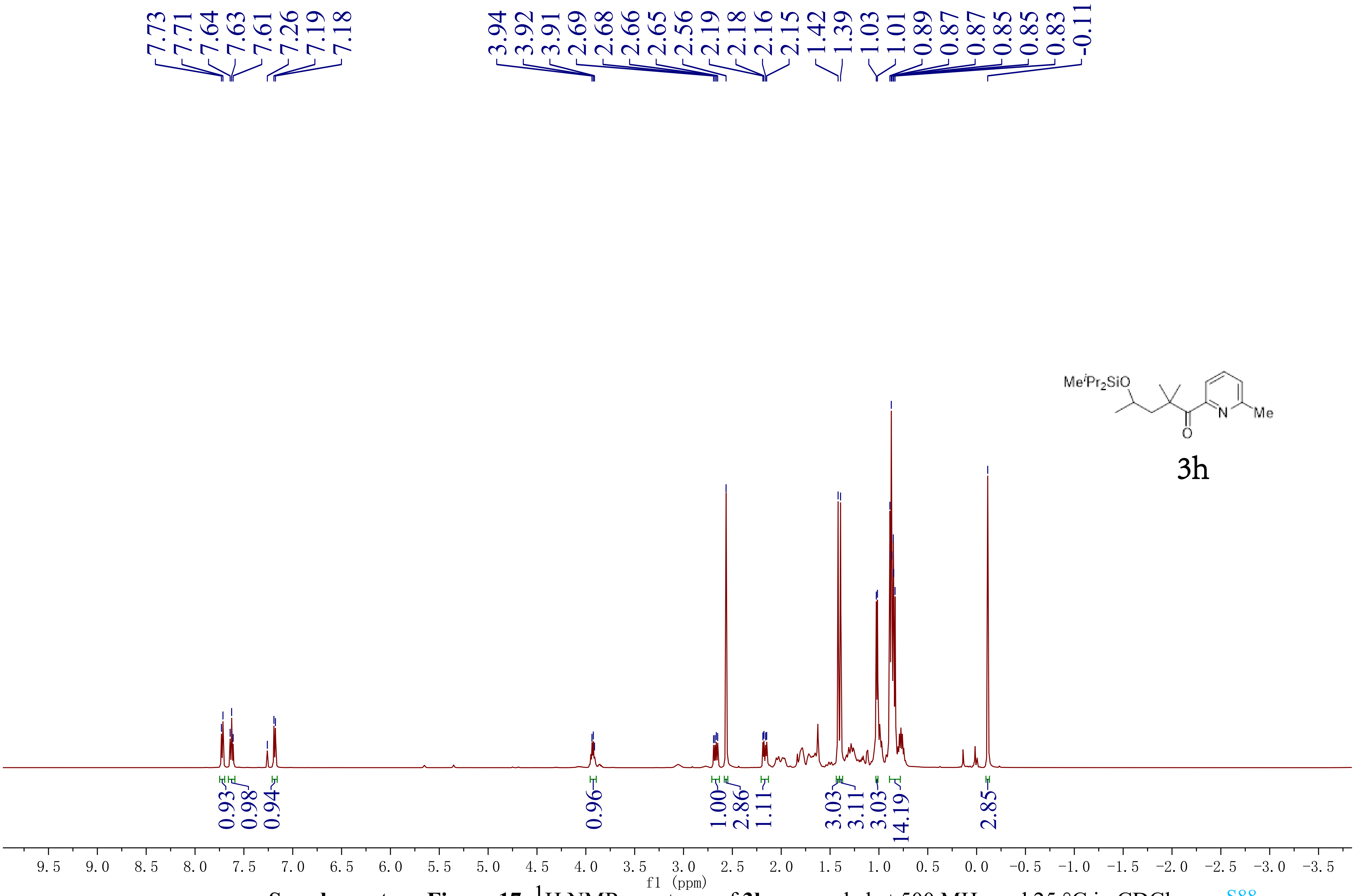

Supplementary Figure 17. ^{1}H NMR spectrum of **3h**, recorded at 500 MHz and 25 °C in $CDCl_3$

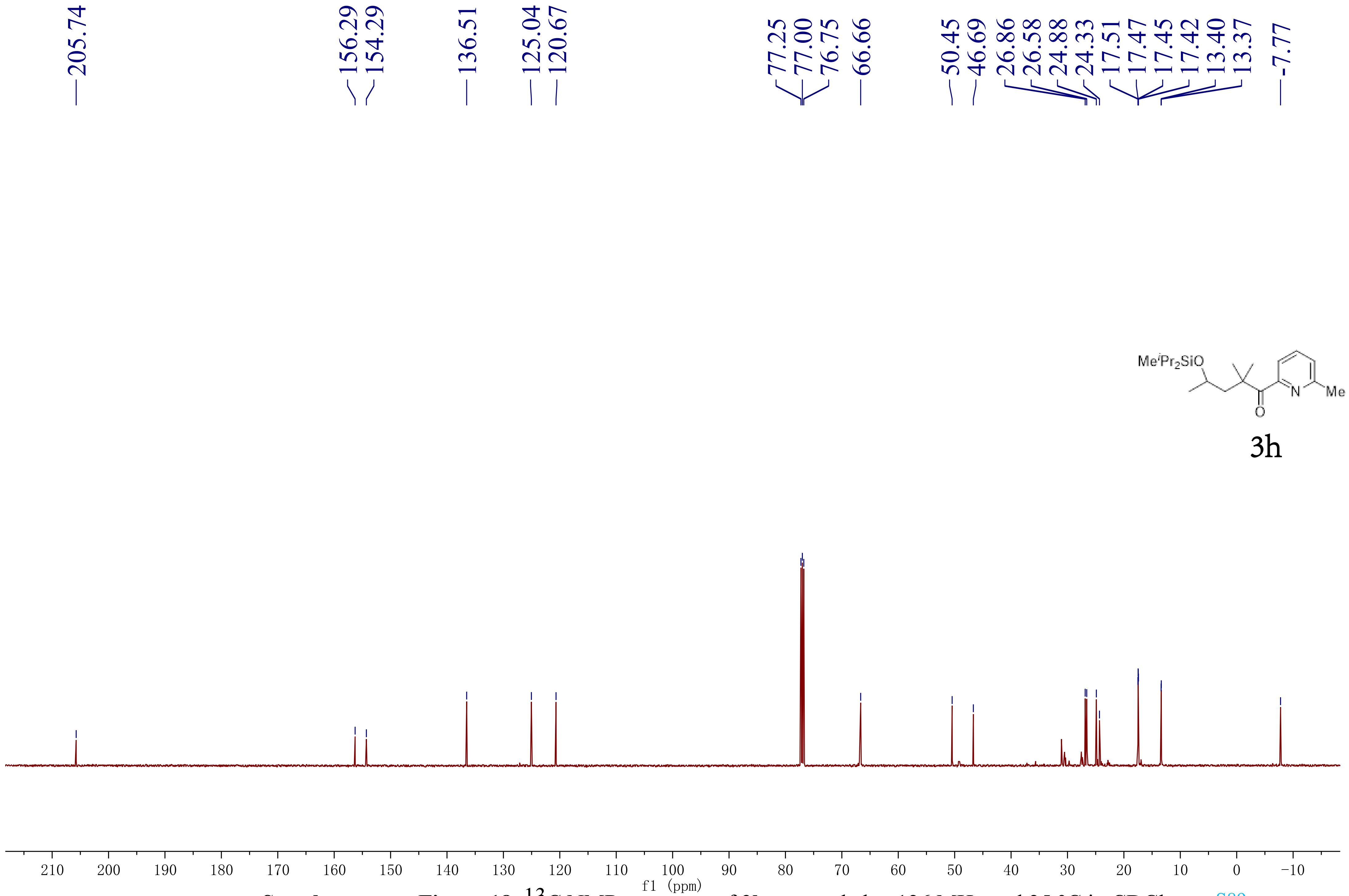

Supplementary Figure 18. ^{13}C NMR spectrum of **3h**, recorded at 126 MHz and 25 °C in $CDCl_3$

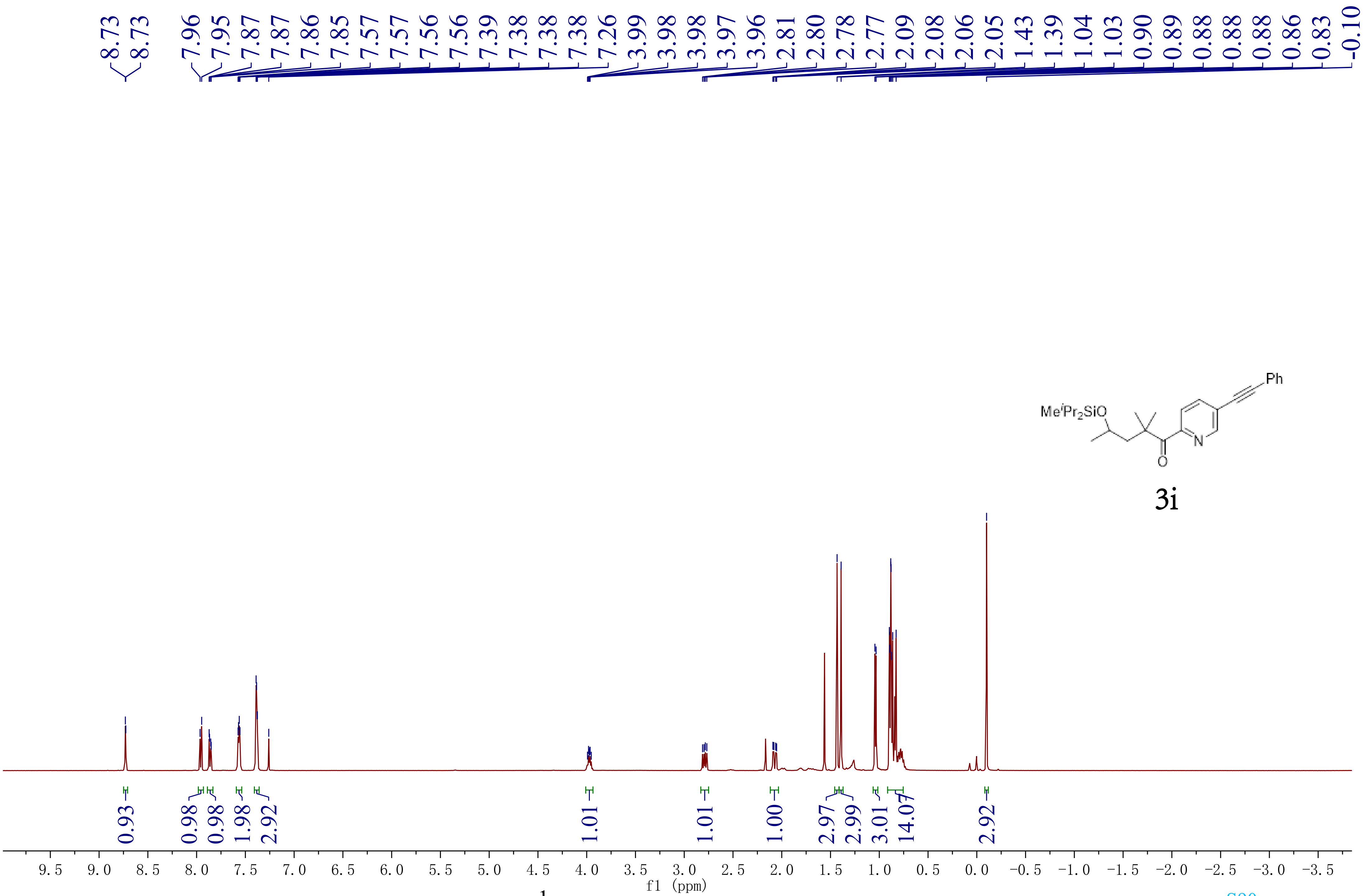

Supplementary Figure 19. ^{1}H NMR spectrum of **3i**, recorded at 500 MHz and 25 °C in $CDCl_3$

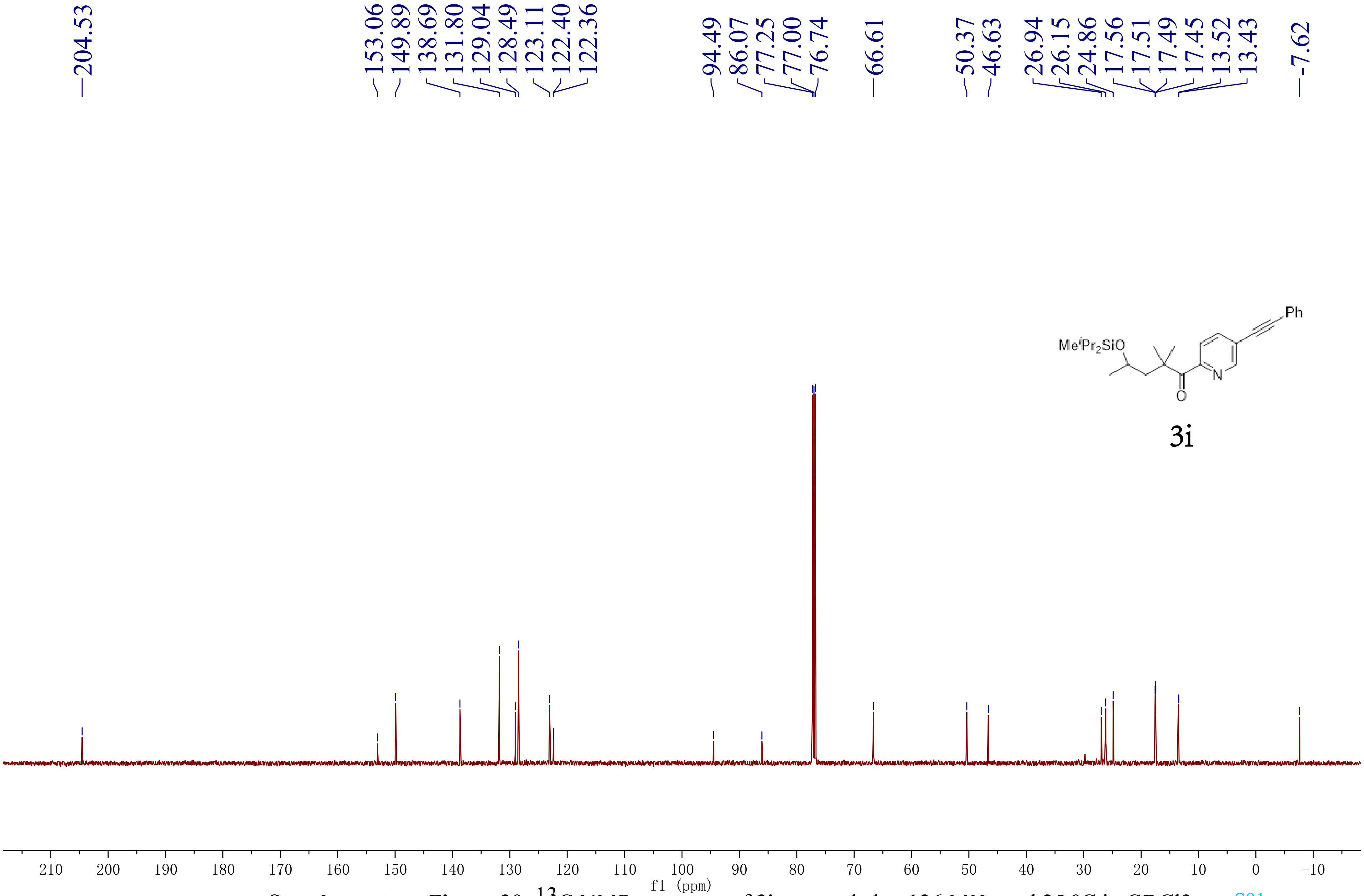

Supplementary Figure 20. ^{13}C NMR spectrum of **3i**, recorded at 126 MHz and 25 °C in CDCl3

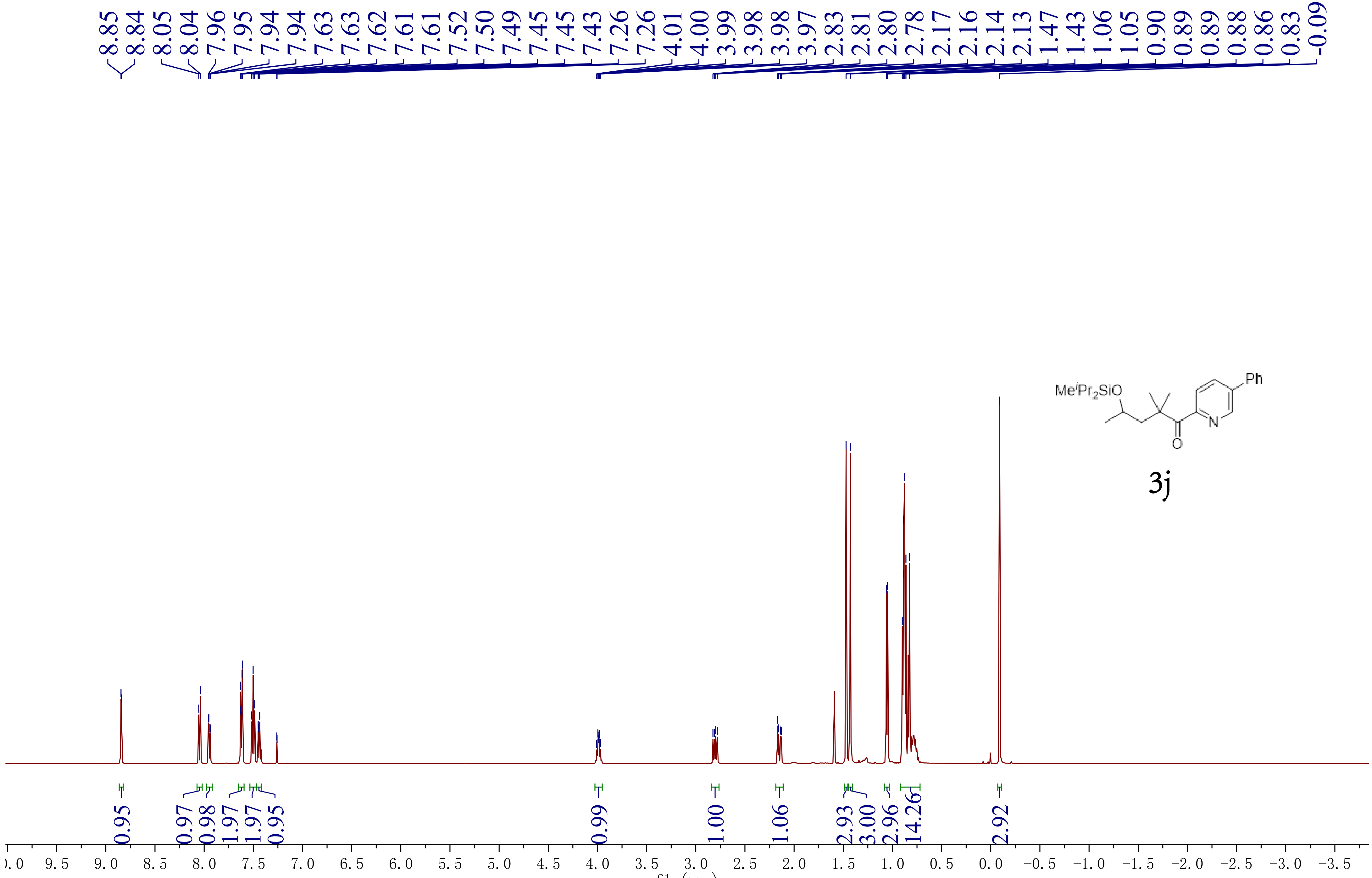

Supplementary Figure 21. ^{1}H NMR spectrum of **3j**, recorded at 500 MHz and 25 °C in $CDCl_3$

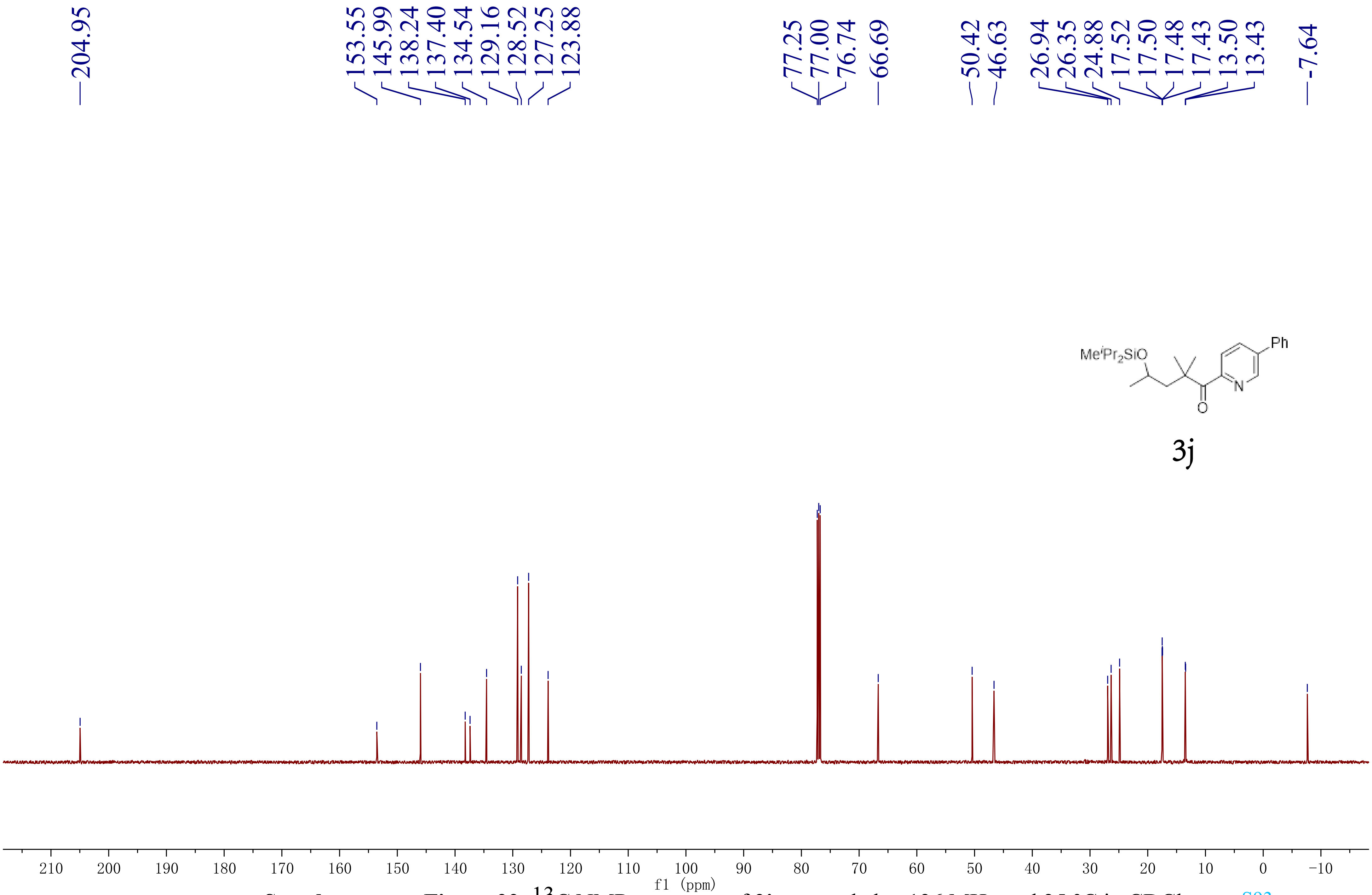

Supplementary Figure 22. ^{13}C NMR spectrum of **3j**, recorded at 126 MHz and 25 °C in $CDCl_3$

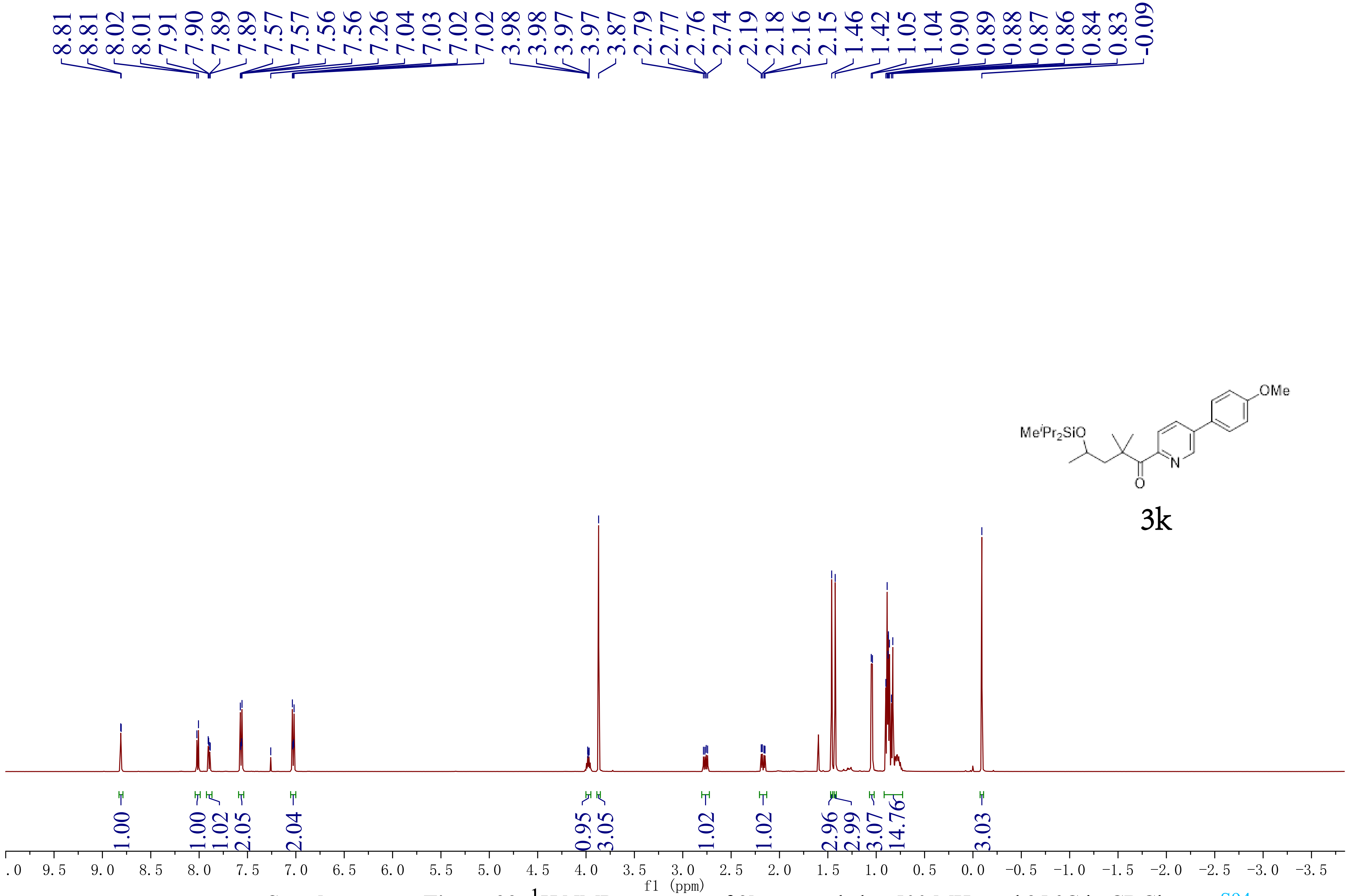

Supplementary Figure 23. ^{1}H NMR spectrum of **3k**, recorded at 500 MHz and 25 °C in $CDCl_3$

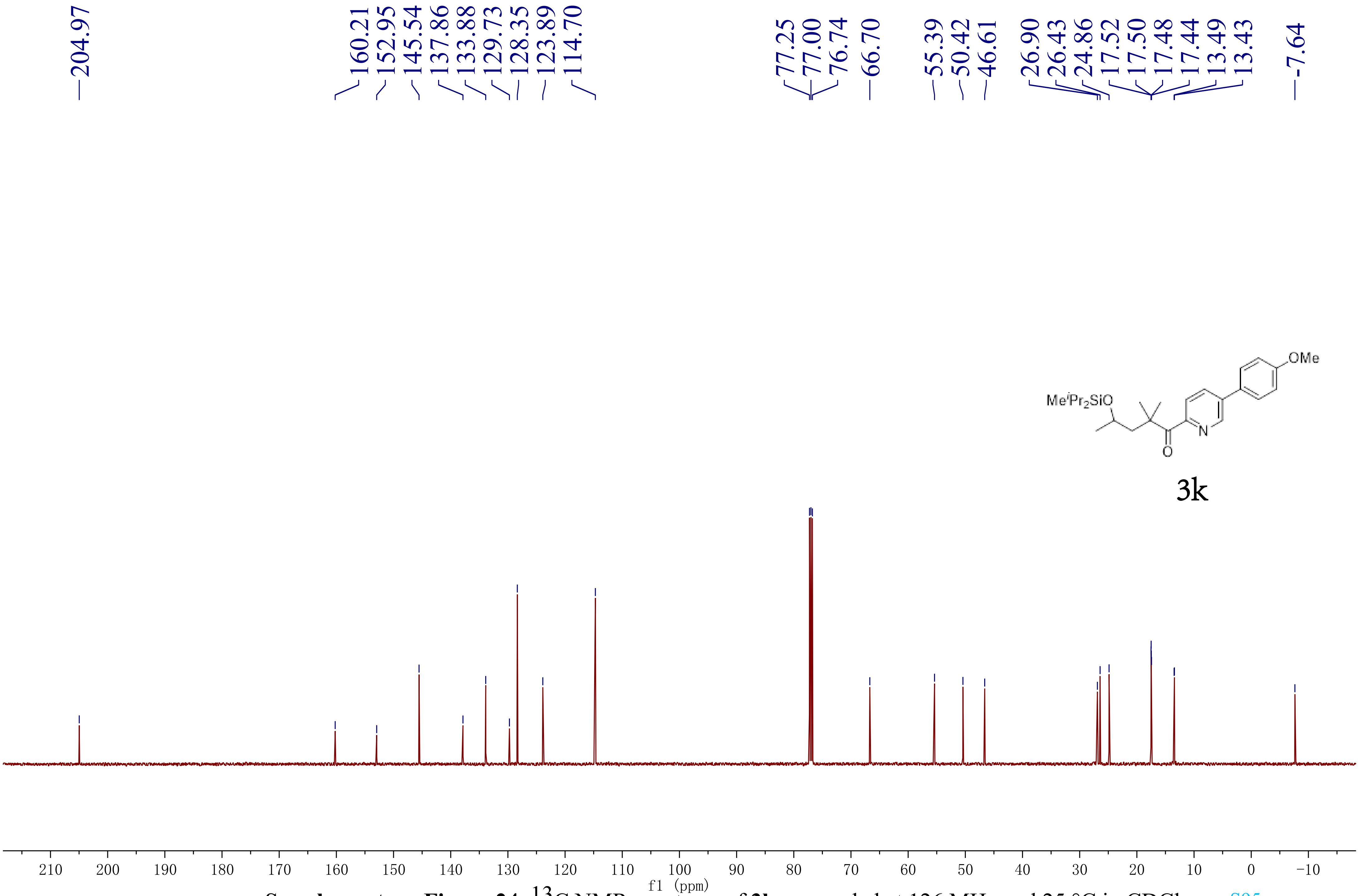

3k

Supplementary Figure 24. ^{13}C NMR spectrum of **3k**, recorded at 126 MHz and 25 °C in $CDCl_3$

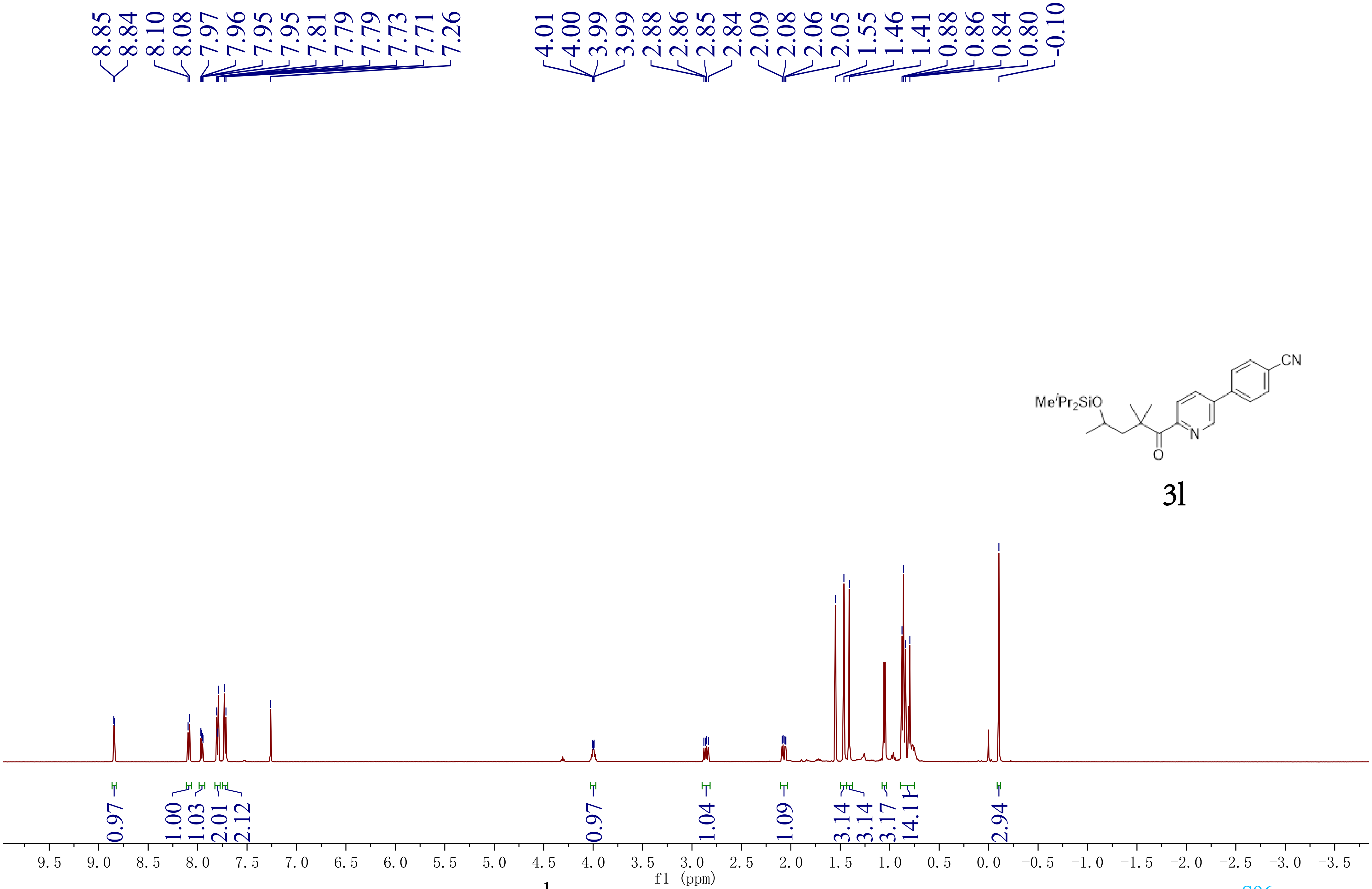

Supplementary Figure 25 . ^{1}H NMR spectrum of **3l**, recorded at 500 MHz and 25 °C in $CDCl_3$

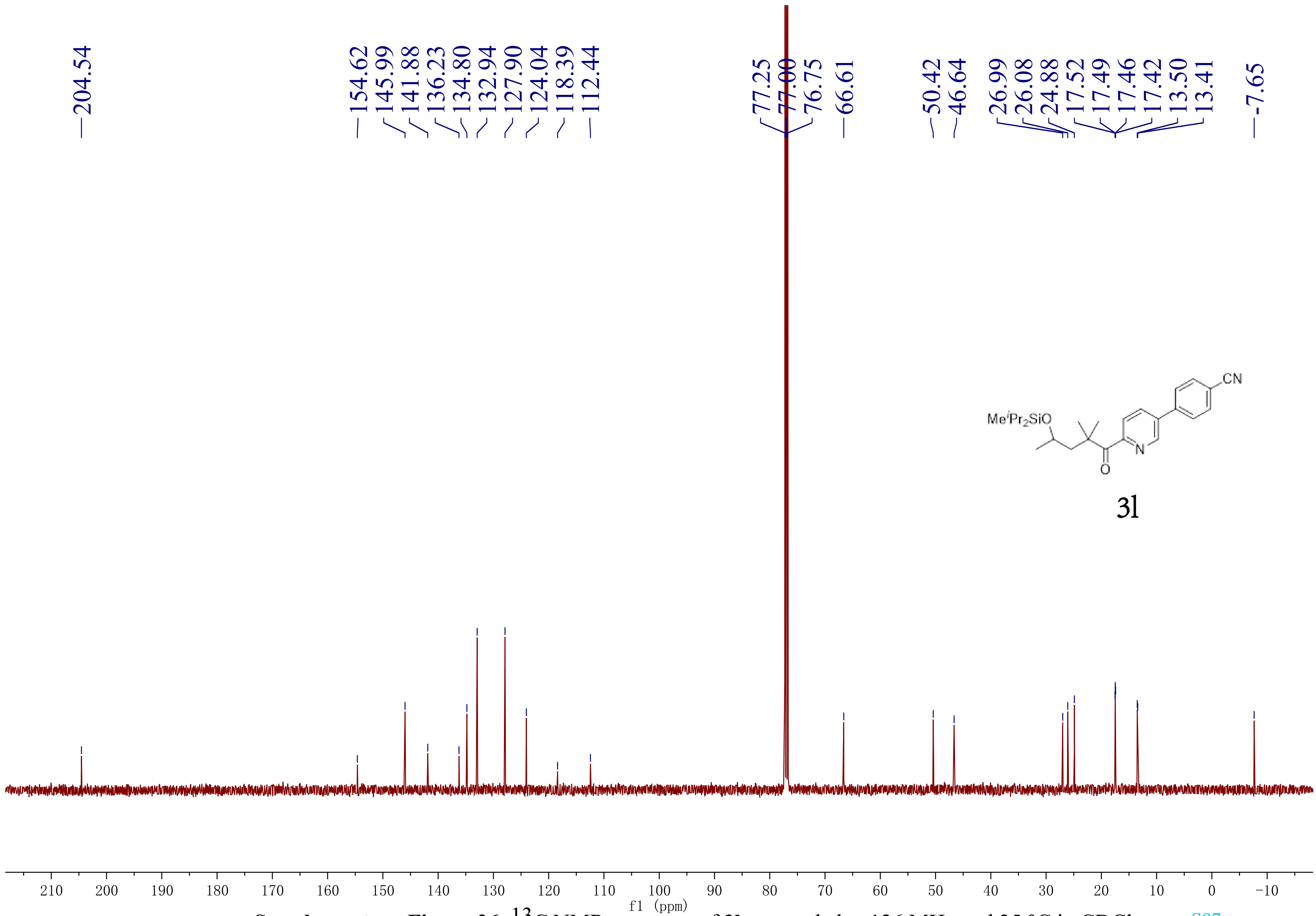

3l

Supplementary Figure 26. ^{13}C NMR spectrum of **3l**, recorded at 126 MHz and 25 °C in $CDCl_3$

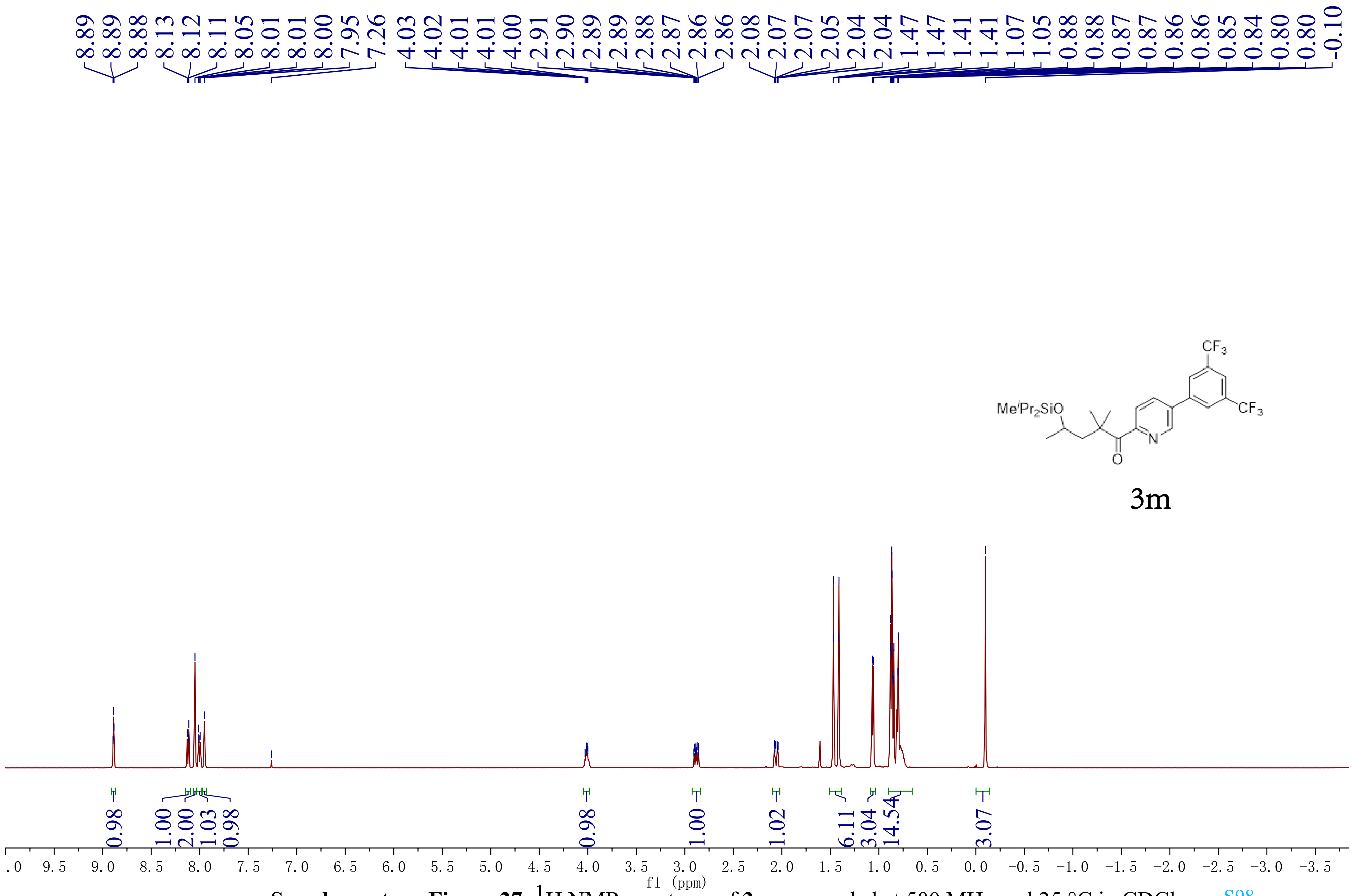

Supplementary Figure 27. ^{1}H NMR spectrum of **3m**, recorded at 500 MHz and 25 °C in $CDCl_3$

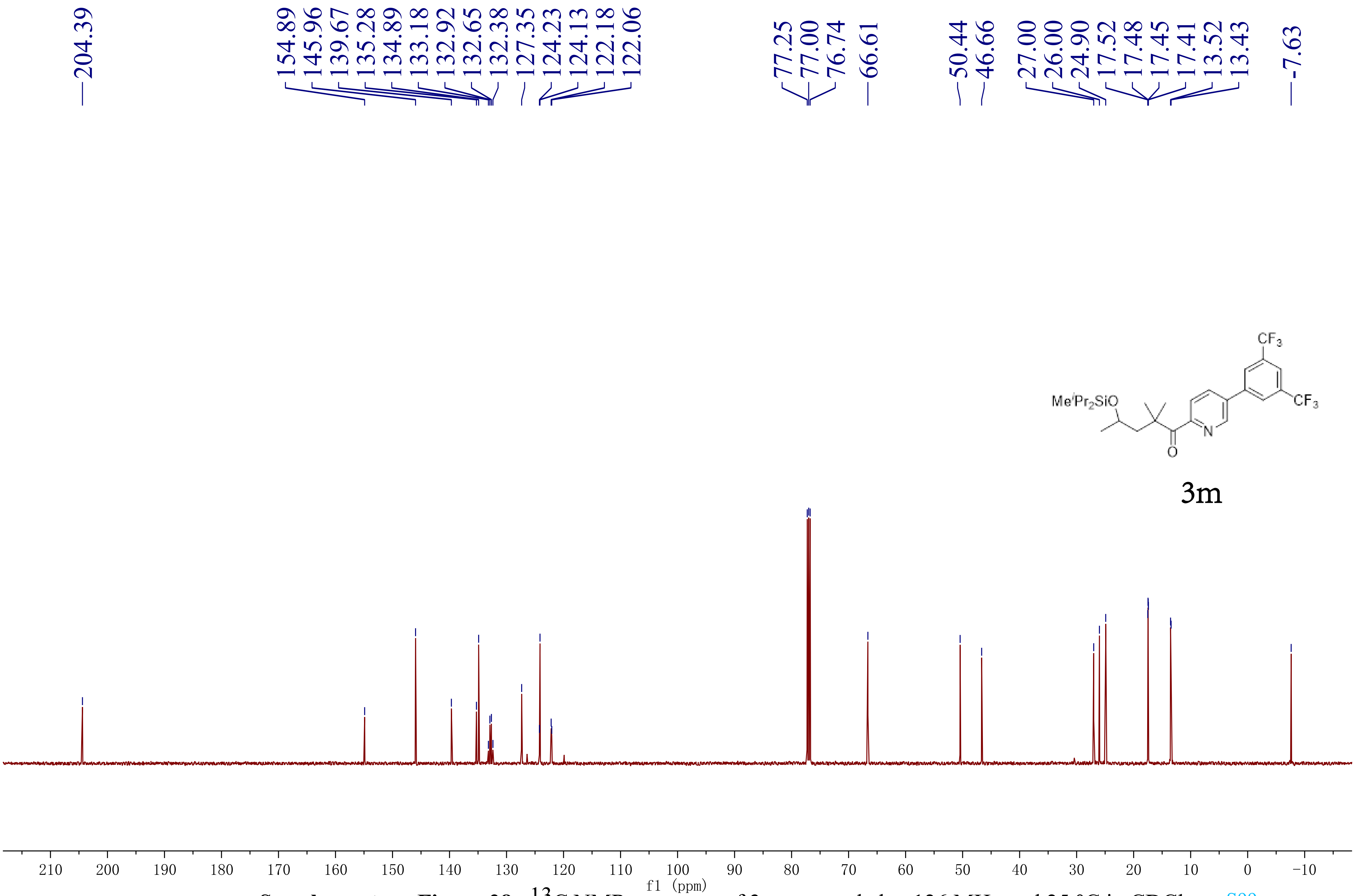

Supplementary Figure 28. ^{13}C NMR spectrum of **3m**, recorded at 126 MHz and 25 °C in $CDCl_3$

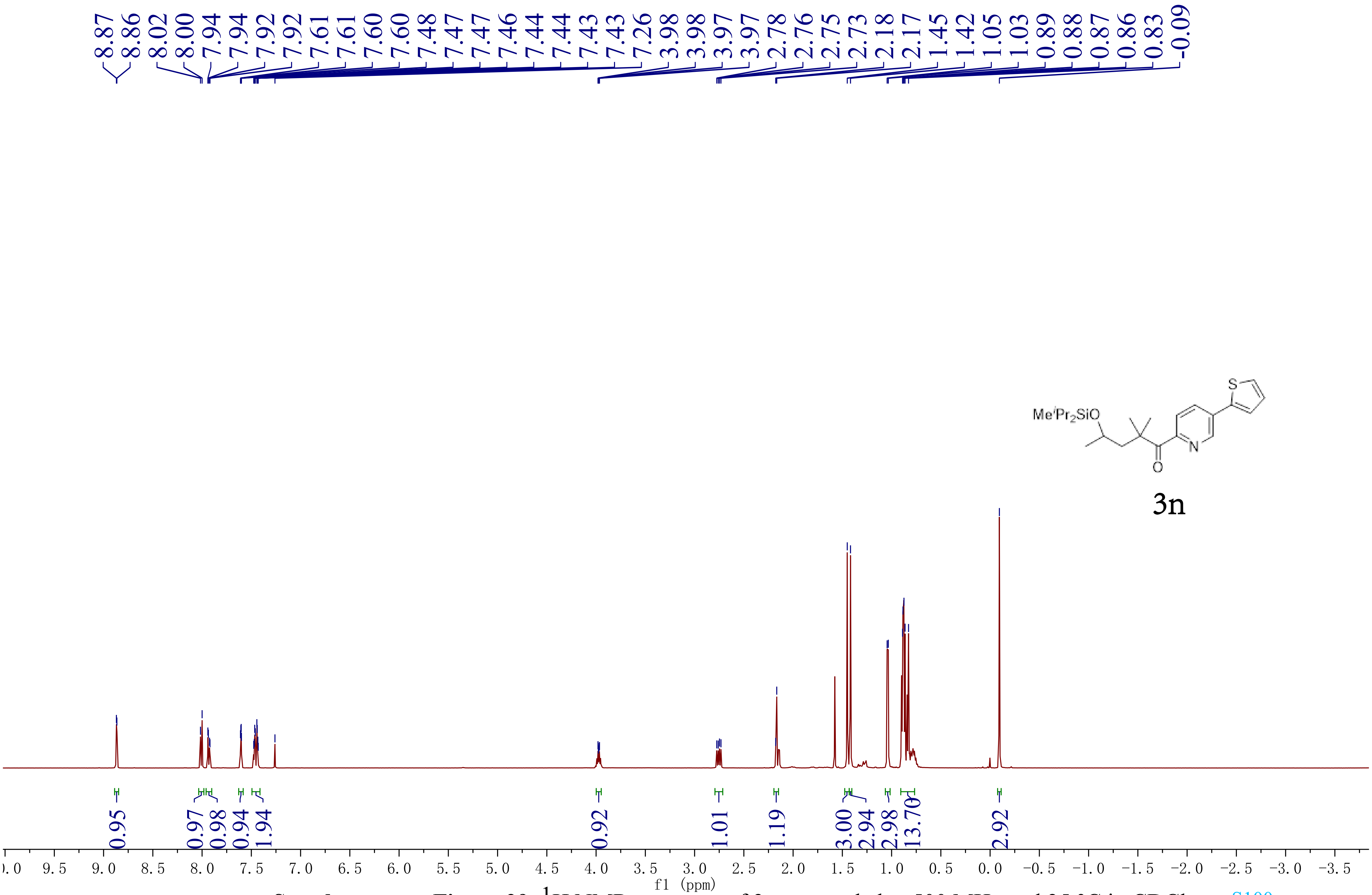

Supplementary Figure 29. ^{1}H NMR spectrum of **3n**, recorded at 500 MHz and 25 °C in $CDCl_3$

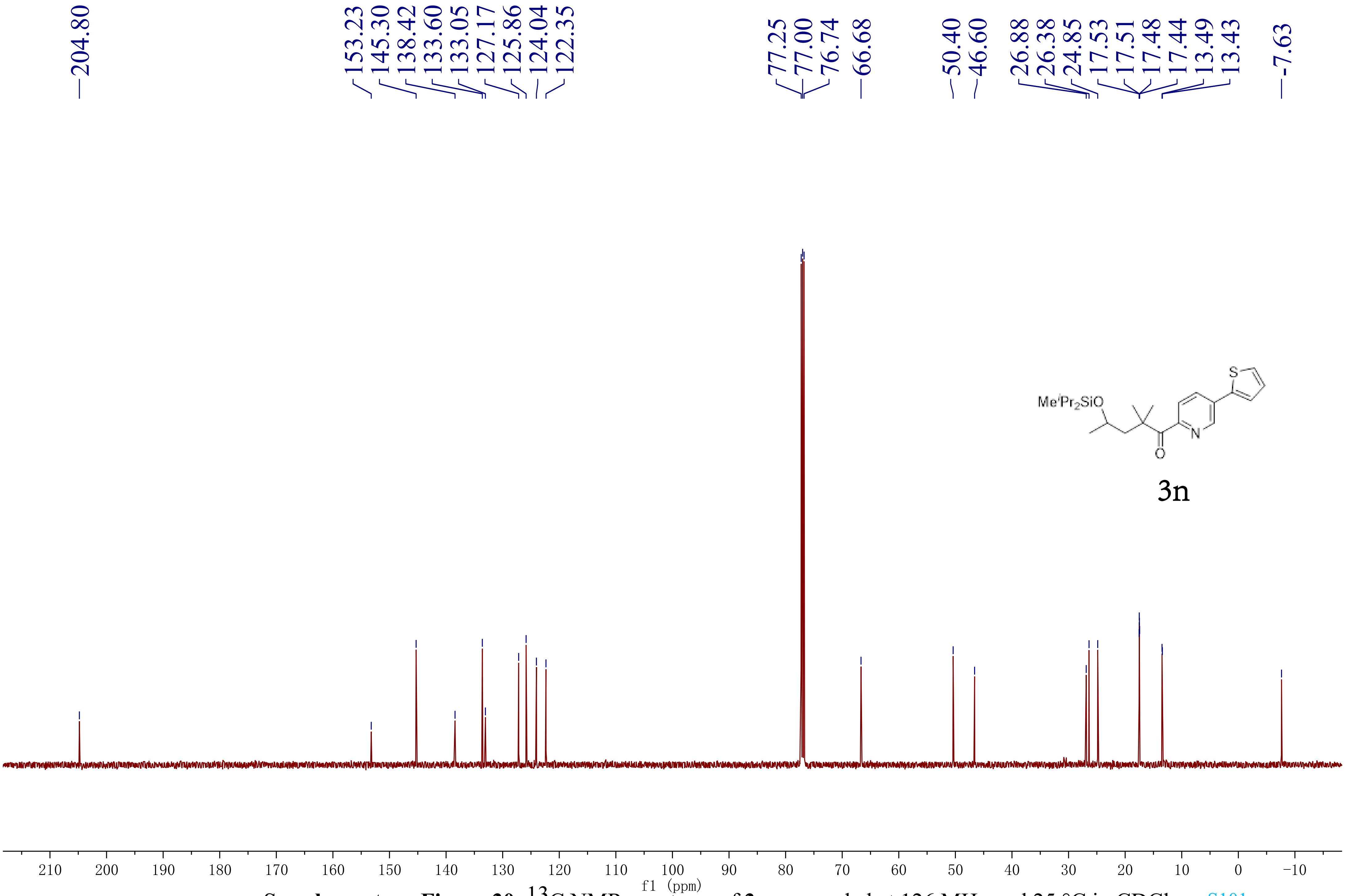

Supplementary Figure 30. ^{13}C NMR spectrum of **3n**, recorded at 126 MHz and 25 °C in $CDCl_3$

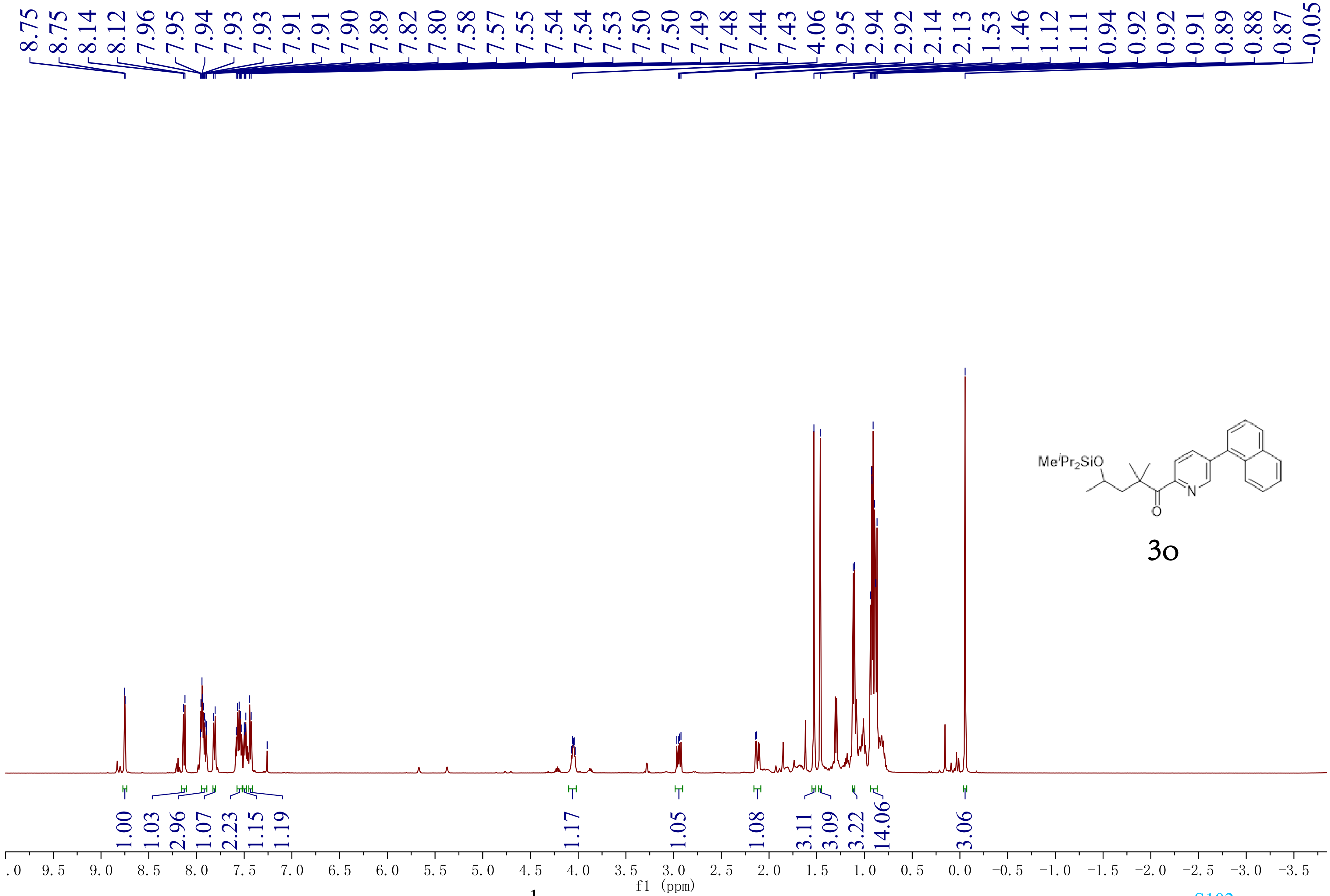

Supplementary Figure 31. ^{1}H NMR spectrum of **3o**, recorded at 500 MHz and 25 °C in $CDCl_3$

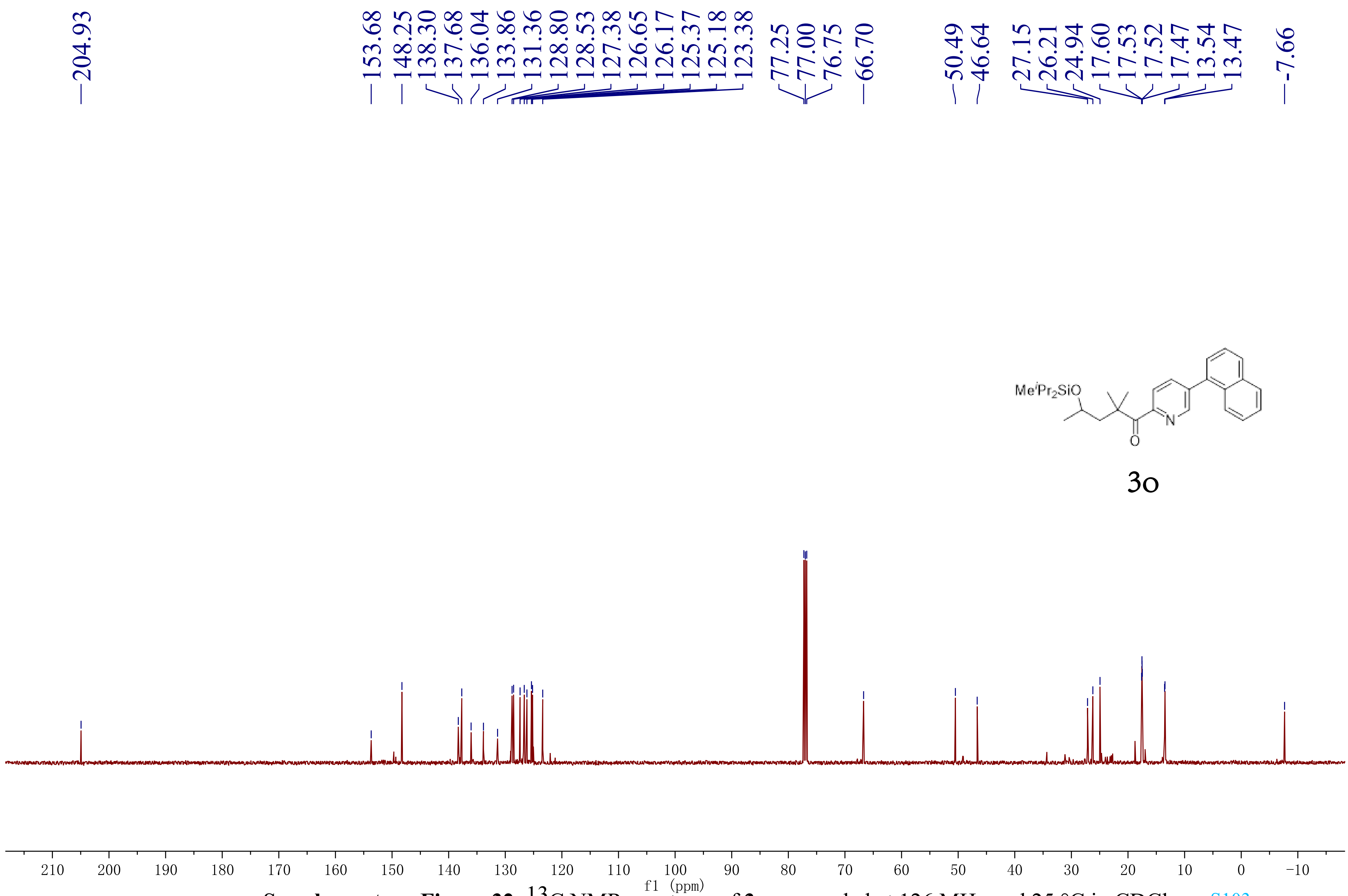

Supplementary Figure 32. ^{13}C NMR spectrum of **3o**, recorded at 126 MHz and 25 °C in $CDCl_3$

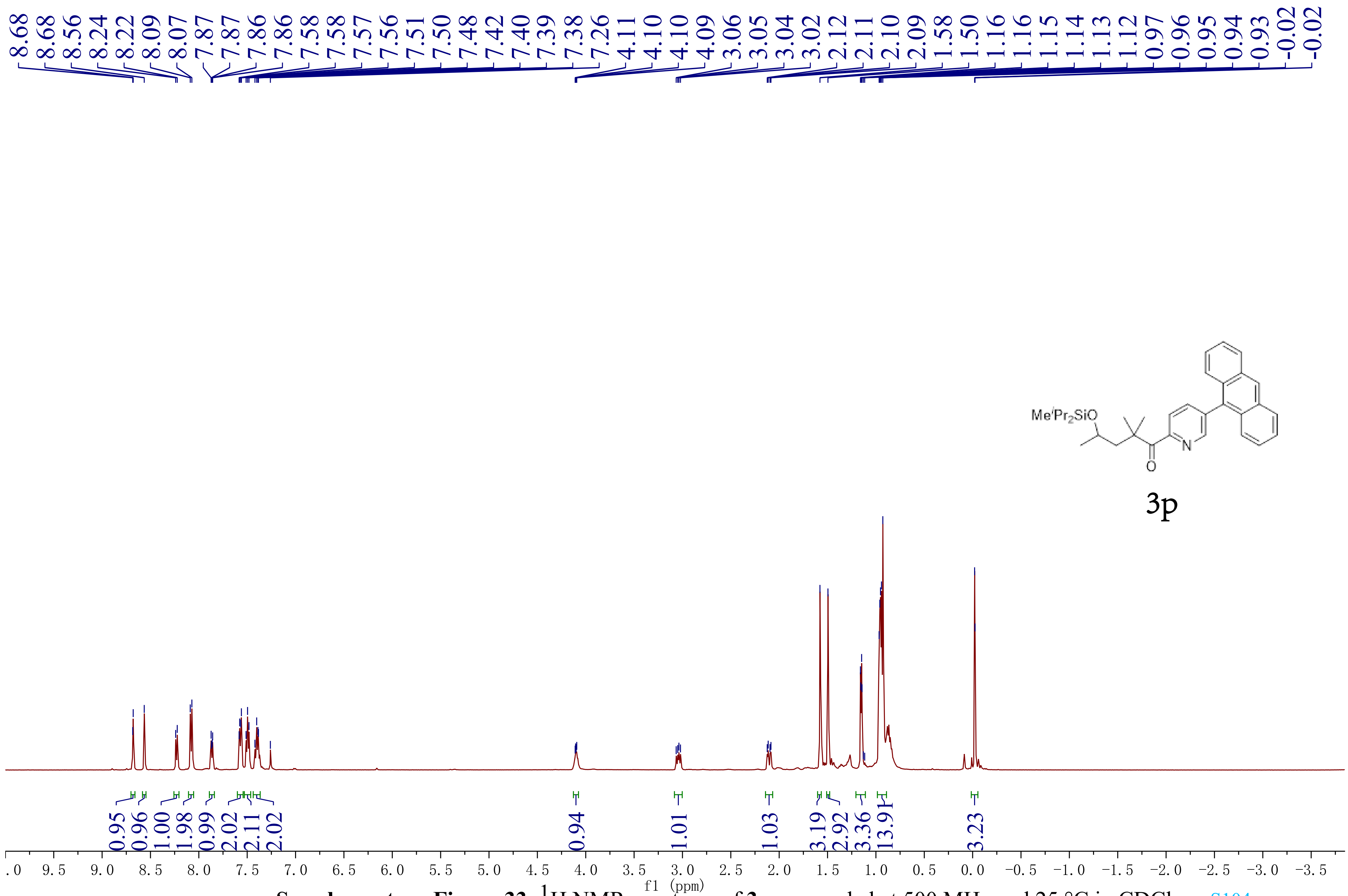

Supplementary Figure 33. ^{1}H NMR spectrum of **3p**, recorded at 500 MHz and 25 °C in $CDCl_3$

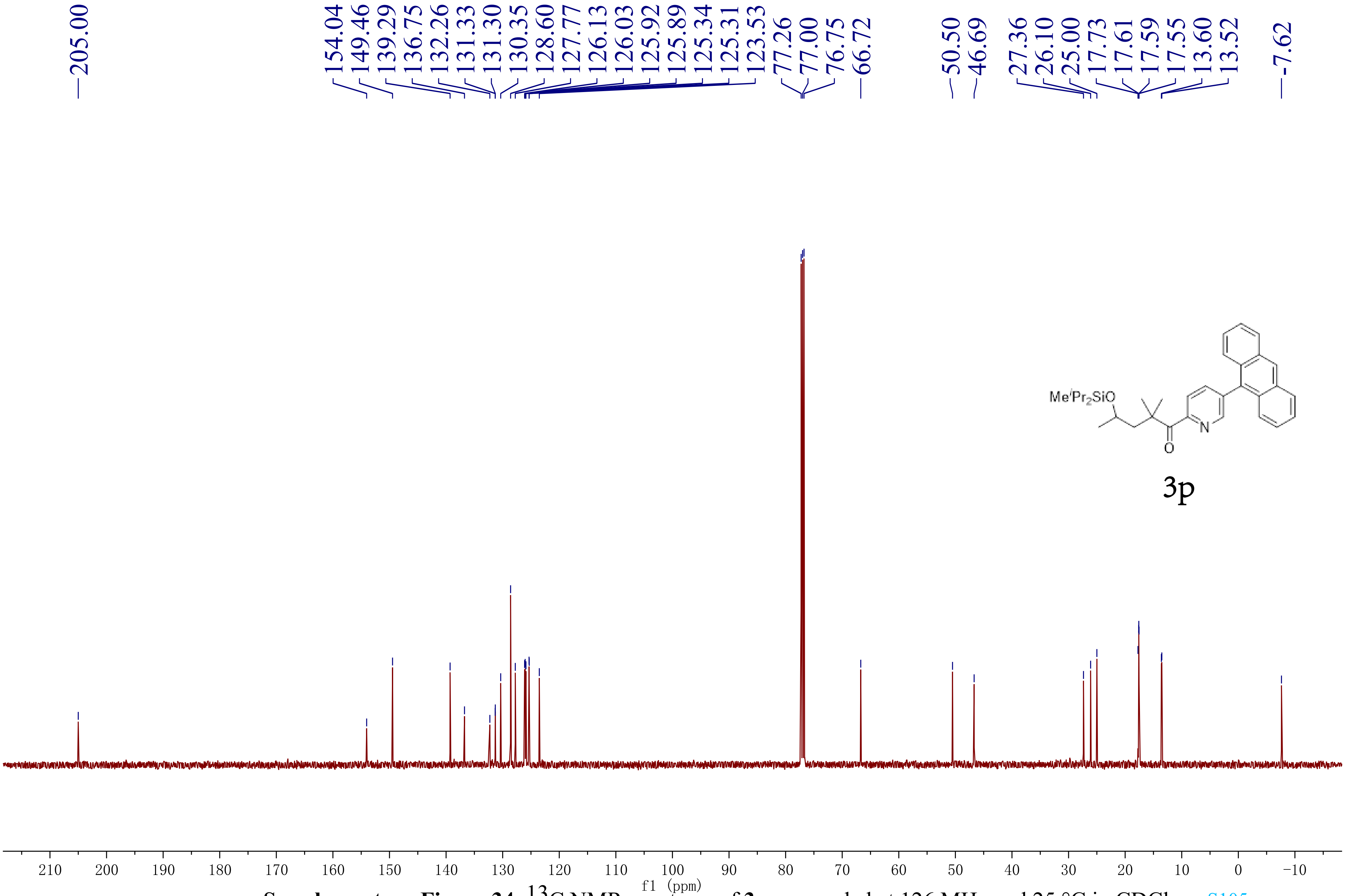

3p

Supplementary Figure 34. ^{13}C NMR spectrum of **3p**, recorded at 126 MHz and 25 °C in $CDCl_3$

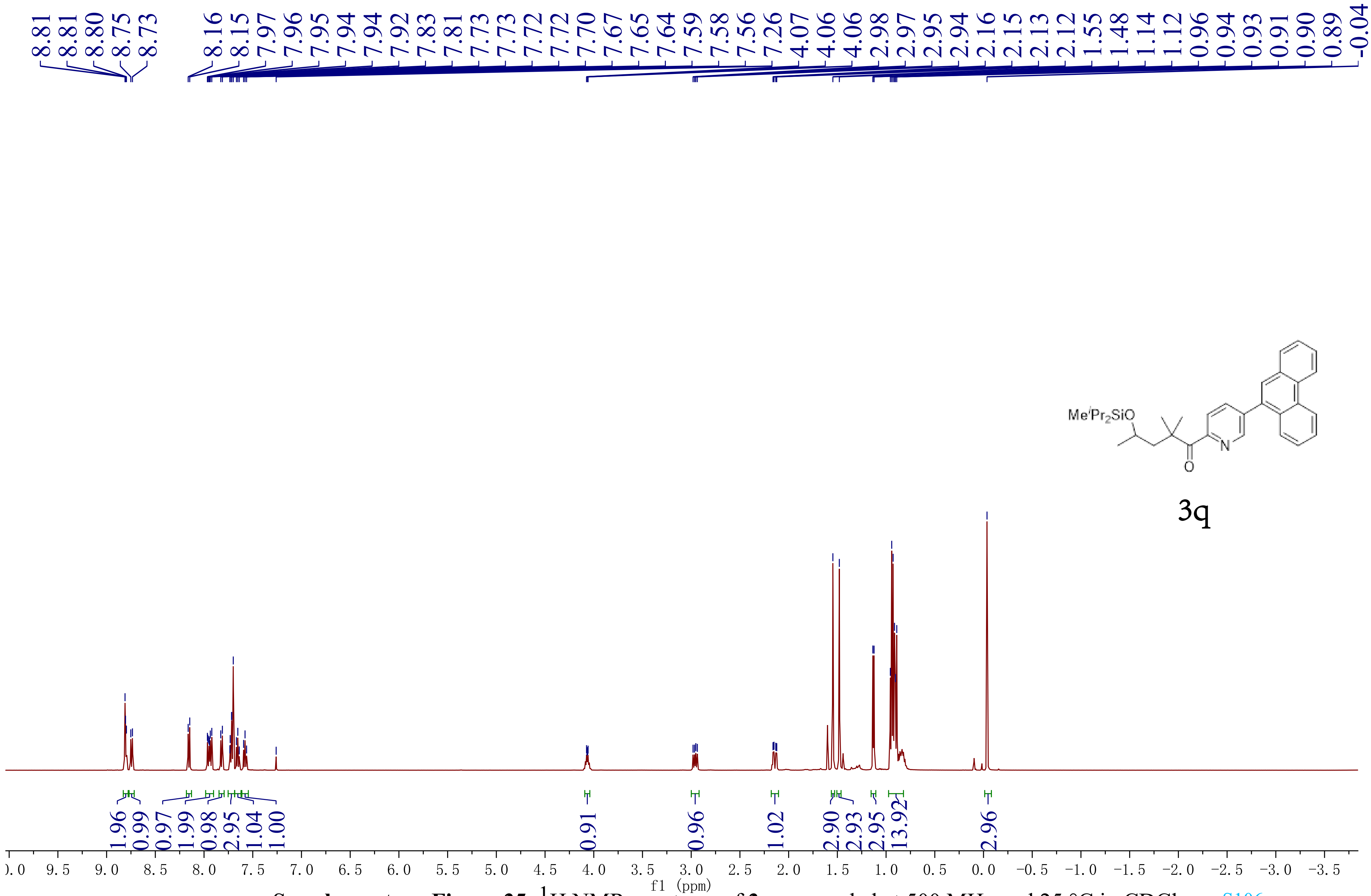

Supplementary Figure 35. ^{1}H NMR spectrum of **3q**, recorded at 500 MHz and 25 °C in $CDCl_3$

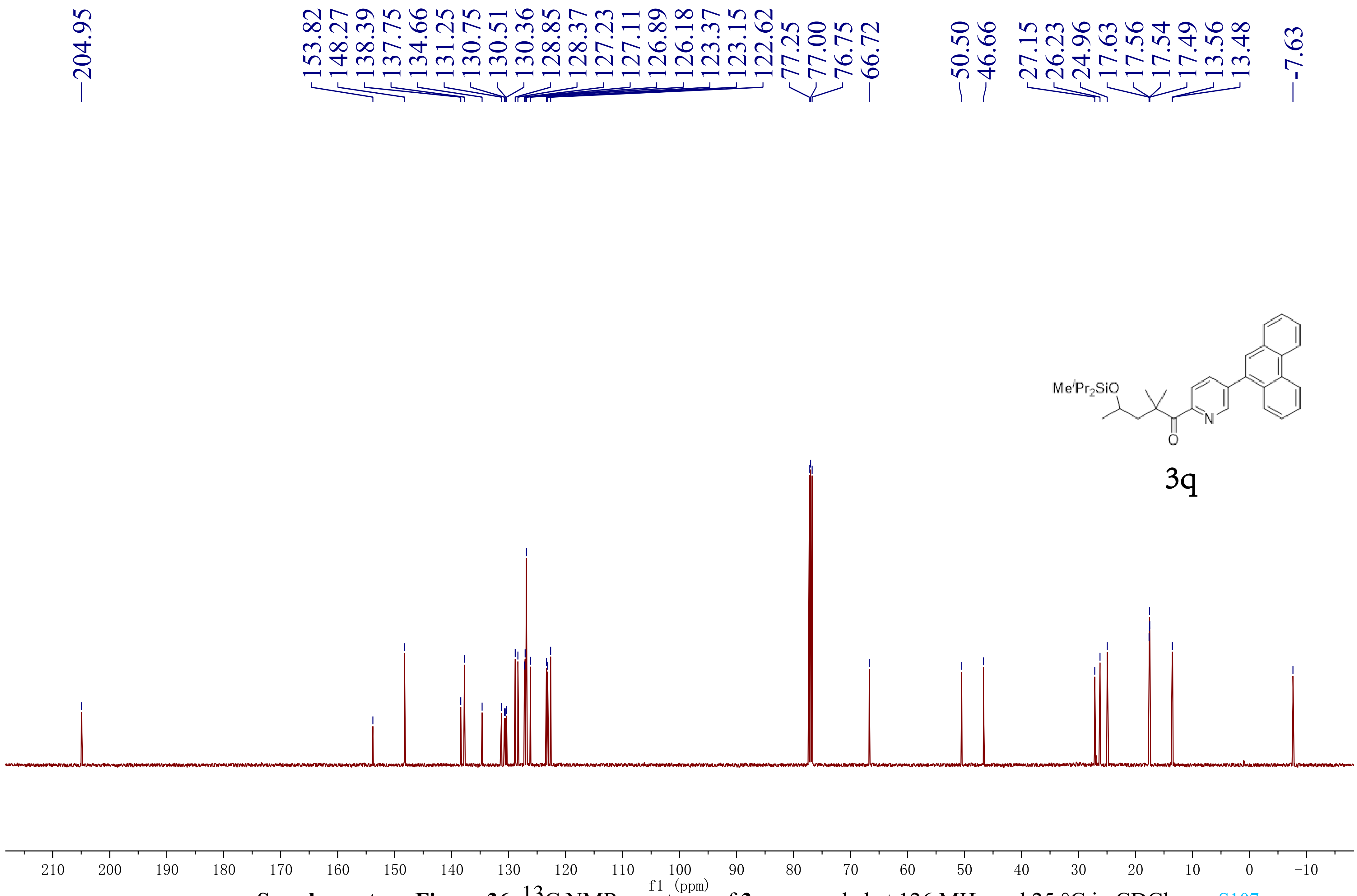

Supplementary Figure 36. ^{13}C NMR spectrum of **3q**, recorded at 126 MHz and 25 °C in $CDCl_3$

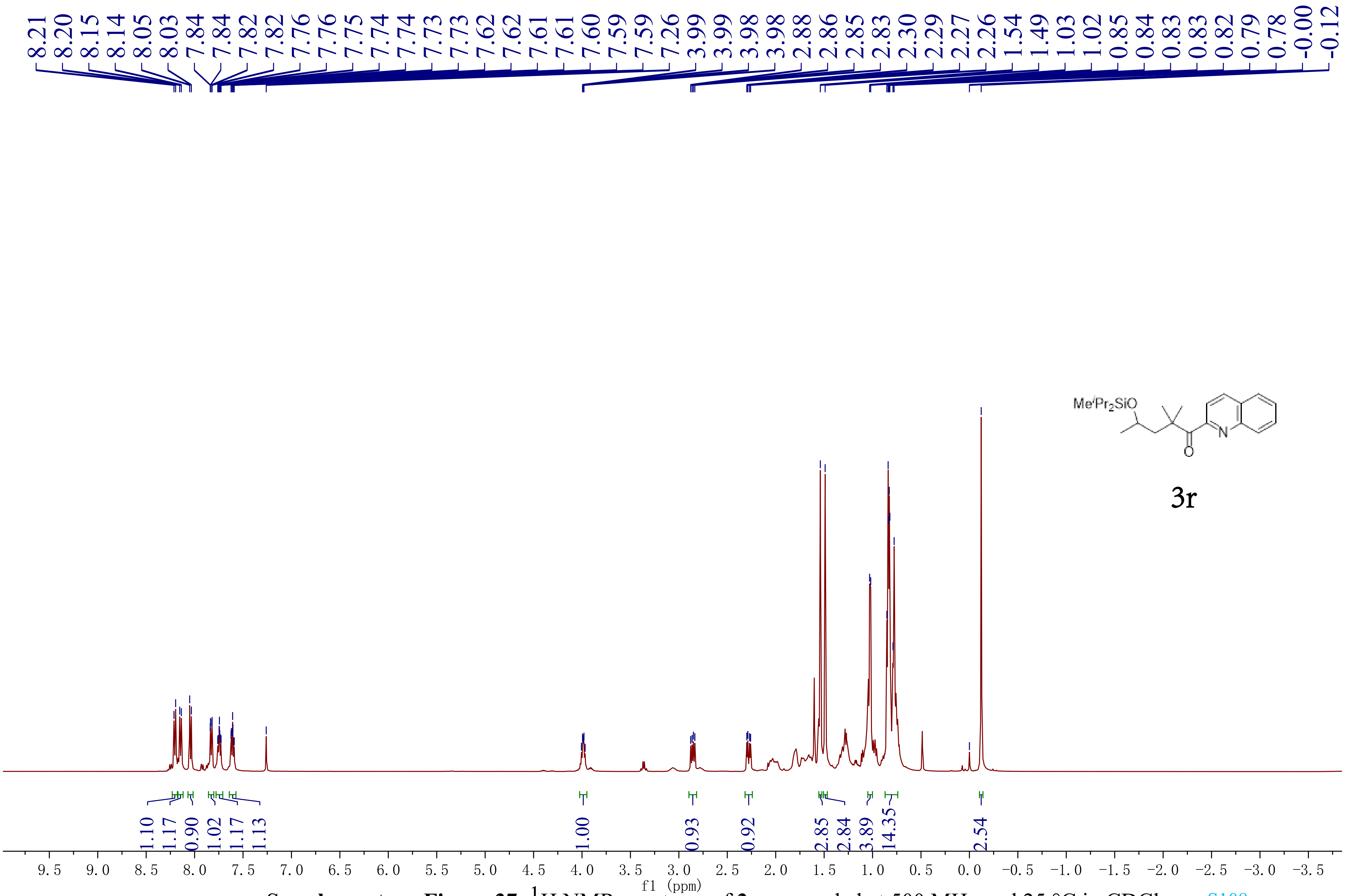

Supplementary Figure 37. ^{1}H NMR spectrum of **3r**, recorded at 500 MHz and 25 °C in $CDCl_3$

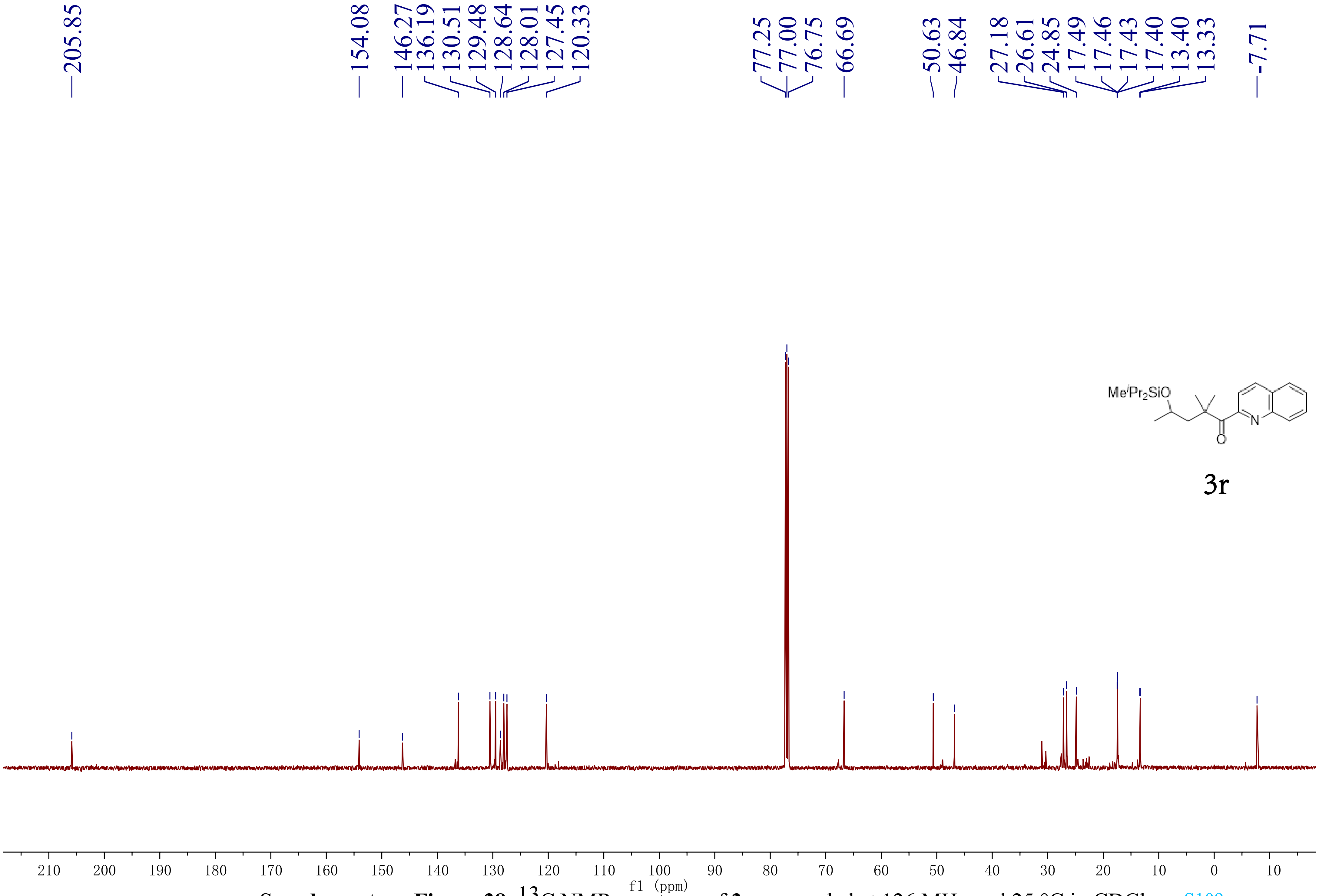

3r

Supplementary Figure 38. ^{13}C NMR spectrum of **3r**, recorded at 126 MHz and 25 °C in $CDCl_3$

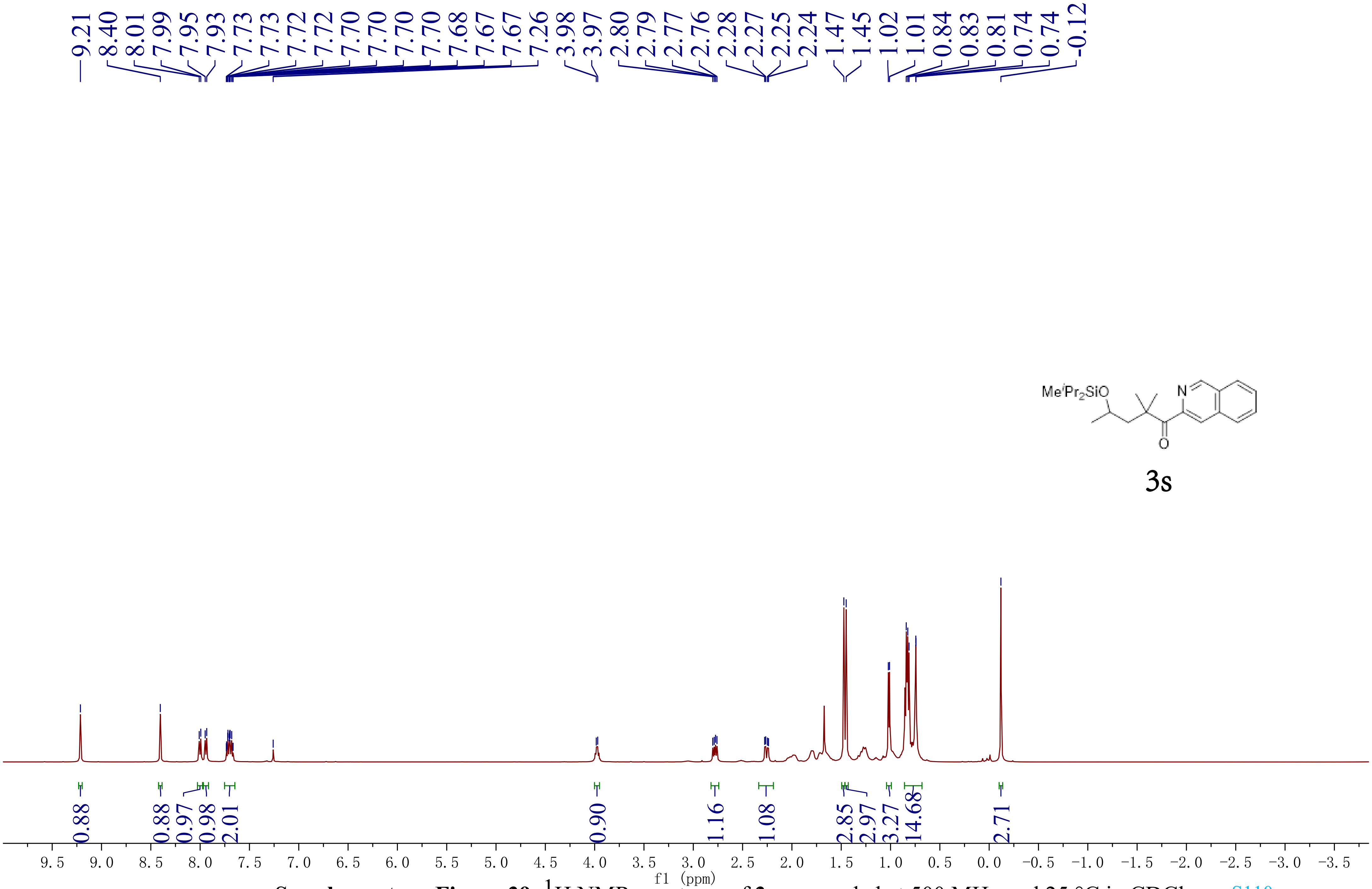

3s

Supplementary Figure 39. ^{1}H NMR spectrum of **3s**, recorded at 500 MHz and 25 °C in $CDCl_3$

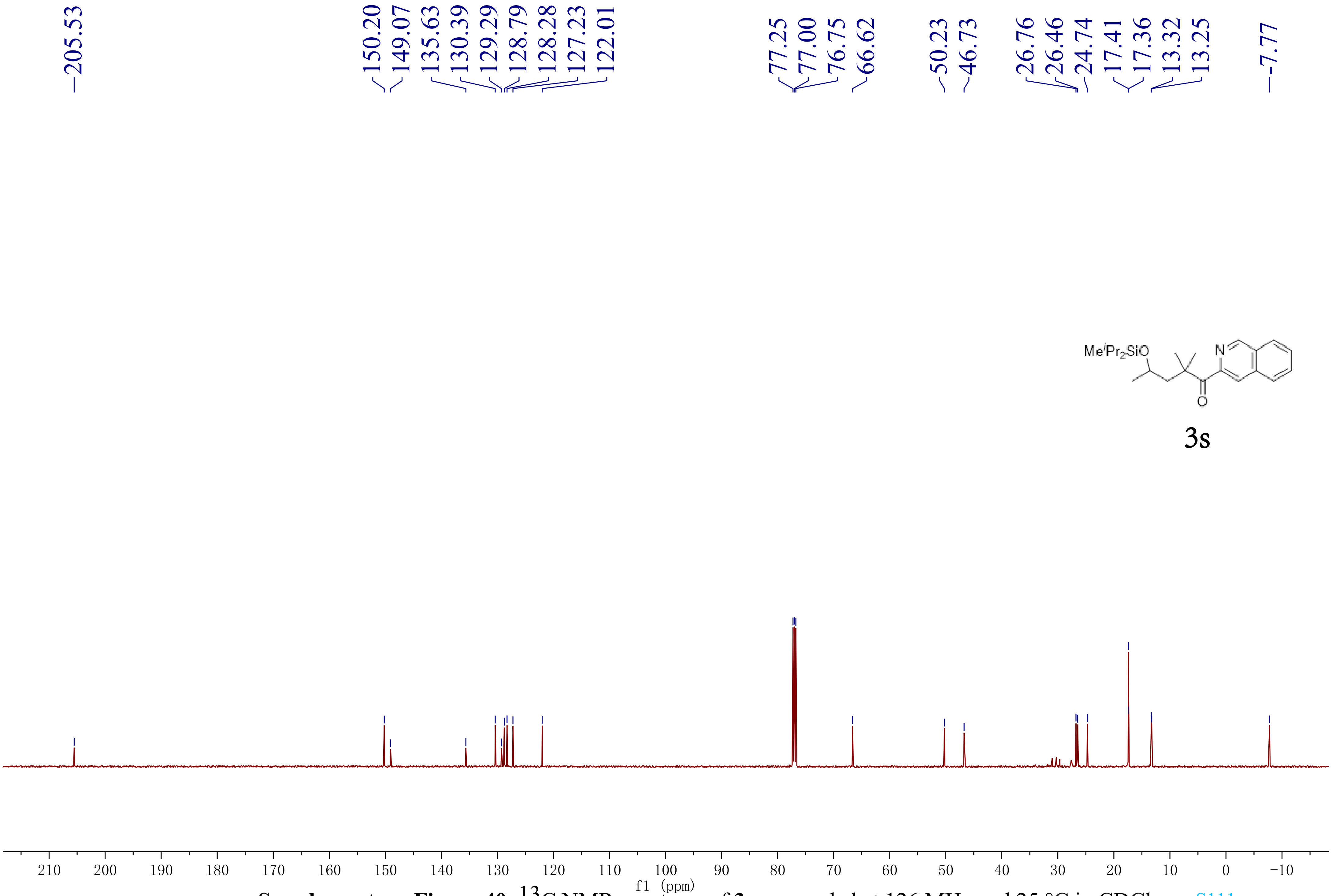

Supplementary Figure 40. ^{13}C NMR spectrum of **3s**, recorded at 126 MHz and 25 °C in $CDCl_3$

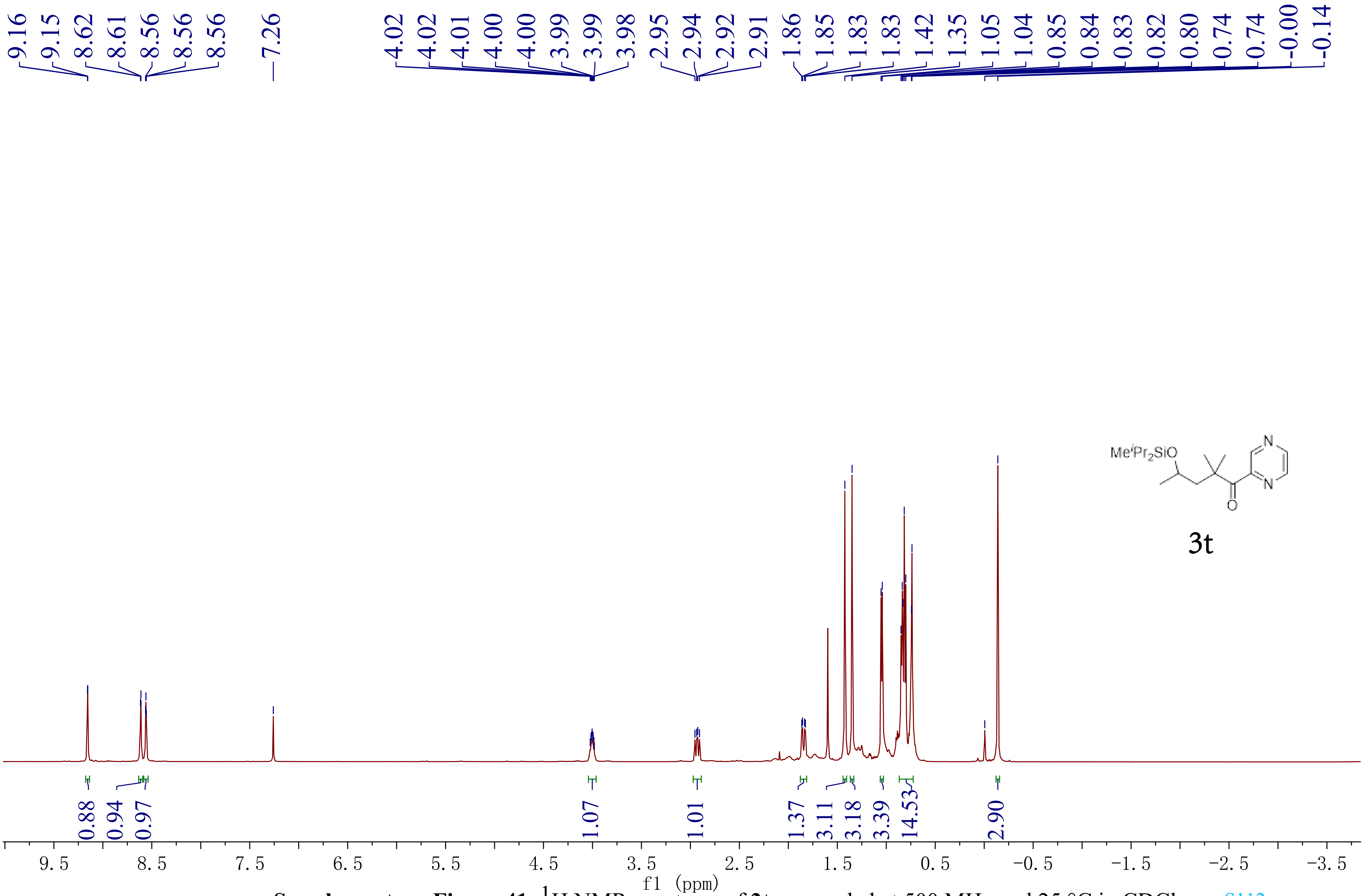

Supplementary Figure 41. ^{1}H NMR spectrum of **3t**, recorded at 500 MHz and 25 °C in $CDCl_3$

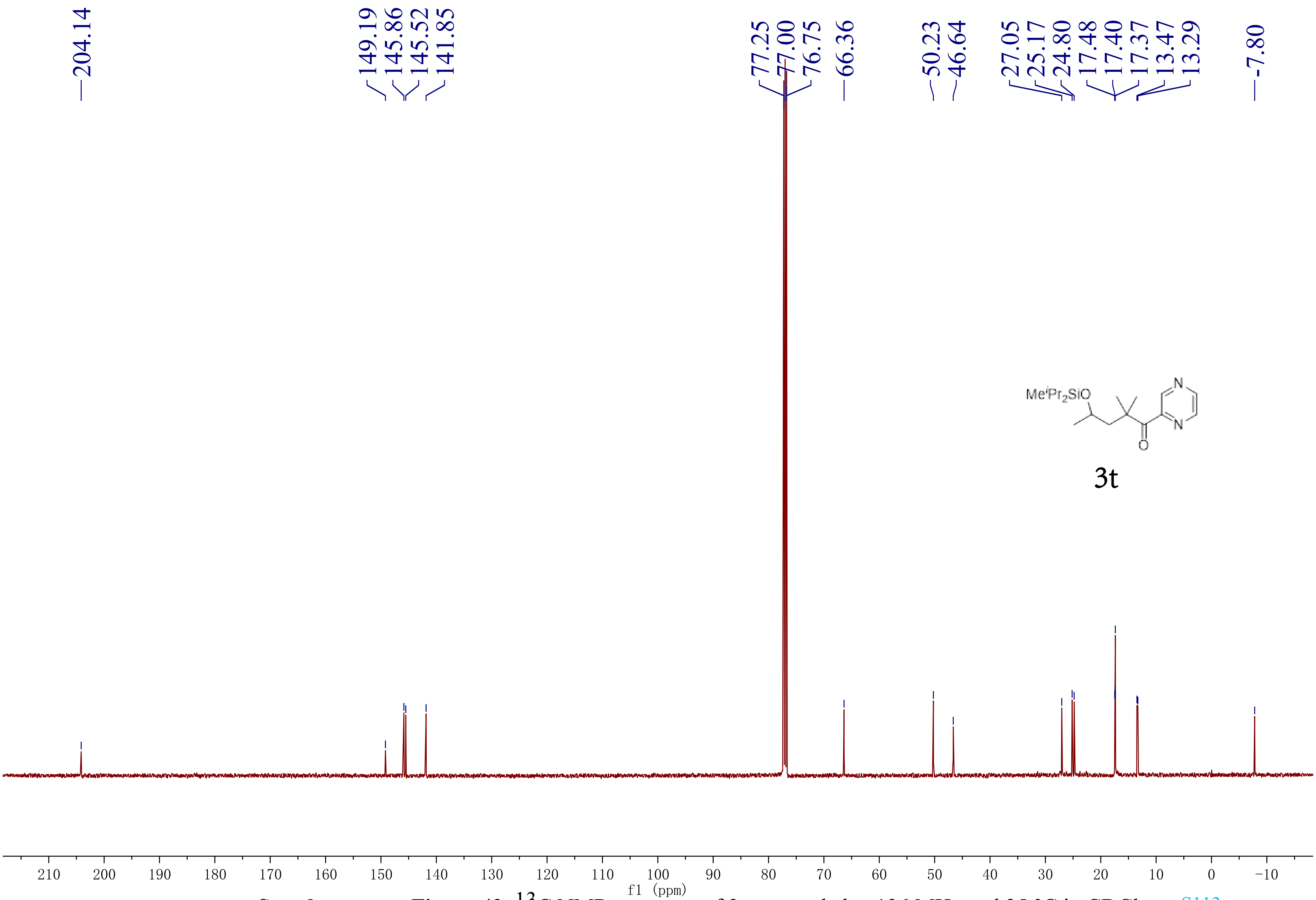

3t

Supplementary Figure 42. ^{13}C NMR spectrum of **3t**, recorded at 126 MHz and 25 °C in $CDCl_3$

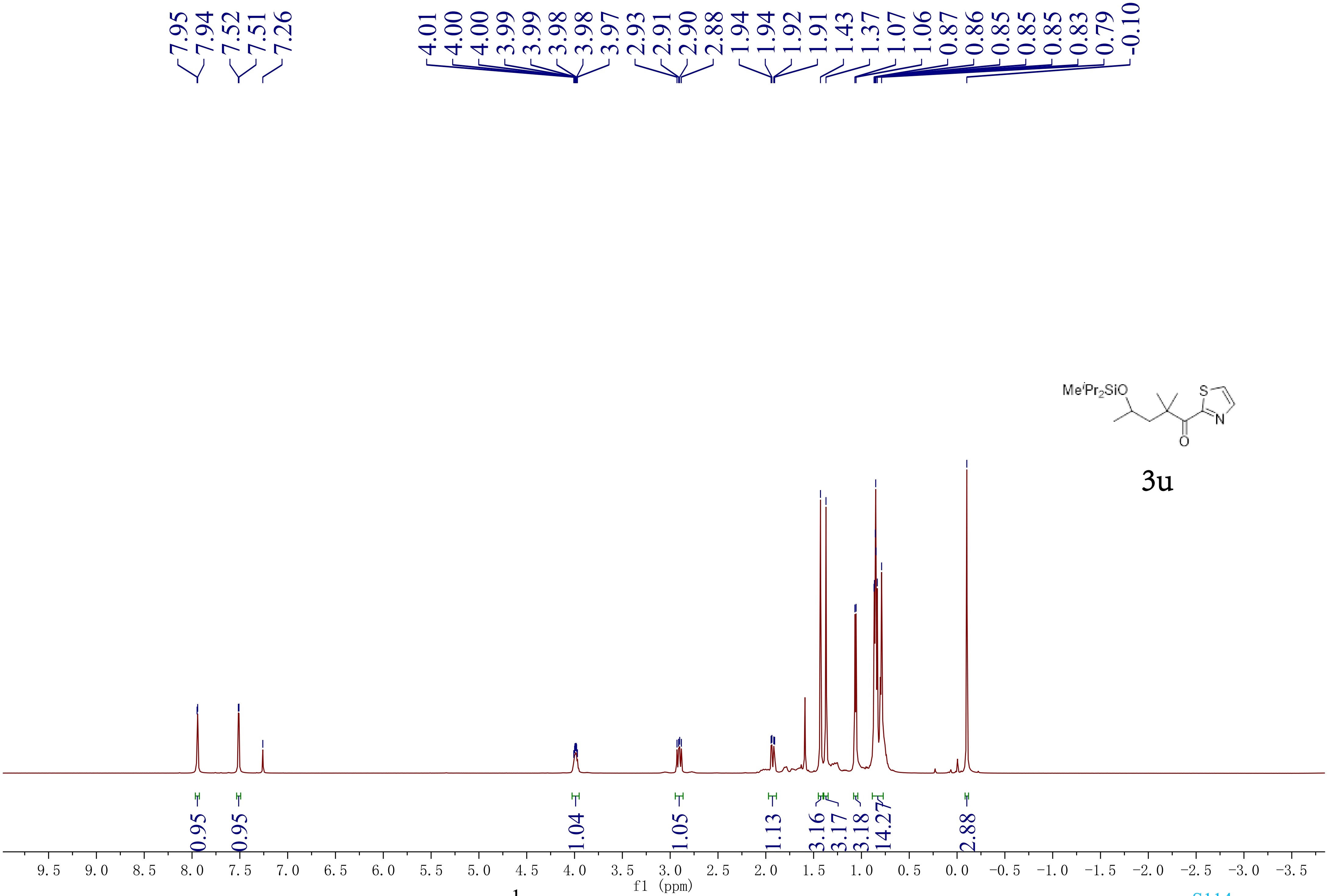

Supplementary Figure 43. ^{1}H NMR spectrum of **3u**, recorded at 500 MHz and 25 °C in $CDCl_3$

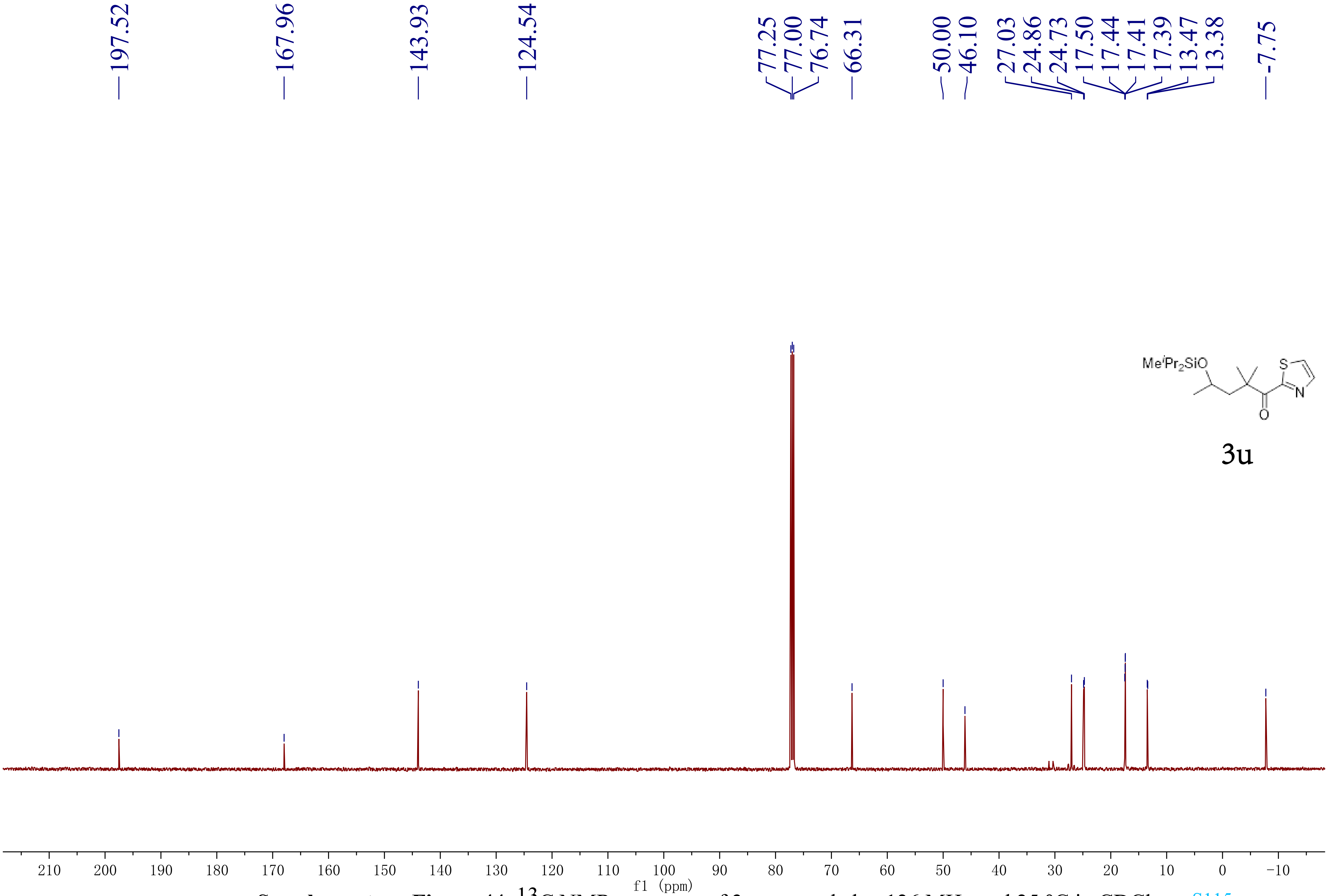

Supplementary Figure 44. ^{13}C NMR spectrum of **3u**, recorded at 126 MHz and 25 °C in $CDCl_3$

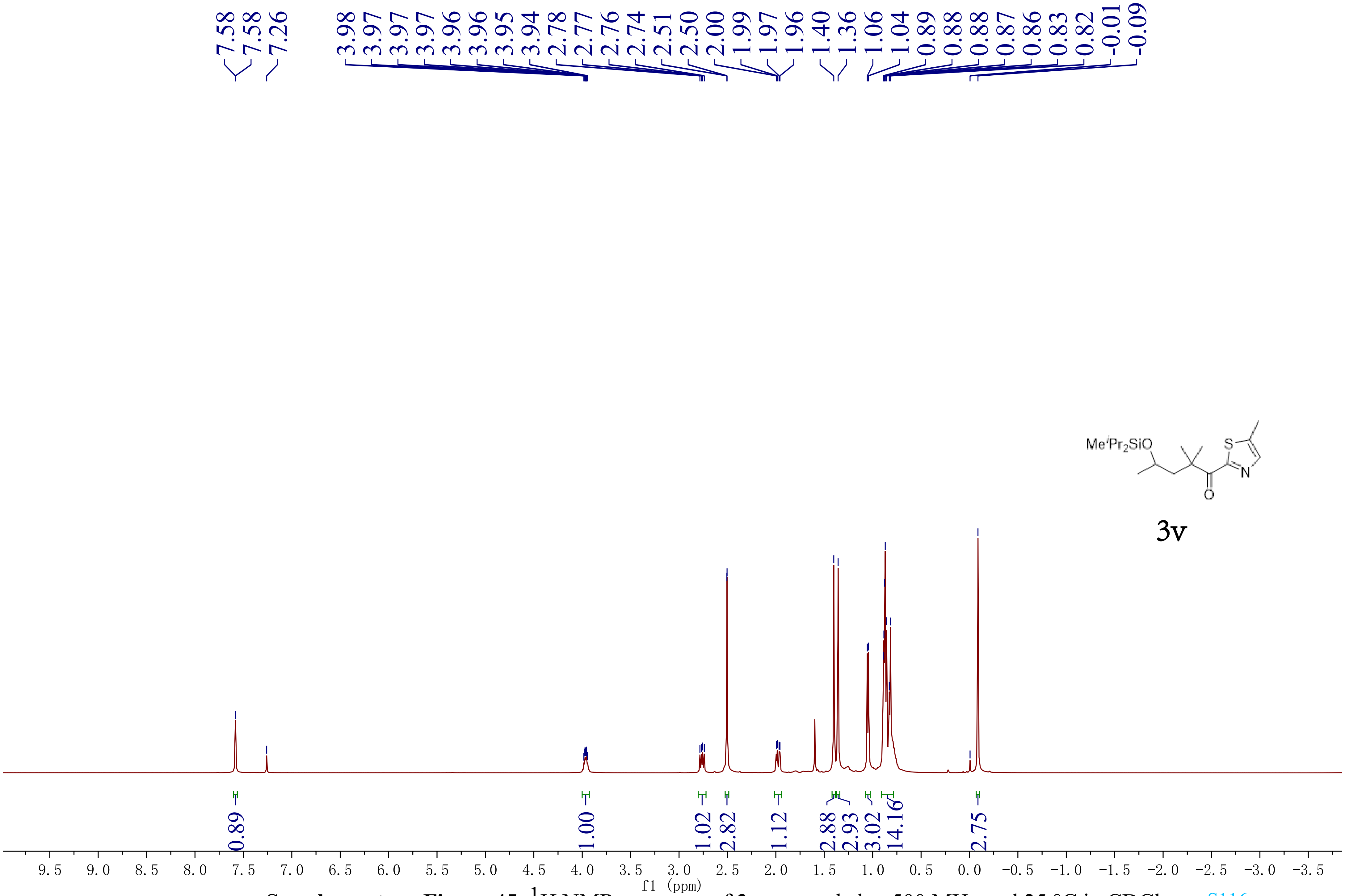

Supplementary Figure 45. ^{1}H NMR spectrum of **3v**, recorded at 500 MHz and 25 °C in $CDCl_3$

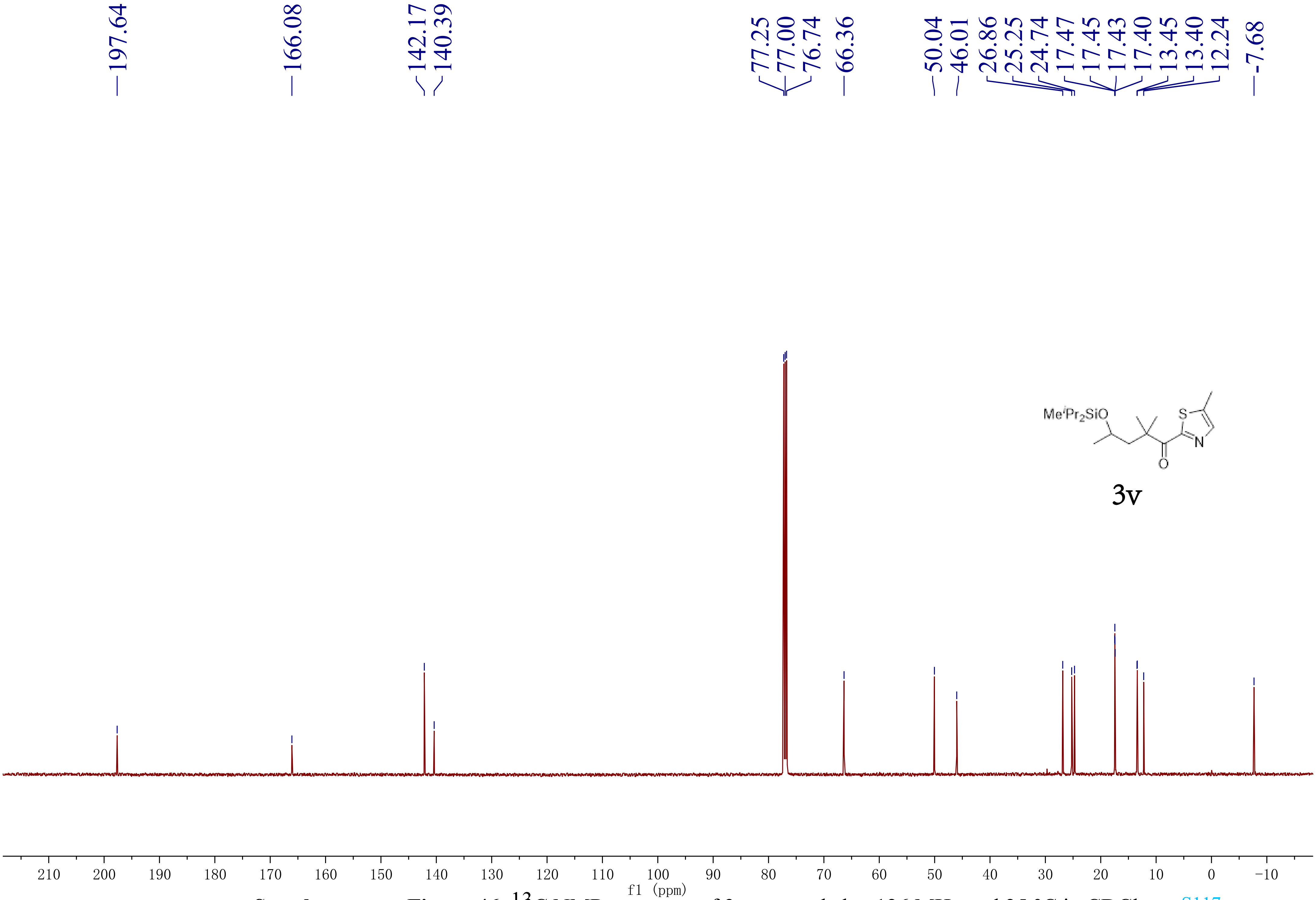

Supplementary Figure 46. ^{13}C NMR spectrum of **3v**, recorded at 126 MHz and 25 °C in $CDCl_3$

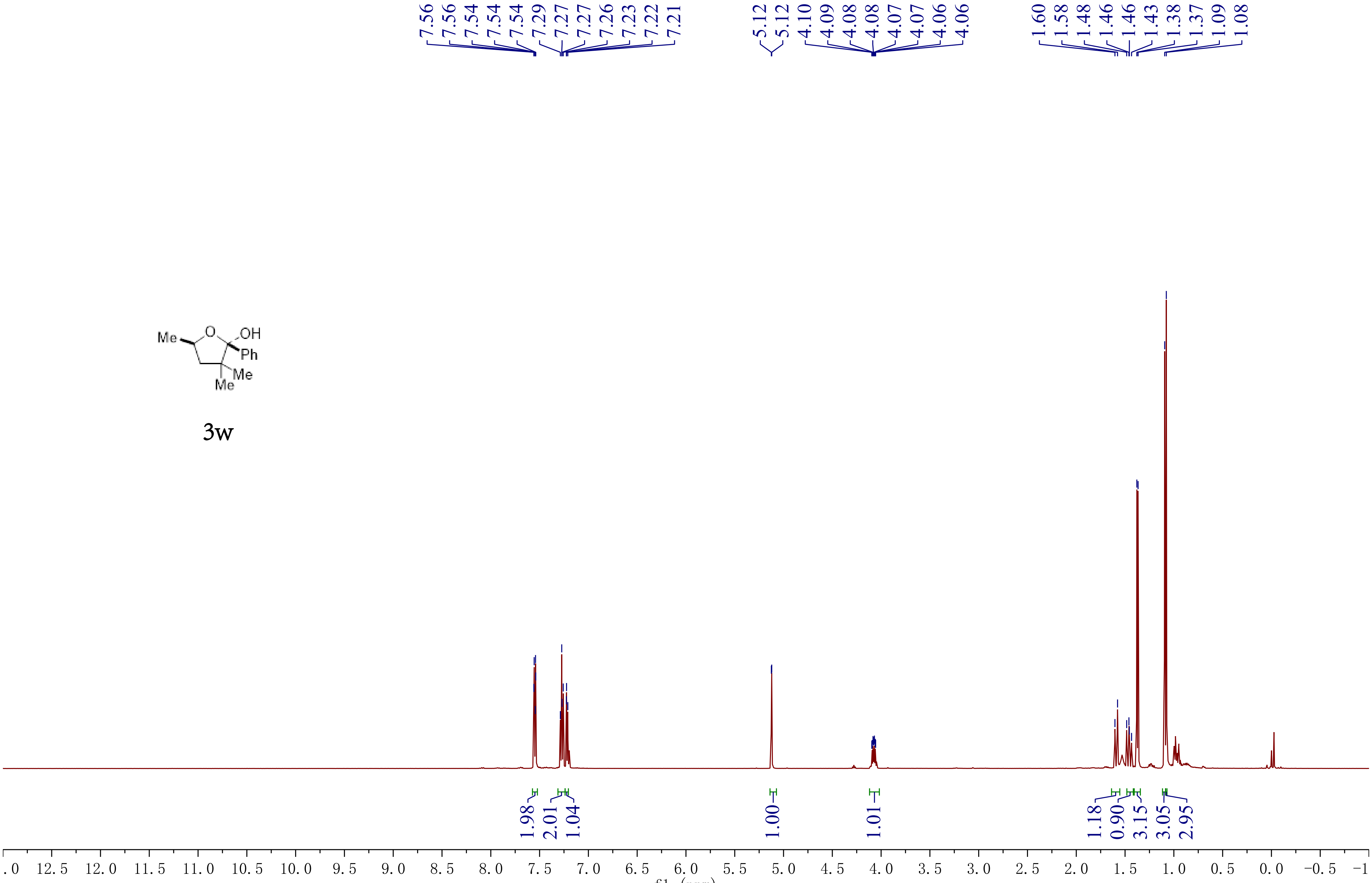

Supplementary Figure 47. ^{1}H NMR spectrum of **3w**, recorded at 500 MHz and 25 °C in $CDCl_3$

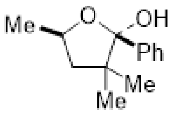

3w

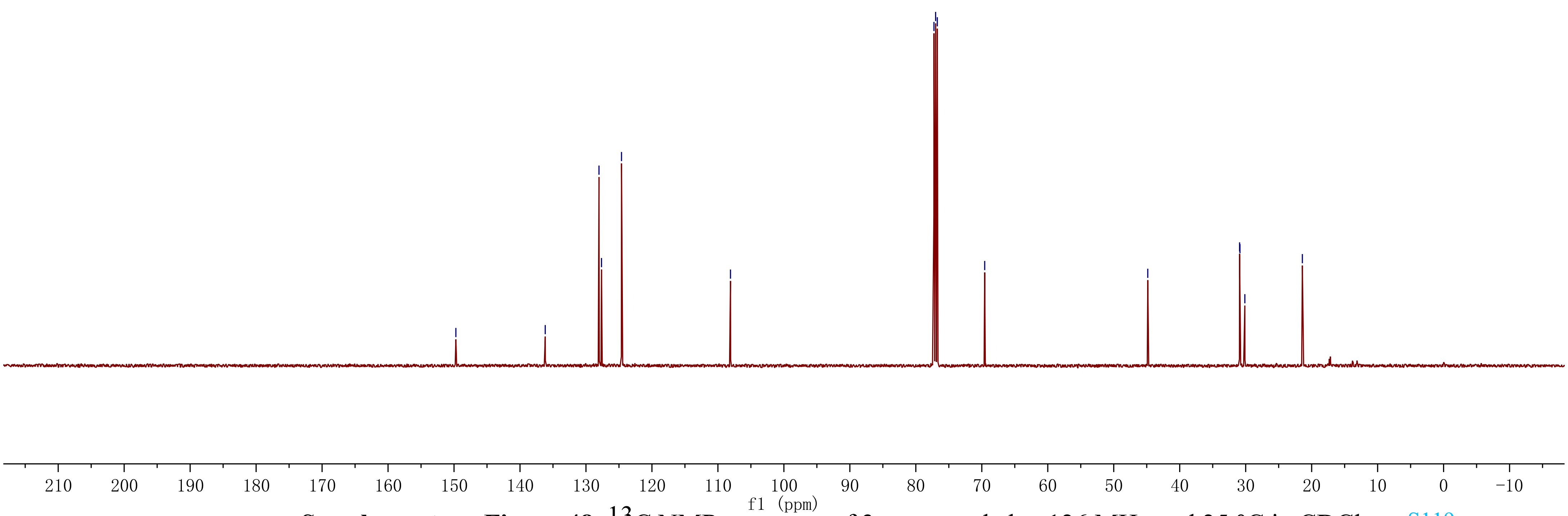

Supplementary Figure 48. ^{13}C NMR spectrum of **3w**, recorded at 126 MHz and 25 °C in $CDCl_3$

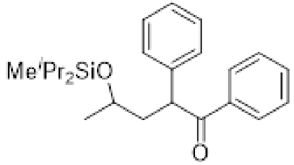

3x
d.r. = 1:1

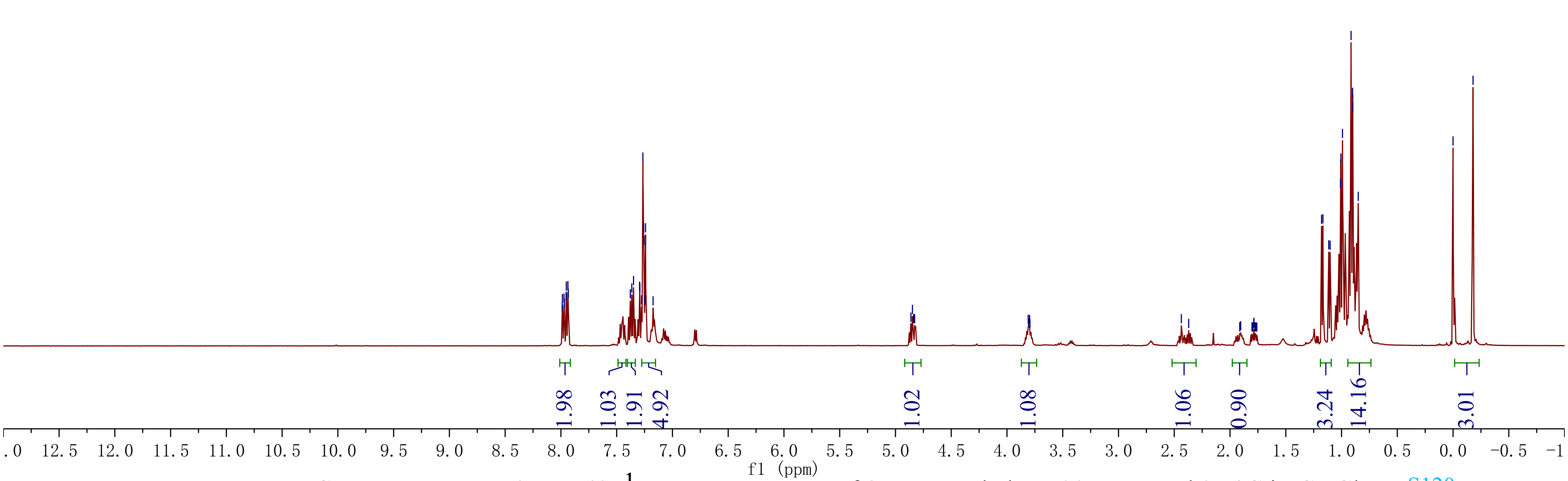

Supplementary Figure 49. ^{1}H NMR spectrum of **3x**, recorded at 500 MHz and 25 °C in $CDCl_3$

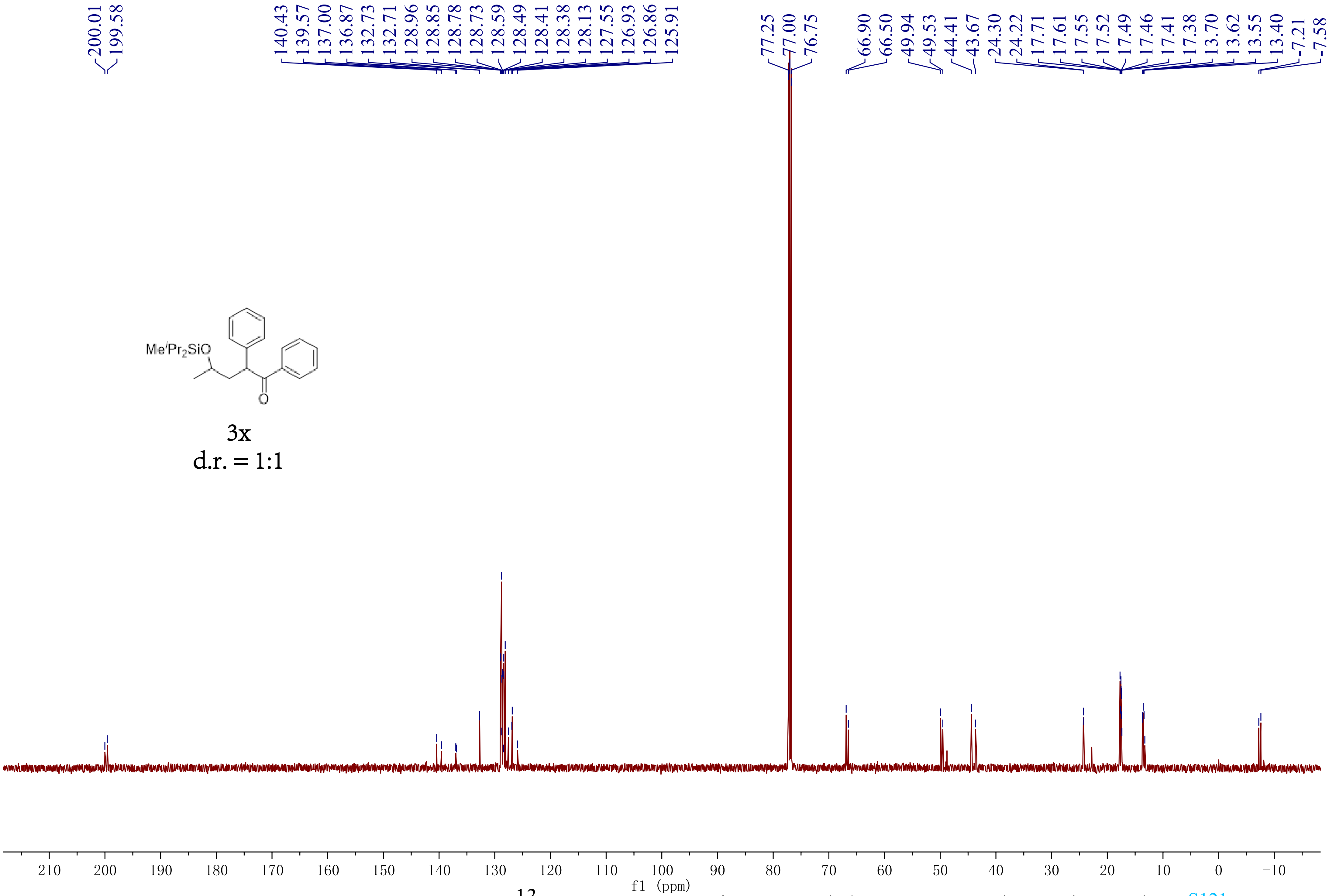

Supplementary Figure 50. ^{13}C NMR spectrum of **3x**, recorded at 126 MHz and 25 °C in $CDCl_3$

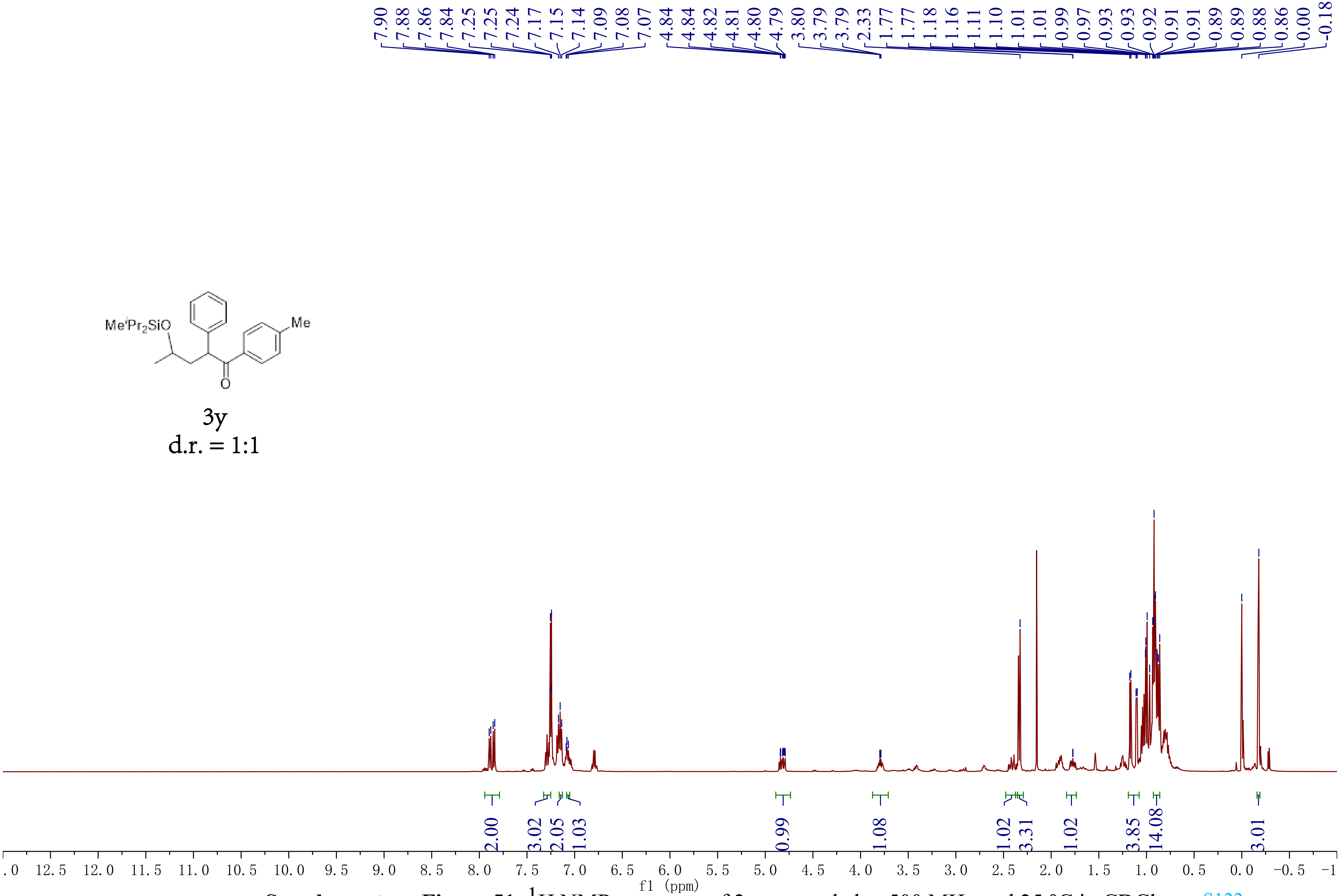

Supplementary Figure 51. ^{1}H NMR spectrum of **3y**, recorded at 500 MHz and 25 °C in $CDCl_3$

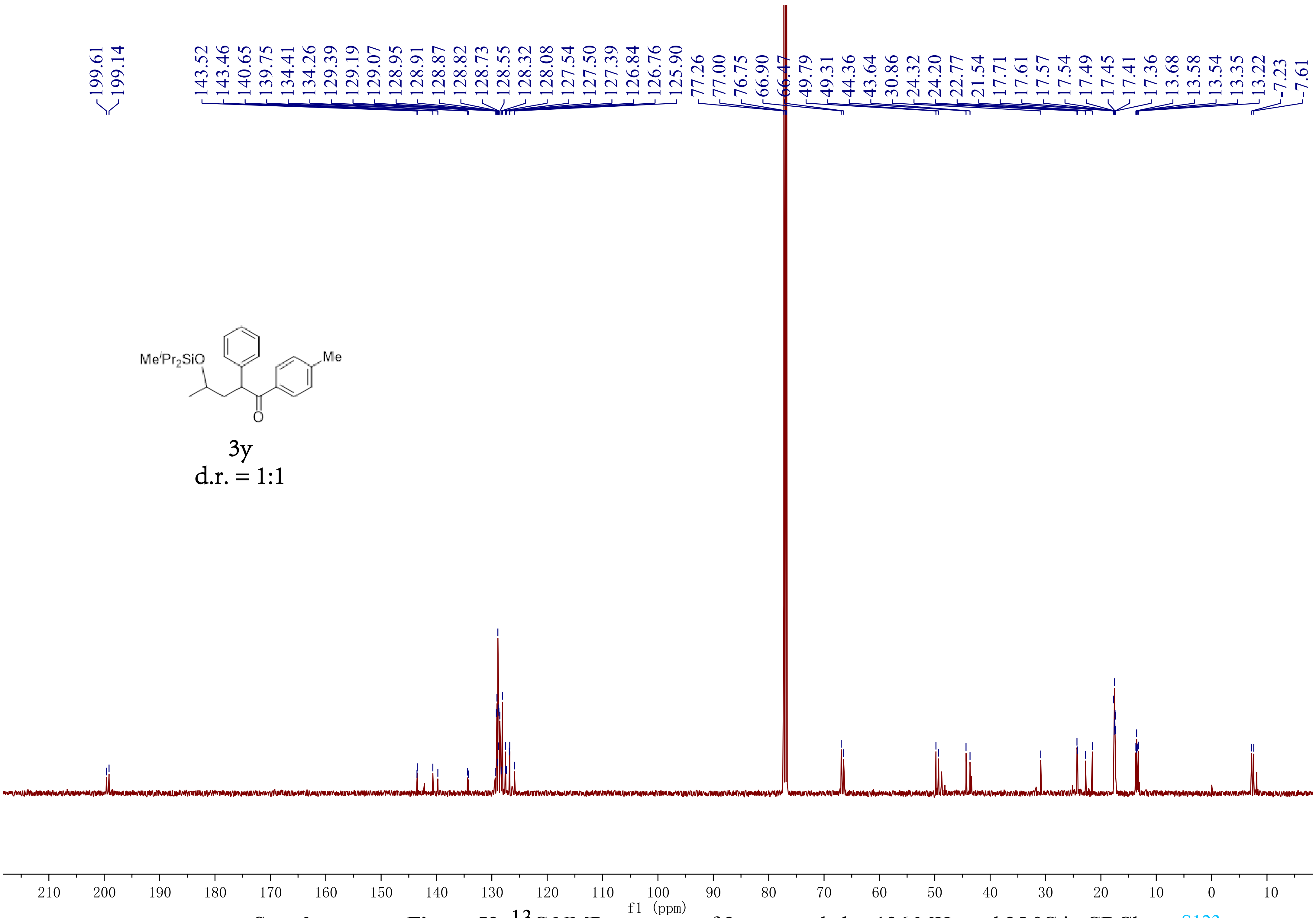

Supplementary Figure 52. ^{13}C NMR spectrum of **3y**, recorded at 126 MHz and 25 °C in $CDCl_3$

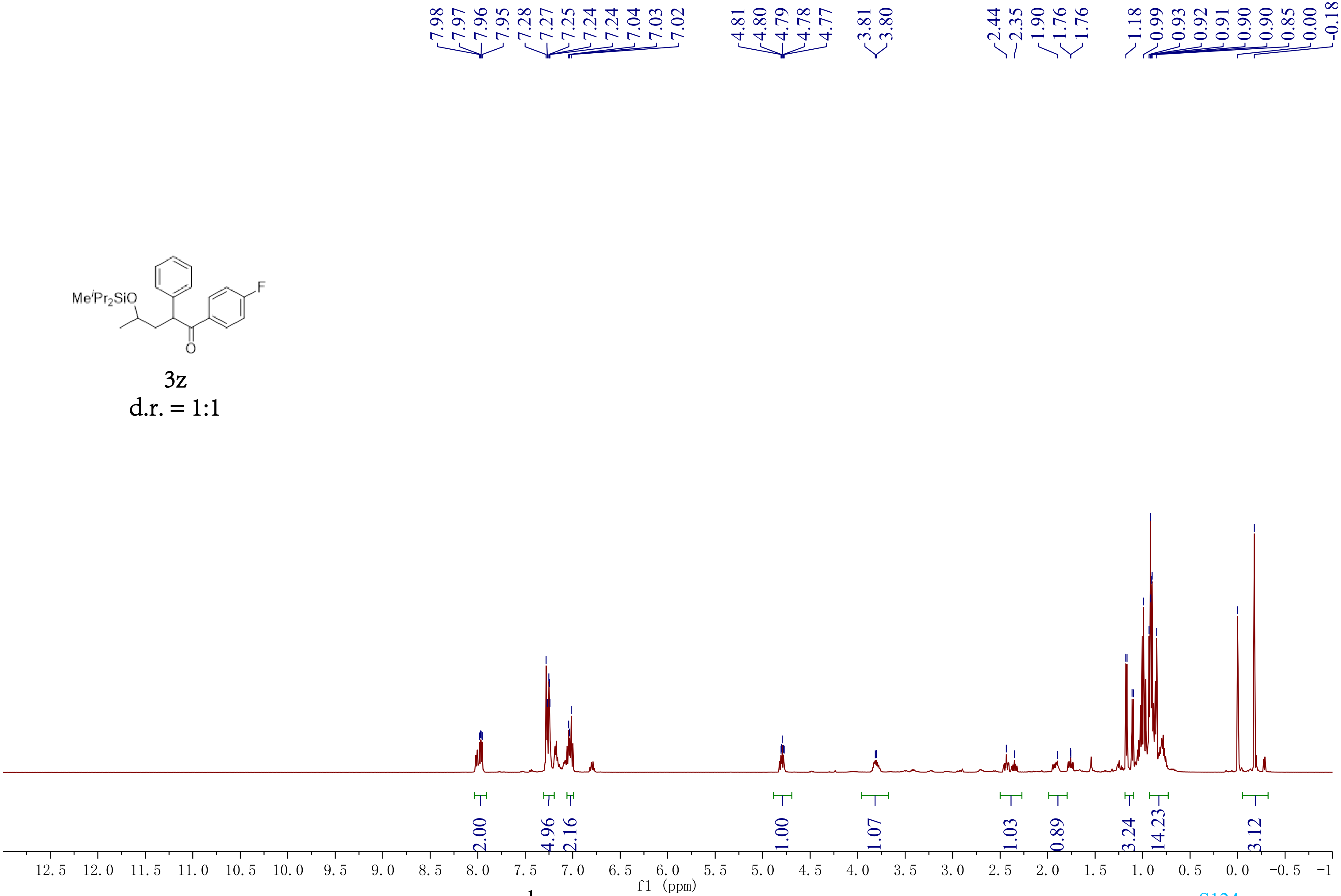

Supplementary Figure 53. ^{1}H NMR spectrum of **3z**, recorded at 500 MHz and 25 °C in $CDCl_3$

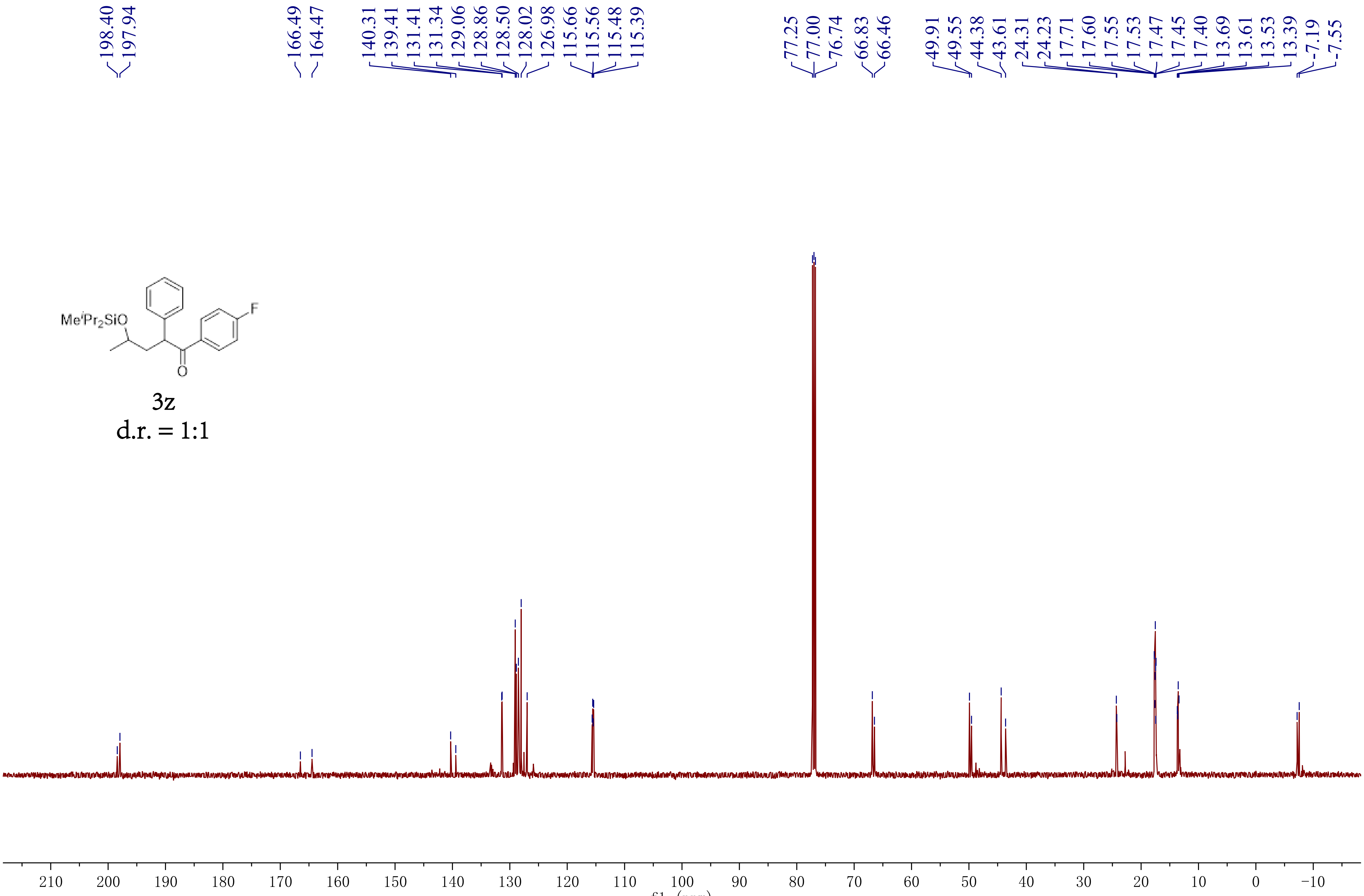

3z
d.r. = 1:1

Supplementary Figure 54. ^{13}C NMR spectrum of **3z**, recorded at 126 MHz and 25 °C in $CDCl_3$

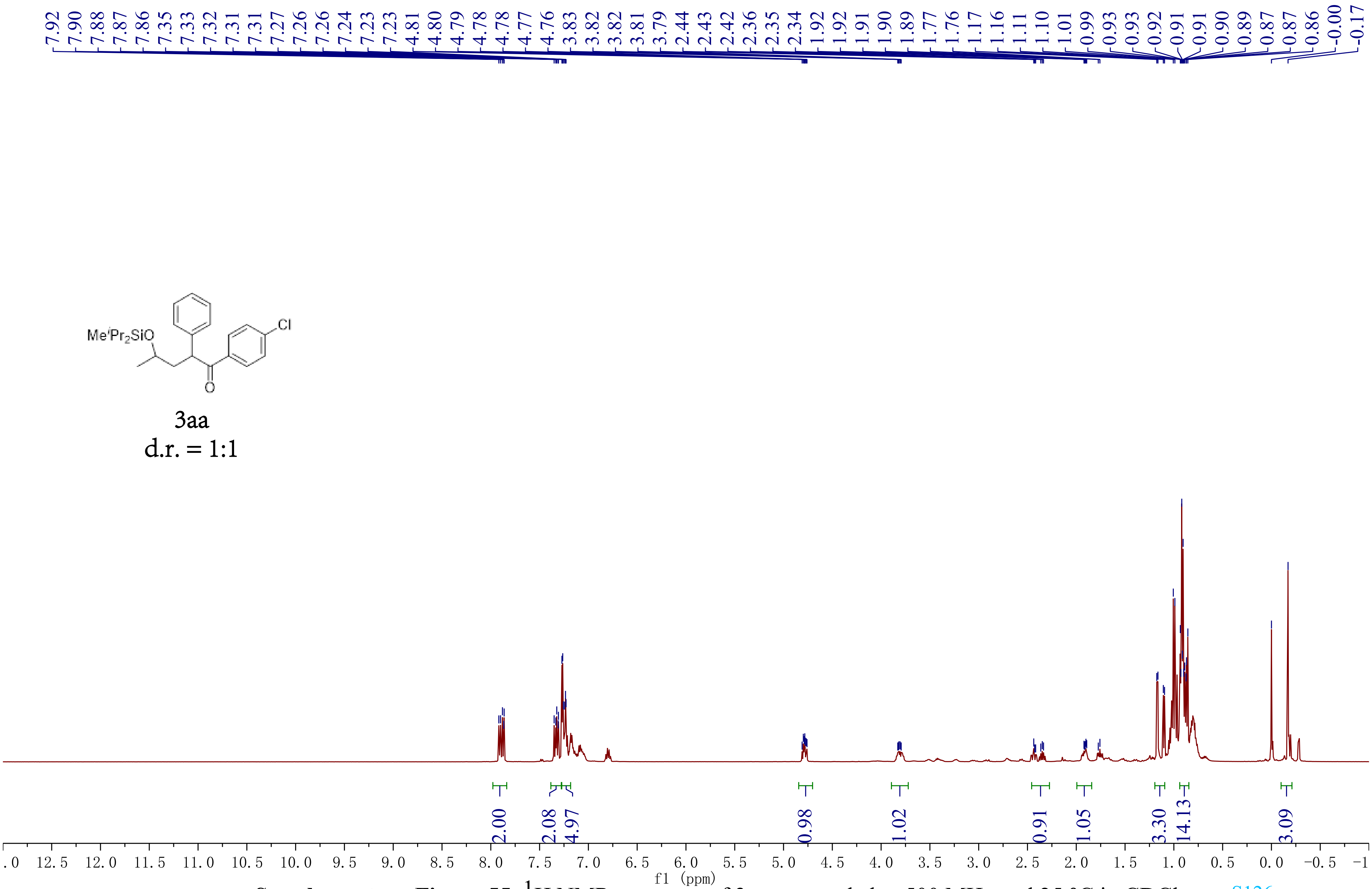

Supplementary Figure 55. ^{1}H NMR spectrum of **3aa**, recorded at 500 MHz and 25 °C in $CDCl_3$

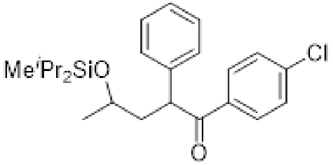

3aa
d.r. = 1:1

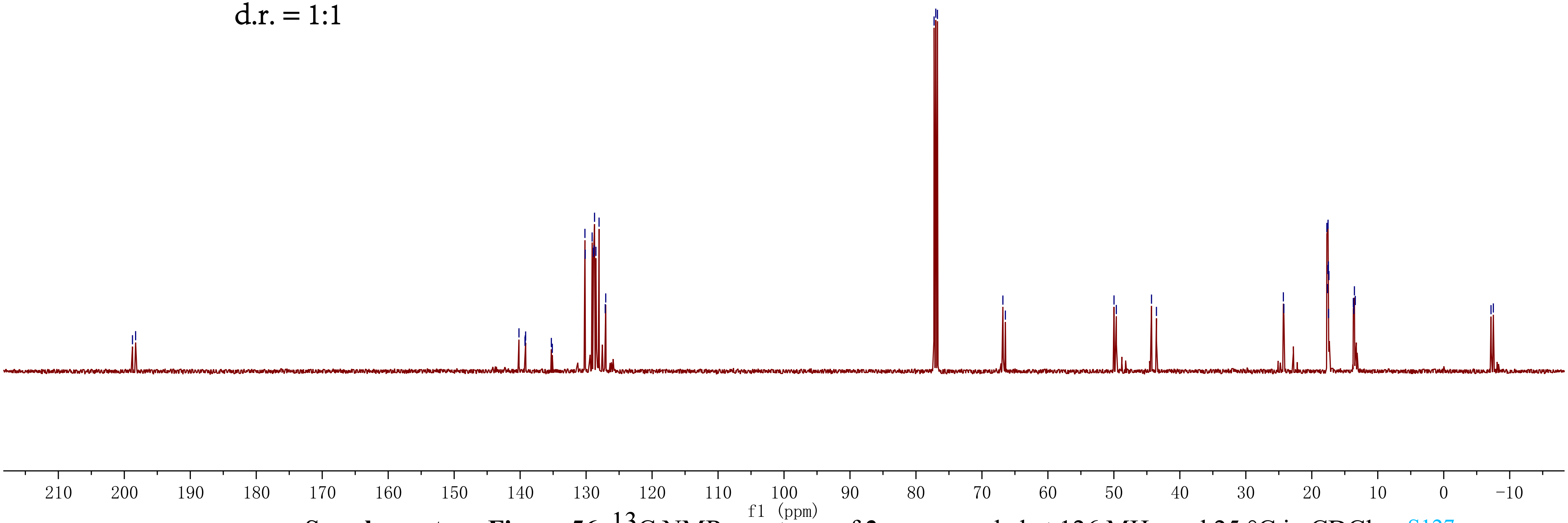

Supplementary Figure 56. ^{13}C NMR spectrum of **3aa**, recorded at 126 MHz and 25 °C in $CDCl_3$

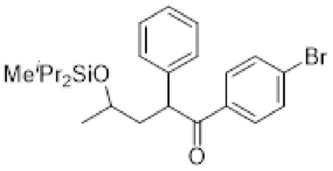

3ab
d.r. = 1:1

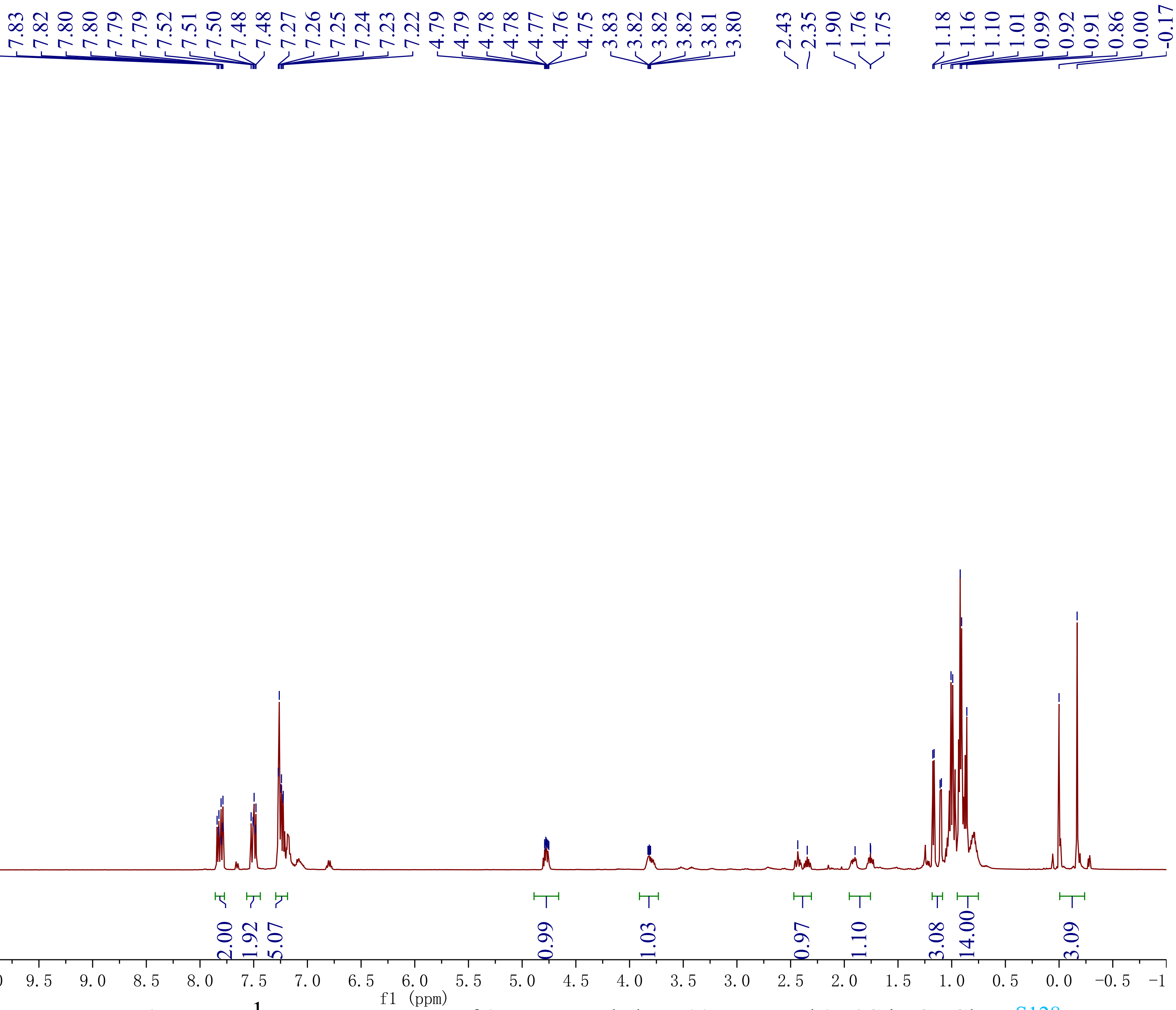

Supplementary Figure 57. ^{1}H NMR spectrum of **3ab**, recorded at 500 MHz and 25 °C in $CDCl_3$

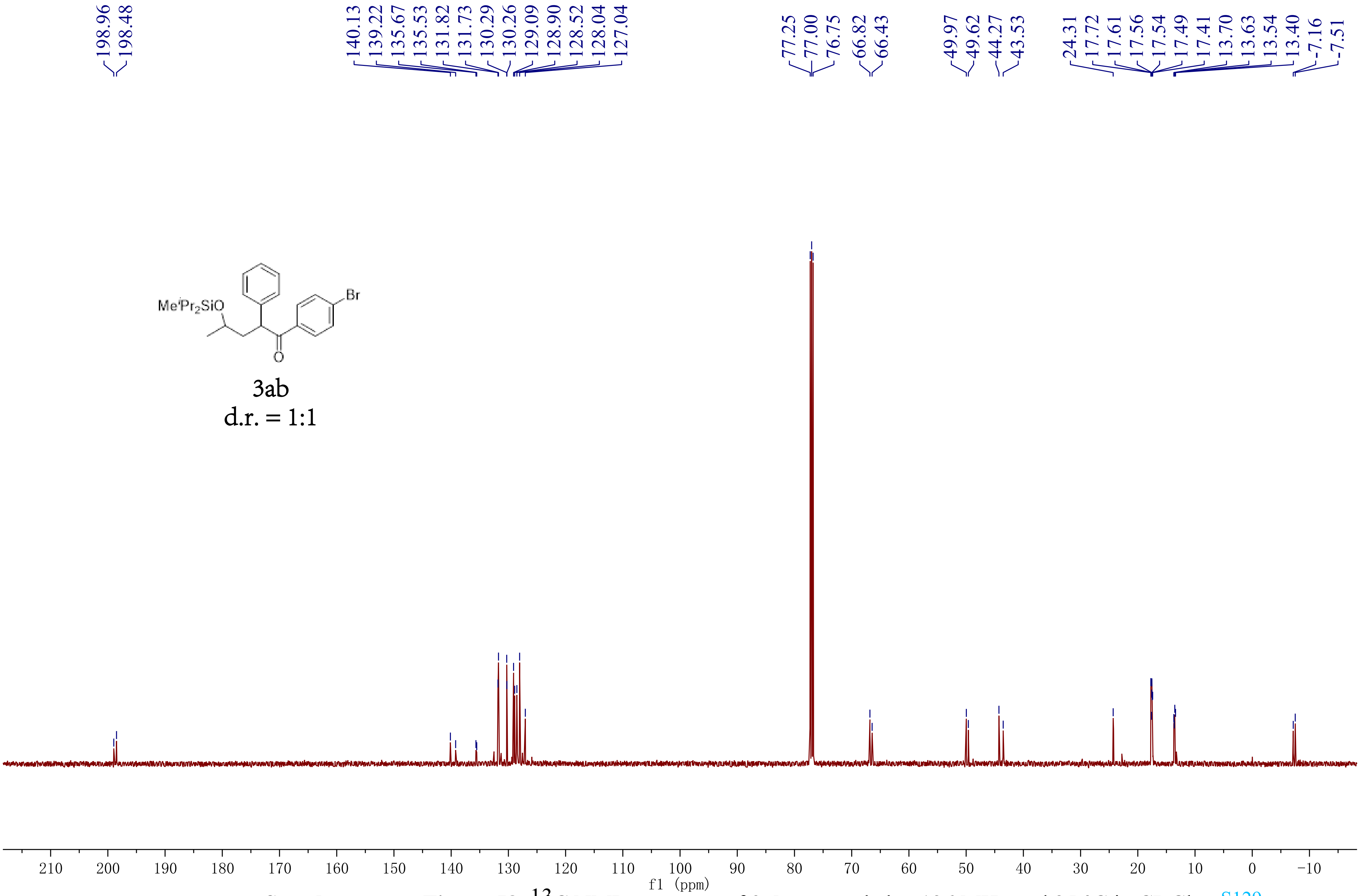

Supplementary Figure 58. ^{13}C NMR spectrum of **3ab**, recorded at 126 MHz and 25 °C in $CDCl_3$

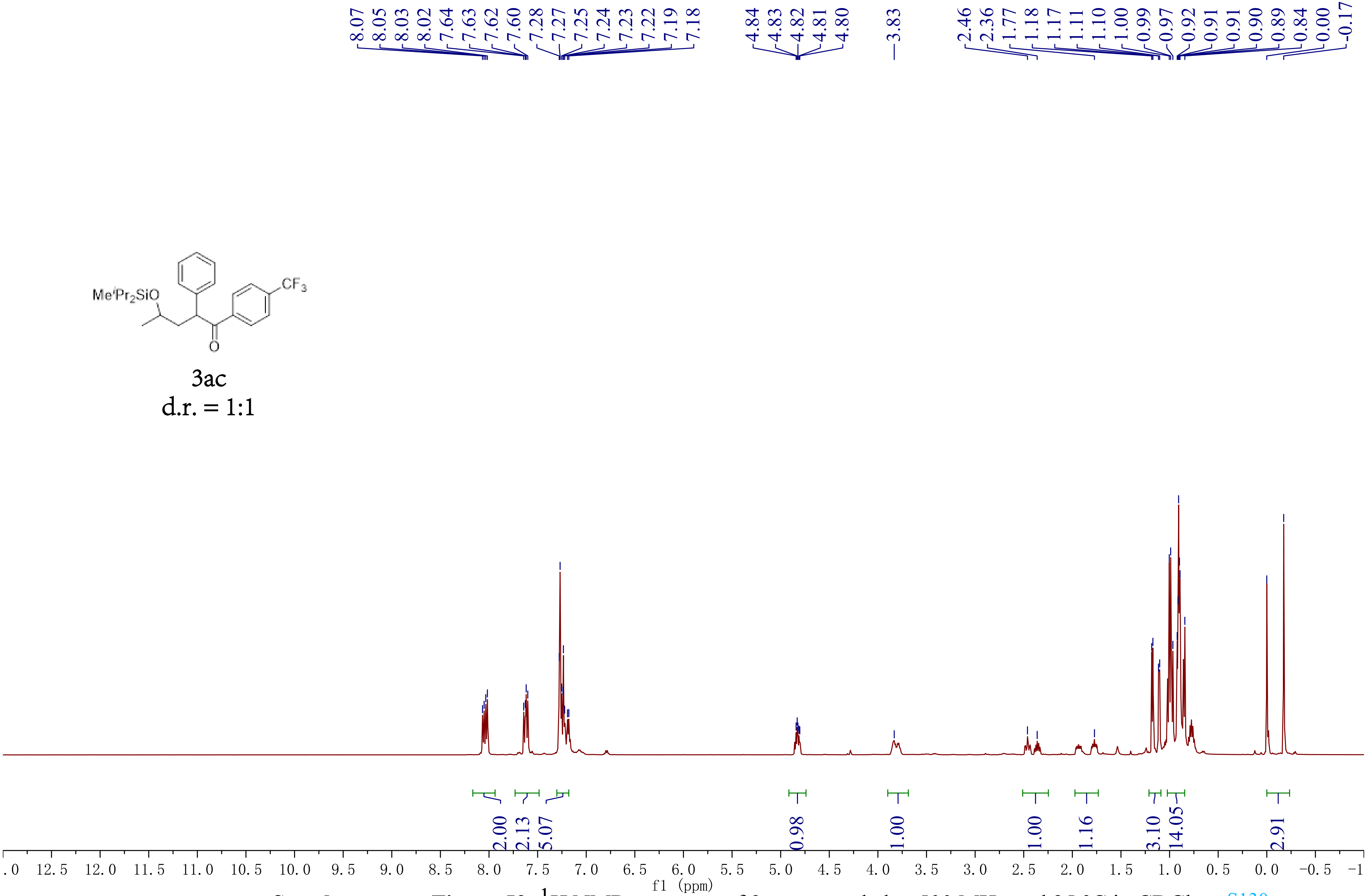

Supplementary Figure 59. ^{1}H NMR spectrum of **3ac**, recorded at 500 MHz and 25 °C in $CDCl_3$

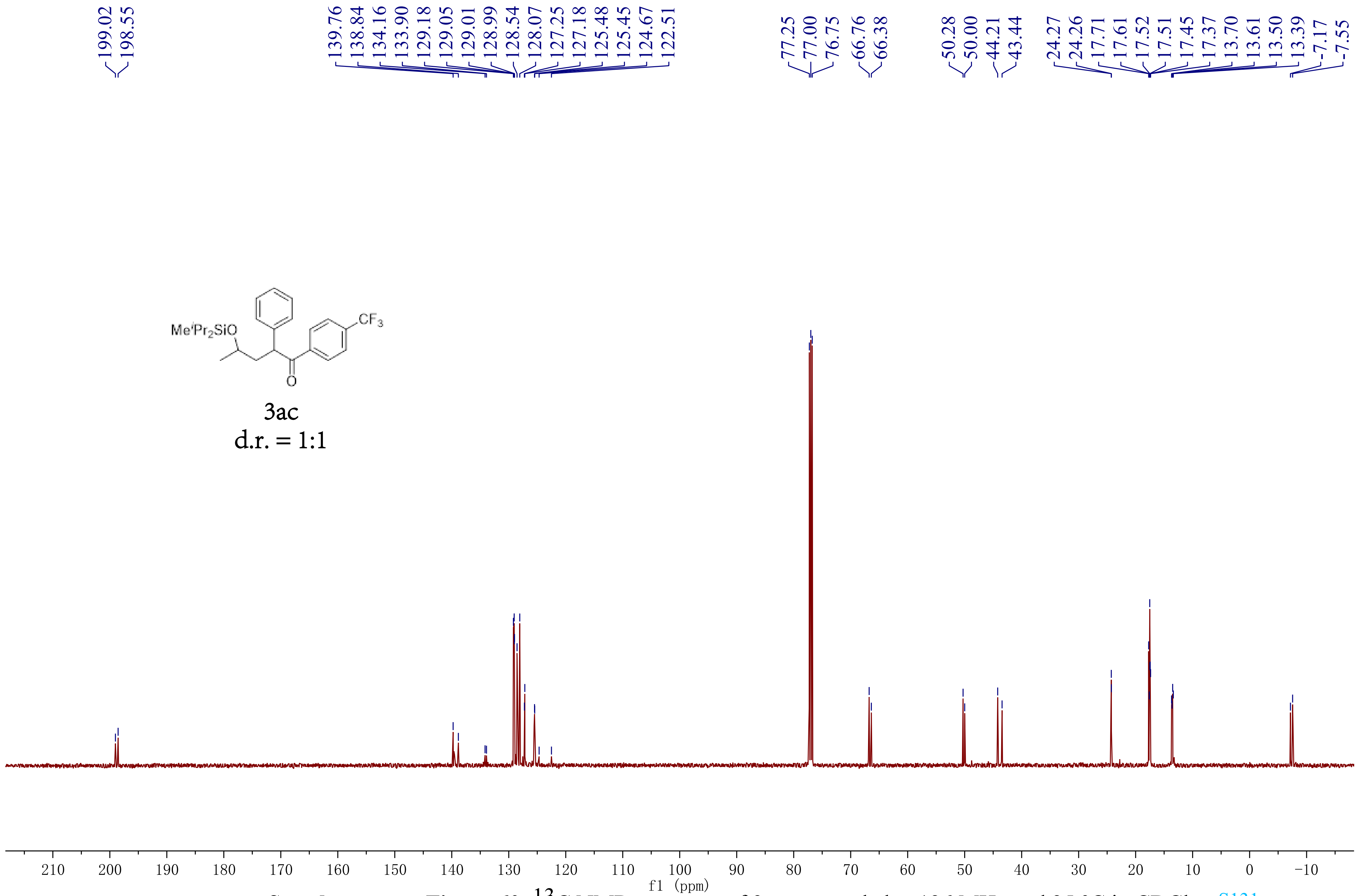

Supplementary Figure 60. ^{13}C NMR spectrum of **3ac**, recorded at 126 MHz and 25 °C in $CDCl_3$

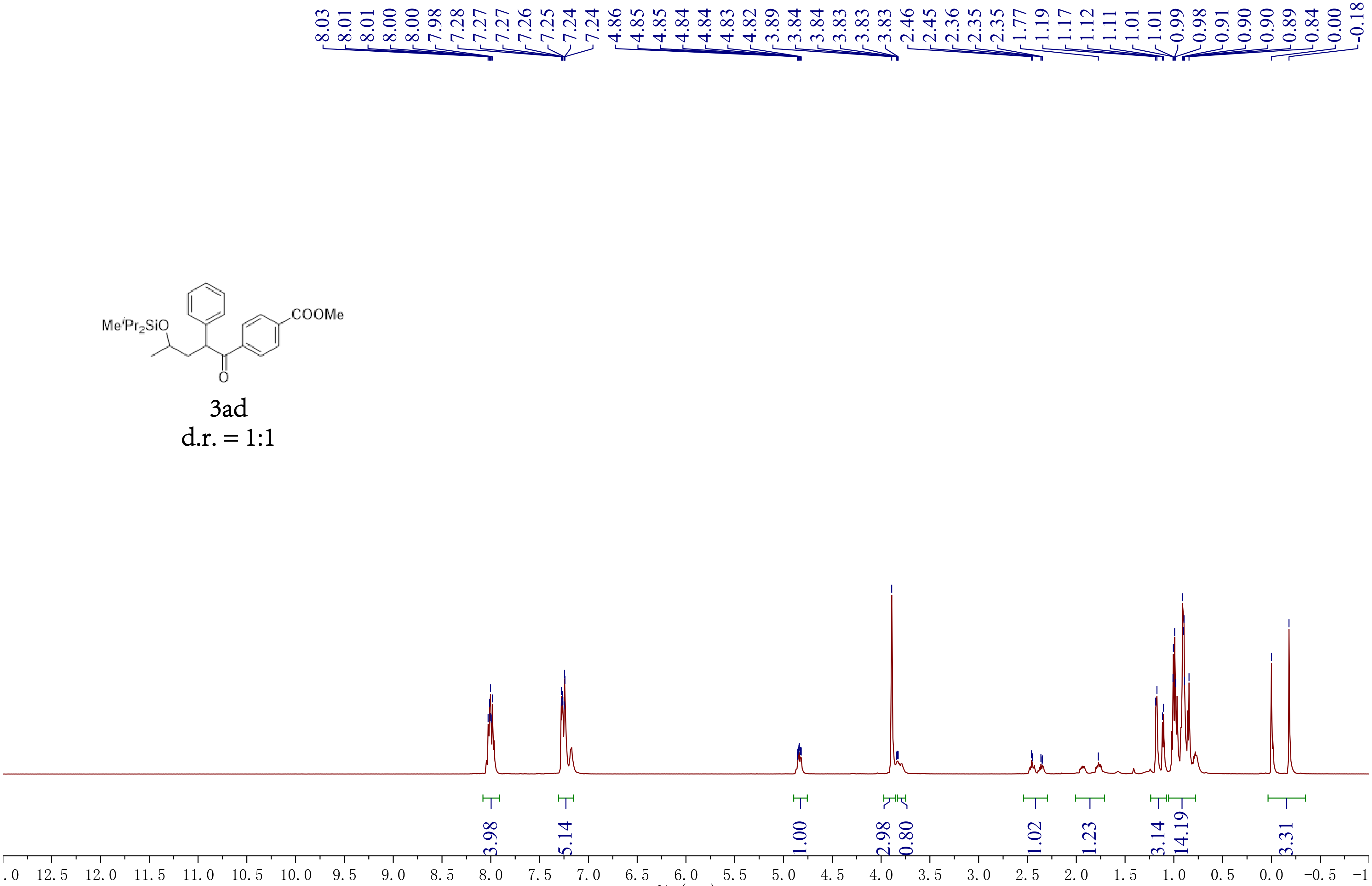

Supplementary Figure 61. ^{1}H NMR spectrum of **3ad**, recorded at 500 MHz and 25 °C in $CDCl_3$

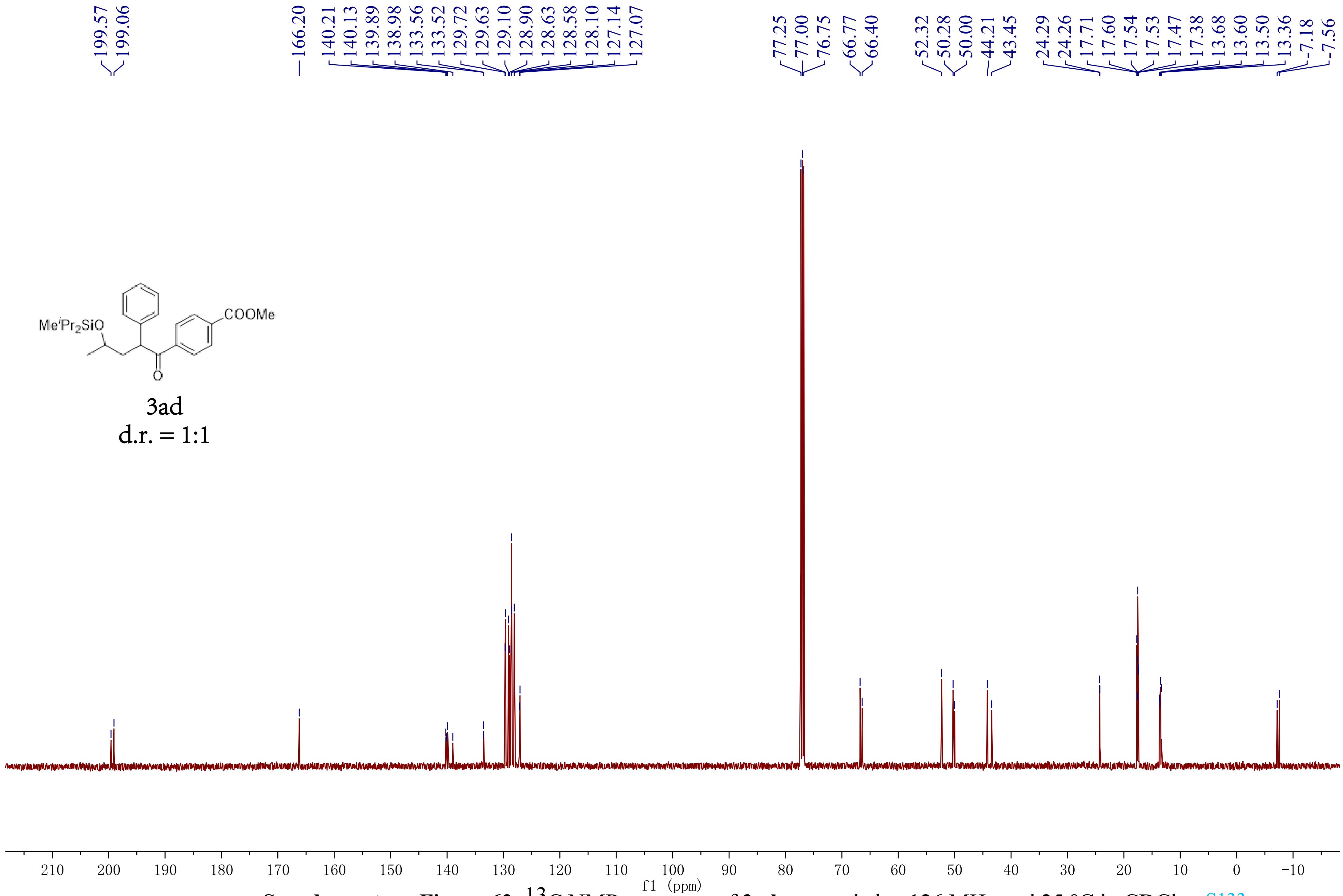

3ad
d.r. = 1:1

Supplementary Figure 62. ^{13}C NMR spectrum of **3ad**, recorded at 126 MHz and 25 °C in $CDCl_3$

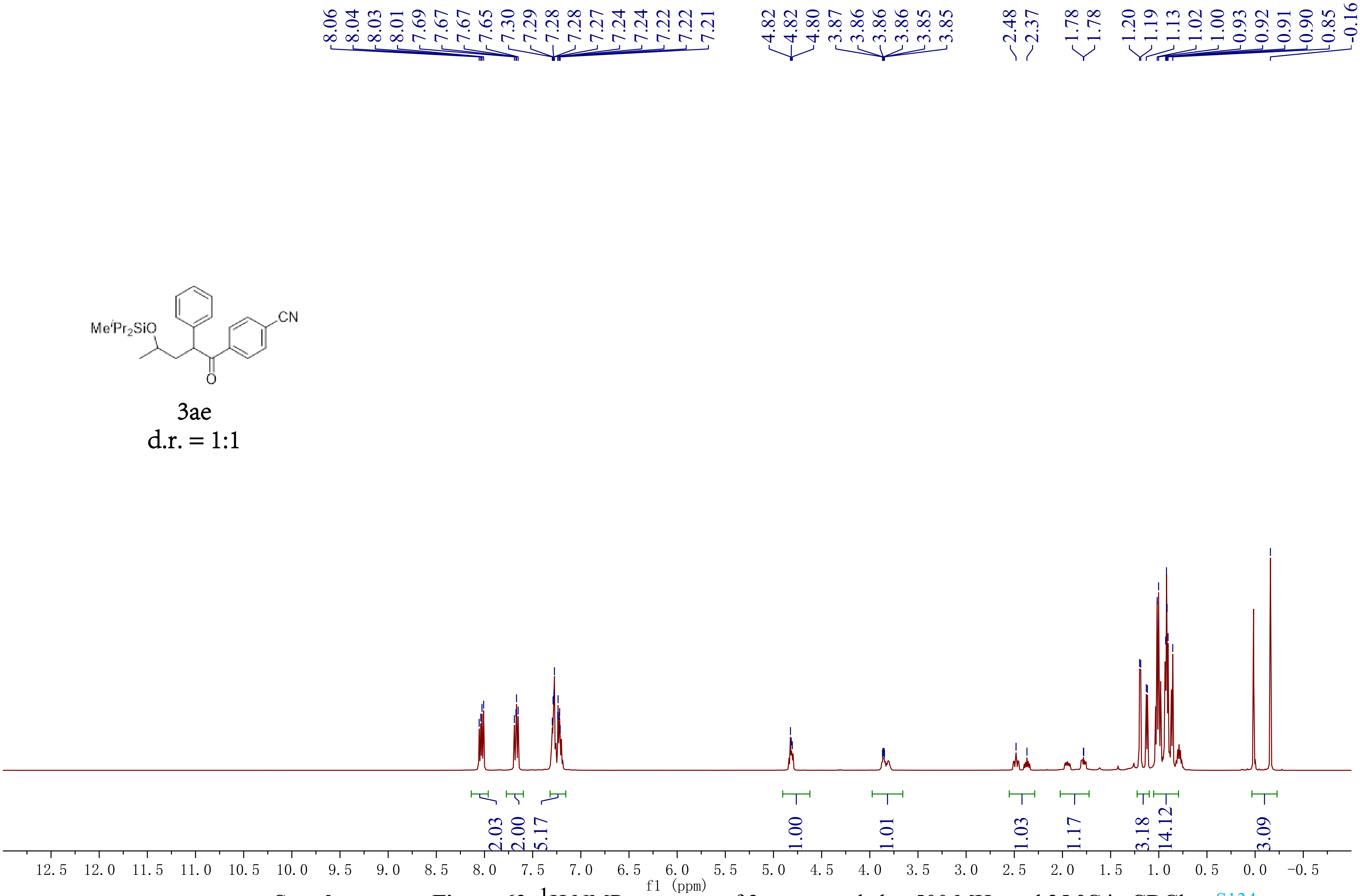

Supplementary Figure 63. ^{1}H NMR spectrum of **3ae**, recorded at 500 MHz and 25 °C in $CDCl_3$

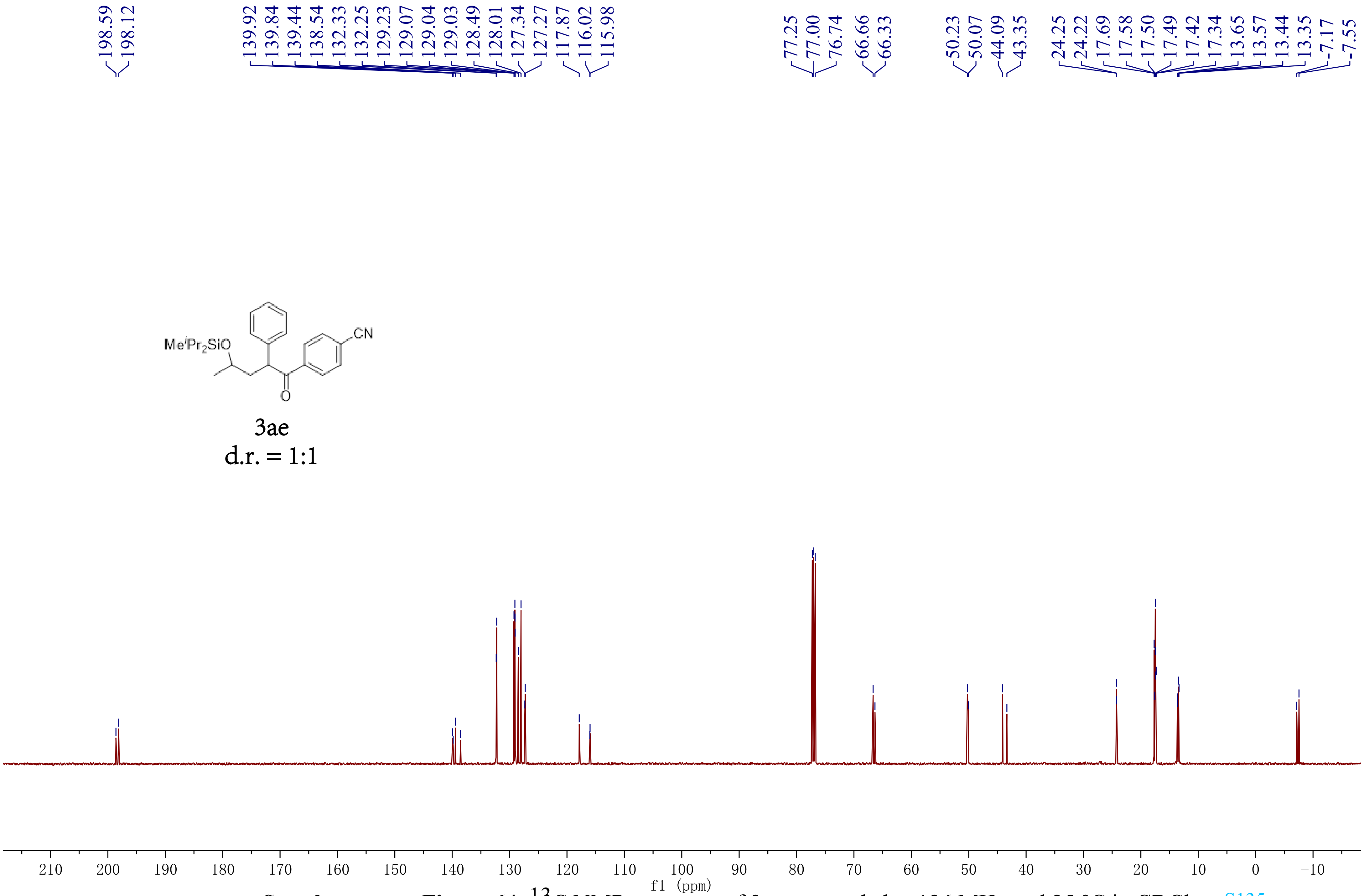

Supplementary Figure 64. ^{13}C NMR spectrum of **3ae**, recorded at 126 MHz and 25 °C in $CDCl_3$

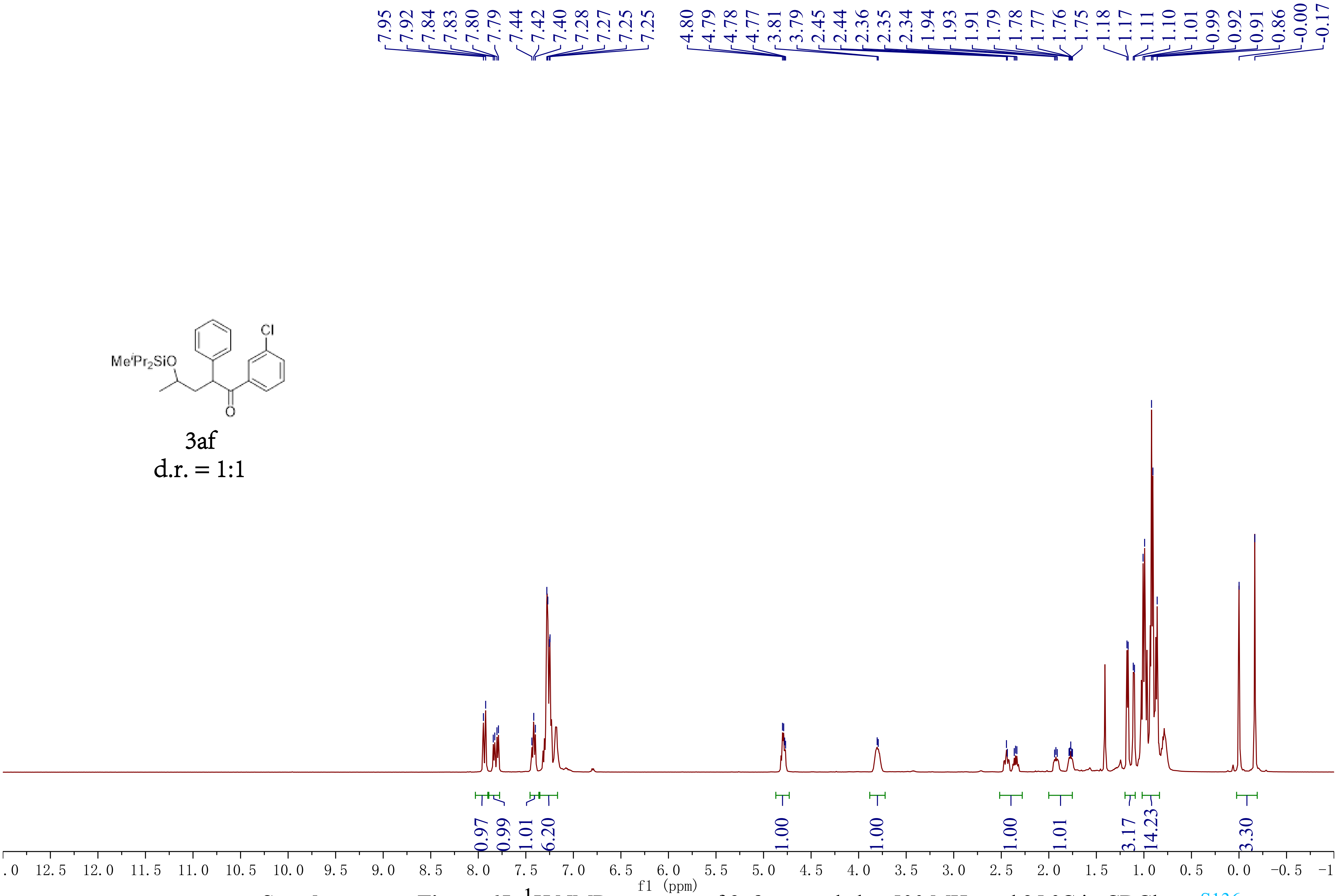

Supplementary Figure 65. ^{1}H NMR spectrum of **3af**, recorded at 500 MHz and 25 °C in $CDCl_3$

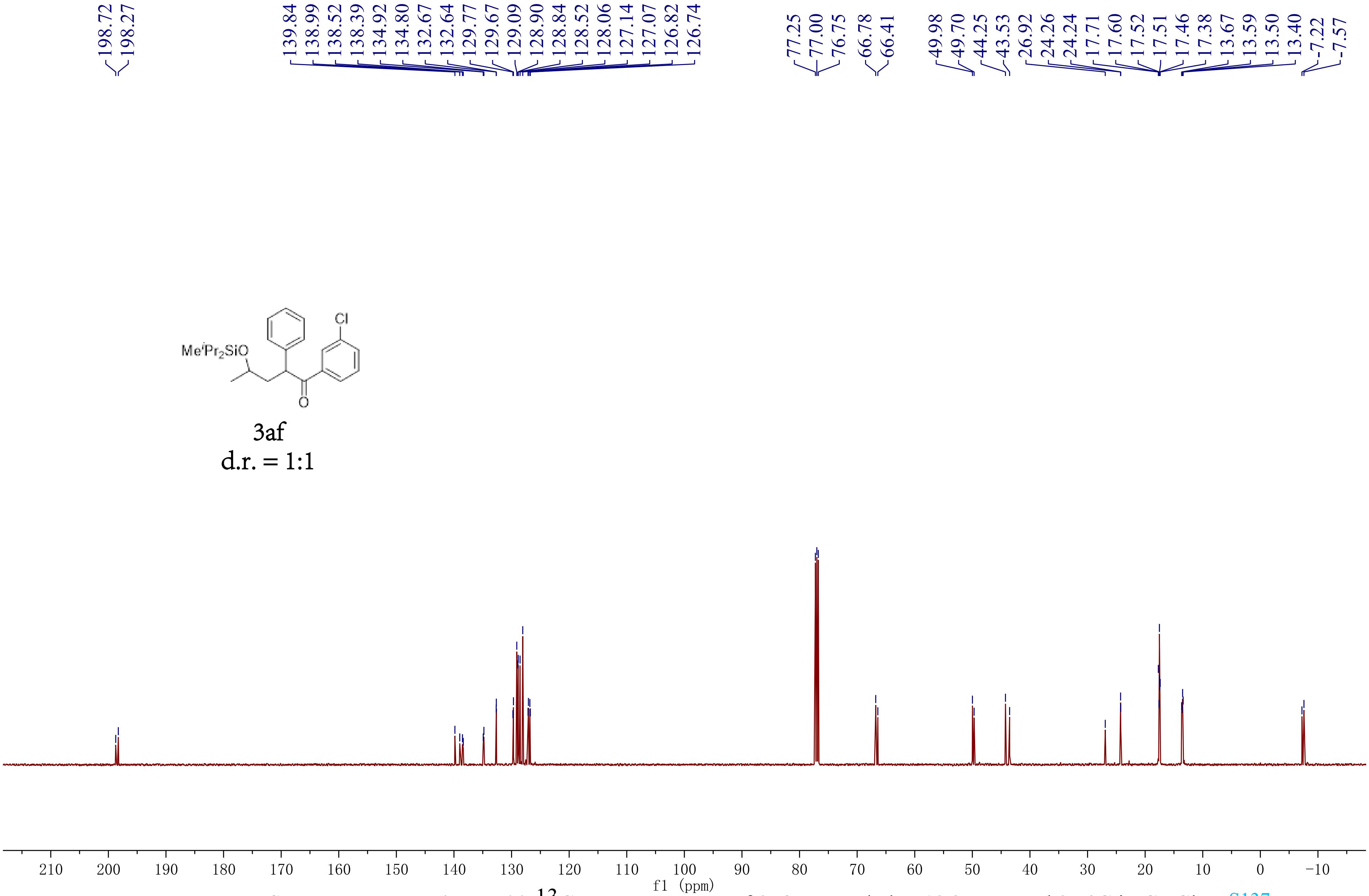

Supplementary Figure 66. ^{13}C NMR spectrum of **3af**, recorded at 126 MHz and 25 °C in $CDCl_3$

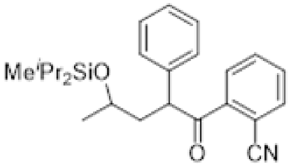

3ag
d.r. = 1:1

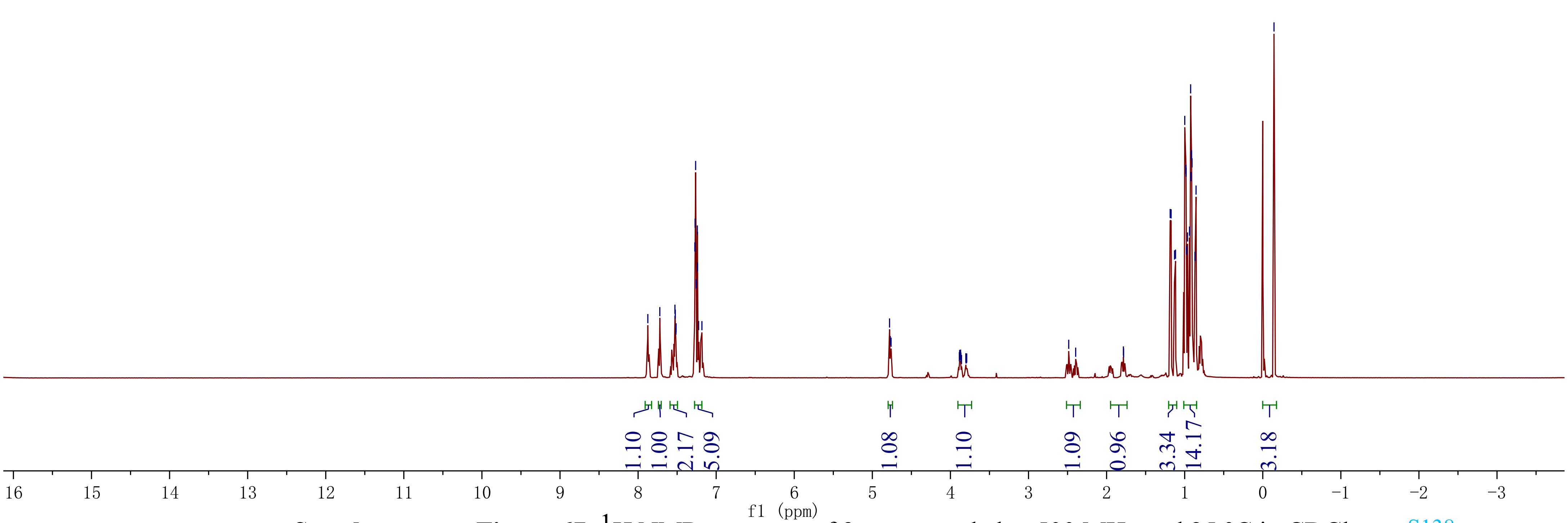

Supplementary Figure 67. ^{1}H NMR spectrum of **3ag**, recorded at 500 MHz and 25 °C in $CDCl_3$

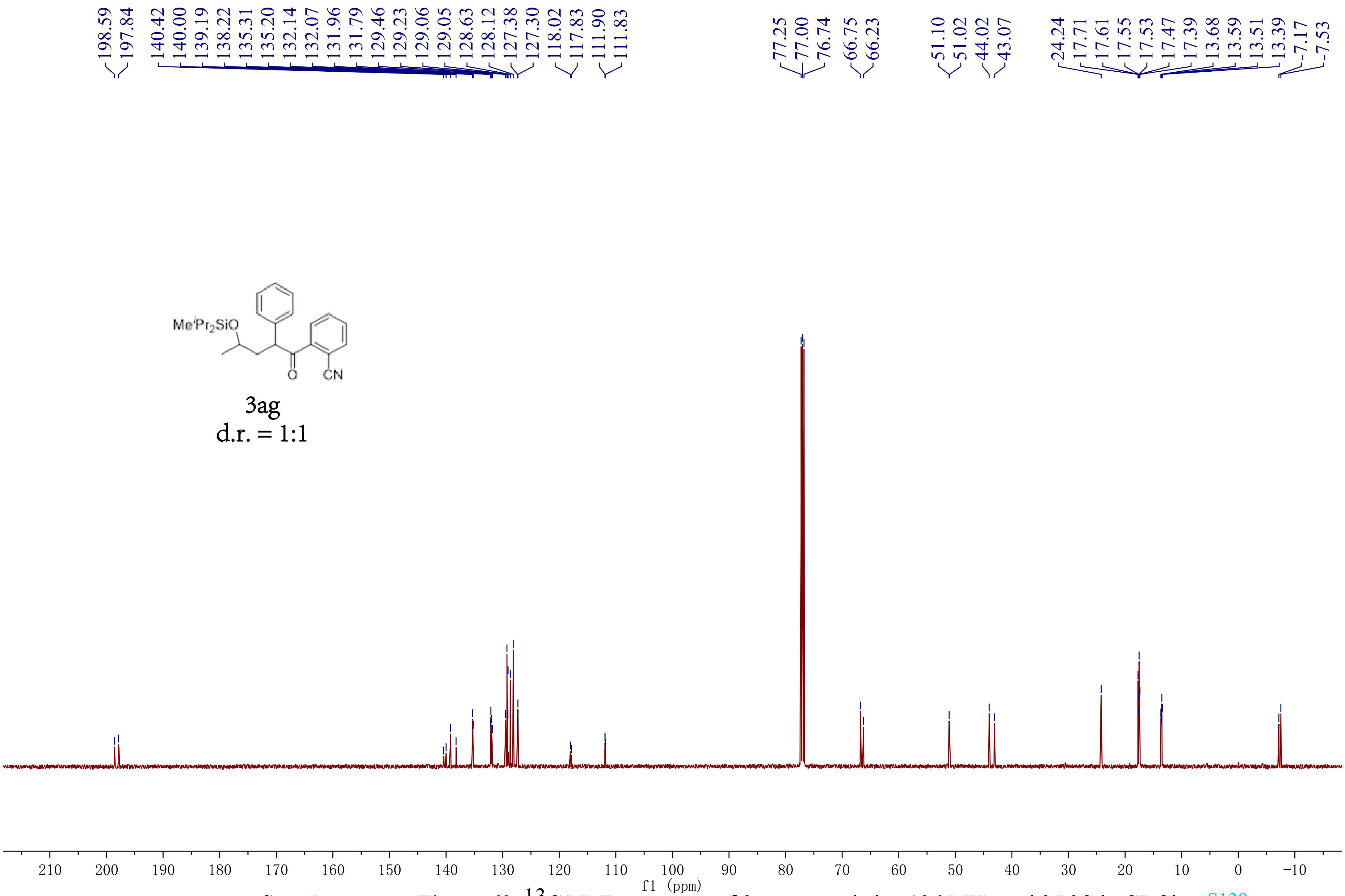

Supplementary Figure 68. ^{13}C NMR spectrum of **3ag**, recorded at 126 MHz and 25 °C in $CDCl_3$

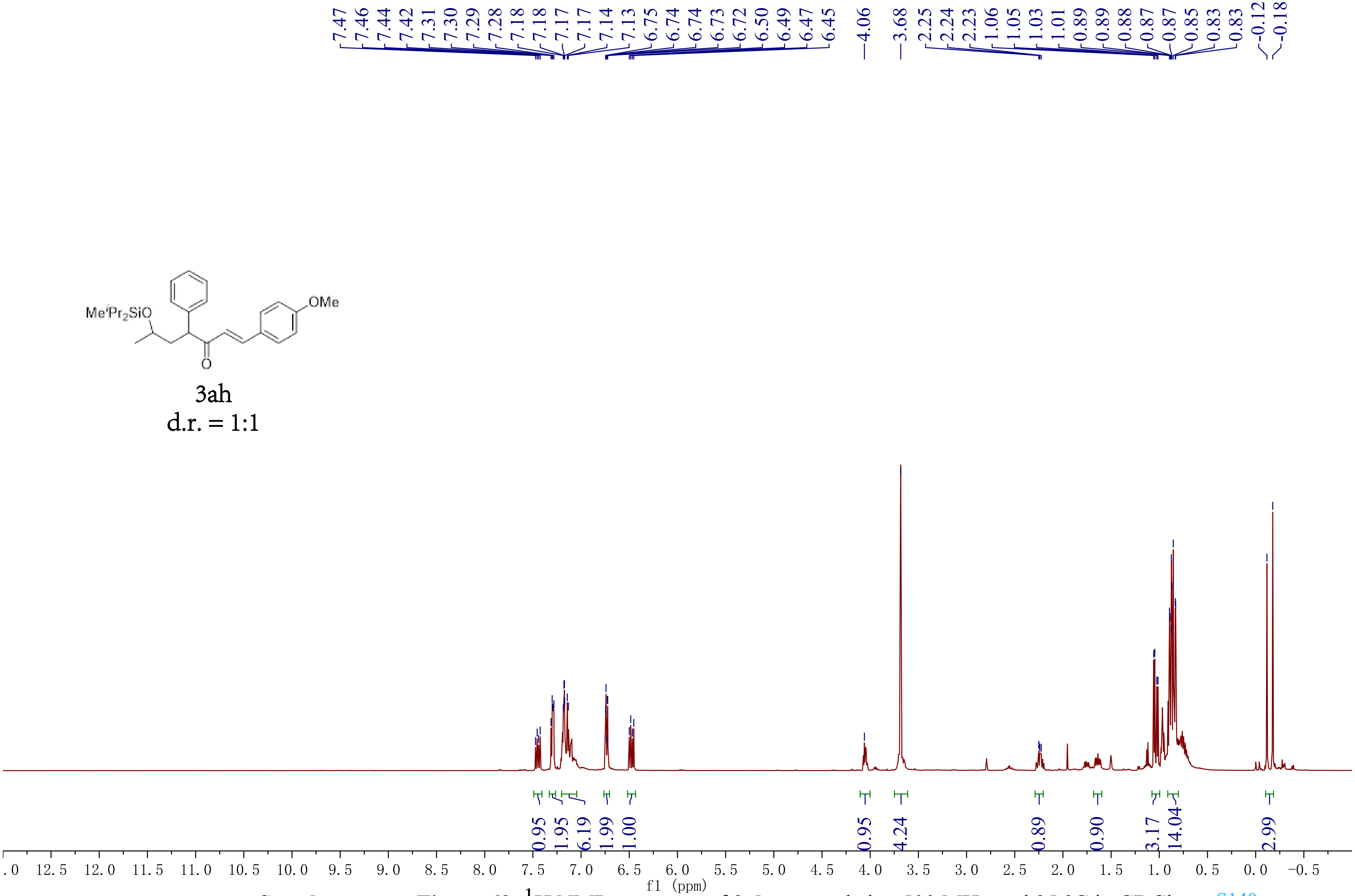

Supplementary Figure 69. ^{1}H NMR spectrum of **3ah**, recorded at 500 MHz and 25 °C in $CDCl_3$

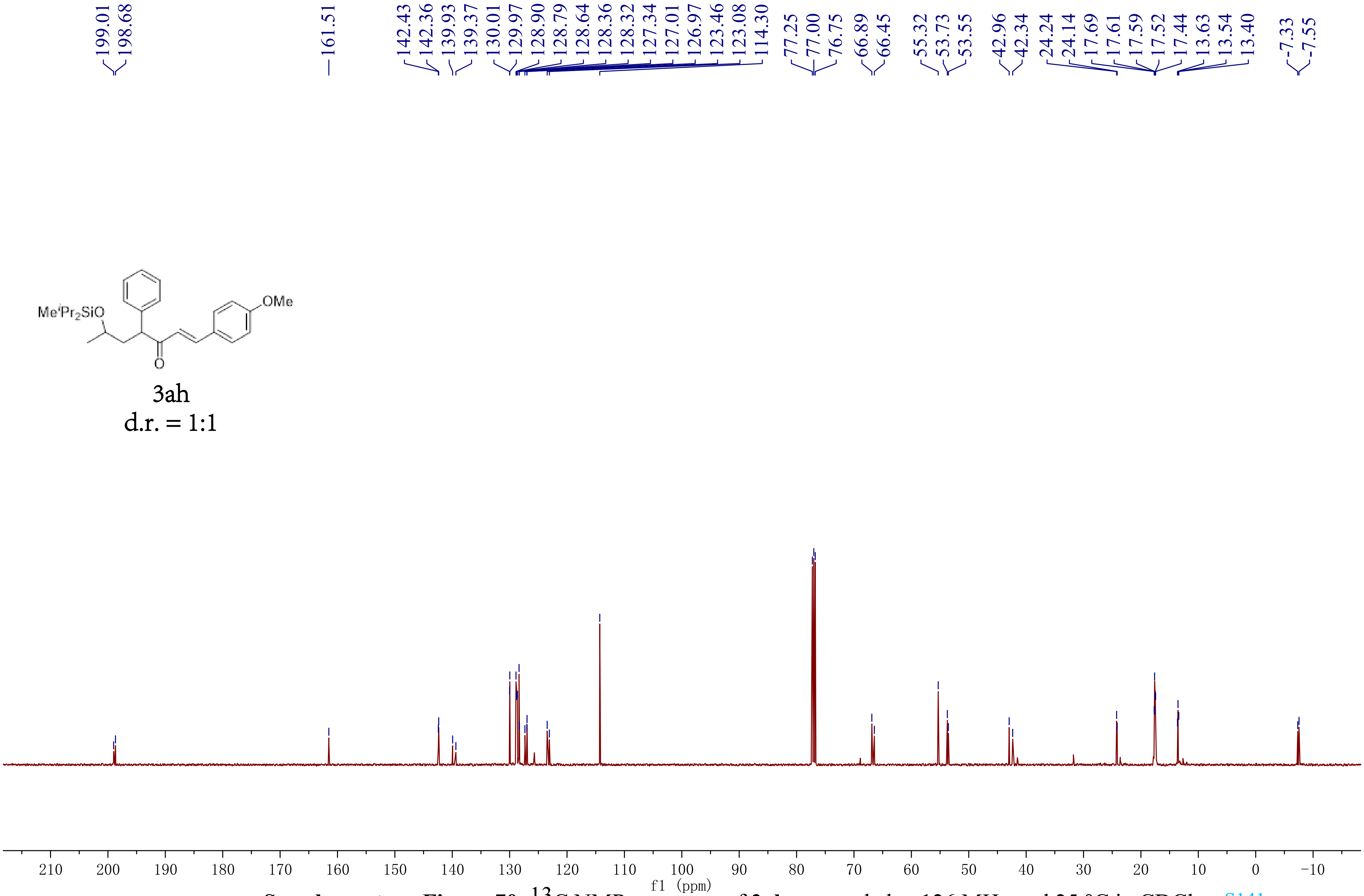

Supplementary Figure 70. ^{13}C NMR spectrum of **3ah**, recorded at 126 MHz and 25 °C in $CDCl_3$

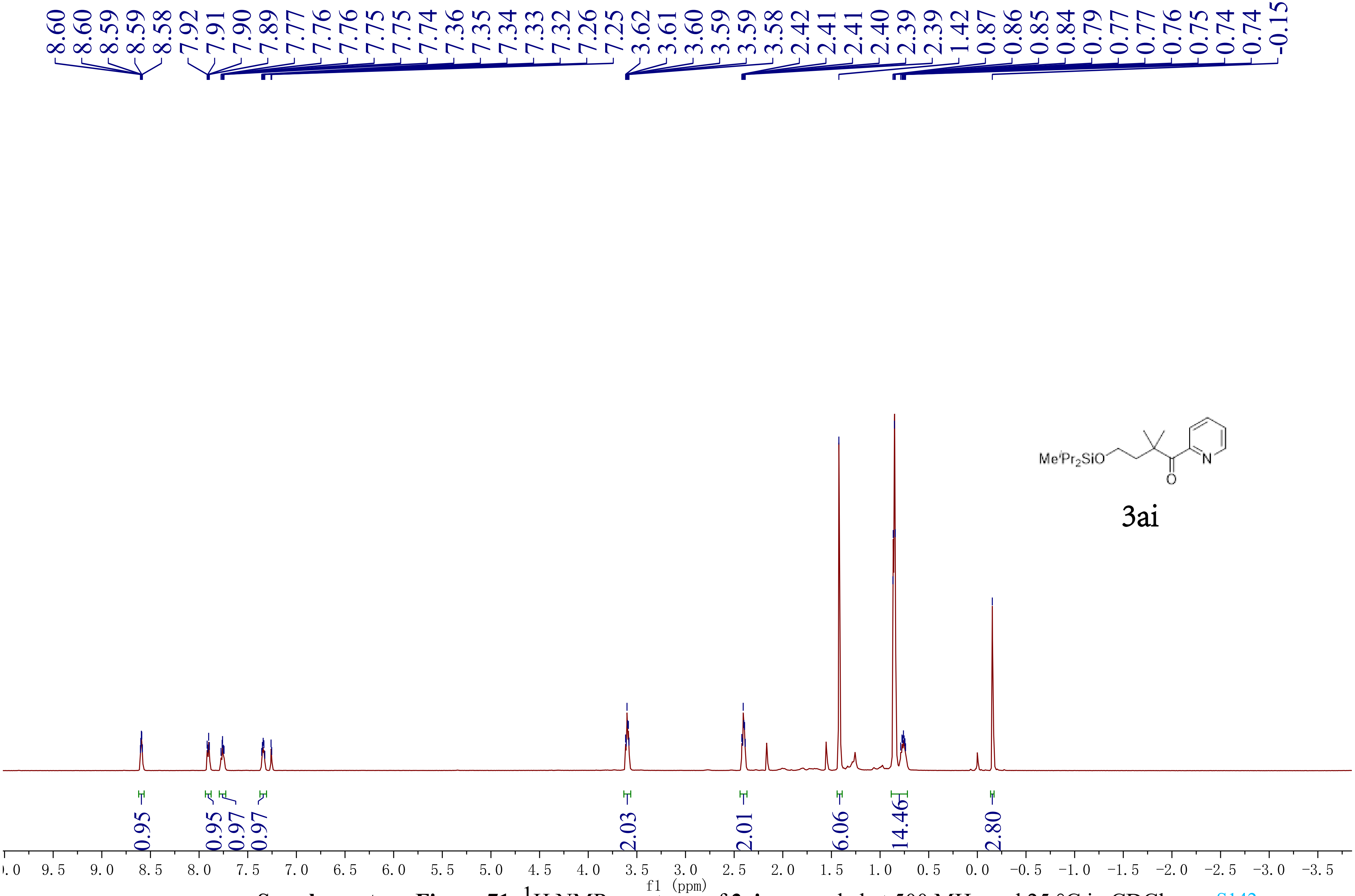

3ai

Supplementary Figure 71. ^{1}H NMR spectrum of **3ai**, recorded at 500 MHz and 25 °C in $CDCl_3$

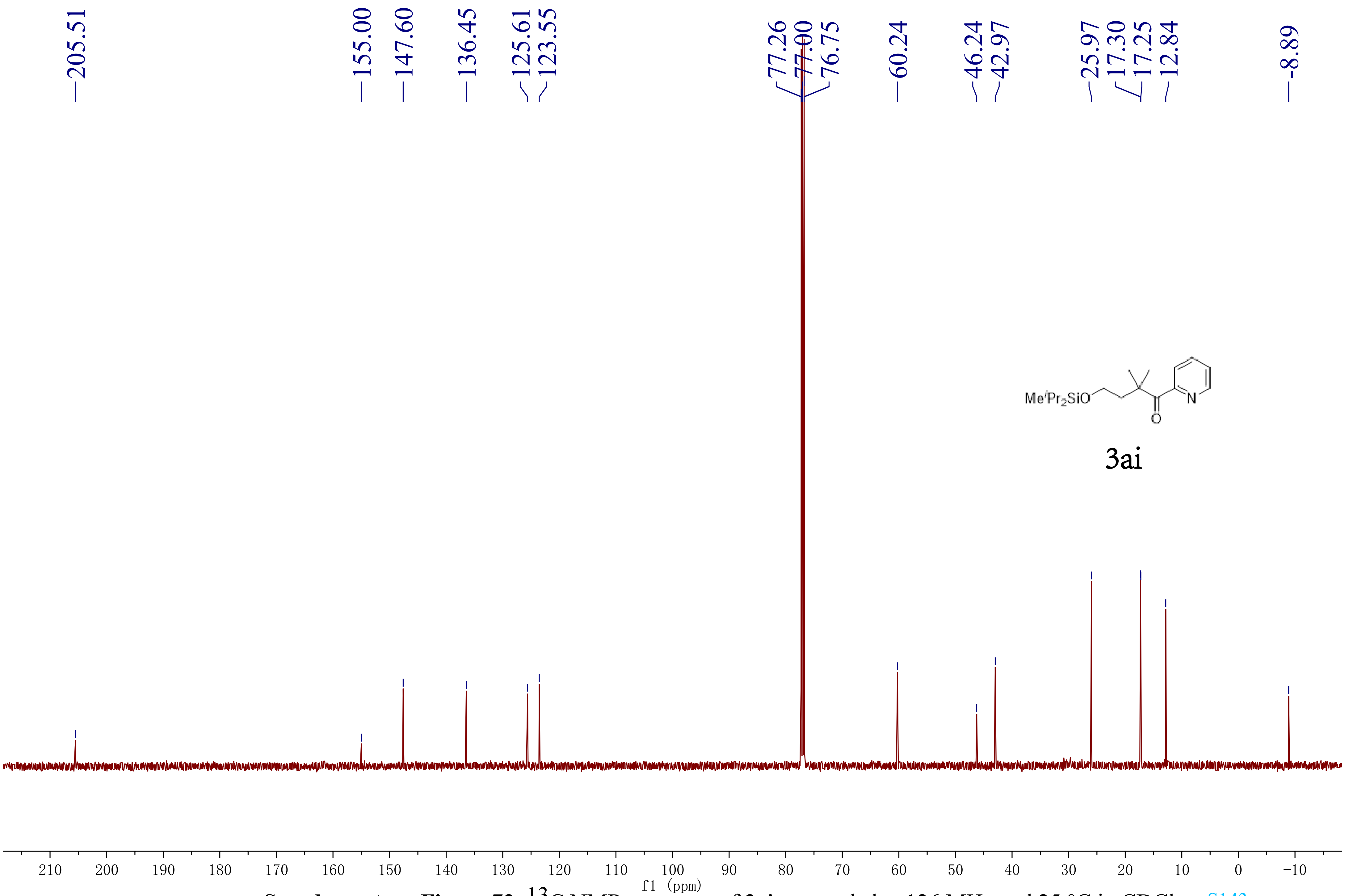

Supplementary Figure 72. ^{13}C NMR spectrum of **3ai**, recorded at 126 MHz and 25 °C in $CDCl_3$

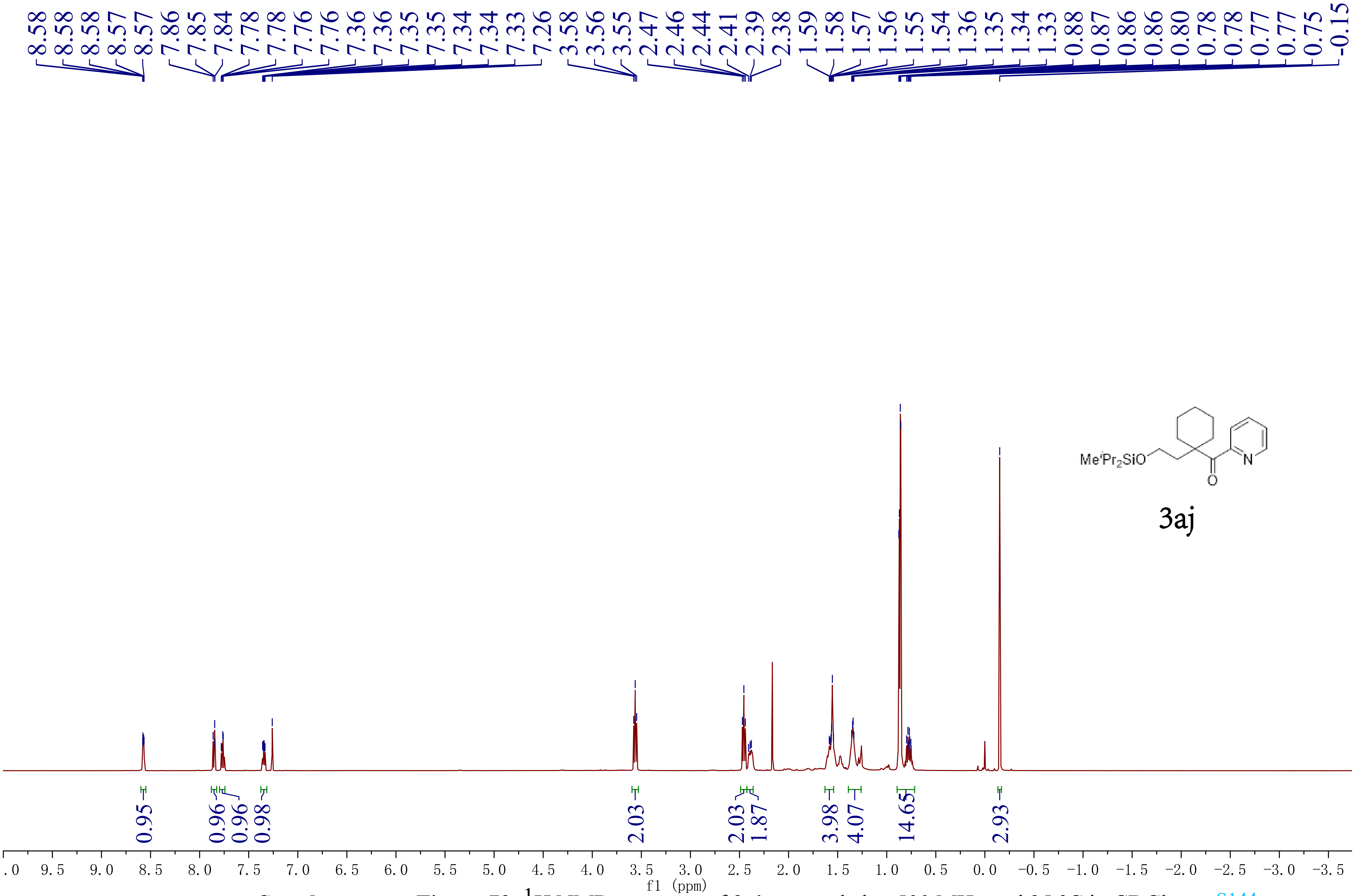

Supplementary Figure 73. ^{1}H NMR spectrum of **3aj**, recorded at 500 MHz and 25 °C in $CDCl_3$

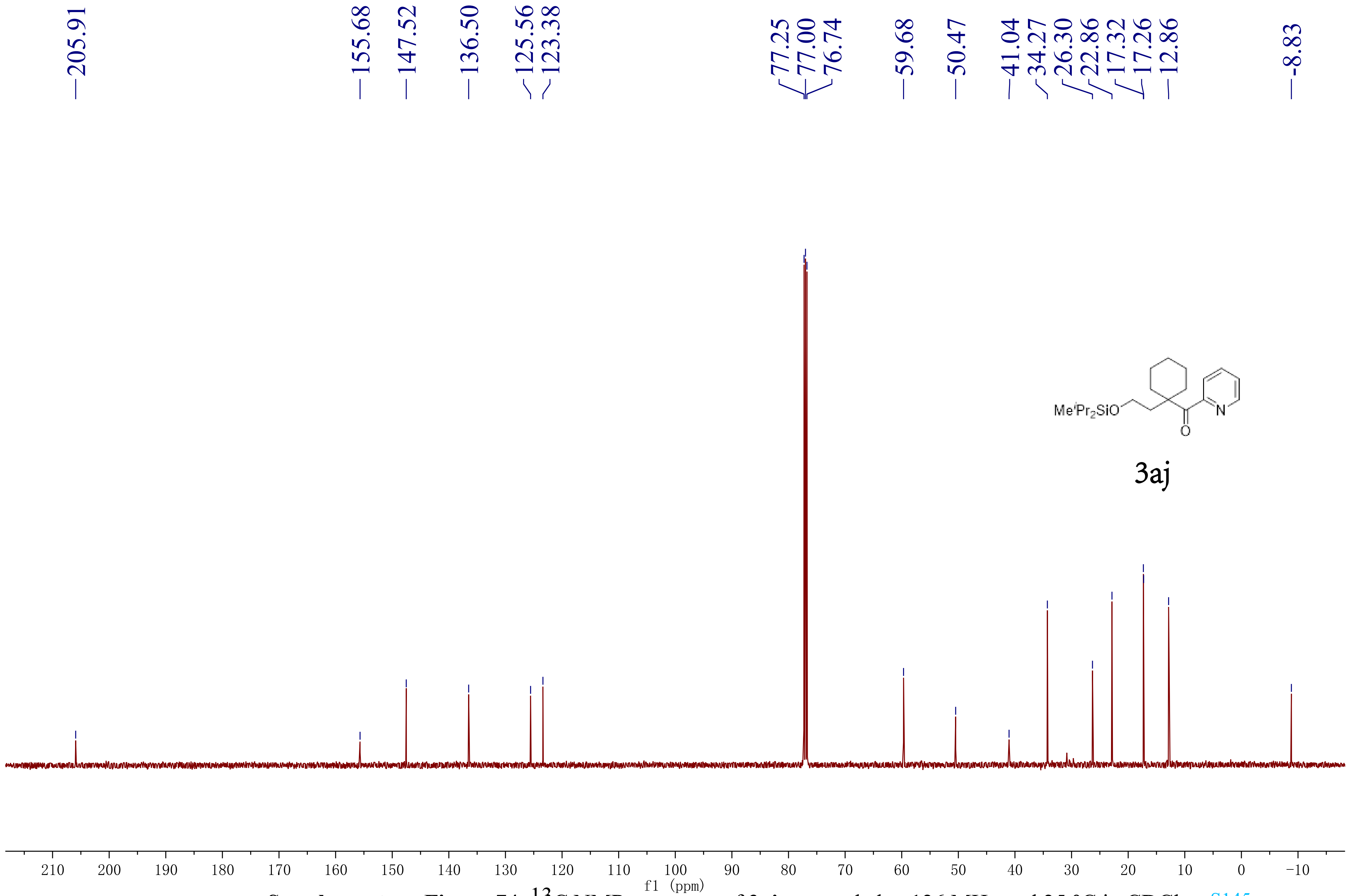

Supplementary Figure 74. ^{13}C NMR spectrum of **3aj**, recorded at 126 MHz and 25 °C in $CDCl_3$

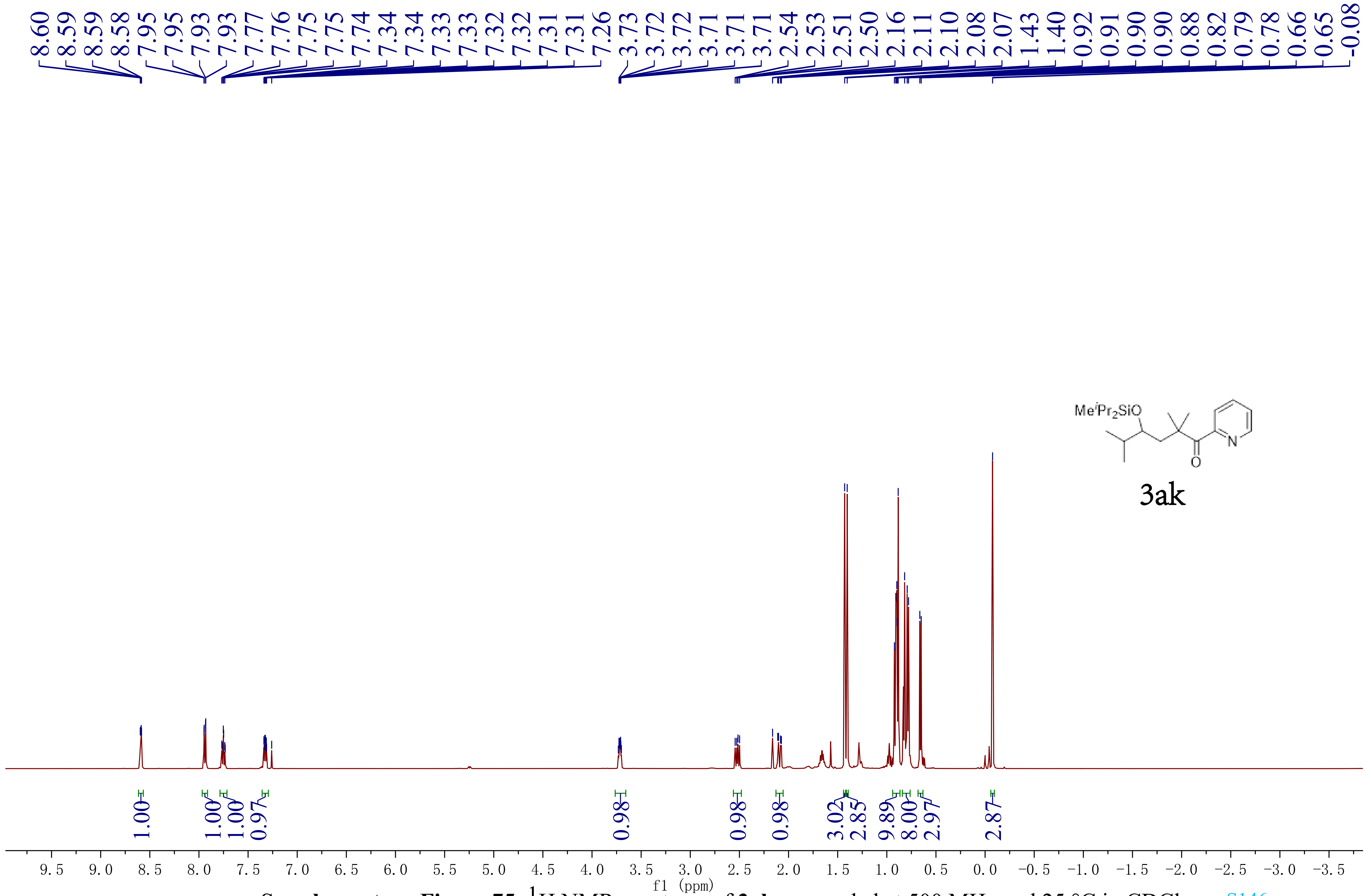

Supplementary Figure 75. ^{1}H NMR spectrum of **3ak**, recorded at 500 MHz and 25 °C in $CDCl_3$

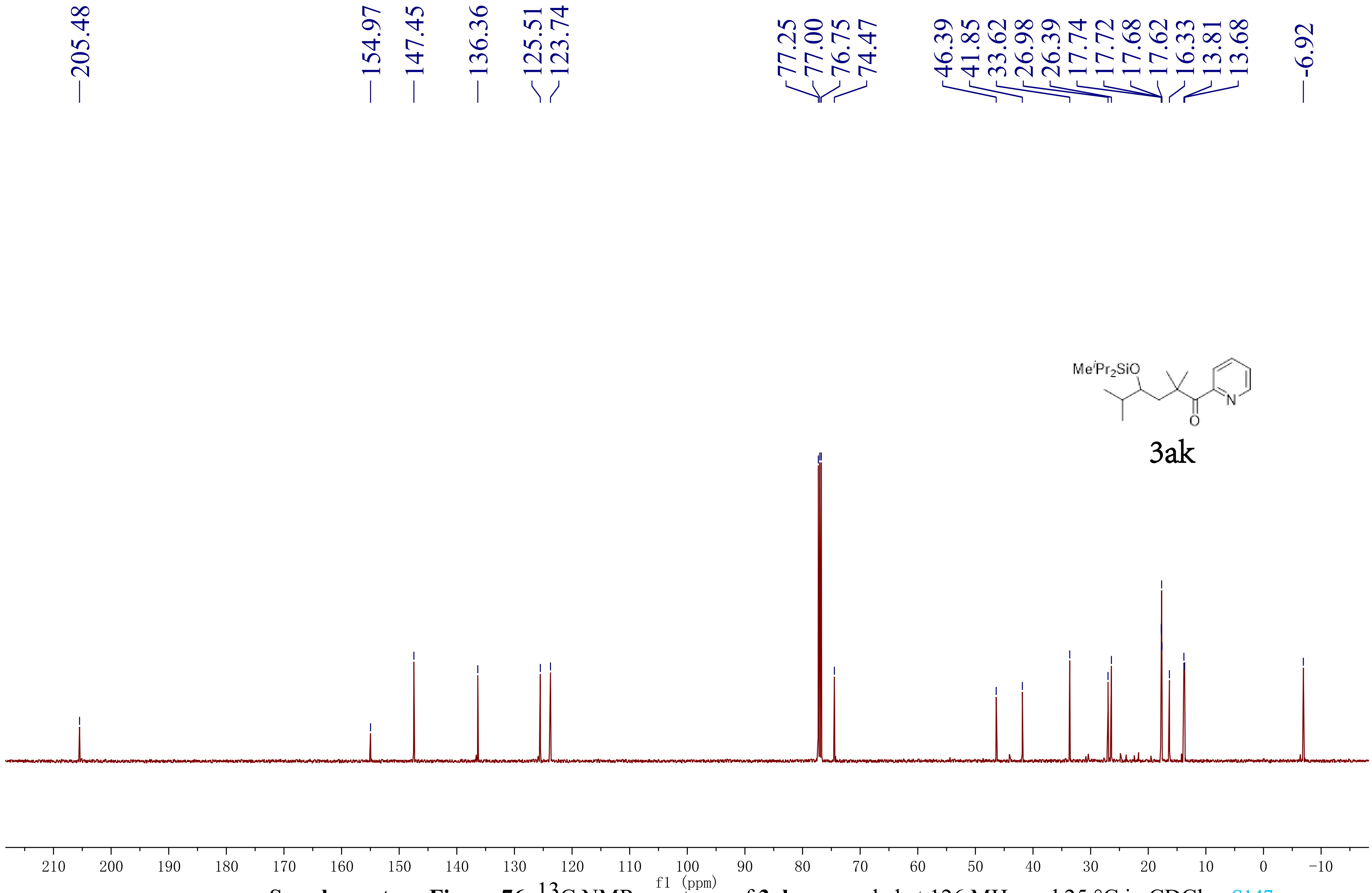

Supplementary Figure 76. ^{13}C NMR spectrum of **3ak**, recorded at 126 MHz and 25 °C in $CDCl_3$

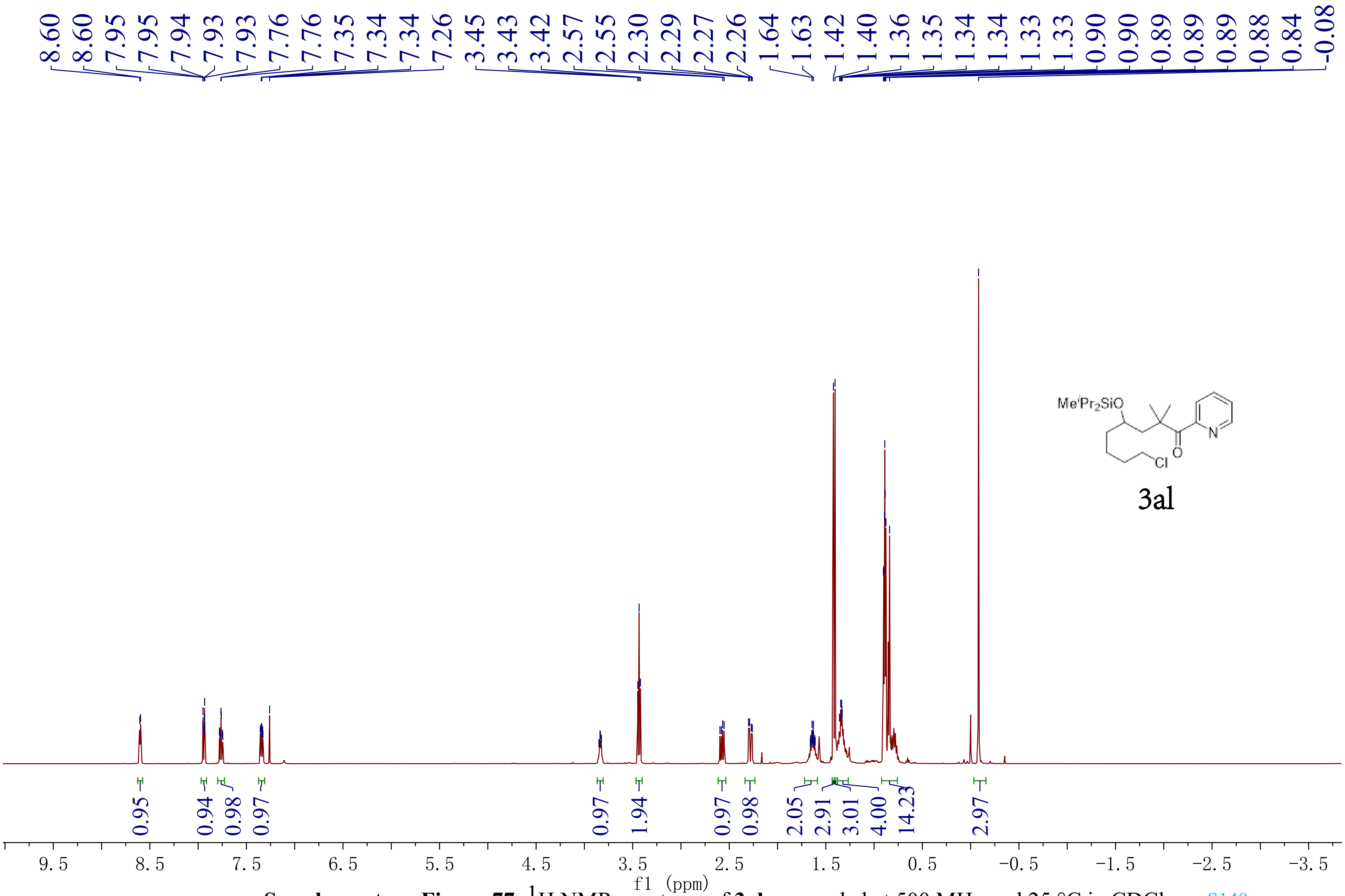

Supplementary Figure 77. ^{1}H NMR spectrum of **3al**, recorded at 500 MHz and 25 °C in $CDCl_3$

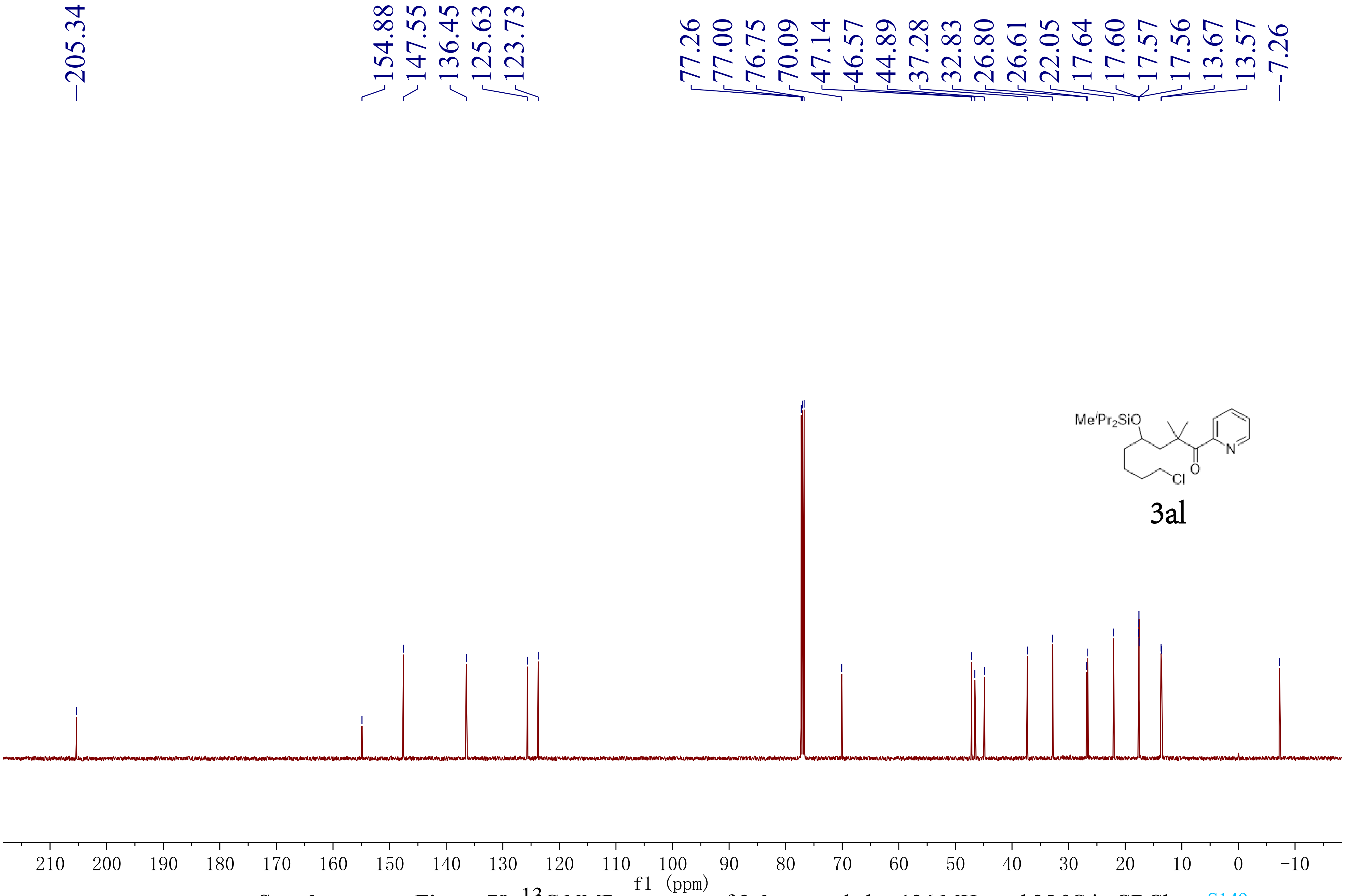

Supplementary Figure 78. ^{13}C NMR spectrum of **3al**, recorded at 126 MHz and 25 °C in $CDCl_3$

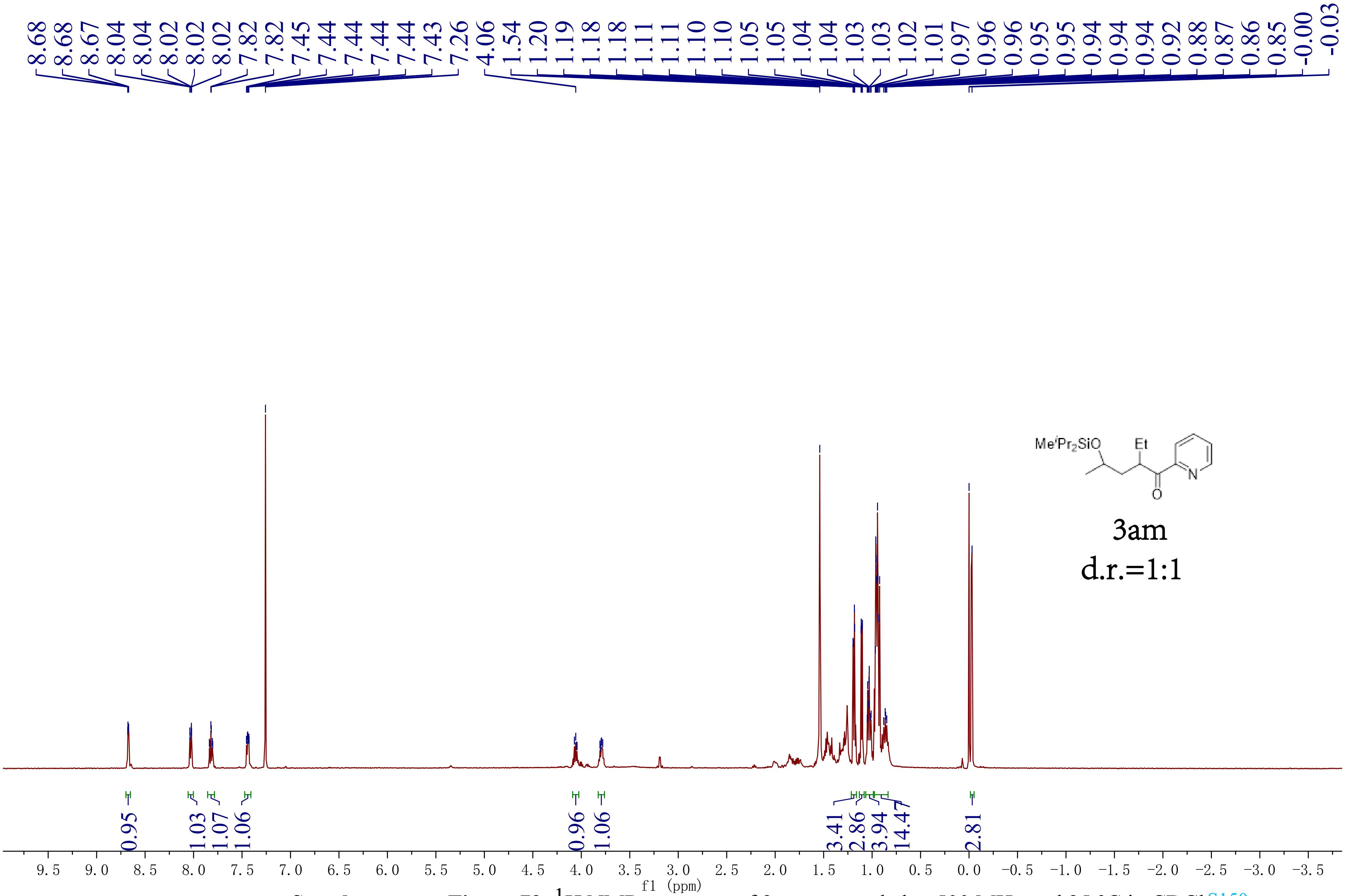

Supplementary Figure 79. ^{1}H NMR spectrum of **3am**, recorded at 500 MHz and 25 °C in $CDCl_3$

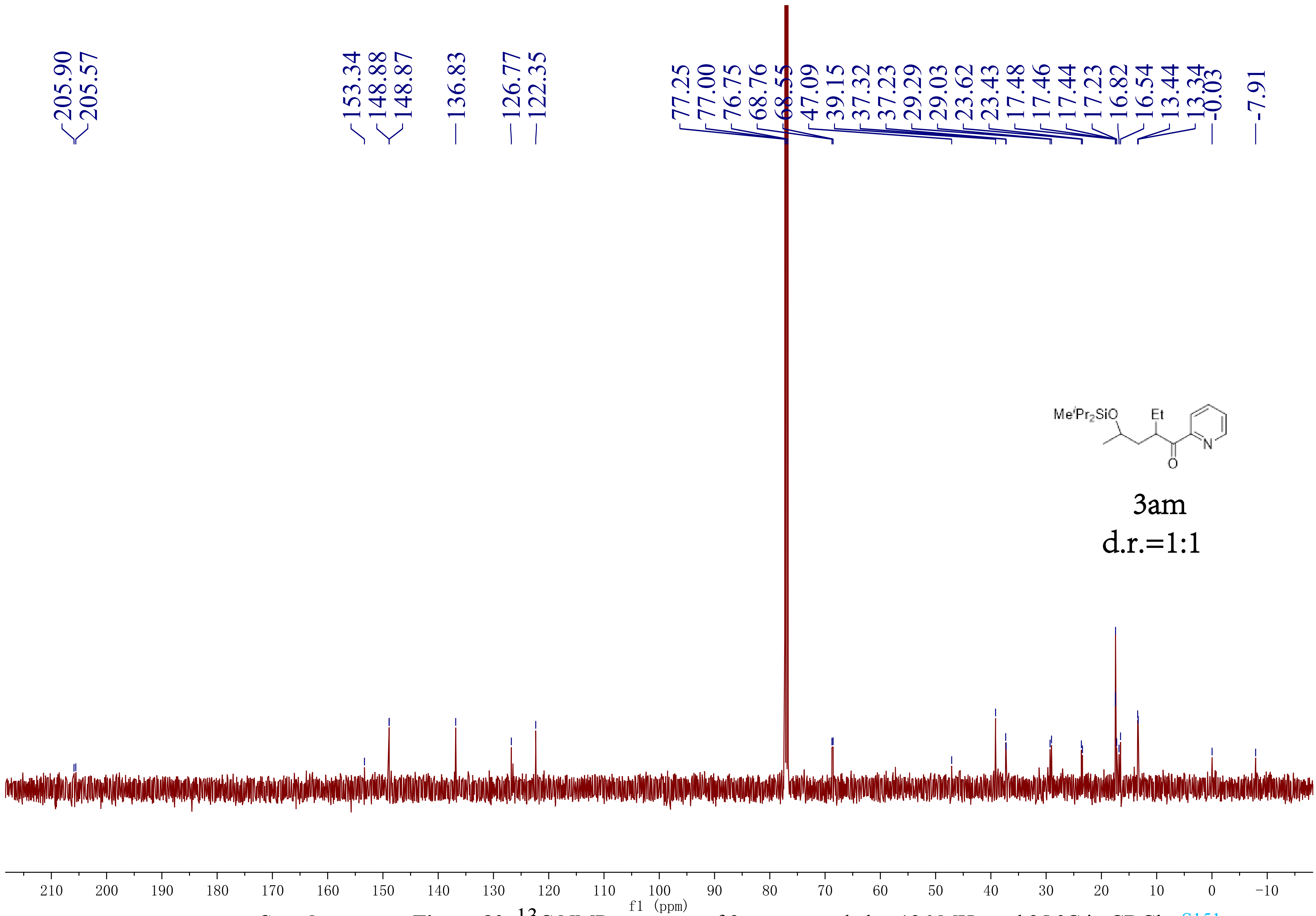

Supplementary Figure 80. ^{13}C NMR spectrum of **3am**, recorded at 126 MHz and 25 °C in $CDCl_3$

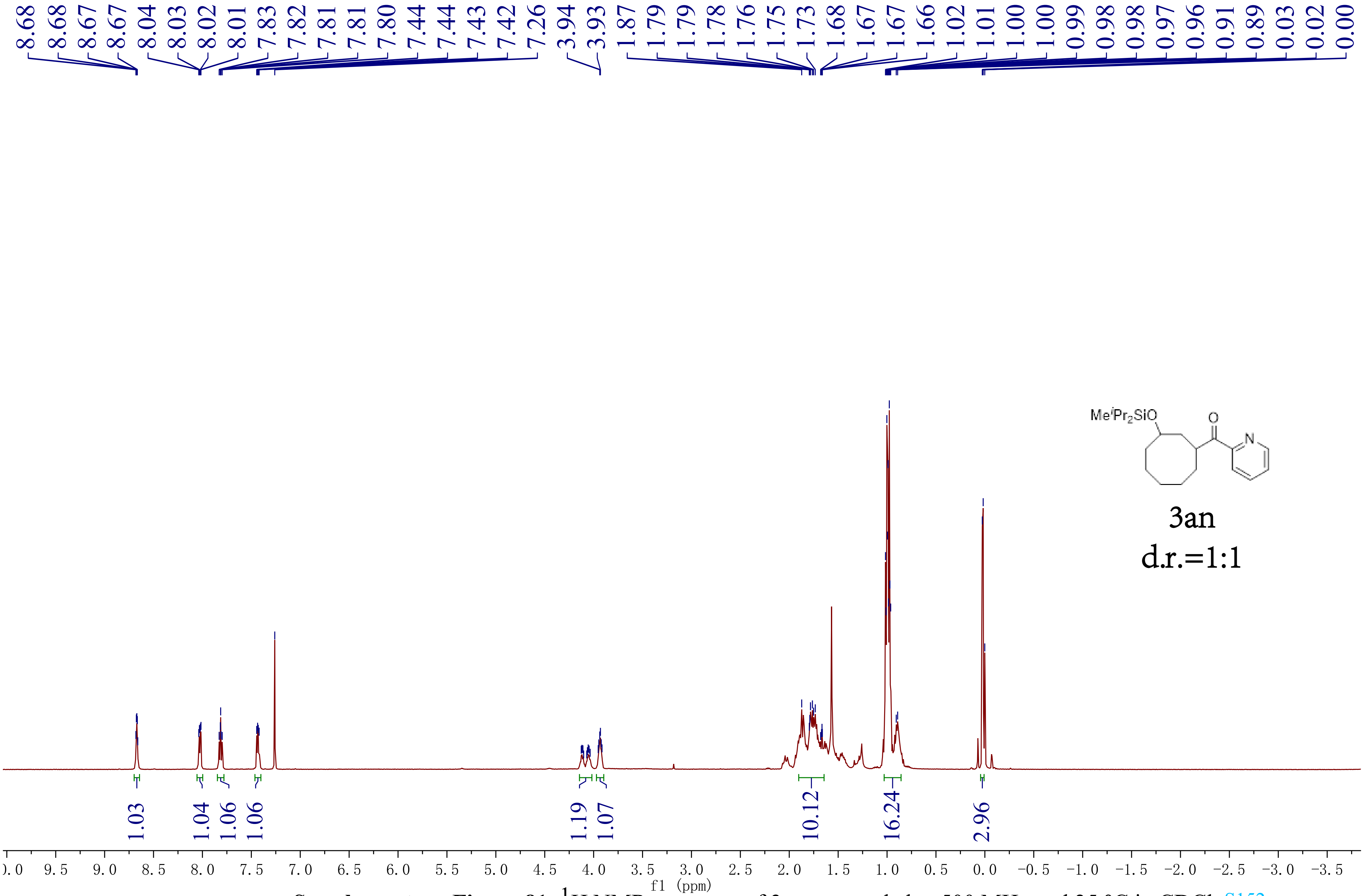

Supplementary Figure 81. ^{1}H NMR spectrum of **3an**, recorded at 500 MHz and 25 °C in $CDCl_3$

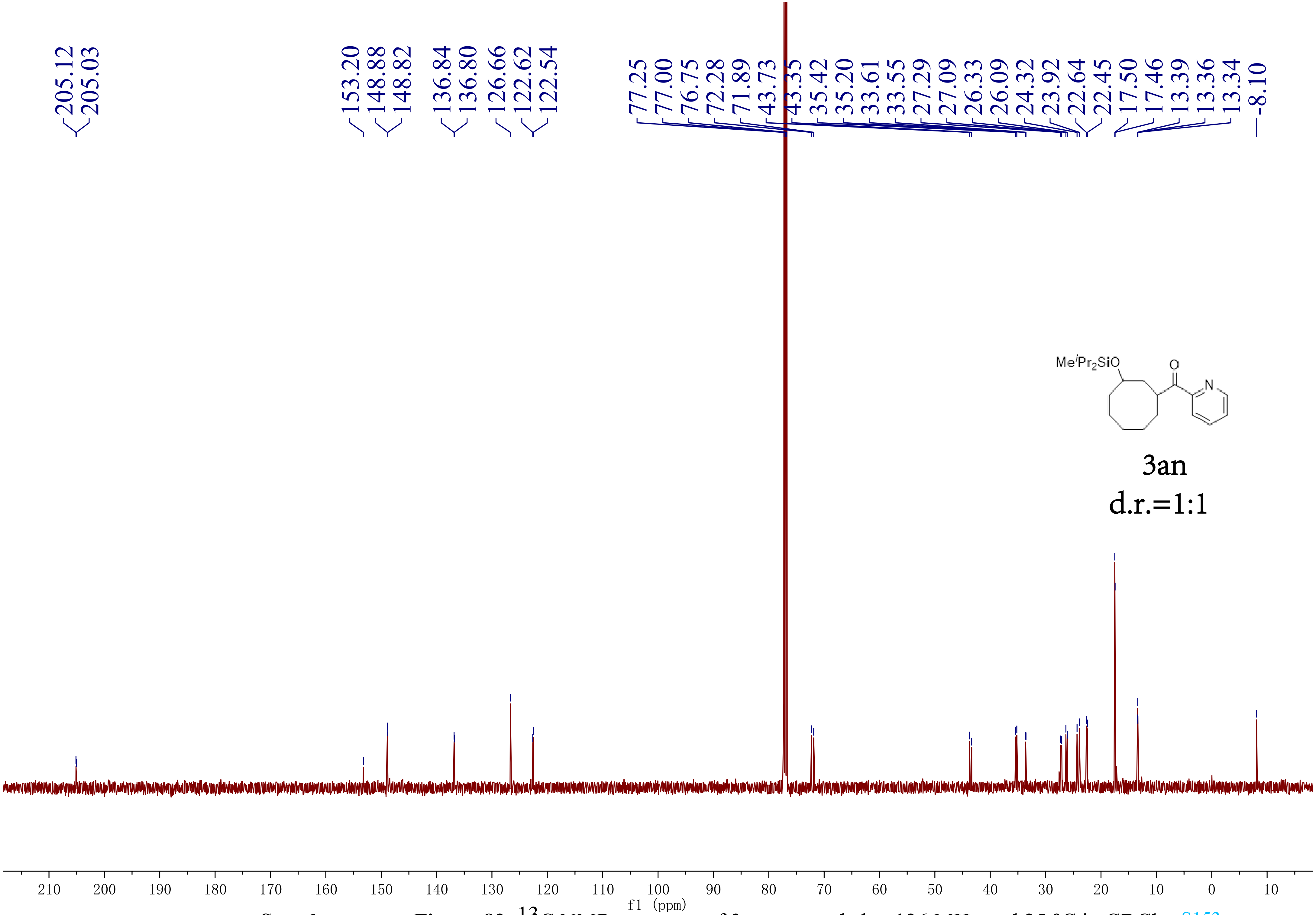

3an
d.r.=1:1

Supplementary Figure 82. ^{13}C NMR spectrum of **3an**, recorded at 126 MHz and 25 °C in $CDCl_3$

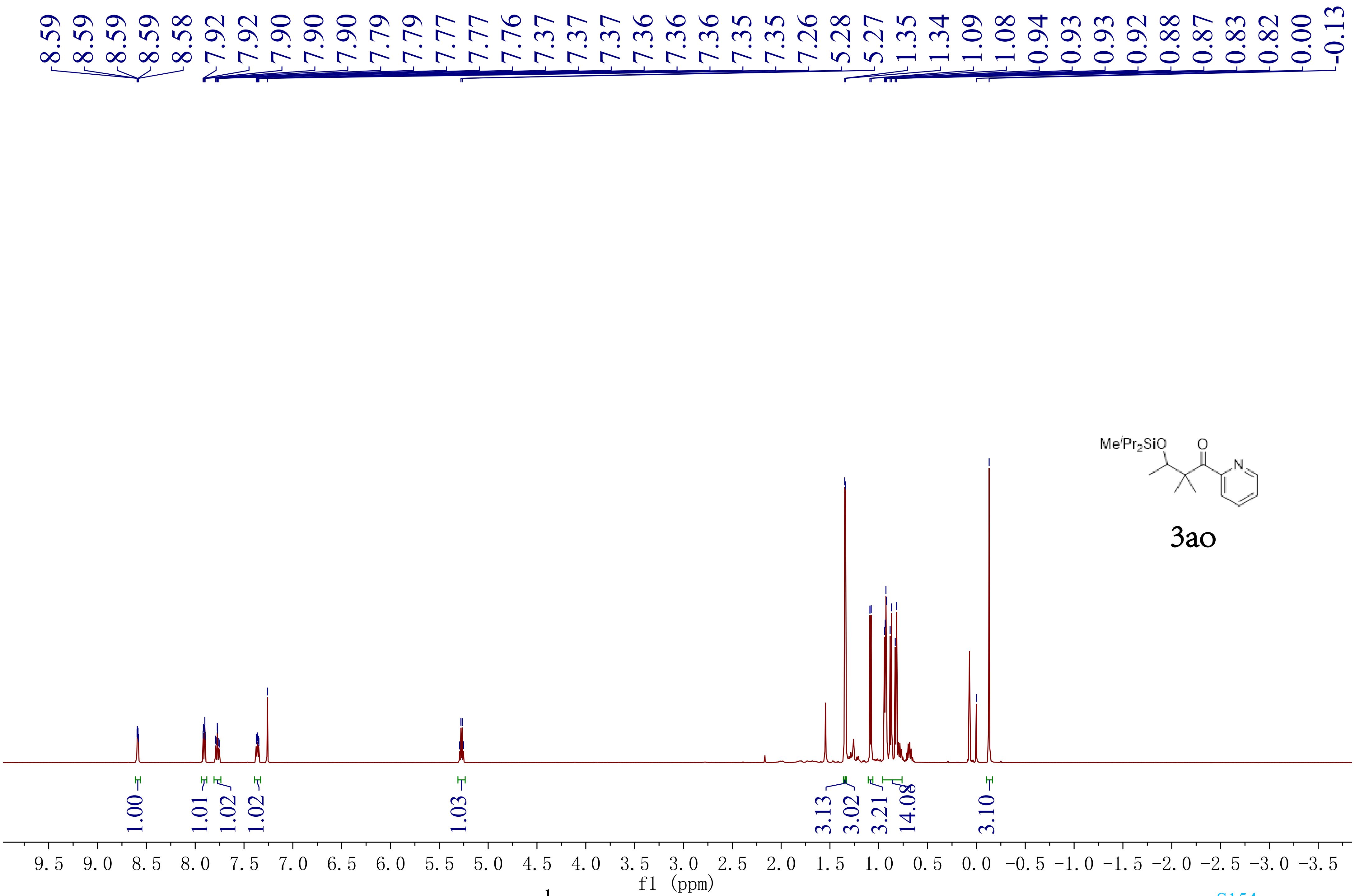

Supplementary Figure 83. ^{1}H NMR spectrum of **3ao**, recorded at 500 MHz and 25 °C in $CDCl_3$

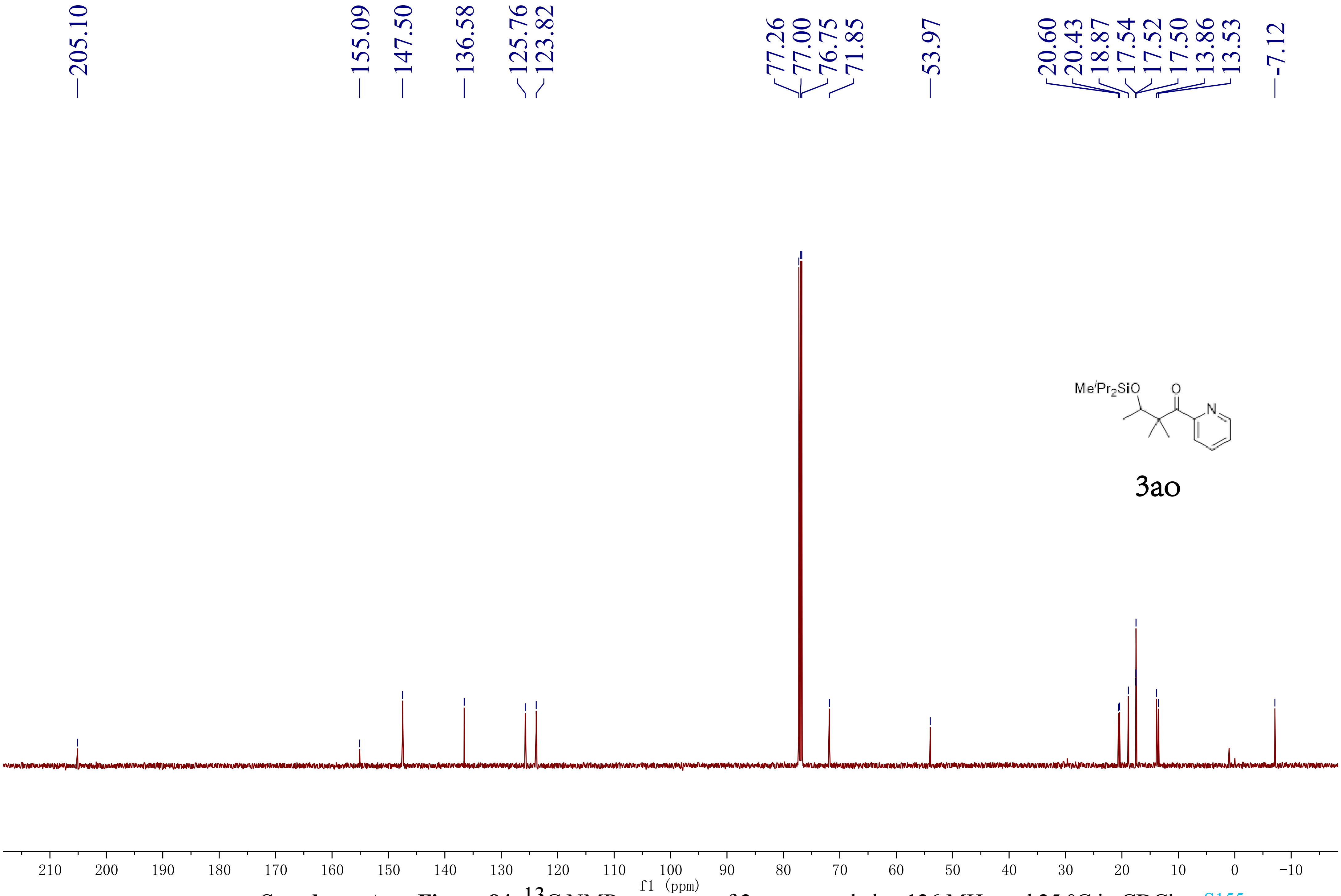

Supplementary Figure 84. ^{13}C NMR spectrum of **3ao**, recorded at 126 MHz and 25 °C in $CDCl_3$

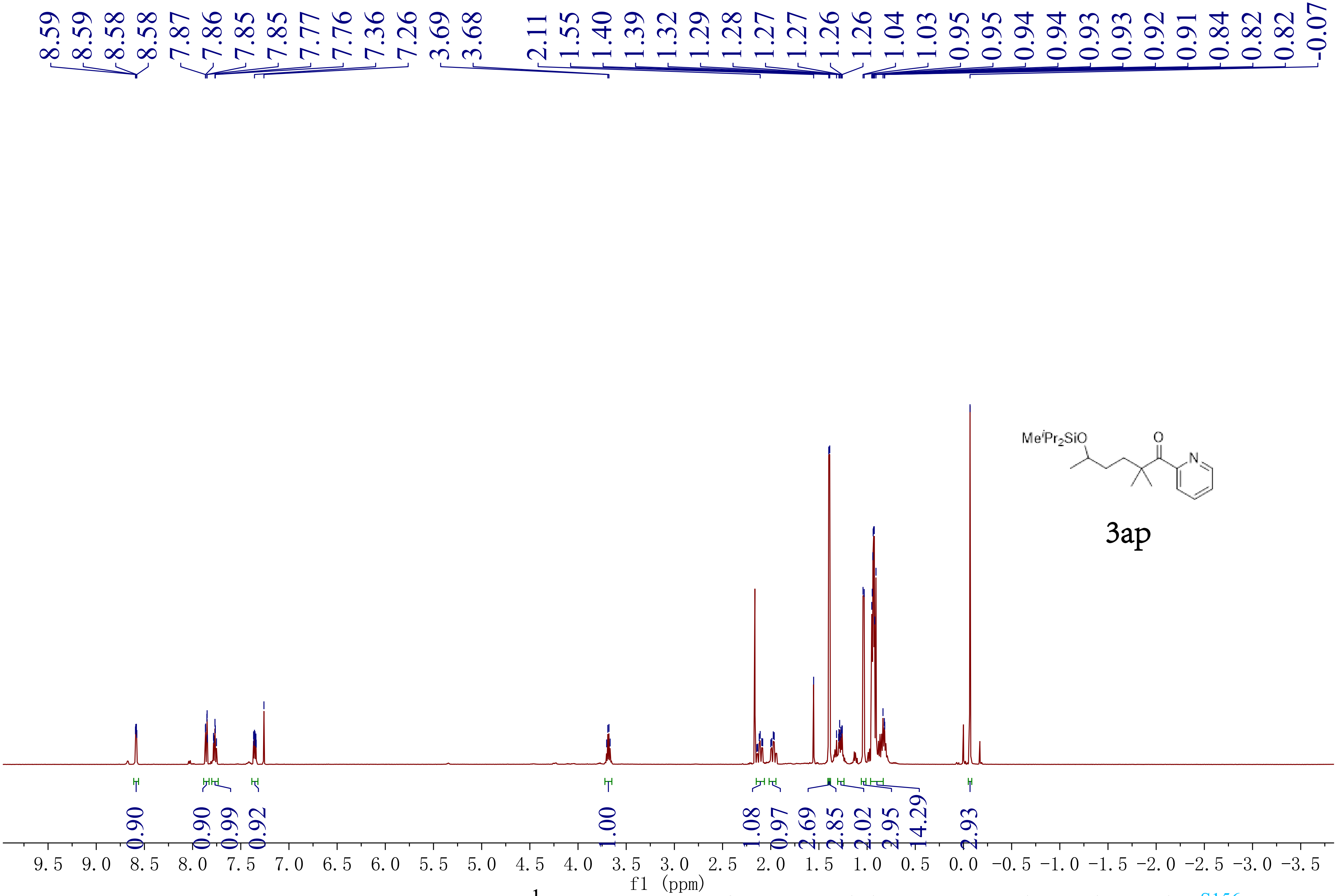

Supplementary Figure 85. ^{1}H NMR spectrum of **3ap**, recorded at 500 MHz and 25 °C in $CDCl_3$

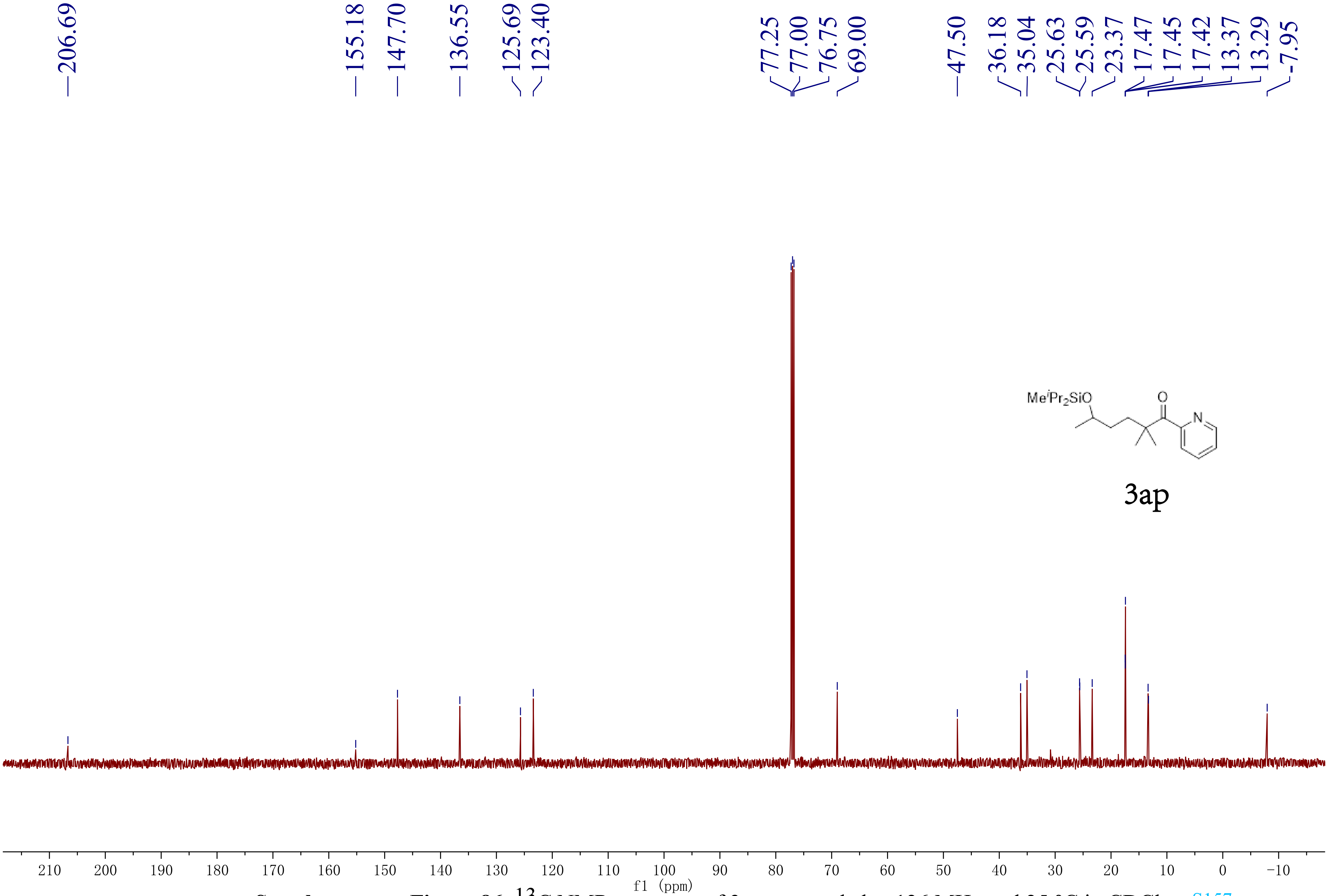

Supplementary Figure 86. ^{13}C NMR spectrum of **3ap**, recorded at 126 MHz and 25 °C in $CDCl_3$

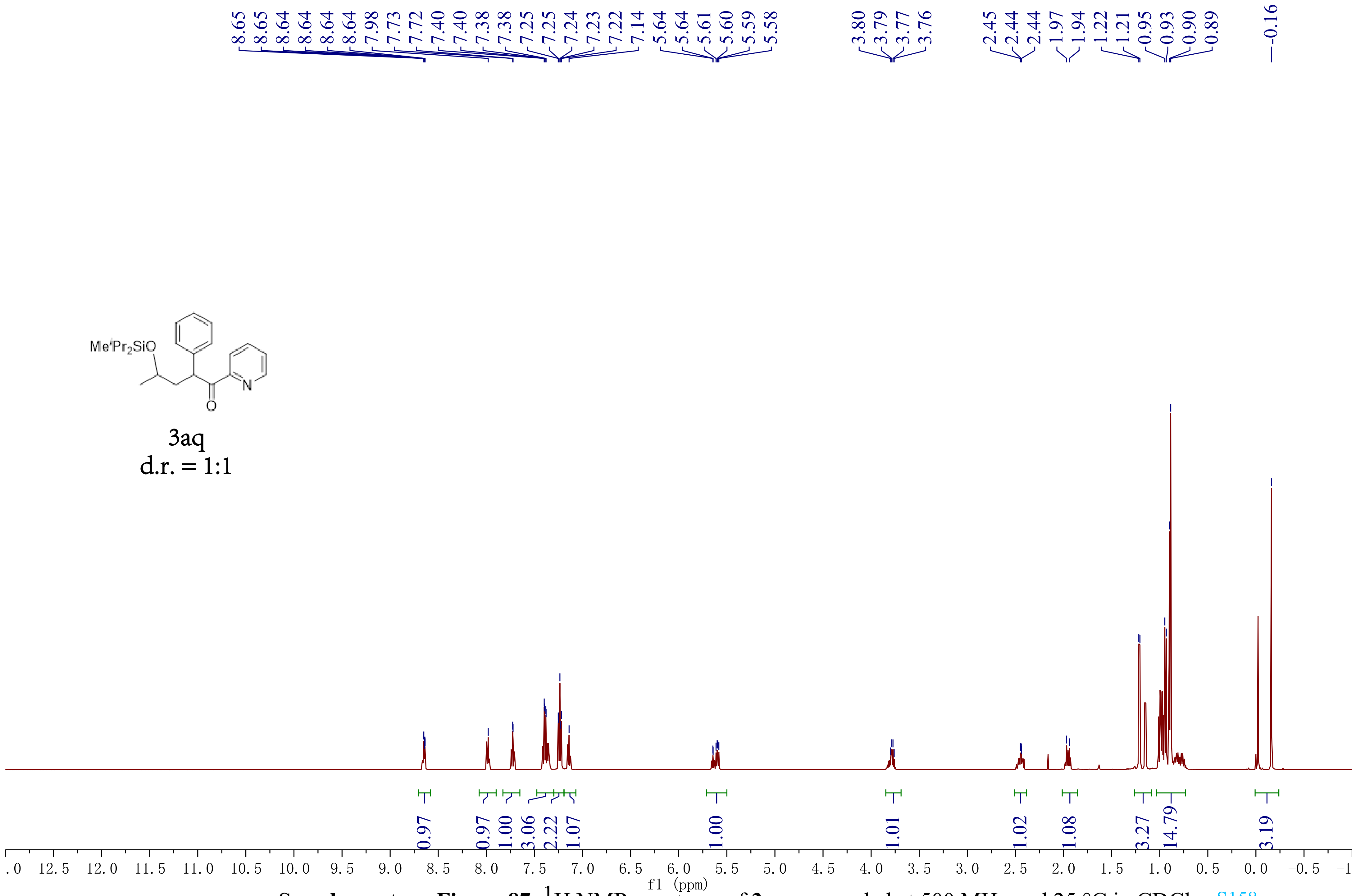

Supplementary Figure 87. ^{1}H NMR spectrum of **3aq**, recorded at 500 MHz and 25 °C in $CDCl_3$

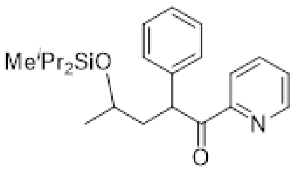

3aq
d.r. = 1:1

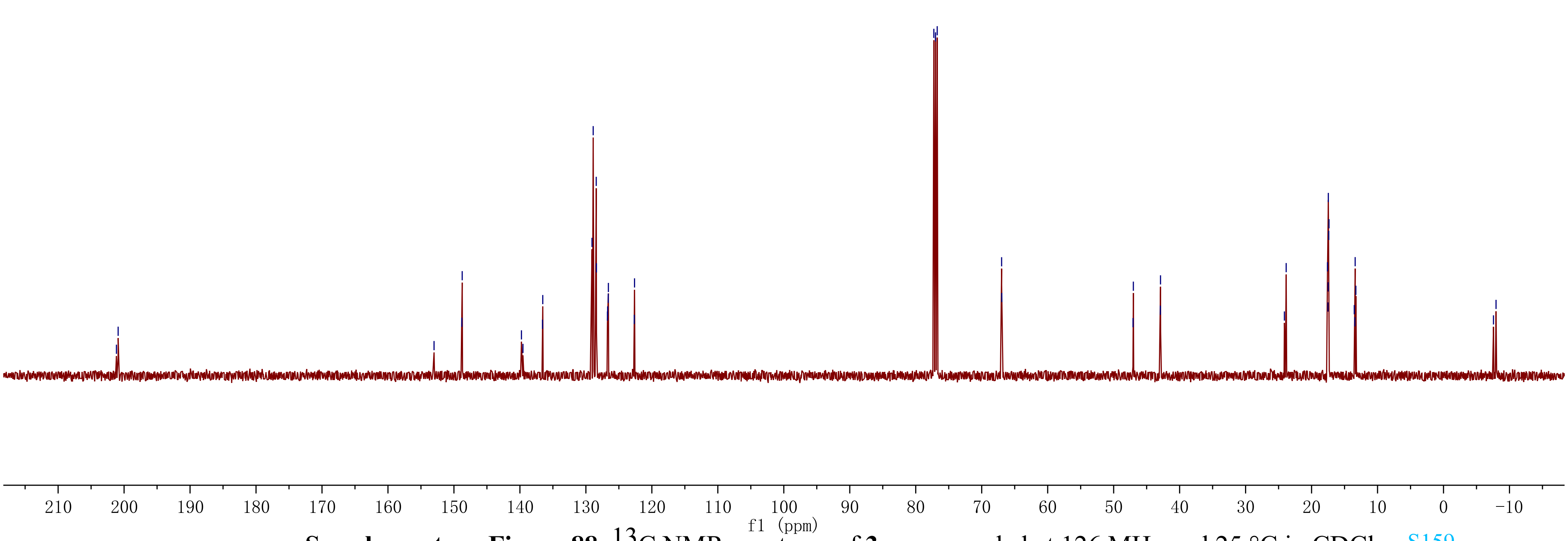

Supplementary Figure 88. ^{13}C NMR spectrum of **3aq**, recorded at 126 MHz and 25 °C in $CDCl_3$

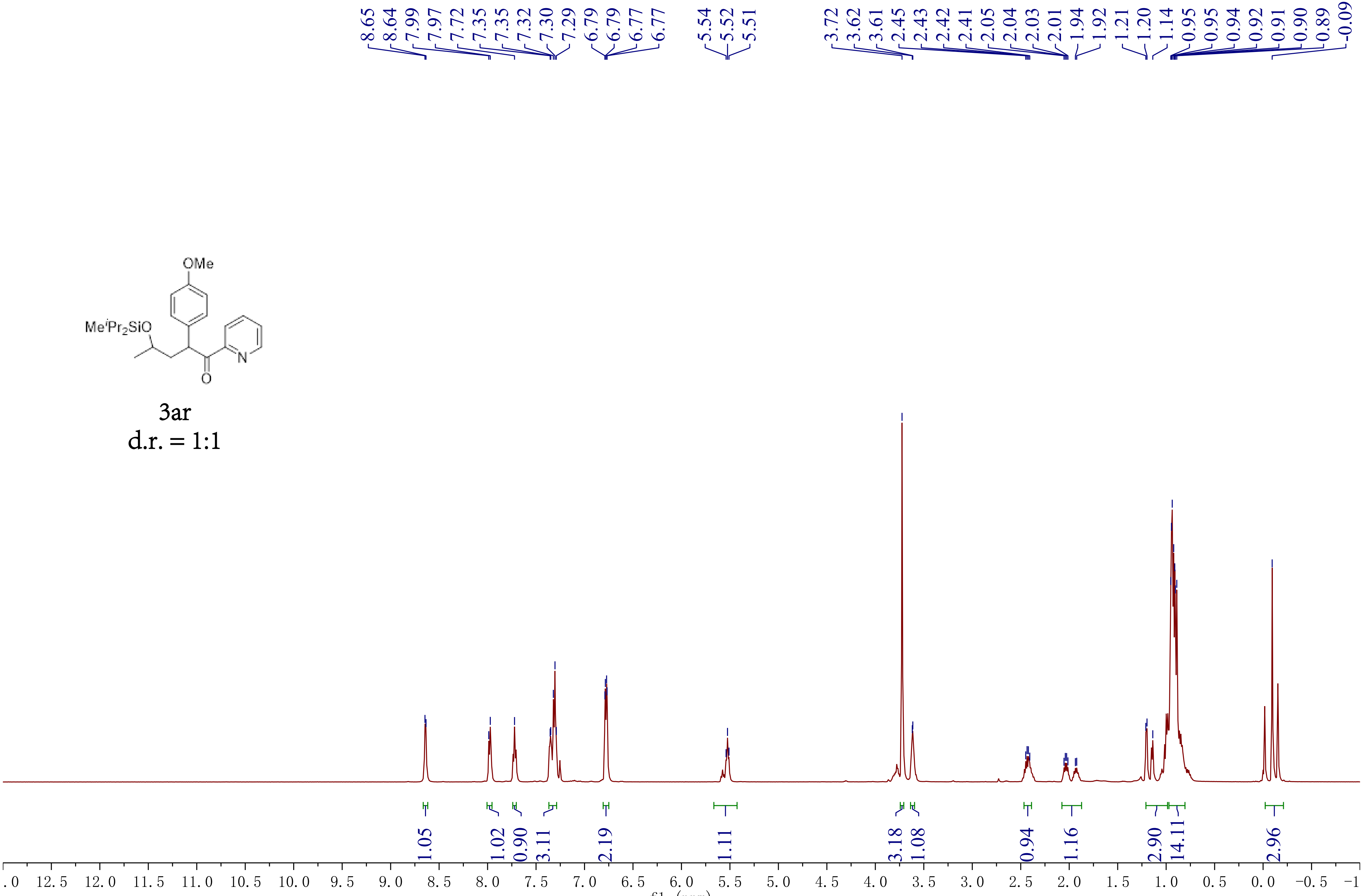

Supplementary Figure 89. ^{1}H NMR spectrum of **3ar**, recorded at 500 MHz and 25 °C in $CDCl_3$

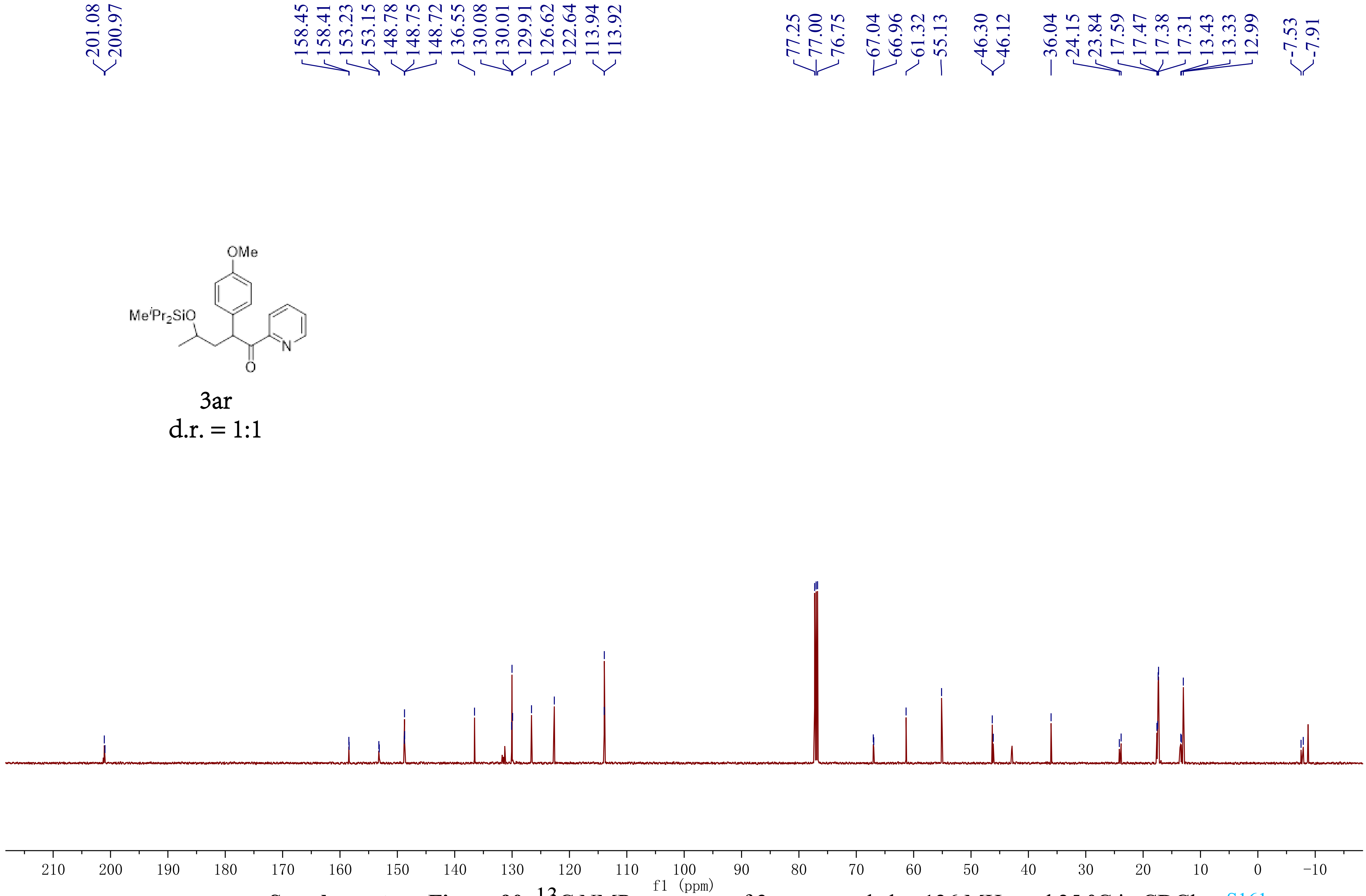

Supplementary Figure 90. ^{13}C NMR spectrum of **3ar**, recorded at 126 MHz and 25 °C in $CDCl_3$

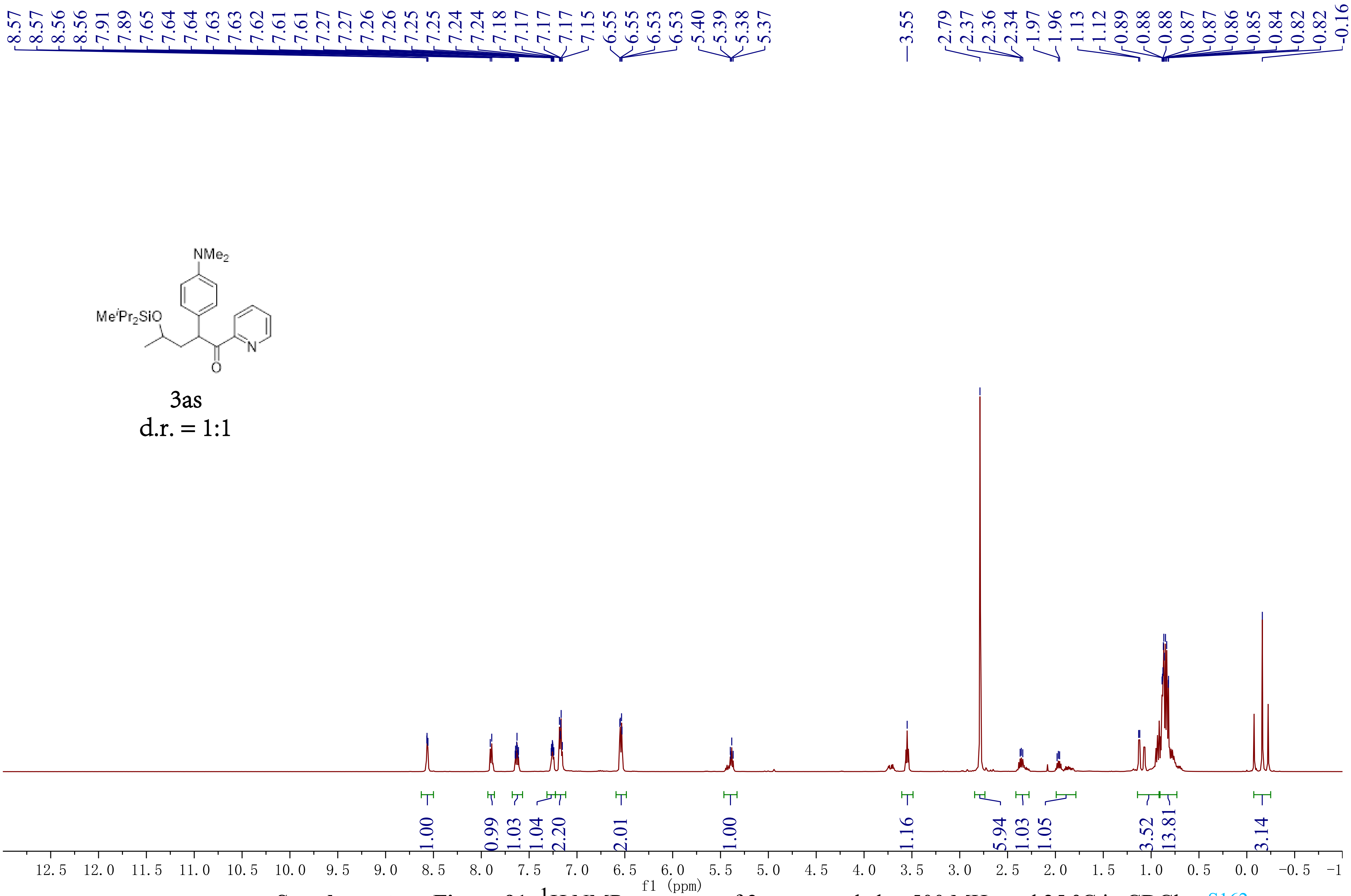

Supplementary Figure 91. ^{1}H NMR spectrum of **3as**, recorded at 500 MHz and 25 °C in $CDCl_3$

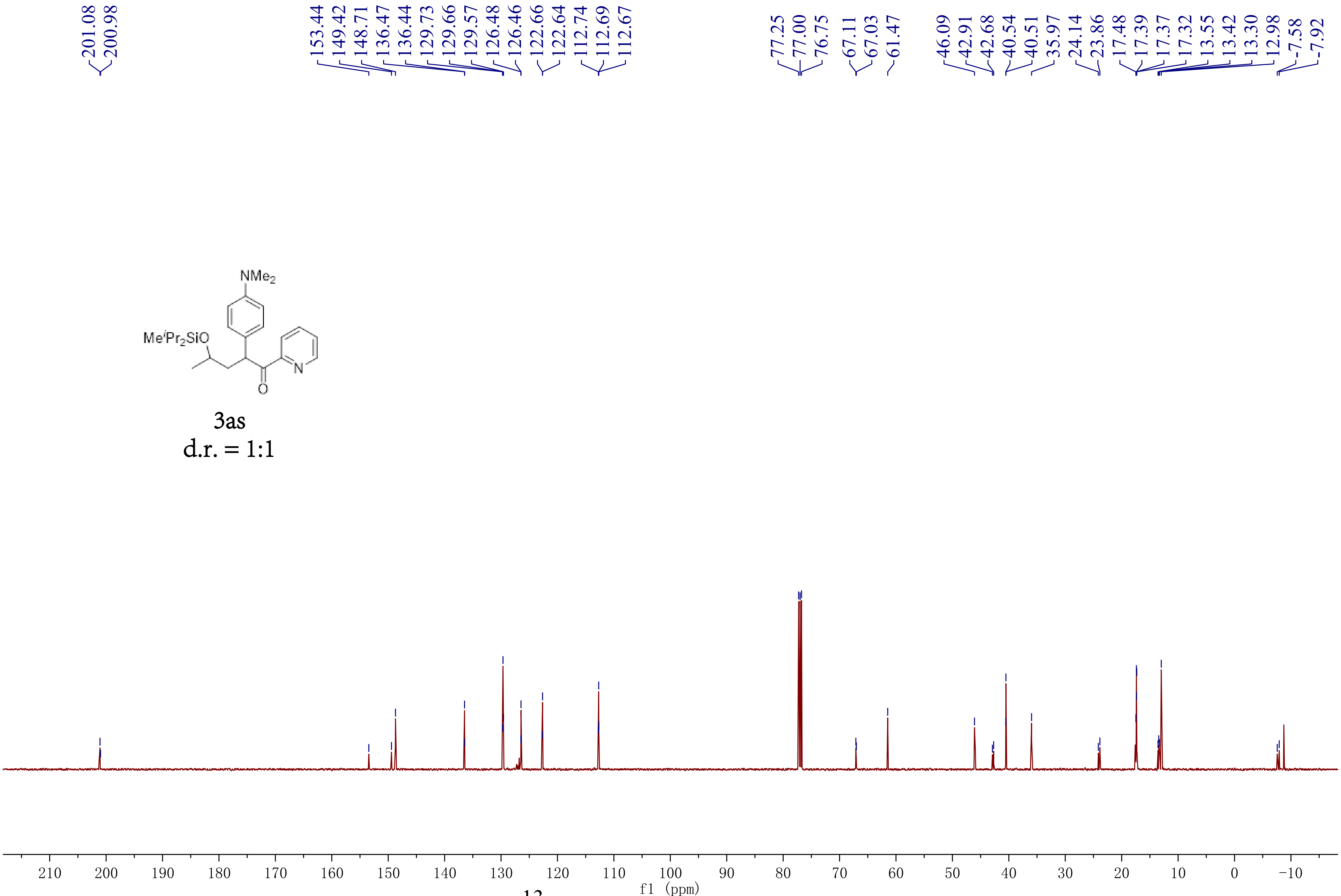

3as

d.r. = 1:1

Supplementary Figure 92. ^{13}C NMR spectrum of **3as**, recorded at 126 MHz and 25 °C in $CDCl_3$

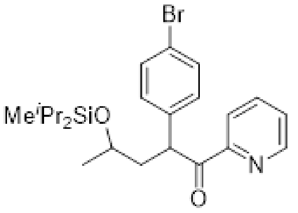

3at
d.r. = 1:1

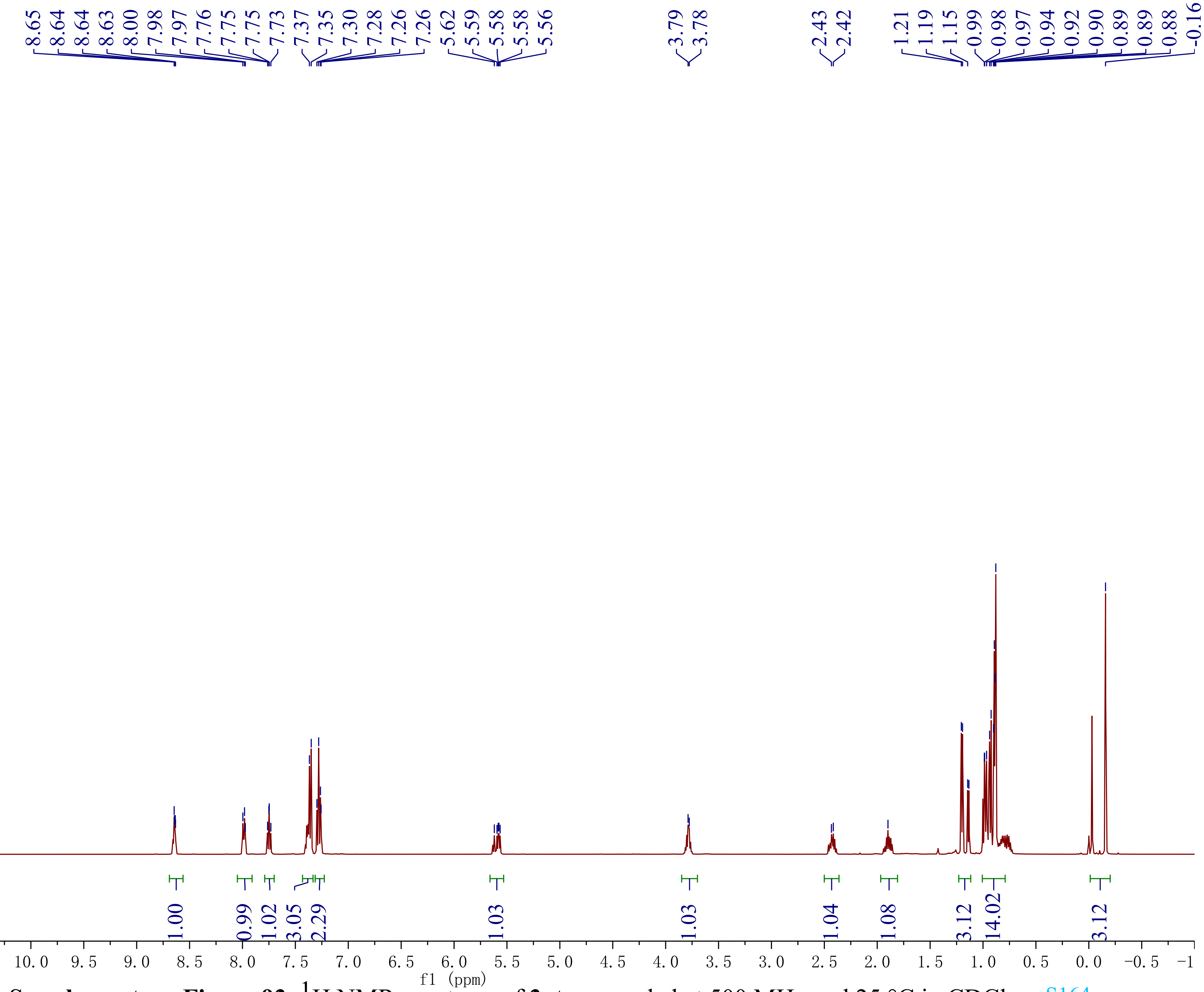

Supplementary Figure 93. ^{1}H NMR spectrum of **3at**, recorded at 500 MHz and 25 °C in $CDCl_3$

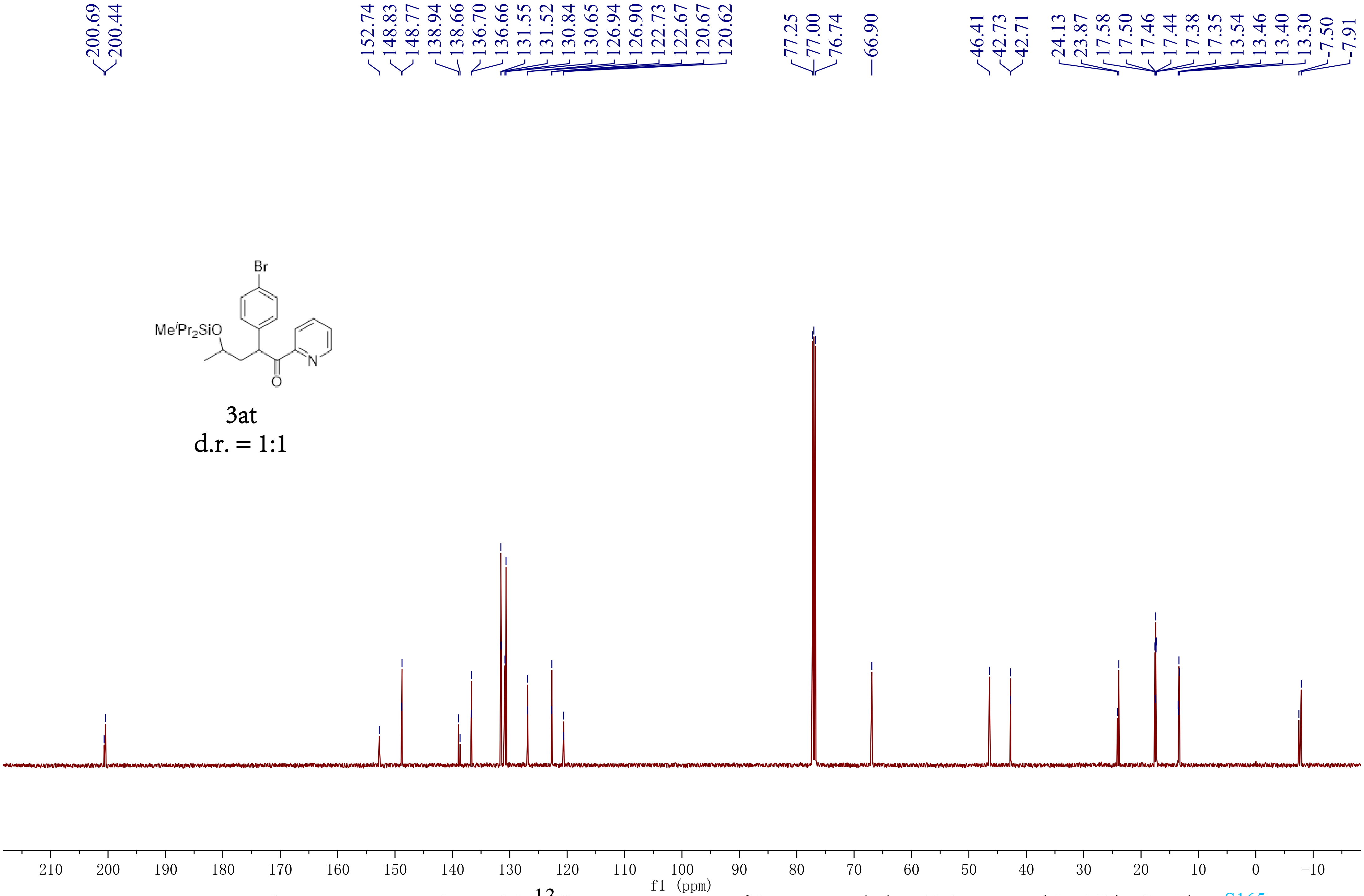

Supplementary Figure 94. ^{13}C NMR spectrum of **3at**, recorded at 126 MHz and 25 °C in $CDCl_3$

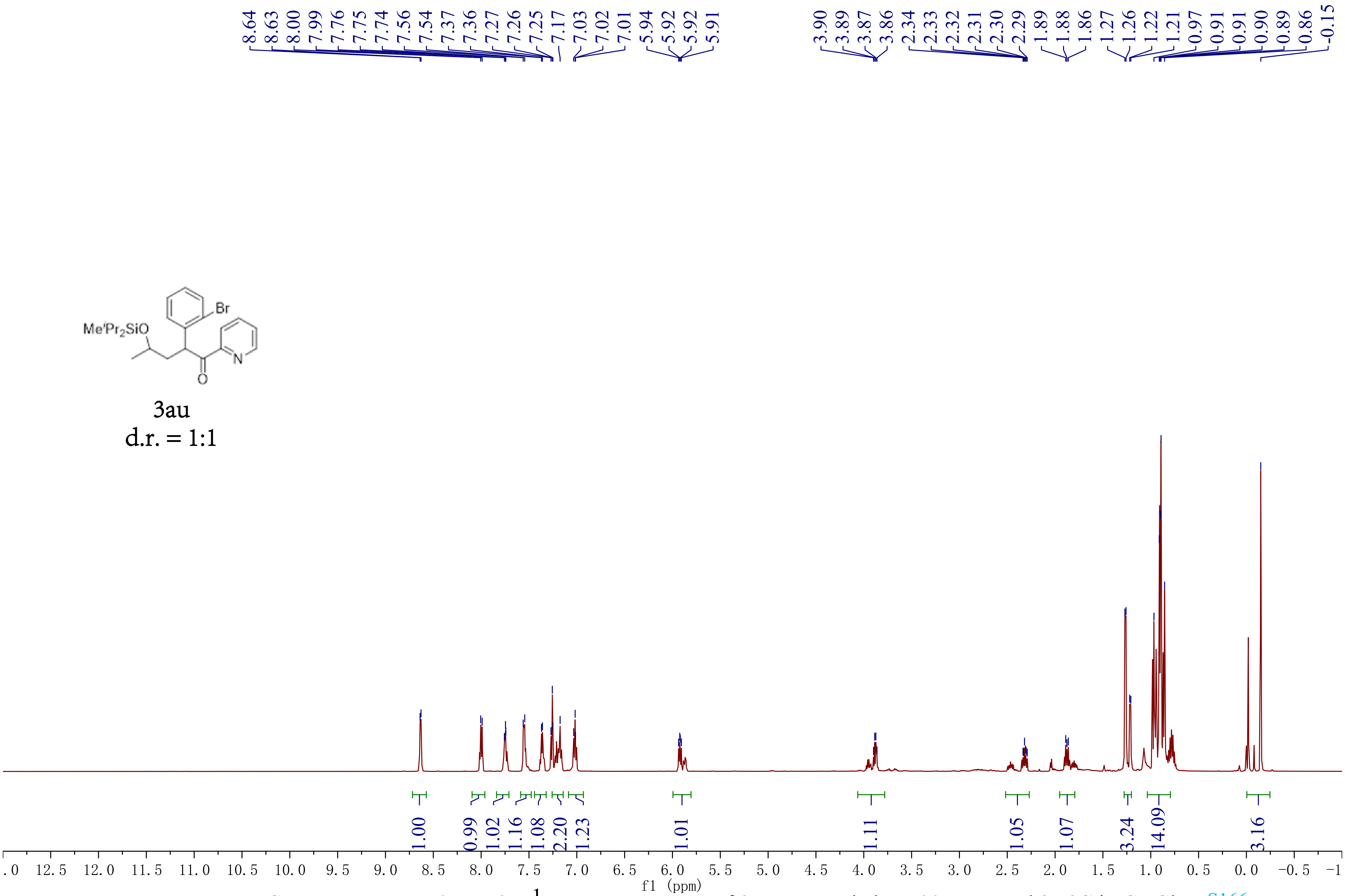

Supplementary Figure 95. ^{1}H NMR spectrum of **3au**, recorded at 500 MHz and 25 °C in $CDCl_3$

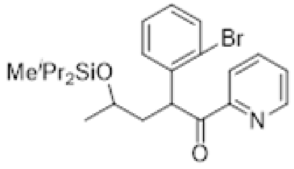

3au
d.r. = 1:1

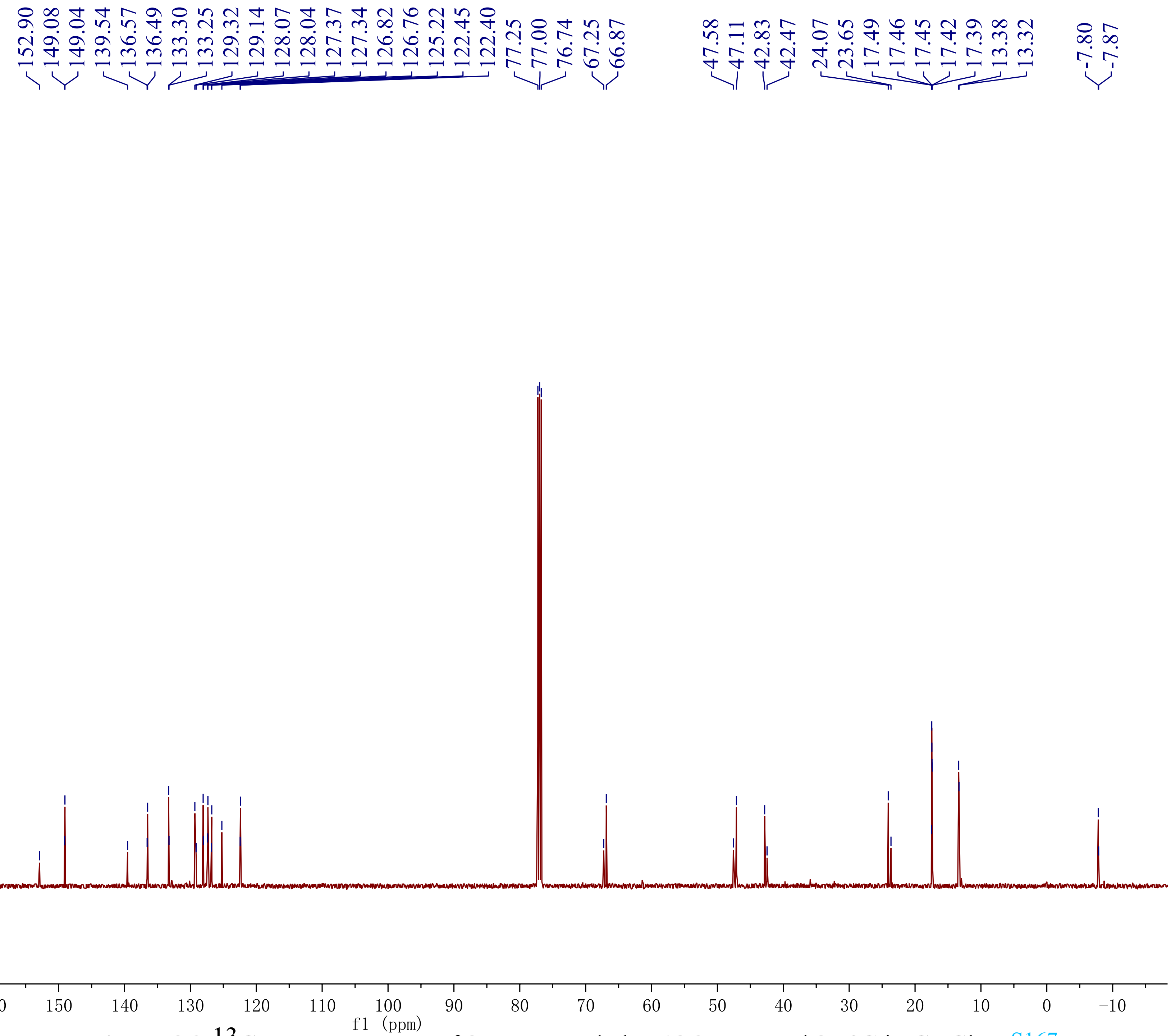

Supplementary Figure 96. ^{13}C NMR spectrum of **3au**, recorded at 126 MHz and 25 °C in $CDCl_3$

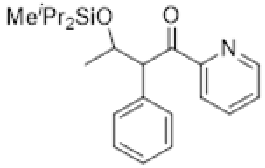

3av

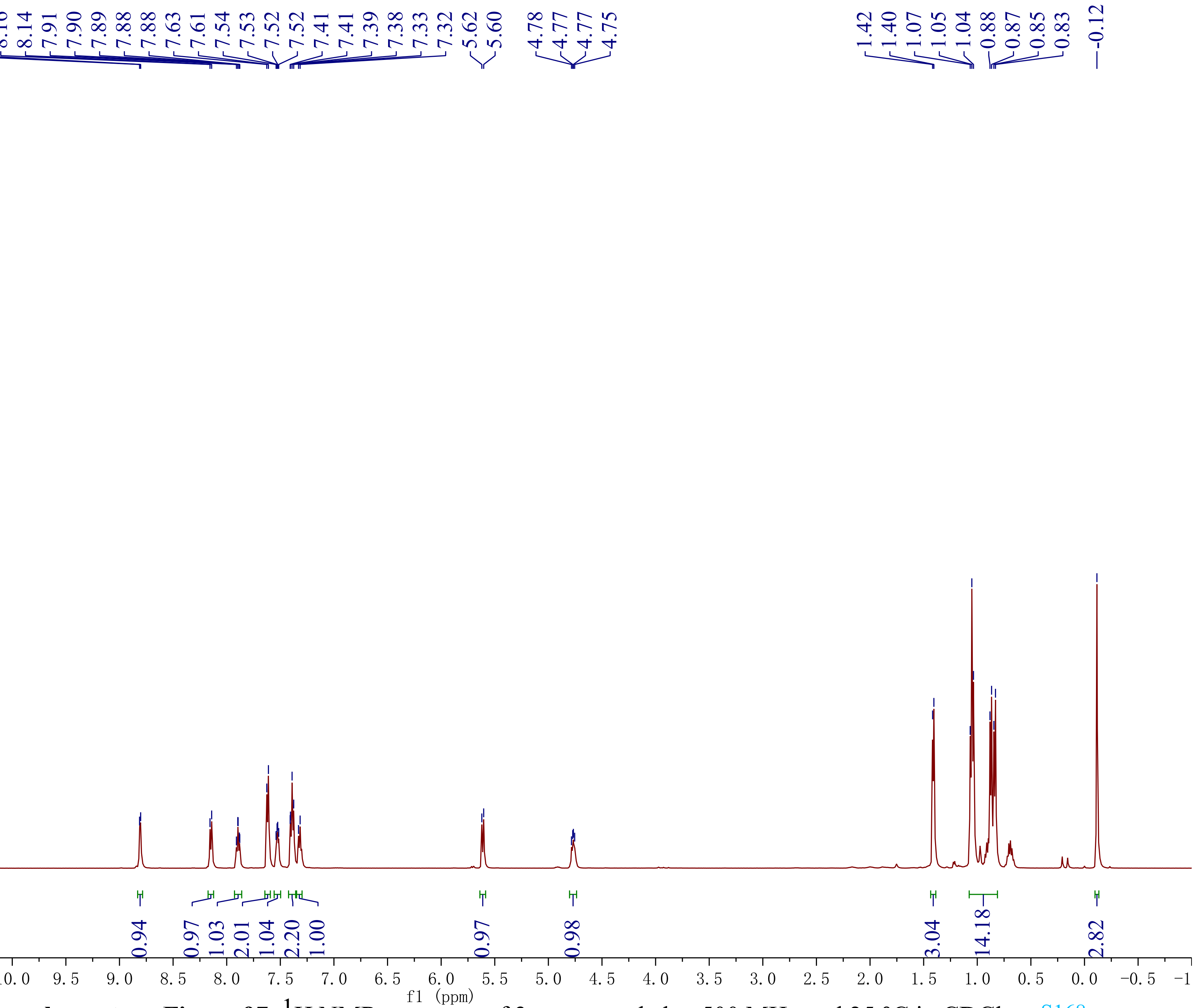

Supplementary Figure 97. ^{1}H NMR spectrum of **3av**, recorded at 500 MHz and 25 °C in $CDCl_3$

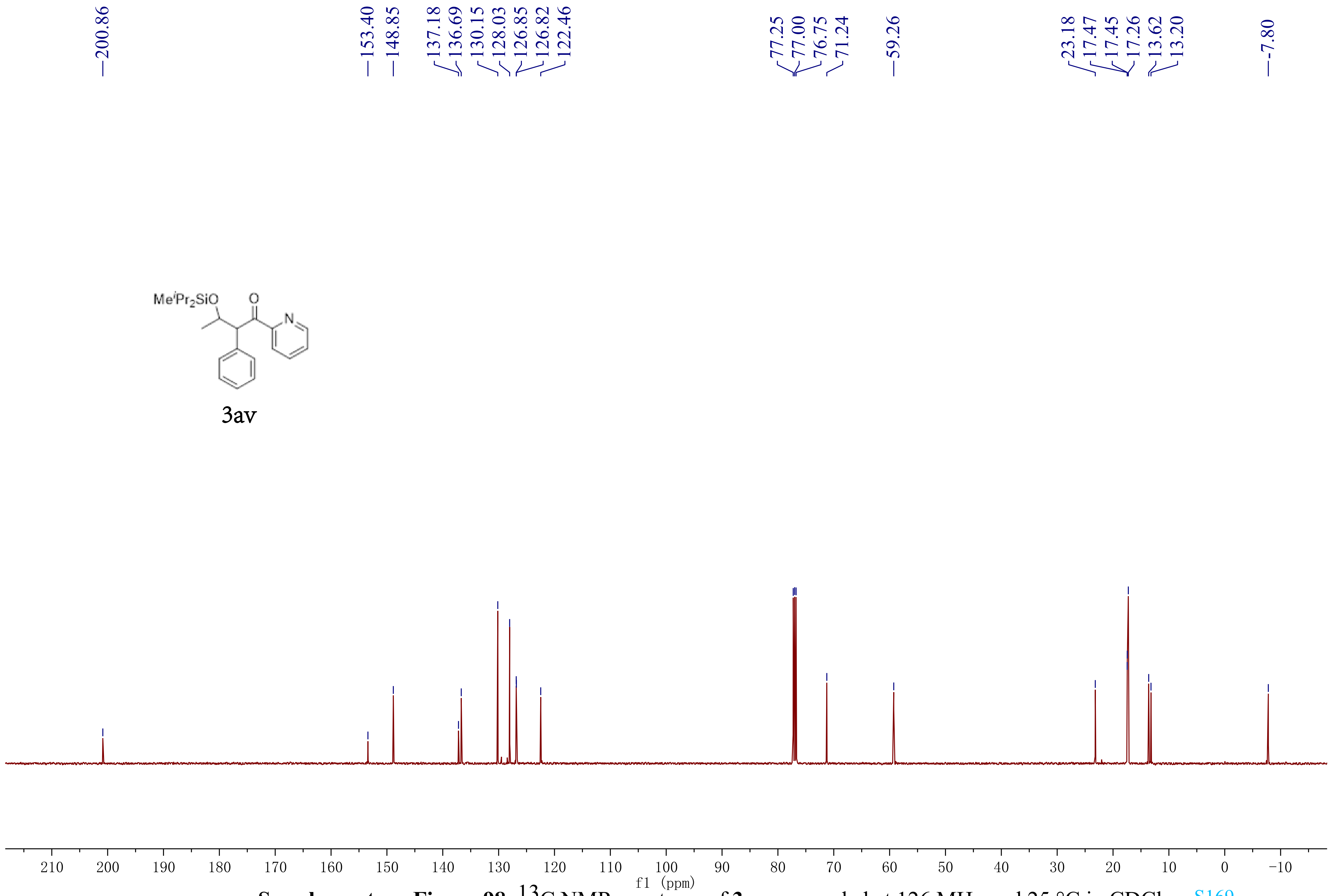

3av

Supplementary Figure 98. ^{13}C NMR spectrum of **3av**, recorded at 126 MHz and 25 °C in $CDCl_3$

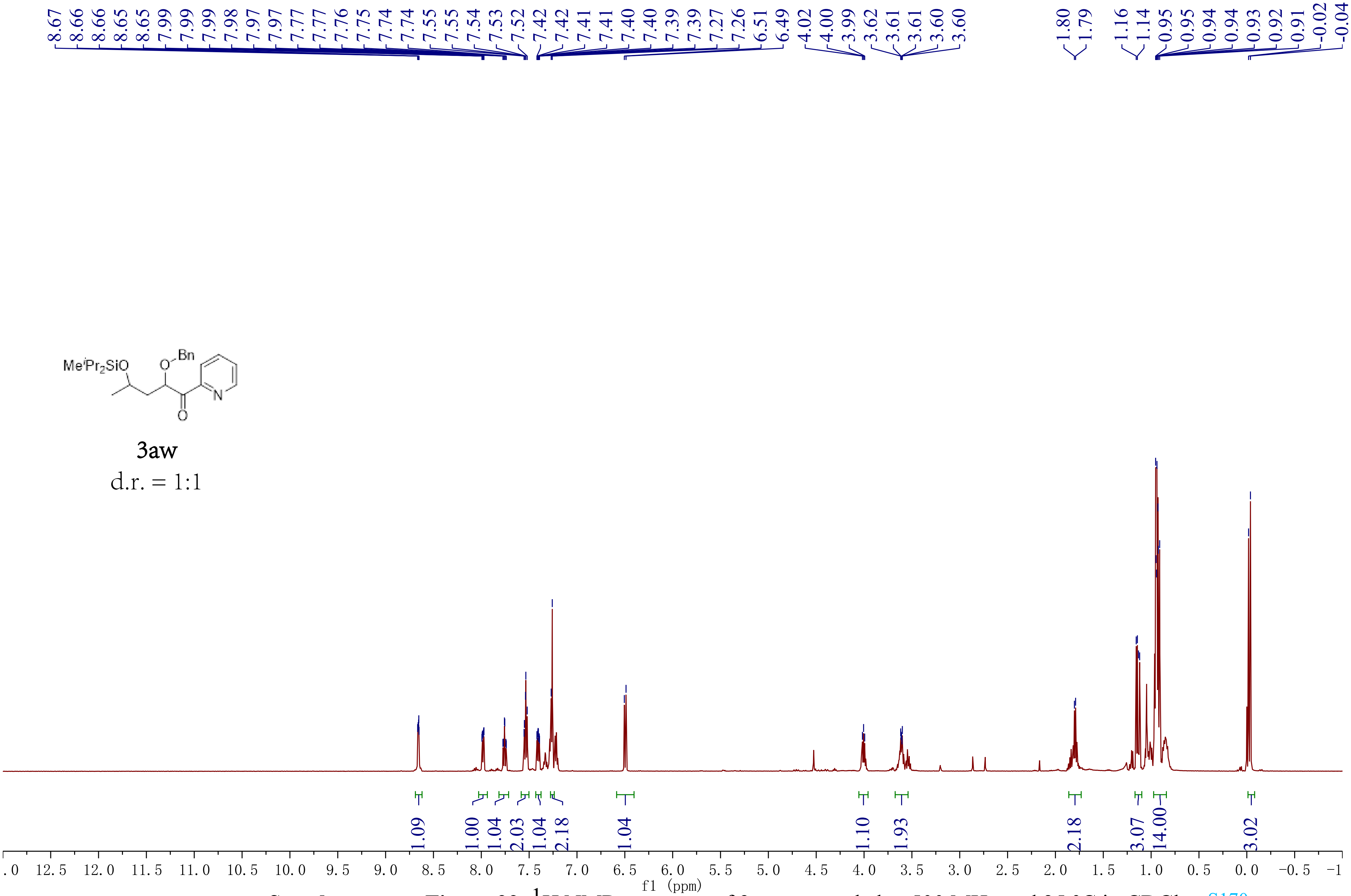

Supplementary Figure 99. ^{1}H NMR spectrum of **3aw**, recorded at 500 MHz and 25 °C in $CDCl_3$

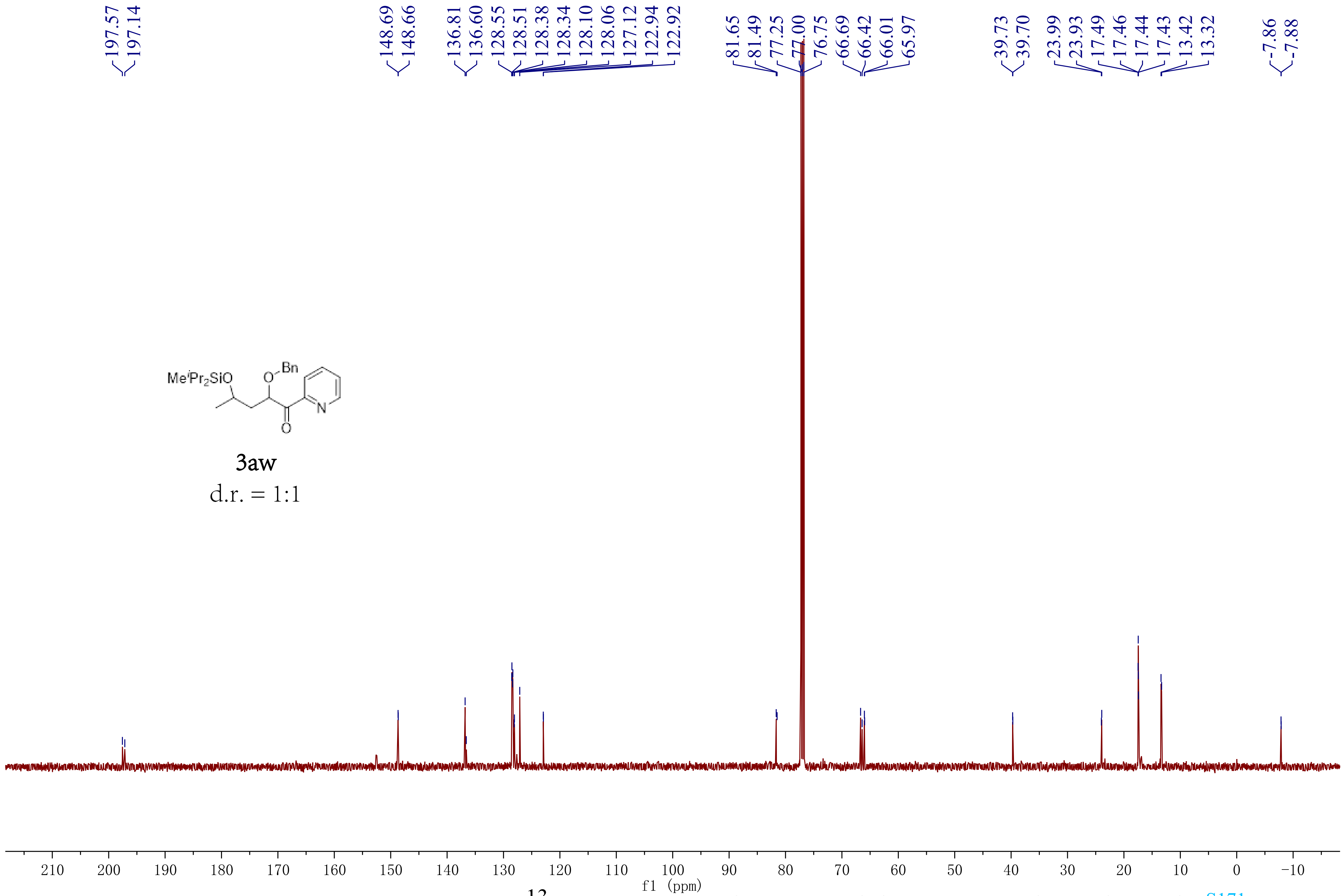

Supplementary Figure 100. ^{13}C NMR spectrum of **3aw**, recorded at 126 MHz and 25 °C in $CDCl_3$

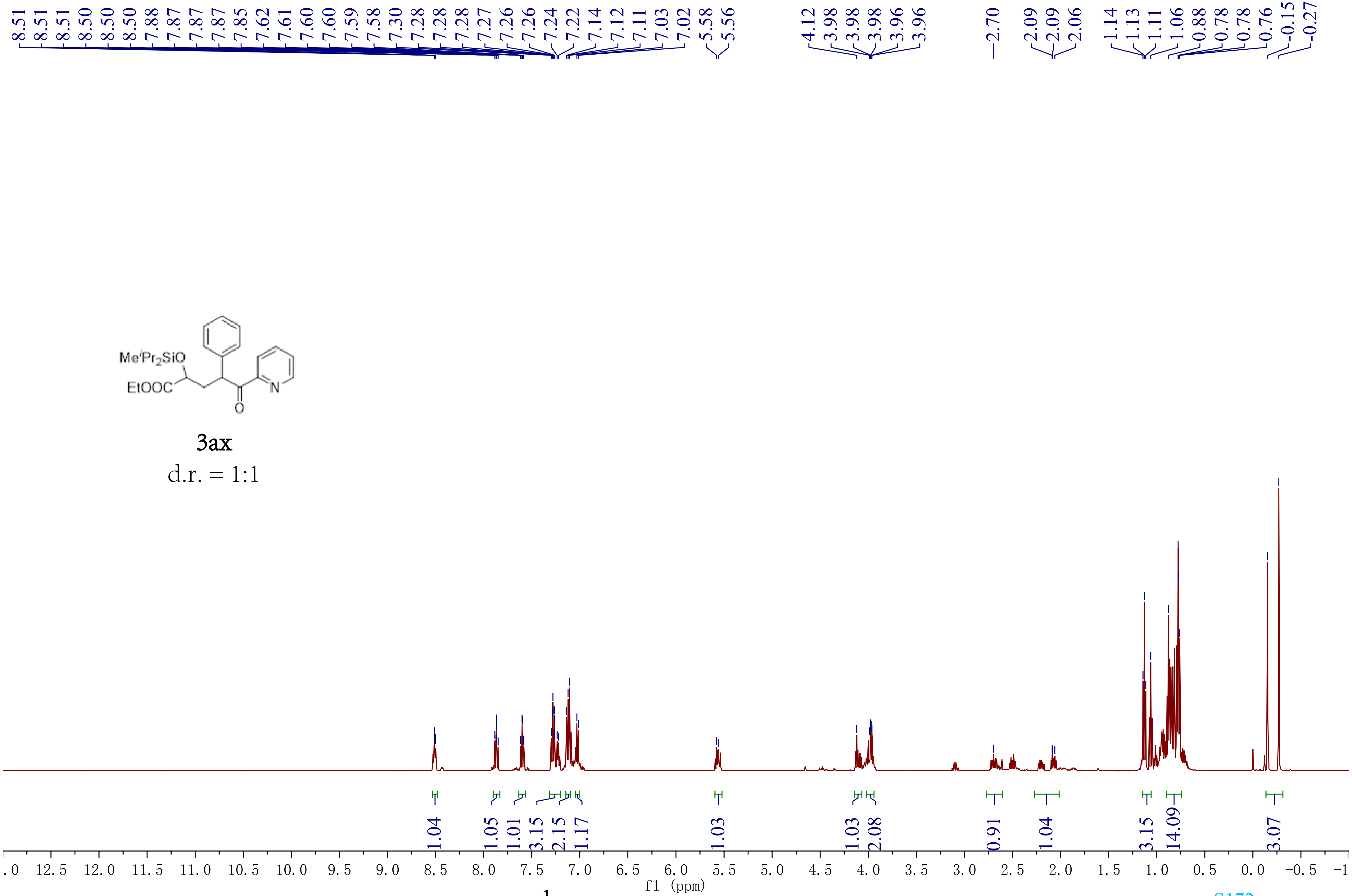

3ax

d.r. = 1:1

Supplementary Figure 101. ^{1}H NMR spectrum of **3ax**, recorded at 500 MHz and 25 °C in $CDCl_3$

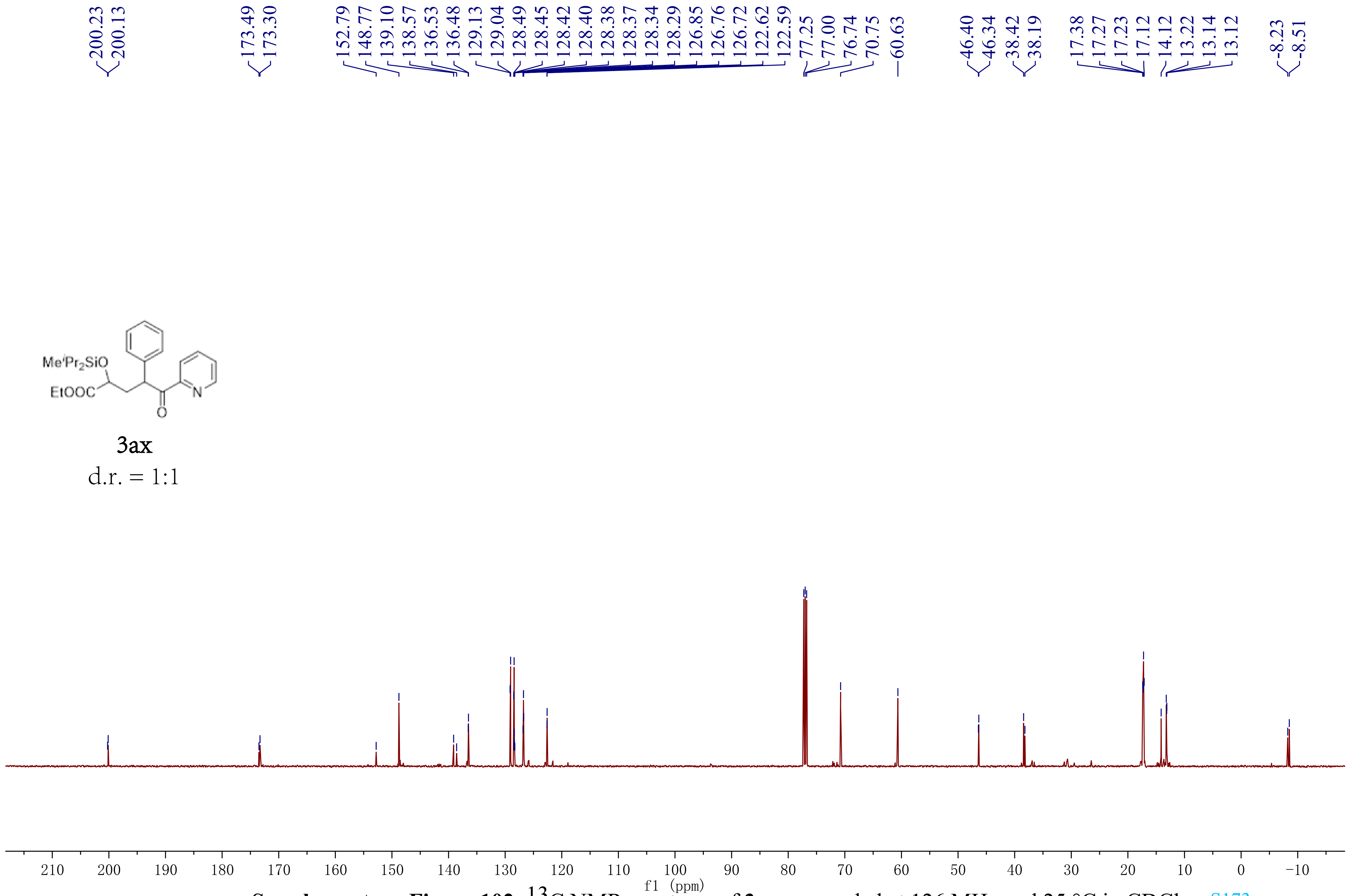

Supplementary Figure 102. ^{13}C NMR spectrum of **3ax**, recorded at 126 MHz and 25 °C in $CDCl_3$

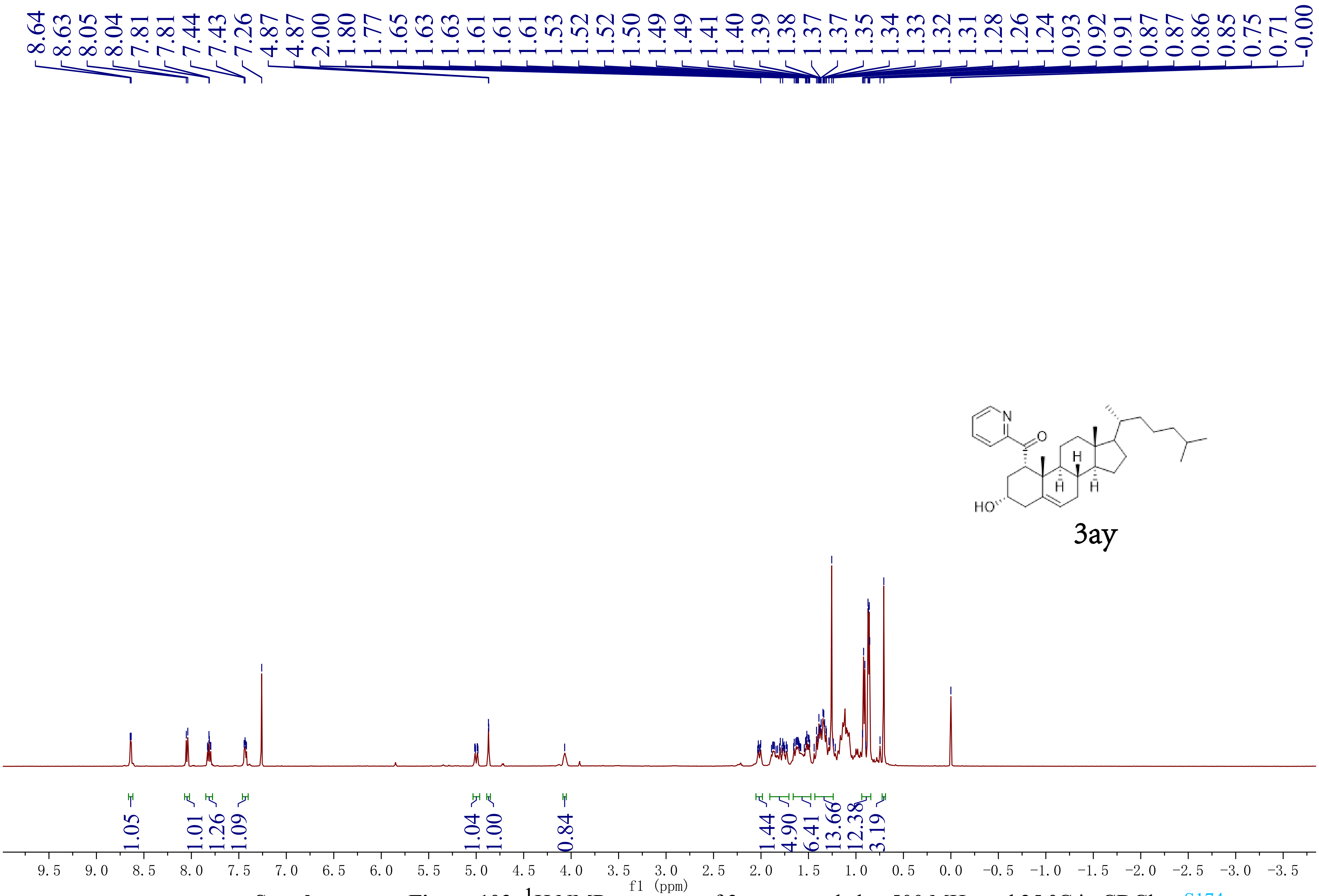

Supplementary Figure 103. ^{1}H NMR spectrum of **3ay**, recorded at 500 MHz and 25 °C in $CDCl_3$

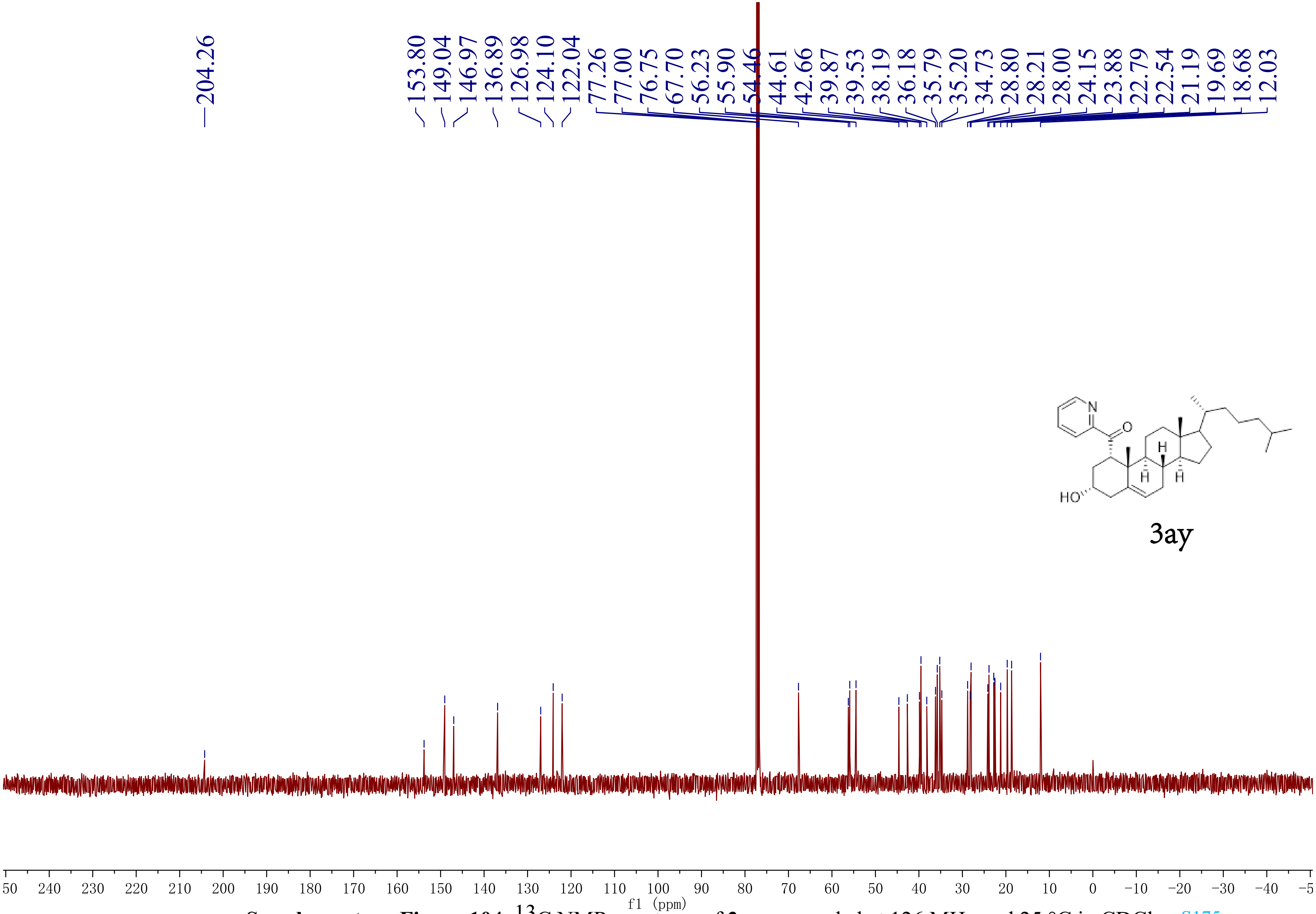

Supplementary Figure 104. ^{13}C NMR spectrum of **3ay**, recorded at 126 MHz and 25 °C in $CDCl_3$

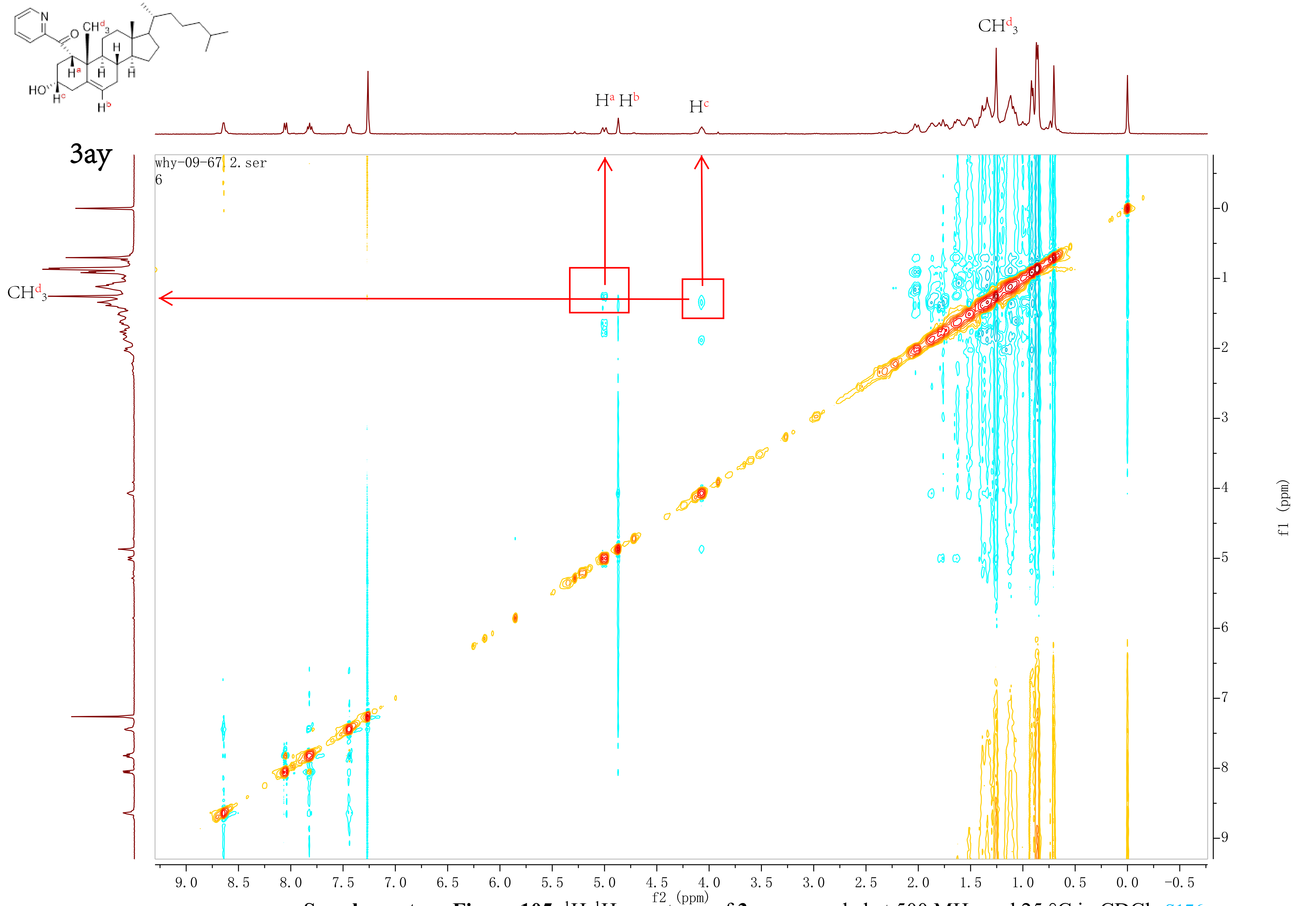

Supplementary Figure 105. ^1H-^1H spectrum of **3ay**, recorded at 500 MHz and 25 °C in $CDCl_3$

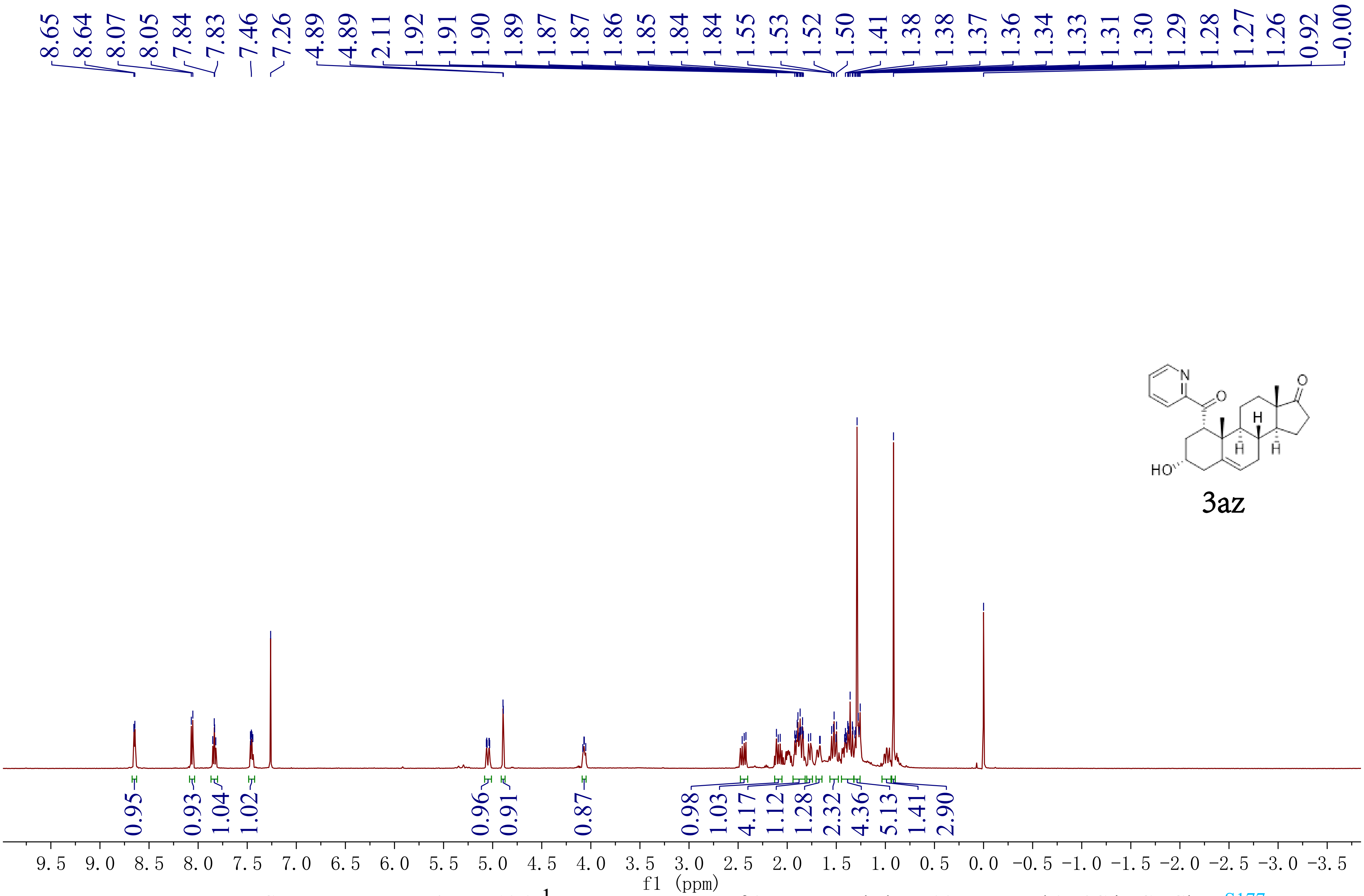

Supplementary Figure 106. ^{1}H NMR spectrum of **3az**, recorded at 500 MHz and 25 °C in $CDCl_3$

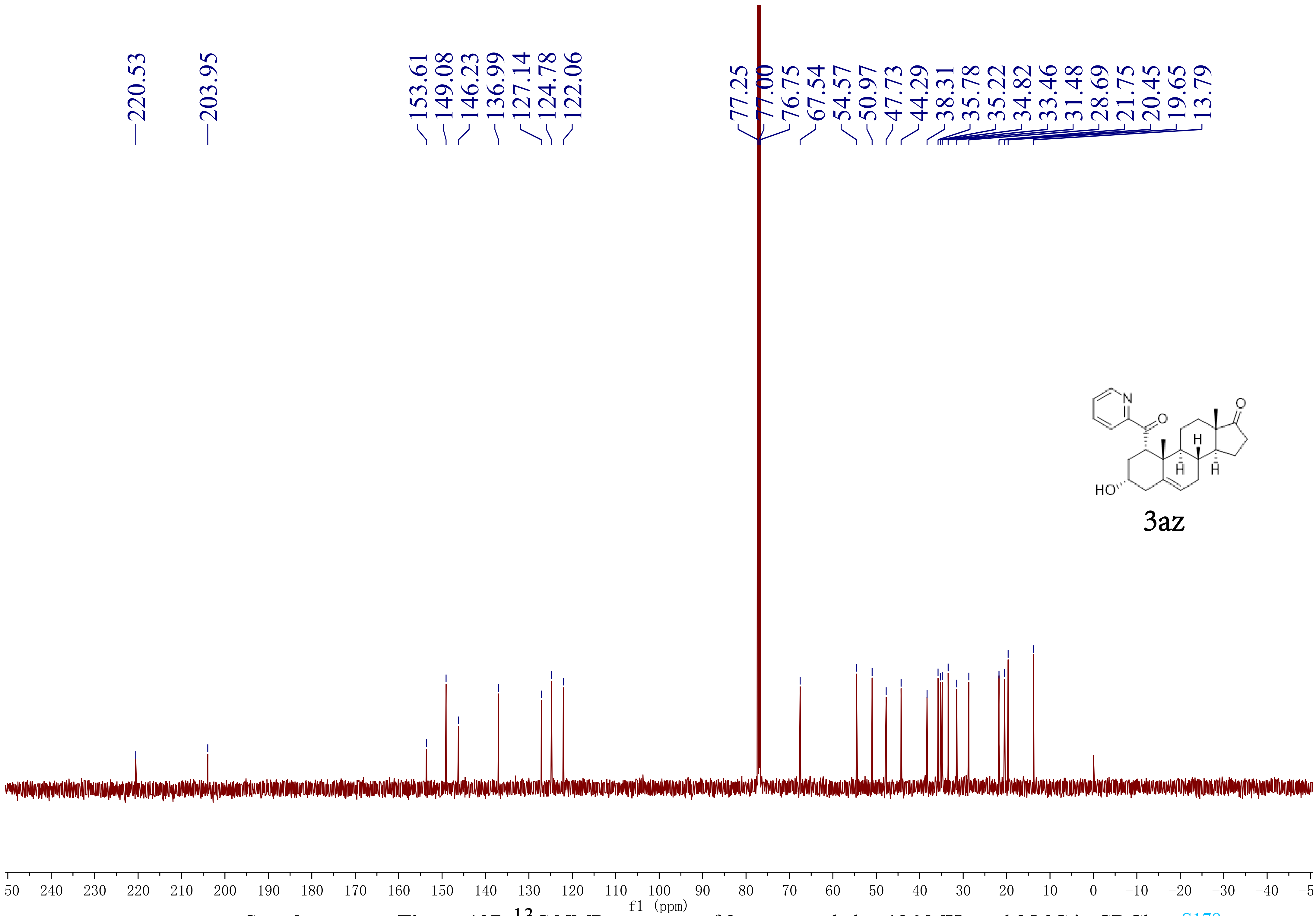

3az

Supplementary Figure 107. ^{13}C NMR spectrum of **3az**, recorded at 126 MHz and 25 °C in $CDCl_3$

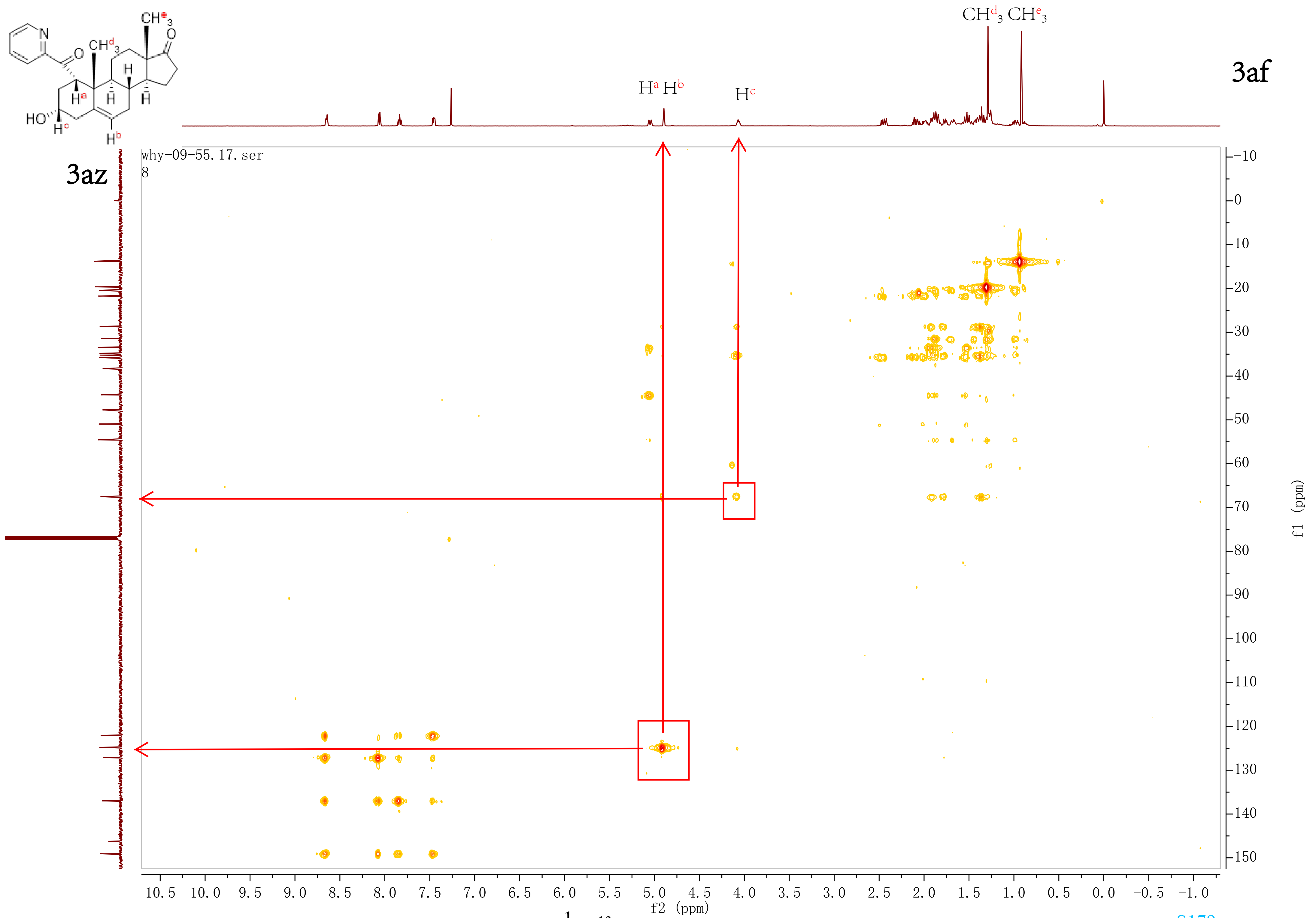

Supplementary Figure 108. ^{1}H-^{13}C spectrum of **3az**, recorded at 500 MHz and 25 °C in $CDCl_3$

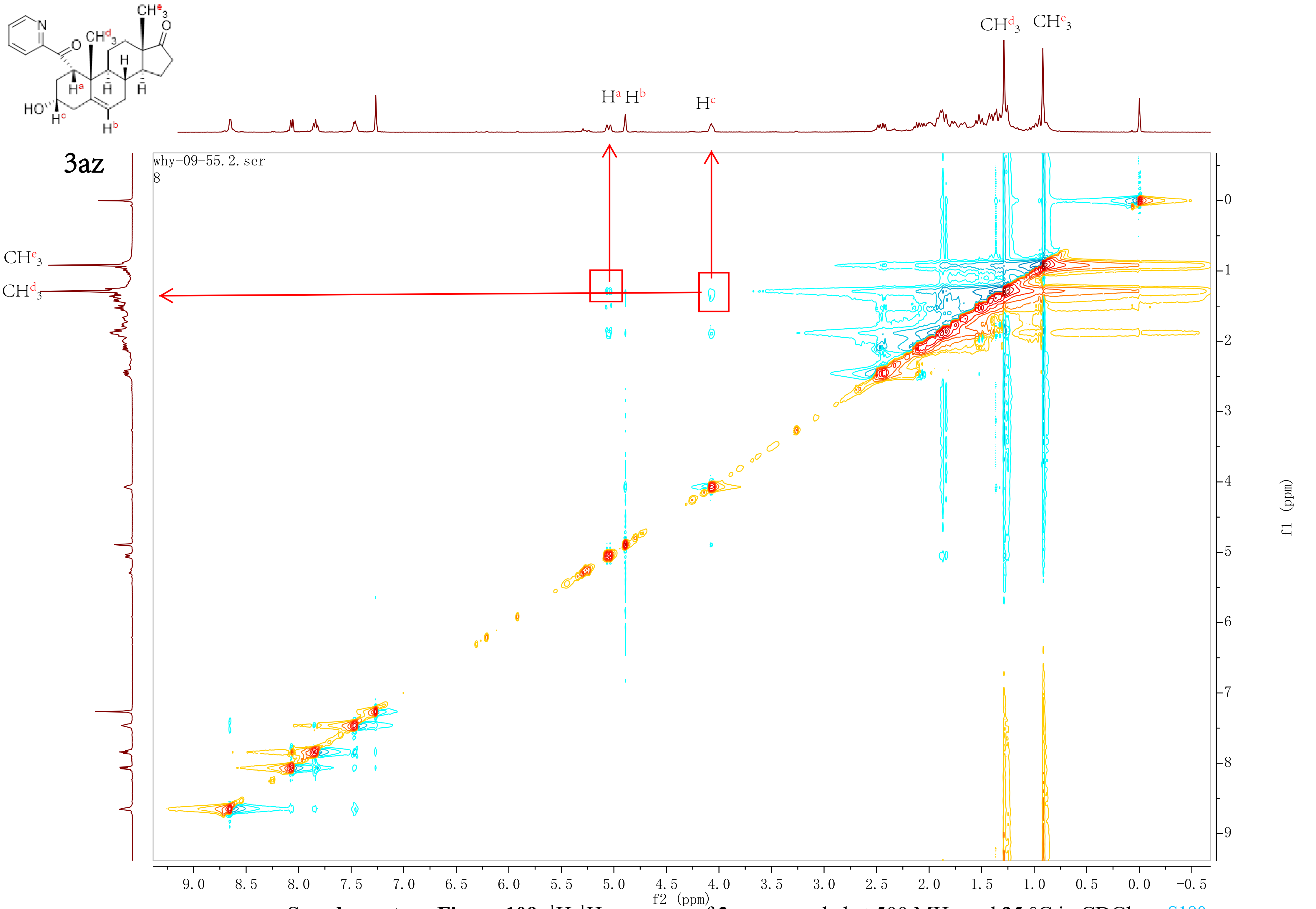

Supplementary Figure 109. ^{1}H-^{1}H spectrum of **3az**, recorded at 500 MHz and 25 °C in $CDCl_3$

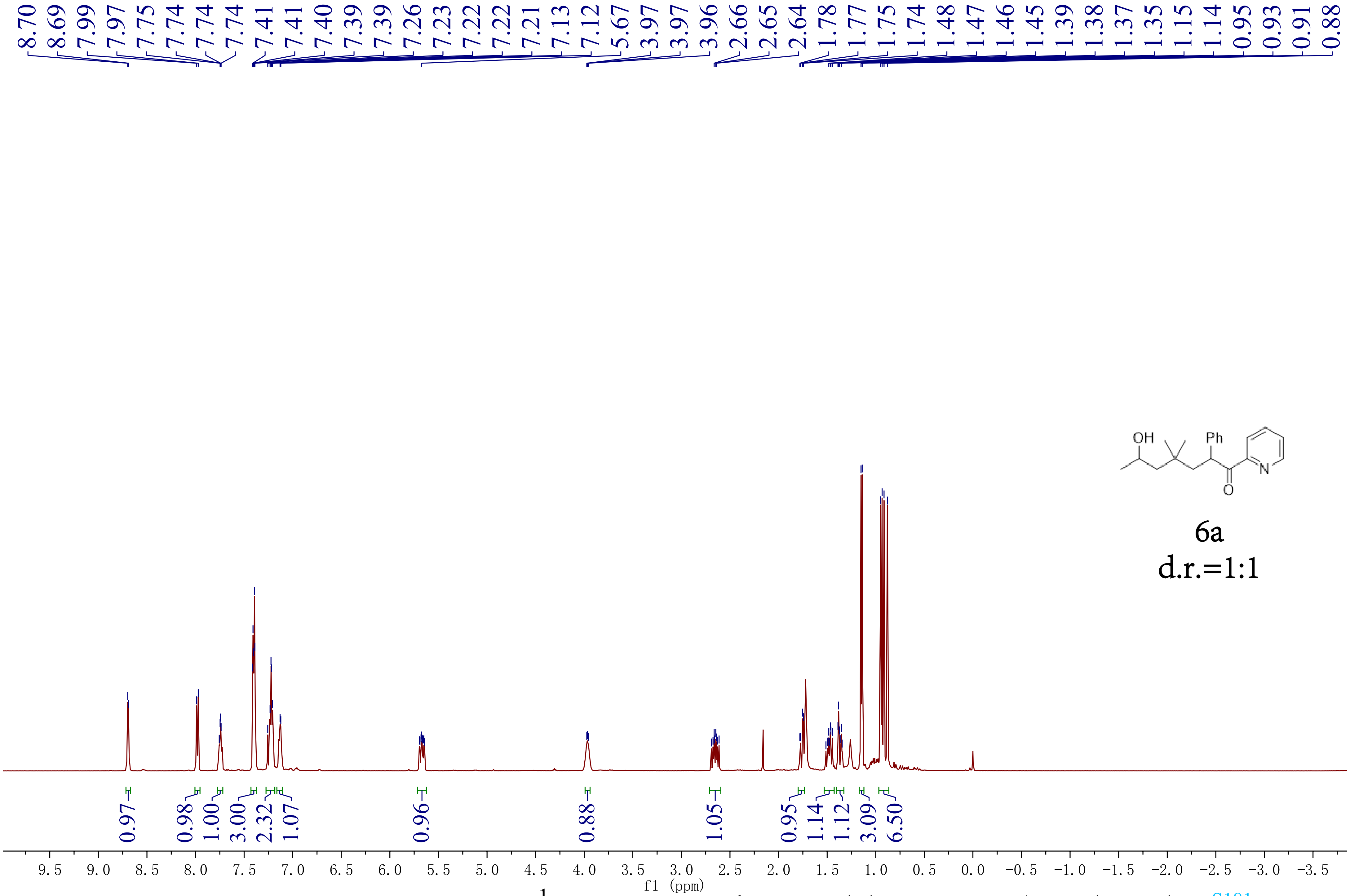

Supplementary Figure 110. ^{1}H NMR spectrum of **6a**, recorded at 500 MHz and 25 °C in $CDCl_3$

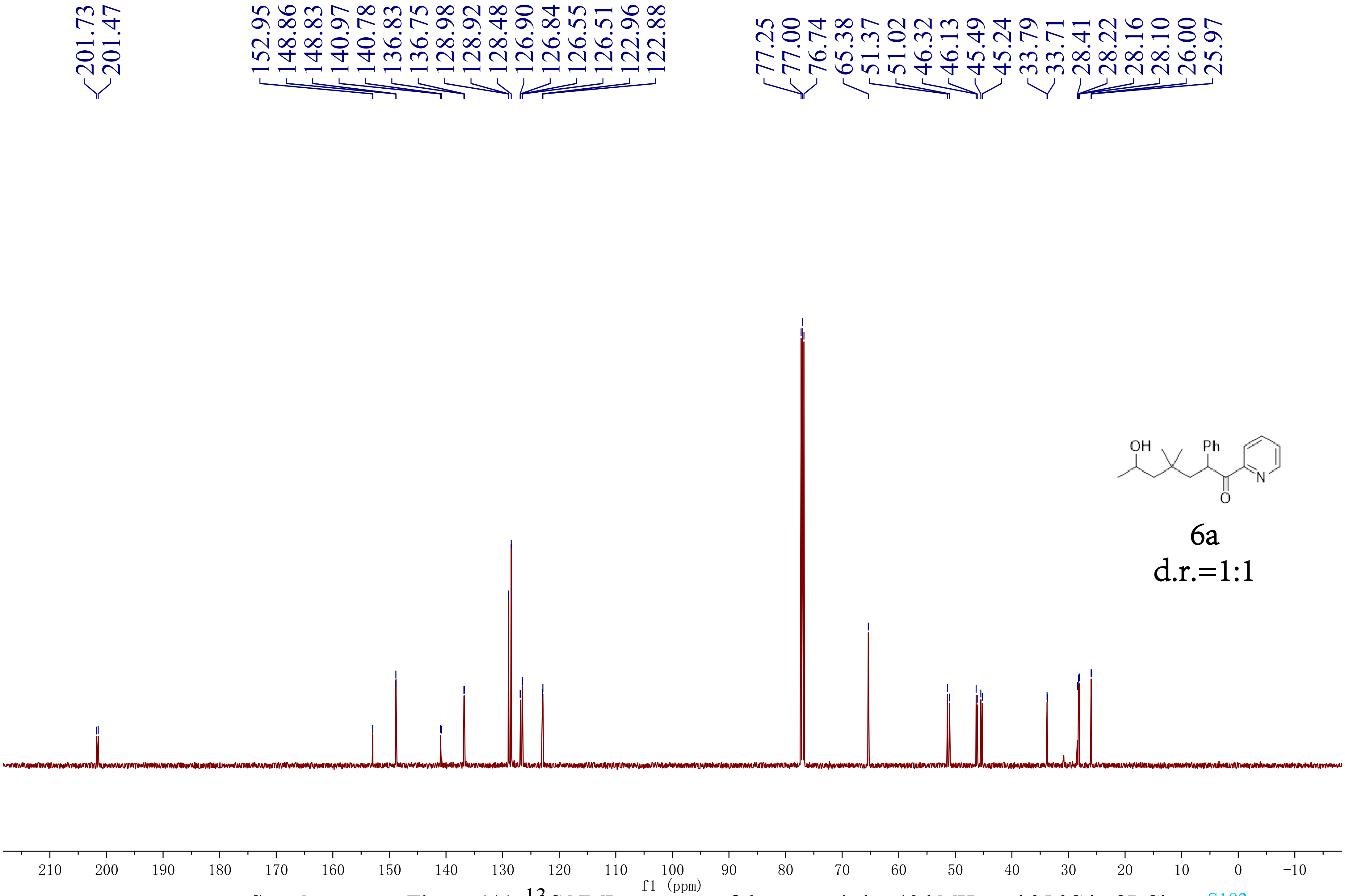

6a
d.r.=1:1

Supplementary Figure 111. ^{13}C NMR spectrum of **6a**, recorded at 126 MHz and 25 °C in $CDCl_3$

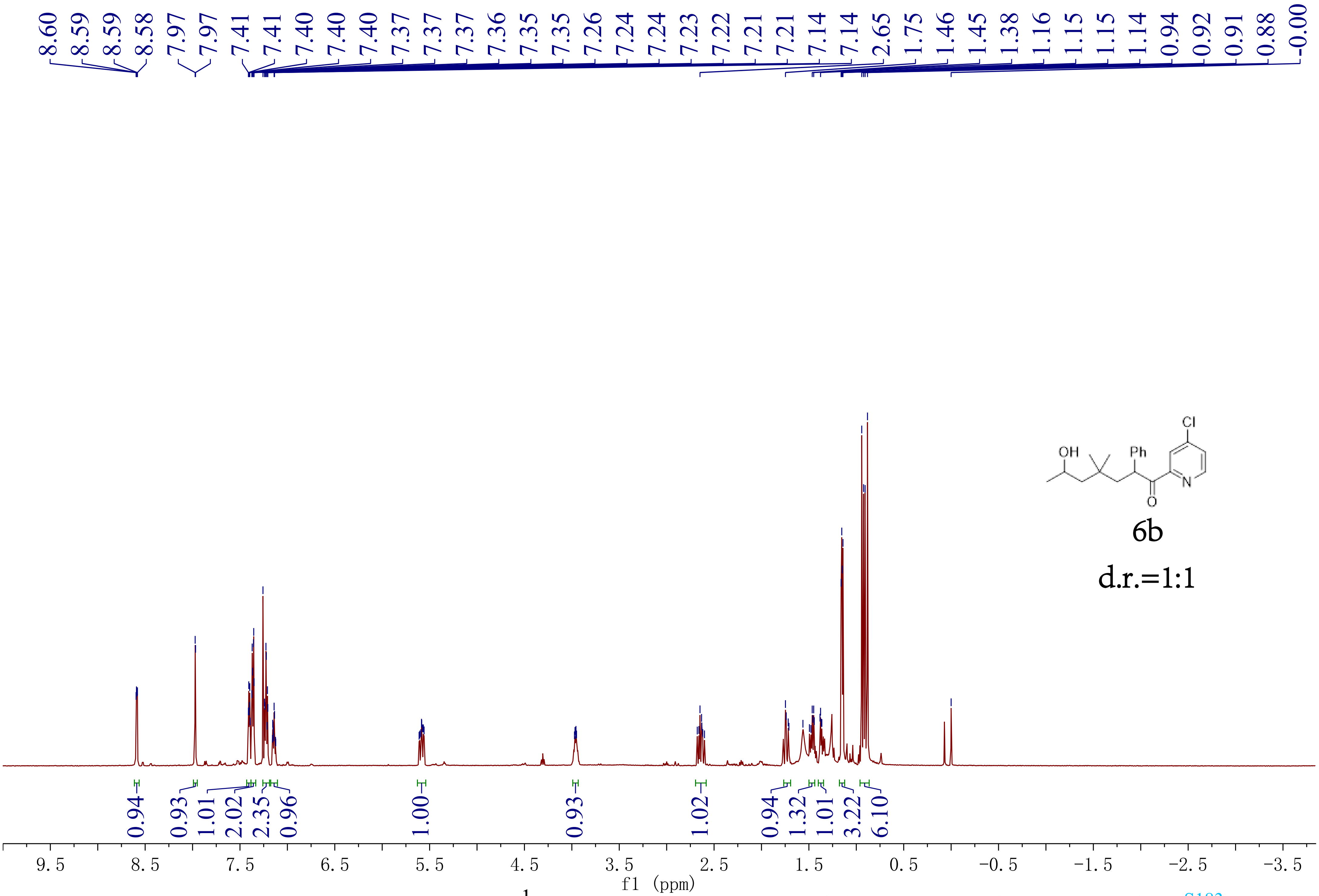

Supplementary Figure 112. ^{1}H NMR spectrum of **6b**, recorded at 500 MHz and 25 °C in $CDCl_3$

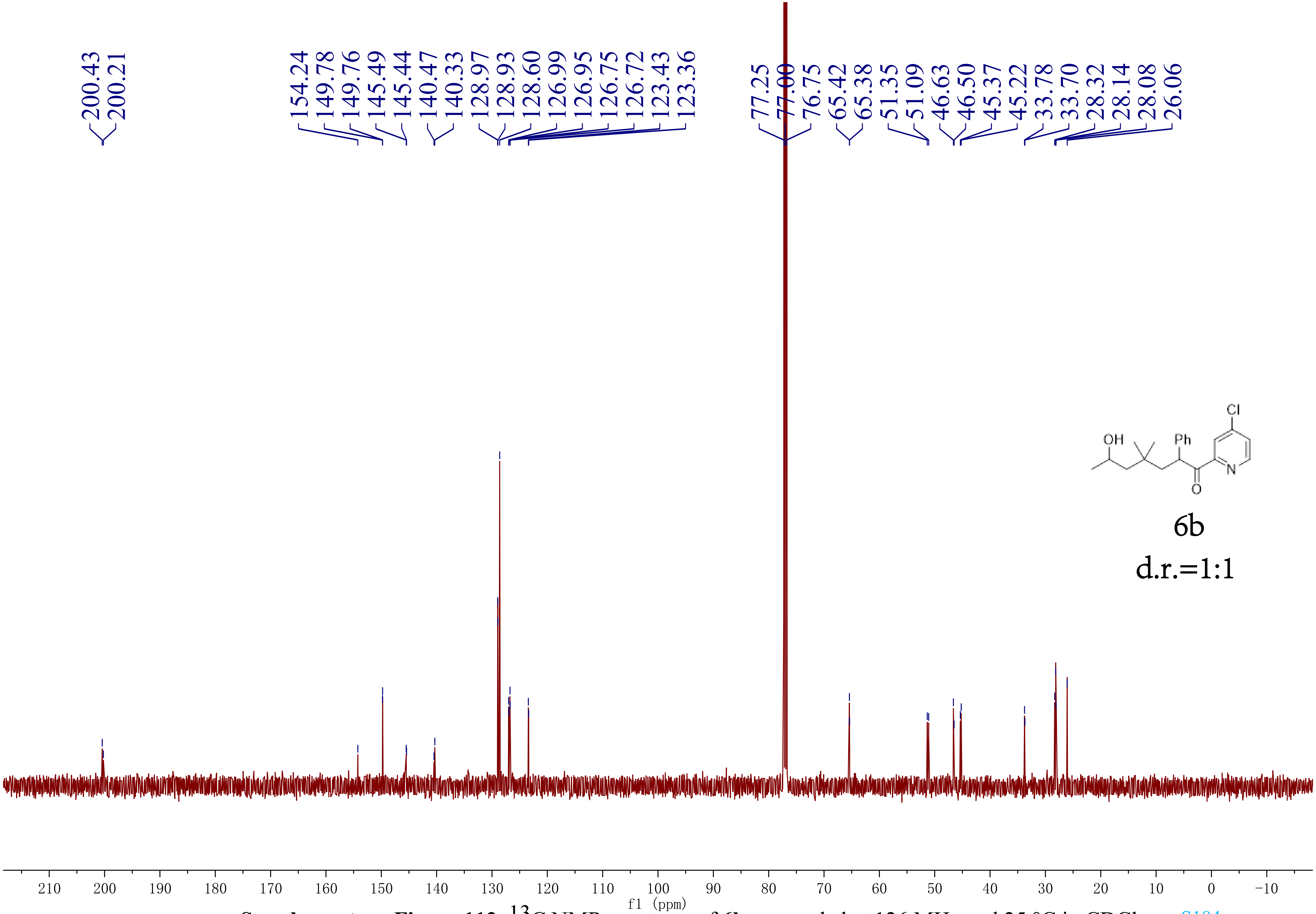

Supplementary Figure 113. ^{13}C NMR spectrum of **6b**, recorded at 126 MHz and 25 °C in $CDCl_3$

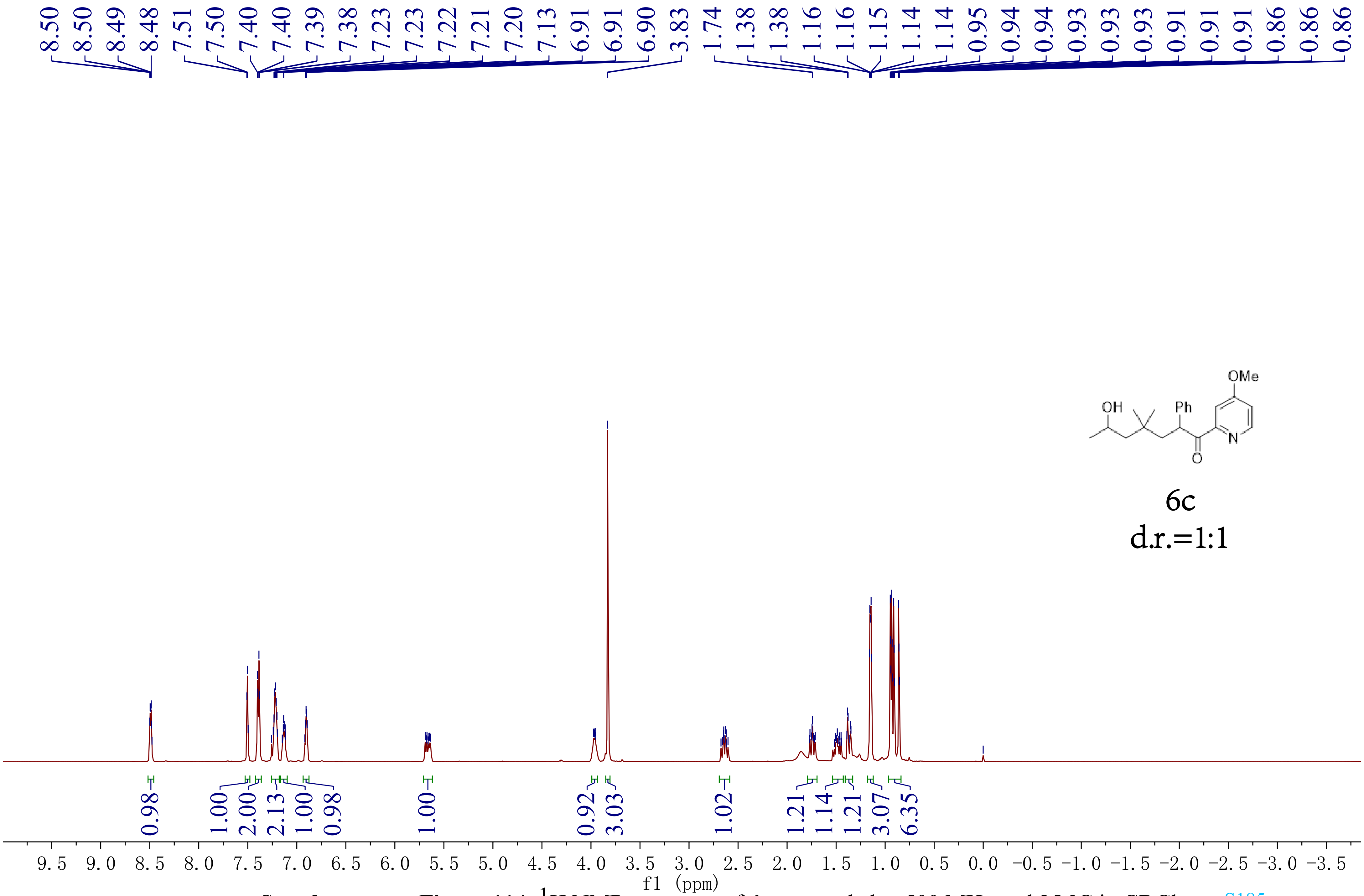

Supplementary Figure 114. ^{1}H NMR spectrum of **6c**, recorded at 500 MHz and 25 °C in $CDCl_3$

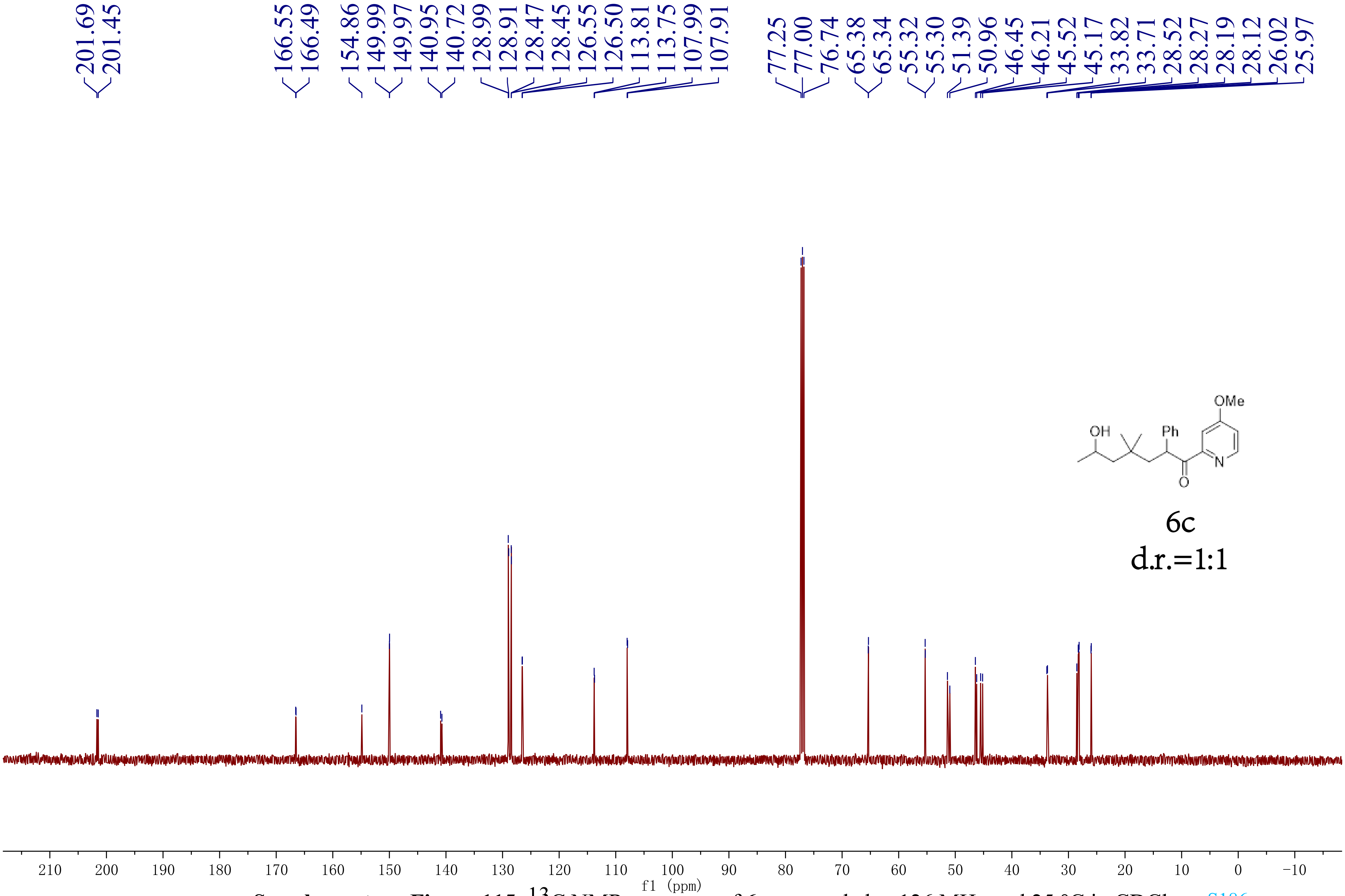

6c
d.r.=1:1

Supplementary Figure 115. ^{13}C NMR spectrum of **6c**, recorded at 126 MHz and 25 °C in $CDCl_3$

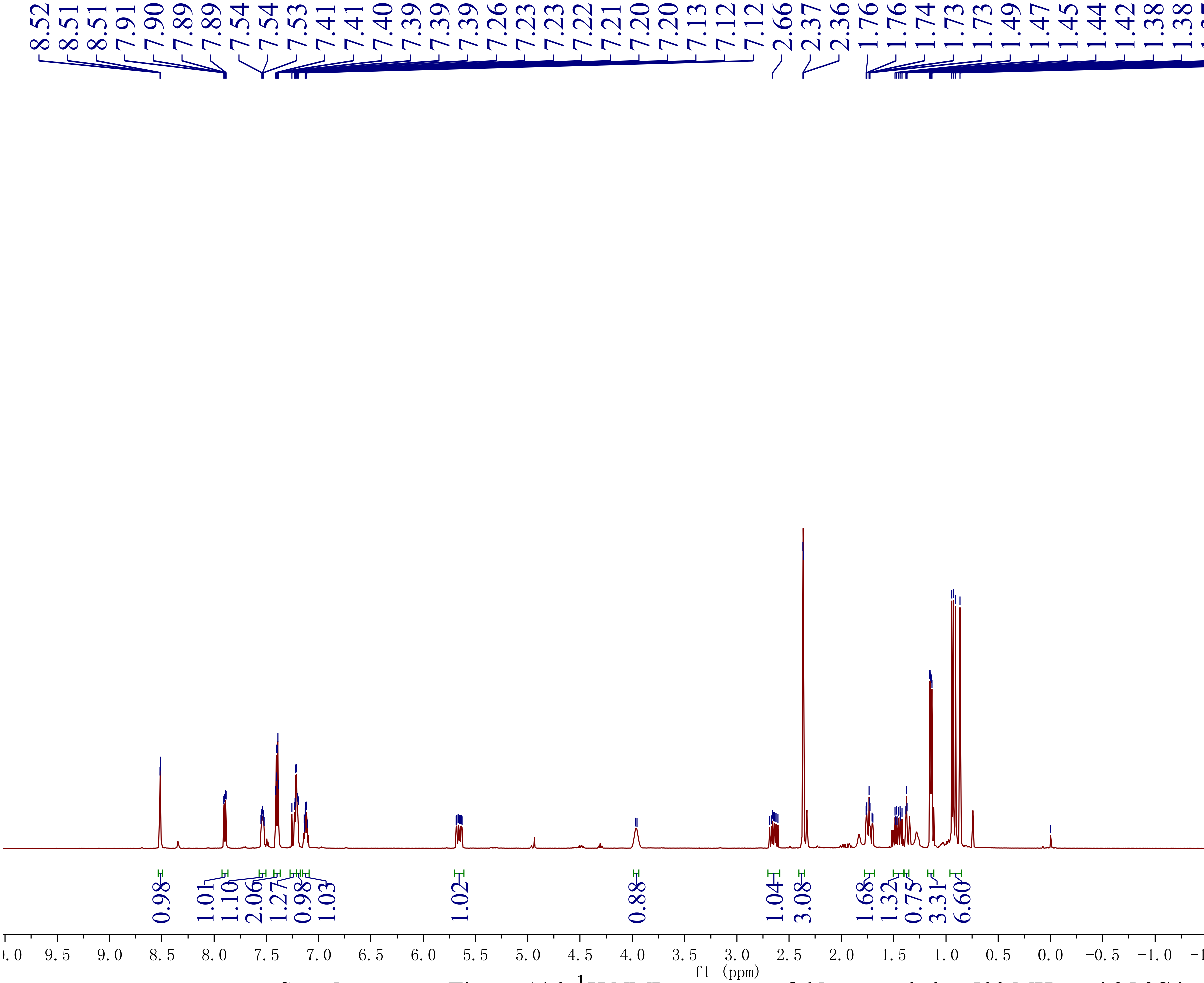

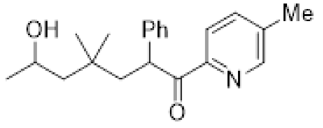

6d
d.r.=1:1

Supplementary Figure 116. ^{1}H NMR spectrum of **6d**, recorded at 500 MHz and 25 °C in $CDCl_3$

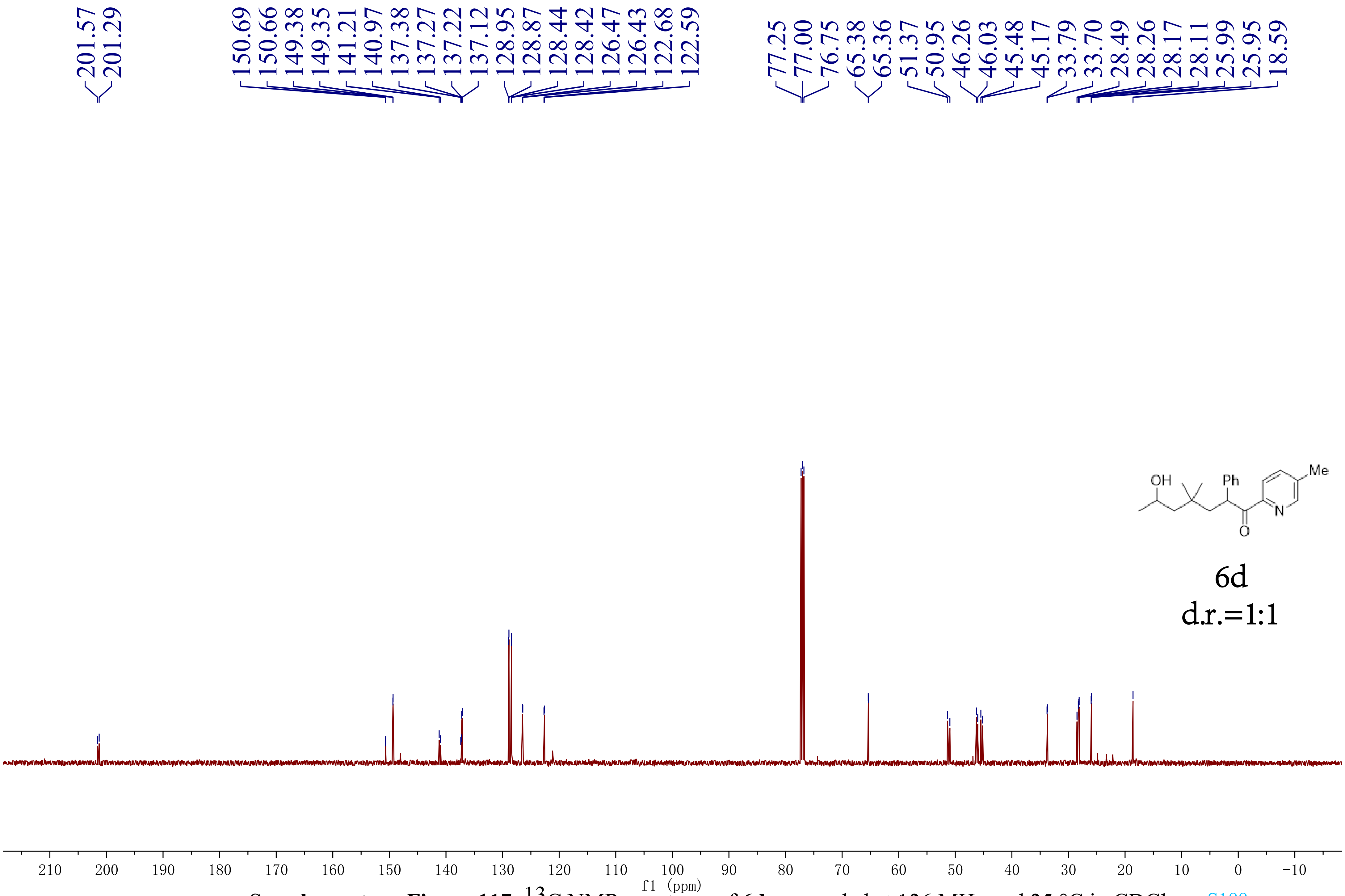

Supplementary Figure 117. ^{13}C NMR spectrum of **6d**, recorded at 126 MHz and 25 °C in $CDCl_3$

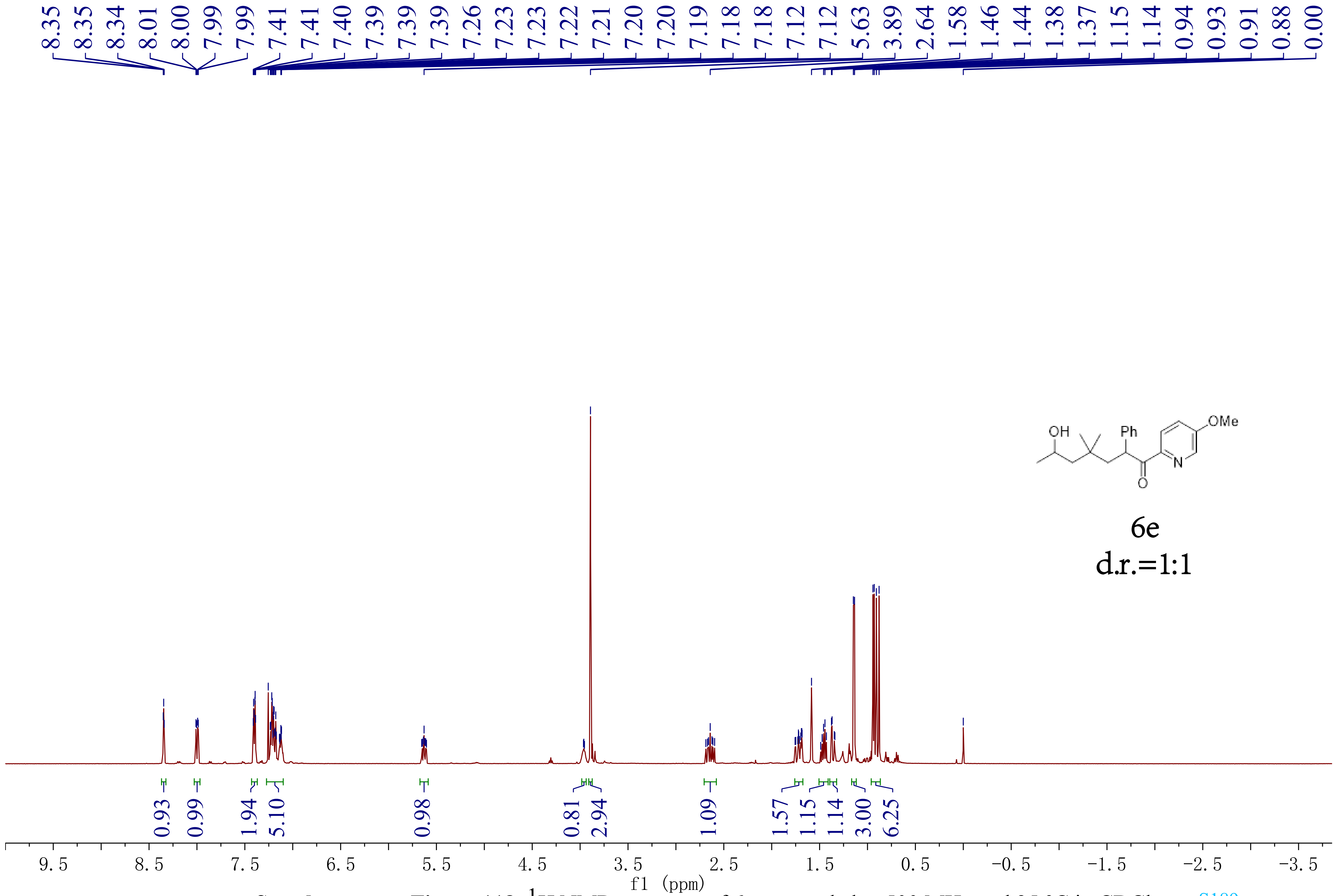

Supplementary Figure 118. ^{1}H NMR spectrum of **6e**, recorded at 500 MHz and 25 °C in $CDCl_3$

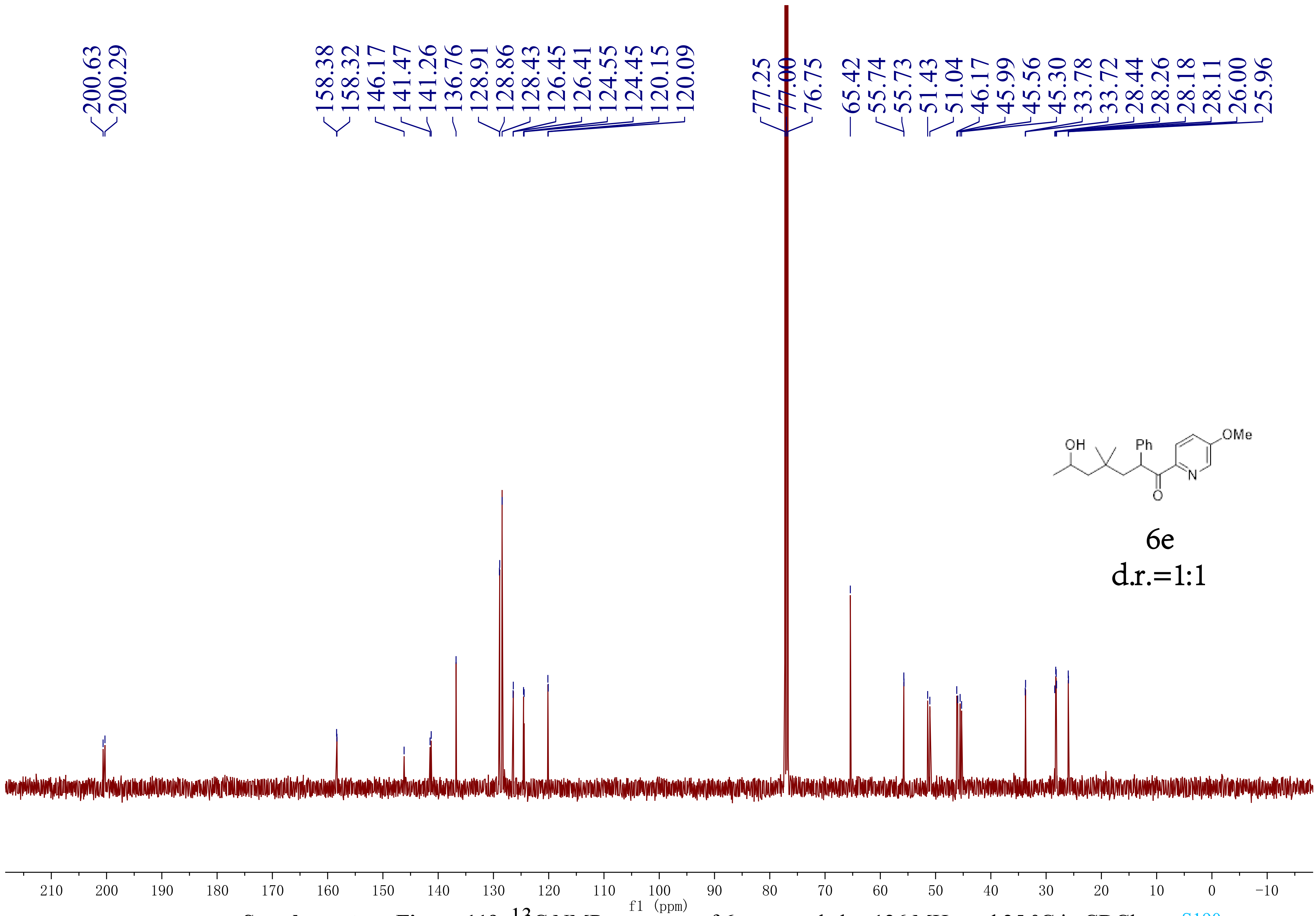

Supplementary Figure 119. ^{13}C NMR spectrum of **6e**, recorded at 126 MHz and 25 °C in $CDCl_3$

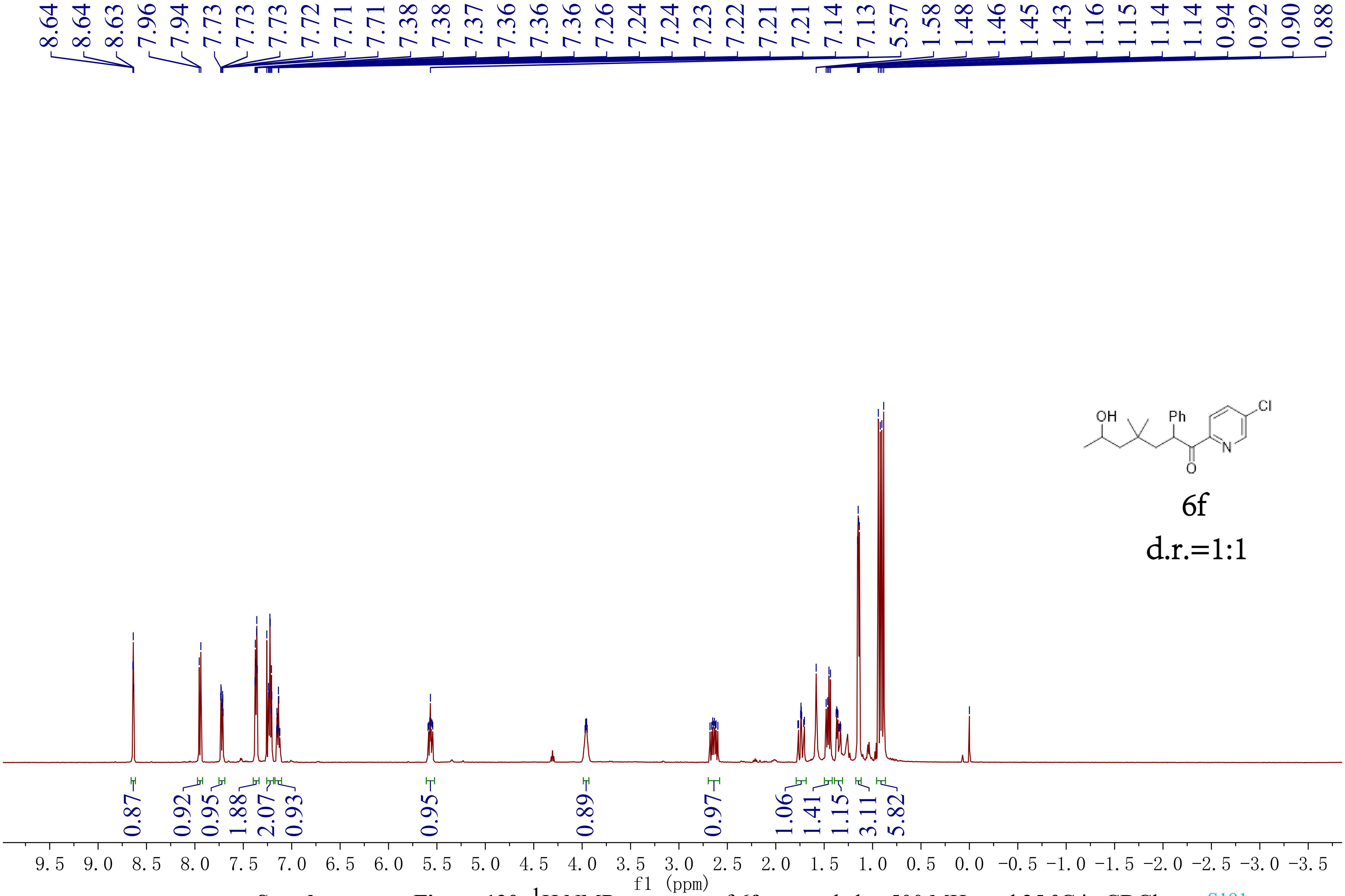

Supplementary Figure 120. ^{1}H NMR spectrum of **6f**, recorded at 500 MHz and 25 °C in $CDCl_3$

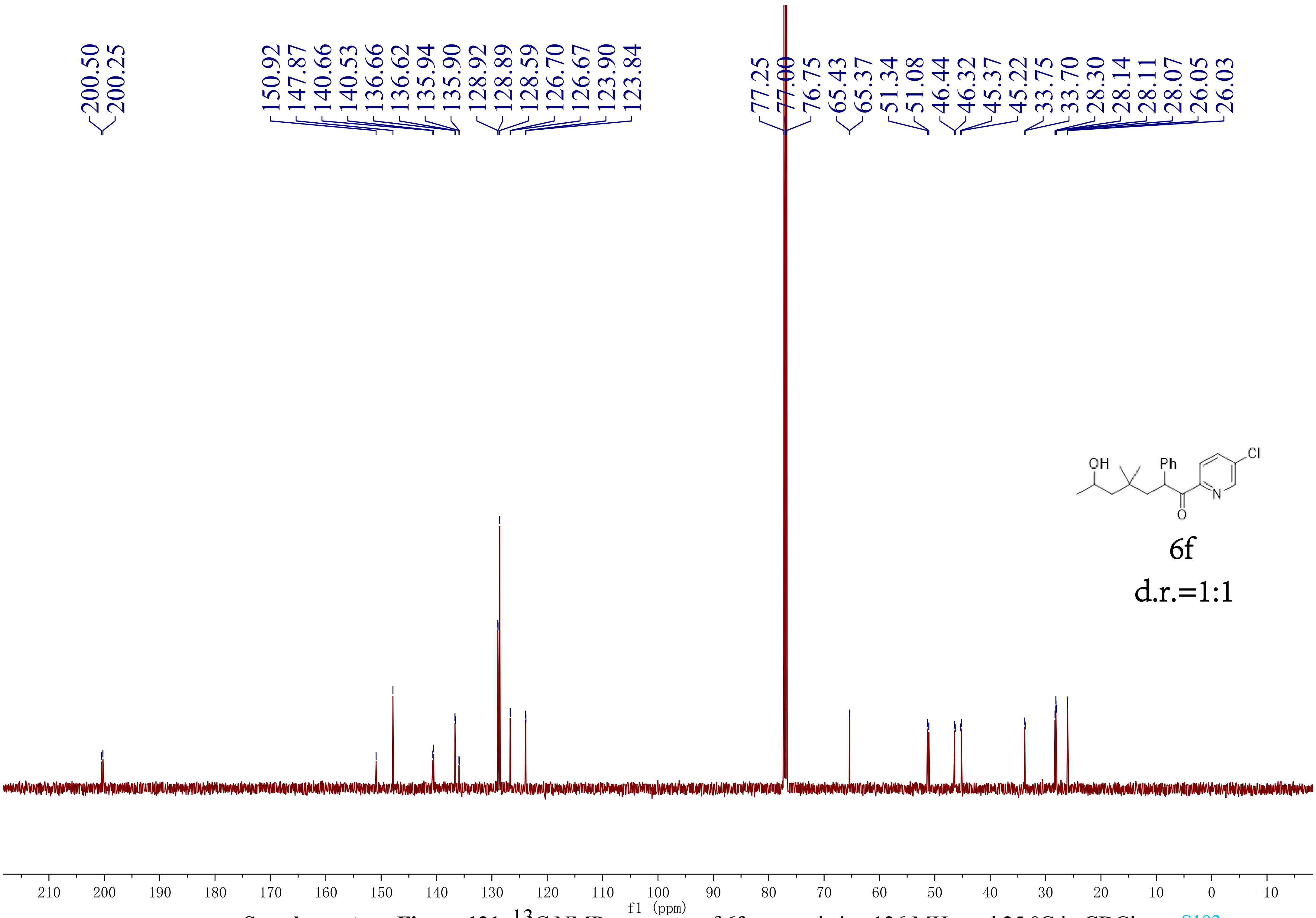

Supplementary Figure 121. ^{13}C NMR spectrum of **6f**, recorded at 126 MHz and 25 °C in $CDCl_3$

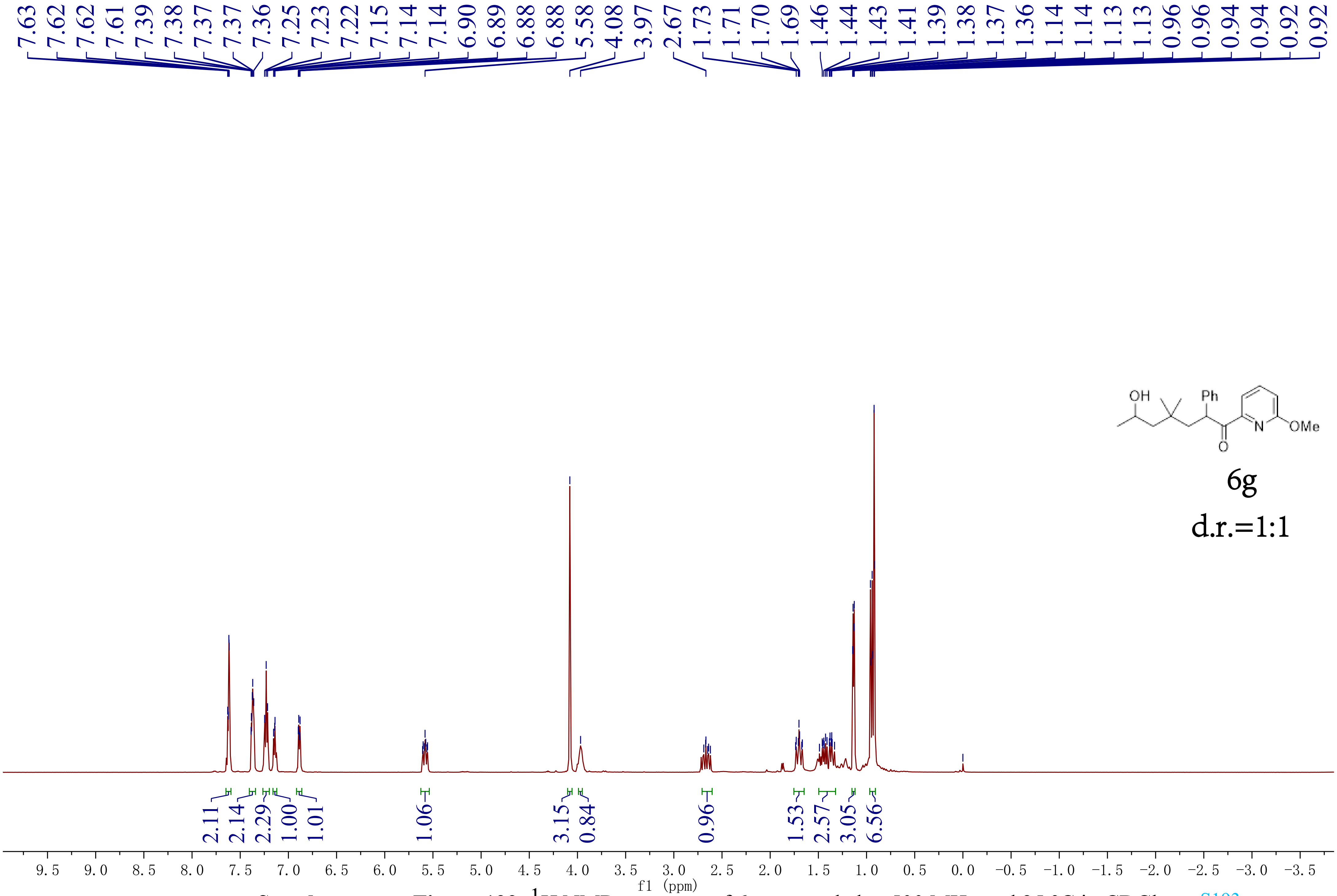

Supplementary Figure 122. ^{1}H NMR spectrum of **6g**, recorded at 500 MHz and 25 °C in $CDCl_3$

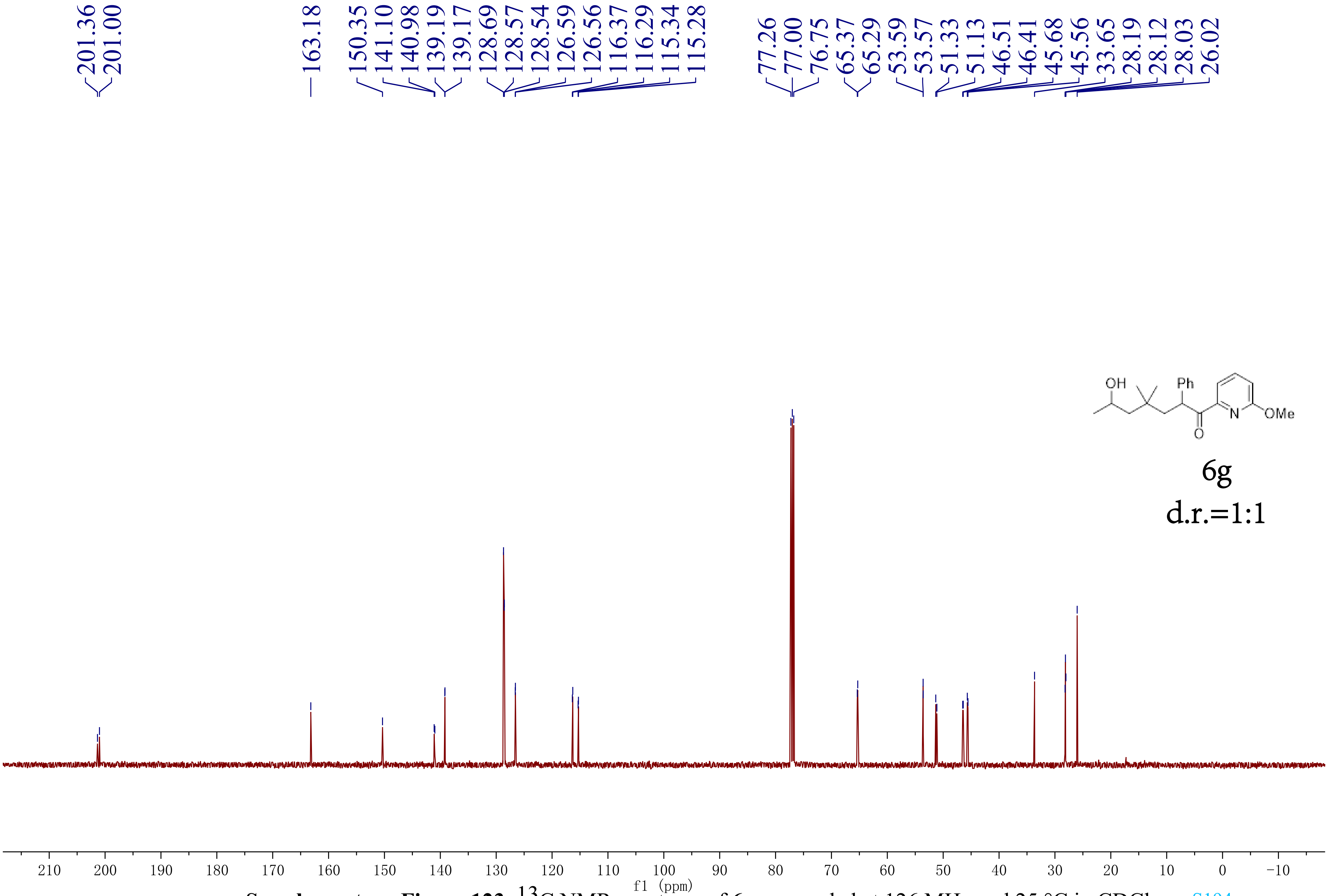

Supplementary Figure 123. ^{13}C NMR spectrum of **6g**, recorded at 126 MHz and 25 °C in $CDCl_3$

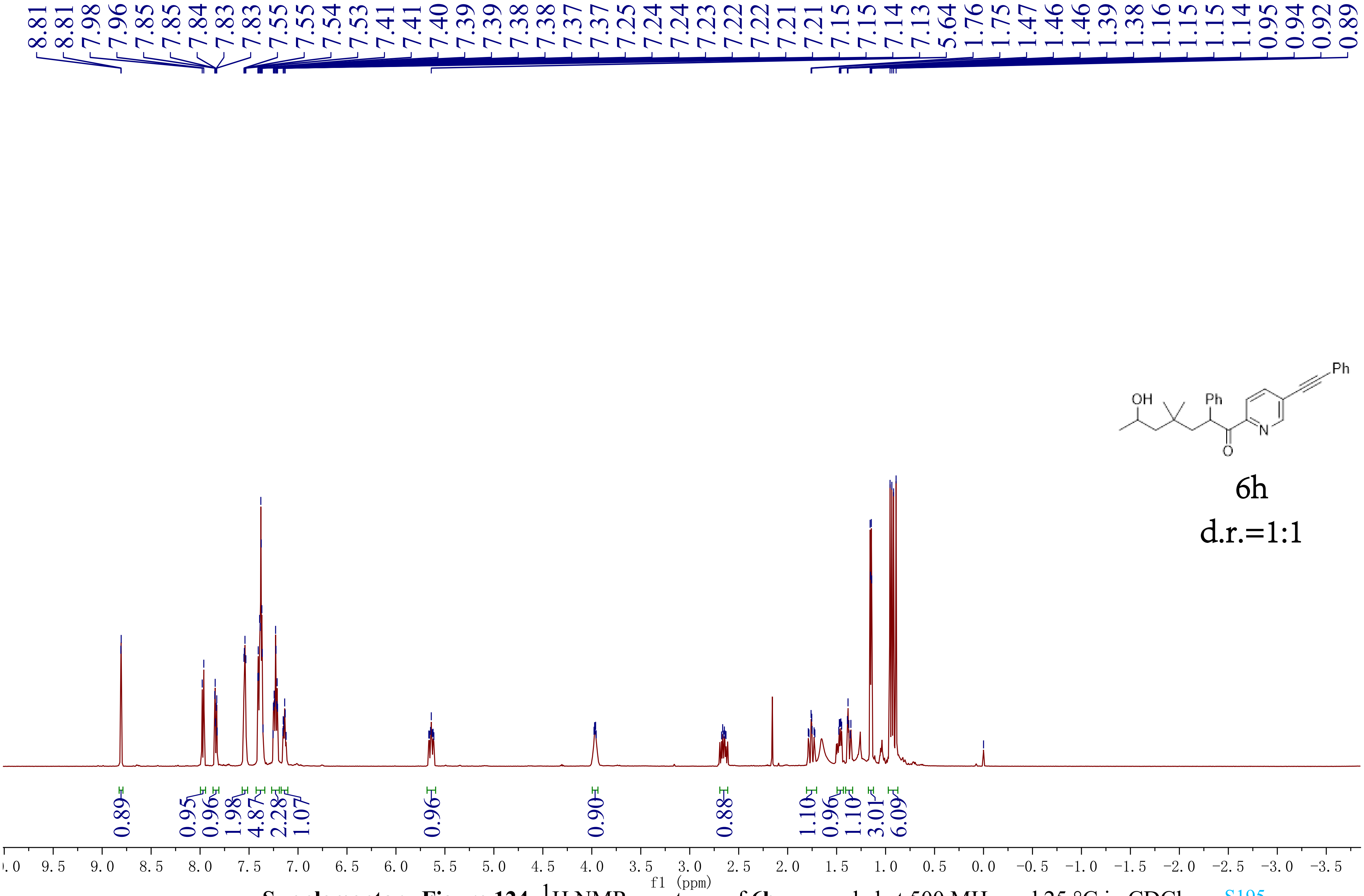

Supplementary Figure 124. ^{1}H NMR spectrum of **6h**, recorded at 500 MHz and 25 °C in $CDCl_3$

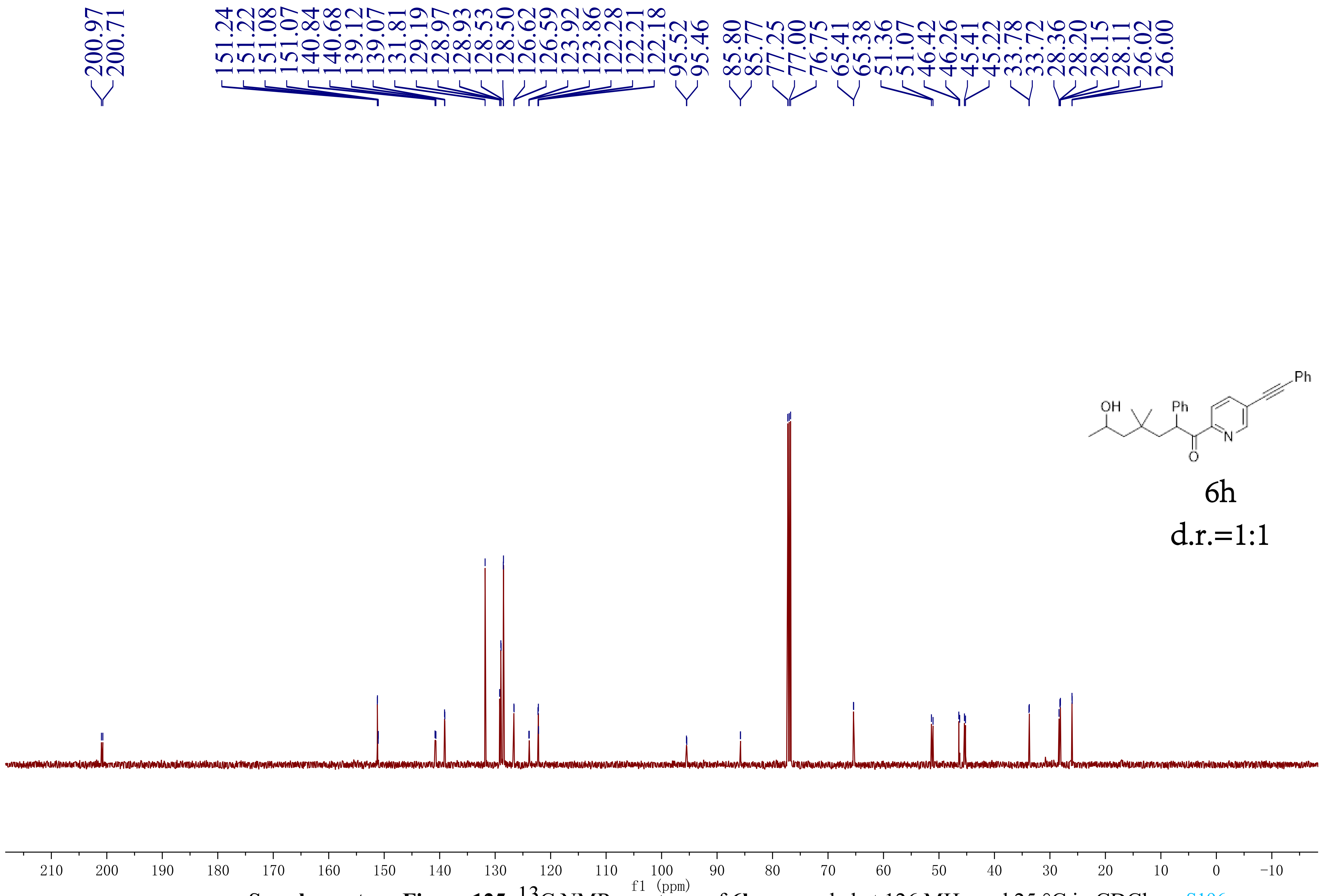

Supplementary Figure 125. ^{13}C NMR spectrum of **6h**， recorded at 126 MHz and 25 °C in $CDCl_3$

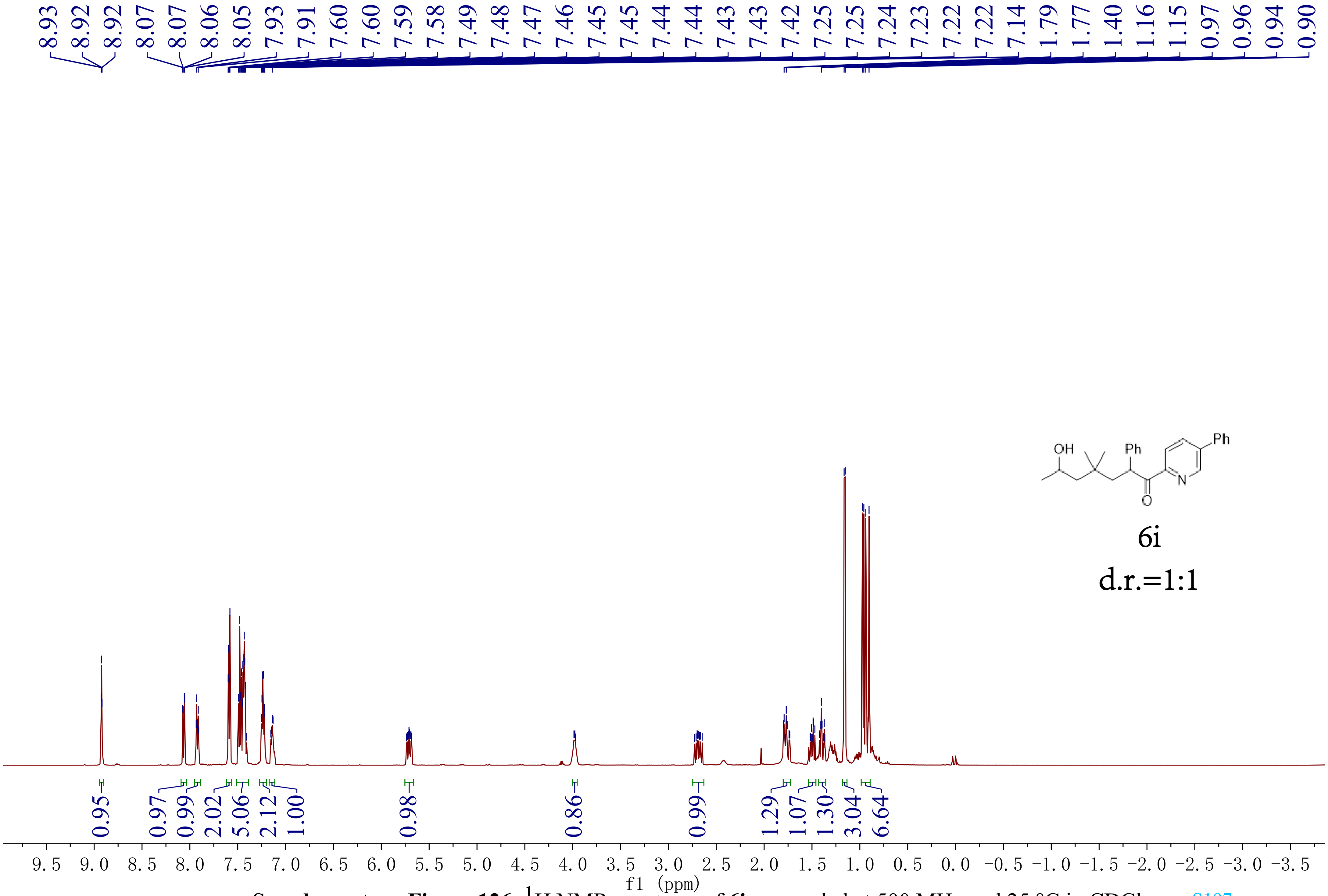

Supplementary Figure 126. ^{1}H NMR spectrum of **6i**, recorded at 500 MHz and 25 °C in $CDCl_3$

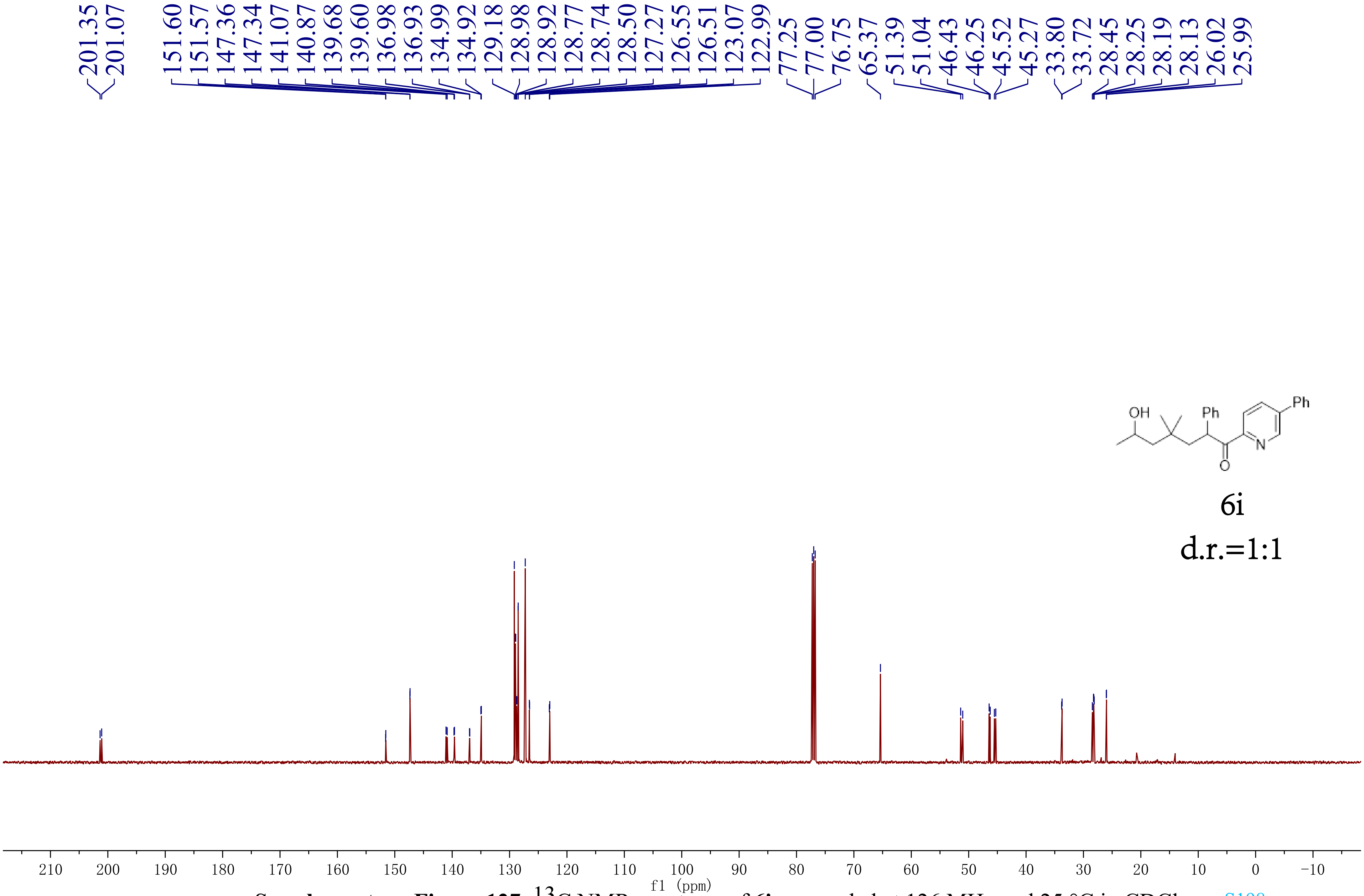

Supplementary Figure 127. ^{13}C NMR spectrum of **6i**, recorded at 126 MHz and 25 °C in $CDCl_3$

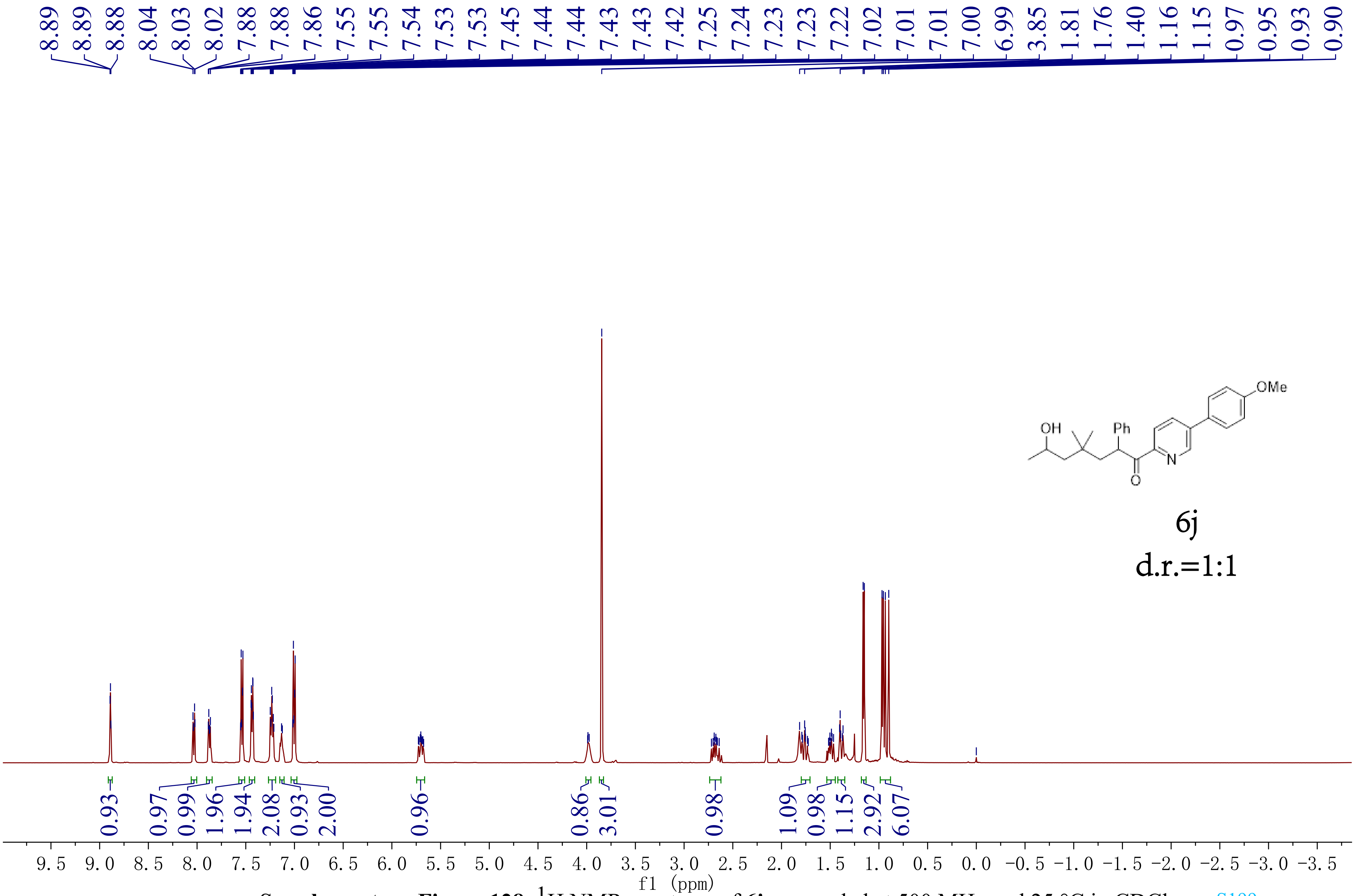

6j

d.r.=1:1

Supplementary Figure 128. ^{1}H NMR spectrum of **6j**, recorded at 500 MHz and 25 °C in $CDCl_3$

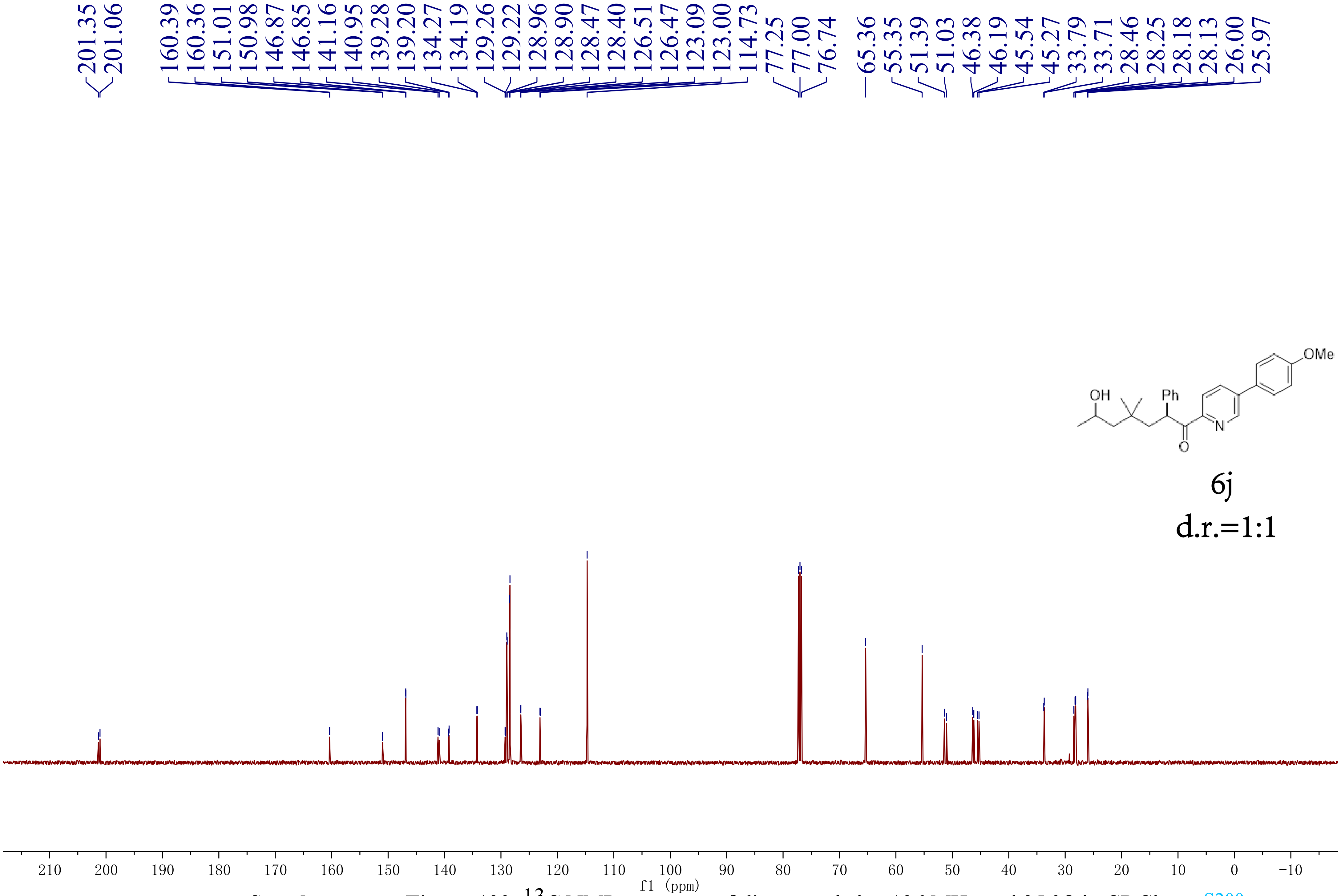

Supplementary Figure 129. ^{13}C NMR spectrum of **6j**, recorded at 126 MHz and 25 °C in $CDCl_3$

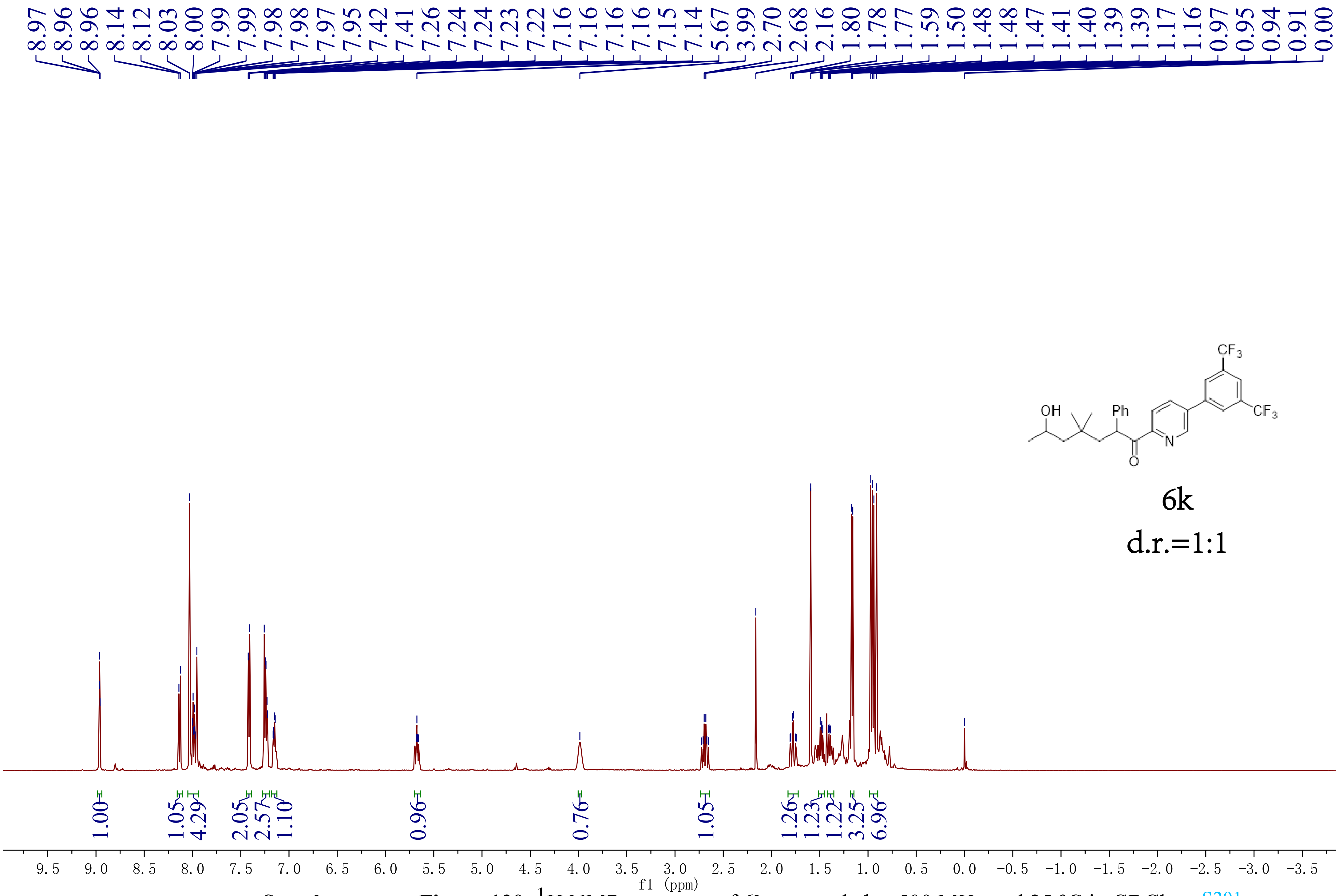

Supplementary Figure 130. ^{1}H NMR spectrum of **6k**, recorded at 500 MHz and 25 °C in $CDCl_3$

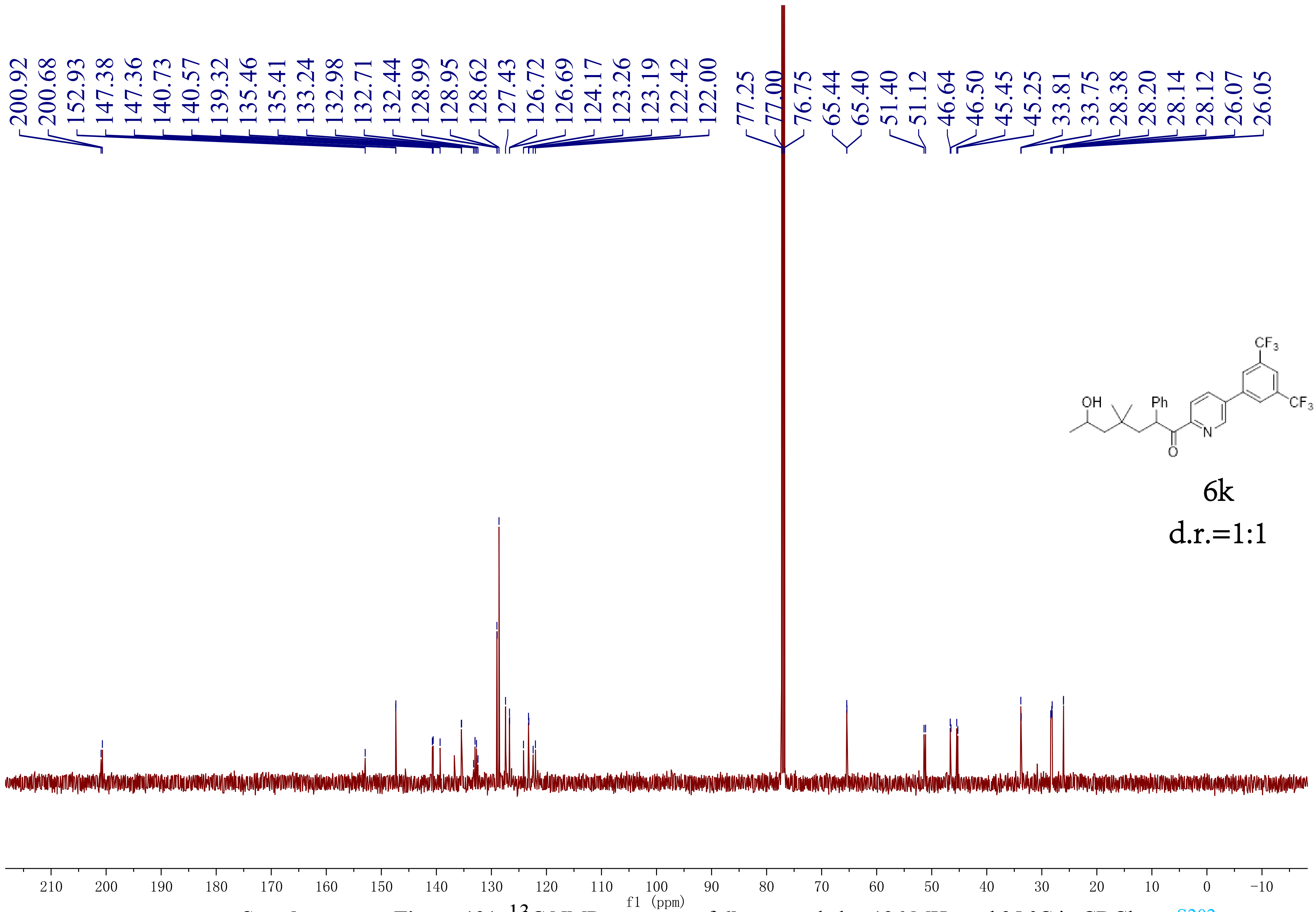

Supplementary Figure 131. ^{13}C NMR spectrum of **6k**, recorded at 126 MHz and 25 °C in $CDCl_3$

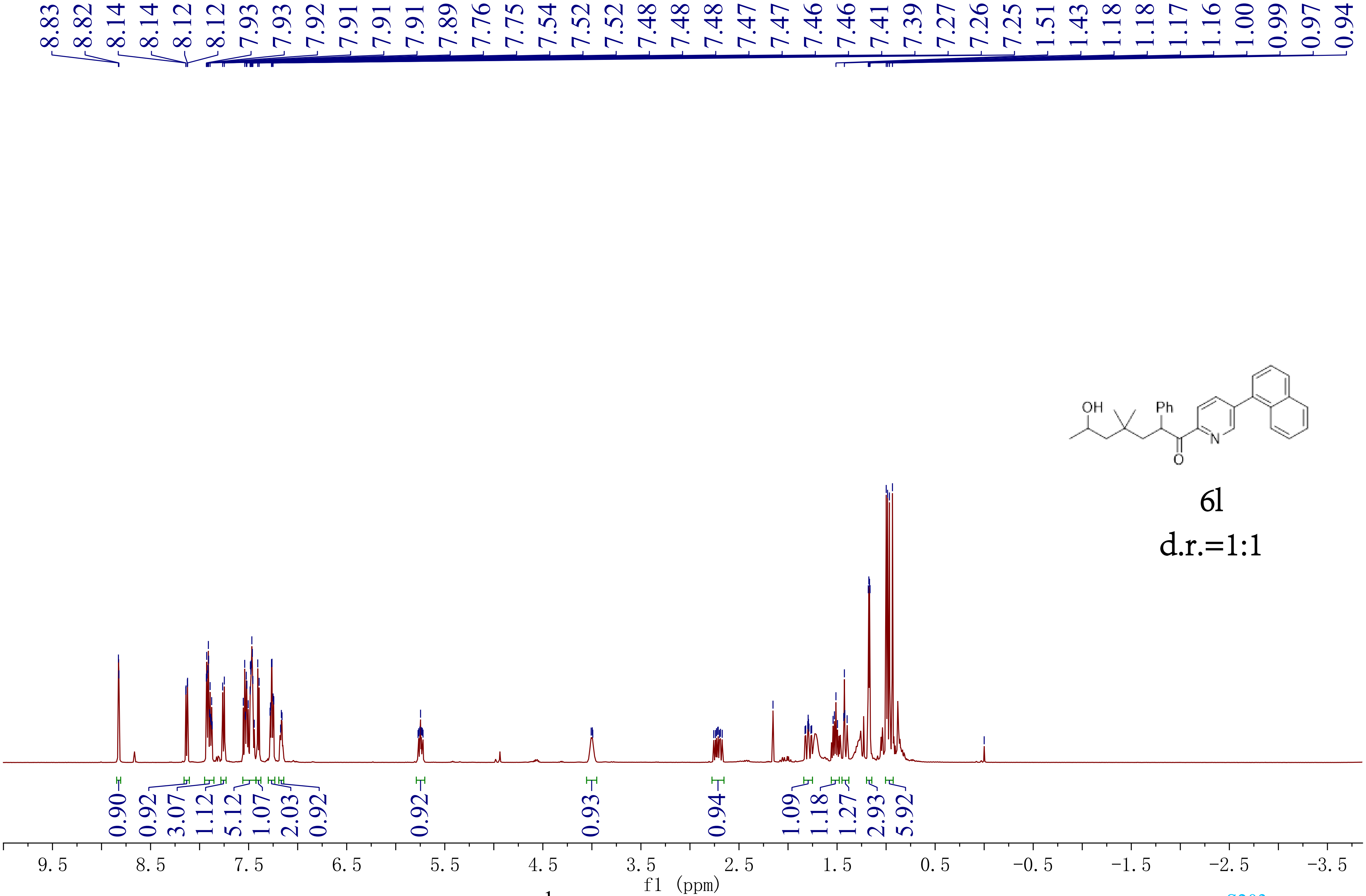

Supplementary Figure 132. ^{1}H NMR spectrum of **6l**, recorded at 500 MHz and 25 °C in $CDCl_3$

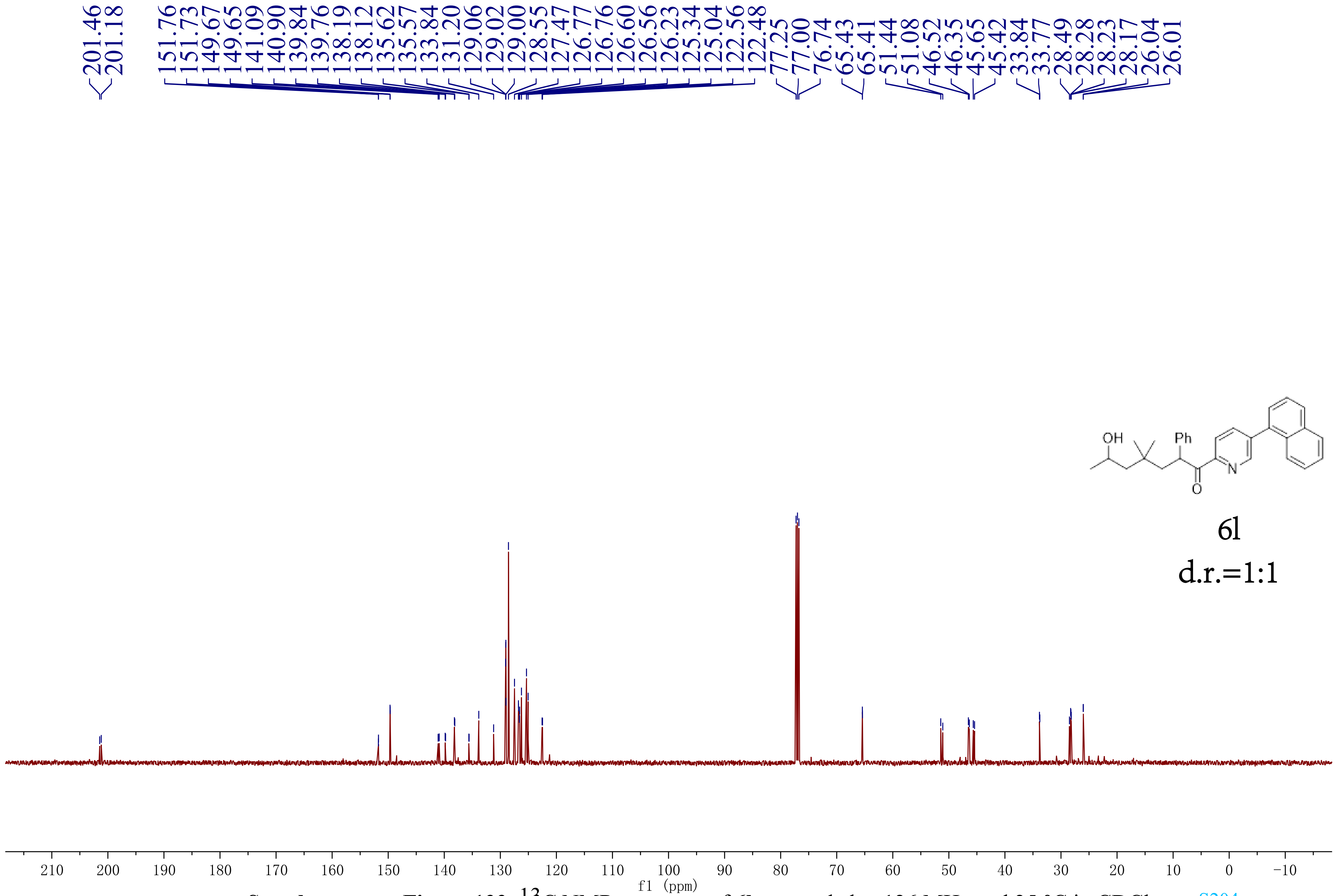

Supplementary Figure 133. ^{13}C NMR spectrum of **6l**, recorded at 126 MHz and 25 °C in $CDCl_3$

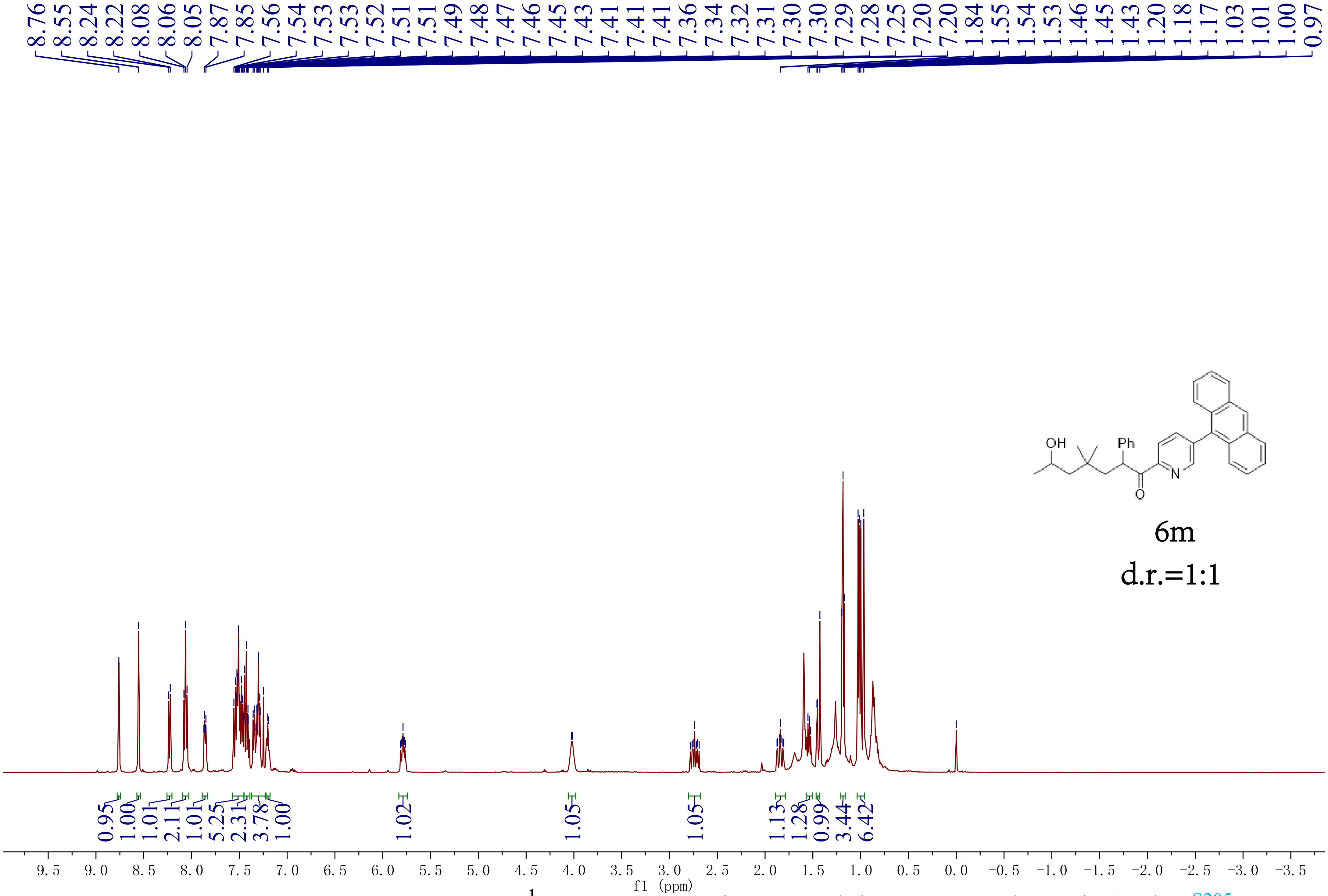

Supplementary Figure 134. ^{1}H NMR spectrum of **6m**, recorded at 500 MHz and 25 °C in $CDCl_3$

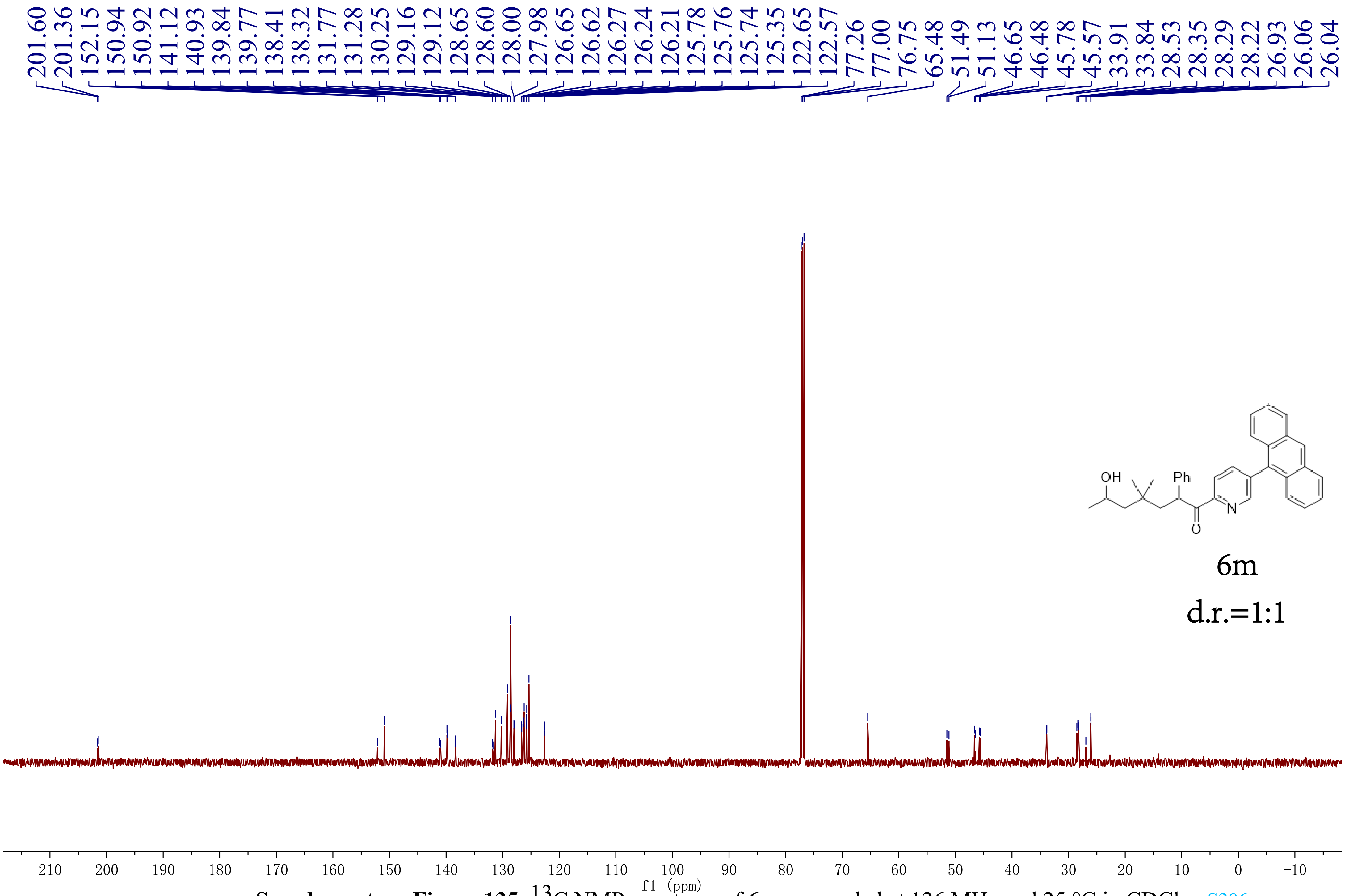

Supplementary Figure 135. ^{13}C NMR spectrum of **6m**, recorded at 126 MHz and 25 °C in $CDCl_3$

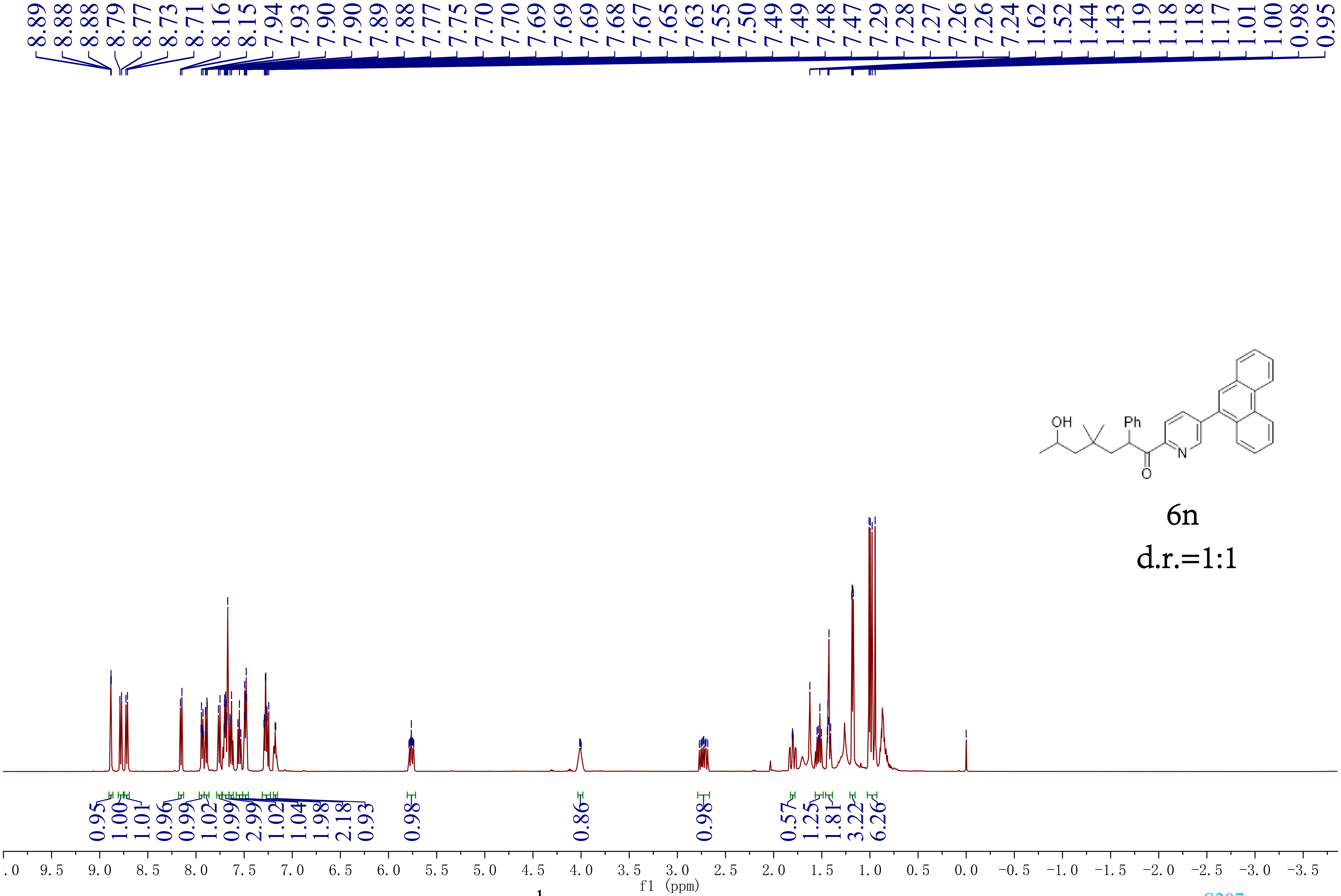

Supplementary Figure 136. ^{1}H NMR spectrum of **6n**, recorded at 500 MHz and 25 °C in $CDCl_3$

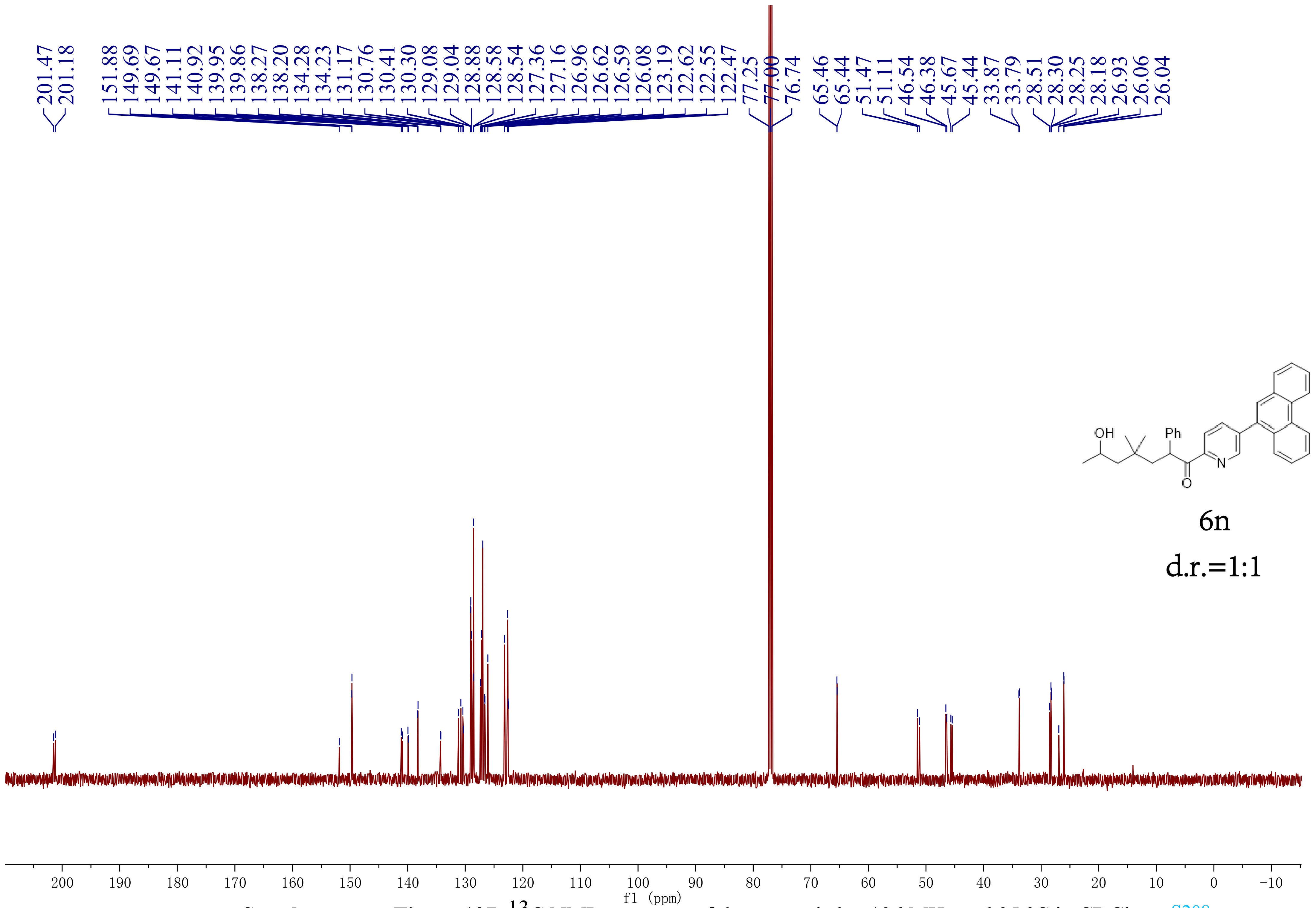

Supplementary Figure 137. ^{13}C NMR spectrum of **6n**, recorded at 126 MHz and 25 °C in $CDCl_3$

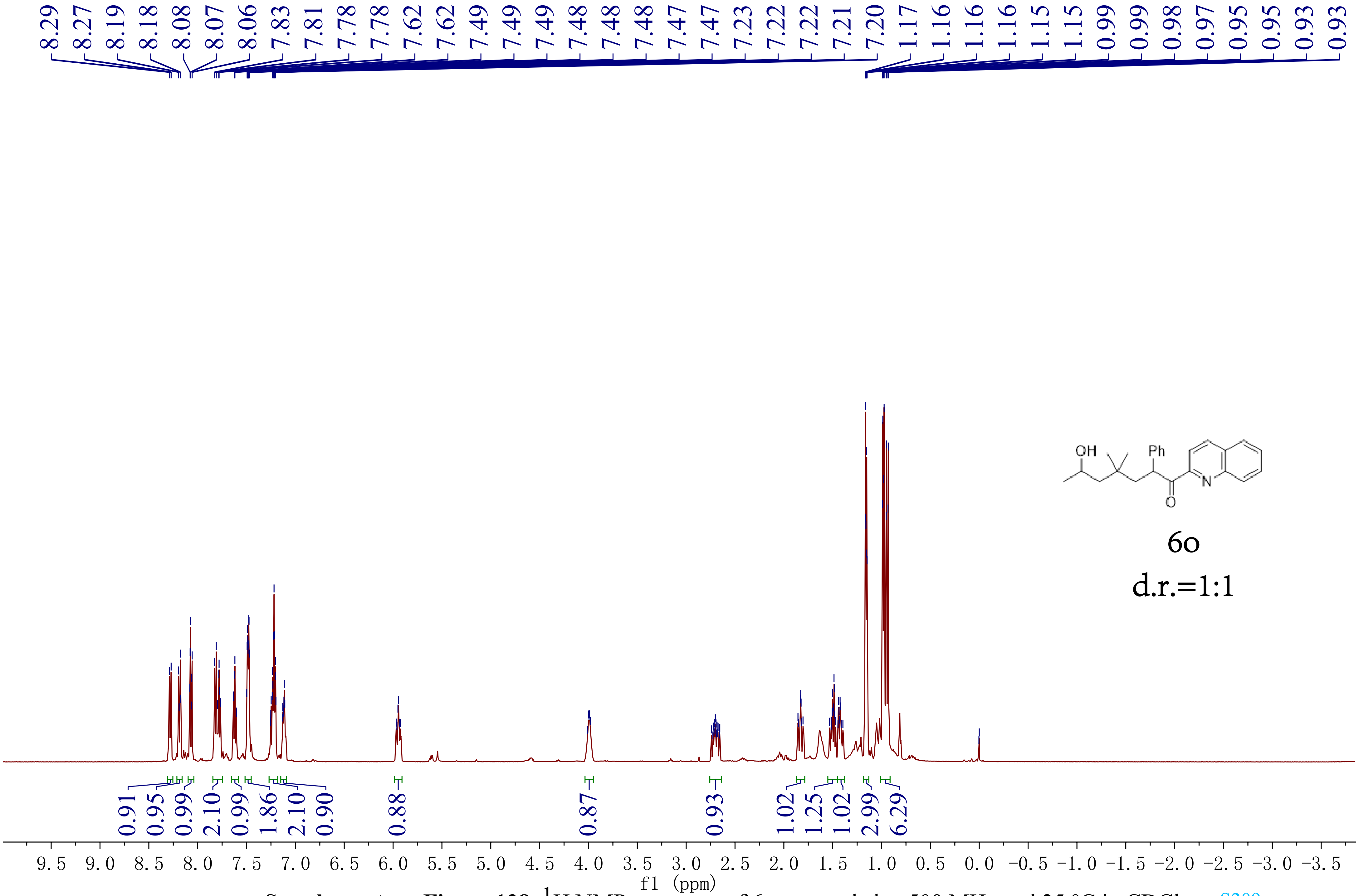

Supplementary Figure 138. ^{1}H NMR spectrum of **6o**, recorded at 500 MHz and 25 °C in $CDCl_3$

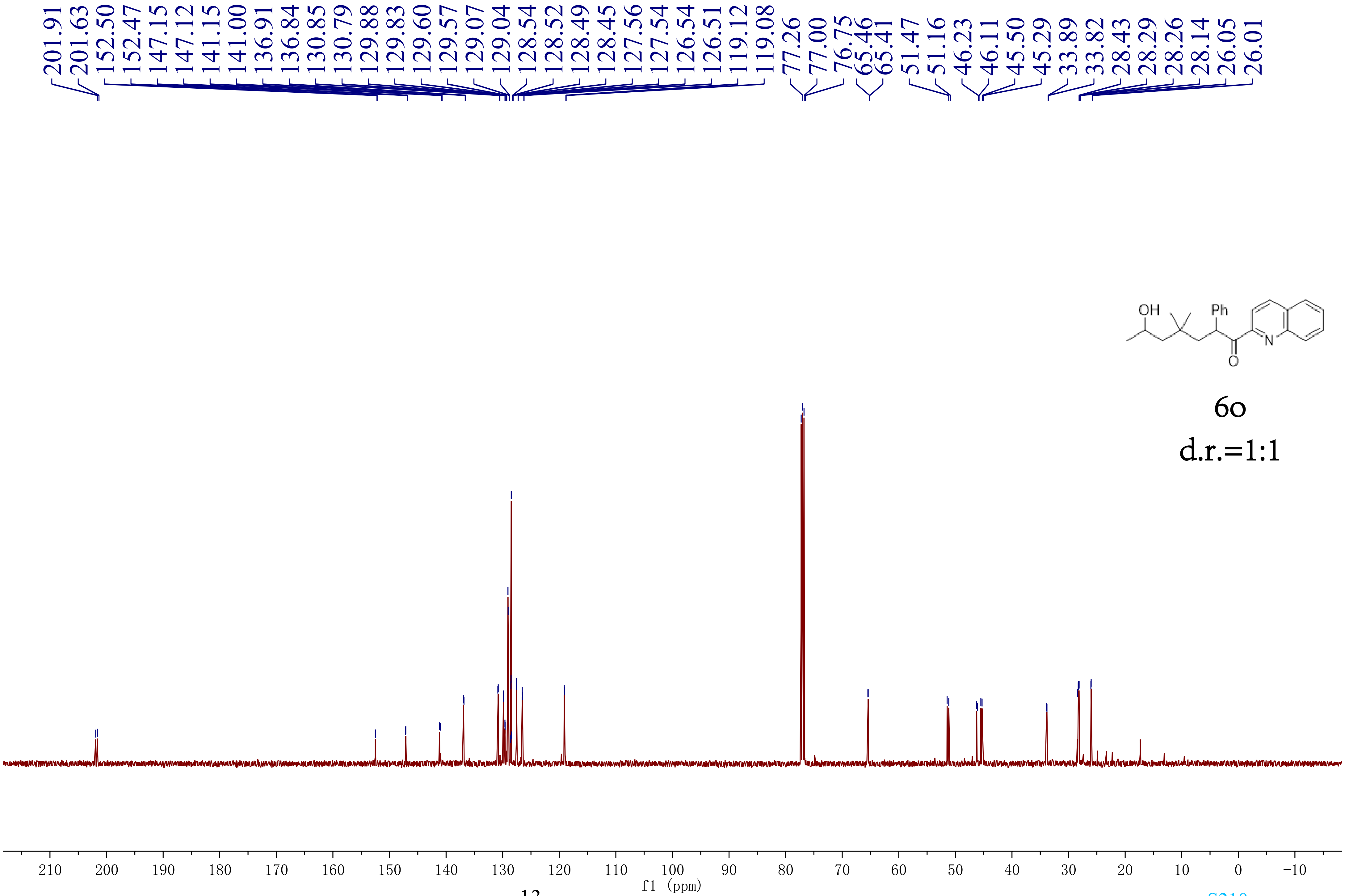

Supplementary Figure 139. ^{13}C NMR spectrum of **6o**, recorded at 126 MHz and 25 °C in $CDCl_3$

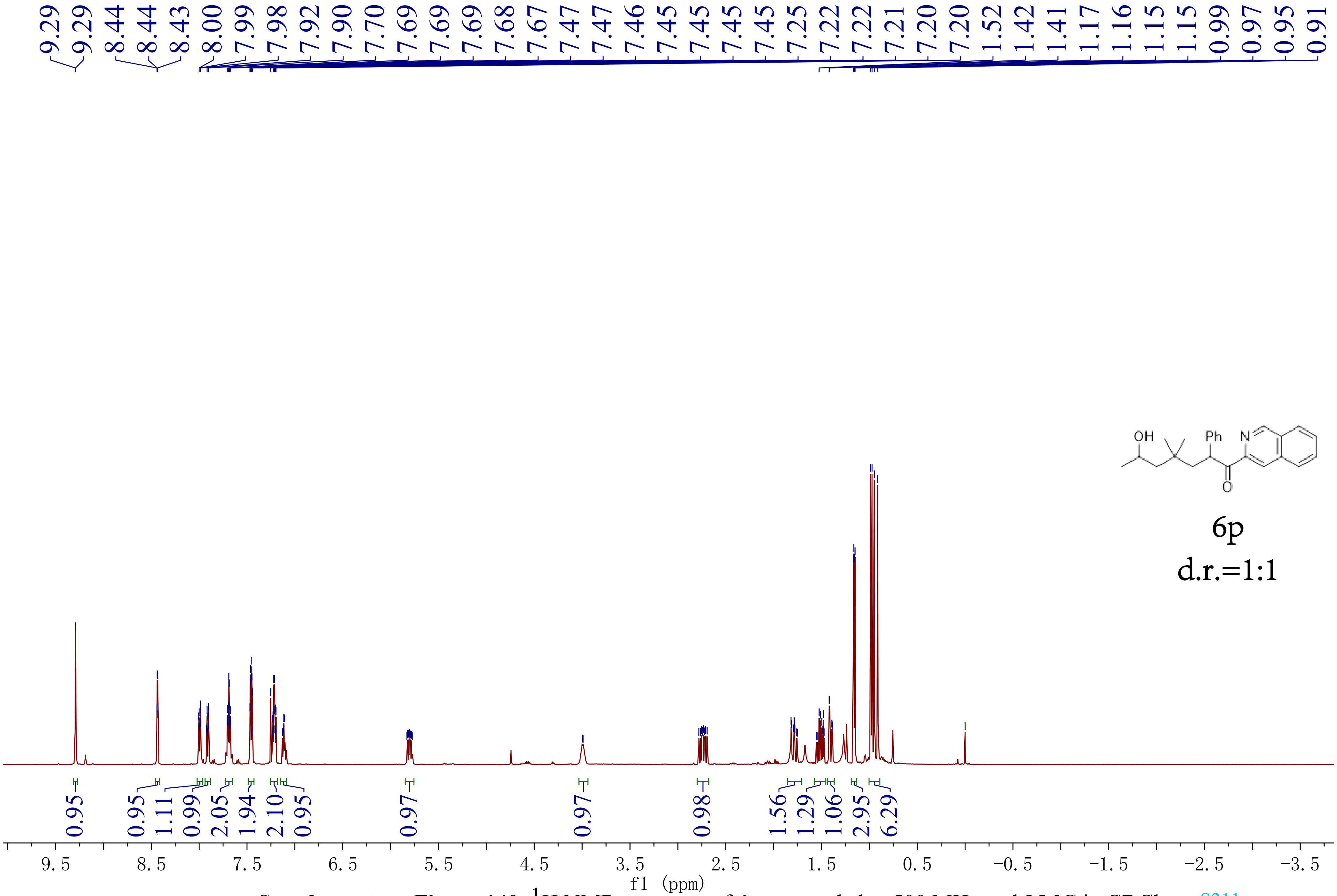

Supplementary Figure 140. ^{1}H NMR spectrum of **6p**, recorded at 500 MHz and 25 °C in $CDCl_3$

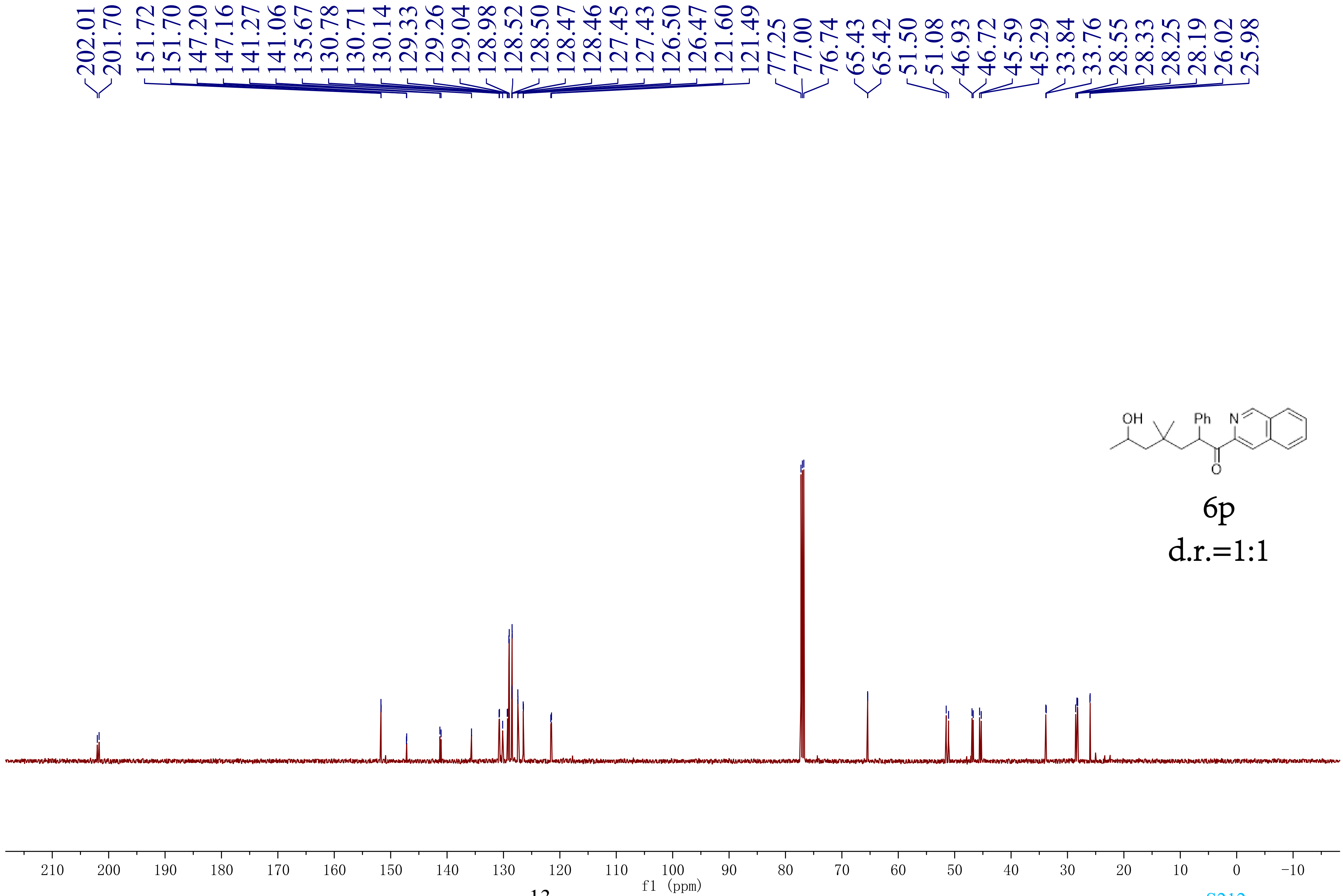

Supplementary Figure 141. ^{13}C NMR spectrum of **6p**, recorded at 126 MHz and 25 °C in $CDCl_3$

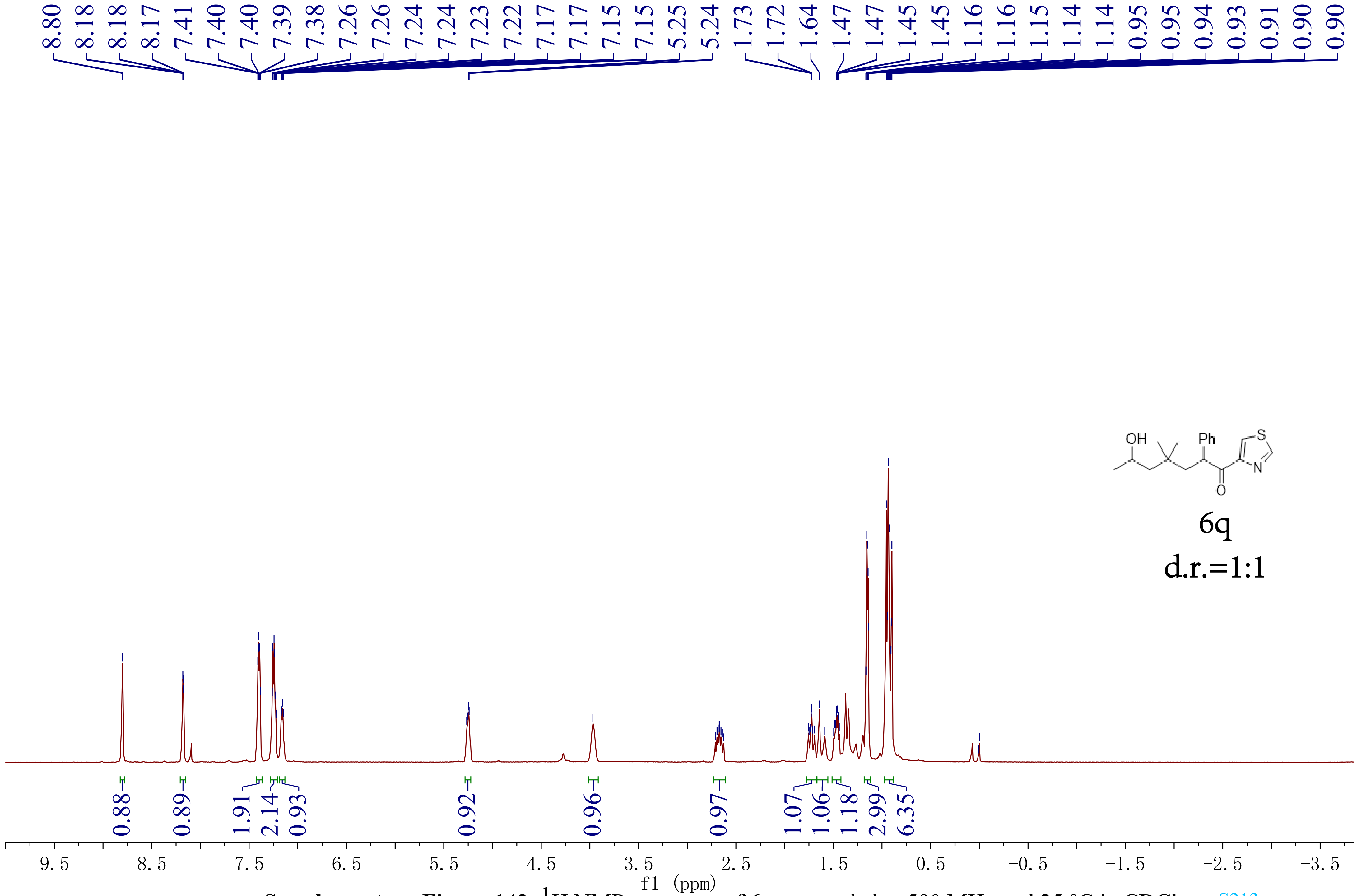

Supplementary Figure 142. ^{1}H NMR spectrum of **6q**, recorded at 500 MHz and 25 °C in $CDCl_3$

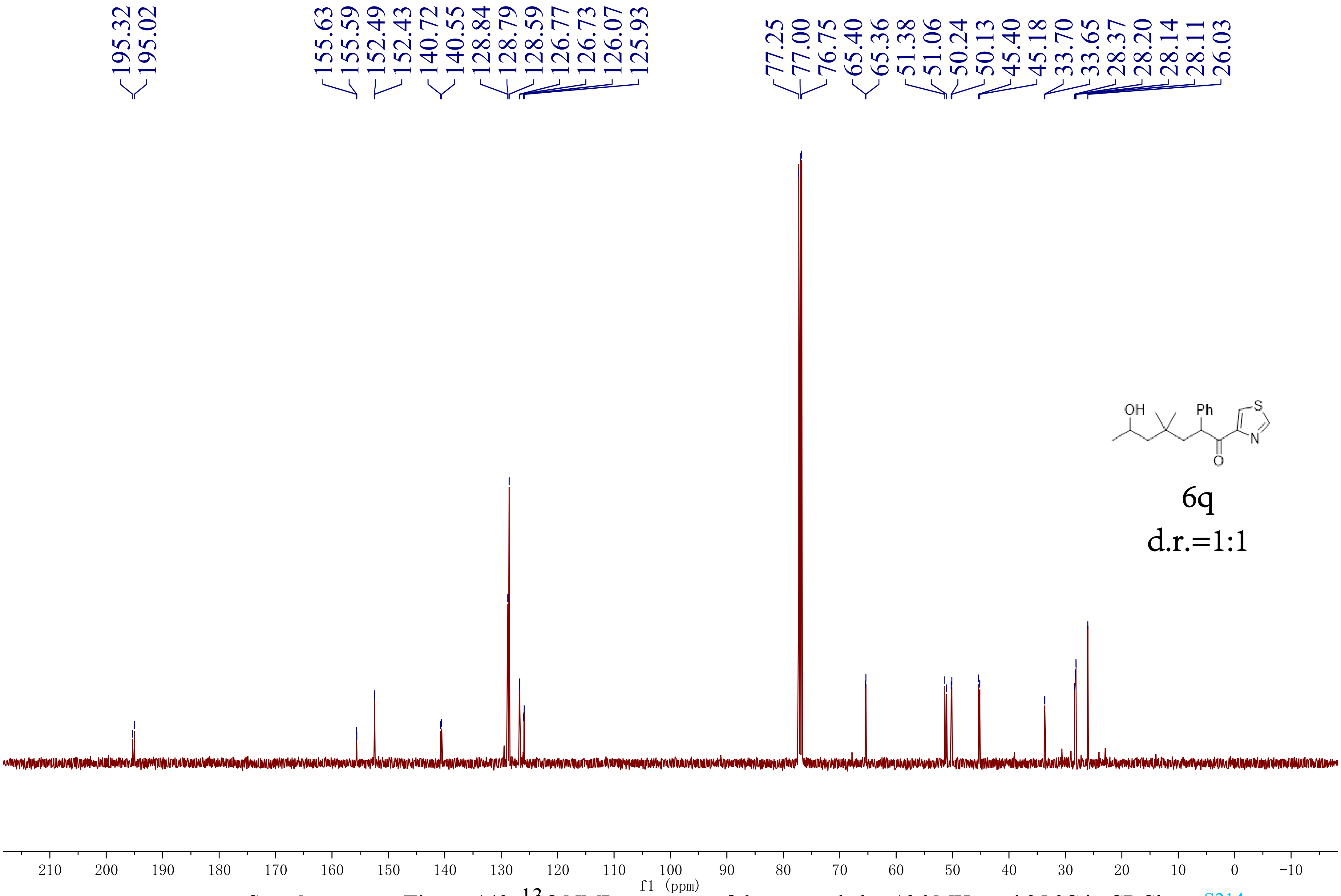

6q

d.r.=1:1

Supplementary Figure 143. ^{13}C NMR spectrum of **6q**, recorded at 126 MHz and 25 °C in $CDCl_3$

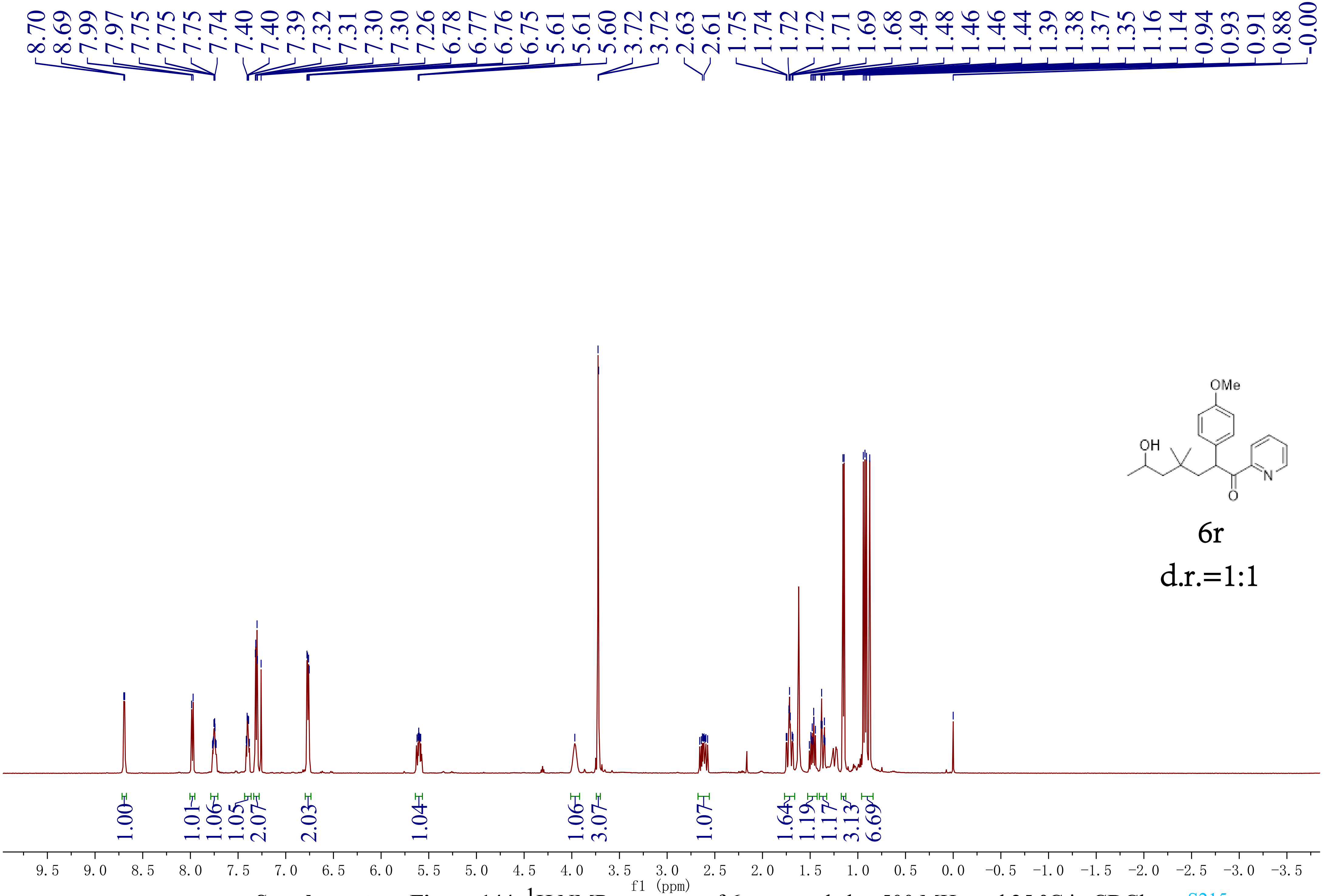

Supplementary Figure 144. ^{1}H NMR spectrum of **6r**, recorded at 500 MHz and 25 °C in $CDCl_3$

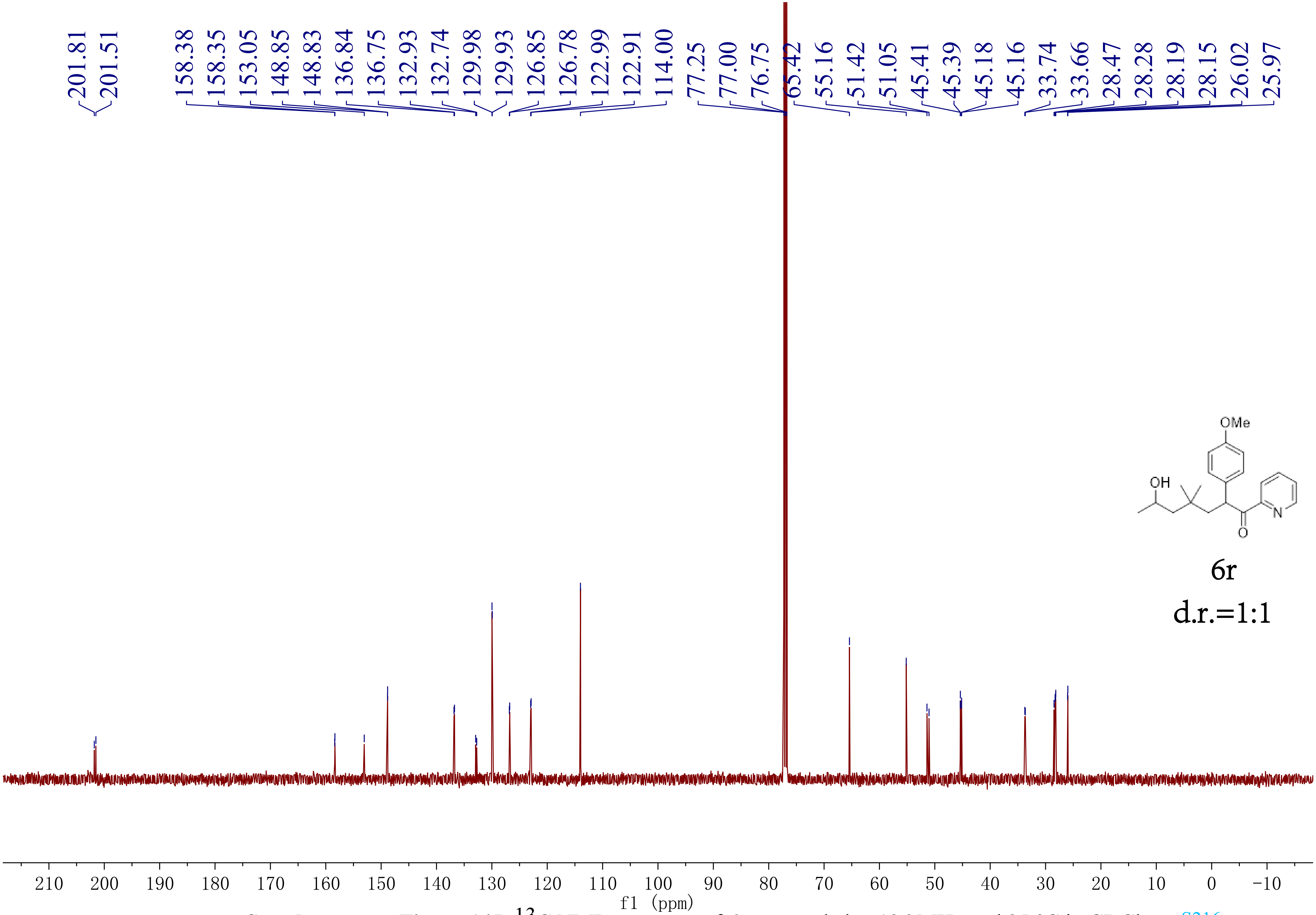

6r

d.r.=1:1

Supplementary Figure 145. ^{13}C NMR spectrum of **6r**, recorded at 126 MHz and 25 °C in $CDCl_3$

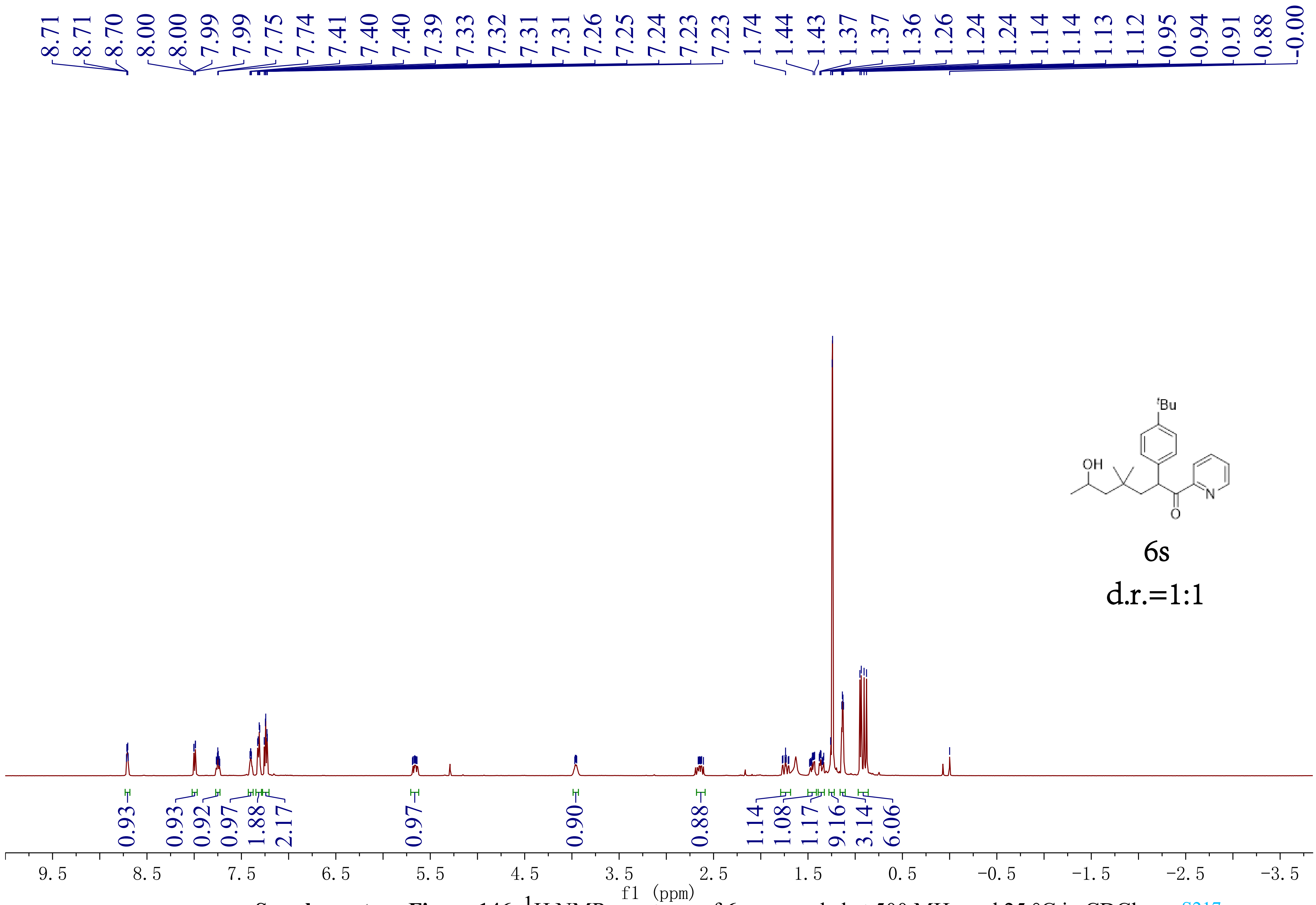

Supplementary Figure 146. ^{1}H NMR spectrum of **6s**, recorded at 500 MHz and 25 °C in $CDCl_3$

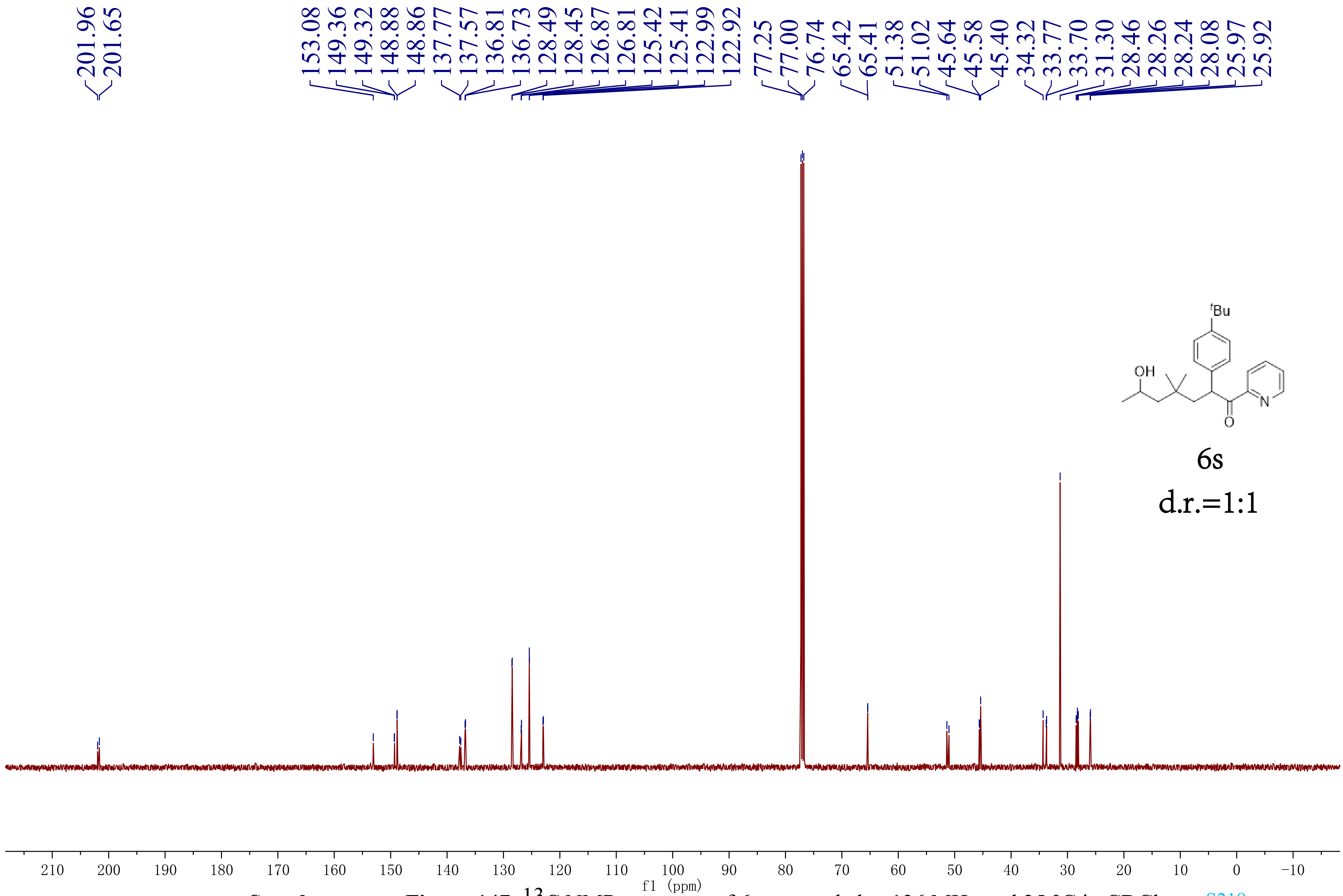

Supplementary Figure 147. ^{13}C NMR spectrum of **6s**, recorded at 126 MHz and 25 °C in $CDCl_3$

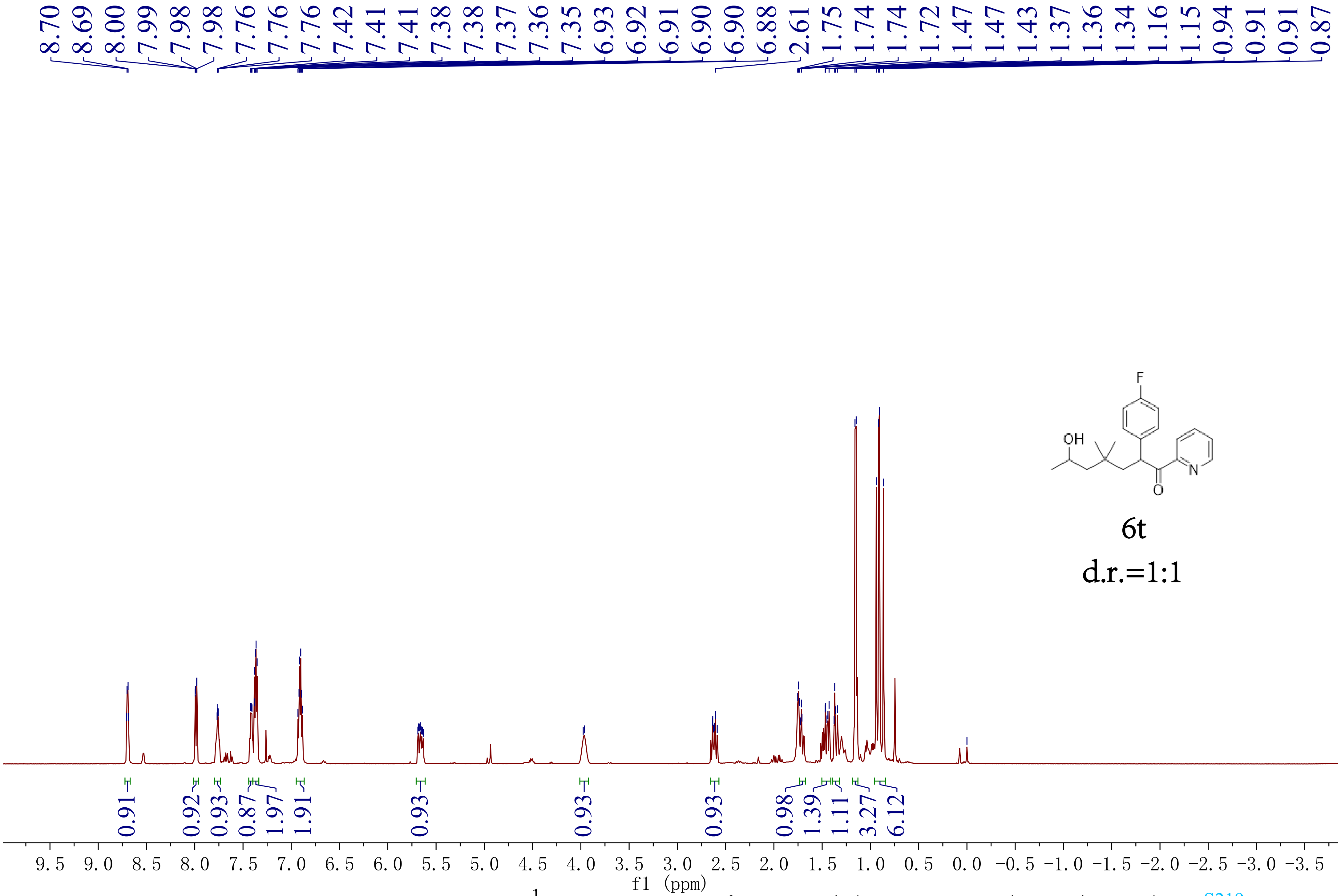

Supplementary Figure 148. ^{1}H NMR spectrum of **6t**, recorded at 500 MHz and 25 °C in $CDCl_3$

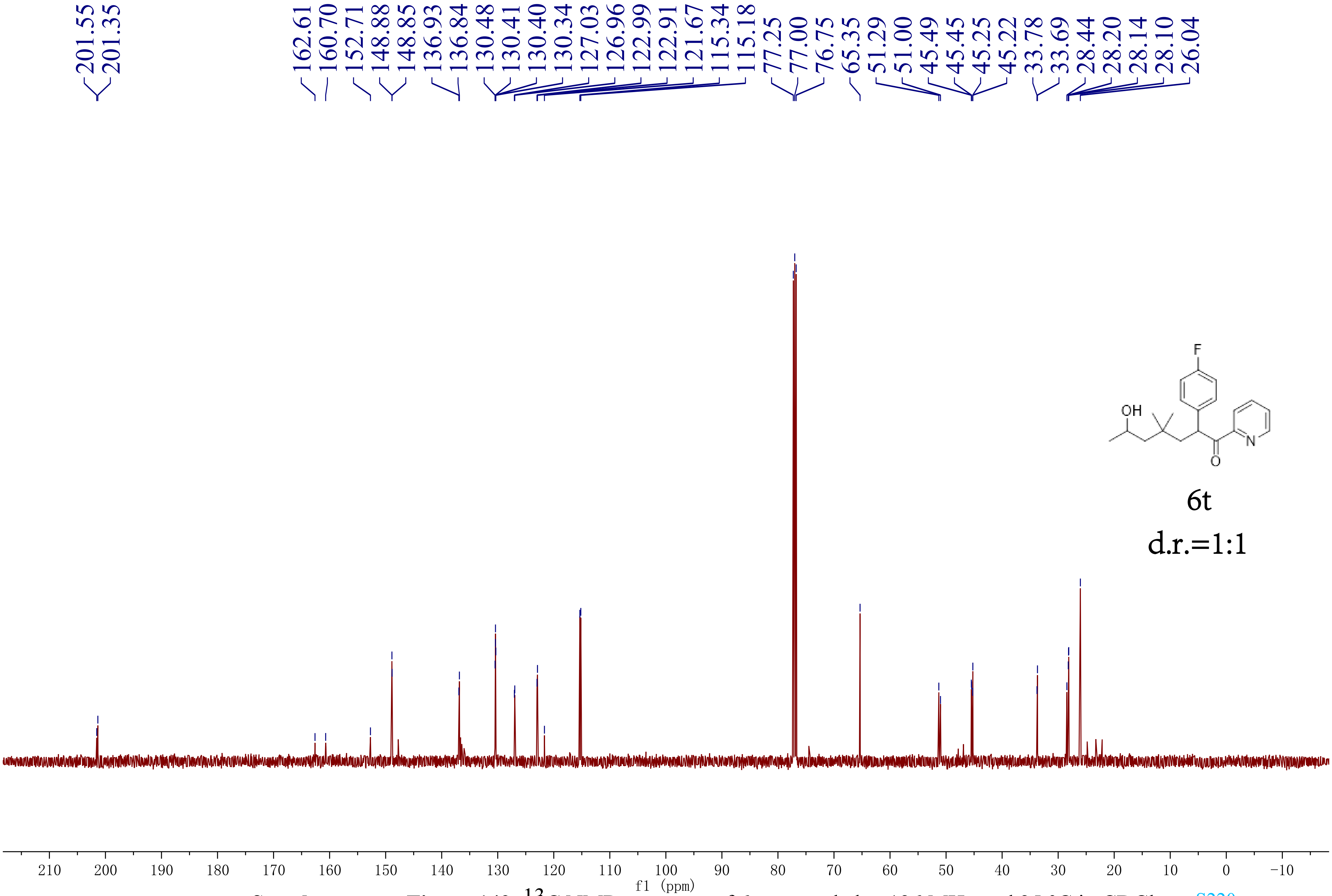

Supplementary Figure 149. ^{13}C NMR spectrum of **6t**, recorded at 126 MHz and 25 °C in $CDCl_3$

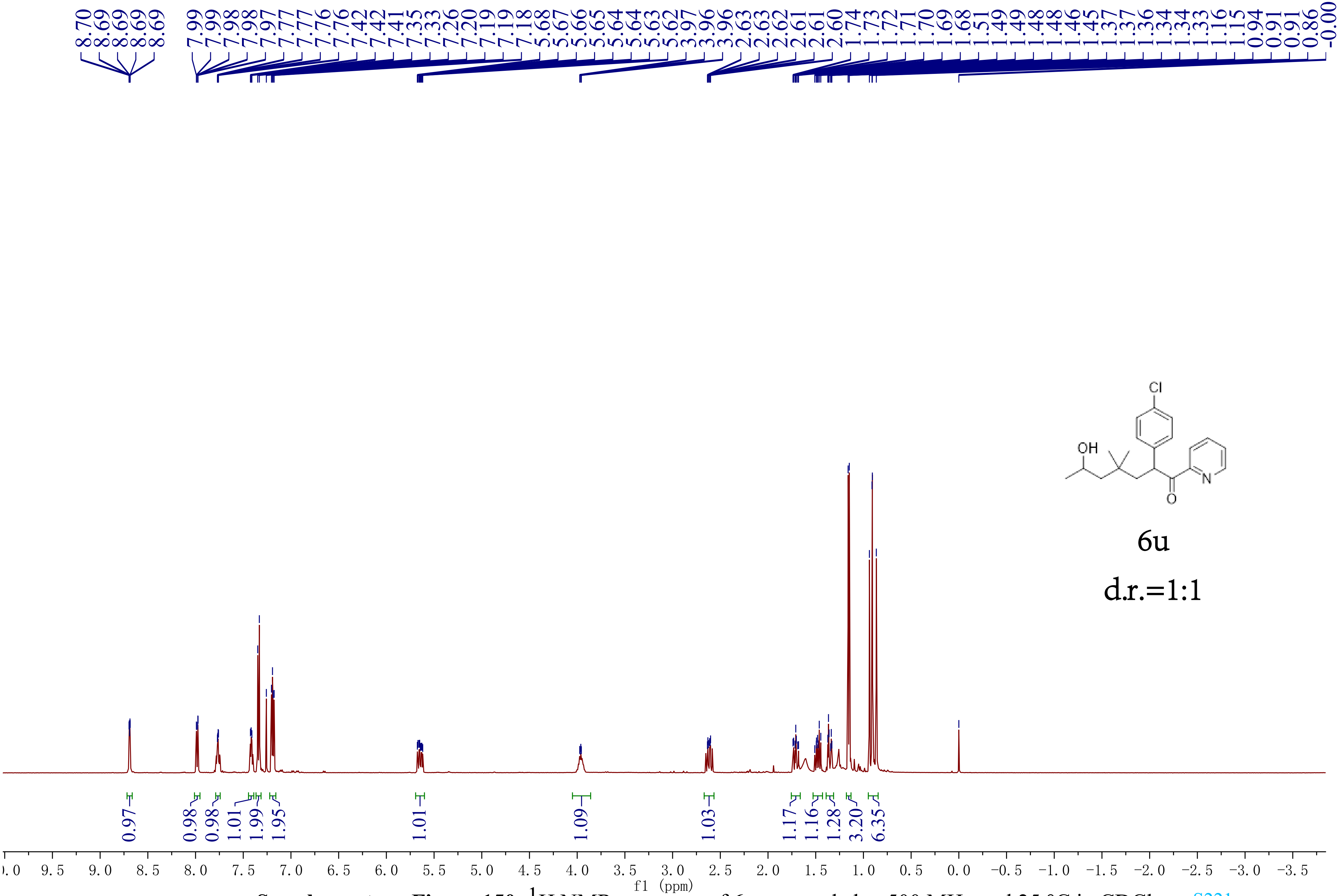

Supplementary Figure 150. ^{1}H NMR spectrum of **6u**, recorded at 500 MHz and 25 °C in $CDCl_3$

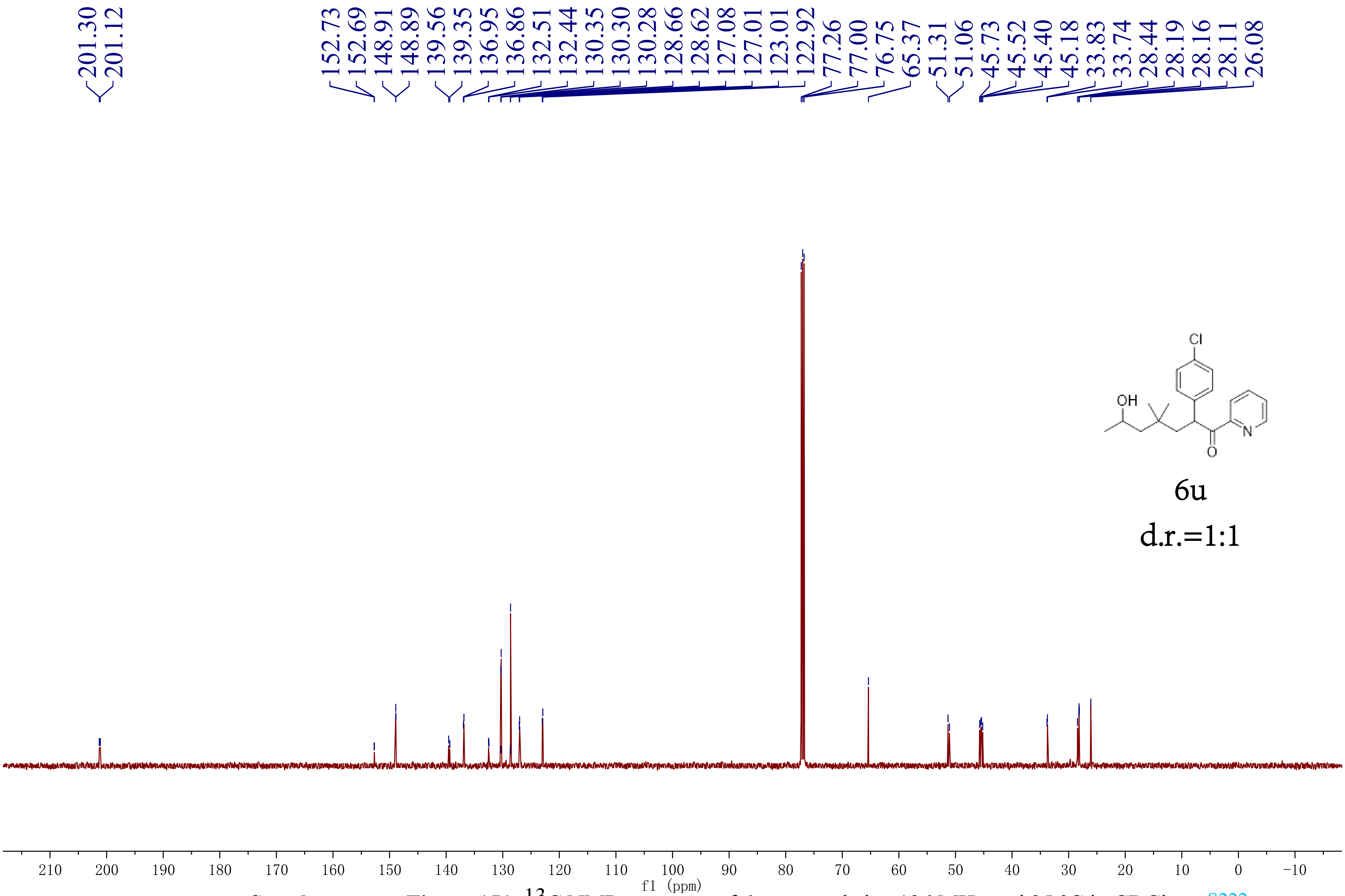

6u

d.r.=1:1

Supplementary Figure 151. ^{13}C NMR spectrum of **6u**, recorded at 126 MHz and 25 °C in $CDCl_3$

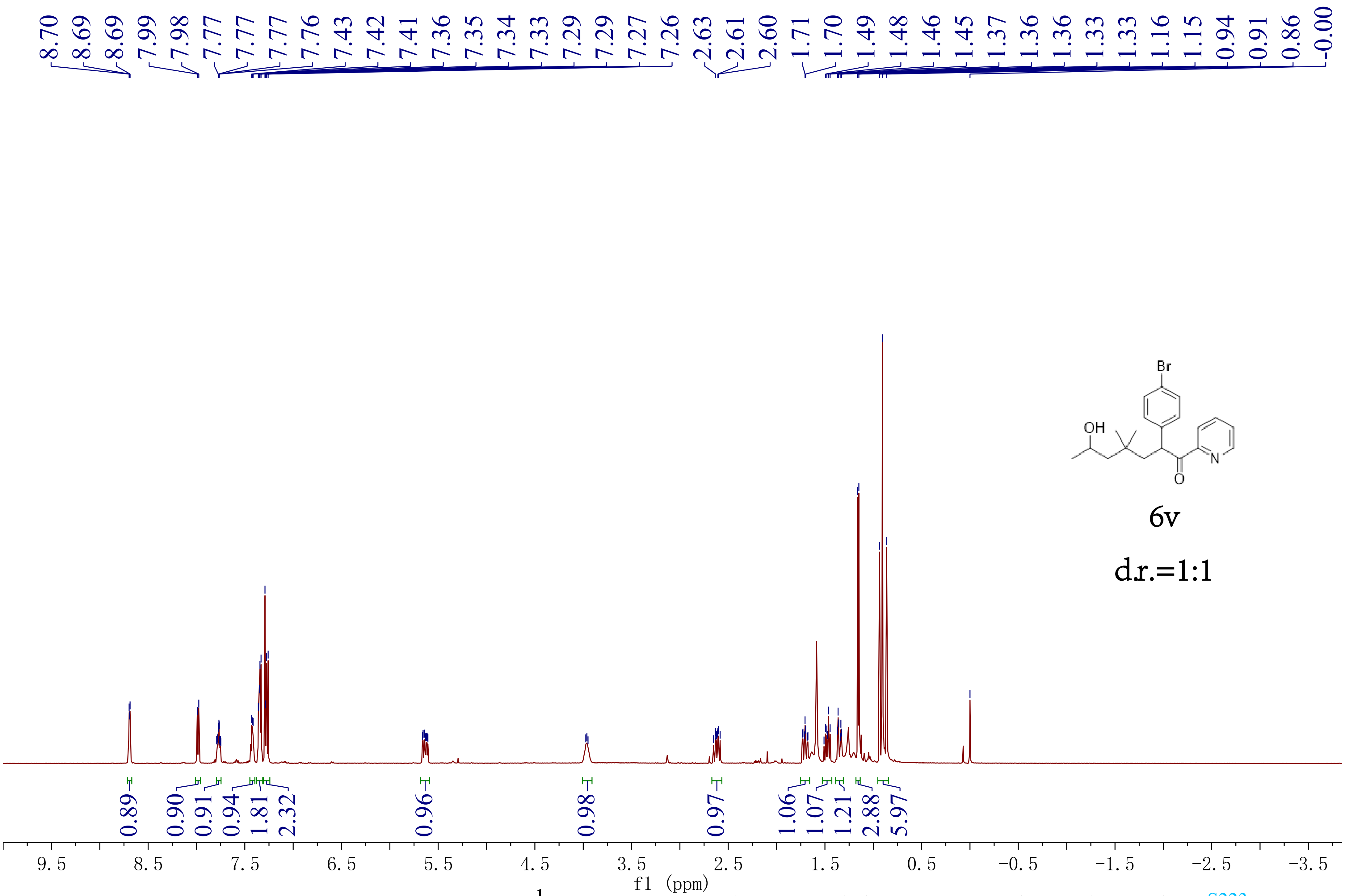

Supplementary Figure 152. ^{1}H NMR spectrum of **6v**, recorded at 500 MHz and 25 °C in $CDCl_3$

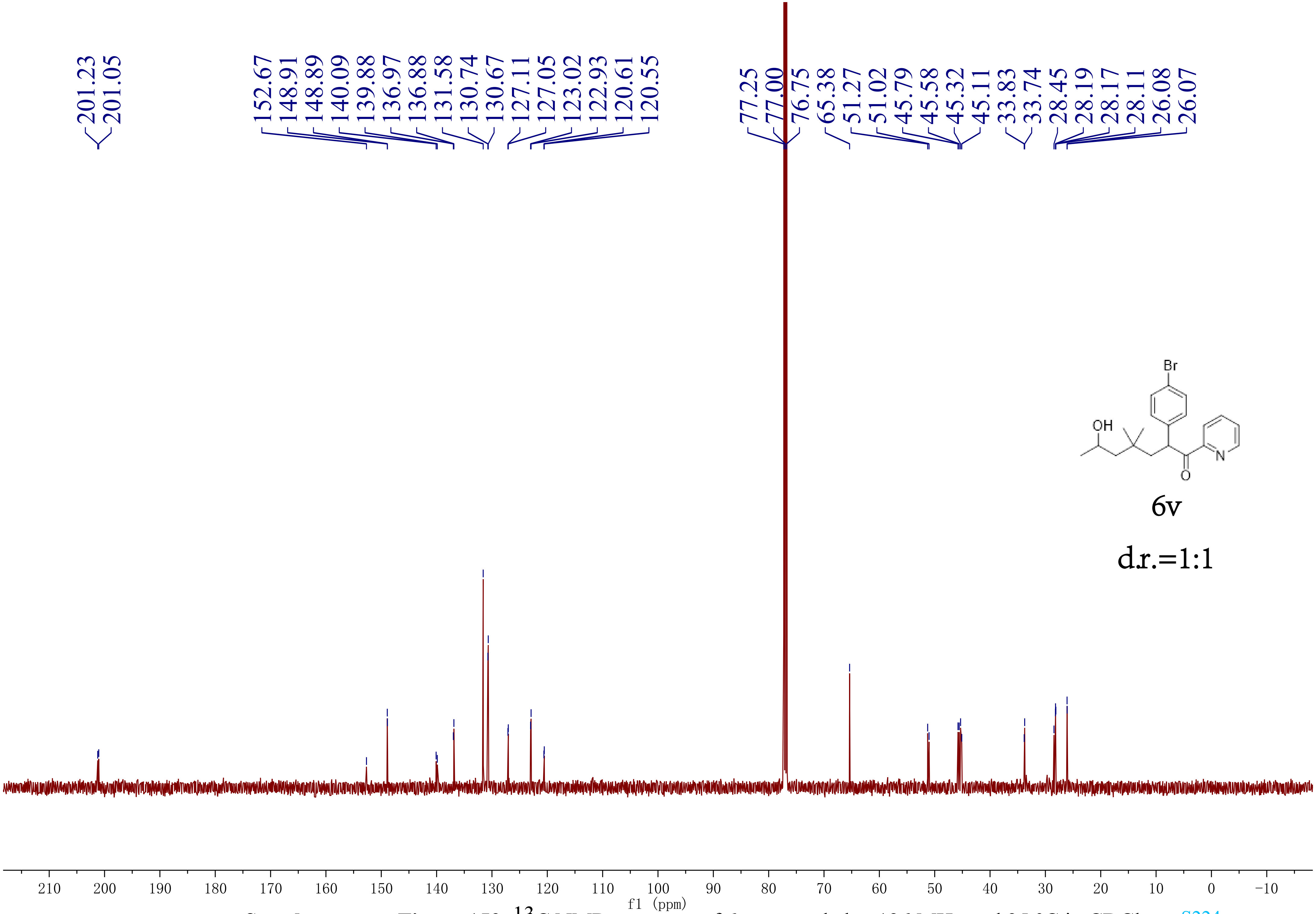

Supplementary Figure 153. ^{13}C NMR spectrum of **6v**, recorded at 126 MHz and 25 °C in $CDCl_3$

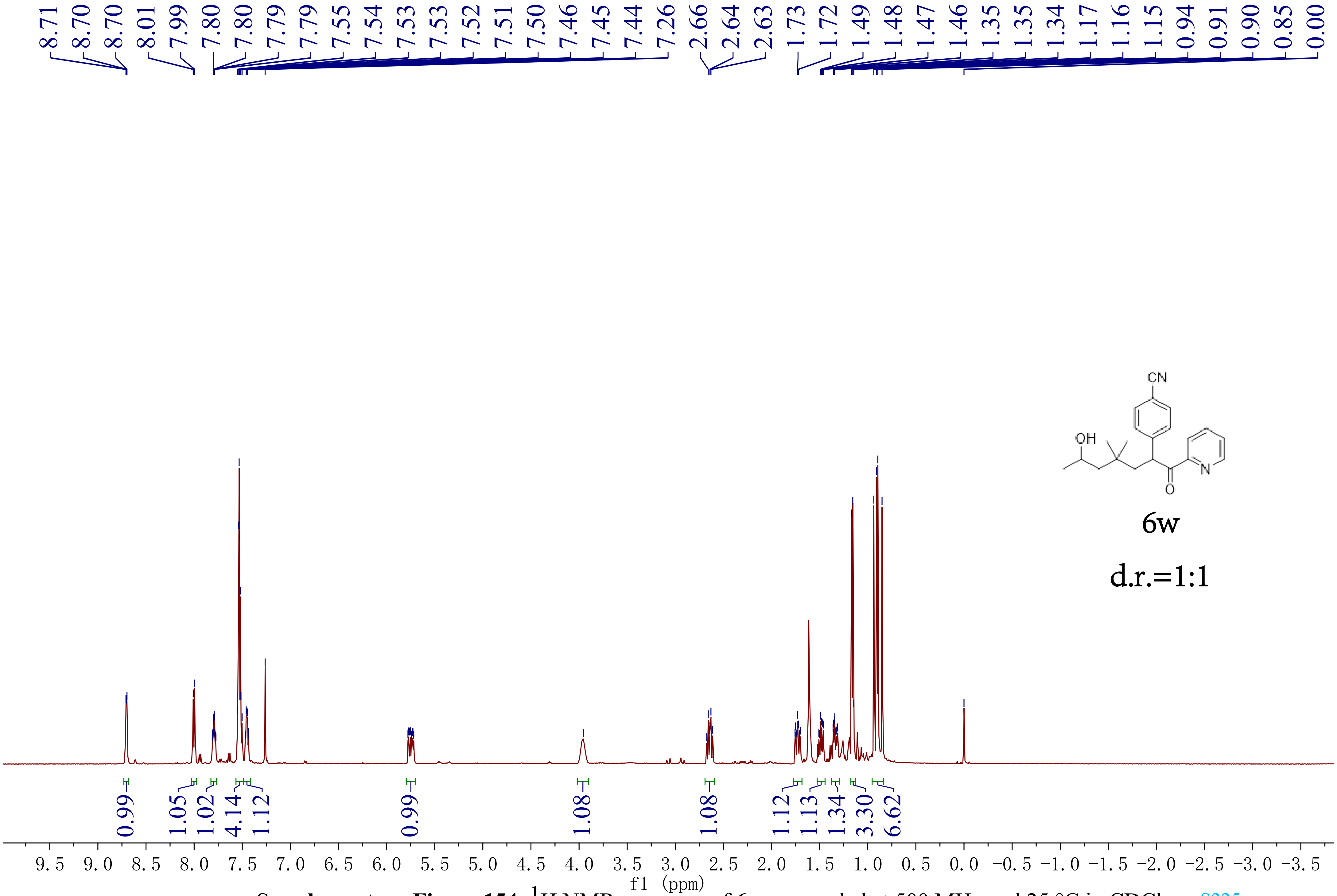

Supplementary Figure 154. ^{1}H NMR spectrum of **6w**, recorded at 500 MHz and 25 °C in $CDCl_3$

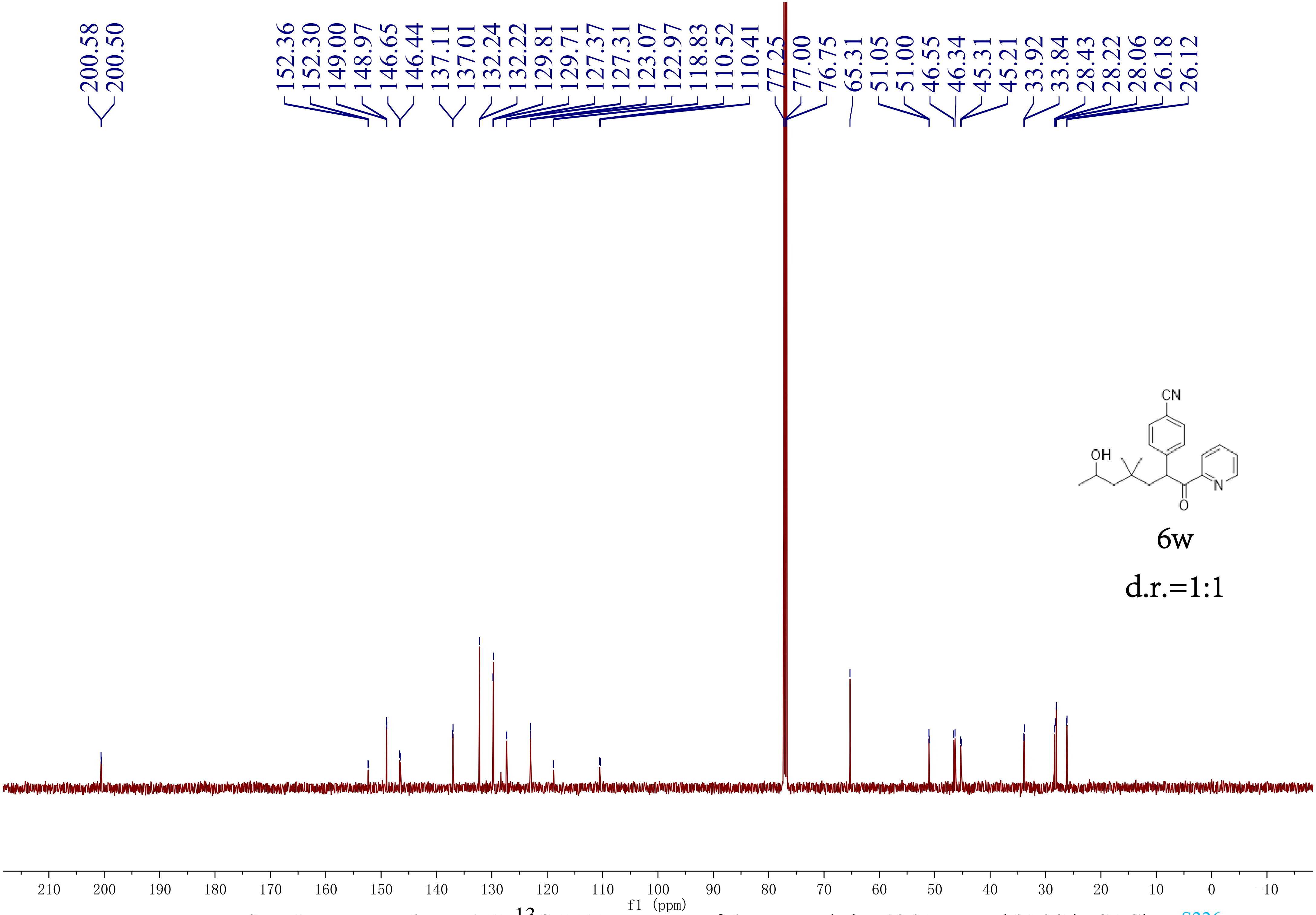

6w

d.r.=1:1

Supplementary Figure 155. ^{13}C NMR spectrum of **6w**, recorded at 126 MHz and 25 °C in $CDCl_3$

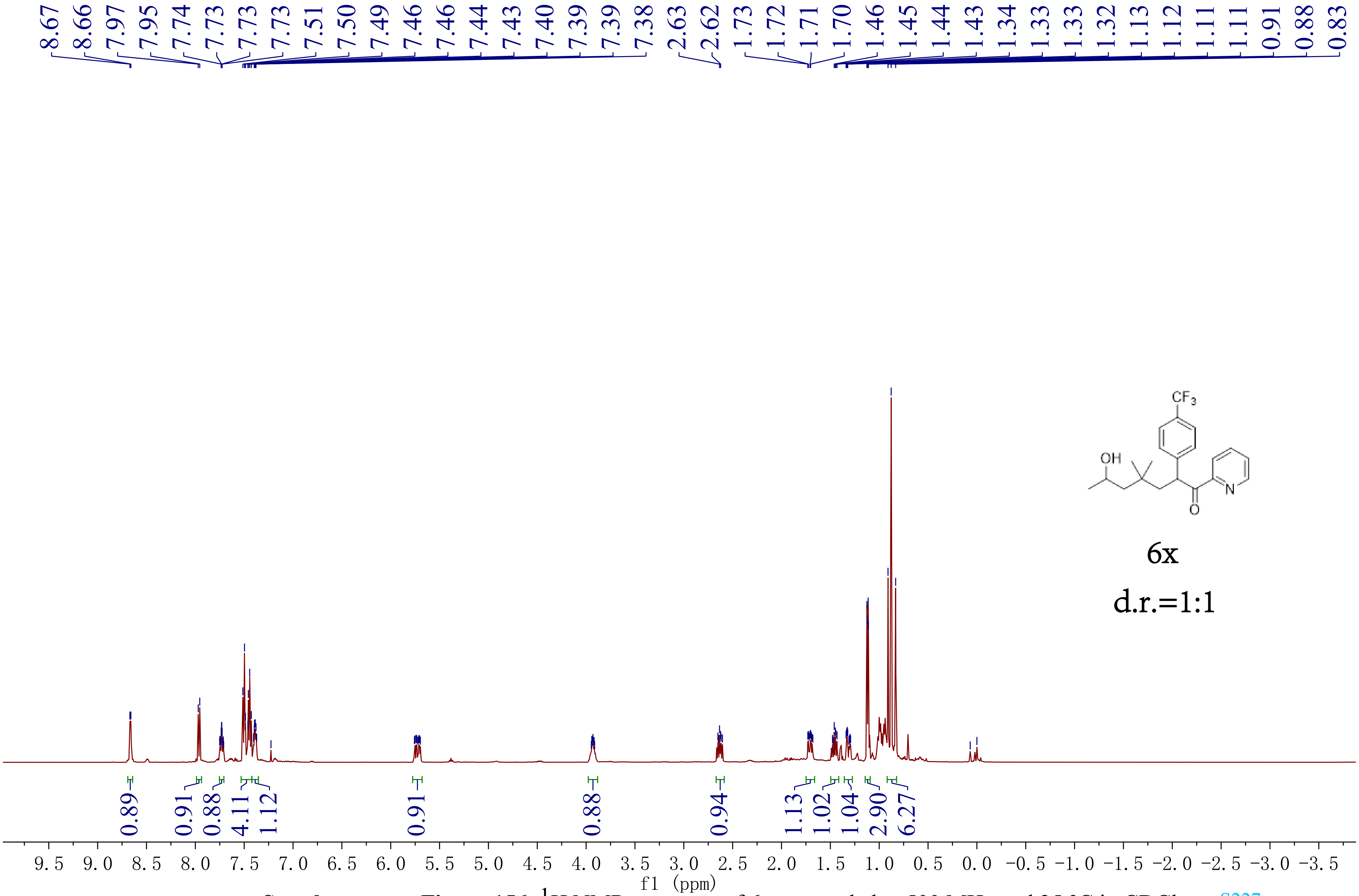

Supplementary Figure 156. ^{1}H NMR spectrum of **6x**, recorded at 500 MHz and 25 °C in $CDCl_3$

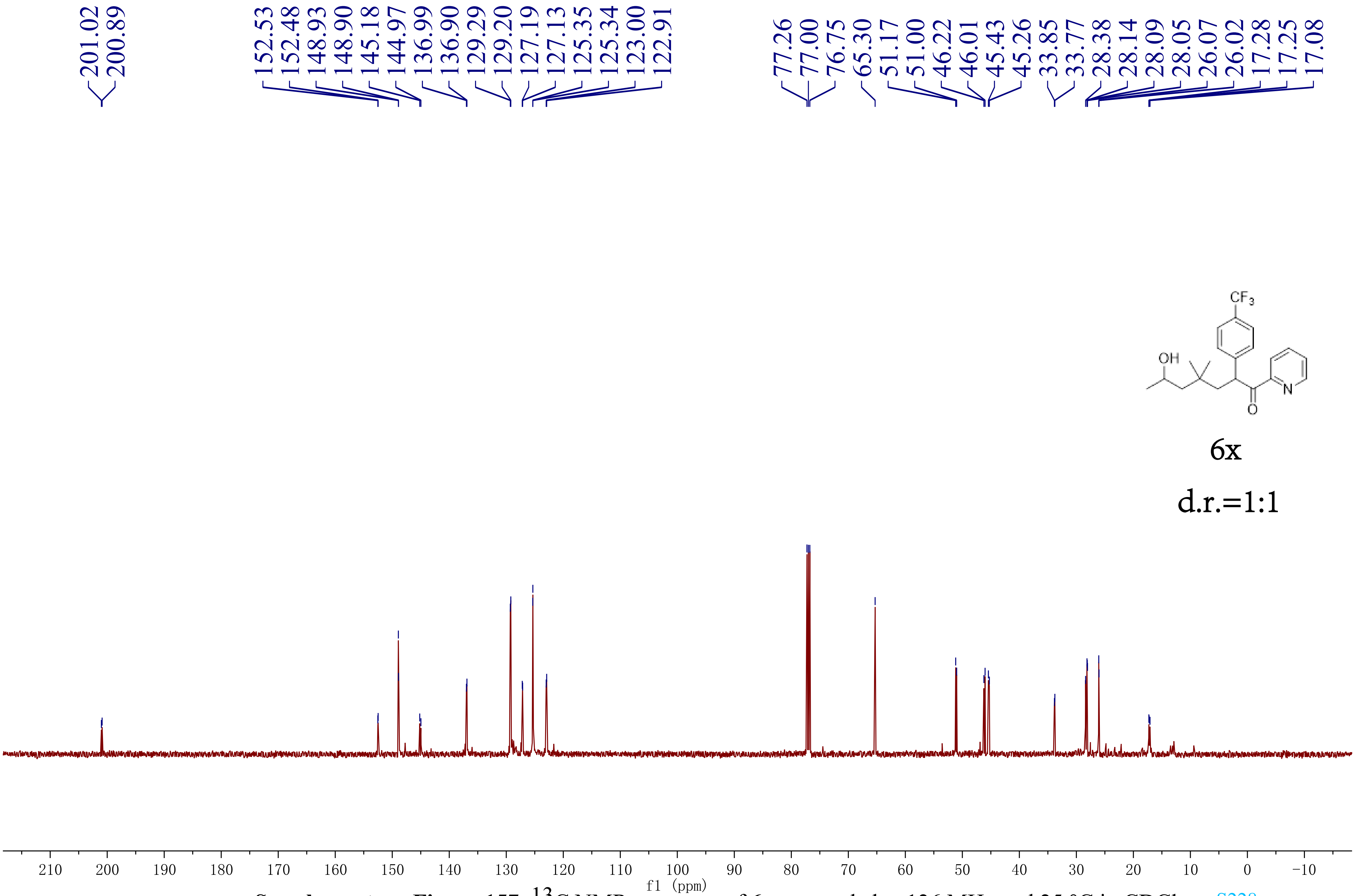

6x

d.r.=1:1

Supplementary Figure 157. ^{13}C NMR spectrum of **6x**, recorded at 126 MHz and 25 °C in $CDCl_3$

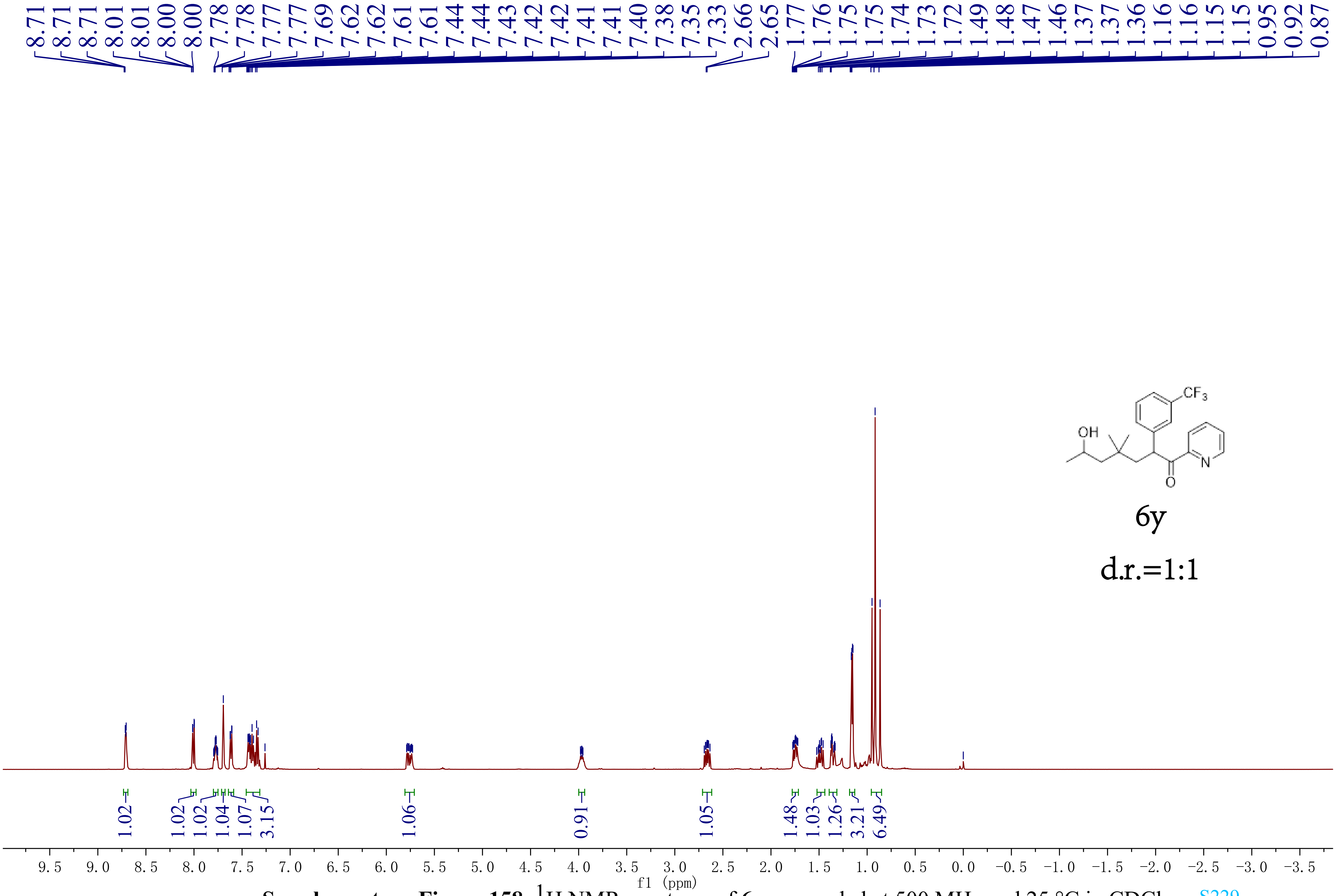

Supplementary Figure 158. ^{1}H NMR spectrum of **6y**, recorded at 500 MHz and 25 °C in $CDCl_3$

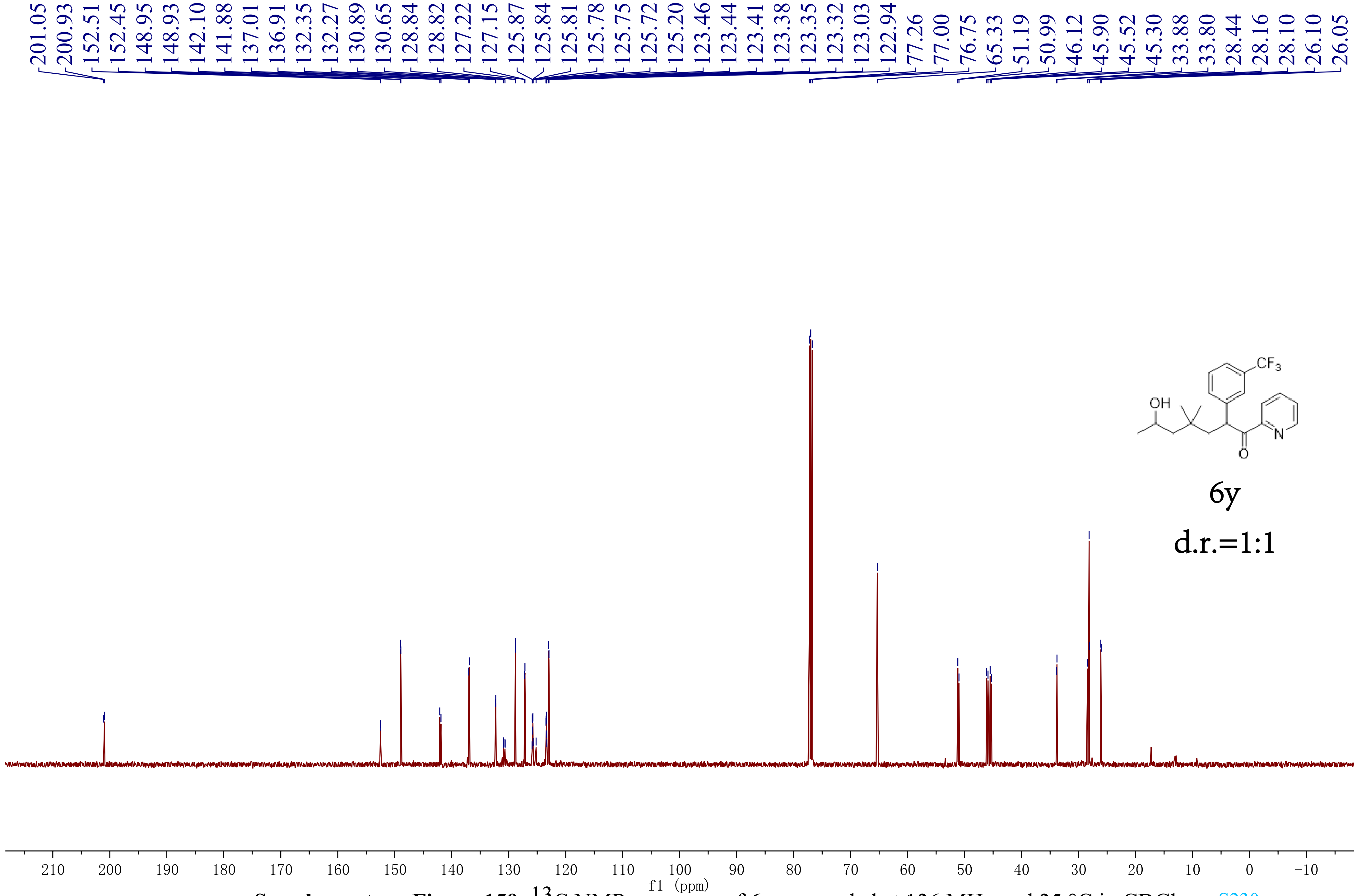

Supplementary Figure 159. ^{13}C NMR spectrum of **6y**, recorded at 126 MHz and 25 °C in $CDCl_3$

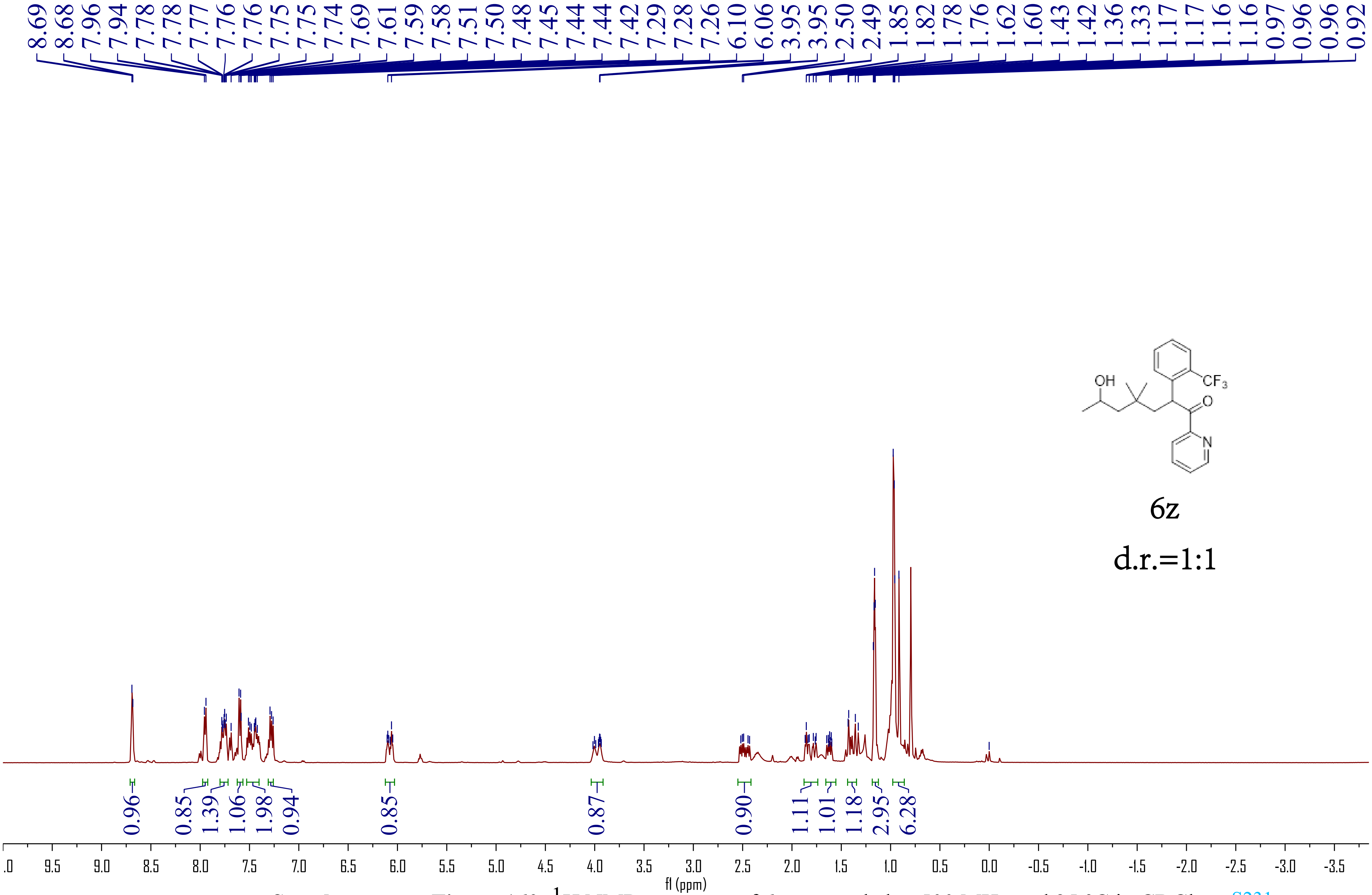

Supplementary Figure 160. ^{1}H NMR spectrum of **6z**, recorded at 500 MHz and 25 °C in $CDCl_3$

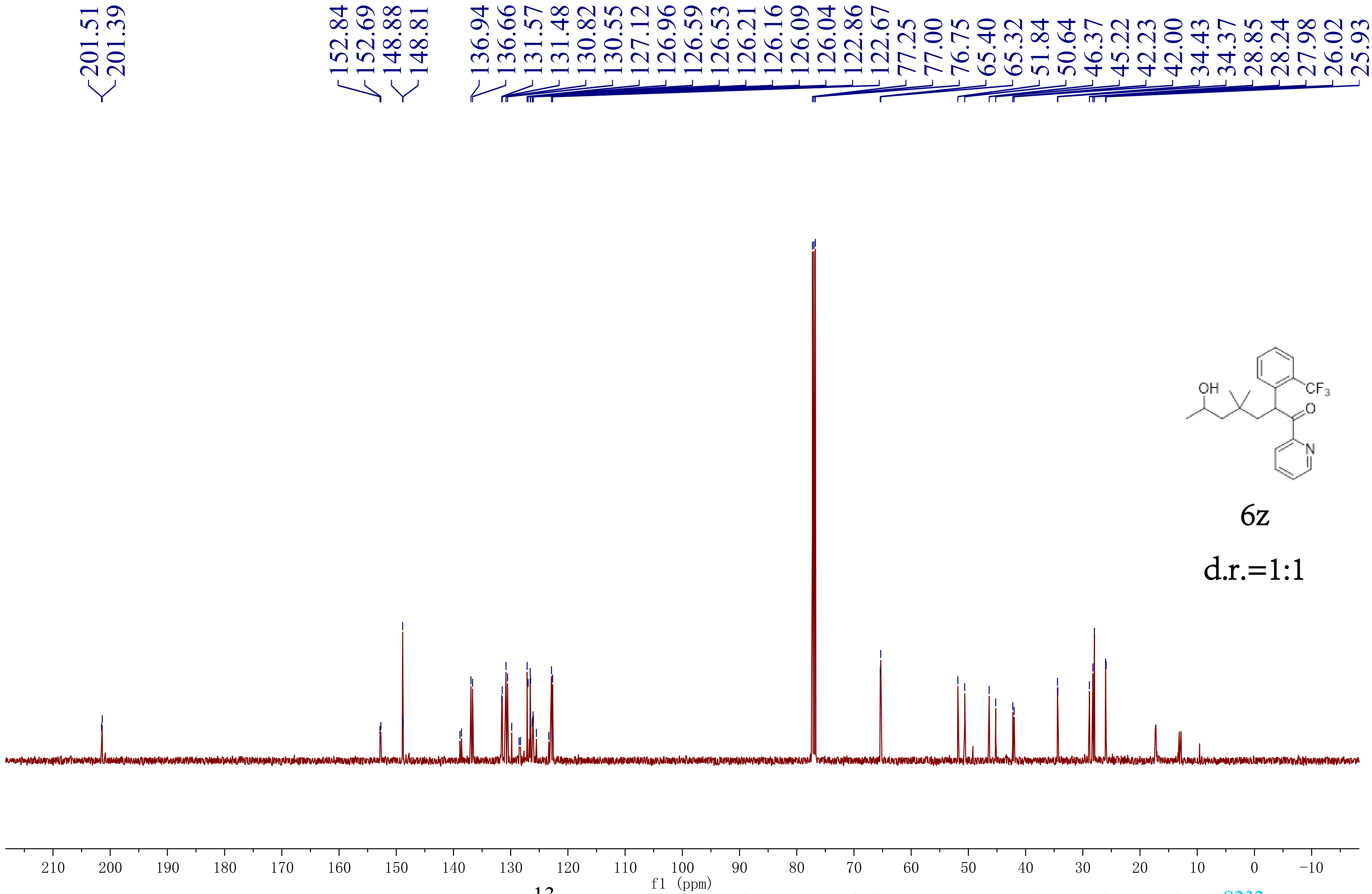

Supplementary Figure 161. ^{13}C NMR spectrum of **6z**, recorded at 126 MHz and 25 °C in $CDCl_3$

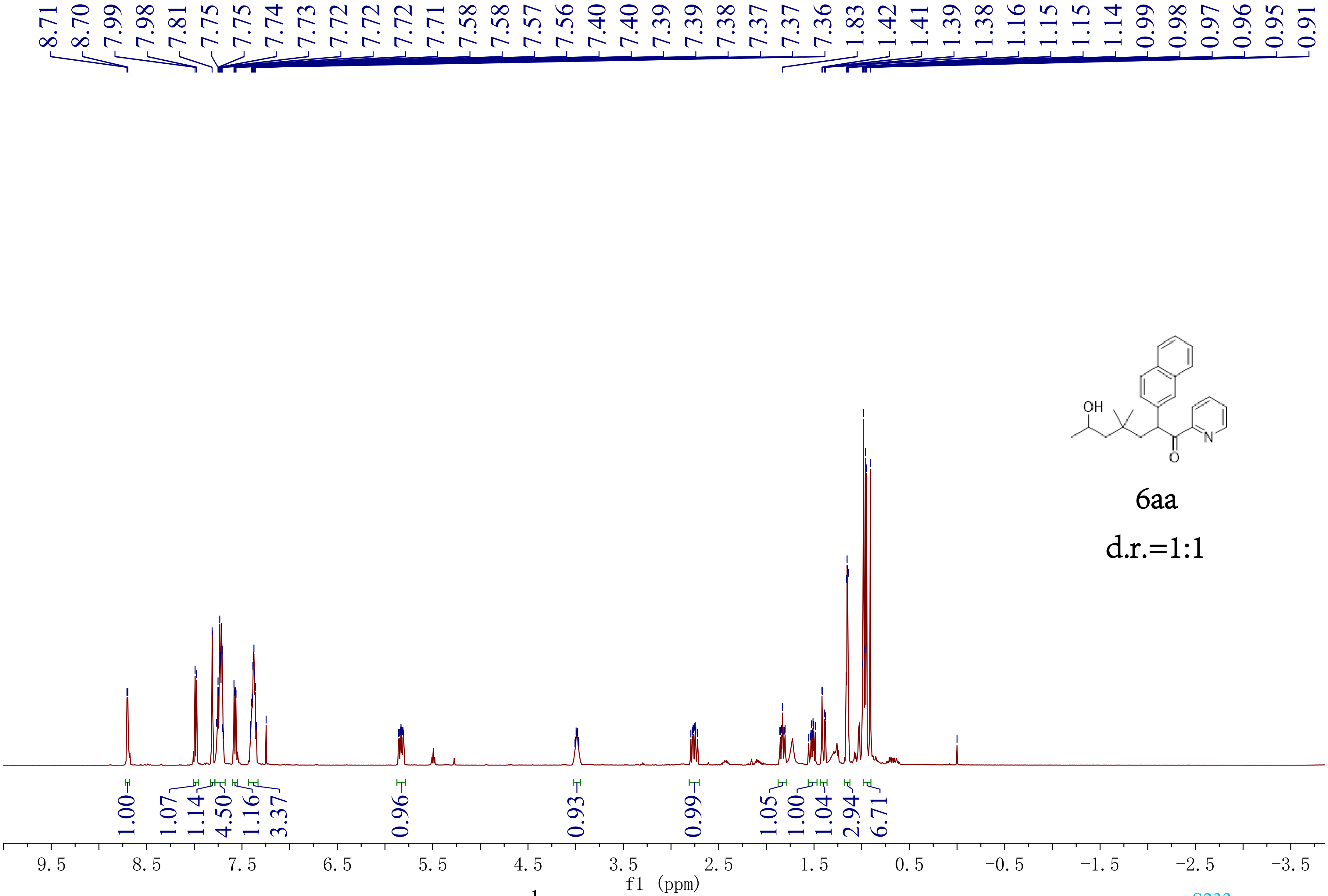

Supplementary Figure 162. ^{1}H NMR spectrum of **6aa**, recorded at 500 MHz and 25 °C in $CDCl_3$

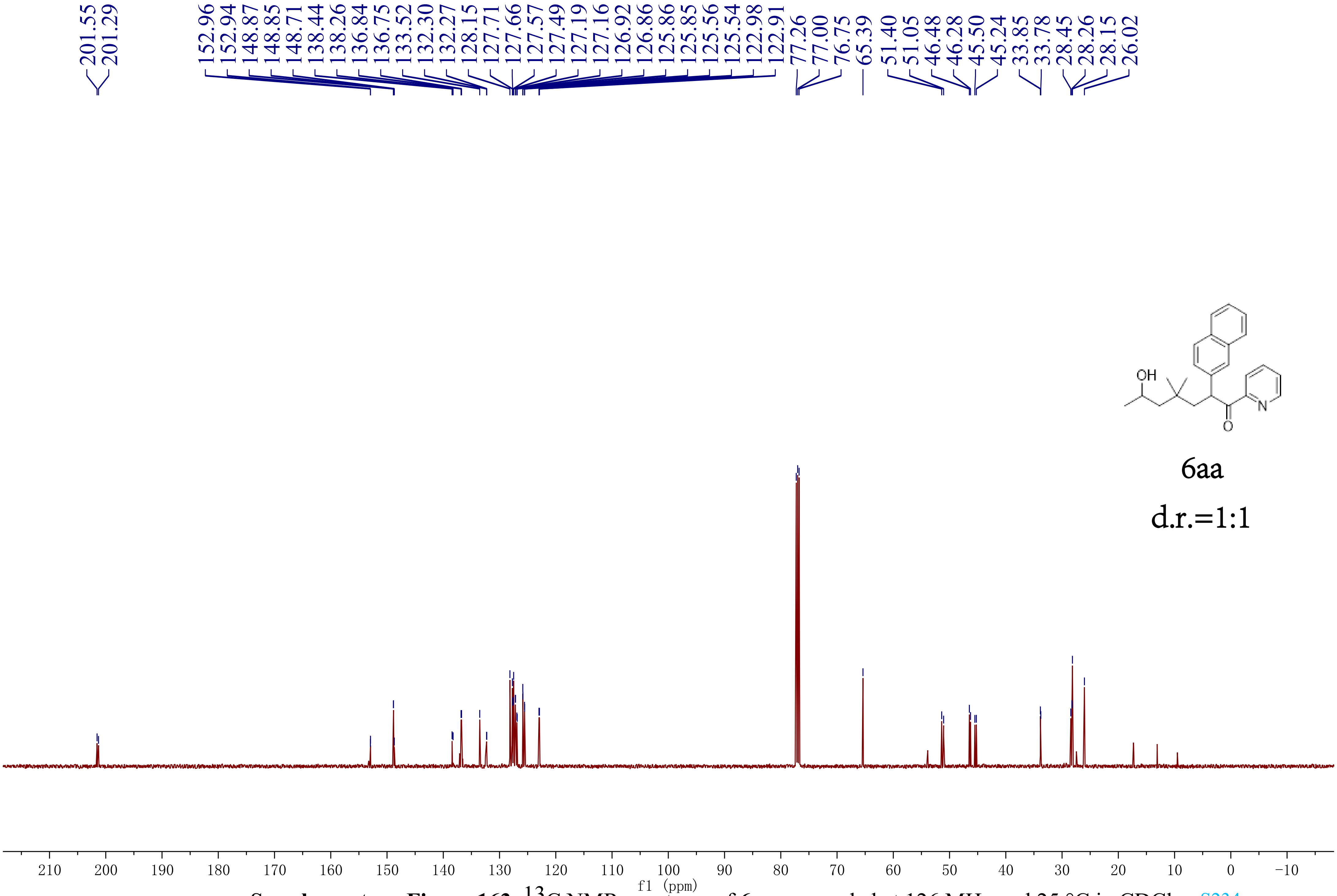

Supplementary Figure 163. ^{13}C NMR spectrum of **6aa**, recorded at 126 MHz and 25 °C in $CDCl_3$

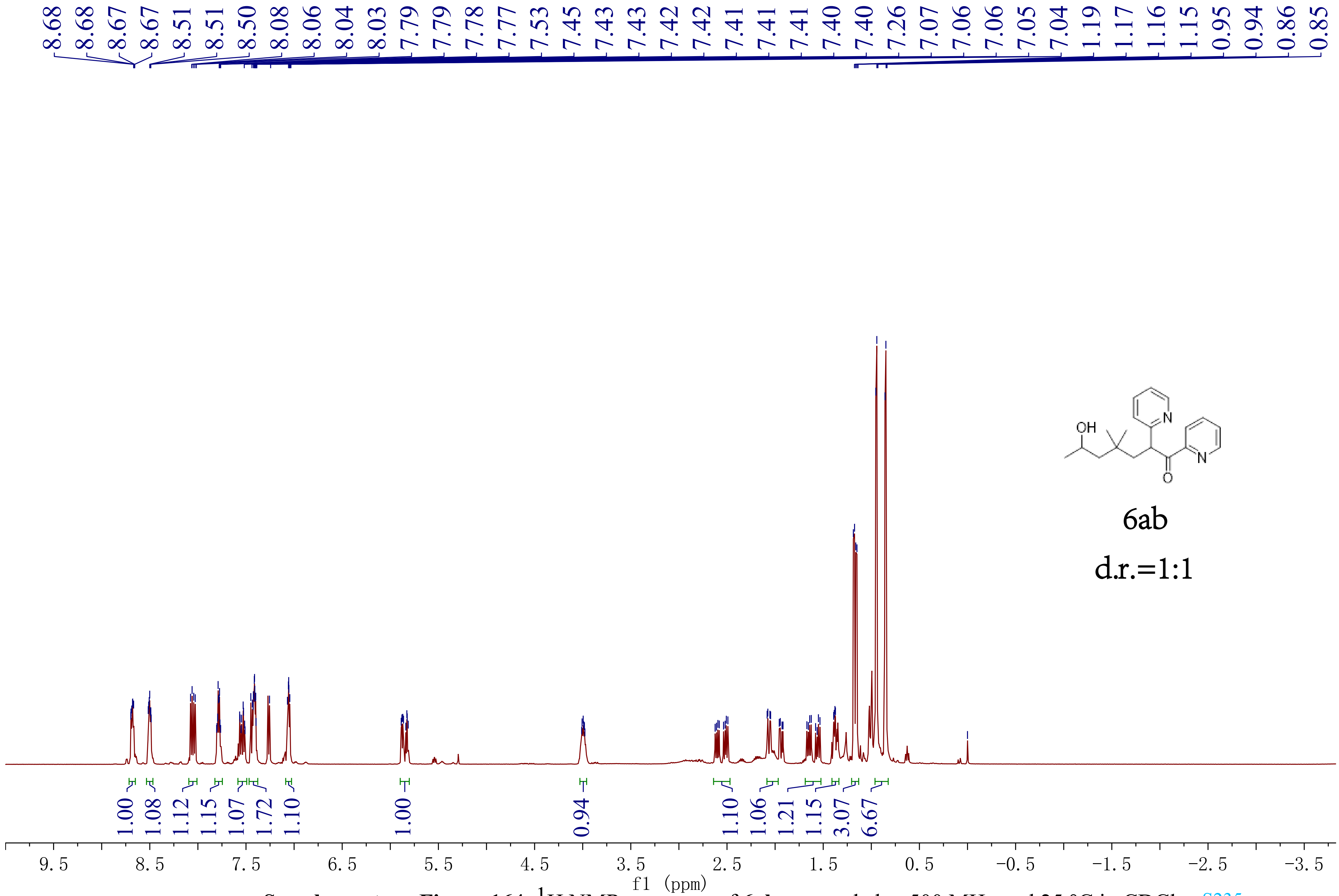

Supplementary Figure 164. ^{1}H NMR spectrum of **6ab**, recorded at 500 MHz and 25 °C in $CDCl_3$

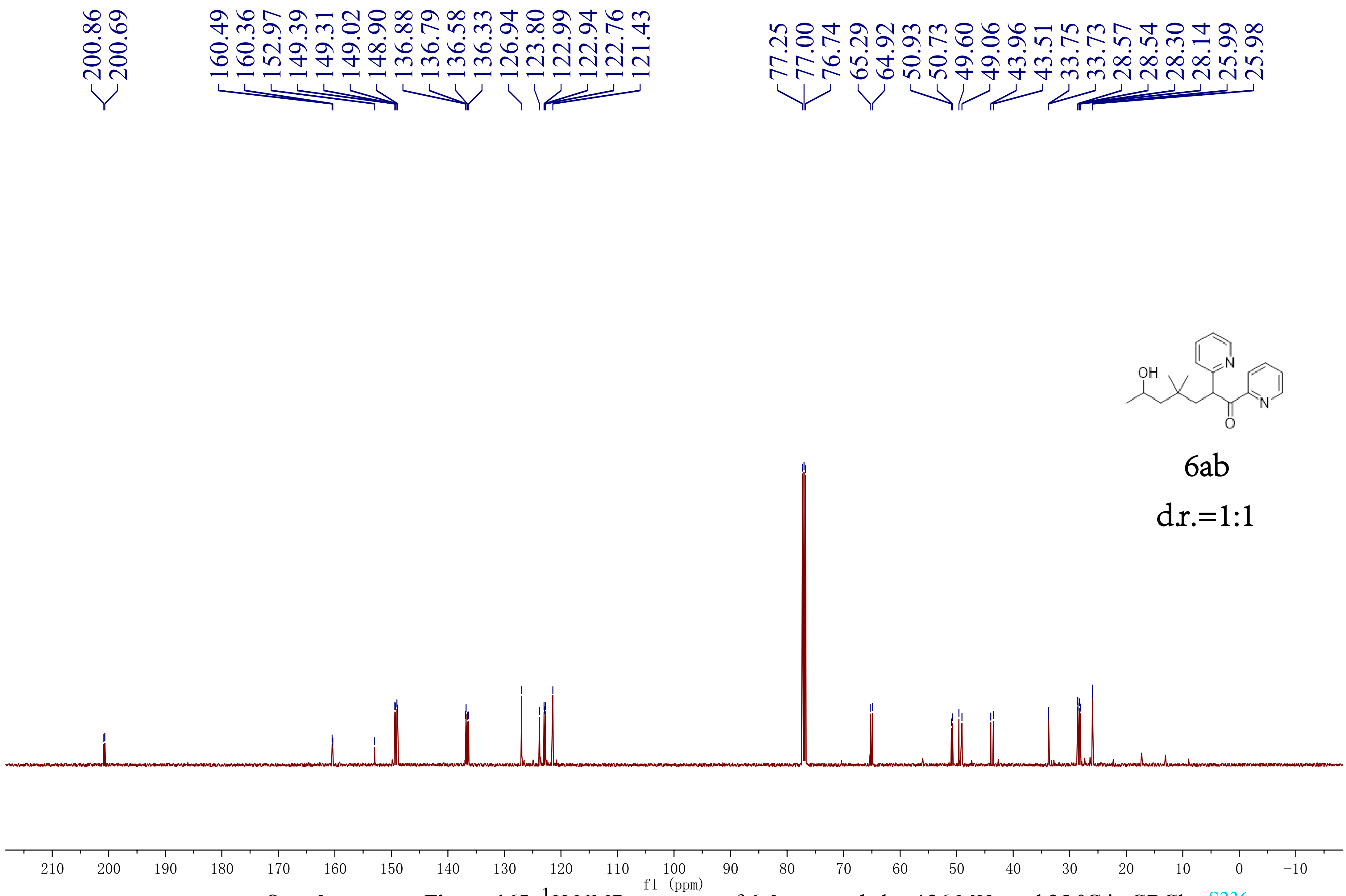

Supplementary Figure 165. ^{1}H NMR spectrum of **6ab**, recorded at 126 MHz and 25 °C in $CDCl_3$

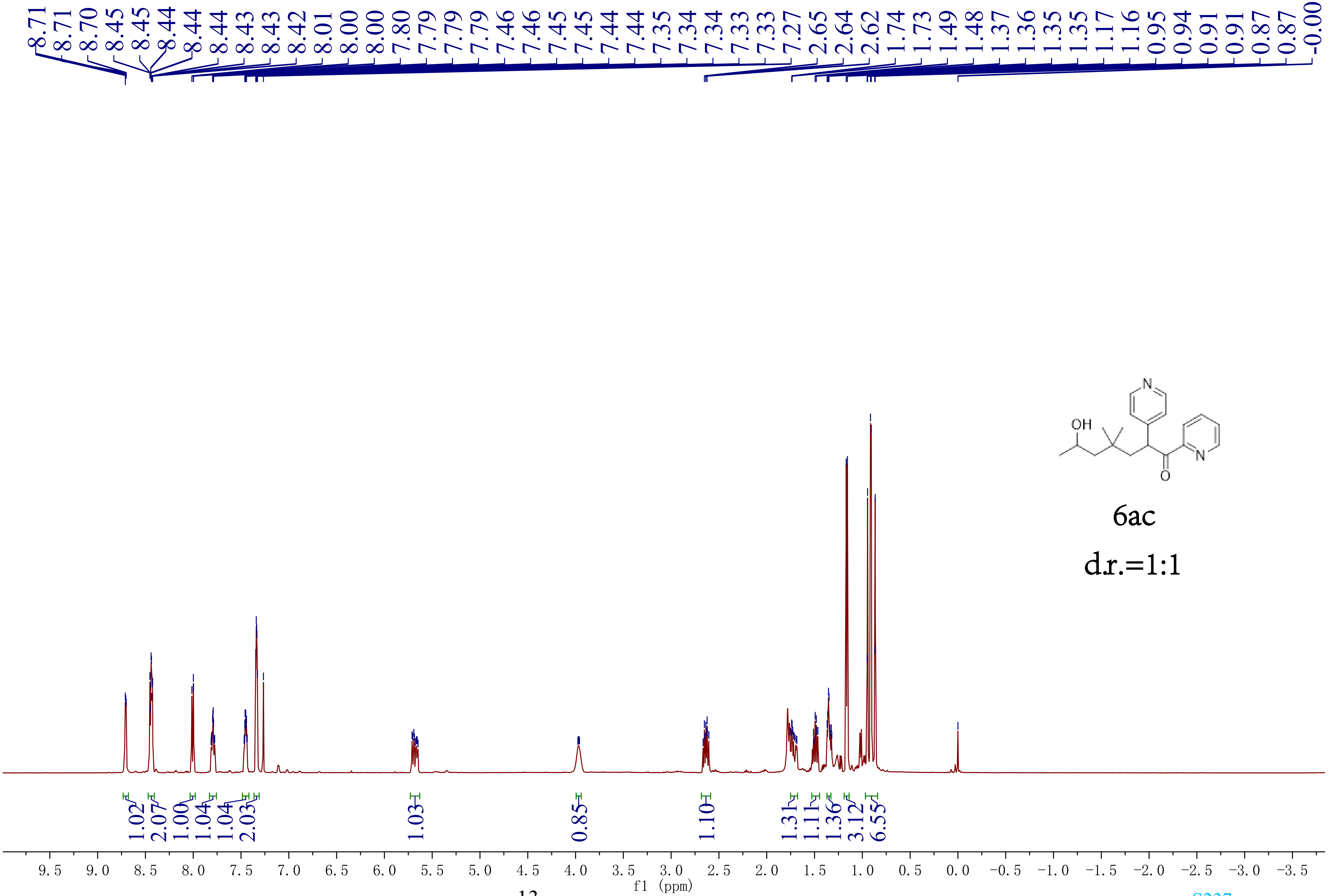

Supplementary Figure 166. ^{13}C NMR spectrum of **6ac**, recorded at 500 MHz and 25 °C in $CDCl_3$

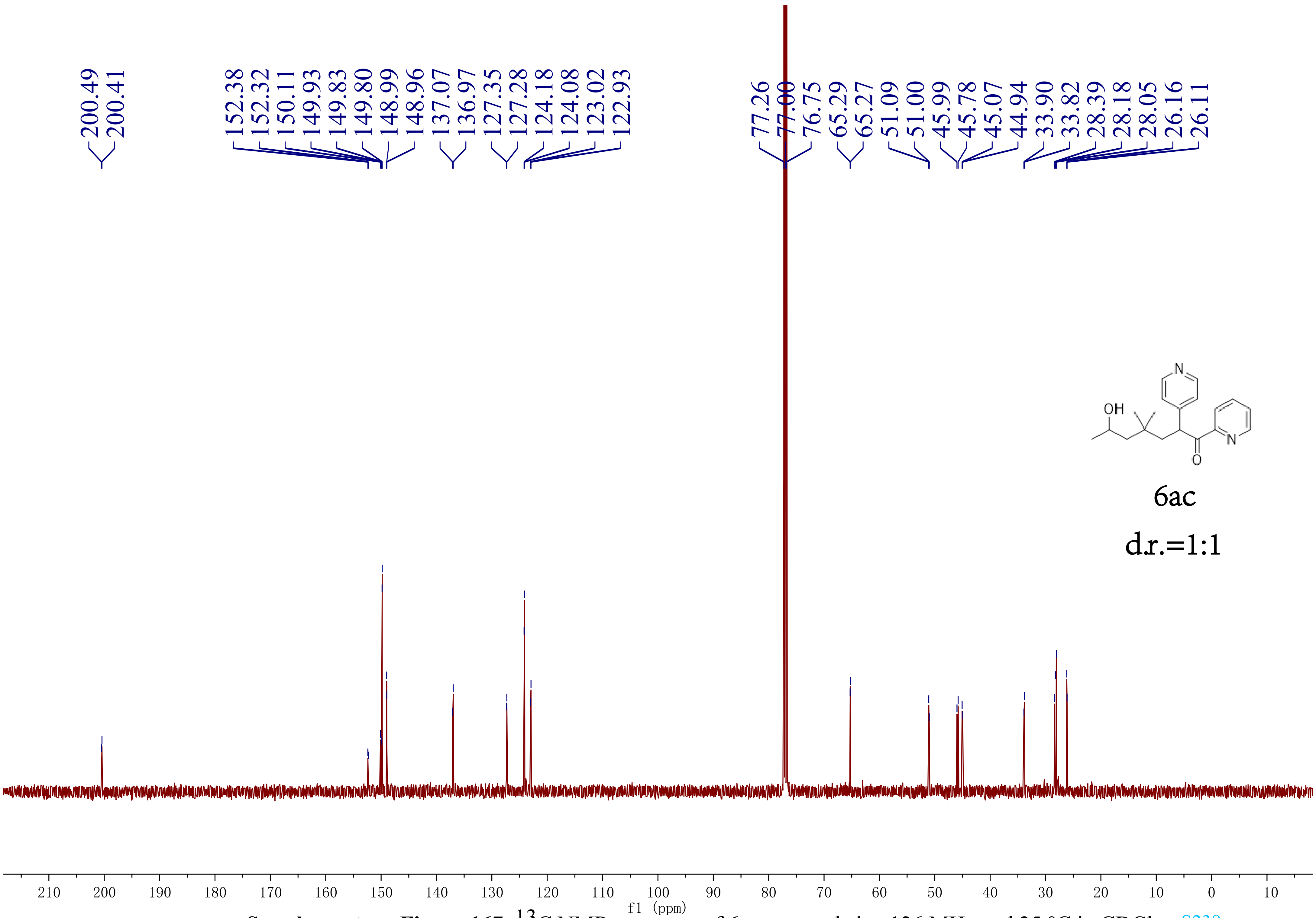

6ac

d.r.=1:1

Supplementary Figure 167. ^{13}C NMR spectrum of **6ac**, recorded at 126 MHz and 25 °C in $CDCl_3$

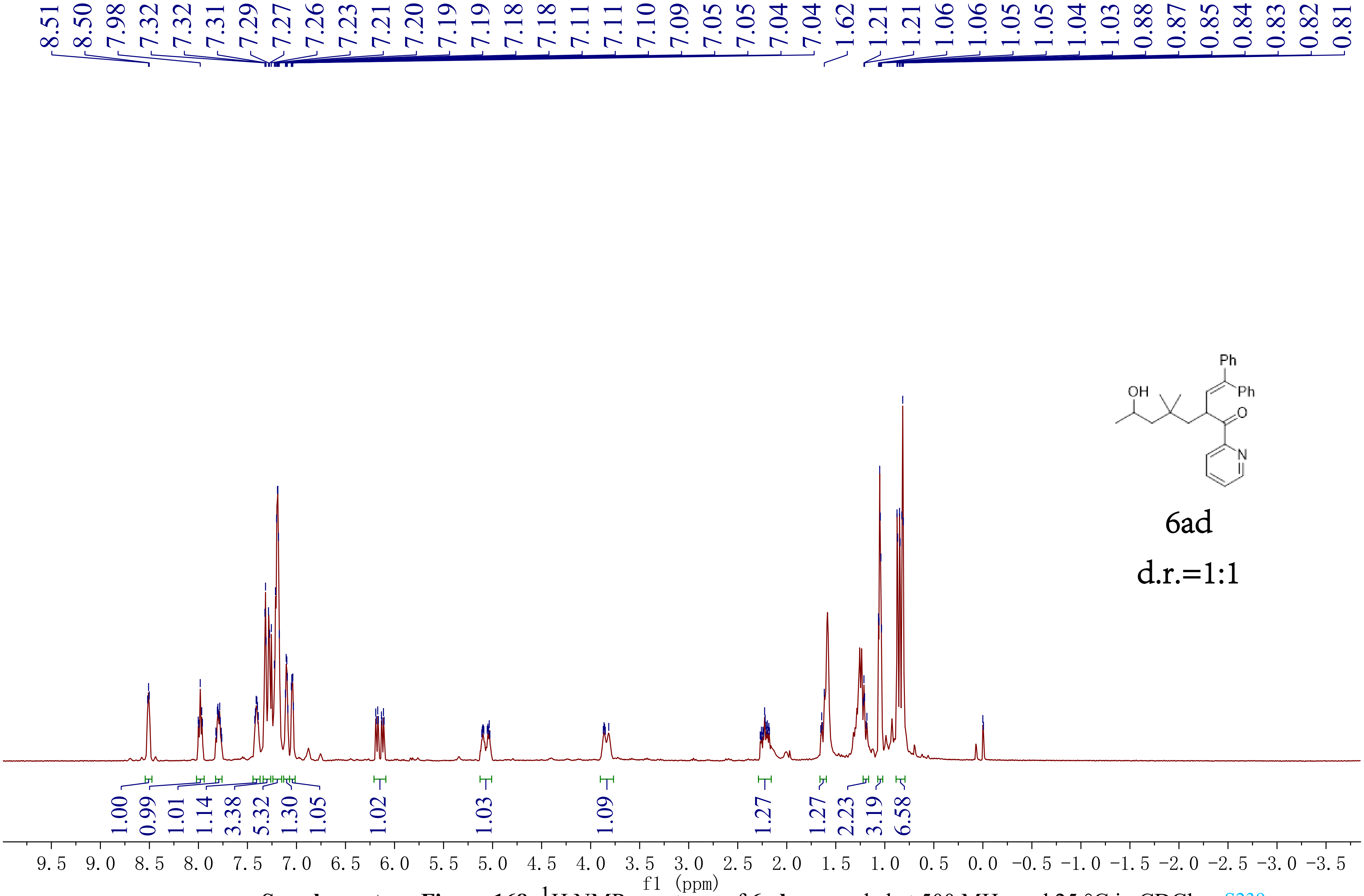

Supplementary Figure 168. ^{1}H NMR spectrum of **6ad**, recorded at 500 MHz and 25 °C in $CDCl_3$

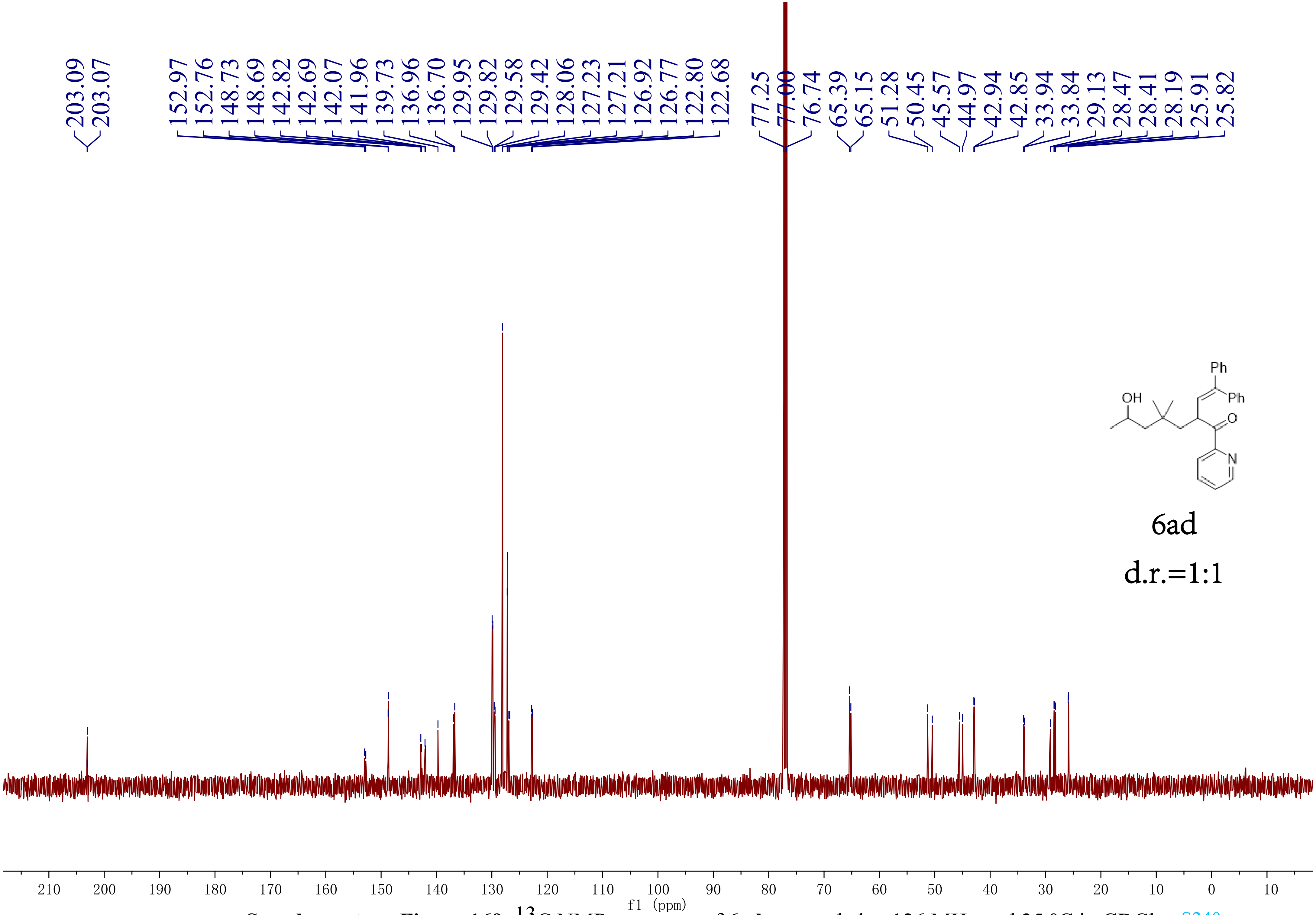

Supplementary Figure 169. ^{13}C NMR spectrum of **6ad**, recorded at 126 MHz and 25 °C in $CDCl_3$

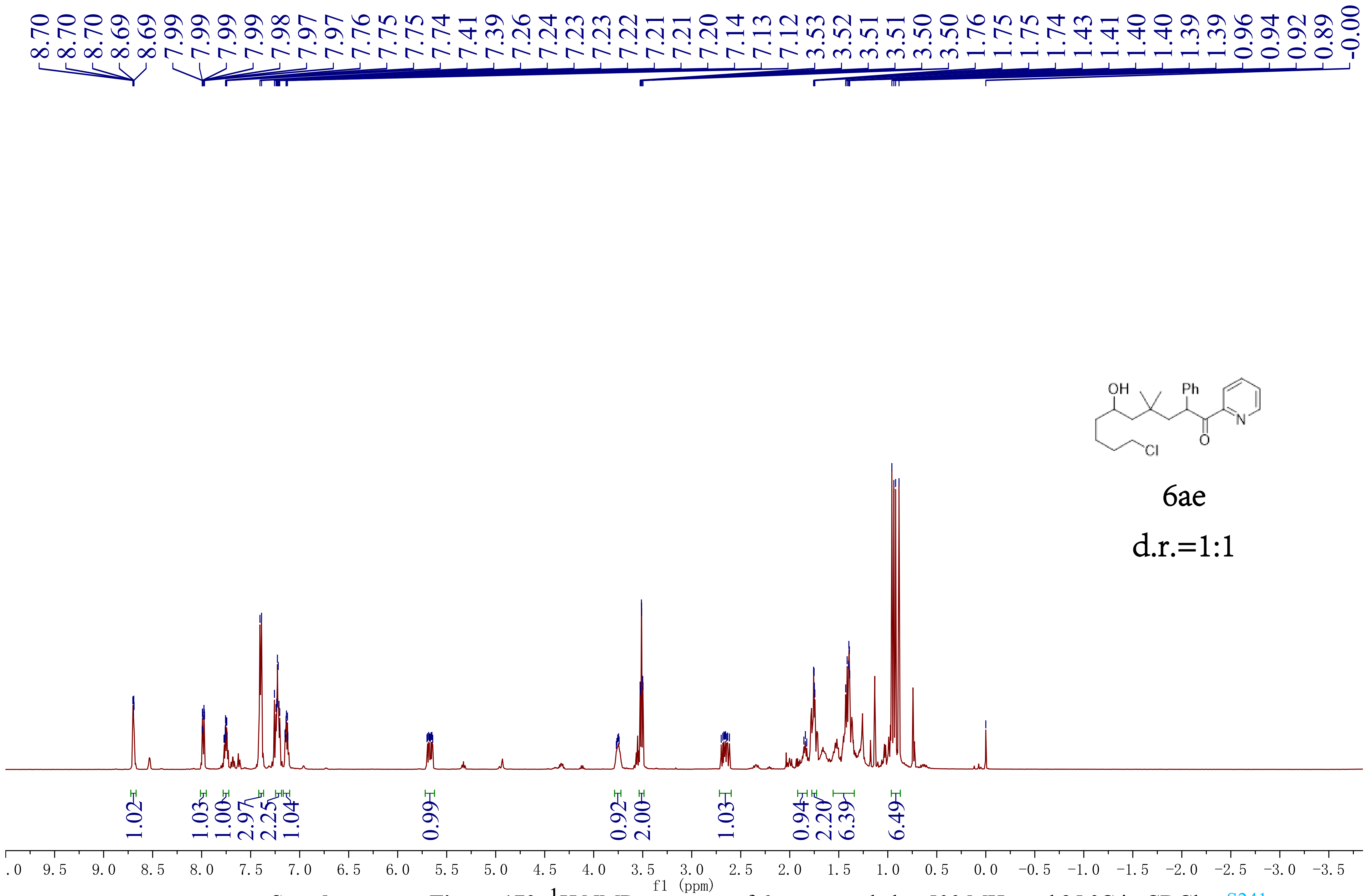

Supplementary Figure 170. ^{1}H NMR spectrum of **6ae**, recorded at 500 MHz and 25 °C in $CDCl_3$

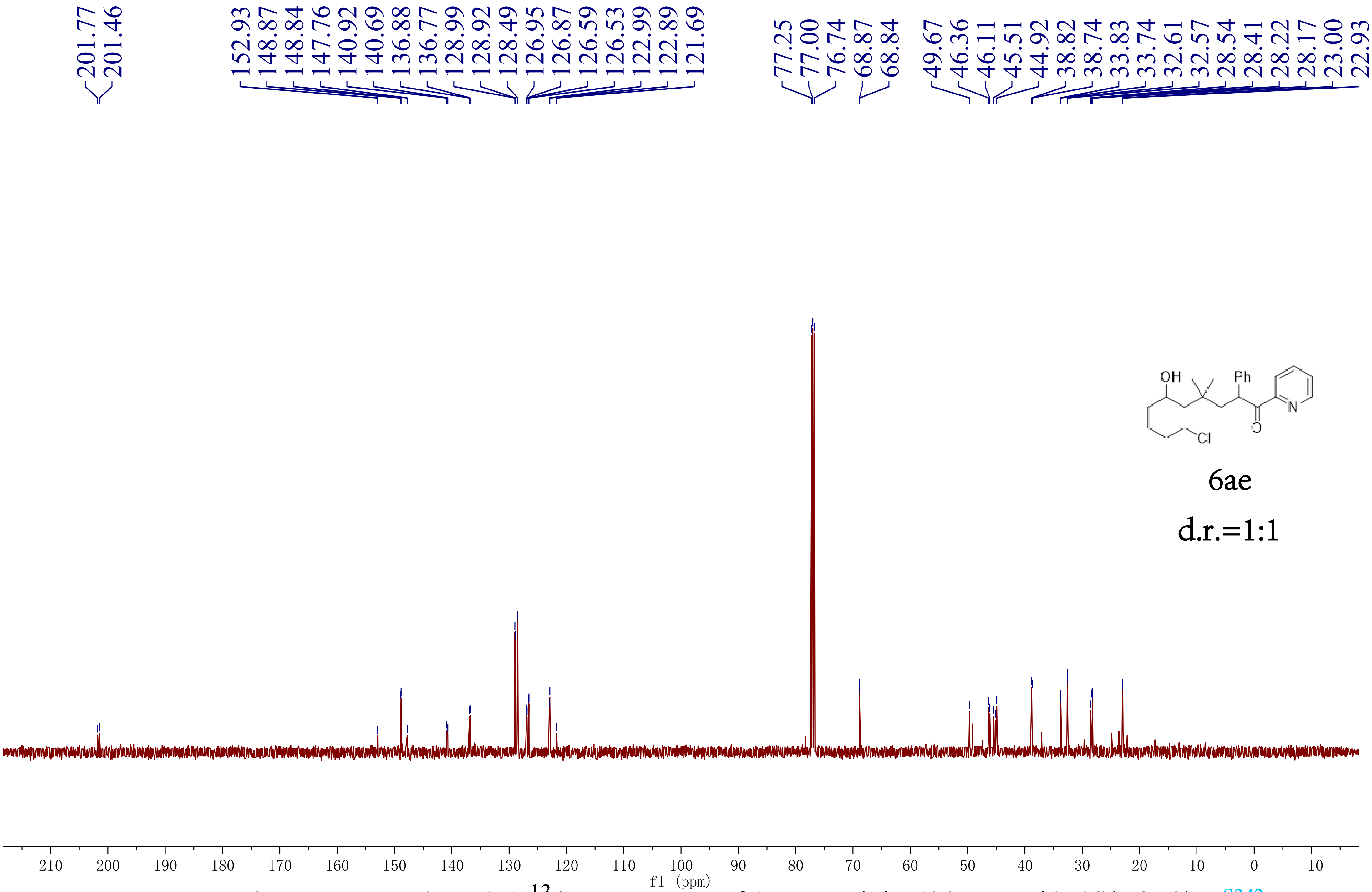

Supplementary Figure 171. ^{13}C NMR spectrum of **6ae**, recorded at 126 MHz and 25 °C in $CDCl_3$

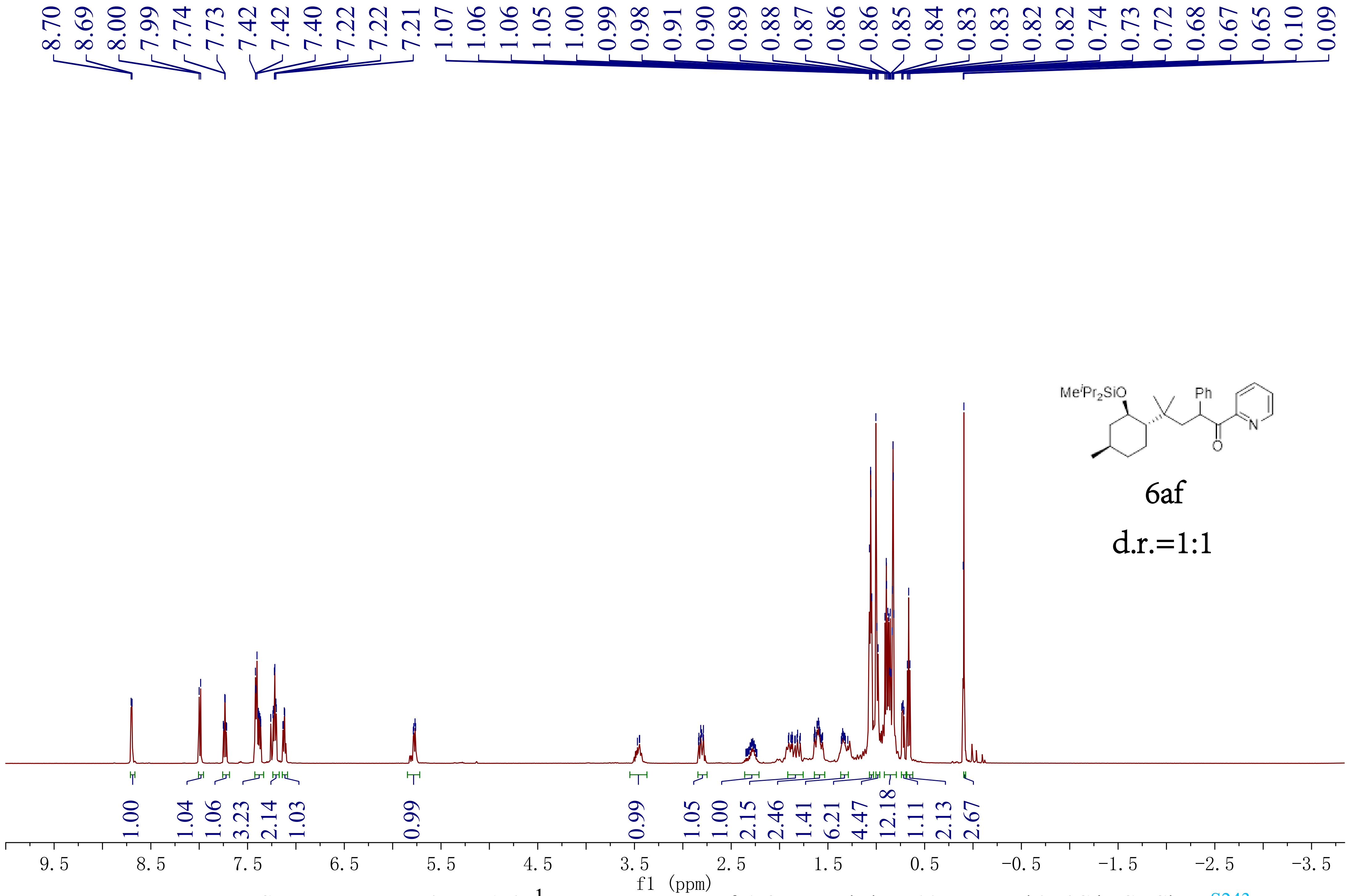

Supplementary Figure 172. ^{1}H NMR spectrum of **6af**, recorded at 500 MHz and 25 °C in $CDCl_3$

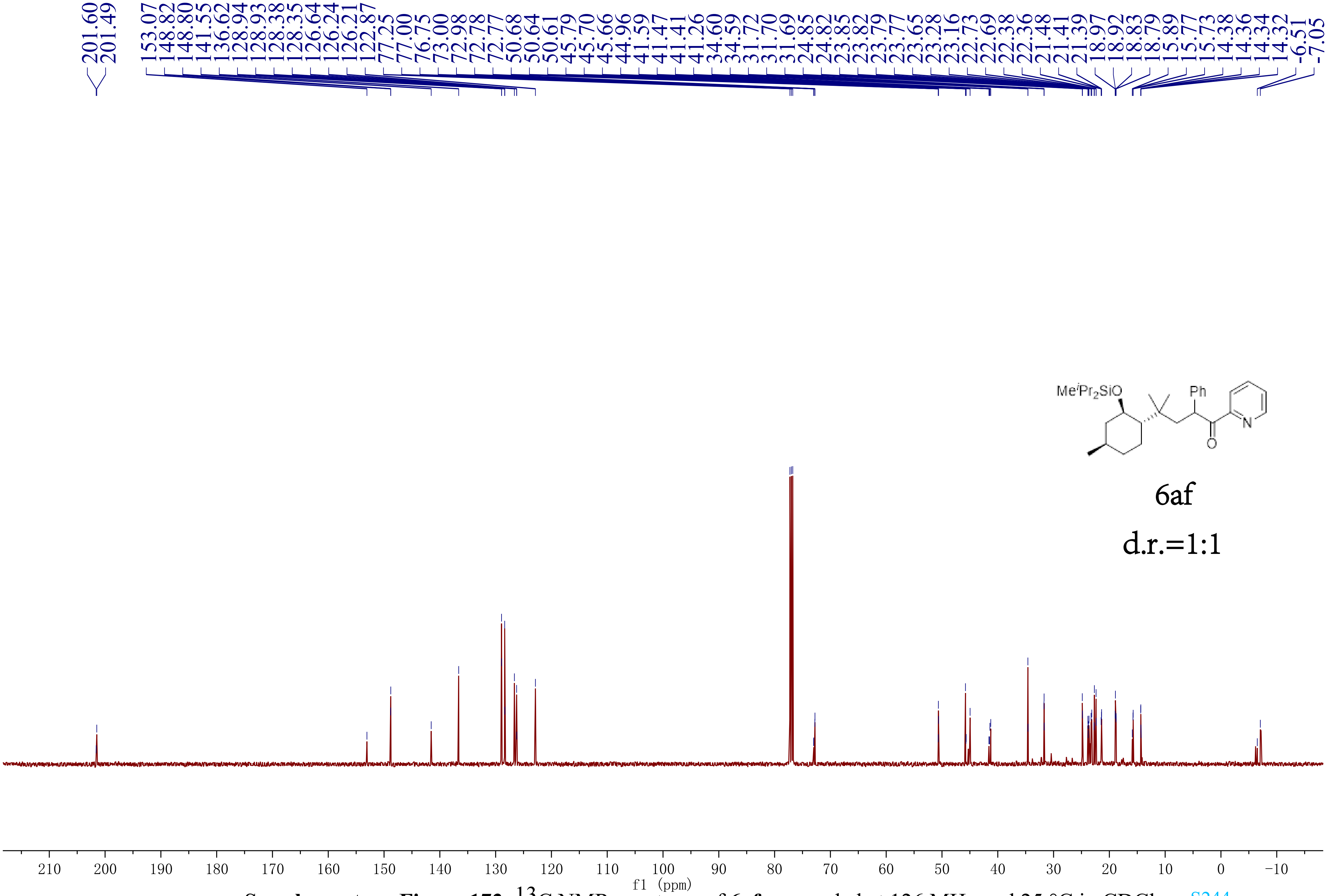

Supplementary Figure 173. ^{13}C NMR spectrum of **6af**, recorded at 126 MHz and 25 °C in $CDCl_3$

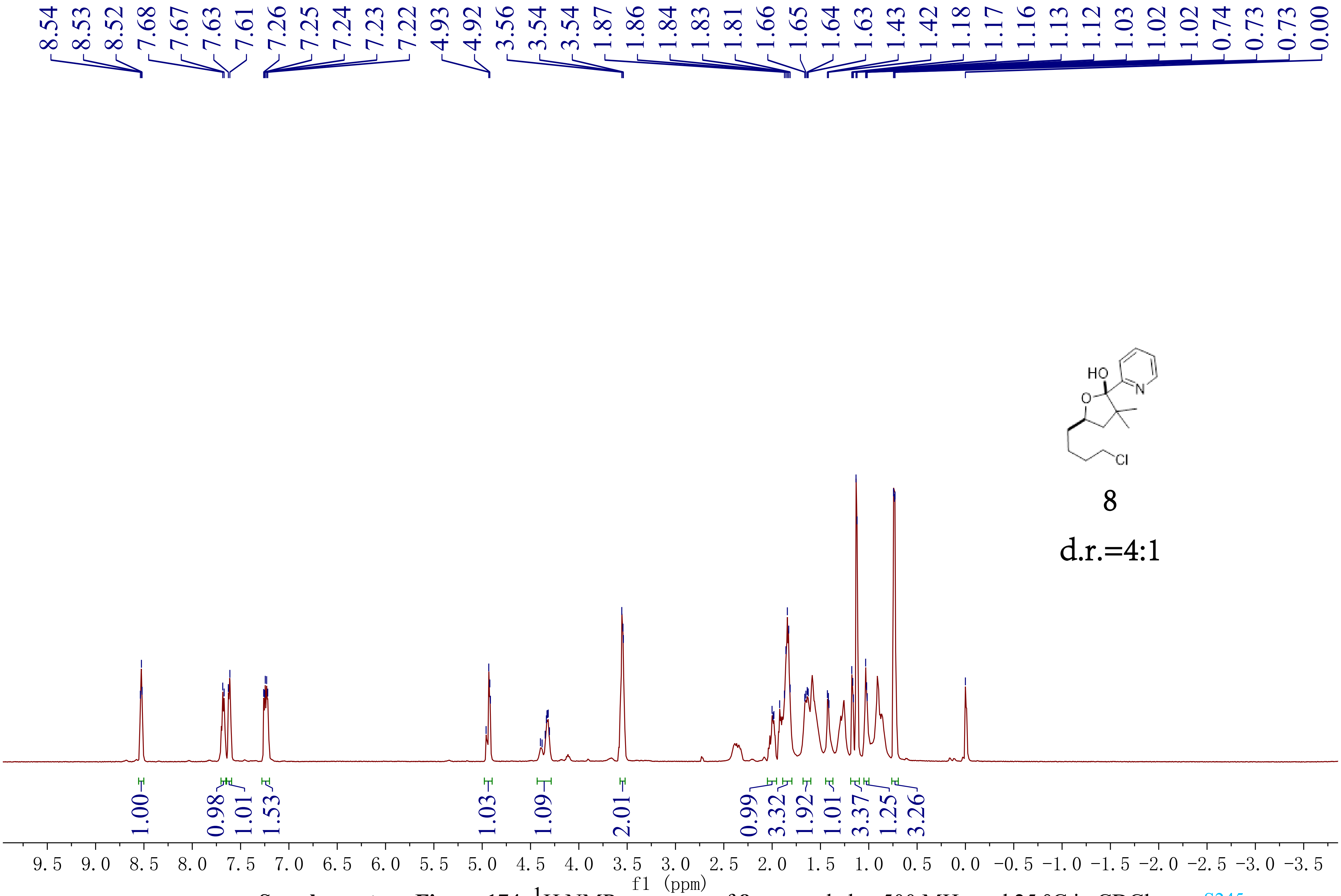

Supplementary Figure 174. ^{1}H NMR spectrum of **8**, recorded at 500 MHz and 25 °C in $CDCl_3$

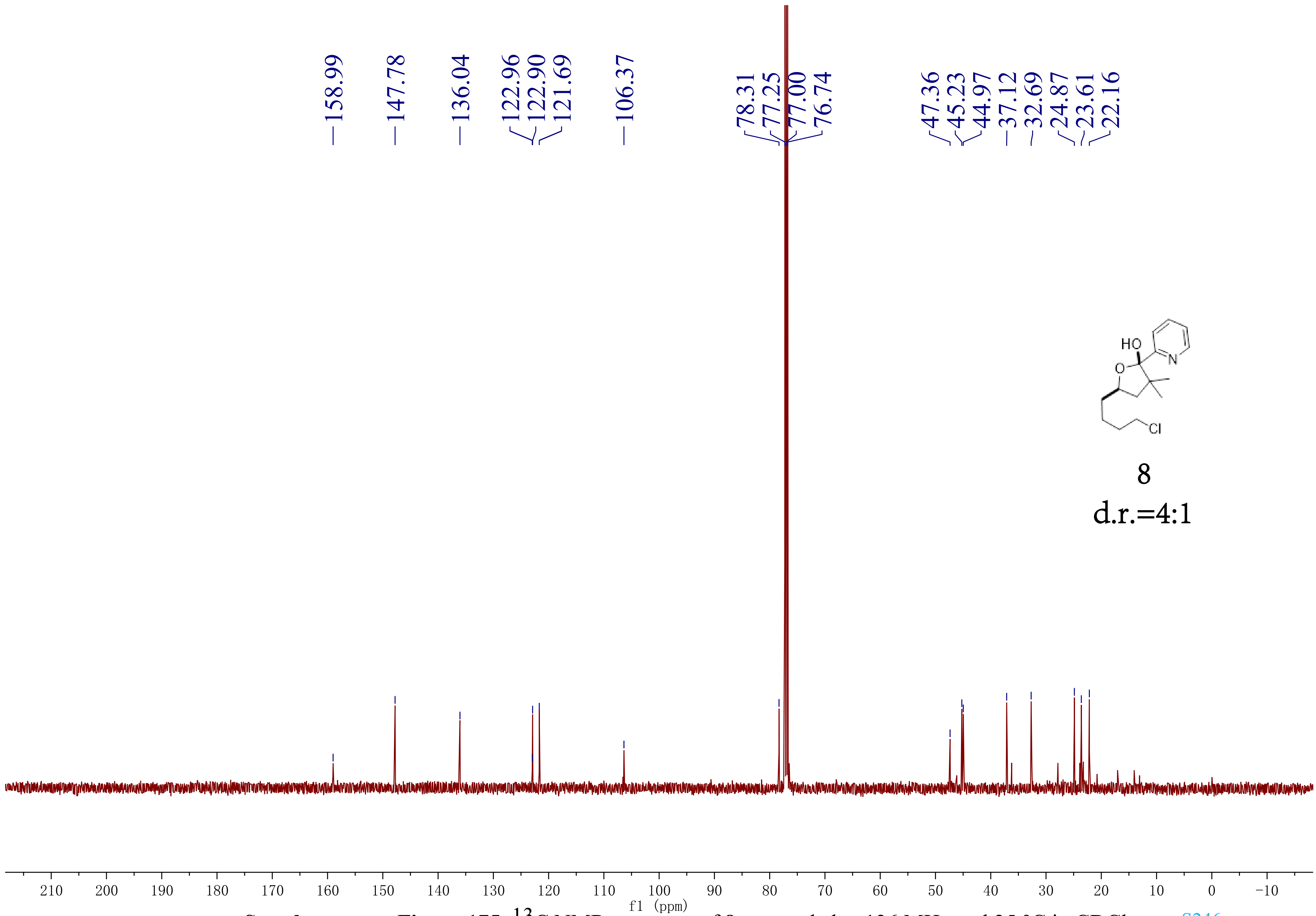

Supplementary Figure 175. ^{13}C NMR spectrum of **8**, recorded at 126 MHz and 25 °C in $CDCl_3$

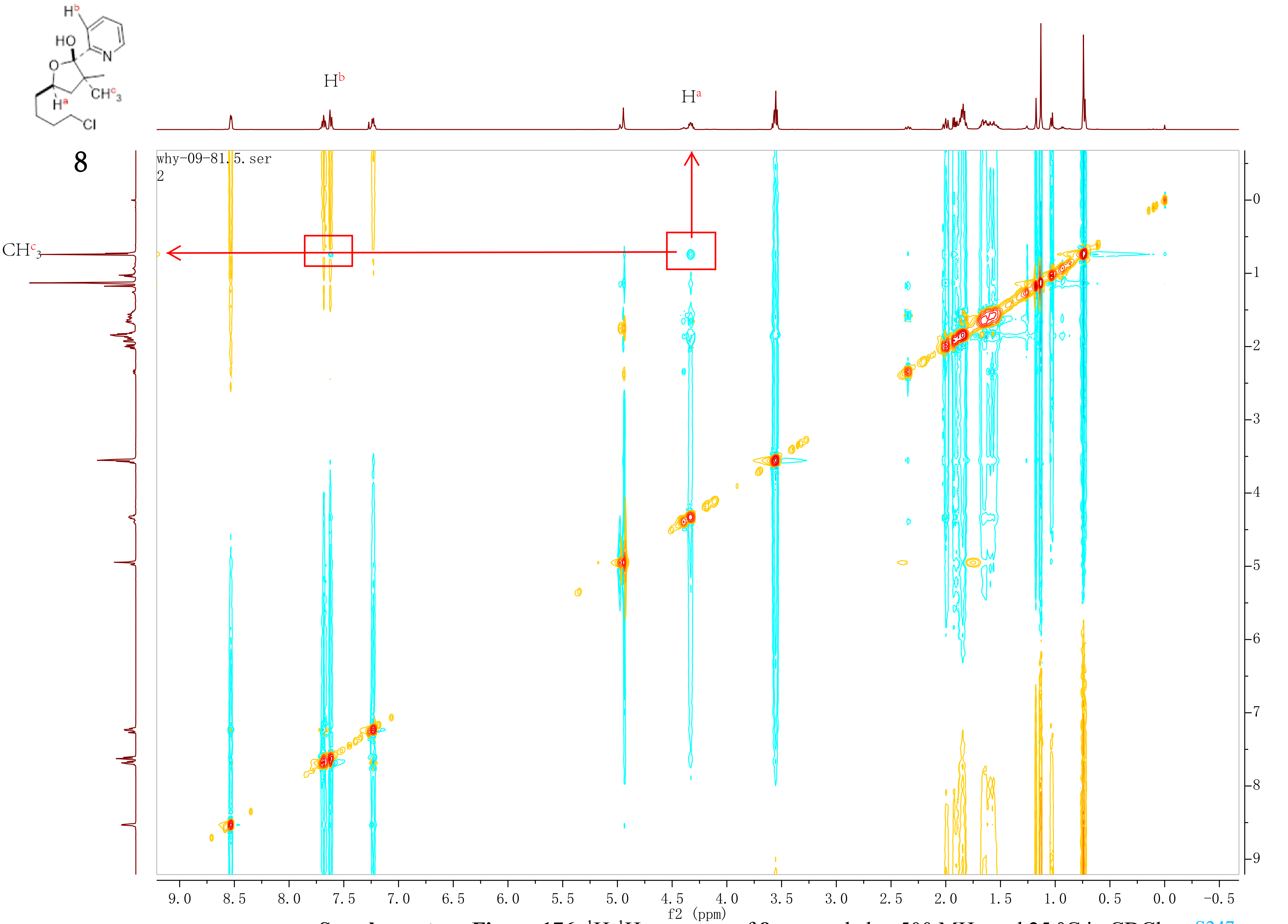

Supplementary Figure 176. ^1H-^1H spectrum of **8**, recorded at 500 MHz and 25 °C in $CDCl_3$

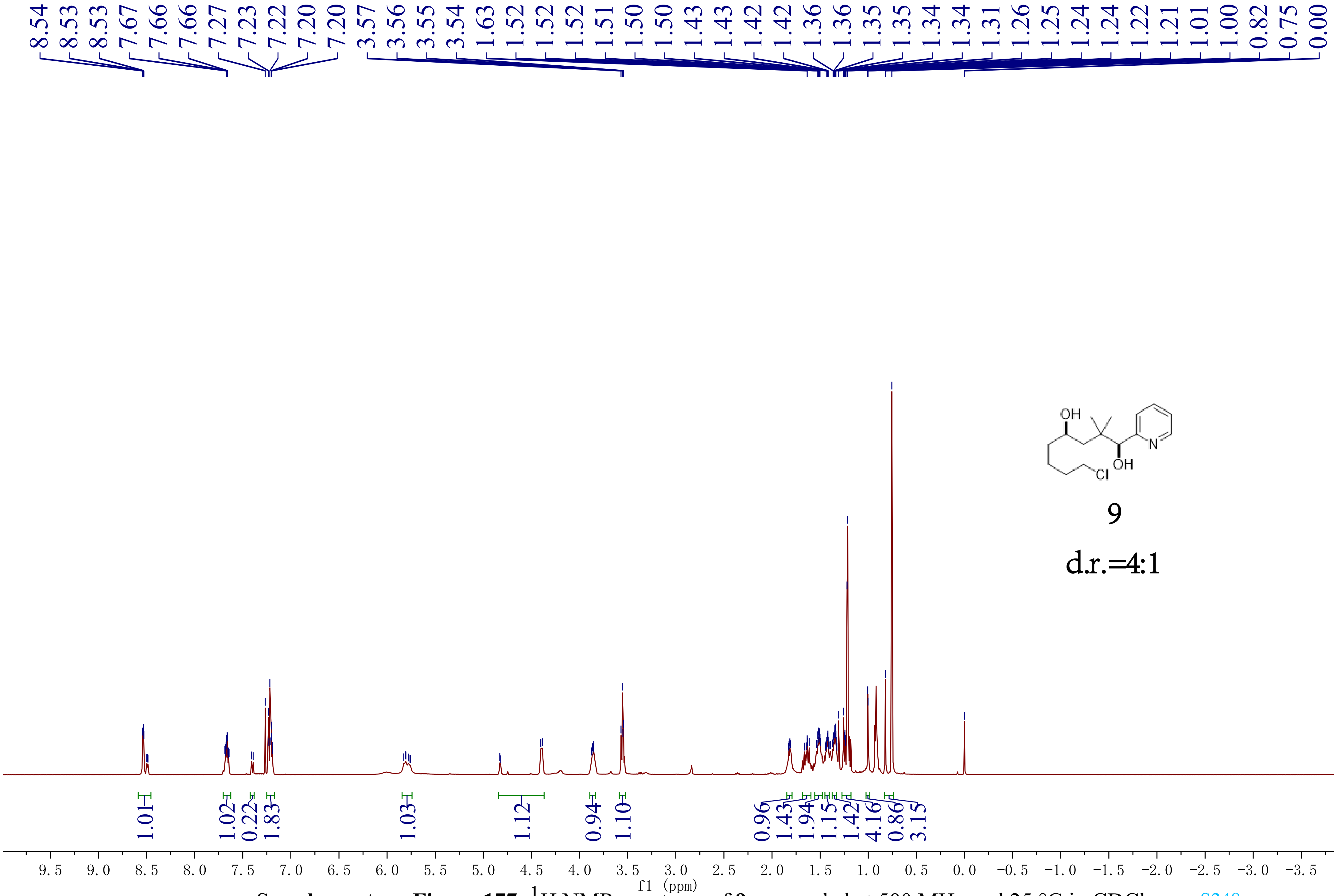

9

d.r.=4:1

Supplementary Figure 177. ^{1}H NMR spectrum of **9**, recorded at 500 MHz and 25 °C in $CDCl_3$

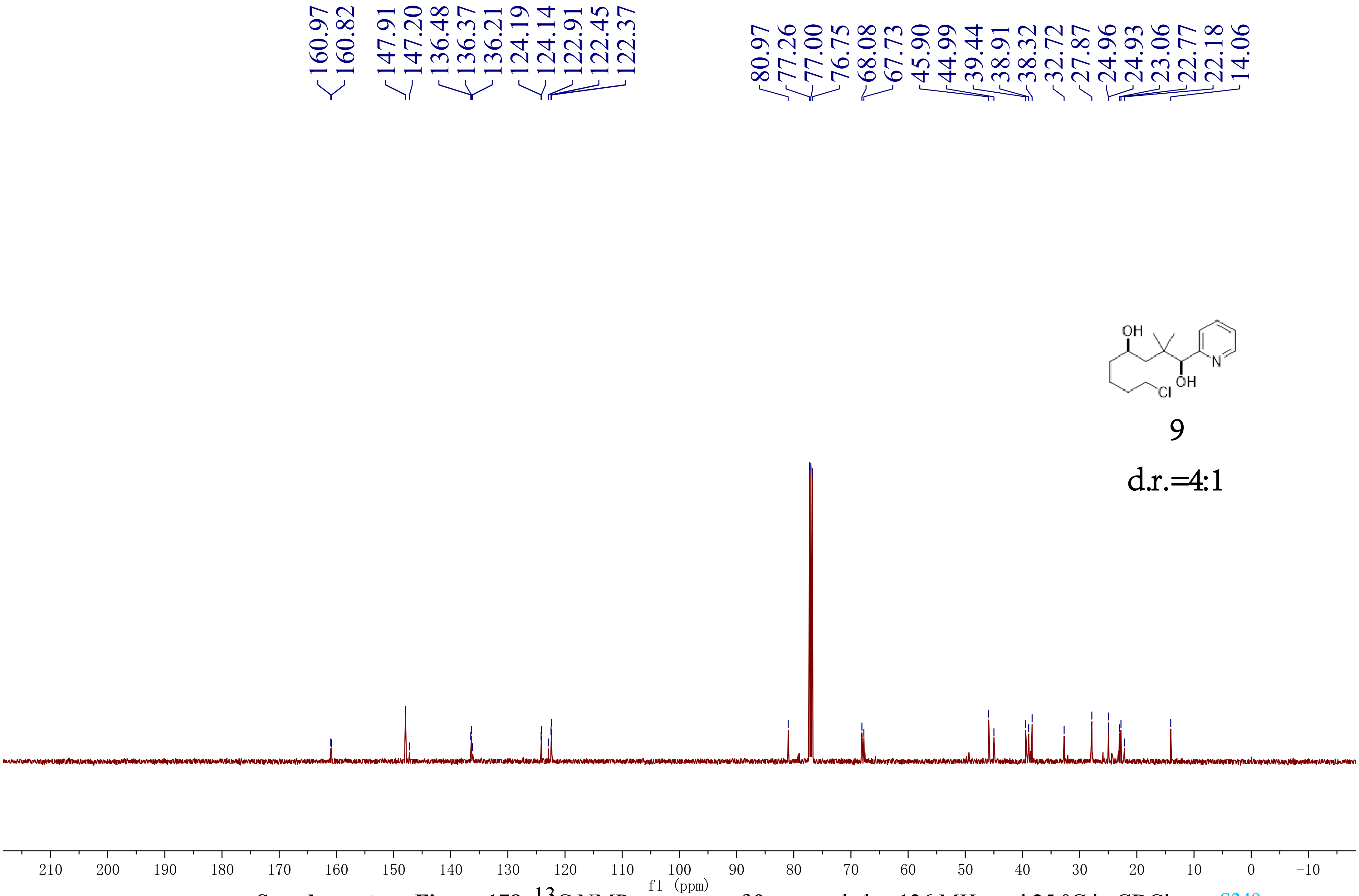

Supplementary Figure 178. ^{13}C NMR spectrum of **9**, recorded at 126 MHz and 25 °C in $CDCl_3$

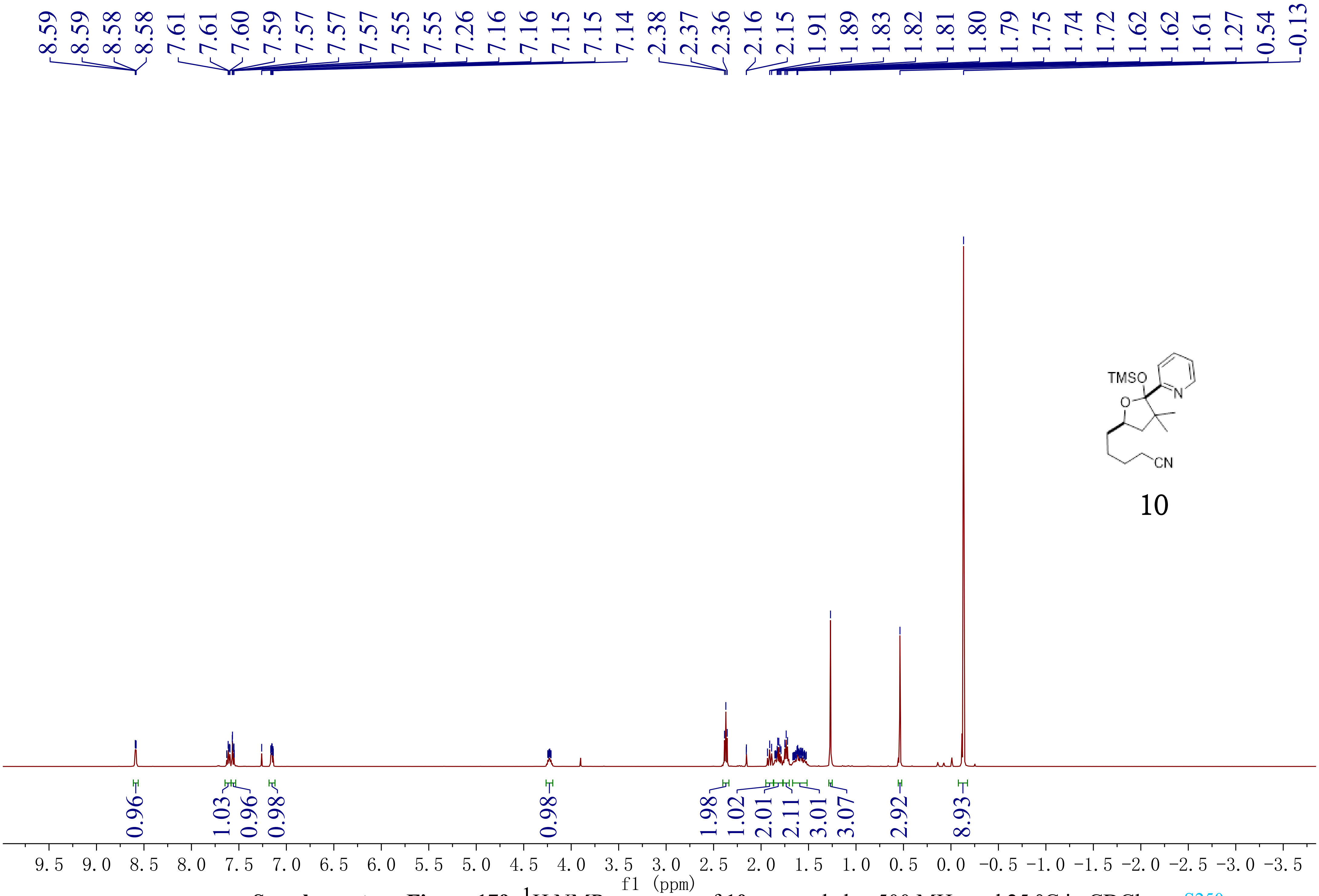

Supplementary Figure 179. ^{1}H NMR spectrum of **10**, recorded at 500 MHz and 25 °C in $CDCl_3$

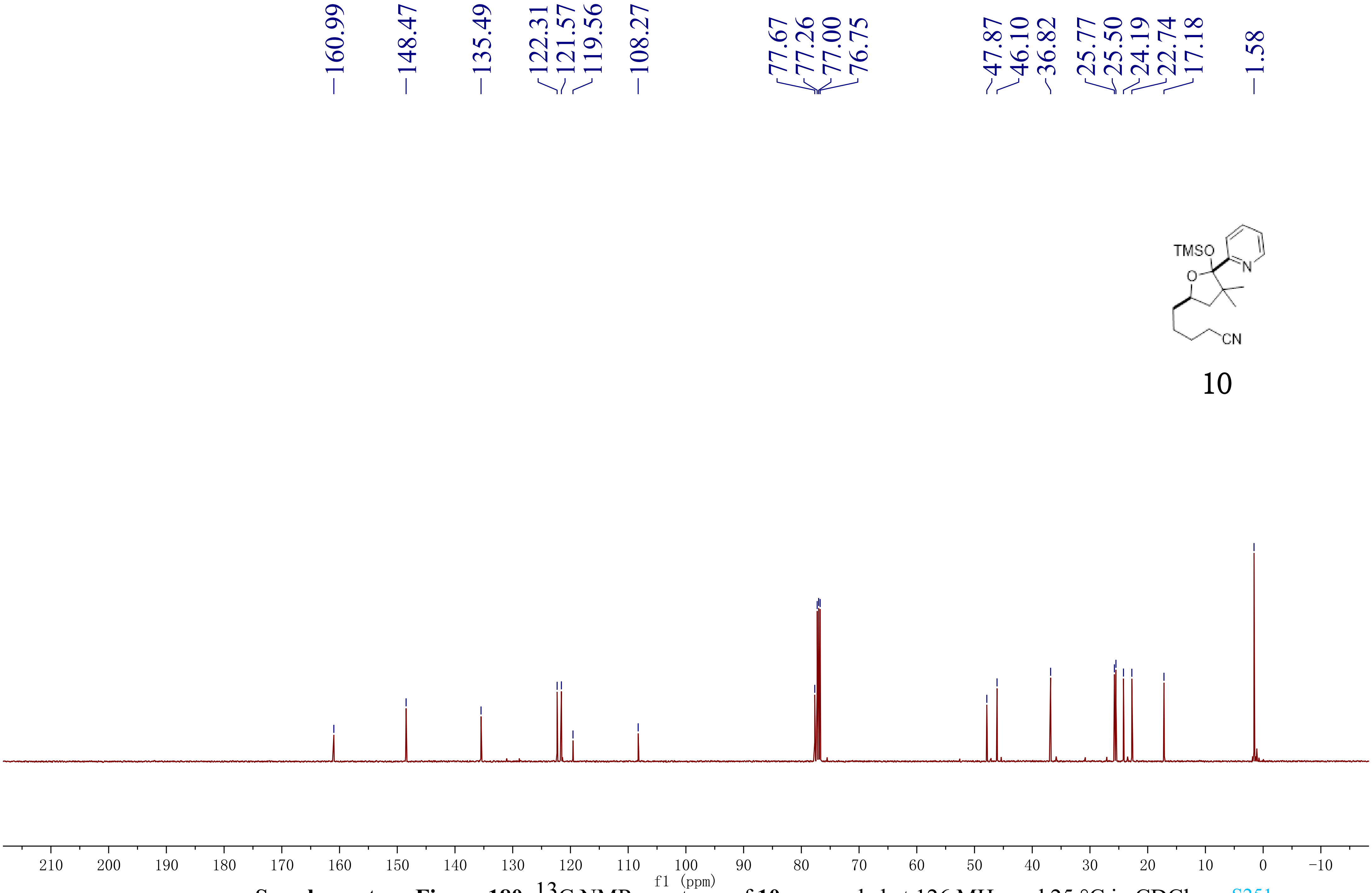

10

Supplementary Figure 180. ^{13}C NMR spectrum of **10**, recorded at 126 MHz and 25 °C in $CDCl_3$

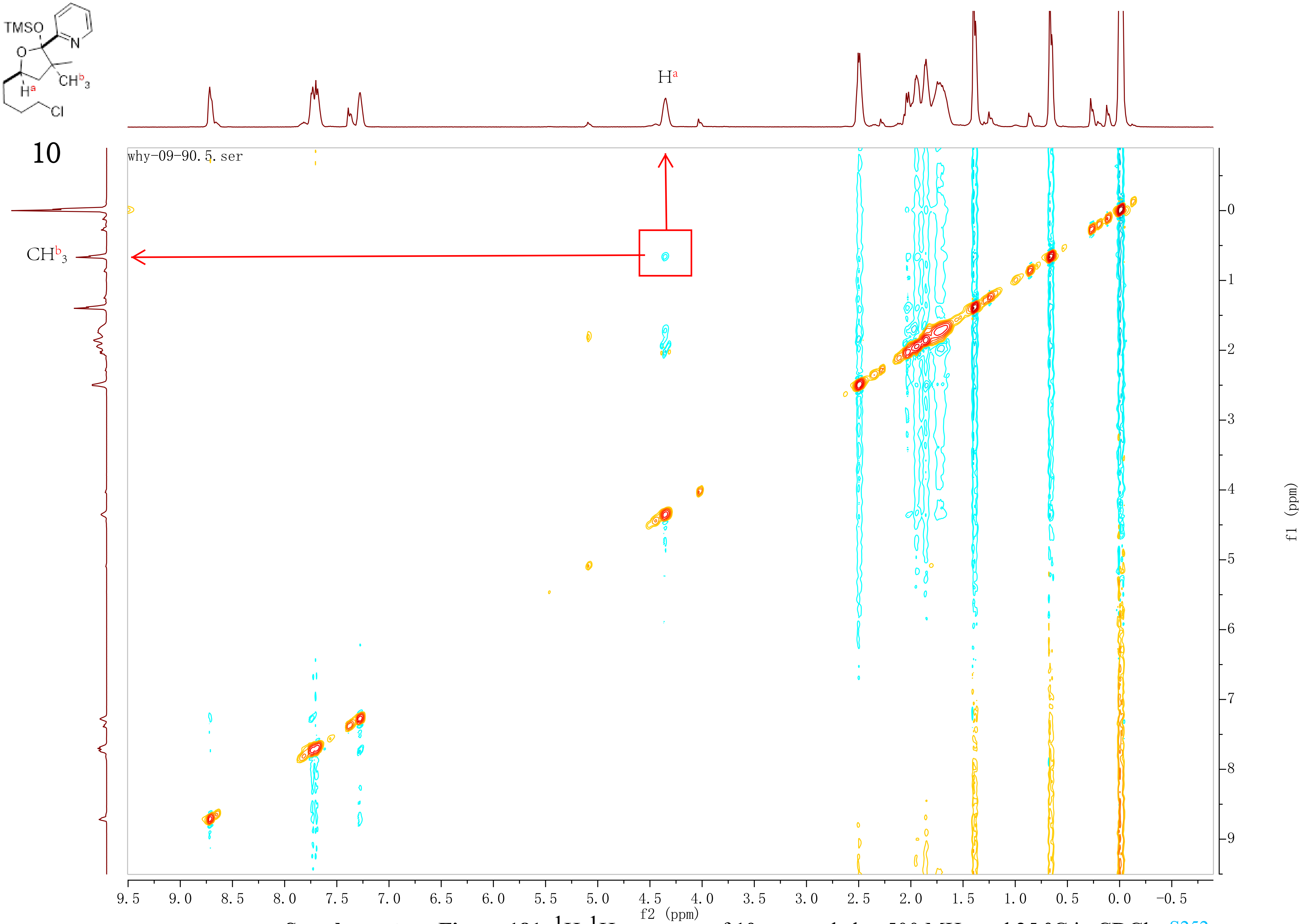

Supplementary Figure 181. ^{1}H-^{1}H spectrum of **10**, recorded at 500 MHz and 25 °C in $CDCl_3$

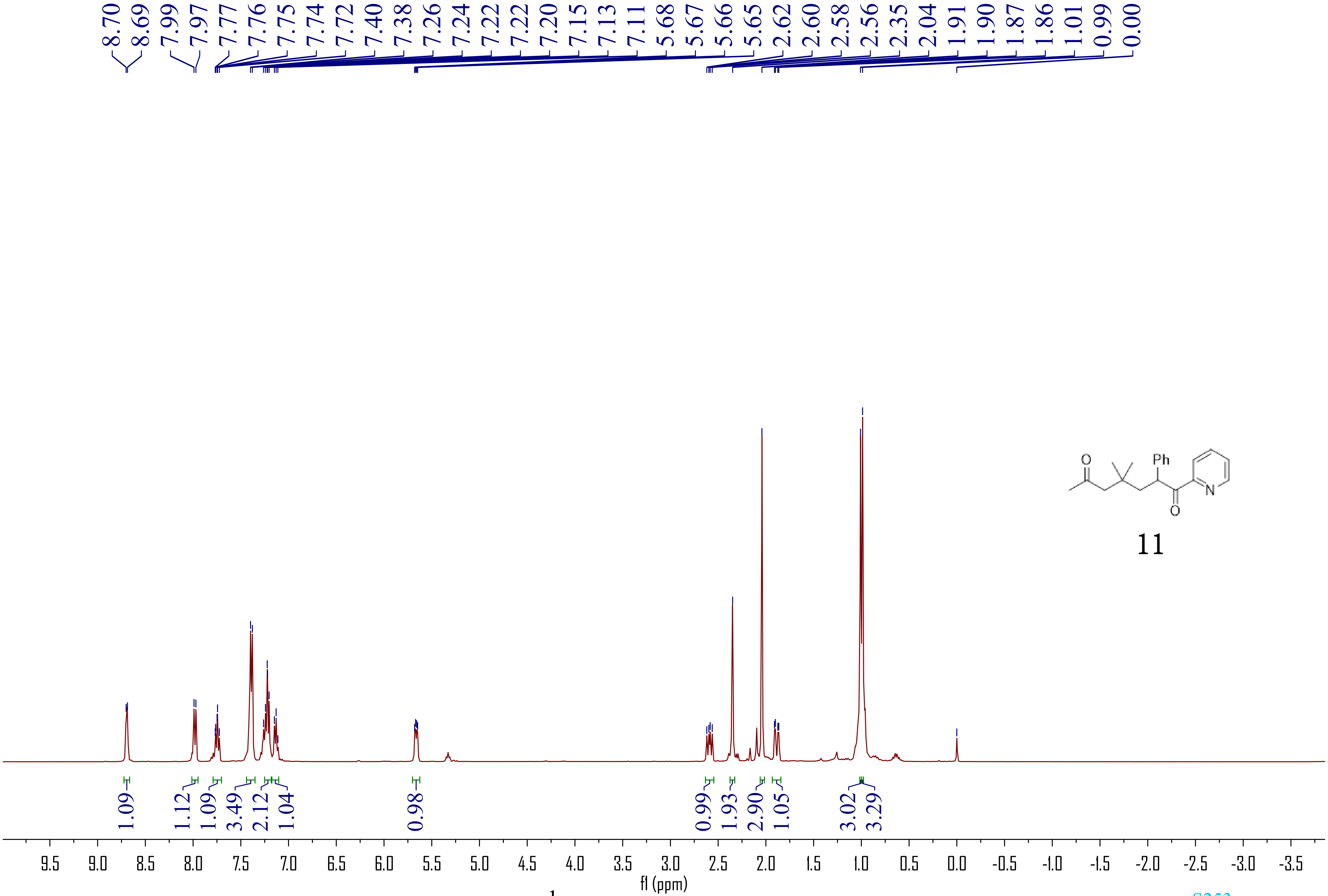

Supplementary Figure S182. ^{1}H NMR spectrum of **11**, recorded at 400 MHz and 25 °C in $CDCl_3$

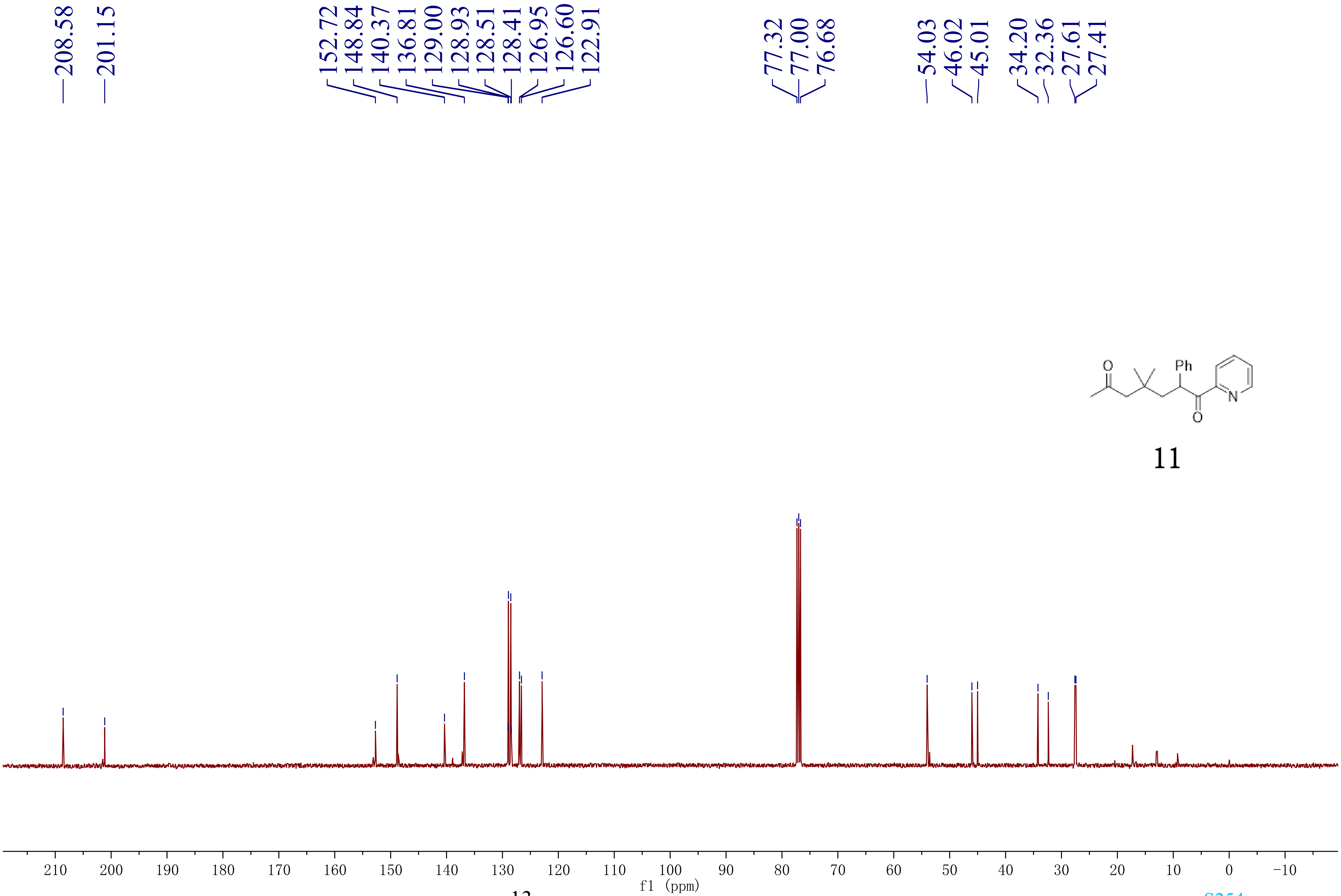

Supplementary Figure 183. ^{13}C NMR spectrum of **11**, recorded at 101 MHz and 25 °C in $CDCl_3$

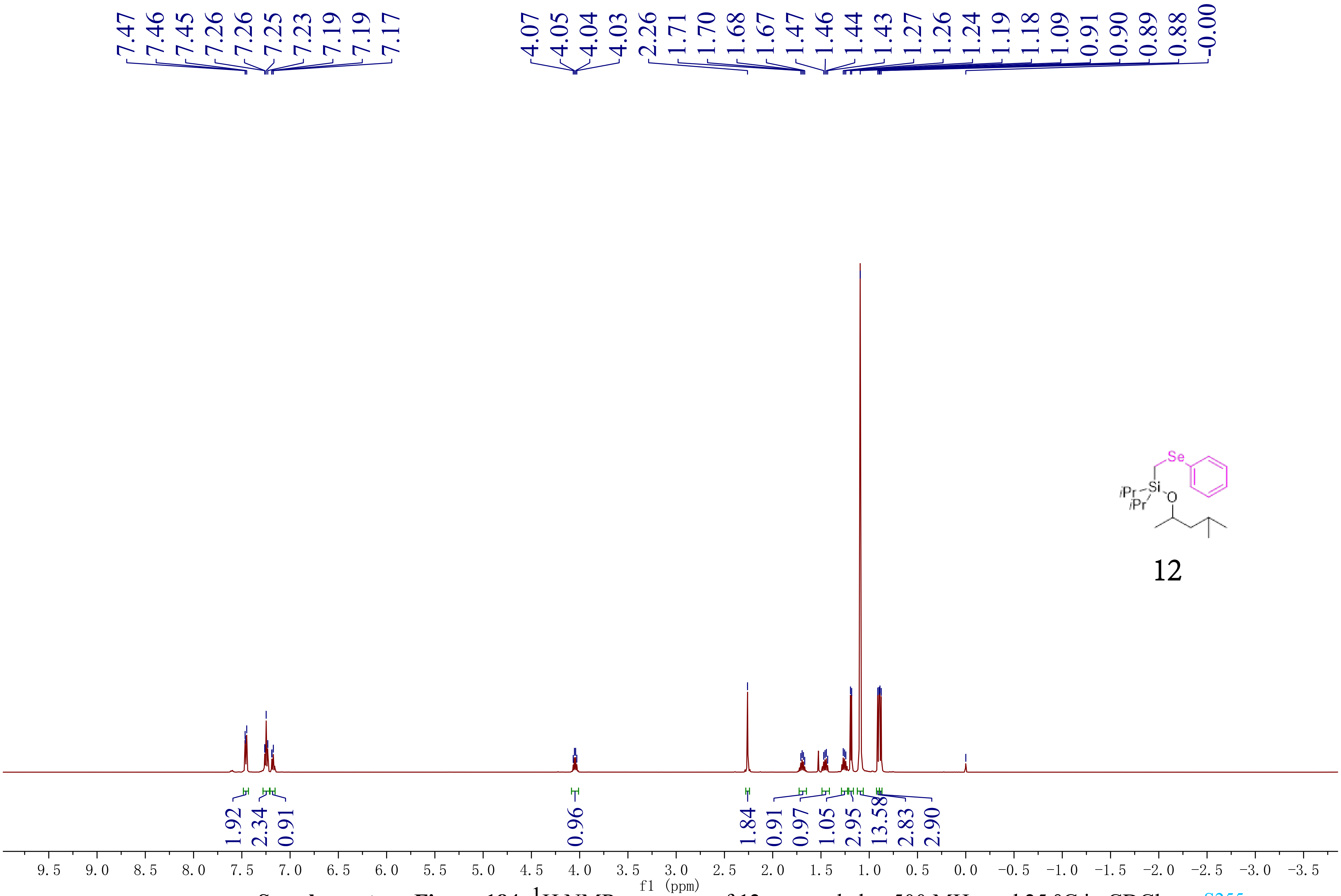

Supplementary Figure 184. ^{1}H NMR spectrum of **12**, recorded at 500 MHz and 25 °C in $CDCl_3$

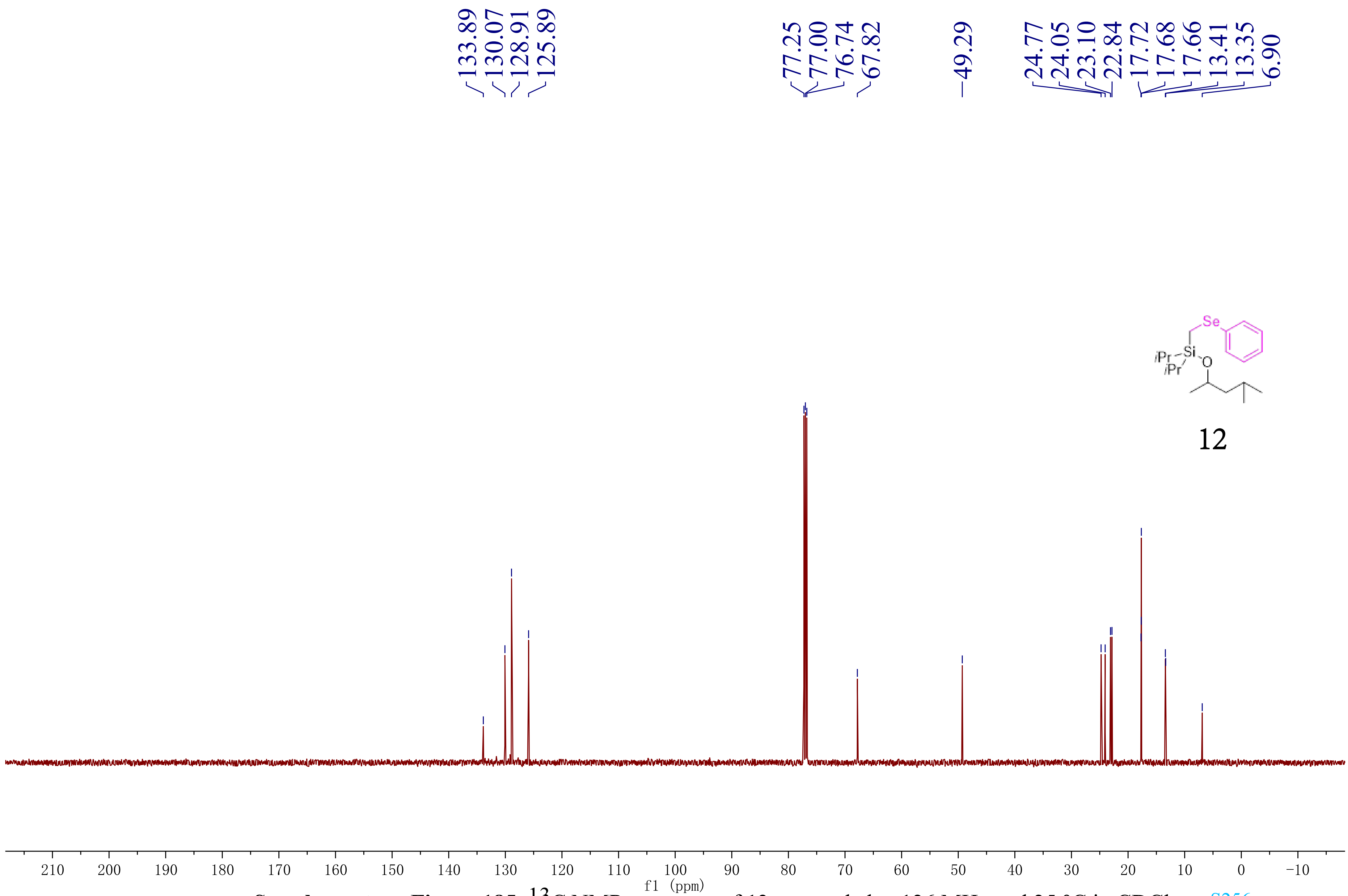

Supplementary Figure 185. ^{13}C NMR spectrum of **12**, recorded at 126 MHz and 25 °C in $CDCl_3$

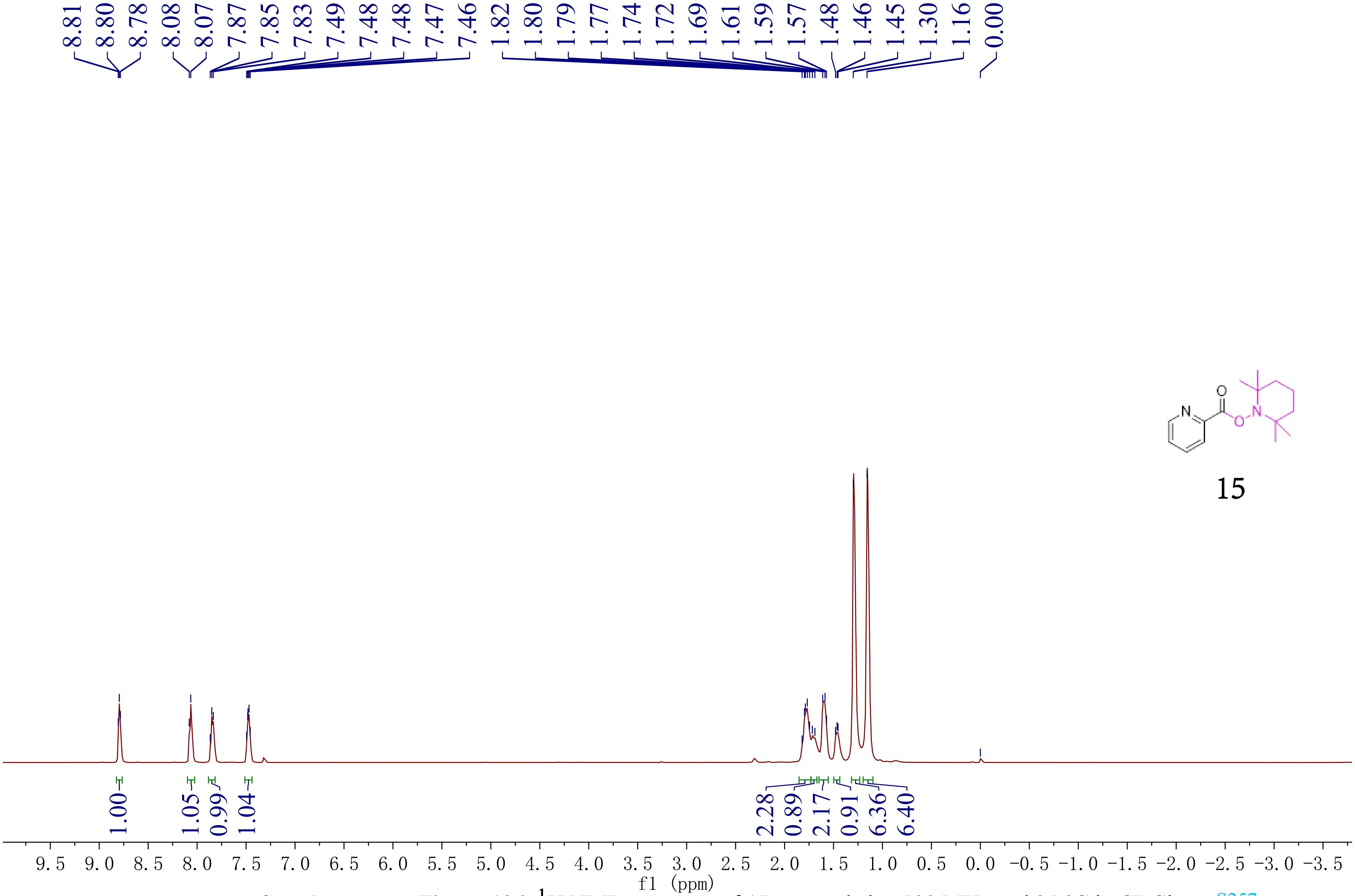

Supplementary Figure 186. ^{1}H NMR spectrum of **15**, recorded at 500 MHz and 25 °C in $CDCl_3$

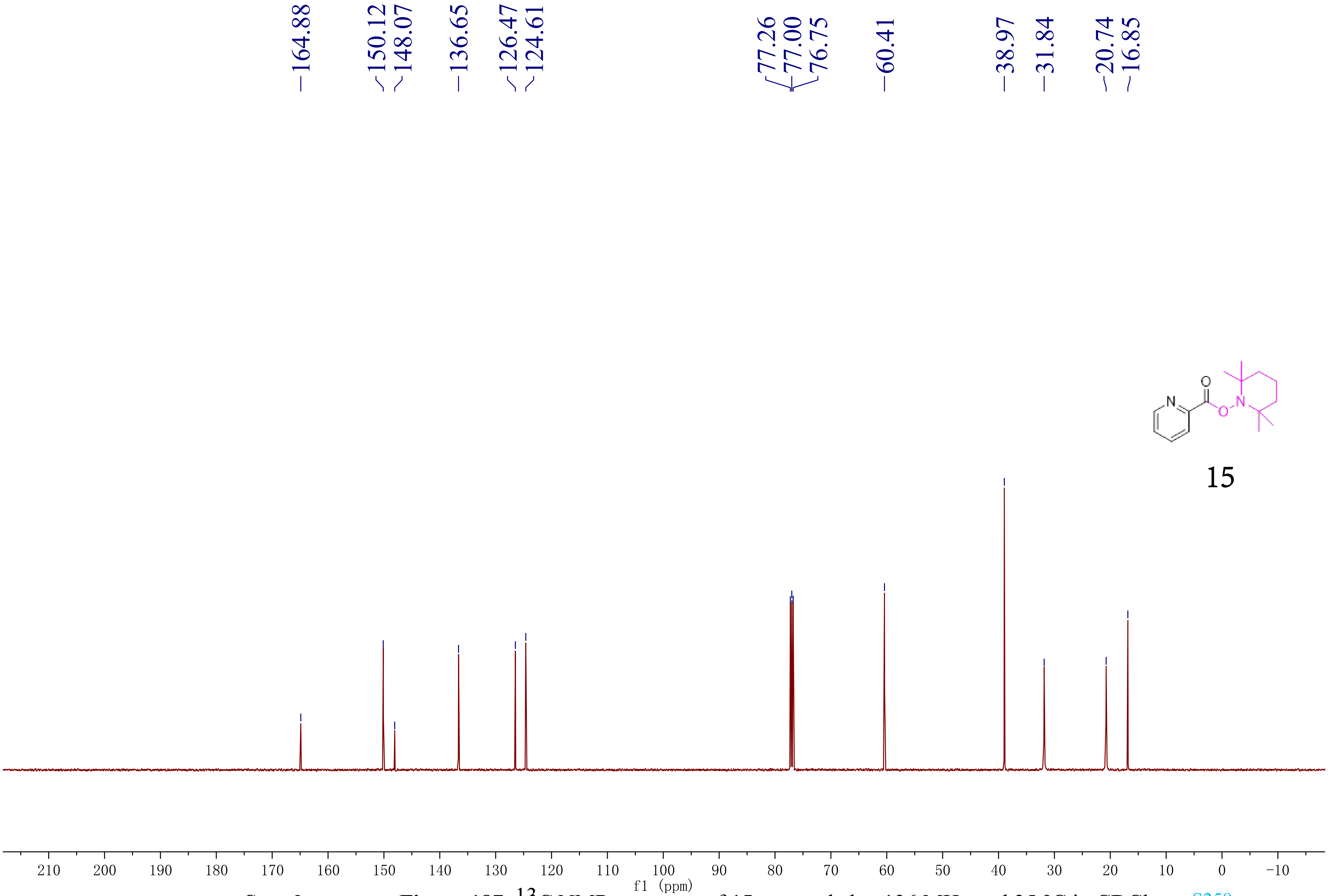

15

Supplementary Figure 187. ^{13}C NMR spectrum of **15**, recorded at 126 MHz and 25 °C in $CDCl_3$

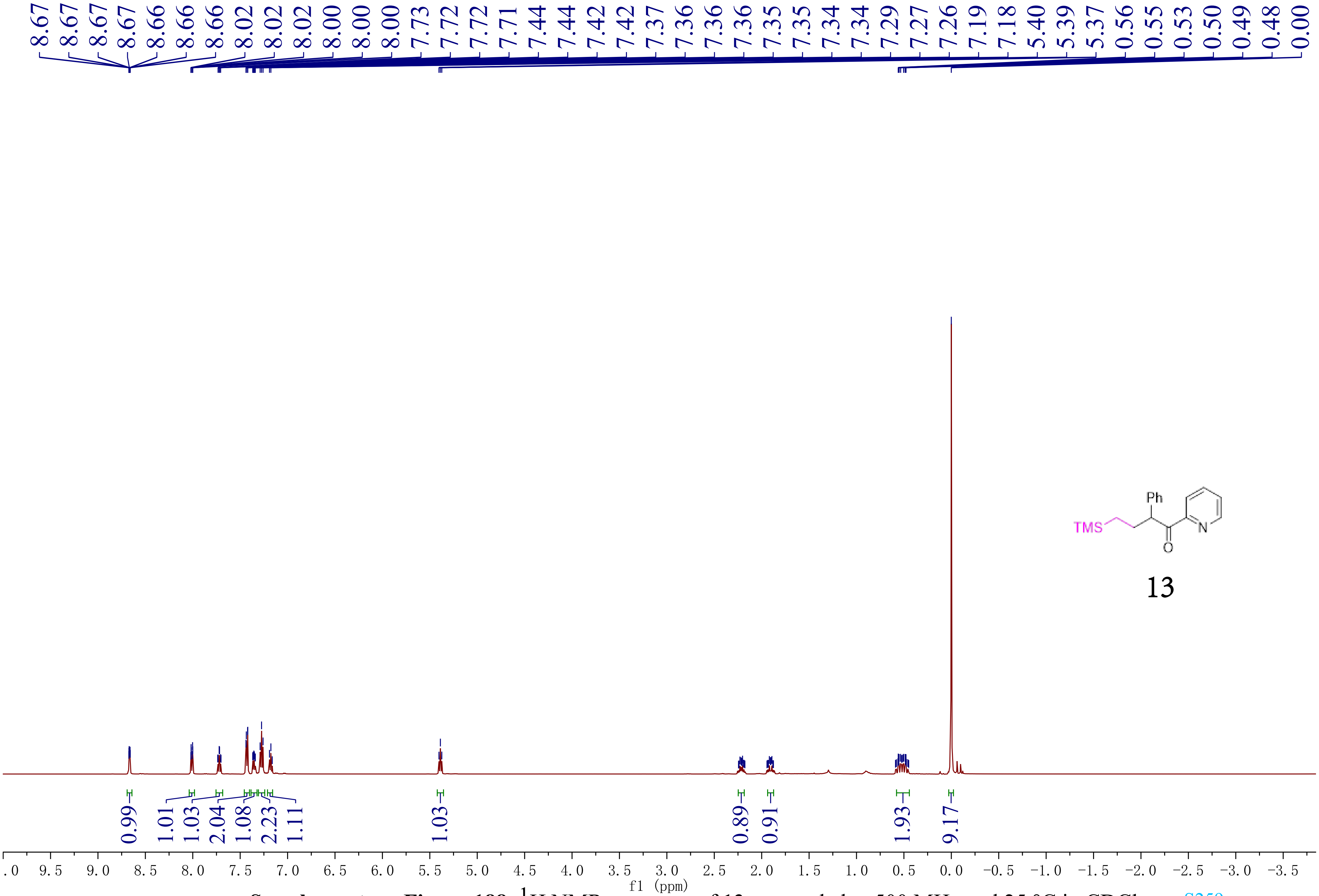

Supplementary Figure 188. ^{1}H NMR spectrum of **13**, recorded at 500 MHz and 25 °C in $CDCl_3$

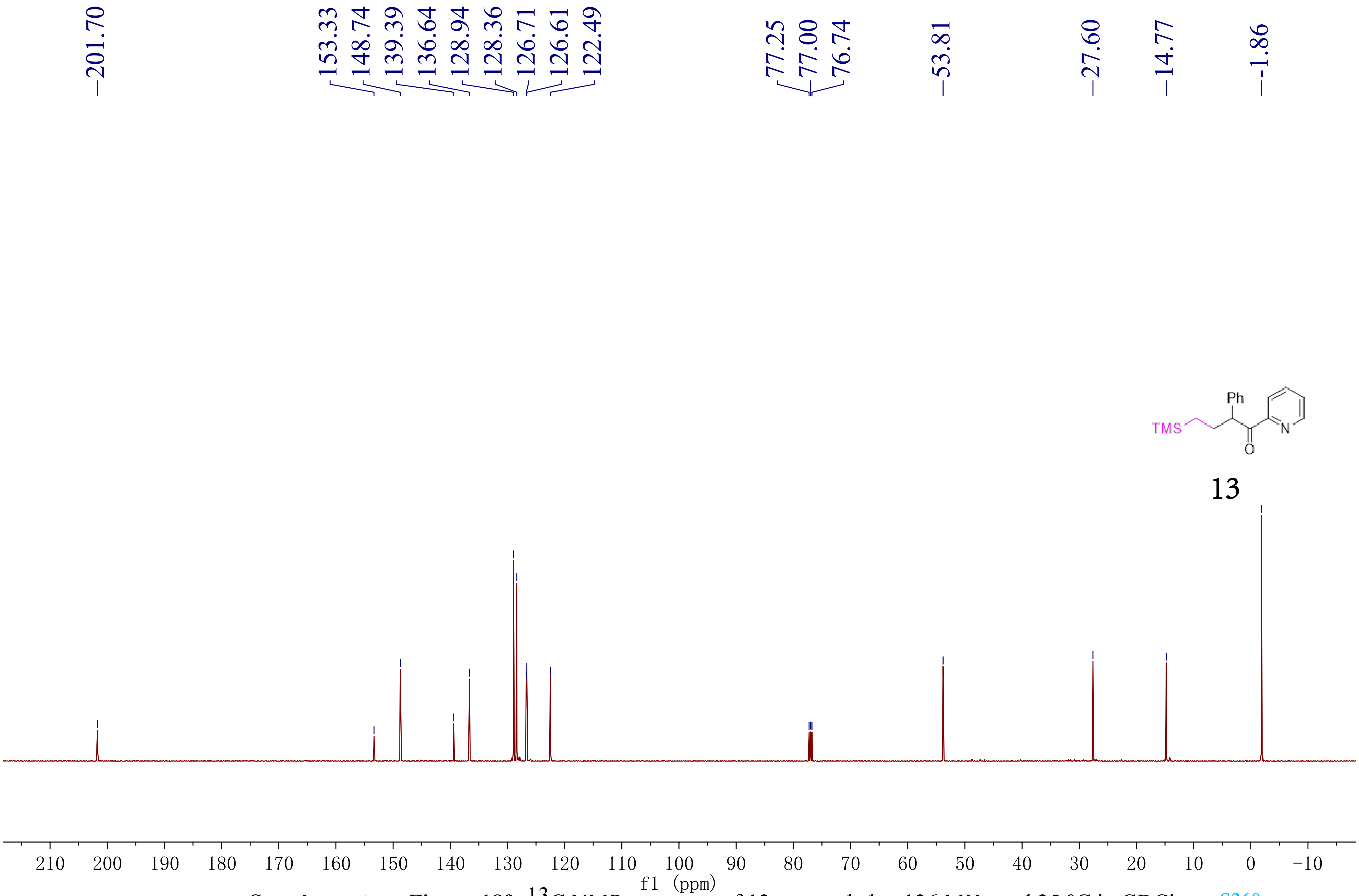

Supplementary Figure 189. ^{13}C NMR spectrum of **13**, recorded at 126 MHz and 25 °C in $CDCl_3$

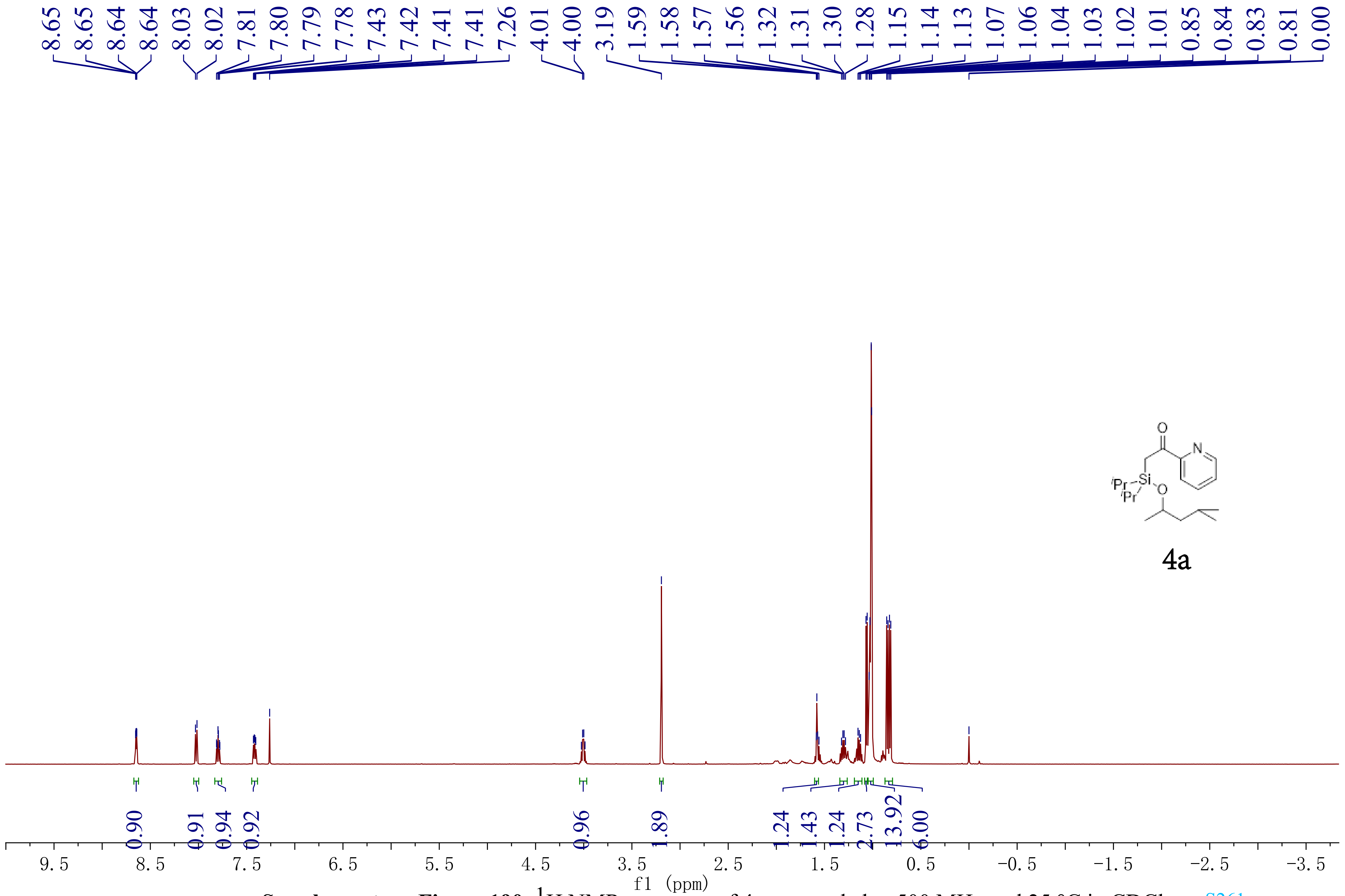

Supplementary Figure 190. ^{1}H NMR spectrum of **4a**, recorded at 500 MHz and 25 °C in $CDCl_3$

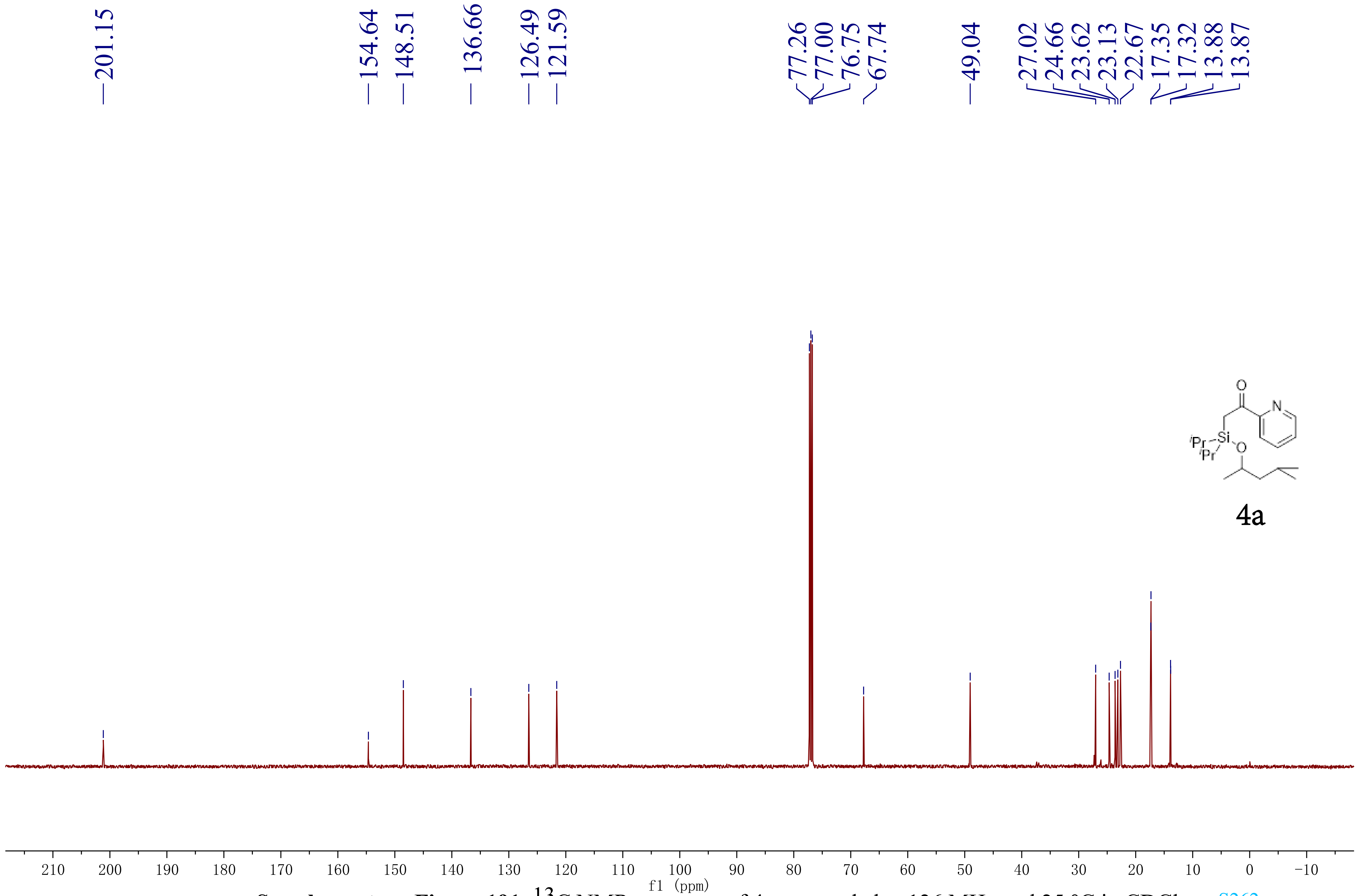

Supplementary Figure 191. ^{13}C NMR spectrum of **4a**, recorded at 126 MHz and 25 °C in $CDCl_3$

4. Supplementary Reference

1 Benhamou, L., Chardon, E., Lavigne, G., Bellemin-Laponnaz, S. & Cesar, V. Synthetic routes to N-heterocyclic carbene precursors. *Chem. Rev.* **111**, 2705-2733, (2011).

2 Marvin, P., Padon, C., Yang, W., Yi, S. & Vladimir, G. General, Auxiliary-Enabled Photoinduced Pd-Catalyzed Remote Desaturation of Aliphatic Alcohols. *J. Am. Chem. Soc.* **139**, 14857-14860, (2017).

3 R, H. G. Fluorinated Paraffins. *J. Org. Chem.* **24**, 1709-1715, (1959).

4 Li, X. Z. *et al.* Synthesis of heterocyclic ring-fused analogs of HMG499 as novel degraders of HMG-CoA reductase that lower cholesterol. *Eur. J. Med. Chem.* **236**, 114323, (2022).

5 Hu, T. J., Zhang, G., Chen, Y. H., Feng, C. G. & Lin, G. Q. Borylation of Olefin C-H Bond via Aryl to Vinyl Palladium 1,4-Migration. *J. Am. Chem. Soc.* **138**, 2897-2900, (2016).

6 Han, Y.-F. *et al.* Photoredox cooperative N-heterocyclic carbene/palladium-catalysed alkylacylation of alkenes. *Nat. Commun.* **13**, 5754, (2022).

7 Fu, M.-C., Shang, R., Zhao, B., Wang, B. & Fu, Y. Photocatalytic decarboxylative alkylations mediated by triphenylphosphine and sodium iodide. *Science* **363**, 1429–1434 (2019).
